# Synthesis of Stereoenriched Piperidines via Chemo-Enzymatic Dearomatization of Activated Pyridines

**DOI:** 10.1021/jacs.2c07143

**Published:** 2022-11-09

**Authors:** Vanessa Harawa, Thomas W. Thorpe, James R. Marshall, Jack J. Sangster, Amelia K. Gilio, Lucian Pirvu, Rachel S. Heath, Antonio Angelastro, James D. Finnigan, Simon J. Charnock, Jordan W. Nafie, Gideon Grogan, Roger C. Whitehead, Nicholas J. Turner

**Affiliations:** † Department of Chemistry, University of Manchester, Manchester Institute of Biotechnology, 131 Princess Street, Manchester M1 7DN, United Kingdom; ‡ Department of Chemistry, University of York, Heslington, York YO10 5DD, United Kingdom; § Prozomix, Building 4, West End Ind. Estate, Haltwhistle NE49 9HA, United Kingdom; ∥ BioTools, Inc., 17546 Bee Line Highway, Jupiter, Florida 33478, United States

## Abstract

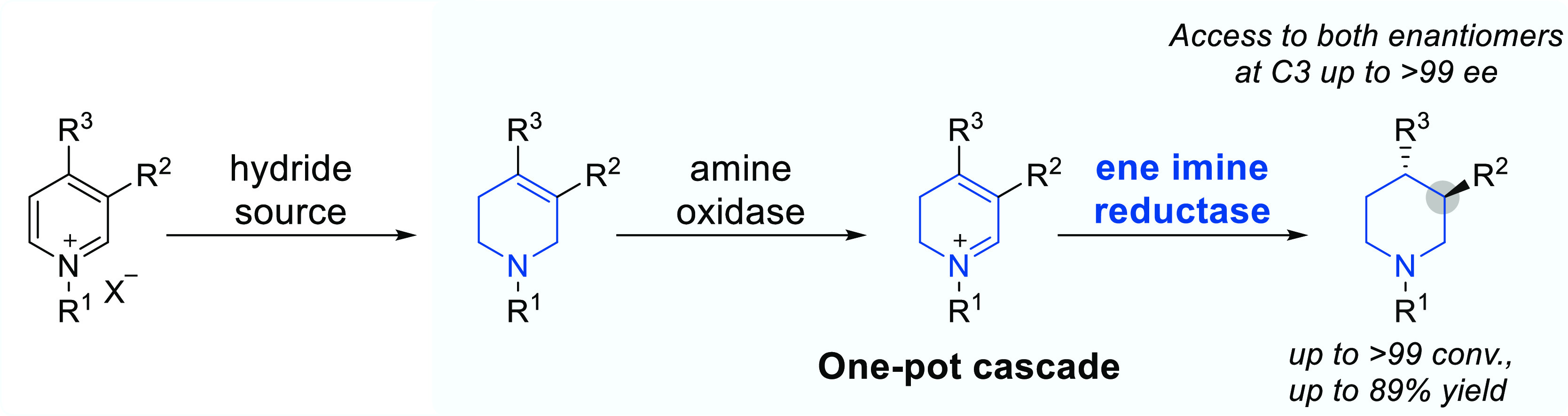

The development of efficient and sustainable methods for the synthesis of nitrogen heterocycles is an important goal for the chemical industry. In particular, substituted chiral piperidines are prominent targets due to their prevalence in medicinally relevant compounds and their precursors. A potential biocatalytic approach to the synthesis of this privileged scaffold would be the asymmetric dearomatization of readily assembled activated pyridines. However, nature is yet to yield a suitable biocatalyst specifically for this reaction. Here, by combining chemical synthesis and biocatalysis, we present a general chemo-enzymatic approach for the asymmetric dearomatization of activated pyridines for the preparation of substituted piperidines with precise stereochemistry. The key step involves a stereoselective one-pot amine oxidase/ene imine reductase cascade to convert N-substituted tetrahydropyridines to stereo-defined 3- and 3,4-substituted piperidines. This chemo-enzymatic approach has proved useful for key transformations in the syntheses of antipsychotic drugs Preclamol and OSU-6162, as well as for the preparation of two important intermediates in synthetic routes of the ovarian cancer monotherapeutic Niraparib.

## Introduction

**Figure 1:**
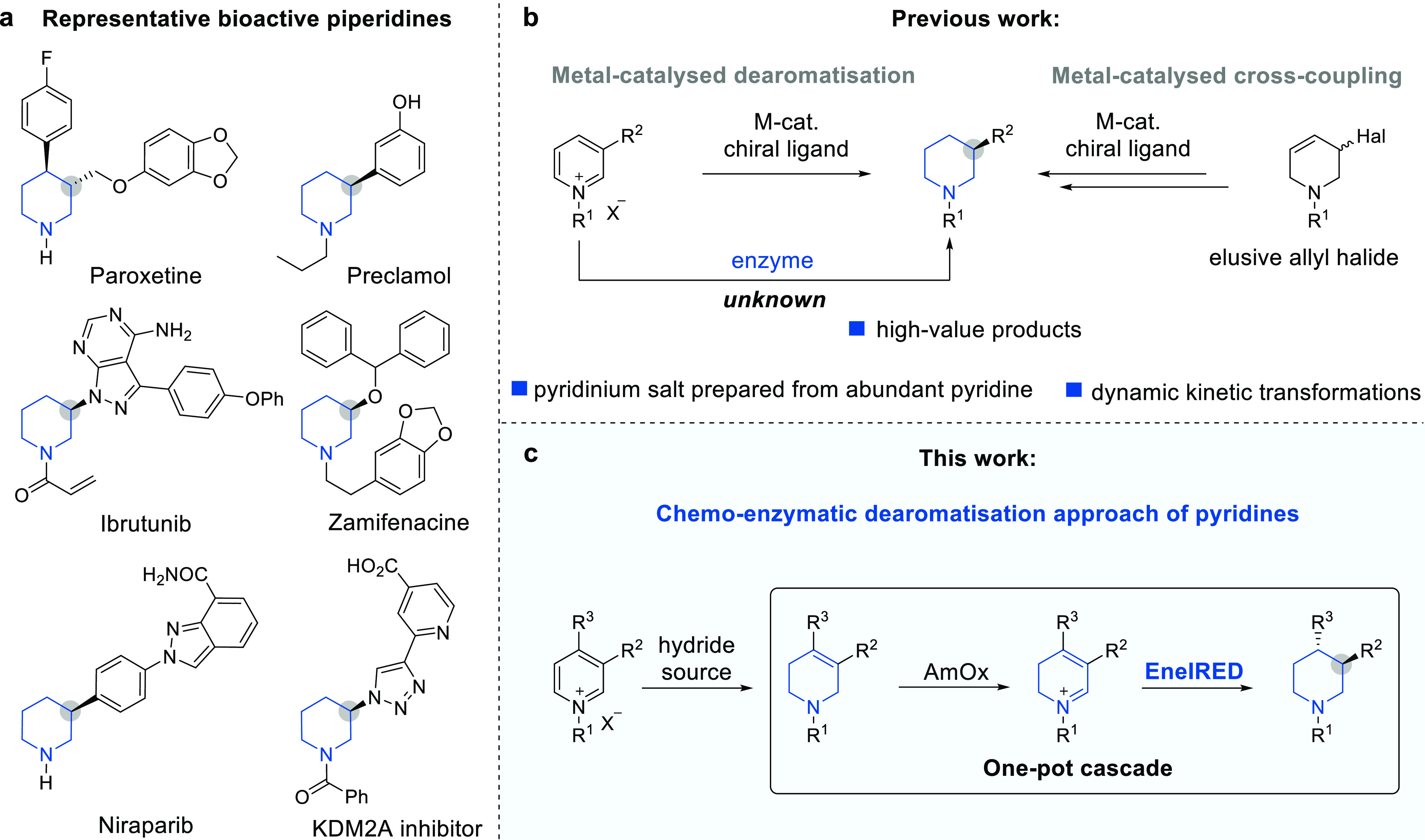
High-value stereo-enriched 3- and 3,4-substituted piperidines and strategies for their synthesis. (a) Representative examples of biologically active chiral substituted piperidines. (b) Previous work: Asymmetric transition-metal-catalyzed synthesis of 3-substituted piperidines. (c) This work: Chemo-enzymatic dearomatization of pyridines for the synthesis of chiral 3- and 3,4-substituted piperidines.

The ubiquity of saturated nitrogen heterocycles (*N*-heterocycles) in natural products and pharmaceuticals continues to drive the development of innovative strategies for their efficient synthesis.^1,2^ In particular, chiral piperidines are much sought after structures due to their prevalence as scaffolds in a range of bioactive molecules including market-approved active pharmaceutical ingredients (APIs).^3^ Nature provides highly efficient biocatalysts for the biosynthesis of *N*-heterocycles,^4,5^ offering high enantio- and regio-selectivity under benign conditions. These biocatalysts have previously enabled the development of one-pot cascade reactions to access stereo-enriched 2-, 2,6-, and 2,3-substituted piperidines.^6−10^ However, the translation of these methods to the corresponding stereoenriched 3-substituted and 3,4-substituted scaffolds, the core of many important therapeutic compounds (Figure 1A), remains challenging due to difficulties in stereoselectivity control combined with limited availability of suitable starting materials.

Asymmetric chemical synthetic approaches for the preparation of 3-substituted and 3,4-disubstituted piperidines include those based on metalation/cross-coupling,^11−14^ Grignard Michael addition,^15^ ring closure,^16^ and transition-metal-catalyzed dearomatization of pyridines.^17−21^ However, limitations are associated with all of these approaches, including high reaction temperatures, sensitivity to moisture, lack of availability of starting materials, and the use of expensive noncommercial chiral ligands.^11,22^ Among reported methods, the catalytic asymmetric dearomatization of pyridines is achieved by quaternization-activation of the pyridine nitrogen, permitting access to mild reduction methods to chiral piperidines (Figure 1B, left).^17,23−26^ Whilst nature has yielded pyridine synthases to prepare pyridines,^27^ an effective biocatalyst for their dearomatization is yet to be discovered. With this in mind, we sought to combine mild chemical reduction of pyridiniums to tetrahydropyridines (THPs) with the exquisite stereoselectivity of a biocatalytic cascade to reduce the final C=C bond as an efficient strategy for asymmetric dearomatization of activated 3- and 3,4-substituted pyridines (Figure 1C). Biocatalysts with broad substrate scope for the reduction of C=C bonds require the conjugation of the alkene to an electron-withdrawing group. Recently, C=C bonds conjugated to C=N bonds have been shown to undergo full reduction to amines through the combination of ene-reductases (EREDs) and imine reductases (IREDs),^6^ as well as the newly discovered ene imine reductase (EneIREDs).^28^ We reasoned that biocatalytic oxidation, using an amine oxidase

(AmOx), of the THP in situ would generate the corresponding dihydropyridiniums (DHPs), generating an activated C=C bond conjugated to the C=N bond, which could then be reduced with these biocatalysts to generate a cascade to the desired 3- and 3,4-substituted piperidines. This cascade complements a previous amine oxidase AmOx-IRED deracemization processes in which only amine oxidation and C=N bond reduction take place.^29,30^

## Results and Discussion

A series of substituted *N-*alkyl THPs **1b**-**21b** was prepared in good yields (50–90%) from activated pyridines (**1a-21a**) using NaBH_4_ as previously reported.^31^ Initially, we explored the conversion of THPs to piperidines using AmOxs in combination with EREDs or EneIREDs (see Supporting Information 2.1.; Figures S1–S5 for the complete list of THPs screened). For the first step, we tested AmOx variants that have been shown to be effective biocatalysts for the oxidation of *N-*alkyl THPs.^32,33^ The 6-hydroxy-D-nicotine oxidase (6-HDNO) variant, E350L/E352D,^34^ was found to be effective, with a broad substrate scope, including oxidation of **1b**, a precursor to Preclamol. We next screened for activity for the second step, namely, reduction of the C=C bond of the α,β-unsaturated iminium ion. Whereas the panel of EREDs displayed no activity, the EneIRED from an unidentified *Pseudomonas* sp. (EneIRED-01),^28^ in combination with 6-HDNO, was effective at reducing a number of THPs and could be used to prepare piperidine (*R*)-**1c** in good yield and with excellent enantioselectivity (see Supporting Information 2.1., Table S1; entry 1–3, ≥42% yield, 96% ee).

Next, we set out to identify further EneIREDs that could also generate enantioenriched 3-substituted piperidines. By screening the recently reported metagenomic IRED collection,^35^ in combination with the 6-HDNO variant, we were able to quickly identify biocatalysts capable of generating either enantiomer of piperidine (*R*/*S*)-**1c** from THP **1b** (see Supporting Information 2.2., Table S2). From this screen, we organized these EneIREDs into two groups: Series A (red: EneIREDs 01–04) that gave piperidine (*R*)-**1c** (Table S2 up to >99% ee) and Series B (blue: EneIREDs 05–09) that generated the enantiocomplementary piperidine (*S*)-**1c** (Table S2, up to 96% ee).

With effective EneIREDs for the preparation of both enantiomeric series, we probed the substrate scope of the 6-HDNO-EneIRED cascade (Table 1). Enzymes in Series A and B accepted a variety of aryl substituents at the C-3-position of the THP scaffold, affording products **1c**-**7c** in high yields, conversion, and enantioselectivity. Five-membered heterocyclic 3-substituents such as furan **8c** and thiophene **9c** were also well tolerated (≥62% yield, ≥86% ee). Sterically demanding substrates, for example, containing a 2-naphthyl substituent **10b**, were also tolerated producing (*R*)-and (*S*)-**10c** in excellent yield and stereoselectivity (73% yield, >99% conv., ≥94% ee). Additionally, a variety of *N*-alkyl substituents were accepted forming the piperidine products **1c**-**12c** in good to excellent yields. Of these, *N*-allyl **3c**-**4c** and *N*-propargyl **12c** piperidines provide useful synthetic handles that can be easily removed or further functionalized.

**Table 1:**
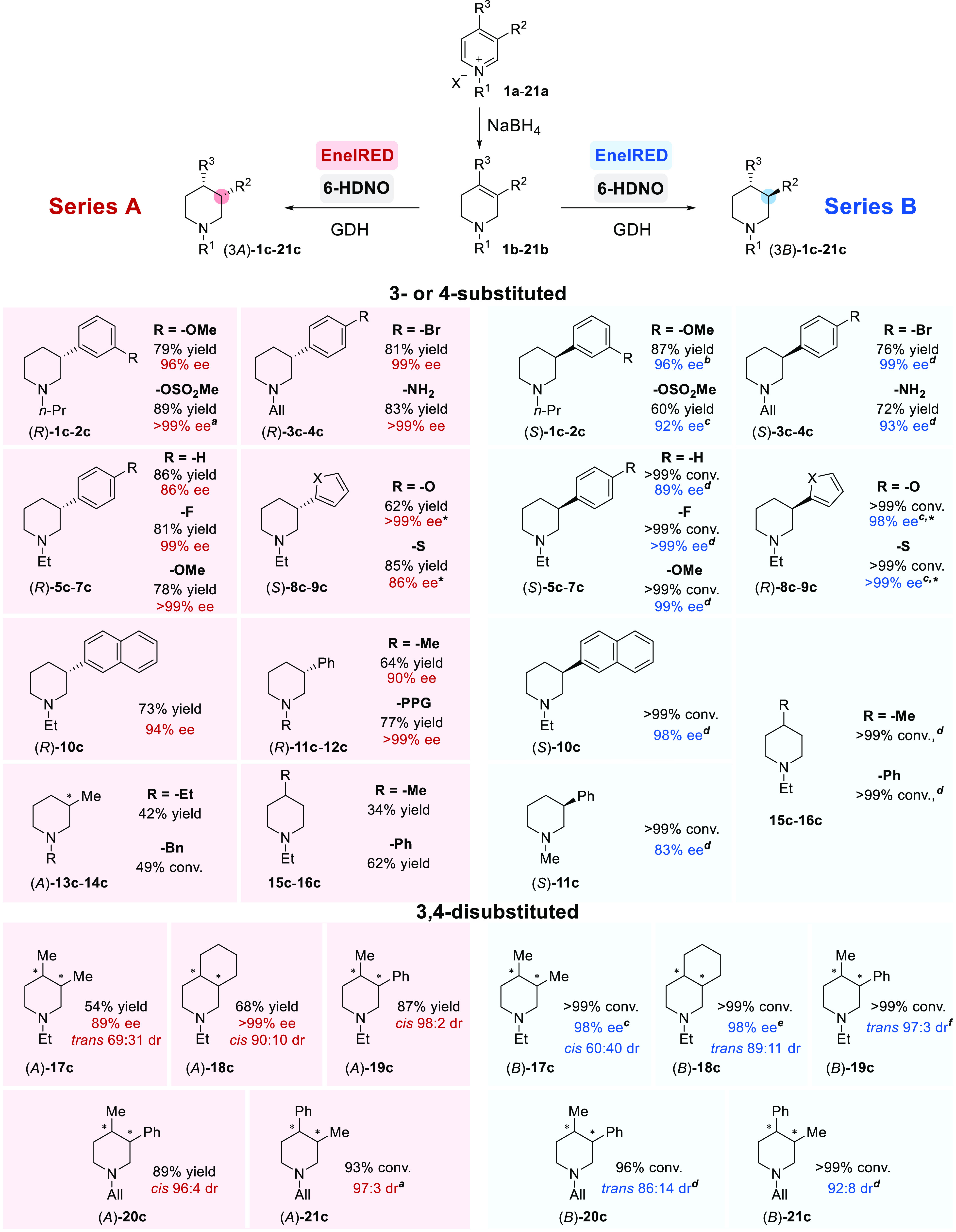
Scope of Chemo-Enzymatic Dearomatization of Activated Pyridines^a^

a Series A and Series B provide enantiocomplementary stereopreference at C-3. All examples use EneIRED-01 except ^a^EneIRED-02, ^b^EneIRED-05, ^c^EneIRED-06, ^d^EneIRED-07, ^e^EneIRED-08, and ^f^EneIRED-09. *Switch in the Cahn-Ingold-Prelog (CIP) priority. Enantiomeric excess (ee) was determined by chiral high-performance liquid chromatography, supercritical fluid chromatography (SFC), and gas chromatography. See Supporting Information 4 for more details on the absolute configuration determination.

More hindered 3,4-disubstitituted THPs could also be reduced using the cascade, resulting in the formation of the substrate-dependent *cis* or *trans* isomer. These included substrates in which both substituents were simple alkyl (3,4-dimethyl) **17b** or part of a fused bi-cyclic ring system (octahydroisoquinoline) **18b**. We then probed the tolerance for a combination of alkyl and aryl substituents at C-3 and C-4. 3-Phenyl-4-methyl disubstituted compounds **19b** and **20b** provided the corresponding piperidines (*A*)-**19c** and (*A*)-**20c** in excellent yields and diastereoselectivity (*cis* major; >96:4 dr; ≥87% yield). The system also tolerated the isomeric 3-methyl-4-phenyl disubstituted THP **21c**. In addition to the inversion of stereochemical outcome at the C-3 position (Series A vs Series B), we also discovered some EneIREDs in Series B that provided an inverted diastereomeric configuration of the 3,4-disubstitituted piperidines **17c-20c** compared to Series A.

**Figure 2:**
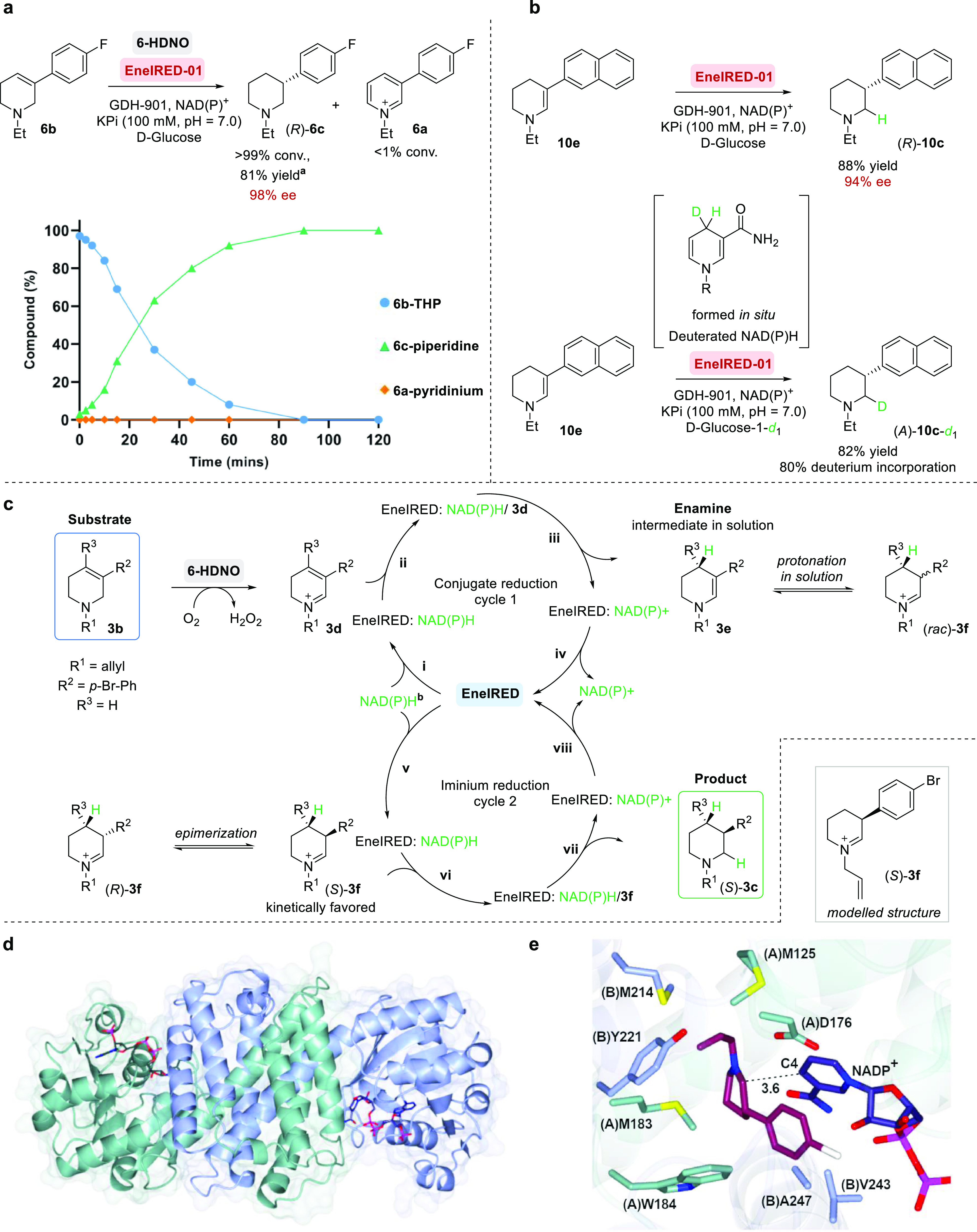
Proposed mechanism for the 6-HDNO-EneIRED cascade. (a) Kinetic profile from in situ ^19^F NMR reaction monitoring of THP-**6b**. ^*a*^Reactions run at 1 mmol. (b) Enamine **10e** is used to probe the role of this species as an intermediate in the cascade. (c) Proposed catalytic sequence for the AmOxEneIRED biocatalytic cascade. ^*b*^Two equivalents of NAD(*P*)H consumed to form piperidine. (d) Structure of the dimer of EneIRED-07 in the ribbon format with subunits shown in green and blue. NADP^+^ can be seen bound at the two active sites. (e) Active site of EneIRED-07 with the (*S*)-enantiomer of iminium ion intermediate **3f** modeled into the active site.

To probe the mechanism of the 6-HDNO-EneIRED cascade, the conversion of THP **6b** to piperidine **6c** was investigated by in situ ^19^F NMR reaction monitoring (Figure 2A). After <5 min, formation of piperidine **6c** was apparent and after 60 min, the THP **6b** was completely consumed. In the absence of the EneIRED, 6-HDNO catalyzed the previously reported aromatization of THP **6b** to the corresponding pyridinium ion **6a** (see Supporting Information 2.3., Figure S6, >99% conversion after 24 h).^33^ As expected, no direct reduction of the THP **6b** to piperidine **6c** was observed in the absence of 6-HDNO, suggesting that a transiently generated dihydropyridinium (DHP) is the substrate for the EneIRED reduction. Using the cascade with THP-**10b**, we were able to isolate the enamine 1**0e** before full conversion to the piperidine **10c** (see Supporting Information 5.1., 63% yield), which strongly suggested the participation of this compound in the reaction pathway. Accordingly, enamine **10e** was converted to piperidine **10c** using EneIRED-01 alone, in excellent yield and high enantioselectivity, equivalent to the full cascade with THP-**10b** (Figure 2B, top; 88% yield, 94% ee). Deuterium labeling experiments were also implemented to further elucidate the mechanism of the EneIRED enamine reduction step. Carrying out the reduction of enamine **10e** using EneIRED-01, in the presence of GDH and d-glucose-1-*d*_1_ to generate deuterated NAD(*P*)D in situ, C-2-mono-deuterated **10c-*d*_*1*_** was formed (80% deuterium incorporation, 82% yield). This suggests that the enamine intermediate **10e** may undergo protonation to the iminium before NAD(*P*)H hydride delivery (Figure 2B, bottom and see Supporting Information 5.2.).

A proposed mechanism for the 6-HDNO-EneIRED cascade is outlined in Figure 2C. For illustrative purposes, we have depicted the transformation of THP-**3b** to (*S*)-**3c** as this product is used in the subsequent synthesis of the key intermediate for Niraparib described below. Initially, 6-HDNO oxidation of THP-**3b** results in the activation of the THP C=C bond for EneIRED-catalyzed asymmetric conjugate reduction of DHP-**3d** at the expense of NADPH to generate enamine **3e** (Cycle 1; step iii). This intermediate **3e** is expected to be in equilibrium with chiral iminium **3f** via a nonselective protonation in solution, which has been extensively documented.^9,36,37^ Depending on the EneIRED employed (Series A or B), the kinetic selective reduction of one enantiomer of chiral iminium **3f** affords the enantioenriched piperidine **3c** as the final product via reduction with a second molecule NADPH (Cycle 2; step vii). In situ epimerization of the enantiomer in **3f** via enamine **3e** enables a dynamic kinetic resolution (DKR) to occur to generate piperidine (*S*)-**3c** mediated by the EneIRED.

Predominantly, EneIREDs in Series A yielded (*R*)-piperidines whilst enzymes in Series B such as EneIRED-07 yielded the (*S*)-product. In order to gain insight into the mode of substrate binding in the active site, we determined the structure of EneIRED-07 from *Micromonospora* sp. Rc5 to a resolution of 2.55 Å in complex with NADP^+^ using X-ray crystallography. Crystals were obtained in the *P*2_1_2_1_2 space group and featured six molecules in the asymmetric unit, representing three dimers. EneIRED-07 displays the canonical fold observed for IREDs,^38,39^ with two monomers associating to form two active sites between the *N*-terminal Rossmann domain of one subunit and the *C*-terminal helical bundle of its neighbor (Figure 2D). Analysis using the DALI server^40^ suggested that the IRED with the most closely related structure was the IRED from *Streptosporangium roseum* (PDB 5OCM) with an rmsd of 1.0 Å over 288 Cα atoms. Following building and refinement of the protein atoms, clear omit density was observed in each active site corresponding to the cofactor NADP^+^. The iminium intermediate (*S*)-**3f**, the preferred enantiomer for EneIRED-07 imine reduction, was modeled into the active site using Autodock Vina (Figure 2E).^41^ The model suggests that the allyl group of (*S*)-**3f** is bound within a hydrophobic pocket formed by methionine residues M125, M183, and M214 at the rear of the active site as shown; the *para*-bromo-phenyl group projects toward the front of the active site bordered by L180, W184, and the NADP^+^ cofactor. This ligand conformation places the electrophilic carbon approximately 3.6 Å from the NADP^+^ pyridinium ring C4 atom, from which hydride is transferred. Modeling of the (*R*)-enantiomer of **3f** places the allyl group at the base of the active site with less favorable interactions with hydrophilic residues Y225 and D238 (see Supporting Information 7.4., Figure S13).

The absolute configuration of **6c**-**10c** was verified using VCD (Vibrational Circular Dichroism), a technique available for the determination of stereochemical configuration of chiral molecules in the solution phase.^42−45^ This was accomplished by the comparison of experimental infrared (IR) and VCD spectra to density functional theory (DFT)-calculated spectra of a specific configuration. Because this series of molecules was of low molecular weight with a limited number of low energy conformations (fewer than 10 in each case), four different DFT methods were completed for each compound. We tested two functionals (B3LYP and B3PW91) each with two basis sets (6-31G(d) and cc-pVTZ) to see which would have the best statistical results in each case.^46^ As expected, all five piperidine methods yielded consistent results for each enantiomer, with the best results coming from cc-pVTZ/B3PW91 for piperidines **6c** and **9c**, cc-pVTZ/B3LYP for piperidines **7c** and **8c**, and 6-31G(d)/B3PW91 for piperidine **10c**. Neighborhood similarity values for IR and VCD, as well as confidence level (≥93%) were obtained using BioTools (Jupiter, Fl) CompareVOA software (see Supporting Information 4.3., and 12.).^47,48^ Because of the similarity of the chiral core, the VCD experimental spectra were very similar for all tested compounds. Future work could therefore forego the calculations in order to streamline the process.

**Figure 3:**
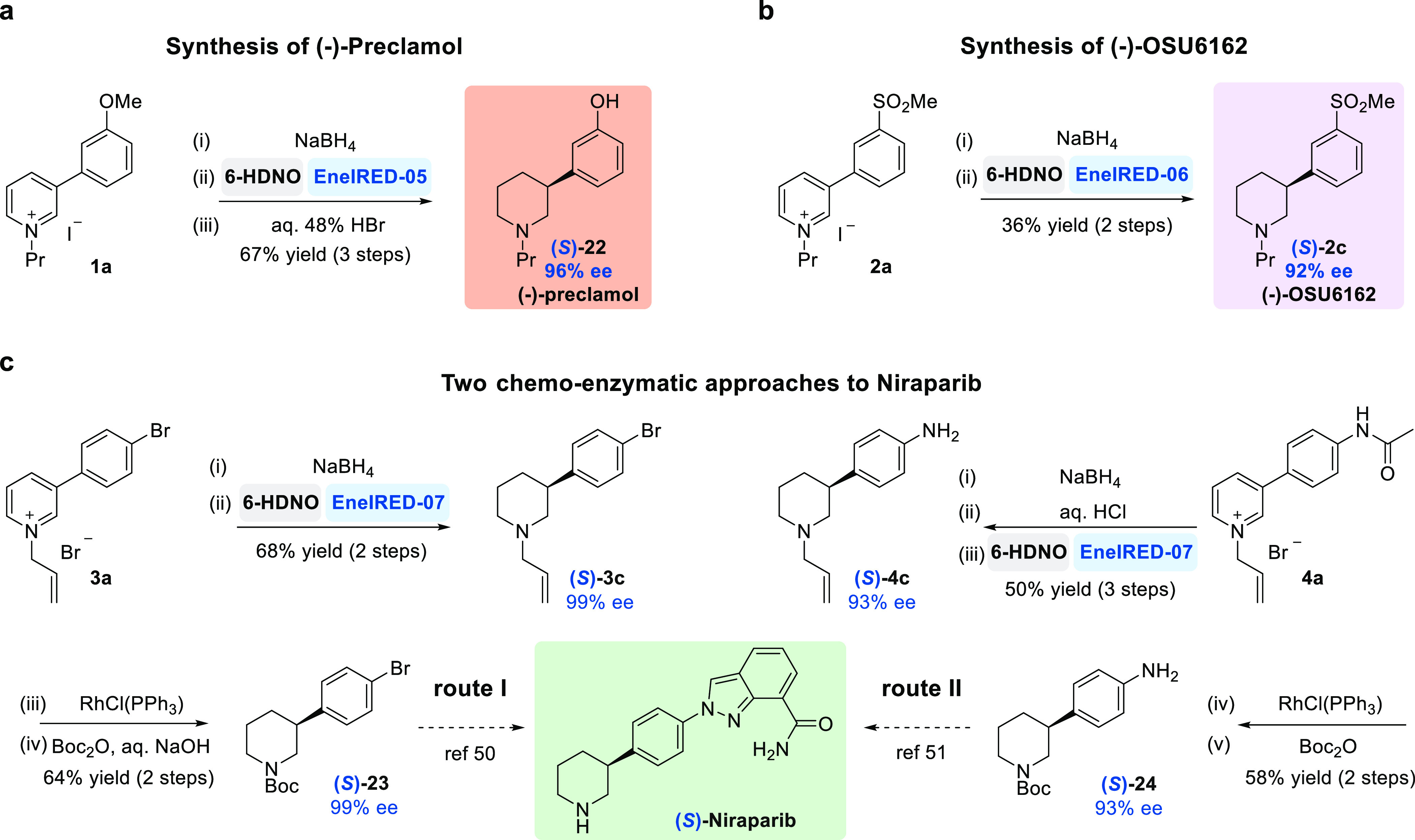
Application of the chemo-enzymatic dearomatization of pyridines for the preparation of APIs. (a) Synthesis of the antipsychotic drug (−)-preclamol. (b) Synthesis of (−)-OSU6162. (c) Two synthetic routes to Niraparib.

Finally, we sought to apply the chemo-enzymatic dearomatization of activated pyridines to several target bioactive molecules (Figure 3). First, we targeted the antipsychotic drug Preclamol. At preparative scale (1 mmol), THP-**1b** was converted to both (*R*)-(+)- and (*S*)-(−)-preclamol **22**, using EneIRED-01 and EneIRED-05, respectively. Both enantiomers were prepared in four steps from 3-(3-methoxyphenyl)pyridine and were obtained in ≥50% overall yield and with 96% ee (Figure 3A and see Supporting Information 5.1.). Next, we carried out the three-step syntheses of both enantiomers of OSU6162 **2c**, using EneIRED-02 and EneIRED-06, and these were both accomplished in ≥36% overall yield and ≥92% ee (Figure 3B), respectively.

To further demonstrate the application of the cascade, we synthesized the two intermediates **23** and **24** en route to Niraparib (Figure 3C), the first poly ADP ribose polymerase (PARP) inhibitor to be approved as a first-line monotherapeutic for the maintenance treatment of patients with advanced ovarian cancer.^49^ For route **I**, we showed that commercially available 3-(4-bromophenyl)pyridine could be efficiently converted to piperidine (*S*)-**3c** in just three steps and 61% overall yield (99% ee). This was followed by deallylation and *N*-Boc-protection to yield (*S*)-**23** in 64% yield, a key intermediate in Merck’s second-generation synthesis.^50^ Alternatively, in route **II**, by starting from commercially available 4-(pyridin-3-yl)aniline, we converted pyridinium salt **4a** to (*S*)-**24** in 29% overall yield and with 93% ee, a key intermediate in Merck’s first-generation synthesis.^51^ The general applicability of the method was also showcased by the preparation of the corresponding (*R*)-enantiomers of both **23** and **24** in good yields and high enantioselectivity (see Supporting Information 5.1.).

In summary, we report the development of a versatile and highly efficient chemo-enzymatic dearomatization of activated pyridines for the preparation of stereo-enriched 3- and 3,4-disubstituted piperidines. The 6-HDNO-catalyzed oxidation of readily accessible THPs facilitates EneIRED-catalyzed conjugate reduction and iminium reduction to yield a broad range of chiral piperidines. The short syntheses of both enantiomers of Preclamol and OSU6162, as well as chiral precursors to Niraparib, highlight the flexibility and utility of the method presented, emphasizing the advantages of combining chemical synthesis with biocatalysis for developing new catalytic methods for the preparation of important chiral compounds. Furthermore, the increasing ability to systematically screen large panels of biocatalysts against new targets leads to the rapid identification of enzymes with applications in asymmetric synthesis.

## Supporting Information Available

Experimental procedures including characterization of compounds, spectroscopic data of analytical biotransformations, and control experiments (PDF)
