# Supplementary material for: Synthesis of Stereoenriched Piperidines via Chemo-Enzymatic Dearomatization of Activated Pyridines
Source: J Am Chem Soc. 2022 Nov 9;144(46):21088–95. doi: 10.1021/jacs.2c07143 (PMC9706572; doi:10.1021/jacs.2c07143)
Supplement: Supplementary file 1 — ja2c07143_si_001.pdf [file ja2c07143_si_001.pdf]

Supplementary Information for

Synthesis of stereoenriched piperidines via chemo-enzymatic dearomatization of activated pyridines

Vanessa Harawa, Thomas W. Thorpe, James R. Marshall, Jack J. Sangster, Amelia K. Gilio, Lucian Pirvu, Rachel S. Heath, Antonio Angelastro, James D. Finnigan, Simon J. Charnock, Jordan W. Nafie, Gideon Grogan, Roger C. Whitehead, Nicholas J. Turner*.

*Corresponding author. Email: Nicholas.Turner@manchester.ac.uk

This PDF file includes:

Materials and Methods

Supplementary Text

Figs. S1 to S64

Tables S1 to S13

NMR Spectra

References

Table of Contents

1. General Analysis 4
2. Biotransformations 6
   2.1. Time course screens with AmOx variants 6
   2.2. Screening 384 metagenomic IREDs panel protocol 9
   2.3. Biocatalytic cascade control experiments 10
3. General procedure for biotransformations 14
4. Absolute Configuration Determination 16
   4.1. Commercial optically pure products: Analysis 16
   4.2. Literature comparison: Analysis 16
   4.3. VCD: Analysis 16
5. Preparative-Scale Procedure and Product Characterisation 17
   5.1. General Method 1: preparative-scale procedure 17
   5.2. Deuterium Labelling Experiments 35
6. Cloning, expression, and purification protocols of biocatalysts 39
   6.1. Amine oxidases 39
   6.2. IREDs 40
   5.2.1. EneIRED Amino Acid Sequence Information 42
7. Protein Crystallization of EneIRED-07; Data Collection and Refinement..44
   7.1. Gene expression and protein purification 44
   7.2. Protein crystallization 44
   7.3. Data collection, structure solution and refinement 44
   7.4. Modelling 45
8. GCMS Analysis: methods and retention times for THP substrates and piperidine products 48
9. Chiral HPLC Analysis: methods and retention times for piperidine products..81
10. Chiral GC Analysis: methods and retention times for piperidine products 92
11. Chiral SFC Analysis: methods and retention times for piperidine products 96
12. VCD Analysis: methods for piperidine products 98
13. Substrate synthesis and characterisation 147
   13.1. General 147
      13.1.1. General Method 2: Suzuki–Miyaura Coupling for the preparation of pyridines 147
      13.1.2. General Method 3: *N*-alkylation for the preparation of pyridinium salts (**1a**-**21a**) 150
      13.1.3. General Method 4: Pyridinium salt reduction for the preparation of THPs (**1b**-**21b**).160

13.2. General Method 5: Hydrogenation of THP for the preparation of racemic standards 171

14. Compound ^{1}H NMR and ^{13}C NMR Spectra 181

1. General Analysis

Solvents used were of HPLC grade and when necessary solvents were further dried over molecular sieves. Column chromatography was performed on silica gel (Fluka (Buchs, Switzerland), 220-440 mesh). Spectra from ^{1}H and ^{13}C NMR runs were recorded on a Bruker Avance 400 and 500 instruments (400 MHz for ^{1}H and 100 MHz for ^{13}C; 500 MHz for ^{1}H and 126 MHz for ^{13}C) in $CDCl_3$ or CD_3OD or $(CD_3)_2SO$ using residual protic solvent as an internal standard. Reported chemical shifts (δ) (in parts per million (ppm)) are relative to the residual protic solvent signal ($CHCl_3$ in $CDCl_3$, ^{1}H = 7.26; ^{13}C = 77.0; CHD_2OD in CD_3OD, ^{1}H = 3.31; ^{13}C = 49.0; $(CHD_2)_2SO$ in $(CD_3)_2SO$, ^{1}H = 2.50; ^{13}C = 39.52). High-resolution mass spectrometry (HRMS) was recorded using a Waters LCT time-of-flight mass spectrometer, connected to a Waters Alliance LC (Waters, Milford, MA, USA). Data were processed with Waters Masslynx software.

Determination of optical rotations was performed on an AA-100 polarimeter at 25°C with the solvent and concentration stated. Chiral normal phase High Performance Liquid Chromatography (HPLC) was performed on an Agilent system (Santa Clara, CA, USA) equipped with a G1379A degasser, G1312A binary pump, a G1367A well plate autosampler unit, a G1316A temperature-controlled column compartment and a G1315C diode array detector. CHIRALPAK®IA, CHIRALPAK®IC and CHIRALPAK®IE Analytical (all Daicel (Osaka, Japan), 250mm length, 4.6 mm diameter, 5 µm particle size) as well as CHIRALCEL®OD-H and CHIRALCEL®AD-H Analytical (Daicel (Osaka, Japan), 250 mm length, 4.6 mm diameter, 5 µm particle size) columns were used. The typical injection volume was 10 µL and chromatograms were monitored at 265 nm. All solvent mixtures are given in (v/v) ratios.

Chiral Supercritical Fluid Chromatography (SFC) screening and optimization was carried out using a Waters Acquity UPC2 system (Waters Corp, Milford, MA, USA). This system contained a photodiode array detector, column managers to allow six orthogonal columns to be run in series, a sample manager, and a fluid delivery module (liquid CO_2 pump as well as a modifier pump). The system was controlled by MassLynx software.

Gas Chromatography (GC) analysis was performed on an Agilent 6850 GC (Agilent, Santa Clara, CA, USA) with a flame ionization detector (FID) and autosampler. Columns used include a 25 m CP-Chirasil-DEX CB column with 0.25 mm inner diameter and 0.25 µm film thickness (Agilent, Santa

Clara, CA, USA); a 30 m βDEX-325 with 0.25 mm inner diameter and 0.25 µm film thickness (Supeclo, Bellefont, PA, USA); a 30 m HP-1MS column with 0.32 mm inner diameter and 0.25 µm film thickness (Agilent, Santa Clara, CA, USA) and an HP-1 column with 0.32 mm inner diameter and 0.25 µm film thickness (Agilent, 6 Santa Clara, CA, USA). Gas chromatography–mass spectrometry (GCMS) analysis was performed on a HP-6890 Series GC coupled to a HP5973 MS detector, EI positive mode with helium as the carrier gas.

2. Biotransformations

2.1. Time course screens with AmOx variants

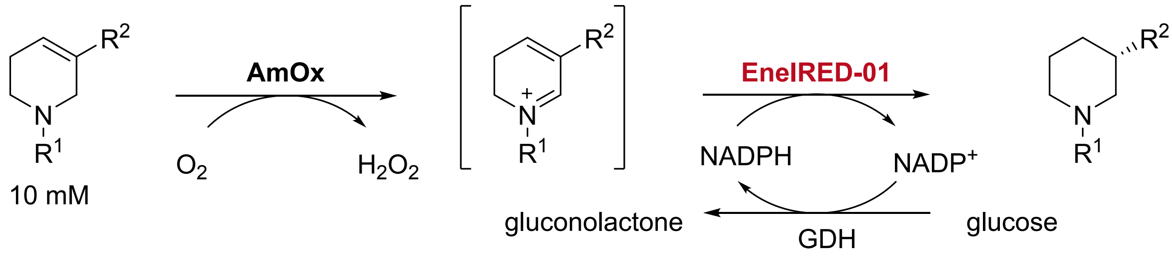

A 500 µL reaction mixture contained D-glucose (50 mM), glucose dehydrogenase (GDH) (0.1 mg/mL, Codexis CDX-901), $NADP^+$ (0.5 mM), EneIRED-01 CFE (4 mg/mL), purified amine oxidase (AmOX) (0.5 mg/mL stored in 100 mM KPi pH 7.8) and THP (1.00 mmol, stock conc. = 1 M in dimethyl sulfoxide (DMSO)). The reaction volume was made up to 500 µL with KP_i buffer (100 mM, pH 7.0). Reactions were incubated at 30 °C with shaking at 200 r.p.m. for 0-16 h, after which they were quenched by the addition of 40 µL of 10 M NaOH and extracted once with 500 µL *tert*-butyl methyl ether (MTBE), dried over anhydrous $MgSO_4$ and analysed on GCMS.

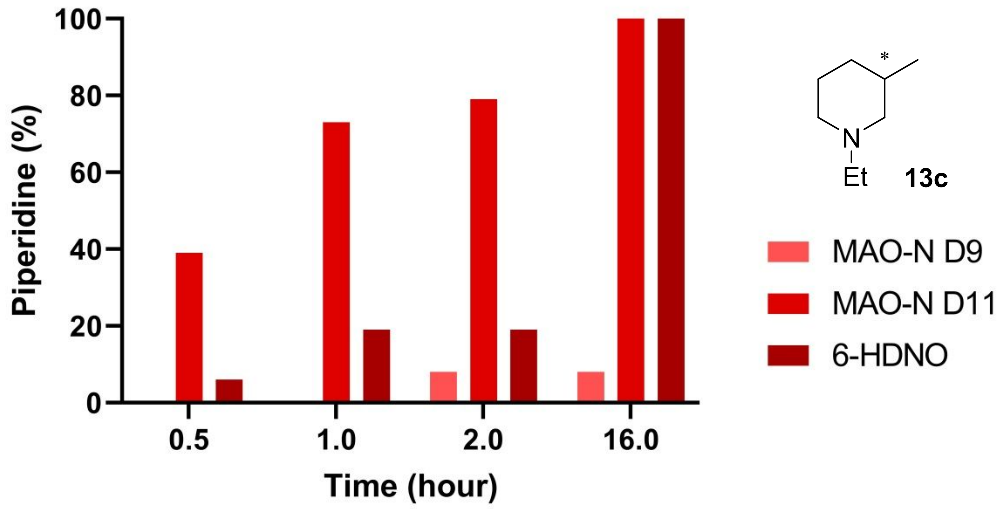

Figure S1: Comparison of AmOx variants. Reaction conditions: 10 mM **13b**, purified AmOx (0.5 mg/mL), EneIRED-01 CFE (4 mg/mL), glucose (50 mM), $NADP^+$ (0.5 mM), GDH-901 (0.1 mg/mL), 100 mM KPi pH 7.0, 30 °C, 200 r.p.m.

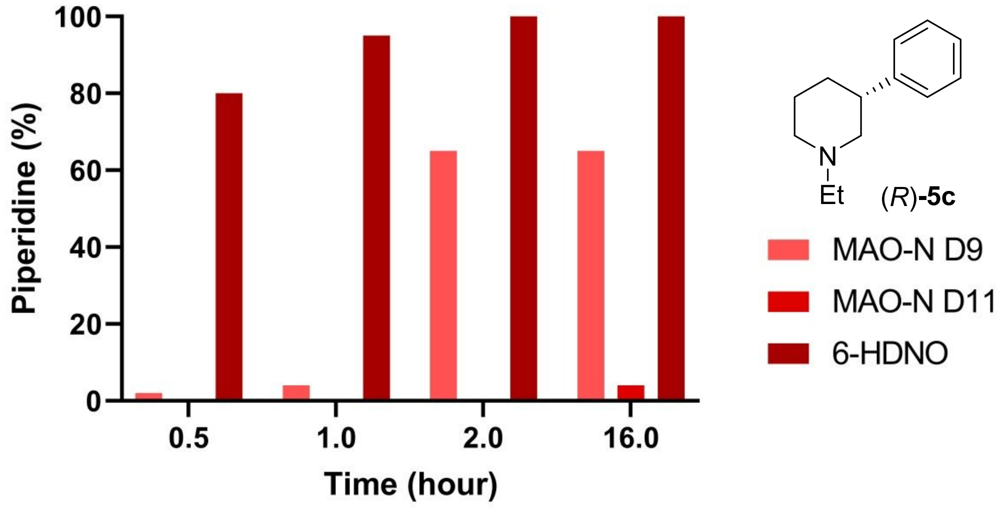

Figure S2: Comparison of AmOx variants. Reaction conditions: 10 mM **5b**, purified AmOx (0.5 mg/mL), EneIRED-01 CFE (4 mg/mL), glucose (50 mM), $NADP^+$ (0.5 mM), GDH-901 (0.1 mg/mL), 100 mM KPi pH 7.0, 30 °C, 200 r.p.m.

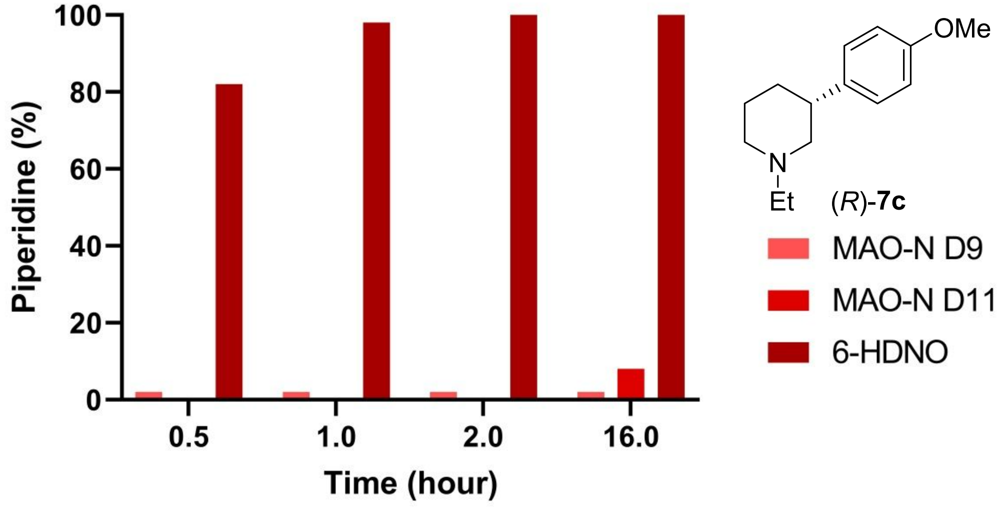

Figure S3: Comparison of AmOx variants. Reaction conditions: 10 mM **7b**, purified AmOx (0.5 mg/mL), EneIRED-01 CFE (4 mg/mL), glucose (50 mM), $NADP^+$ (0.5 mM), GDH-901 (0.1 mg/mL), 100 mM KPi pH 7.0, 30 °C, 200 r.p.m.

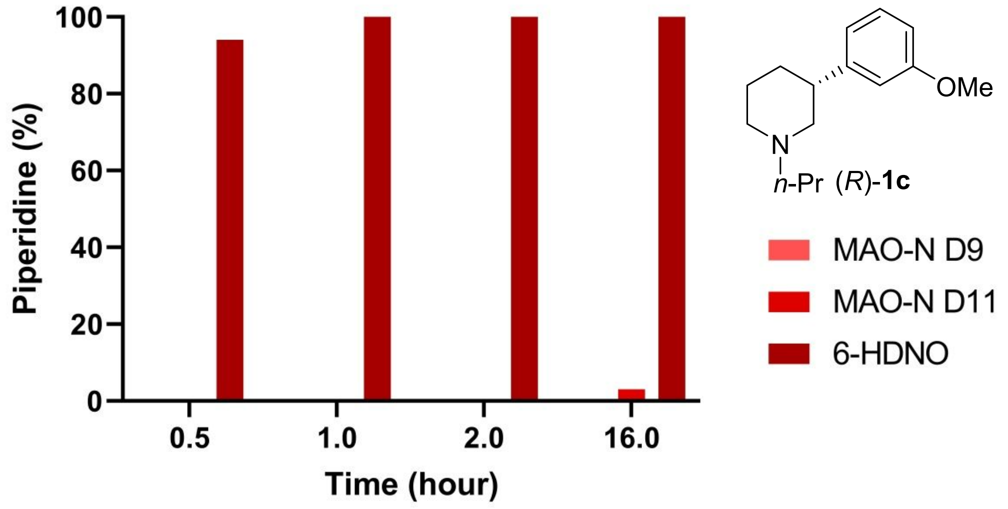

Figure S4: Comparison of AmOx variants. Reaction conditions: 10 mM **1b**, purified AmOx (0.5 mg/mL), EneIRED-01 CFE (4 mg/mL), glucose (50 mM), NADP$^+$ (0.5 mM), GDH-901 (0.1 mg/mL), 100 mM KPi pH 7.0, 30 °C, 200 r.p.m.

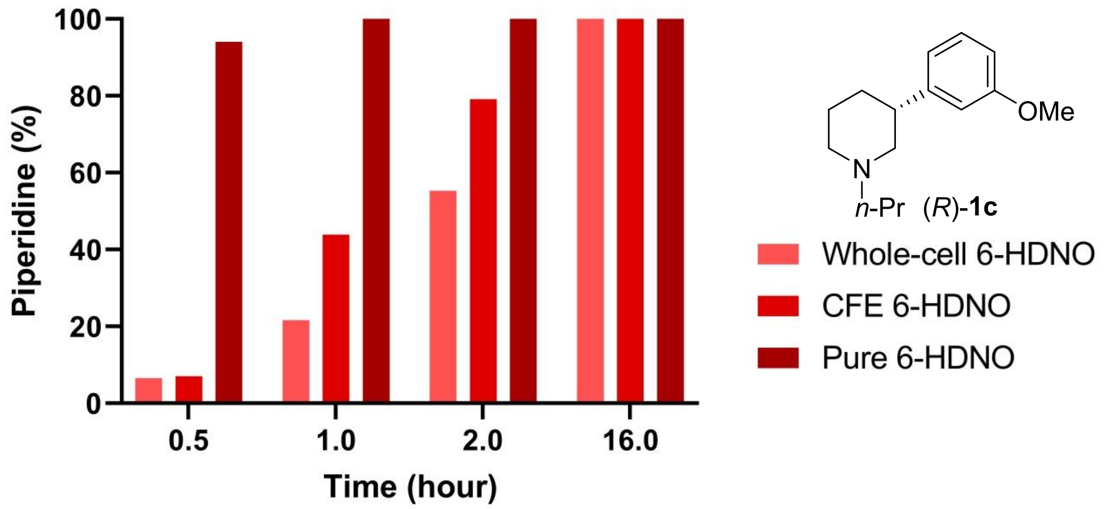

Figure S5: Comparison of AmOx variants. Reaction conditions: 10 mM **1b**, Whole-cell 6-HDNO (100 mg/mL) or 6-HDNO CFE (25 mg/mL) or pure 6-HDNO (0.5 mg/mL), EneIRED-01 CFE (4 mg/mL), glucose (50 mM), NADP$^+$ (0.5 mM), GDH-901 (0.1 mg/mL), 100 mM KPi pH 7.0, 30 °C, 200 r.p.m.

Table S1: Preliminary AmOx studies.

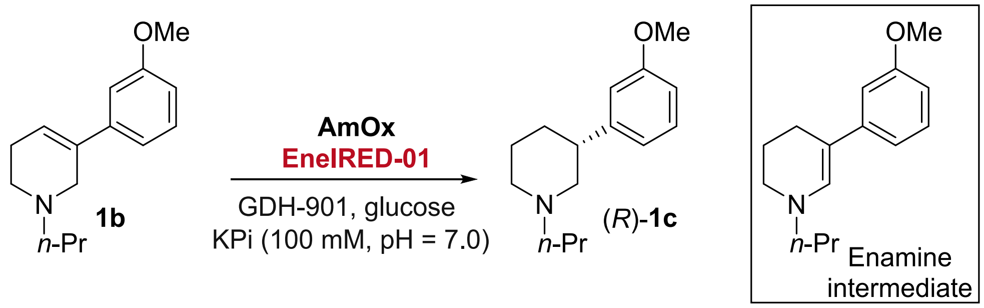

Entry	AmOx	Yield of 2 (%)[a]	ee (%)
**1**	Purified 6-HDNO	79	96
**2**	6-HDNO CFE	60	96
**3**	Whole-cell 6-HDNO	42[b]	96
**4**	Purified MAO-N D9	<1	_
**5**	Purified MAO-N D11	<1	_

AmOx enzyme screen. [a]Reaction conditions (preparative scale): Whole-cell AmOx (200 mg/mL) or CFE AmOx (50 mg/mL) or pure AmOx (1 mg/mL), EneIRED-01 CFE (10 mg/mL), 50 mM **1b** (1 mmol), glucose (250 mM), GDH (0.5 mg/mL), $NADP^+$ (0.5 mM), DMSO (1% v/v), 100 mM KPi pH 7.0, 16 h ([b]24 h), 30 °C, 200 r.p.m.

2.2. Screening 384 metagenomic IREDs panel protocol

384-well plate of 50 μL reaction mixture contained D-glucose (50 mM), GDH (0.1 mg/mL, Codexis CDX-901), $NADP^+$ (0.5 mM), IRED CFE (4 mg/mL), purified 6-HDNO (0.5 mg/mL stored in 100 mM KPi pH 7.8) and THP (10 mM, stock conc. = 1 M in DMSO). Each well was made up to 50 μL with KPi buffer of reaction volume (100 mM, pH 7.0). The 384-well plate was spun down at 1,000 r.p.m for 1 min, sealed with foil based seal and incubated at 30 °C with shaking at 1,000 r.p.m. for 16 h. The plate was spun down at 1,000 r.p.m. for 1 min and each well quenched with 20 μL of 10 M NaOH in pH indicator dye and extracted once with 70 μL heptane (to avoid drying over anhydrous $MgSO_4$ before GC analysis). 384 samples were analysed by GC.

Table S2: Selected results from 384 metagenomic IREDs screening.

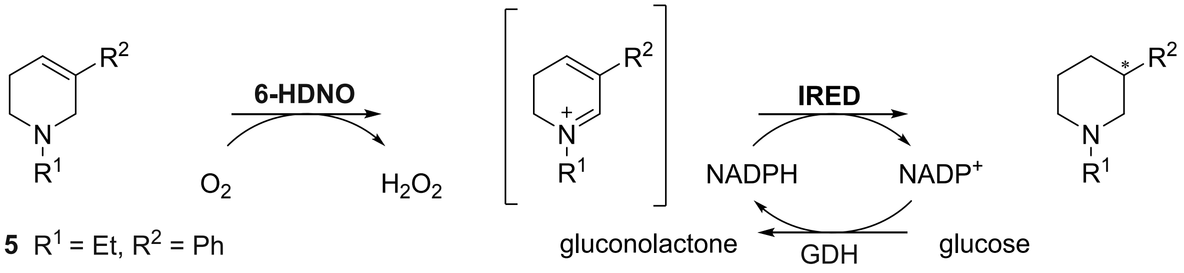

5 R^1 = Et, R^2 = Ph

1 R^1 = Pr, R^2 = Ph(3-OMe)

Entry	IREDs (series A)	**5b** conversion (%)	(*R*)-**5c** (ee%)	**1b** conversion (%)	(*R*)-**1c** (ee%)
1	pIR120/EneIRED-01*	>99	>99	>99	96
2	pIR259/EneIRED-02	>99	75	>99	>99
3	pIR088/EneIRED-03	>99	67	>99	98
4	pIR336/EneIRED-04*	>99	80	>99	96
	**IREDs (series B)**	**5b** conversion (%)	(*S*)-**5c** (ee%)	**1b** conversion (%)	(*S*)-**1c** (ee%)
7	pIR358/EneIRED-05	>99	59	>99	96
5	pIR117/EneIRED-06	>99	>99	>99	82
8	pIR361/EneIRED-07	>99	>99	>99	93
6	pIR260/EneIRED-08	>99	>99	>99	65
9	pIR374/EneIRED-09	>99	59	>99	84

Reaction conditions (analytical scale): purified 6-HDNO (0.5 mg/mL), IRED CFE (4 mg/mL), 10 mM substrate, 50 mM glucose, GDH-901 (0.1 mg/mL), 0.5 mM $NADP^+$, 16 h. *The EneIRED-01 and EneIRED-04 have previously been discovered[1].

2.3. Biocatalytic cascade control experiments

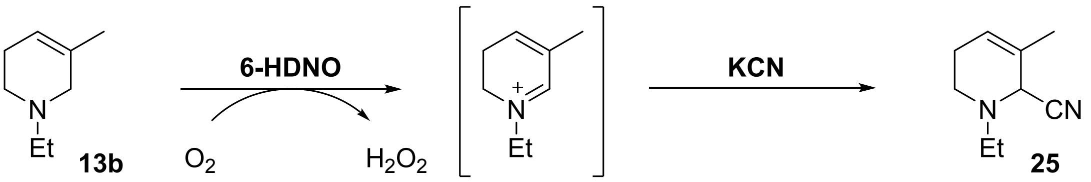

50 mM

70% yield

In a 50 mL falcon tube, KCN (326 mg, 5 mmol) and purified 6-HDNO (1 mg/mL) were dissolved in 100 mM KP_i buffer (20 mL, pH 7.0). 1-ethyl-5-methyl-1,2,3,6-THP (**13b**) (125 mg, 1 mmol, stock conc. = 1 M in DMSO) was added and the reaction shaken at 30 °C, 250 r.p.m. for 16 h. The reaction was basified to pH 12 with 10 M NaOH solution and the product extracted into MTBE (3 x 20 mL) with intermediate centrifugation (4 °C, 4000 r.p.m., 3 min) to improve the separation of phases. The organic layers were combined, dried over anhydrous $MgSO_4$ and concentrated *in vacuo* to give the

crude product. The crude was purified on a short pad of celite to afford **31** as a colourless oil (105 mg, 70%). **^{1}H NMR** (400 MHz, $CDCl_3$): δ 5.70 – 5.63 (m, 1H), 3.98 (s, 1H), 2.88 – 2.79 (m, 1H), 2.76 – 2.57 (m, 2H), 2.45 (td, J = 11.4, 4.2 Hz, 1H), 2.38 – 2.23 (m, 1H), 2.10 – 1.99 (m, 1H), 1.86 – 1.80 (m, 3H), 1.16 (t, J = 7.2 Hz, 3H); **^{13}C NMR** (100 MHz, $CDCl_3$): δ 127.7, 124.7, 116.1, 55.5, 49.5, 45.4, 25.7, 20.5, 12.5; **HRMS** calcd. for $C_9H_{15}N_2^+$ 151.1230 [M+H]$^+$, found 151.1225.

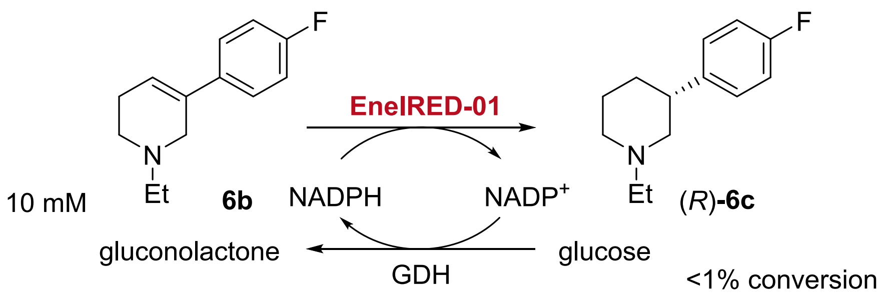

A 500 µL reaction mixture contained D-glucose (50 mM), GDH (0.1 mg/mL, Codexis CDX-901), NADP$^+$ (0.5 mM), EneIRED-01 CFE (4 mg/mL) and 1-ethyl-5-(4-fluorophenyl)-1,2,3,6-THP (**6b**) (10 mM, 5 µmol, stock conc. = 1 M in DMSO). The reaction volume was made up to 500 µL with KP_i buffer (100 mM, pH 7.0). Reactions were shaken at 30 °C, 200 r.p.m. and monitored with ^{19}F NMR over 16 h.

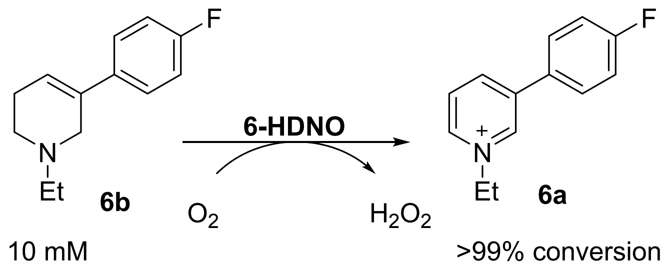

A 500 µL reaction mixture contained D-glucose (50 mM), GDH (0.1 mg/mL, Codexis CDX-901), NADP$^+$ (0.5 mM), purified 6-HDNO (0.5 mg/mL) and 1-ethyl-5-(4-fluorophenyl)-1,2,3,6-THP (**6b**) (10 mM, 5 µmol, stock conc. = 1 M in DMSO). The reaction volume was made up to 500 µL with KP_i buffer (100 mM, pH 7.0). Reactions were shaken at 30 °C, 200 r.p.m. and monitored with ^{19}F NMR over 24 h.

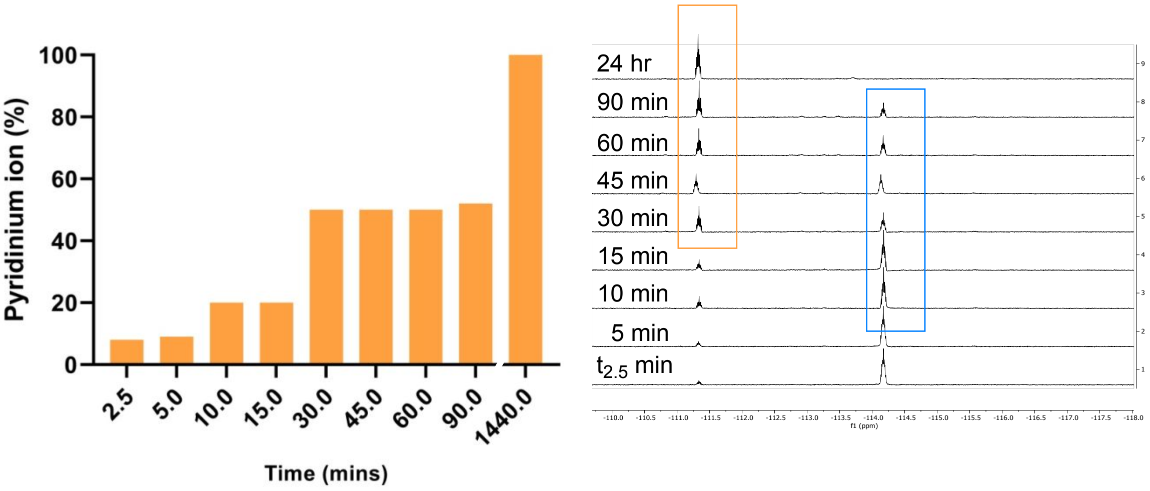

Figure S6: Time trial from *in situ* ^{19}F NMR spectra of the reaction with 6-HDNO only shows the disappearance of THP-**6b** and the formation of pyridinium ion **6a**.

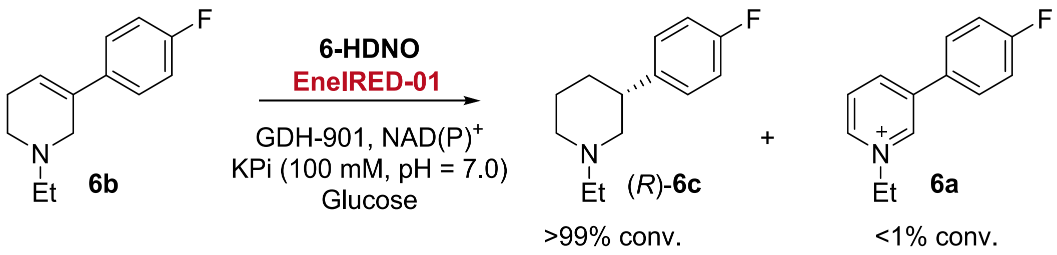

A 500 µL reaction mixture contained D-glucose (50 mM), GDH (0.1 mg/mL, Codexis CDX-901), $NADP^+$ (0.5 mM), EneIRED-01 CFE (4 mg/mL), purified 6-HDNO (0.5 mg/mL) and 1-ethyl-5-(4-fluorophenyl)-1,2,3,6-THP (**6b**) (10 mM, 5 µmol, stock conc. = 1 M in DMSO). The reaction volume was made up to 500 µL with KPi buffer (100 mM, pH 7.0). Reactions were shaken at 30 °C, 200 r.p.m. and monitored with ^{19}F NMR over 16 h.

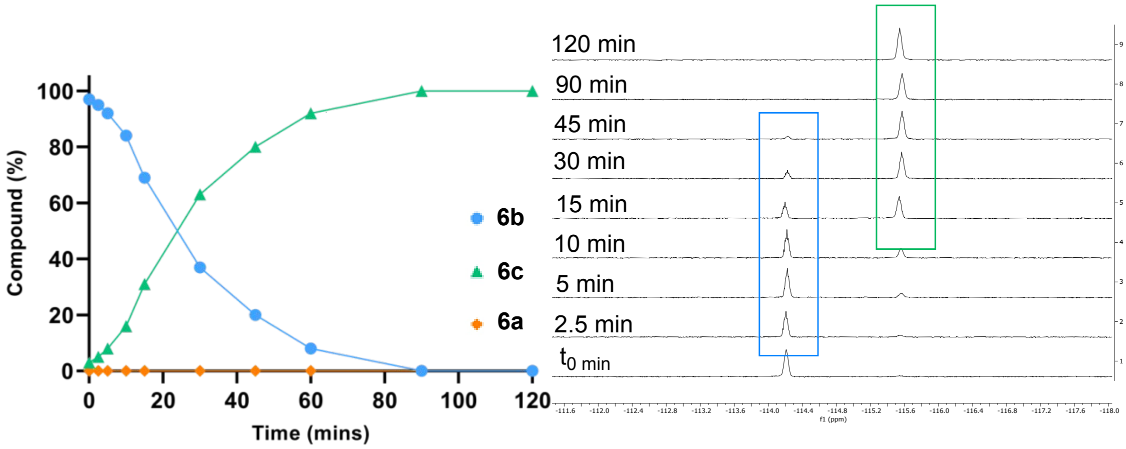

Figure S7: Kinetic profile from *in situ* ^{19}F NMR spectra of the cascade reaction with 6-HDNO and EneIRED-01 shows the disappearance of THP-**6b** and the formation of piperidine (*R*)-**6c**.

3. General procedure for biotransformations

A 500 µL reaction mixture contained D-glucose (50 mM), GDH (0.1 mg/mL, Codexis CDX-901), $NADP^+$ (0.5 mM), EneIRED CFE (4 mg/mL), purified 6-HDNO (0.5 mg/mL stored in 100 mM KP_i pH 7.8) and THP (10 mM, stock conc. = 1 M in DMSO). The reaction volume was made up to 500 µL with KPi buffer (100 mM, pH 7.0). Reactions were incubated at 30 °C with shaking at 200 r.p.m. for 0-16 h, after which they were quenched by the addition of 40 µL of 10 M NaOH and extracted once with 500 µL MTBE, dried over anhydrous $MgSO_4$ and analysed on GCMS followed by chiral HPLC or GC.

Table S3: Substrate scope of the 6-HDNO-EneIRED cascade (full table of Manuscript **Fig. 2**).

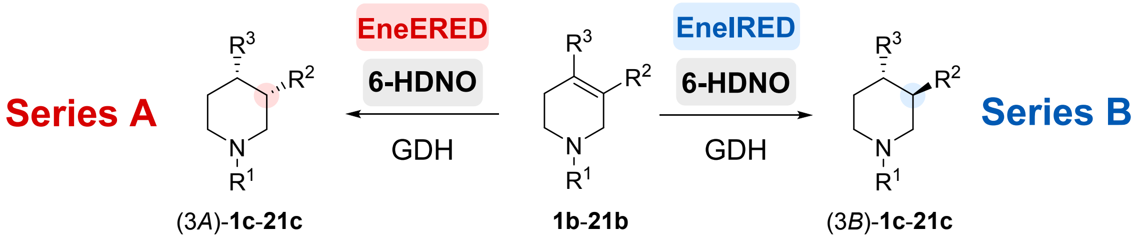

	Series A (EneIRED)				Series B (EneIRED)			
**Piperidine**	**Conversion (%)**	**Yield (%)**	**ee (%)**	**dr**	**Conversion (%)**	**Yield (%)**	**ee (%)**	**dr**
**1c**	>99	79	96	-	>99	87	96[b]	-
**2c**	>99	89	>99[a]	-	>99	60	92[c]	-
**3c**	>99	81	99	-	>99	76	99[d]	-
**4c**	>99	83	>99	-	>99	72	93[d]	-
**5c**	>99	86	86	-	>99	n.d.	89[d]	-
**6c**	>99	81	99	-	>99	n.d.	>99[d]	-
**7c**	>99	78	>99	-	>99	n.d.	99[d]	-
**8c**	88	62	>99	-	>99	n.d.	98[c]	-
**9c**	>99	85	86	-	>99	n.d.	>99[c]	-
**10c**	>99	73	94	-	>99	n.d.	98[d]	-
**11c**	>99	64	90	-	>99	n.d.	83[d]	-
**12c**	>99	77	>99	-	n.d.	-	-	-
**13c**	>99	42	n.d.	-	n.d.	-	-	-

**14c**	49	n.d.	n.d.	-	n.d.	-	-	-
**15c**	>99	34	-	-	>99	n.d.	-	-
**16c**	>99	62	-	-	>99	n.d.	-	-
**17c**	>99	54	89	*trans*<br>69:31	>99	n.d.	98[c]	*cis*<br>60:40
**18c**	>99	68	>99	*cis*<br>90:10	>99	n.d.	98[e]	*trans*<br>89:11
**19c**	>99	87	n.d.	*cis*<br>98:2	>99	n.d.	n.d.	*trans*<br>97:3[f]
**20c**	>99	89	n.d.	*cis*<br>96:4	96	n.d.	n.d.	*trans*<br>86:14[d]
**21c**	93	n.d	n.d.	97:3[a]	>99	n.d.	n.d.	92:8[c]

Reaction conditions (preparative scale): purified 6-HDNO (1 mg/mL), EneIRED CFE (10 mg/mL), THP (1 mmol), glucose (5 mmol), GDH CDX-901 (0.5 mg/mL), $NADP^+$ (0.5 mmol), DMSO (1% v/v), 100 mM KPi pH 7.0, 16-24 h, 30 °C, 200 r.p.m. (analytical conditions): purified 6-HDNO (0.5 mg/mL), EneIRED CFE (4 mg/mL), 10 mM THP (5 µmol), 50 mM glucose (25 µmol), GDH CDX-901 (0.1 mg/mL), 0.5 mM $NADP^+$ (0.05 µM), 2-16 h). All examples use EneIRED-01 except; [a]EneIRED-02, [b]EneIRED-05, [c]EneIRED-06, [d]EneIRED-07, [e]EneIRED-08 and [f]EneIRED-09. n.d. = not determined.

4. Absolute Configuration Determination

Enantiomeric excess (ee) was determined chiral HPLC, SFC and GC methods.

4.1. Commercial optically pure products: Analysis

Absolute configurations of **1c** (demethylated product) and **2c** were determined by comparing the HPLC traces of commercial optically pure (*R*)-(+)-3-(3-Hydroxyphenyl)-*N*-propylpiperidine and (*S*)-(-)-(3-methanesulfonyl-phenyl)-1-propyl-piperidine hydrochloride respectively.

Absolute configurations of **5c** and **11c** were determined by comparing the SFC traces of *N*-alkylated commercial optically pure (*S*)-3-phenylpiperidine with iodoethane and iodomethane respectively. Please see **section 13.2** for more details.

4.2. Literature comparison: Analysis

Absolute configuration of **3c** was determined by comparing the optical rotation data of previous studies[2]. The product of the biotransformation was deallylated and Boc protected to yield *tert*-butyl (*R*/*S*)-3-(4-bromophenyl)piperidine-1-carboxylate (23) product for direct comparison.

Absolute configuration of **4c** was determined by comparing the spectroscopic data of previous studies[3]. The product of the biotransformation were deallylated to yield (*R*/*S*)-4-(piperidin-3-yl)aniline (27) product for direct comparison.

4.3. VCD: Analysis

Absolute configurations of **6c**-**10c** were determined by the vibrational circular dichroism methodology (VCD, BioTools, Jupiter, FL). Please see **section 12** for more details.

5. Preparative-Scale Procedure and Product Characterisation

5.1. General Method 1: preparative-scale procedure

In a 50 mL falcon tube, glucose (900 mg, 5.00 mmol), $NADP^+$ (6 mg, 0.01 mmol), GDH (0.5 mg/mL, CDX-901) EneIRED CFE (10 mg/mL) and purified 6-HDNO (1 mg/mL) were dissolved in 100 mM KP_i buffer (20 mL, pH 7.0). THP (1.00 mmol, stock conc. = 1 M in DMSO) was added and the reaction shaken at 30 °C, 250 r.p.m. for 16 h. The reaction was basified to pH 12 with 10 M NaOH solution and the product extracted into MTBE (3 x 20 mL) with intermediate centrifugation (4 °C, 4000 r.p.m., 3 min) to improve the separation of phases. The organic layers were combined, dried over anhydrous $MgSO_4$ and concentrated *in vacuo* to give the crude product. The crude was purified on a short pad of celite to afford the corresponding compound.

(*R*)-3-(3-methoxyphenyl)-1-propylpiperidine (1c)

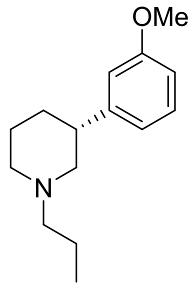

Following General Method 1 with 5-(3-methoxyphenyl)-1-propyl-1,2,3,6-THP (**1b**) (231 mg, 1.00 mmol) and shaking at 30 °C, 250 r.p.m. for 16 h to afford the title compound as a colourless oil (184 mg, 79%).

Enantioselectivity: EneIRED-01, 96% ee

^{1}H NMR (400 MHz, $CDCl_3$): δ 7.21 (t, *J* = 7.8 Hz, 1H), 6.83 (d, *J* = 7.8 Hz, 1H), 6.81 – 6.78 (m, 1H), 6.78 – 6.71 (m, 1H), 3.80 (s, 3H), 3.06 – 2.93 (m, 2H), 2.81 (tt, *J* = 11.7, 3.6 Hz, 1H), 2.36 – 2.25 (m, 2H), 2.00 – 1.86 (m, 3H), 1.84 – 1.63 (m, 2H), 1.58 – 1.37 (m, 3H), 0.89 (t, *J* = 7.4 Hz, 3H); **^{13}C NMR** (100 MHz, $CDCl_3$): δ 159.7, 146.7, 129.4, 119.8, 113.4, 111.4, 61.5, 61.4, 55.3, 54.1, 43.2, 31.8, 25.9, 20.2, 12.2; **HRMS** calcd. for $C_{15}H_{24}NO^+$ 243.1852 $[M+H]^+$, found 234.1841.

(*R*)-3-(1-propylpiperidin-3-yl)phenol (22)

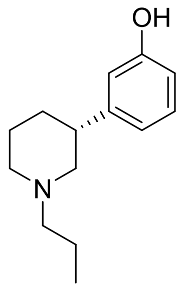

(R)-3-(3-methoxyphenyl)-1-propylpiperidine (**1c**) (150 mg, 0.64 mmol) was stirred in aqueous HBr (48% in H_2O, 4.4 mL, 39.19 mmol) at 100 °C for 1 h. The reaction mixture was cooled over ice and basified to pH 12 by addition of 5 M NaOH. The product was extracted into DCM (3 x 5 mL). The organic phase was dried over anhydrous $MgSO_4$ and concentrated *in vacuo* to afford the title compound as a colourless oil (126 mg, 89%).

^{1}H NMR (400 MHz, $CDCl_3$): δ 7.18 (t, J = 7.8 Hz, 1H), 6.82 – 6.67 (m, 3H), 3.25 – 3.17 (m, 1H), 3.06 (d, J = 11.5 Hz, 1H), 2.93 (tt, J = 11.8, 3.6 Hz, 1H), 2.47 – 2.25 (m, 2H), 2.05 – 1.93 (m, 3H), 1.88 – 1.68 (m, 2H), 1.62 – 1.44 (m, 3H), 0.88 (t, J = 7.3 Hz, 3H); **^{13}C NMR** (100 MHz, $CDCl_3$): δ 156.7, 145.8, 129.93, 117.9, 114.7, 114.2, 61.6, 61.3, 54.2, 42.1, 30.1, 25.5, 19.5, 12.2; **HRMS** calcd. for $C_{14}H_{22}NO^+$ 220.1696 [M+H]$^+$, found 220.1696.

(S)-3-(3-methoxyphenyl)-1-propylpiperidine (1c)

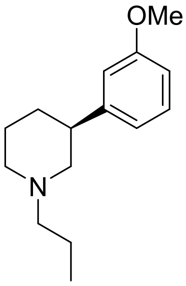

Following General Method 1 with 5-(3-methoxyphenyl)-1-propyl-1,2,3,6-THP (**1b**) (231 mg, 1.00 mmol) and shaking at 30 °C, 250 r.p.m. for 16 h to afford the title compound as a colourless oil (203 mg, 87%).

Enantioselectivity: EneIRED-05, 96% ee

^{1}H NMR (400 MHz, $CDCl_3$): δ 7.21 (t, J = 7.9 Hz, 1H), 6.87 – 6.71 (m, 3H), 3.80 (s, 3H), 3.06 – 2.93 (m, 2H), 2.81 (tt, J = 11.7, 3.6 Hz, 1H), 2.36 – 2.25 (m, 2H), 2.00 – 1.86 (m, 3H), 1.84 – 1.63 (m, 2H),

1.60 – 1.48 (m, 2H), 1.48 – 1.36 (m, 1H), 0.89 (t, *J* = 7.4 Hz, 3H); **^{13}C NMR** (100 MHz, $CDCl_3$): δ 159.7, 146.7, 129.4, 119.7, 113.4, 111.4, 61.5, 61.4, 55.3, 54.1, 43.2, 31.8, 25.9, 20.2, 12.2; **HRMS** calcd. for $C_{15}H_{24}NO^+$ 234.1852 $[M+H]^+$, found 234.1857.

(–)-preclamol (22)

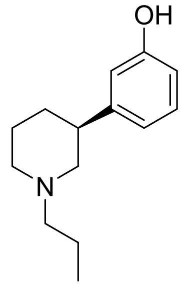

(*S*)-3-(3-methoxyphenyl)-1-propylpiperidine (**1c**) (190 mg, 0.81 mmol) was stirred in aqueous HBr (48% in H_2O, 5.6 mL, 49.60 mmol) at 100 °C for 1 h. The reaction mixture was cooled over ice and basified to pH 12 by addition of 5 M NaOH. The product was extracted into DCM (3 x 5 mL). The organic phase was dried over anhydrous $MgSO_4$ and concentrated *in vacuo* to afford the title compound as a colourless oil (164 mg, 92%).

^{1}H NMR (400 MHz, $CDCl_3$): δ 7.18 (t, *J* = 7.8 Hz, 1H), 6.81 – 6.67 (m, 3H), 3.27 – 3.19 (m, 1H), 3.09 (d, *J* = 11.0 Hz, 1H), 2.93 (tt, *J* = 11.8, 3.5 Hz, 1H), 2.48 – 2.27 (m, 2H), 2.07 – 1.92 (m, 3H), 1.86 – 1.71 (m, 2H), 1.64 – 1.43 (m, 3H), 0.87 (t, *J* = 7.4 Hz, 3H); **^{13}C NMR** (100 MHz, $CDCl_3$): δ 157.1, 145.5, 129.9, 117.5, 114.8, 114.4, 61.5, 61.3, 54.1, 41.9, 30.0, 25.3, 19.3, 12.2; **HRMS** calcd. for $C_{14}H_{22}NO^+$ 220.1696 $[M+H]^+$, found 220.1698.

(*R*)-3-(3-(methylsulfonyl)phenyl)-1-propylpiperidine (2c)

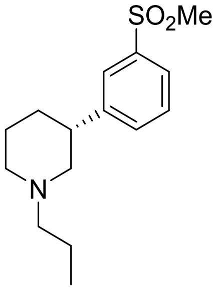

Following General Method 1 with 5-(3-(methylsulfonyl)phenyl)-1-propyl-1,2,3,6-THP (**2b**) (279 mg, 1.00 mmol) and shaking at 30 °C, 250 r.p.m. for 16 h to afford the title compound as a colourless oil (250 mg, 89%).

Enantioselectivity: EneIRED-02, >99% ee

^{1}H NMR (400 MHz, $CDCl_3$): δ 7.82 – 7.73 (m, 2H), 7.54 – 7.44 (m, 2H), 3.04 (s, 3H), 3.02 – 2.84 (m, 3H), 2.36 – 2.26 (m, 2H), 2.05 – 1.88 (m, 3H), 1.83 – 1.75 (m, 1H), 1.75 – 1.63 (m, 1H), 1.57 – 1.37 (m, 3H), 0.88 (t, *J* = 7.4 Hz, 3H); **^{13}C NMR** (100 MHz, $CDCl_3$): δ 146.9, 140.7, 133.0, 129.5, 125.9, 125.4, 61.2, 60.9, 53.8, 44.6, 42.9, 31.7, 25.6, 20.1, 12.1; **HRMS** calcd. for $C_{15}H_{24}NO_2S^+$ 282.1522 $[M+H]^+$, found 282.1530.

(–)-OSU6162 (2c)

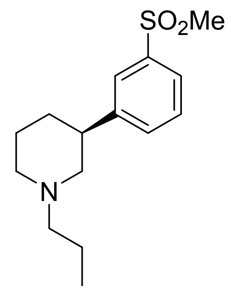

Following General Method 1 with 5-(3-(methylsulfonyl)phenyl)-1-propyl-1,2,3,6-THP (**2b**) (279 mg, 1.00 mmol) and shaking at 30 °C, 250 r.p.m. for 16 h to afford the title compound as a colourless oil (169 mg, 60%).

Enantioselectivity: EneIRED-06, 92% ee

^{1}H NMR (400 MHz, $CDCl_3$): δ 7.82 – 7.73 (m, 2H), 7.54 – 7.44 (m, 2H), 3.04 (s, 3H), 3.02 – 2.84 (m, 3H), 2.36 – 2.26 (m, 2H), 2.05 – 1.88 (m, 3H), 1.83 – 1.75 (m, 1H), 1.75 – 1.63 (m, 1H), 1.57 – 1.37 (m, 3H), 0.88 (t, *J* = 7.4 Hz, 3H); **^{13}C NMR** (100 MHz, $CDCl_3$): δ 146.9, 140.7, 133.0, 129.5, 125.9, 125.4, 61.2, 60.9, 53.8, 44.6, 42.9, 31.7, 25.6, 20.1, 12.1; **HRMS** calcd. for $C_{15}H_{24}NO_2S^+$ 282.1522 $[M+H]^+$, found 282.1530.

(*R*)-1-allyl-3-(4-bromophenyl)piperidine (3c)

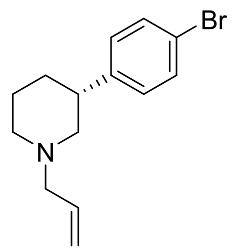

Following General Method 1 with 1-allyl-5-(4-bromophenyl)-1,2,3,6-THP (**3b**) (278 mg, 1.00 mmol) and shaking at 30 °C, 250 r.p.m. for 24 h to afford the title compound as a yellow oil (227 mg, 81%).

Enantioselectivity: EneIRED-01, 99% ee

^{1}H NMR (400 MHz, $CDCl_3$): δ 7.44 – 7.36 (m, 2H), 7.14 – 7.06 (m, 2H), 5.88 (ddt, *J* = 16.8, 10.1, 6.6 Hz, 1H), 5.20 – 5.10 (m, 2H), 3.04 – 2.93 (m, 4H), 2.79 (tt, *J* = 11.5, 3.6 Hz, 1H), 1.99 – 1.84 (m, 3H), 1.83 – 1.64 (m, 2H), 1.40 (qd, *J* = 12.4, 4.4 Hz, 1H); **^{13}C NMR** (100 MHz, $CDCl_3$): δ 143.8, 135.3, 131.5, 129.1, 120.1, 118.0, 62.3, 61.0, 53.8, 42.6, 31.6, 25.8; **HRMS** calcd. for $C_{14}H_{19}BrN^+$ 280.0695 $[M+H]^+$, found 280.0697.

(*R*)-3-(4-bromophenyl)piperidine (26)

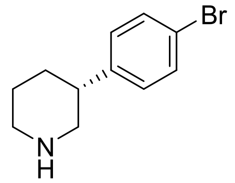

Under nitrogen, $RhCl(PPh_3)_3$ (33 mg, 0.04 mmol) was added to a solution of (*R*)-1-allyl-3-(4-bromophenyl)piperidine (**3c**) (200 mg, 0.71 mmol) in MeCN:H_2O (84:16, w/w, 18 mL). The reaction mixture was stirred at 100 ^{o}C for 16 h, cooled to room temperature and diluted with DCM. The organic layer was washed with $NaHCO_3$ (2 × 20 mL), dried over anhydrous $MgSO_4$ and concentrated *in vacuo* to yield crude product which was carried forward without further purification (160 mg, 94%). **^{1}H NMR** (400 MHz, $CDCl_3$): δ 7.45 – 7.37 (m, 2H), 7.12 – 7.05 (m, 2H), 3.12 (t, *J* = 11.7 Hz, 2H), 2.70 – 2.55 (m, 3H), 1.97 (d, *J* = 9.2 Hz, 1H), 1.85 – 1.81 (m, 2H), 1.66 – 1.51 (m, 2H); **^{13}C NMR** (100 MHz, $CDCl_3$): δ 144.0, 131.6, 129.0, 120.0, 54.0, 46.7, 43.9, 29.8, 27.1.

***tert*-butyl (*R*)-3-(4-bromophenyl)piperidine-1-carboxylate (23)**

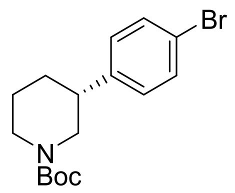

1 M NaOH (0.80 mL, 0.80 mmol) was added to a mixture of (*R*)-3-(4-bromophenyl)piperidine (**26**) (160 mg 0.67 mmol) in MTBE (5 mL) at room temperature. After 5 min Boc anhydride (146 mg, 0.67 mmol) was slowly added to the reaction mixture which was stirred at room temperature until **26** was consumed (monitored by GC-MS). The organic layer was washed with water (2 × 10 mL), dried over anhydrous $MgSO_4$ and concentrated *in vacuo* to yield the crude product. The crude product was subjected to flash column chromatography on silica (gradient elution from 20:1 to 10:1 hexane:MTBE) to afford the title compound as a colourless oil (214 mg, 94%). $[\alpha]^{25}_{D}$ = +30 (*c* = 0.04, $CHCl_3$); **^{1}H NMR** (500 MHz, $CDCl_3$, 273 K): δ 7.42 (dd, *J* = 14.1, 8.0 Hz, 2H), 7.10 (d, *J* = 8.2 Hz, 2H), 4.21 (dd, *J* = 24.4, 12.1 Hz, 1H), 4.07 (t, *J* = 13.2 Hz, 1H), 2.79 – 2.56 (m, 3H), 2.00 – 1.94 (m, 1H), 1.79 – 1.71 (m, 1H), 1.60 – 1.52 (m, 2H), 1.45 (d, *J* = 11.1 Hz, 9H); **^{13}C NMR** (100 MHz, $CDCl_3$): δ 154.9, 142.6, 131.7, 129.0, 120.4, 79.7, 42.1, 31.8, 28.6, 25.5; **HRMS** calcd. for $C_{16}H_{23}BrNO_2^+$ 340.0907 $[M+H]^+$, found 340.0916.

(*S*)-1-allyl-3-(4-bromophenyl)piperidine (3c)

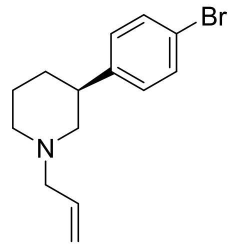

Following General Method 1 with 1-allyl-5-(4-bromophenyl)-1,2,3,6-THP (**3b**) (278 mg, 1.00 mmol) and shaking at 30 °C, 250 r.p.m. for 24 h to afford the title compound as a yellow oil (213 mg, 76%).

Enantioselectivity: EneIRED-07, 99% ee

^{1}H NMR (400 MHz, $CDCl_3$): δ 7.43 – 7.39 (m, 2H), 7.14 – 7.06 (m, 2H), 5.91 (ddt, *J* = 16.9, 10.1, 6.7 Hz, 1H), 5.25 – 5.14 (m, 2H), 3.16 – 2.76 (m, 5H), 2.04 – 1.89 (m, 3H), 1.82 – 1.77 (m, 2H), 1.50 –

1.35 (m, 1H); **^{13}C NMR** (100 MHz, $CDCl_3$): δ 143.8, 135.3, 131.5, 129.1, 120.1, 118.0, 62.3, 61.0, 53.8, 42.6, 31.6, 25.8; **HRMS** calcd. for $C_{14}H_{19}BrN^+$ 280.0695 $[M+H]^+$, found 280.0689.

(*S*)-3-(4-bromophenyl)piperidine (26)

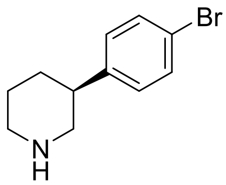

Under nitrogen, $RhCl(PPh_3)_3$ (33 mg, 0.04 mmol) was added to a solution of (*S*)-1-allyl-3-(4-bromophenyl)piperidine (**3c**) (213 mg, 0.76 mmol) in MeCN:H_2O (84:16, w/w, 18 mL). The reaction mixture was stirred at 100 ^oC for 16 h, cooled to room temperature and diluted with DCM. The organic layer was washed with $NaHCO_3$ (2 × 20 mL), dried over anhydrous $MgSO_4$ and concentrated *in vacuo* to yield crude product which was carried forward without further purification (179 mg, 98%). **^{1}H NMR** (400 MHz, $CDCl_3$): δ 7.44 – 7.38 (m, 2H), 7.12 – 7.05 (m, 2H), 3.15 (t, *J* = 11.8 Hz, 2H), 2.73 – 2.58 (m, 3H), 2.09 – 2.06 (m, 1H), 1.87 – 1.83 (m, 2H), 1.62 – 1.57 (m, 2H); **^{13}C NMR** (100 MHz, $CDCl_3$): δ 144.0, 131.6, 129.0, 120.0, 54.0, 46.7, 43.9, 29.8, 27.1.

***tert*-butyl (*S*)-3-(4-bromophenyl)piperidine-1-carboxylate (23)**

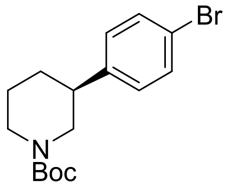

1 M NaOH (0.84 mL, 0.84 mmol) was added to a mixture of (*S*)-3-(4-bromophenyl)piperidine (**26**) (167 mg 0.70 mmol) in MTBE (5 mL) at room temperature. After 5 min Boc anhydride (153 mg, 0.70 mmol) was slowly added to the reaction mixture which was stirred at room temperature until **26** was consumed (monitored by GC-MS). The organic layer was washed with water (2 × 10 mL), dried over anhydrous $MgSO_4$ and concentrated *in vacuo* to yield the crude product. The crude product was subjected to flash column chromatography on silica (gradient elution from 20:1 to 10:1 hexane:MTBE) to afford the title compound as a colourless oil (155 mg, 65%). $[\alpha]^{25}_D$ -39.4 (*c* = 0.03, $CHCl_3$), lit.[2] $[\alpha]^{25}_D$ = −62.4 (*c* = 0.37, $CHCl_3$); **^{1}H NMR** (500 MHz, $CDCl_3$, 273 K): δ 7.42 (dd, *J* = 15.4, 8.0 Hz, 2H), 7.10 (dd, *J* = 8.4, 3.2 Hz, 2H), 4.21 (dd, *J* = 21.6, 12.1 Hz, 1H), 4.11 – 4.02 (m, 1H), 2.78 – 2.71 (m, 1H),

2.71 – 2.55 (m, 2H), 1.99 – 1.94 (m, 1H), 1.80 – 1.69 (m, 1H), 1.62 – 1.49 (m, 2H), 1.44 (d, *J* = 12.6 Hz, 9H); **^{13}C NMR** (100 MHz, $CDCl_3$): δ 154.9, 142.6, 131.7, 129.0, 120.4, 79.7, 42.1, 31.8, 29.8, 28.6, 25.5; **HRMS** calcd. for $C_{16}H_{23}BrNO_2^+$ 340.0907 $[M+H]^+$, found 340.0911.

(*R*)-4-(1-allylpiperidin-3-yl)aniline (4c)

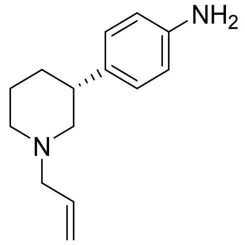

Following General Method 1 with 4-(1-allyl-1,2,5,6-tetrahydropyridin-3-yl)aniline (**4b**) (214 mg, 1.00 mmol) and shaking at 30 °C, 250 r.p.m. for 24 h to afford the title compound as an orange oil (180 mg, 83%).

Enantioselectivity: EneIRED-01, >99% ee

^{1}H NMR (400 MHz, $CDCl_3$): δ 7.05 – 6.97 (m, 2H), 6.66 – 6.58 (m, 2H), 5.89 (ddt, *J* = 16.8, 10.1, 6.6 Hz, 1H), 5.19 – 5.07 (m, 2H), 3.54 (s, 1H), 3.05 – 2.92 (m, 4H), 2.71 (tt, *J* = 11.8, 3.7 Hz, 1H), 1.97 – 1.83 (m, 3H), 1.81 – 1.62 (m, 2H), 1.38 (qd, *J* = 12.4, 4.3 Hz, 1H); **^{13}C NMR** (100 MHz, $CDCl_3$): δ 144.78, 135.41, 135.00, 128.04, 117.83, 115.24, 62.39, 61.65, 53.87, 42.16, 31.73, 25.93; **HRMS** calcd. for $C_{14}H_{21}N_2^+$ 217.1699 $[M+H]^+$, found 217.1691.

(*R*)-4-(piperidin-3-yl)aniline (27)

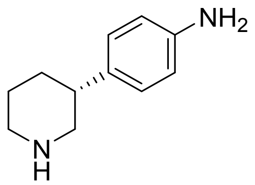

Under nitrogen, $RhCl(PPh_3)_3$ (33 mg, 0.04 mmol) was added to a solution of (*R*)-4-(1-allylpiperidin-3-yl)aniline (**4c**) (151 mg, 0.70 mmol) in MeCN:H_2O (84:16, w/w, 18 mL). The reaction mixture was stirred at 100 °C for 16 h, cooled to room temperature and diluted with DCM. The organic layer was washed with $NaHCO_3$ (2 × 20 mL), dried over anhydrous $MgSO_4$ and concentrated *in vacuo* to yield crude product which was carried forward without further purification (39 mg, 32%). **^{1}H NMR** (400 MHz,

$CDCl_3$): δ 7.00 – 6.92 (m, 2H), 6.66 – 6.58 (m, 2H), 3.34 – 3.23 (m, 2H), 2.88 – 2.65 (m, 3H), 2.05 – 1.95 (m, 1H), 1.94 – 1.87 (m, 1H), 1.87 – 1.74 (m, 1H), 1.66 – 1.50 (m, 1H); **^{13}C NMR** (100 MHz, $CDCl_3$): δ 143.4, 130.3, 125.9, 113.4, 49.4, 42.9, 38.6, 29.1, 22.4; **GCMS** calcd. for $C_{11}H_{16}N_2$ 176.1313 [M], found 176,1300.

***tert*-butyl (*R*)-3-(4-aminophenyl)piperidine-1-carboxylate (24)**

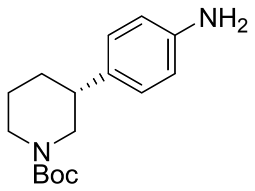

To a mixture of (*R*)-4-(piperidin-3-yl)aniline (**33**) (39 mg 0.22 mmol) in DCM (5 mL) at 0 °C, Boc anhydride (48 mg, 0.22 mmol) was added and stirred at 0 °C until **33** was consumed (monitored by GC-MS). Then a solution of 7 N NH_3 in MeOH (1 mL) was added and the mixture was stirred for 30 min at 0 °C. After dilution with DCM, the organic layer was washed with $NaHCO_3$ (2 × 10 mL), dried over anhydrous $MgSO_4$ and concentrated *in vacuo* to yield the crude product. The crude product was subjected to flash column chromatography on silica (gradient elution from 20:1 to 10:1 hexane:MTBE) to afford the title compound as a white solid (52 mg, 86%). **^{1}H NMR** (500 MHz, $CDCl_3$, 273 K): δ 7.05 – 6.99 (m, 2H), 6.69 – 6.60 (m, 2H), 4.26 – 4.16 (m, 1H), 4.10 – 4.04 (m, 1H), 3.64 (s, 2H), 2.75 – 2.61 (m, 2H), 2.60 – 2.52 (m, 1H), 1.99 – 1.91 (m, 1H), 1.73 (s, 1H), 1.60 – 1.38 (m, 12H); **^{13}C NMR** (100 MHz, $CDCl_3$): 155.0, 145.0, 133.8, 128.0, 115.3, 79.4, 49.6, 41.8, 32.0, 28.6, 28.4, 27.1; **HRMS** calcd. for $C_{16}H_{25}N_2O_2^+$ 277.1911 [M+H]$^+$, found 277.1920.

(*S*)-4-(1-allylpiperidin-3-yl)aniline (4c)

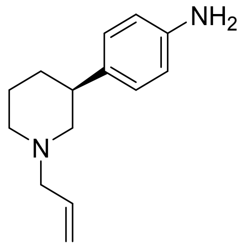

Following General Method 1 with 4-(1-allyl-1,2,5,6-tetrahydropyridin-3-yl)aniline (**4b**) (214 mg, 1.00 mmol) and shaking at 30 °C, 250 r.p.m. for 24 h to afford the title compound as an orange oil (156 mg, 72%).

Enantioselectivity: EneIRED-07, 93% ee

^{1}H NMR (400 MHz, $CDCl_3$): δ 7.05 – 6.97 (m, 2H), 6.66 – 6.58 (m, 2H), 5.89 (ddt, *J* = 16.8, 10.1, 6.6 Hz, 1H), 5.19 – 5.07 (m, 2H), 3.54 (s, 1H), 3.05 – 2.92 (m, 4H), 2.71 (tt, *J* = 11.8, 3.7 Hz, 1H), 1.97 – 1.83 (m, 3H), 1.81 – 1.62 (m, 2H), 1.38 (qd, *J* = 12.4, 4.3 Hz, 1H); **^{13}C NMR** (100 MHz, $CDCl_3$): δ 144.78, 135.41, 135.00, 128.04, 117.83, 115.24, 62.39, 61.65, 53.87, 42.16, 31.73, 25.93; **HRMS** calcd. for $C_{14}H_{21}N_2^+$ 217.1699 $[M+H]^+$, found 217.1691.

(*S*)-4-(piperidin-3-yl)aniline (27)

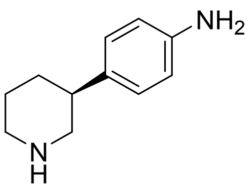

Under nitrogen, $RhCl(PPh_3)_3$ (32 mg, 0.03 mmol) was added to a solution of (*S*)-4-(1-allylpiperidin-3-yl)aniline (**4c**) (150 mg, 0.69 mmol) in MeCN:H_2O (84:16, w/w, 18 mL). The reaction mixture was stirred at 100 °C for 16 h, cooled to room temperature and diluted with DCM. The organic layer was washed with $NaHCO_3$ (2 × 20 mL), dried over anhydrous $MgSO_4$ and concentrated *in vacuo* to yield crude product which was carried forward without further purification (79 mg, 65%). **^{1}H NMR** (400 MHz, $CDCl_3$): δ 6.93 – 6.85 (m, 2H), 6.59 – 6.51 (m, 2H), 3.27 – 3.16 (m, 2H), 2.89 – 2.58 (m, 3H), 1.98 – 1.88 (m, 1H), 1.87 – 1.80 (m, 1H), 1.80 – 1.65 (m, 1H), 1.59 – 1.42 (m, 1H); **^{13}C NMR** (100 MHz, $CDCl_3$): δ 145.4, 132.3, 127.9, 115.4, 51.4, 44.8, 40.6, 31.1, 24.4; **GCMS** calcd. for $C_{11}H_{16}N_2$ 176.1313 [M], found 176,1200.

***tert*-butyl (*S*)-3-(4-aminophenyl)piperidine-1-carboxylate (24)**

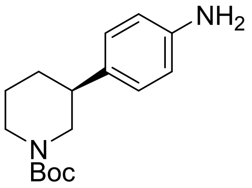

To a mixture of (*S*)-4-(piperidin-3-yl)aniline (**27**) (79 mg 0.45 mmol) in DCM (5 mL) at 0 °C, Boc anhydride (98 mg, 0.45 mmol) was added and stirred at 0 °C until **27** was consumed (monitored by GC-MS). Then a solution of 7 N NH_3 in MeOH (1 mL) was added and the mixture was stirred for 30 min at 0 °C. After dilution with DCM, the organic layer was washed with $NaHCO_3$ (2 × 10 mL), dried

over anhydrous $MgSO_4$ and concentrated *in vacuo* to yield the crude product. The crude product was subjected to flash column chromatography on silica (gradient elution from 20:1 to 10:1 hexane:MTBE) to afford the title compound as a white solid (110 mg, 89%). **^{1}H NMR** (500 MHz, $CDCl_3$, 273 K): δ 6.99 – 6.93 (m, 2H), 6.62 – 6.54 (m, 2H), 4.20 – 4.09 (m, 1H), 4.03 – 3.97 (m, 1H), 3.57 (s, 2H), 2.69 – 2.57 (m, 1H), 2.53 – 2.46 (m, 1H), 1.92 – 1.84 (m, 1H), 1.69 – 1.63 (m, 1H), 1.54 – 1.32 (m, 12H); **^{13}C NMR** (100 MHz, $CDCl_3$): δ 154.9, 145.0, 133.8, 127.9, 115.3, 79.4, 32.0, 28.6, 28.4; **HRMS** calcd. for $C_{16}H_{25}N_2O_2^+$ 277.1911 [M+H]$^+$, found 277.1898.

(*R*)-1-ethyl-3-phenylpiperidine (5c)

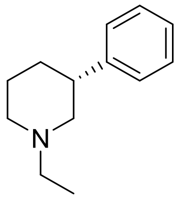

Following General Method 1 with 1-ethyl-5-phenyl-1,2,3,6-THP (**5b**) (187 mg, 1.00 mmol) and shaking at 30 °C, 250 r.p.m. for 16 h to afford the title compound as a pale-yellow oil (163 mg, 86%).

Enantioselectivity: EneIRED-01, 86% ee

^{1}H NMR (400 MHz, $CDCl_3$): δ 7.31 – 7.11 (m, 5H), 3.04 – 2.91 (m, 2H), 2.78 (tt, *J* = 11.8, 3.6 Hz, 1H), 2.38 (q, *J* = 7.2 Hz, 2H), 1.93 – 1.86 (m, 2H), 1.86 – 1.59 (m, 3H), 1.41 (qd, *J* = 12.4, 4.4 Hz, 1H), 1.04 (t, *J* = 7.2 Hz, 3H); **^{13}C NMR** (100 MHz, $CDCl_3$): δ 145.0, 128.5, 127.4, 126.5, 61.2, 53.6, 52.9, 43.2, 31.8, 26.0, 12.20; **HRMS** calcd. for $C_{13}H_{20}N^+$ 190.1590 [M+H]$^+$, found 190.1581.

(*R*)-1-ethyl-3-(4-fluorophenyl)piperidine (6c)

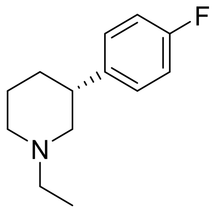

Following General Method 1 with 1-ethyl-5-(4-fluorophenyl)-1,2,3,6-THP (**6b**) (205 mg, 1.00 mmol) and shaking at 30 °C, 250 r.p.m. for 16 h to afford the title compound as a pale-yellow oil (167 mg, 81%).

Enantioselectivity: EneIRED-01, 99% ee

^{1}H NMR (400 MHz, $CDCl_3$): δ 7.19 – 7.11 (m, 2H), 6.98 – 6.90 (m, 2H), 2.97 (d, *J* = 11.2 Hz, 2H), 2.85 – 2.73 (m, 1H), 2.40 (q, *J* = 7.2 Hz, 2H), 1.92 – 1.82 (m, 3H), 1.81 – 1.62 (m, 2H), 1.38 (qd, *J* = 12.4, 4.4 Hz, 1H), 1.07 (t, *J* = 7.2 Hz, 3H); **^{13}C NMR** (100 MHz, $CDCl_3$): δ 162.7, 160.2, 140.6, 128.6, 128.5, 115.2, 115.0, 61.2, 53.4, 52.8, 42.3, 31.9, 25.8, 12.1; **HRMS** calcd. for $C_{13}H_{19}FN^+$ 208.1496 [M+H]$^+$, found 208.1488.

(*R*)-1-ethyl-3-(4-methoxyphenyl)piperidine (7c)

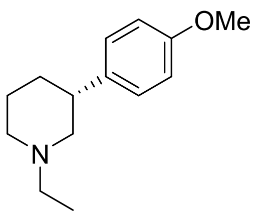

Following General Method 1 with 1-ethyl-5-(4-methoxyphenyl)-1,2,3,6-THP (**7b**) (217 mg, 1.00 mmol) and shaking at 30 °C, 250 r.p.m. for 16 h to afford the title compound as a yellow oil (170 mg, 78%).

Enantioselectivity: EneIRED-01, >99% ee

^{1}H NMR (400 MHz, $CDCl_3$): δ 7.20 – 7.12 (m, 2H), 6.89 – 6.80 (m, 2H), 3.79 (s, 3H), 3.06 – 2.95 (m, 2H), 2.78 (tt, *J* = 11.7, 3.6 Hz, 1H), 2.43 (q, *J* = 7.2 Hz, 2H), 1.94 – 1.85 (m, 3H), 1.85 – 1.77 (m, 1H), 1.77 – 1.63 (m, 1H), 1.42 (qd, *J* = 12.4, 4.4 Hz, 1H), 1.09 (t, *J* = 7.2 Hz, 3H); **^{13}C NMR** (100 MHz, $CDCl_3$): δ 158.2, 137.2, 128.2, 113.9, 61.5, 55.4, 53.6, 52.9, 42.3, 32.0, 26.0, 12.2; **HRMS** calcd. for $C_{14}H_{22}NO^+$ 220.1696 [M+H]$^+$, found 220.1685.

(*S*)-1-ethyl-3-(furan-2-yl)piperidine (8c)

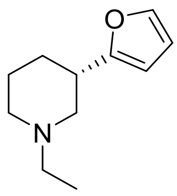

Following General Method 1 with 1-ethyl-5-(furan-2-yl)-1,2,3,6-THP (**8b**) (177 mg, 1.00 mmol) and shaking at 30 °C, 250 r.p.m. for 16 h to afford the title compound as a brown oil (111 mg, 62%).

Enantioselectivity: EneIRED-01, >99% ee

^{1}H NMR (400 MHz, $CDCl_3$): δ 7.33 – 7.29 (m, 1H), 6.31 – 6.27 (m, 1H), 6.06 (d, *J* = 3.2 Hz, 1H), 3.46 – 3.39 (m, 1H), 3.38 – 3.22 (m, 2H), 2.82 – 2.73 (m, 2H), 2.35 – 2.24 (m, 2H), 2.14 – 2.03 (m, 2H), 1.91 – 1.83 (m, 1H), 1.52 (qd, *J* = 13.5, 13.0, 3.9 Hz, 1H), 1.30 (t, *J* = 7.3 Hz, 3H); **^{13}C NMR** (126 MHz, $CDCl_3$): δ 155.9, 141.5, 110.2, 104.9, 56.6, 52.9, 52.7, 34.8, 28.5, 23.7, 10.6; **HRMS** calcd. for $C_{11}H_{18}NO^+$ 180.1383 $[M+H]^+$, found 180.13758.

(*S*)-1-ethyl-3-(thiophen-2-yl)piperidine (9c)

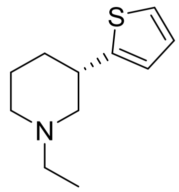

Following General Method 1 with 1-ethyl-5-(thiophen-2-yl)-1,2,3,6-THP (**9b**) (193 mg, 1.00 mmol) and shaking at 30 °C, 250 r.p.m. for 16 h to afford the title compound as a brown oil (166 mg, 85%).

Enantioselectivity: EneIRED-01, 86% ee

^{1}H NMR (400 MHz, $CDCl_3$): δ 7.13 (d, *J* = 5.1 Hz, 1H), 6.96 – 6.90 (m, 1H), 6.84 (d, *J* = 3.4 Hz, 1H), 3.29 – 3.16 (m, 2H), 3.08 – 3.00 (m, 1H), 2.53 (q, *J* = 7.2 Hz, 2H), 2.14 – 1.97 (m, 3H), 1.86 – 1.77 (m, 2H), 1.51 – 1.40 (m, 1H), 1.15 (t, *J* = 7.2 Hz, 3H); **^{13}C NMR** (100 MHz, $CDCl_3$): δ 148.0, 126.7, 123.1, 123.0, 61.0, 53.2, 52.7, 37.8, 33.0, 25.2, 11.7; **HRMS** calcd. for $C_{11}H_{18}NS^+$ 196.1154 $[M+H]^+$, found 196.1148.

(*R*)-1-ethyl-3-(naphthalen-2-yl)piperidine (10c)

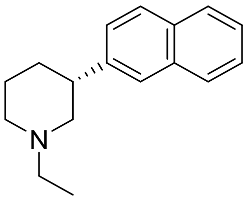

Following General Method 1 with 1-ethyl-5-(naphthalen-2-yl)-1,2,3,6-THP (**10b**) (237 mg, 1.00 mmol) and shaking at 30 °C, 250 r.p.m. for 16 h to afford the title compound as a yellow oil (173 mg, 73%).

Enantioselectivity: EneIRED-01, 94% ee

^{1}H NMR (400 MHz, $CDCl_3$): δ 7.83 – 7.76 (m, 3H), 7.67 (s, 1H), 7.49 – 7.36 (m, 3H), 3.18 – 3.10 (m, 1H), 3.10 – 2.95 (m, 2H), 2.48 (q, *J* = 7.2 Hz, 2H), 2.11 – 1.92 (m, 3H), 1.89 – 1.75 (m, 2H), 1.58 (qd, *J* = 12.3, 4.7 Hz, 1H), 1.12 (t, *J* = 7.2 Hz, 3H); **^{13}C NMR** (100 MHz, $CDCl_3$): δ 142.4, 133.7, 132.5, 128.0, 127.8, 127.7, 126.4, 126.1, 125.5, 125.4, 61.0, 53.7, 52.9, 43.2, 31.8, 26.0, 12.2; **HRMS** calcd. for $C_{17}H_{22}N^+$ 240.1747 $[M+H]^+$, found 240.1745.

1-ethyl-5-(naphthalen-2-yl)-1,2,3,4-THP (10e)

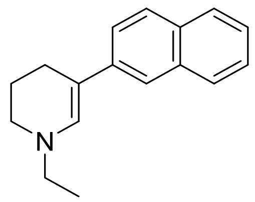

In a 50 mL falcon tube, D-glucose (900 mg, 5.00 mmol), $NADP^+$ (6 mg, 0.01 mmol), CFE lysate GDH (0.5 mg/mL, CDX-901) CFE lysate EneIRED-01 (10 mg/mL) and purified 6-HDNO (1 mg/mL) were dissolved in 100 mM KP_i buffer (20 mL, pH 7.0). 1-ethyl-5-(naphthalen-2-yl)-1,2,3,6-THP (**10b**) (237 mg, 1.00 mmol, stock conc. = 1 M in DMSO) was added and the reaction shaken at 30 °C, 250 r.p.m. and monitored by GCMS. The reaction was basified to pH 12 with 10 M NaOH solution and the product extracted into MTBE (3 x 20 mL) with intermediate centrifugation (4 °C, 4000 r.p.m., 3 min) to improve the separation of phases. The organic layers were combined, dried over anhydrous $MgSO_4$ and concentrated *in vacuo* to give the crude product. The crude product was subjected to flash column chromatography on silica (gradient elution from 100% to 95:5 DCM:methanol) to afford the title compound as a brown oil (149 mg, 63%). **^{1}H NMR** (400 MHz, $CDCl_3$): δ 7.72 (dd, *J* = 17.9, 8.6 Hz, 3H), 7.56 (dd, *J* = 8.6, 2.0 Hz, 1H), 7.52 (d, *J* = 2.0 Hz, 1H), 7.41 (ddd, *J* = 8.2, 6.9, 1.3 Hz, 1H), 7.32 (ddd, *J* = 8.2, 6.9, 1.3 Hz, 1H), 6.78 (s, 1H), 3.16 – 3.03 (m, 4H), 2.55 (q, *J* = 7.3, 6.4 Hz, 2H), 2.08 (p, *J* = 6.2 Hz, 2H), 1.18 (t, *J* = 7.1 Hz, 3H); **^{13}C NMR** (100 MHz, $CDCl_3$): δ 139.2, 134.4, 134.31, 131.0, 127.5, 127.5, 127.4, 125.9, 123.9, 122.0, 118.9, 105.7, 50.2, 45.9, 23.3, 22.7, 13.5; **GCMS** calcd. for $C_{17}H_{19}N$ 237.1517 [M], found 237.1600

(*R*)-1-ethyl-3-(naphthalen-2-yl)piperidine (10c)

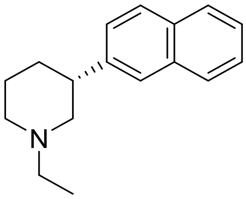

In a 50 mL falcon tube, D-glucose (900 mg, 5.00 mmol), NADP+ (6 mg, 0.01 mmol), CFE lysate GDH (0.5 mg/mL, CDX-901) and CFE lysate EneIRED-01 (10 mg/mL) were dissolved in 100 mM KP_i buffer (20 mL, pH 7.0). 1-ethyl-5-(naphthalen-2-yl)-1,2,3,4-THP (**10e**) (237 mg, 1.00 mmol, stock conc. = 1 M in DMSO) was added and the reaction shaken at 30 °C, 250 r.p.m. for 16 h. The reaction was basified to pH 12 with 10 M NaOH solution and the product extracted into MTBE (3 x 20 mL) with intermediate centrifugation (4 °C, 4000 r.p.m., 3 min) to improve the separation of phases. The organic layers were combined, dried over anhydrous $MgSO_4$ and concentrated *in vacuo* to give the crude product. The crude was purified on a short pad of celite to afford the title compound as a pale brown oil (210 mg, 88%).

Enantioselectivity: EneIRED-01, 94% ee

(*R*)-1-methyl-3-phenylpiperidine (11c)

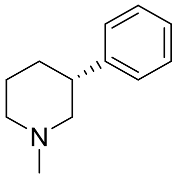

Following General Method 1 with 1-methyl-5-phenyl-1,2,3,6-THP (**11b**) (173 mg, 1.00 mmol) and shaking at 30 °C, 250 r.p.m. for 16 h to afford the title compound as a pale-yellow oil (112 mg, 64%).

Enantioselectivity: EneIRED-01, 90% ee

^{1}H NMR (400 MHz, $CDCl_3$): δ 7.34 – 7.16 (m, 5H), 3.00 – 2.88 (m, 2H), 2.88 – 2.77 (m, 1H), 2.30 (s, 3H), 2.10 – 1.86 (m, 3H), 1.85 – 1.66 (m, 2H), 1.43 (qd, *J* = 12.4, 4.5 Hz, 1H); **^{13}C NMR** (100 MHz, $CDCl_3$): δ 144.8, 128.5, 127.3, 126.5, 63.4, 56.0, 46.7, 43.2, 31.1, 26.0; **HRMS** calcd. for $C_{12}H_{18}N^+$ 176.1434 $[M+H]^+$, found 176.1429.

(*A*)-3-phenyl-1-(prop-2-yn-1-yl)piperidine (12c)

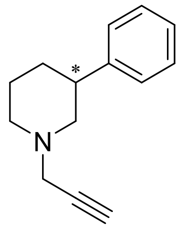

Following General Method 1 with 5-phenyl-1-(prop-2-yn-1-yl)-1,2,3,6-THP (**12b**) (197 mg, 1.00 mmol) and shaking at 30 °C, 250 r.p.m. for 16 h to afford the title compound as a pale-yellow oil (153 mg, 77%).

Enantioselectivity: EneIRED-01, >99% ee

^{1}H NMR (400 MHz, $CDCl_3$): δ 7.41 – 7.25 (m, 5H), 3.44 – 3.39 (m, 2H), 3.10 – 2.97 (m, 2H), 2.97 – 2.86 (m, 1H), 2.39 – 2.25 (m, 3H), 2.05 – 1.96 (m, 1H), 1.95 – 1.86 (m, 1H), 1.86 – 1.74 (m, 1H), 1.53 (qd, *J* = 12.5, 4.3 Hz, 1H); **^{13}C NMR** (100 MHz, $CDCl_3$): δ 144.5, 128.5, 127.3, 126.5, 79.1, 73.2, 59.9, 52.6, 47.4, 43.1, 31.2, 25.8; **HRMS** calcd. for $C_{14}H_{18}N^{+}$ 200.1434 $[M+H]^{+}$, found 200.1443.

(*A*)-1-ethyl-3-methylpiperidine (13c)

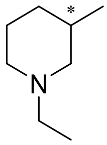

Following General Method 1 with 1-ethyl-5-methyl-1,2,3,6-THP (**13b**) (125 mg, 1.00 mmol) and shaking at 30 °C, 250 r.p.m. for 16 h to afford the title compound which was isolated as a HCl salt using HCl (2M, MeOH) (53 mg, 42%).

Enantioselectivity: EneIRED-01, n.d.

^{1}H NMR (400 MHz, $CDCl_3$): δ 11.26 (s, 1H), 3.39 – 3.30 (m, 1H), 3.27 – 3.17 (m, 1H), 3.00 – 2.87 (m, 2H), 2.51 (tdd, *J* = 12.7, 9.7, 2.8 Hz, 1H), 2.26 – 2.13 (m, 2H), 2.13 – 1.99 (m, 1H), 1.77 – 1.67 (m, 2H), 1.30 (t, *J* = 7.3 Hz, 3H), 1.03 – 0.90 (m, 1H), 0.80 (d, *J* = 5.7 Hz, 3H); **^{13}C NMR** (100 MHz, $CDCl_3$): δ 58.0, 52.3, 51.8, 30.5, 28.6, 22.4, 18.7, 9.0; **HRMS** calcd. for $C_8H_{18}ClN^{+}$ 128.1434 $[M-Cl]^{+}$, found 128.1429.

1-ethyl-4-methylpiperidine (15c)

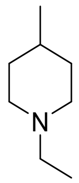

Following General Method 1 with 1-ethyl-4-methyl-1,2,3,6-THP (**15b**) (125 mg, 1.00 mmol) and shaking at 30 °C, 250 r.p.m. for 16 h to afford the title compound which was isolated as a HCl salt using HCl (2M, MeOH) (55 mg, 34%). **^{1}H NMR** (400 MHz, $CDCl_3$): δ 10.87 (s, 1H), 3.43 (d, *J* = 11.5 Hz, 2H), 3.09 – 2.96 (m, 2H), 2.79 – 2.65 (m, 2H), 1.87 – 1.75 (m, 3H), 1.67 – 1.56 (m, 1H), 1.36 (t, *J* = 7.0 Hz, 3H), 0.99 (dd, *J* = 11.9, 6.5 Hz, 1H), 0.91 (d, *J* = 6.3 Hz, 3H); **^{13}C NMR** (100 MHz, $CDCl_3$): δ 52.3, 52.3, 30.8, 29.1, 20.9, 9.3; **HRMS** calcd. for $C_8H_{18}ClN^+$ 128.1434 [M-Cl]$^+$, found 128.1430.

1-ethyl-4-phenylpiperidine (16c)

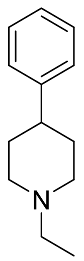

Following General Method 1 with 1-ethyl-4-phenyl-1,2,3,6-THP (**16b**) (187 mg, 1.00 mmol) and shaking at 30 °C, 250 r.p.m. for 24 h to afford the title compound as a yellow oil (117 mg, 62%). **^{1}H NMR** (400 MHz, $CDCl_3$): δ 7.26 – 7.07 (m, 5H), 3.01 (dt, *J* = 11.4, 3.4 Hz, 2H), 2.49 – 2.32 (m, 3H), 1.94 (td, *J* = 11.1, 4.1 Hz, 2H), 1.82 – 1.66 (m, 4H), 1.05 (t, *J* = 7.2 Hz, 3H); **^{13}C NMR** (100 MHz, $CDCl_3$): δ 146.6, 128.5, 127.0, 126.2, 54.1, 52.8, 43.0, 33.6, 12.2; **HRMS** calcd. for $C_{13}H_{19}N^+$ 190.1590 [M+H]$^+$, found 190.1585.

1-ethyl-3,4-dimethylpiperidine (17c)

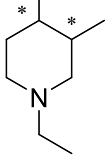

Following General Method 1 with 1-ethyl-4,5-dimethyl-1,2,3,6-THP (**17b**) (139 mg, 1.00 mmol) and shaking at 30 °C, 250 r.p.m. for 16 h to afford the title compound as a colourless oil (76 mg, 54%).

Diastereoselectivity: EneIRED-01, *trans* 69:31 dr, 89% ee

^{1}H NMR (400 MHz, $CDCl_3$): δ 3.55 – 2.95 (m, 5H), 1.44 (t, *J* = 7.3 Hz, 3H), 1.36 – 1.06 (m, 4H), 1.04 – 0.88 (m, 7H); **^{13}C NMR** (100 MHz, $CDCl_3$): δ 52.4, 36.2, 34.8, 31.2, 31.0, 29.8, 18.5, 16.5, 9.3; **HRMS** calcd. for $C_9H_{20}N^+$ 142.1590 [M+H]$^+$, found 142.1586.

2-ethyldecahydroisoquinoline (18c)

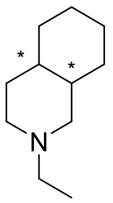

Following General Method 1 with 2-ethyl-1,2,3,4,5,6,7,8-octahydroisoquinoline (**18b**) (165 mg, 1.00 mmol) and shaking at 30 °C, 250 r.p.m. for 24 h to afford the title compound as a colourless oil (114 mg, 68%).

Diastereoselectivity: EneIRED-01, *cis* 90:10 dr, >99% ee

^{1}H NMR (400 MHz, $CDCl_3$): δ 3.02 – 2.63 (m, 4H), 2.13 – 0.97 (m, 17H); **^{13}C NMR** (100 MHz, $CDCl_3$): δ 58.1, 53.1, 52.7, 40.8, 39.2, 32.0, 30.2, 29.8, 26.1, 25.4, 10.1; **HRMS** calcd. for $C_{11}H_{22}N^+$ 168.1747 [M+H]$^+$, found 168.1751.

1-ethyl-4-methyl-3-phenylpiperidine (19c)

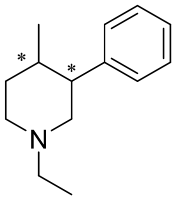

Following General Method 1 with 1-ethyl-4-methyl-5-phenyl-1,2,3,6-THP (**19b**) (201 mg, 1.00 mmol) and shaking at 30 °C, 250 r.p.m. for 16 h to afford the title compound as a yellow oil (177 mg, 87%).

Diastereoselectivity: EneIRED-01, *cis* 98:2 dr

^{1}**H NMR** (400 MHz, $CDCl_3$): δ 7.32 – 7.25 (m, 4H), 7.24 – 7.15 (m, 1H), 3.11 – 3.02 (m, 1H), 2.78 – 2.70 (m, 1H), 2.66 – 2.53 (m, 2H), 2.47 (q, *J* = 7.2 Hz, 2H), 2.39 – 2.35 (m, 1H), 2.13 – 2.02 (m, 1H), 1.97 – 1.84 (m, 1H), 1.65 – 1.54 (m, 1H), 1.11 (t, *J* = 7.2 Hz, 3H), 0.71 (d, *J* = 7.2 Hz, 3H); ^{13}**C NMR** (100 MHz, $CDCl_3$): δ 142.6, 128.6, 128.2, 126.3, 58.3, 52.9, 44.2, 32.3, 31.4, 29.8, 18.5, 11.6; **HRMS** calcd. for $C_{14}H_{22}N^+$ 204.1747 $[M+H]^+$, found 204.1750.

1-allyl-4-methyl-3-phenylpiperidine (20c)

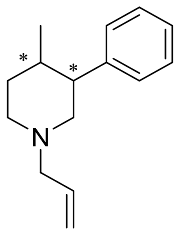

Following General Method 1 with 1-allyl-4-methyl-5-phenyl-1,2,3,6-THP (**20b**) (213 mg, 1.00 mmol) and shaking at 30 °C, 250 r.p.m. for 16 h to afford the title compound as a yellow oil (191 mg, 89%).

Diastereoselectivity: EneIRED-01, *cis* 96:4 dr

^{1}**H NMR** (400 MHz, $CDCl_3$): δ 7.36 – 7.24 (m, 4H), 7.24 – 7.16 (m, 1H), 5.91 (ddt, *J* = 16.8, 10.1, 6.5 Hz, 1H), 5.26 – 5.15 (m, 1H), 5.15 – 5.08 (m, 1H), 3.10 – 2.97 (m, 3H), 2.77 – 2.61 (m, 2H), 2.59 – 2.51 (m, 1H), 2.47 – 2.42 (m, 1H), 2.10 – 2.03 (m, 1H), 1.90 – 1.82 (m, 1H), 1.67 – 1.54 (m, 1H), 0.71 (d, *J* = 7.1 Hz, 3H); ^{13}**C NMR** (100 MHz, $CDCl_3$): δ 143.6, 135.8, 128.5, 128.0, 126.0, 117.5, 62.5, 54.2, 51.0, 45.3, 32.9, 32.0, 19.8; **HRMS** calcd. for $C_{15}H_{22}N^+$ 216.1747 $[M+H]^+$, found 216.1738.

5.2. Deuterium Labelling Experiments

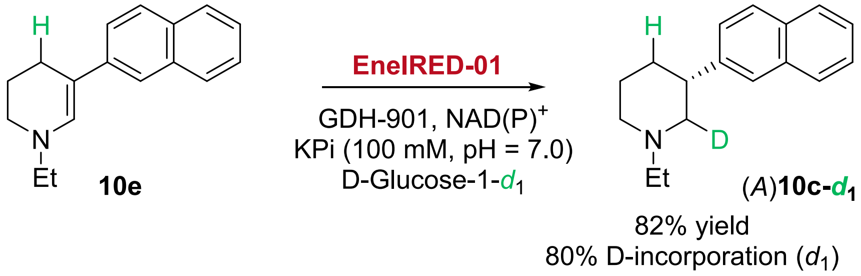

(3*A*)-1-ethyl-3-(naphthalen-2-yl)piperidine-2-*d* (10c)

In a 50 mL falcon tube, D-glucose-1-d_1 (900 mg, 5.00 mmol), $NADP^+$ (6 mg, 0.01 mmol), GDH (0.5 mg/mL, CDX-901) and EneIRED-01 CFE (10 mg/mL) were dissolved in 100 mM KP_i buffer (20 mL, pH 7.0). 1-ethyl-5-(naphthalen-2-yl)-1,2,3,4-THP (**10e**) (237 mg, 1 mmol, stock conc. = 1 M in DMSO)

was added and the reaction shaken at 30 °C, 250 r.p.m. for 16 h. The reaction was basified to pH 12 with 10 M NaOH solution and the product extracted into MTBE (3 x 20 mL) with intermediate centrifugation (4 °C, 4000 r.p.m., 3 min) to improve the separation of phases. The organic layers were combined, dried over anhydrous $MgSO_4$ and concentrated *in vacuo* to give the crude product. The crude was purified on a short pad of celite to afford the title compound as pale yellow oil (197 mg, 82%). **^{1}H NMR** (400 MHz, $CDCl_3$): δ 7.84 – 7.75 (m, 3H), 7.69 – 7.64 (m, 1H), 7.50 – 7.36 (m, 3H), 3.20 – 2.97 (m, 2H), 2.49 (qd, *J* = 7.2, 1.5 Hz, 2H), 2.12 – 2.05 (m, 1H), 2.05 – 1.93 (m, 2H), 1.92 – 1.74 (m, 2H), 1.59 (qd, *J* = 12.2, 5.1 Hz, 1H), 1.13 (t, *J* = 7.2 Hz, 3H); **^{13}C NMR** (100 MHz, $CDCl_3$): δ 142.3, 133.7, 132.5, 128.0, 127.8, 127.7, 126.4, 126.1, 125.5, 125.4, 60.9, 53.6, 52.9, 43.0, 31.7, 25.9, 12.1; **GCMS** calcd. for $C_{17}H_{20}DN$ 240.1737 [M], found 240.1400.

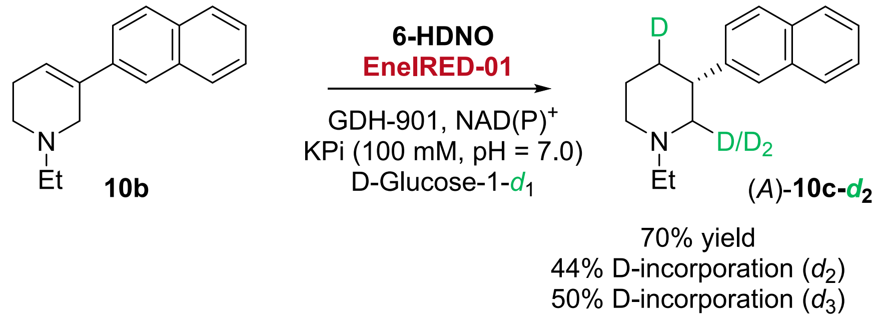

(3*A*)-1-ethyl-3-(naphthalen-2-yl)piperidine-2,4-*d*$_2$ (10c)

In a 50 mL falcon tube, >98% D-glucose-1-*d*$_1$ (900 mg, 5.00 mmol), NADP$^+$ (6 mg, 0.01 mmol), GDH (0.5 mg/mL, CDX-901) EneIRED-01 CFE (10 mg/mL) and purified 6-HDNO (1 mg/mL) were dissolved in 100 mM KP_i buffer (20 mL, pH 7.0). 1-ethyl-5-(naphthalen-2-yl)-1,2,3,6-THP (**10b**) (237 mg, 1.00 mmol, stock conc. = 1 M in DMSO) was added and the reaction shaken at 30 °C, 250 r.p.m. for 16 h. The reaction was basified to pH 12 with 10 M NaOH solution and the product extracted into MTBE (3 x 20 mL) with intermediate centrifugation (4 °C, 4000 r.p.m., 3 min) to improve the separation of phases. The organic layers were combined, dried over anhydrous $MgSO_4$ and concentrated *in vacuo* to give the crude product. The crude was purified on a short pad of celite to afford the title compound as yellow oil (169 mg, 70%). **^{1}H NMR** (400 MHz, $CDCl_3$): δ 7.84 – 7.74 (m, 3H), 7.66 (s, 1H), 7.50 – 7.35 (m, 3H), 3.20 – 2.96 (m, 2H), 2.52 – 2.44 (m, 2H), 2.11 – 1.92 (m, 3H), 1.90 – 1.72 (m, 2H), 1.12 (t, *J* = 7.2 Hz, 3H); **^{13}C NMR** (100 MHz, $CDCl_3$): δ 142.4, 133.7, 132.5, 128.0, 127.8, 127.7, 126.4, 126.1, 125.4, 125.3, 53.6, 52.8, 42.9, 42.8, 29.8, 25.8, 25.7, 12.1; **GCMS** calcd. for $C_{17}H_{19}D_2N$ 241.1800 [M], found 241.1900.

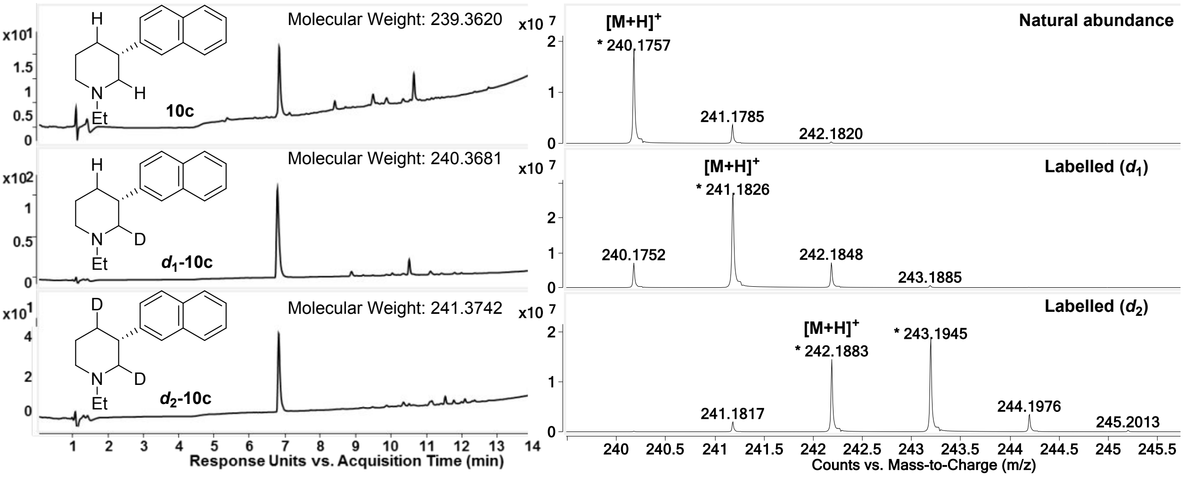

Figure S8: Comparison between LC and positive ion ESI-MS traces relevant to the deuterium labelling experiments.

Table S4: Experimental high resolution mass of natural abundance **10c**.

Natural abundance	$[M+H]^+$ (m/z)	Abundance
	240.1757	18285096.72
	241.1785	3658588.57
	242.182	284262.12

Table S5: Experimental high resolution mass of the isotopically enriched ***d*₁-10c** compared to the natural abundance **10c**.

Mono-deuterated	$[M+H]^+$ (m/z)	Abundance	Contribution of Nat. Abd.
Nat. Abd.	240.1752	7023949.66	-
Nat. Abd. + *d*1	241.1826	26914571.93	1405392.727
Nat. abd + *d*1	242.1848	7199602.81	109195.0921
*d*1	243.1885	594515.6	-

$$\%Nat.\,Abd. = \left[\frac{sum\ of\ Nat.\,Abd.}{sum\ of\ all\ Abundances}\right] \times 100 = 20.5\%$$

$$\%Dincorp\ (d1) = \left[\frac{sum\ of\ Abundance\ of\ d1}{sum\ of\ all\ Abundances}\right] \times 100 = 79.5\%$$

Table S6: Experimental high resolution mass of the isotopically enriched $\boldsymbol{d_2}$**-10c** compared to the contributions worked out with predicted % values from isotopic distribution calculator.[a]

Double-deuterated	[M+H]$^+$ (m/z)	Abundance	Contribution of *d*1[a]	Contribution of *d2*[a]	Contribution of *d*3[a]
*d*1	241.1817	2001409.02	-	-	-
*d*2 + *d*1	242.1883	14479708.87	380067.5729	14099641.3	-
*d*2 + *d*1 +*d*3	243.1945	18708050.83	34023.95334	2676111.918	15997914.96
*d*2 +*d*3	244.1976	3534357	-	239693.9021	3294663.098
*d*3	245.2013	299768.97	-	-	271964.5543

$$\%Dincorp\ (d1) = \left[\frac{sum\ of\ Abundance\ of\ d1}{sum\ of\ all\ Abundances}\right] \times 100 = 6.2\%$$

$$\%Dincorp\ (d2) = \left[\frac{sum\ of\ Abundance\ of\ d2}{sum\ of\ all\ Abundances}\right] \times 100 = 43.6\%$$

$$\%Dincorp\ (d3) = \left[\frac{sum\ of\ Abundance\ of\ d3}{sum\ of\ all\ Abundances}\right] \times 100 = 50.1\%$$

Discussion:

^{1}H, GCMS and LC-HMRS data all support the conclusion that the nicotinamide cofactor is the hydride-source. Examination of the ^{1}H-NMR trace of the reaction **10e** run with D-glucose-1-*d*1 reveals nearly complete suppression of one C-2 proton signal. The ^{1}H-NMR trace of the product isolated from the reaction of **10b** run with D-glucose-1-*d*1 shows high suppression of one C-2 and one C-4 proton signals (see **section 14**). Additional evidence for deuterium incorporation in compounds $\boldsymbol{d_1}$**-10c** and $\boldsymbol{d_2}$**-10c** can be found in the GCMS traces (see **section 8**, Fig. S28). The extent of deuterium incorporation can be calculated using comparison between high resolution positive ion ESI-MS (Fig. S8). The LC-HRMS gives a calculated 79.5% D-incorporation for product $\boldsymbol{d_1}$**-10c** and a mixture of d_2 and d_3 for product $\boldsymbol{d_2}$**-10c** (44.3% and 50.1% D-incorporation respectively). The D-incorporation calculations for product $\boldsymbol{d_2}$**-10c** shows some evidence of a non-productive pathway where EneIRED-01-catalysed C=N bond reduction of endocyclic α,β-unsaturated iminium (DHP) contends with the EneIRED-01-catalysed C=C bond conjugate reduction. In the presence of 6-HDNO, the THP produced is re-activated and ultimately a mixture of deuterated $\boldsymbol{d_2}$**-10c** and $\boldsymbol{d_3}$**-10c** is formed.

6. Cloning, expression, and purification protocols of biocatalysts

6.1. Amine oxidases

Preparation of MAO-N D5, D9, D11 and 6-HDNO E350L/E352D mutants used in this study are described in former publications[4–6]. Relevant genes were cloned into the vector pET16b using NdeI/BamHI (restriction enzymes from New England Biolabs) restriction sites encoding an *N*-terminal poly-histidine tag for subsequent downstream affinity purification of the enzyme. Following successful cloning as confirmed through Sanger Sequencing (Eurofins, Luxembourg), the pET16b-amine oxidase construct was transformed into chemically competent *E. coli* BL21 (DE3) (New England Biolabs) for recombinant protein expression.

For flask scale expression, overnight cultures were prepared by inoculating ~6 mL of lysogeny broth (LB) supplemented with 100 µg/mL ampicillin from a single colony, and subsequently grown for 16 h at 37 °C at 250 r.p.m. The overnight culture was used to inoculate a 2 L baffled Erlenmeyer flask containing 600 mL auto induction media (LB broth base including trace elements), supplemented with 100 µg/mL ampicillin. The culture was incubated for 48 h, 25 °C, 200 r.p.m. Cells were harvested by centrifugation and cell pellets were collected.

Buffer A: 100 mM KPi pH 7.8 + 300 mM NaCl + 30 mM imidazole. Buffer B: 100 mM KPi pH 7.8 + 300 mM NaCl + 1 M imidazole.

For affinity purification, cell pellets were re-suspended in Buffer A (~1 g cell / 3 mL) and lysed first by adding lysozyme (Sigma) (1 mg/mL) and incubating at 30 °C for 20 min. Further lysis was performed by sonication under the following conditions: (20s on, 20s off, x12 cycles) using a Soniprep 150 (MSE UK Ltd.) whilst the suspended cell solution was kept on an ice bath. Lysed cells were clarified using a JA-25.50 Aluminium Fixed-Angle Rotor in a Beckman Coulter centrifuge at 18000 r.p.m. for 45 min. The CFE lysate was loaded onto a HisTrap 5 mL FastFlow column (GE Healthcare) and the column washed with 10 mL Buffer A before the protein was eluted with a gradient of 0 – 100% Buffer B over 30 min using an AKTA (Cytiva). Fractions corresponding to the peak at 280 nM were checked for purity by SDS-PAGE (Tris/glycine 4–20% Tris-Hepes gel, NuSep) (Fig. S9 and S10). These fractions were pooled concentrated and buffer exchanged into 0.1 M potassium phosphate buffer, pH 7.8, by spin column (Vivaspin 6, 30 kDa cutoff, GE Healthcare). Purified protein was stored at 4 °C until use.

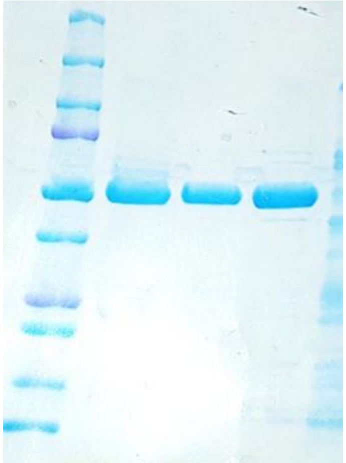

Figure S9: Weight Marker (PageRuler™ PreStained Protein Ladder): 10 kDa, 15 kDa, 25 kDa, 35 kDa, 40 kDa, 55 kDa, 70 kDa, 100 kDa, 130 kDa, 180 kDa,. **SDS-PAGE 1** Lane 1: Molecular Weight Marker. Lane 2: Pure fractions MAO-N D5. Lane 3: Pure fractions MAO-N D9. Lane 4: Pure fractions MAO-N D11.

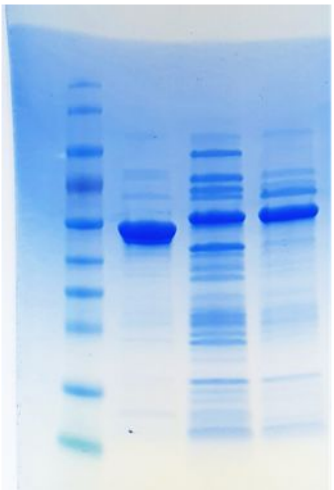

Figure S10: Weight Marker (PageRuler™ PreStained Protein Ladder): 10 kDa, 15 kDa, 25 kDa, 35 kDa, 40 kDa, 55 kDa, 70 kDa, 100 kDa, 130 kDa, 180 kDa,. **SDS-PAGE 1** Lane 1: Molecular Weight Marker. Lane 2: Pure fractions 6-HDNO. Lane 3: CFE lysate MAO-N D9. Lane 4: CFE lysate MAO-N D11.

6.2. IREDs

All the IREDs described in this study were obtained and expressed using the protocols described previously in: https://doi.org/10.1038/s41557-020-00606-w

All IREDs were expressed through recombinant expression using *E. coli* as the host. Flask scale expression of the selected metagenomic enzymes was performed in a 2 L Erlenmeyer baffled flask. Initially, a single colony following transformation of the pET28a-IRED construct into chemically competent *E. coli* BL21(DE3) was used to inoculate 20 mL of lysogeny broth (LB) media supplemented with 35 μg/mL of kanamycin. This culture was incubated at 37 °C for 16 h, 200 r.p.m.

This pre-culture was then used to inoculate 400 mL of Terrific Broth (TB) (Formedium, Hunstanton, Norfolk, UK) in a 2 L Erlenmeyer baffled flask supplemented with 35 µg/mL of kanamycin. This was then incubated at 37 °C, 200 r.p.m. for 1.5 h. The culture was then induced with 0.1 mM of isopropyl β-d-1-thiogalactopyranoside (IPTG) and then incubated at 23 °C, 200 r.p.m. for a further 24 h. Cell pellets were then collected by centrifugation.

E. coli cell pellets were then processed to obtain the biocatalyst, or the desired IRED in crude CFE lysate format through resuspension of wet-cell pellet, lysis, centrifugation and lyophilisation. *E. coli* cells were resuspended to approx. 5x wet-cell pellet weight in 0.1 M sodium phosphate pH 7.0. Lysis was undertaken using a MSE Soniprep 150 sonicator with a precooled 9.5 mm probe at amplitude 16 microns with 1 minute bursts per 50 mL of re-suspended cells with 5x repetitions. Lysed cells were clarified using a JA-25.50 Aluminium Fixed-Angle Rotor in a Beckman Coulter centrifuge for 45 min at 18,000 r.p.m. Following clarification the supernatant of the E. coli CFE lysate expressing the selected IRED was decanted. This CFE lysate was then lyophilised in a Lyovapor™ L-200 (Buchi, Suffolk, UK). Protein expression was then monitored *via* 4-20% SDS-PAGE.

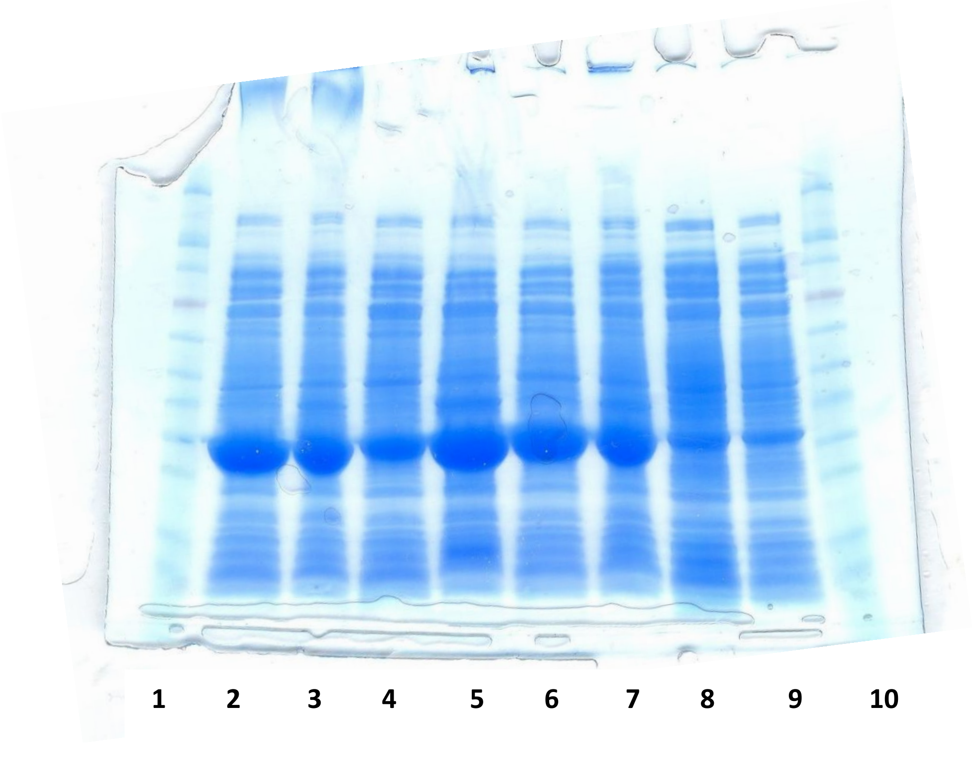

Figure S11: Molecular Weight Marker (PageRuler™ PreStained Protein Ladder): 10 kDa, 15 kDa, 25 kDa, 35 kDa, 40 kDa, 55 kDa, 70 kDa, 100 kDa, 130 kDa, 180 kDa,. Lane 1: Molecular Weight Marker. Lane 2: EneIRED-06 WC. Lane 3: EneIRED-06 CFE. Lane 4: EneIRED-01 WC. Lane 5:

EneIRED-01 CFE. Lane 6: EneIRED-08 WC. Lane 7: EneIRED-08 CFE. Lane 8: EneIRED-05 WC. Lane 9: EneIRED-05 CFE. Lane 10: Molecular Weight Marker.

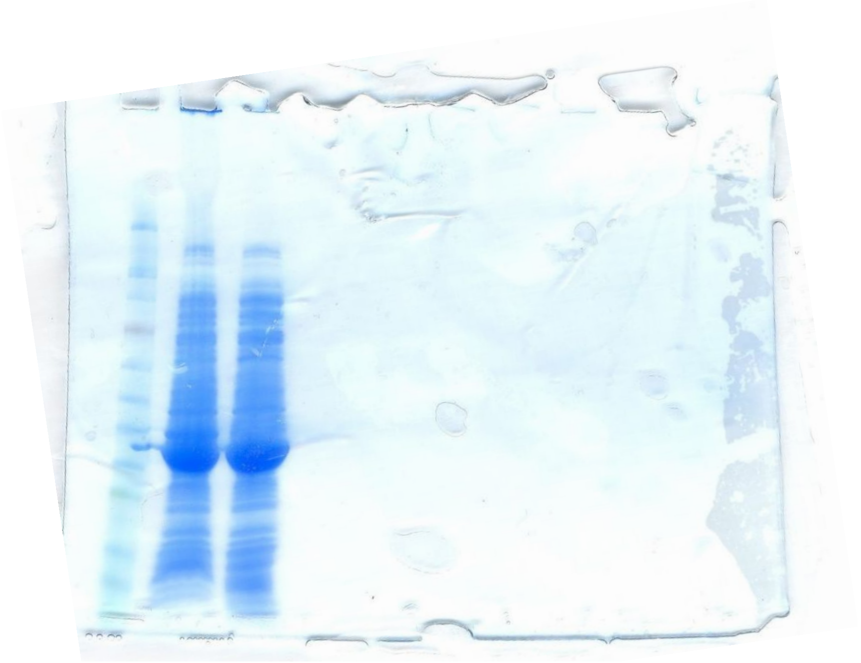

1 2 3

Figure S12: Molecular Weight Marker (PageRuler™ PreStained Protein Ladder): 10 kDa, 15 kDa, 25 kDa, 35 kDa, 40 kDa, 55 kDa, 70 kDa, 100 kDa, 130 kDa, 180 kDa,. Lane 1: Molecular Weight Marker. Lane 2: EneIRED-07 WC. Lane 3: EneIRED-07 CFE.

5.2.1. EneIRED Amino Acid Sequence Information

```
>EneIRED-01
MSMSGSNKPSVSVLGLGAMGSVLARTLLQAGYGVTVWNRSPERATALVQEGASLAREASE
AINASNLIIICMIDKAVFQDVLSSLDPLLNMSGKTIVNMSTGTVDDVERIAKRVDQHNGL
YVDAGIMCYPKDIGGQHTTILYSGNSDAYHAHESTLKVLAGNPKFLGADPTACTPTYLAL
YAFYFGAFAAWLEGAVLASCAGVSVQDFKALSPIMSDMLVDGIKTAADRIAASDYSGEQA
SVDVHVAGQEVVLDALQRANAPHASTDAYLSYCRMAQTAGMGELDIASLFKAMHP*
>EneIRED-02
MNVTTPEPTTVAVIGLGNLGQVLARTLLDQGHKVTVWNRSQDKADDLVARGATRAATPAD
AIRASDLVIICVLDYTTVRDLLTPAADALAGRVVVNVTSGIPEPARELATRVNGSGAAYV
DGAVYAIPQTIGTPEAFVLYSGDEEAFARHRALLETLGTAEFVGADAGLAAVHDVALLSG
MYGMFAGFFQTVAVSGSAGIEAVRVTELLVRWLKEAIAALPAFAAEIDAGDYRTQTSNLD
INAVGLANILAATRAQGVGVELLAPLHALFEQQVSAGHGAESLSRTIESLR
>EneIRED-03
MNMTTSRNVTAFGVGNMGAALAHALLKADTKVTIWNRTVDRPQVQSVLKAGATLEADVKA
AISGSSDILLFCLIDYDAMYKTLEPIKGTSDGLAGKTIVNVTNGTPQQALEMRDWIKARG
AARYFDGAVLVTPQMVATPQSLLVYSGESQETFDNIKTILQPLGTPLYYGPQVDAAAAQD
LAMLATMYGMFYGAFVGFGILKRSGQGQDVKVAPGTKQITIPVMAALTEYLGLLADVIDS
EDWASNGGNPLLMQVAGVANIIQAAKDANVNASGLEVLAEAMGKAVEDGWADGNVAAAAK
FI
>EneIRED-04
MSTSGSNKPCVSVLGLGAMGSVLARTLLQAGYGVTVWNRSPARATALLQEGASLAREASE
AINASNLIIVCMIDKAASQDVLSSLDPLLDMSGKTIVNMSTGTVDDVQRIARQVSQHNGL
YVDAGIMCYPKDIGGQHTTILYSGNSDAYHAHESTLKVLAGNPKFLGADPTACTPTYLAL
YAFYFGAFAAWLEGAVLASCAGVSVQDFKALSPIMSDMLVDGINTAADRIAASDFSGEQA
SVDVHVAGQEVVLDALQRAKAPHASTHAYLSYCRMAQTAGMGELDIASLFKAMHP*
>EneIRED-05
MSSKQKITVIGLGAMGATIARLYLEQGHEVTIWNRSADKAAPLVAQGAVLADSAAAAVRA
```

SRVVLMCVYDYRAADAILGAEGVAAAMDGRLLVQLTTGSPRDARDAQAWAQRHGATFLEG
AIQAAPEQMGKGDTPILMSGDEQVFRAVEPLLAVLGGGIVYLGEKISNAAAMDLATLSTI
YGTMLGFLHGARVAESEGFDVAEFGRIVAGIMPTFASFLQHEGAVIQSGDFKISQSPMRI
SVEATQRILQTARESGINSEFPAFAAGLFQRADAAGLGGEELAALIKLLRAPA
>EneIRED-06
MQPAISVLGTGRMGSALAYALLKAGHPTTVWNRTPAKAAPLAAAGAEVAASVRNAVAVSE
VVIVNVSDYQATQSLLRDKEVAGALEGRLIIELTSGTPDGGREAHGWAQRQGARYLDGAI
LATPDFIGTEAGTLLVSGPSGVFEEGRNVLGALGGNVQFIGEDPGLANALDSAVLALMWG
ALFGALQSIAVCRAEAIDLGVLARQWTATAPVVEGLVSDLIKRSAAGRYDADAETLSSVS
PHYSAFHHLVDLMEARGIDRTITGGYEAIFRRAIEAGHLHDDFASLSQFMGQPA*
>EneIRED-07
MSDPNADRPPVTVVGLGLMGQALAAAFLKGGHPTTVWNRSPEKAERLVADGAVLADTLES
AVTASPLVIVCVSDYDAVHELIRPVESALAGRVLVNLTTATSTQARETAEWAAQRNIPYL
DGAIMAIPPVIGTDGAVLLYSGHKSAFEAHESTLKAIAPAATTYLEEDHGLSSLYDMALL
GIMWGILNGFLHGAALLGTAKVKAETFAPLANTMISAITEYVTAYAPQVDEGRYEATDAT
MTVHQAAMEHLAEESEHLGIHSELPRFFKTLADRAVADGHAENSYAAMIELFRKPTA
>EneIRED-08
MPHASQSVTIIGLGPMGQAMAAAYLDRGYDVTLWNRTPSRADALVARGATLAPTAEQALS
AAELVILSLTDFDAMYAILEPAKDAVAGRTLVNLSSDTPEKARAGARWVAELGGTHLTGG
VLCPPPLIGTPDTSTFYSGPREAYDAHRATLEVITGKSDYRGDDQGLAALMYQLNMFIFW
PAMVAYWQAVAVAGAHGIQAREIAPYVTENFAGMGHFIDFYASRVDAGNHAGDVDRASMG
LASMEHVVHTASDAGVDTAFPAAVHDVFRRVVEAGHGADSFSWAVELLKKR
>EneIRED-09
MSSVSIFGLGAMGTAMAARFLEKNFKVTVWNRTPEKANKLLDKGASVSNTVLDGINASDL
IIVCLLDNAAVQTTFDQALEHVRGKTVINLTNGTPKQARKLSELFVSHGSQYVHGGIMAT
PSMIGSPHALILYSGSKDAYNTTESTLSLLAKGWFLGDDAGVASLHDLALLSGMYGLFSG
FFHATALVQSSTPATKFLDLLLPWLGAMTEYLKAMAQQVDDGKYSSEGSNMAMQLVAIQN
IADASADQGVSADFIHPMRDFVQRAVDAGHGSDDISALISFMKS

7. Protein Crystallization of EneIRED-07; Data Collection and Refinement

7.1. Gene expression and protein purification

The LIC3C plasmid containing the gene for EneIRED-07 was used to transform *E. coli* BL21 (DE3) competent cells for gene expression. Pre-cultures were grown in LB-medium (10 mL) containing 30 µg/mL kanamycin for 18 h at 37 °C with shaking at 180 r.p.m. 1 L volume cultures were inoculated with the pre-culture (10 mL) and incubated at 37 °C, with shaking at 180 r.p.m. until an OD600 of 0.6-0.8 was reached. Gene expression was induced by addition of IPTG (1 mM) and shaking was continued overnight at 16 °C at 180 r.p.m. The cells were then harvested by centrifugation at 5000 x g for 20 min and resuspended in 50 mM Tris buffer pH 7.1, containing 300 mM NaCl. Cells were disrupted using a French press, and the suspension was centrifuged at 15,000 x g for 40 min to yield a clear CFE lysate. The CFE lysate was loaded onto a pre-equilibrated Ni-NTA column, followed by washing with a load buffer (50 mM Tris-HCl, 300 mM NaCl, pH 7.1). The bound protein was eluted using the same buffer with a linear gradient of 0-500 mM imidazole. EneIRED-07 fractions were pooled, concentrated and loaded onto a HiLoad 16/600 Superdex 75 gel filtration column pre-equilibrated with 50 mM Tris-HCl, 300 mM NaCl pH 7.1 buffer. The concentrated protein sample after gel filtration was used for crystallization screening.

7.2. Protein crystallization

Initial screening of crystallization conditions was performed using commercially available INDEX (Hampton Research), PACT premier and CSSI/II (Molecular Dimensions) screens in 96-well sitting drop trays. Optimization was carried out in a 48-well sitting-drop format to obtain crystals for X-ray diffraction studies. For co-crystallization experiments, a 0.1 M stock solution of cofactor $NADP^+$ in water was prepared. Crystals of the EneIRED-07-$NADP^+$ complex were grown using EneIRED-07 concentrated to 25 mg/mL in 50 mM Tris buffer at pH 7.1 containing 300 mM NaCl. The crystallization drop contained 0.15 µL protein: 0.15 µL mother liquor, comprising 0.1 M Tris pH 8.5, 20% PEG (polyethylene glycol) 3350 w/v, 0.1 M $MgCl_2$, and 2.0 mM $NADP^+$. Crystals were harvested directly into liquid nitrogen with nylon CryoLoops™ (Hampton Research), using the mother liquor without any further cryoprotectant.

7.3. Data collection, structure solution and refinement

The dataset described in this report was collected at the Diamond Light Source, Didcot, Oxfordshire, U.K. on beamline I03. Data were processed and integrated using XDS[7] and scaled using SCALA[8]

included in the Xia2 processing system[9]. Data collection statistics are provided in Table 7. The crystal of EneIRED-07-$NADP^+$ was obtained in space group P21212, with six molecules in the asymmetric unit; the solvent content in the crystals was 48%. The structure of EneIRED-07-$NADP^+$ was solved by molecular replacement using MOLREP[10] with the monomer of *R*-IRED-*Sr* (PDB code 5OCM[11]) as the model. The structure was built and refined using iterative cycles in Coot[12] and REFMAC[13], employing local NCS restraints in the refinement cycles. Following building and refinement of the protein and water molecules in this complex, residual density was observed in the omit maps at the dimer interfaces, which could be clearly modelled as $NADP^+$. The final structures exhibited % R_{cryst}/R_{free} values of 26.0/30.7. Refinement statistics for the structures are presented in Table S7. The Ramachandran plot for EneIRED-07-$NADP^+$ showed 93.3% of residues to be situated in the most favoured regions, 6.0% in additional allowed and 0.7% residues in outlier regions. The structure has been deposited in the Protein Databank (PDB) with accession code 7OSN.

7.4. Modelling

Automated docking was performed using AUTODOCK VINA 1.1.2[14]. Coordinates for the ligand were prepared using the ligand builder in COOT[12] with the relevant topology files generated by Lidia within the COOT program. The appropriate pdbqt files for the dimeric model of EneIRED-07 and the ligand were prepared in AUTODOCK Tools. The active site of EneIRED-07 was contained in a grid size of 12 Å × 12 Å × 12 Å (corresponding to x, y, z) with 1 Å spacing, centred around the catalytic centre at positions 9.89 Å × 47.91 Å × -41.30 Å (corresponding to x, y, z), which was generated using AutoGrid in the AUTODOCK Tools interface. The dockings were performed by VINA, therefore the posed dockings were below 2 Å r.m.s.d. The results generated by VINA were visualised in AUTODOCK Tools 1.5.6 where the ligand conformations were assessed based upon lowest VINA energy. The model that features the (*R*)-enantiomer of **3f** is illustrated in Figure S13.

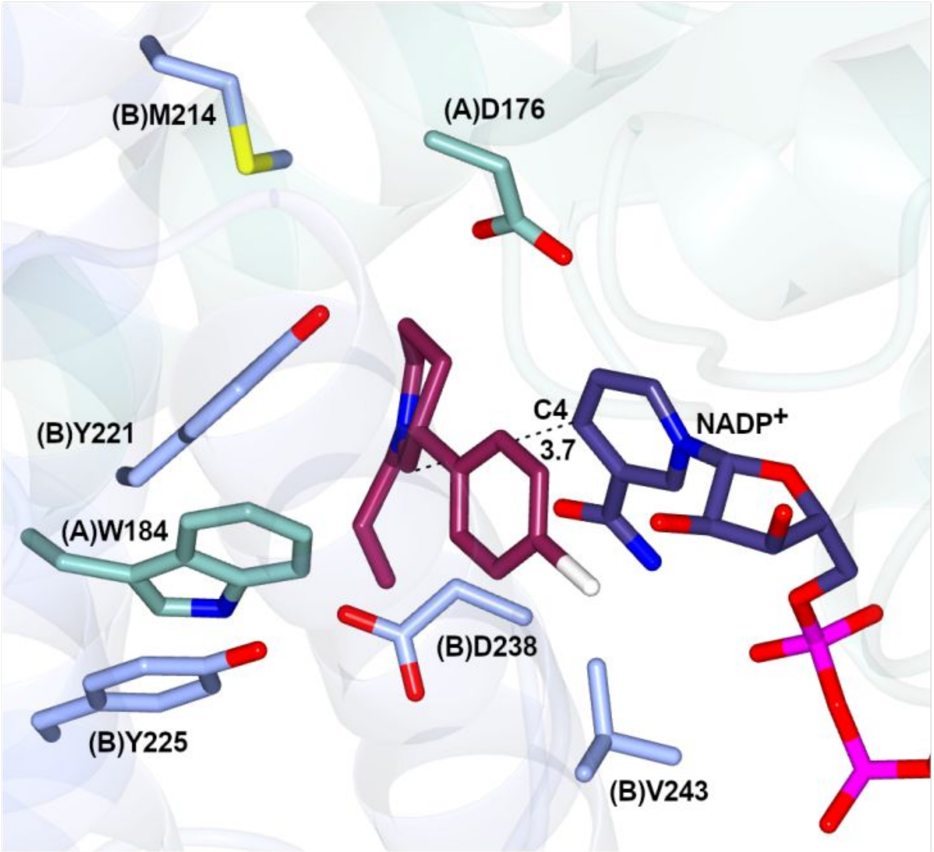

Figure S13: Active site of EneIRED-07 with the (*R*)-enantiomer of intermediate **3f** (where R^1 = allyl and R^2 = *para*-bromophenyl) modelled into the active site. $NADP^+$ and (*R*)-**3f** are shown in cylinder format with carbon atoms in grey and purple respectively.

Table S7: Data Collection and Refinement Statistics for EneIRED-07 in complex with $NADP^+$

	**EneIRED-07-NADP$^+$**
Beamline	Diamond I03
Wavelength (Å)	0.97625
Resolution (Å)	50.00-2.55 (2.61-2.55)
Space Group	*P* 2_12_12
Unit cell (Å)	a = 50.00; b = 142.00; c = 266.00
	α = γ = β = 90.00 °
No. of molecules in the asymmetric unit	6
Unique reflections	63163 (4397)
Completeness (%)	100 (100)
R_{merge} (%)	0.12 (0.91)
$R_{p.i.m.}$	0.04 (0.26)

Multiplicity	12.9 (13.4)
$<I/\sigma(I)>$	10.2 (1.8)
Overall *B* factor from Wilson plot (Å^2)	55
$CC_{1/2}$	1.00 (0.97)
R_{cryst}/R_{free} (%)	26.0/30.7
r.m.s.d 1-2 bonds (Å)	0.08
r.m.s.d 1-3 angles (°)	1.55
Avge main chain B (Å^2)	80
Avge side chain B (Å^2)	81
$NADP^+$ B (Å^2)	66
Avge water B (Å^2)	56

Numbers in brackets refer to data for highest resolution shells.

8. GCMS Analysis: methods and retention times for THP substrates and piperidine products

Table S8: GCMS analysis: methods and retention times of THP substrates and piperidine products from biotransformations.

THP	Piperidine	Column	Oven temp.	THP retention time (min)	Piperidine retention time (min)	
					T[1]	T[2]
**1b**	**1c**	HP-1MS	**Method 1**	17.6	16.8	
**2b**	**2c**	HP-1MS	**Method 2**	11.8	11.6	
**3b**	**3c**	HP-1MS	**Method 1**	18.2	17.4	
**4b**	**4c**	HP-1MS	**Method 1**	18.5	17.5	
**5b**	**5c**	HP-1MS	**Method 1**	14.2	13.2	
**6b**	**6c**	HP-1MS	**Method 1**	14.1	13.2	
**7b**	**7c**	HP-1MS	**Method 1**	17.1	16.1	
**8b**	**8c**	HP-1MS	**Method 1**	12.3	10.9	
**9b**	**9c**	HP-1MS	**Method 1**	14.4	13.1	
**10b**	**10c**	HP-1MS	**Method 1**	20.1	19.2	
**11b**	**11c**	HP-1MS	**Method 1**	13.2	12.1	
**12b**	**12c**	HP-1MS	**Method 1**	15.6	14.6	
**13b**	**13c**	HP-1MS	**Method 1**	5.7	4.8	
**14b**	**14c**	HP-1MS	**Method 1**	13.2	12.5	
**15b**	**15c**	HP-1MS	**Method 1**	5.7	4.8	
**16b**	**16c**	HP-1MS	**Method 1**	14.5	13.4	
**17b**	**17c**	HP-1MS	**Method 1**	7.2	6.0	6.1
**18b**	**18c**	HP-1MS	**Method 1**	11.1	9.9	10.1
**19b**	**19c**	HP-1MS	**Method 1**	13.9	13.4	13.7
**20b**	**20c**	HP-1MS	**Method 1**	14.8	14.3	14.6
**21b**	**21c**	HP-1MS	**Method 1**	15.0	14.4	14.8

Injector temperature: 200 °C, detector temperature: 270 °C, helium flow: 1.2 mL/min. **Method 1:** Hold 50 °C for 2 mins, 50 – 300 °C, 10 °C/min, hold 300 °C for 1 min; **Method 2:** Hold 50 °C for 1 min, 50 – 70 °C, 7 °C/min, 70 – 270 °C, 30 °C/min, 270 – 320 °C, 40 °C/min, hold 320 °C for 2 mins.

Table S9: GCMS analysis: methods and retention times of piperidine products from biotransformations.

Piperidine product	Column	Oven temp.	Piperidine retention time (min)
**22**	HP-1MS	**Method 1**	17.3
**26**	HP-1MS	**Method 1**	15.8
**23**	HP-1MS	**Method 1**	20.3
**27**	HP-1MS	**Method 2**	8.8
**24**	HP-1MS	**Method 2**	10.4
**10c-d_1**	HP-1MS	**Method 1**	19.1
**10c-d_2**	HP-1MS	**Method 1**	19.0

Injector temperature: 200 °C, detector temperature: 270 °C, helium flow: 1.2 mL/min. **Method 1:** Hold 50 °C for 2 mins, 50 – 300 °C, 10°C/min, hold 300 °C for 1 min; **Method 2:** Hold 50 °C for 1 min, 50 - 70 °C, 7 °C/min, 70 – 270 °C, 30 °C/min, 270 – 320 °C, 40 °C/min, hold 320 °C for 2 mins.

Table S10: GCMS analysis: methods and retention times of piperidine products from biotransformations.

Other	Column	Oven temp.	retention time (min)
THP-**25**	HP-1MS	**Method 1**	9.4
Enamine-**10e**	HP-1MS	**Method 1**	21.0

Injector temperature: 200 °C, detector temperature: 270 °C, helium flow: 1.2 mL min^{-1}.
Method 1: Hold 50 °C for 2 mins, 50 – 300 °C, 10 °C/min, hold 300 °C for 1 min.

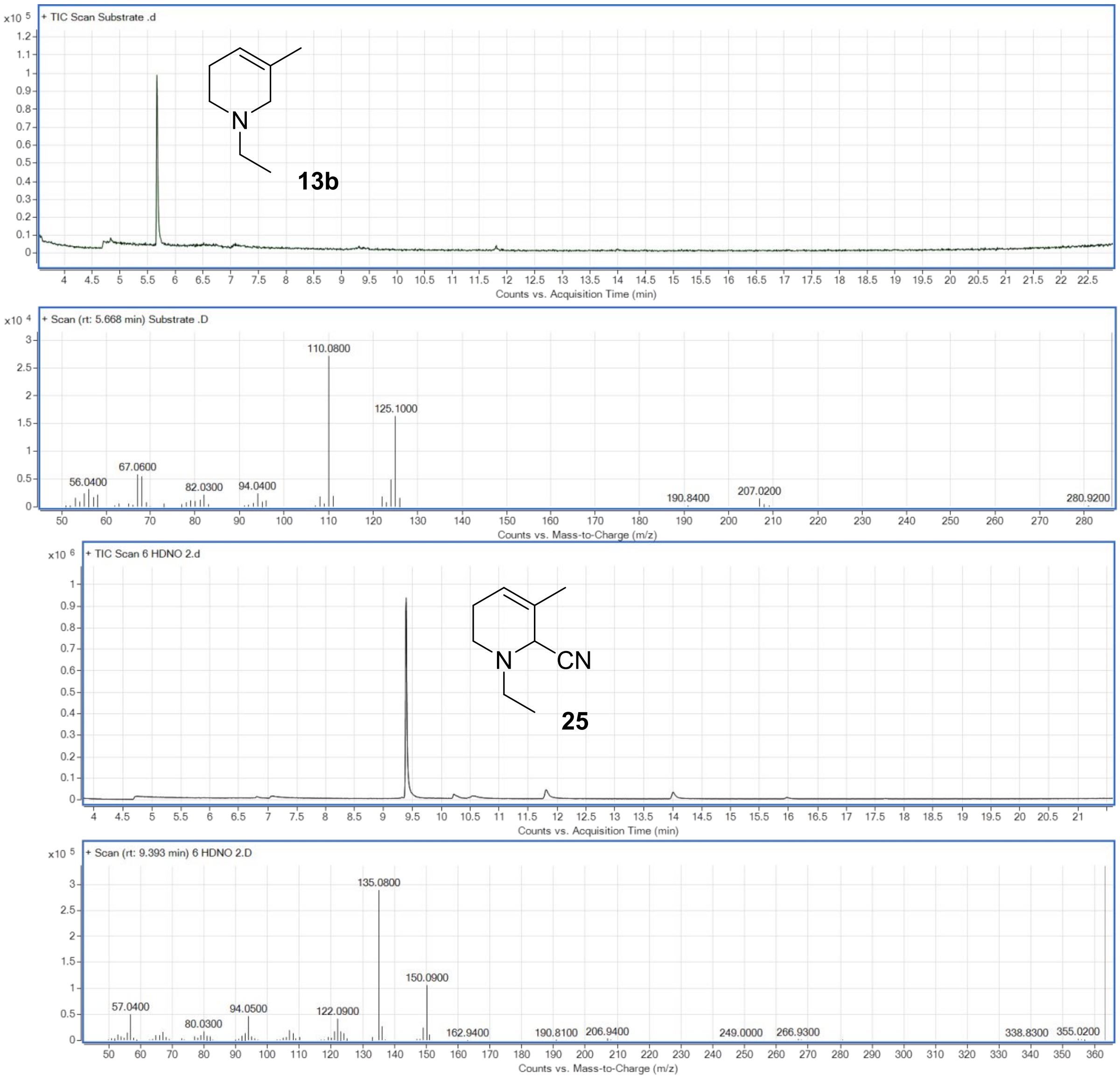

Figure S14: GC-MS spectra for THP-**25**.

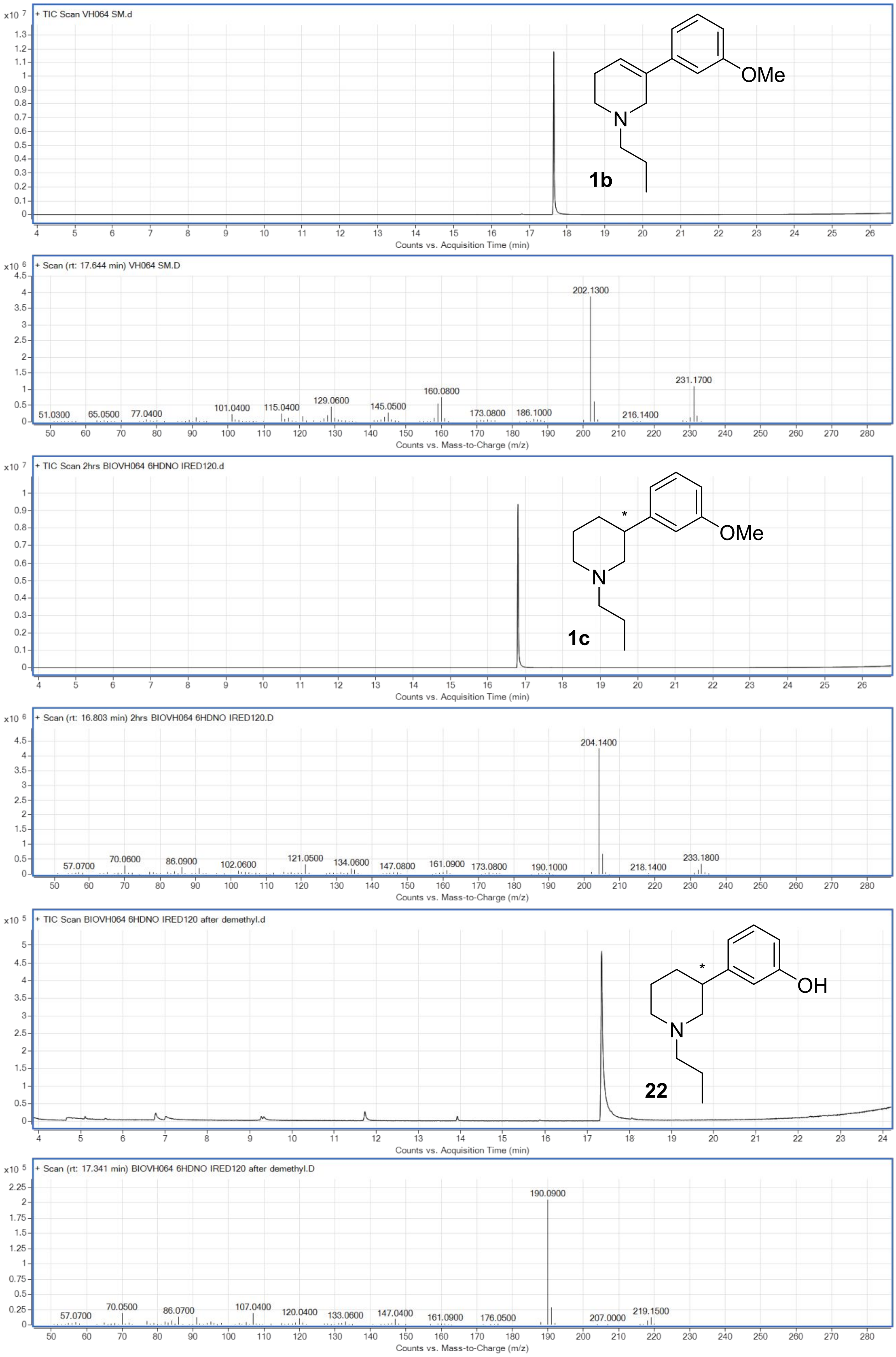

Figure S15: GC-MS spectra for piperidine **1c** and **22**.

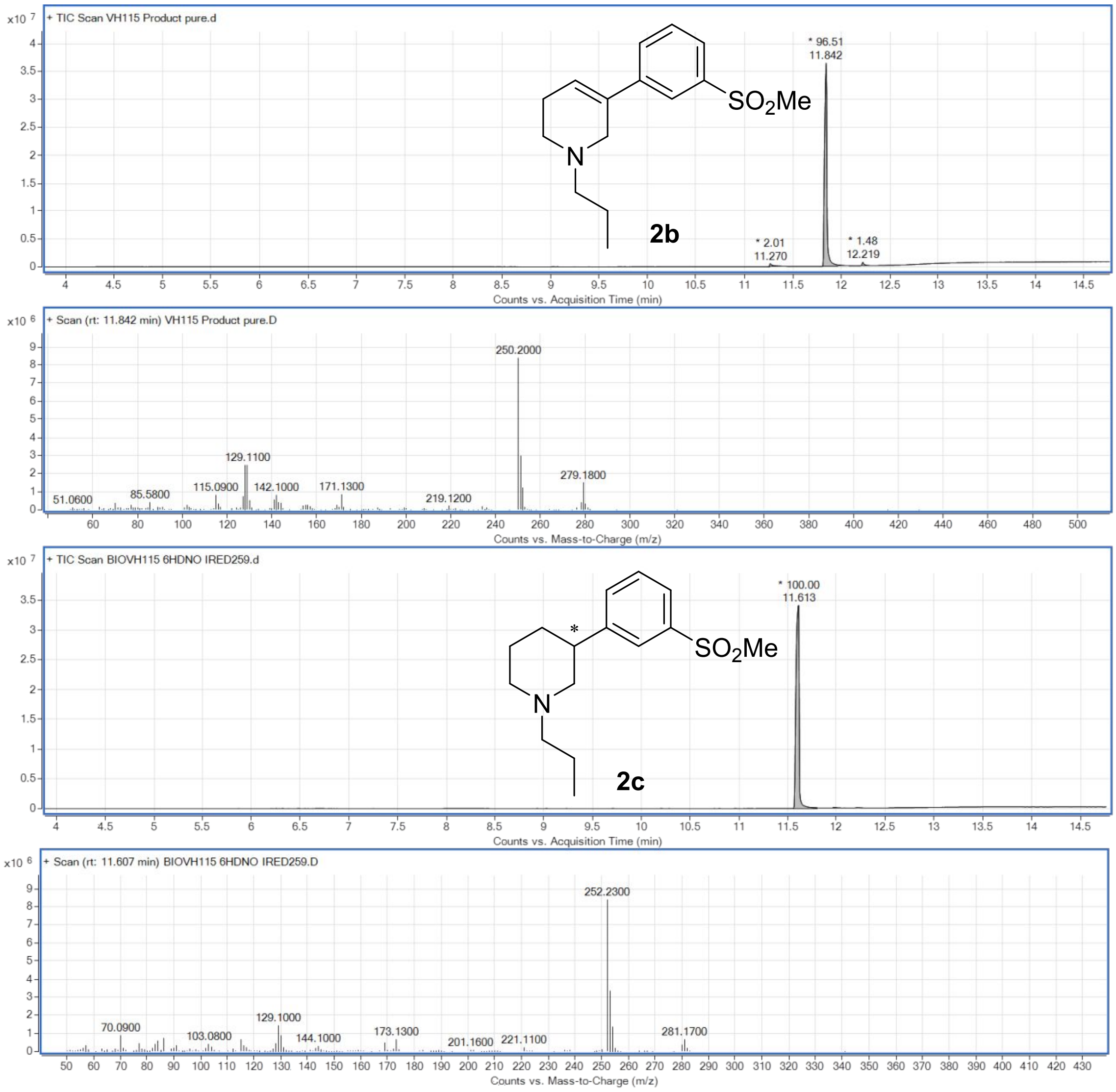

Figure S16: GC-MS spectra for piperidine **2c**.

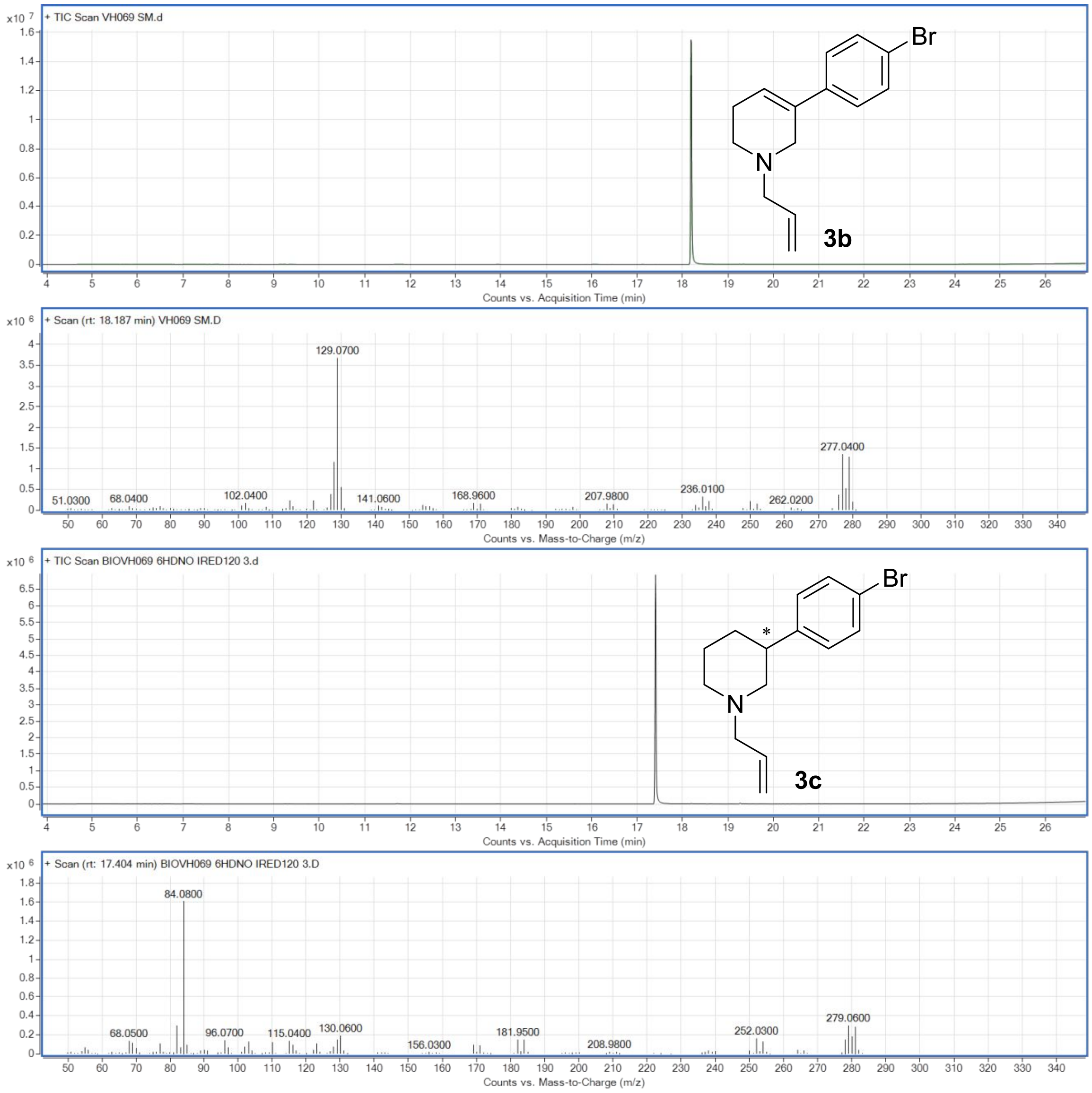

Figure S17: GC-MS spectra for piperidine **3c**.

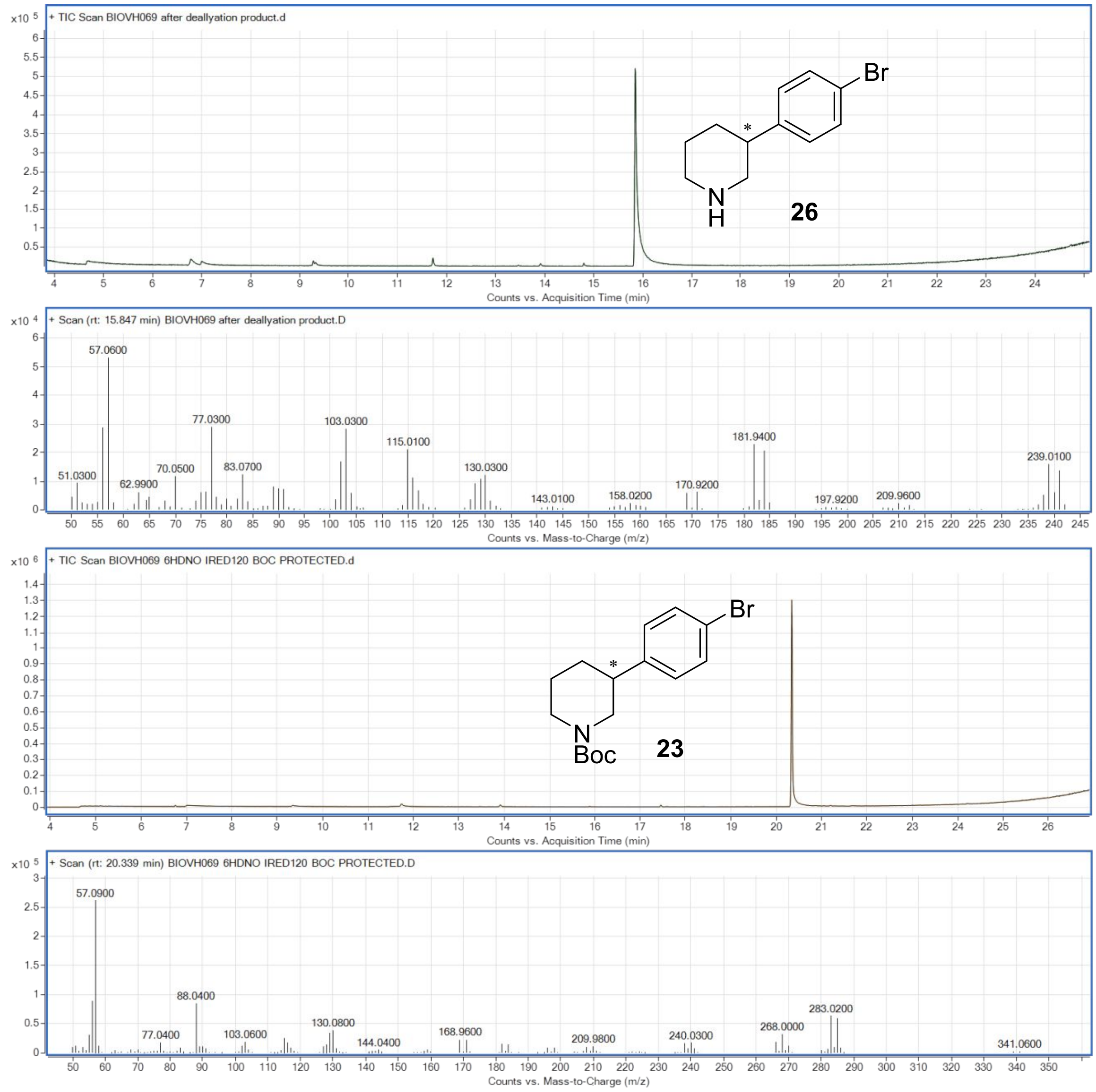

Figure S18: GC-MS spectra for piperidine **26** and **23**.

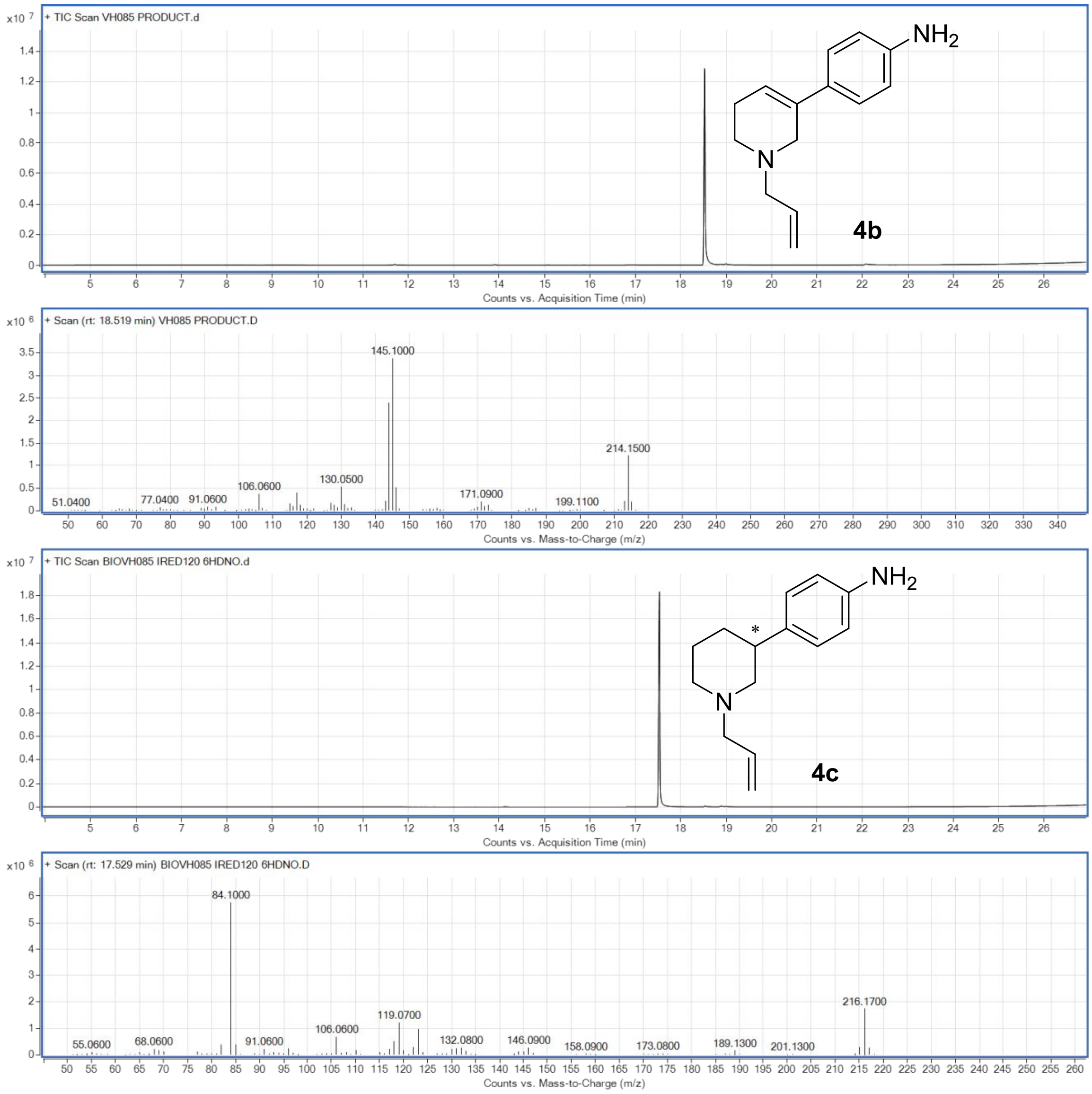

Figure S19: GC-MS spectra for piperidine **4c**.

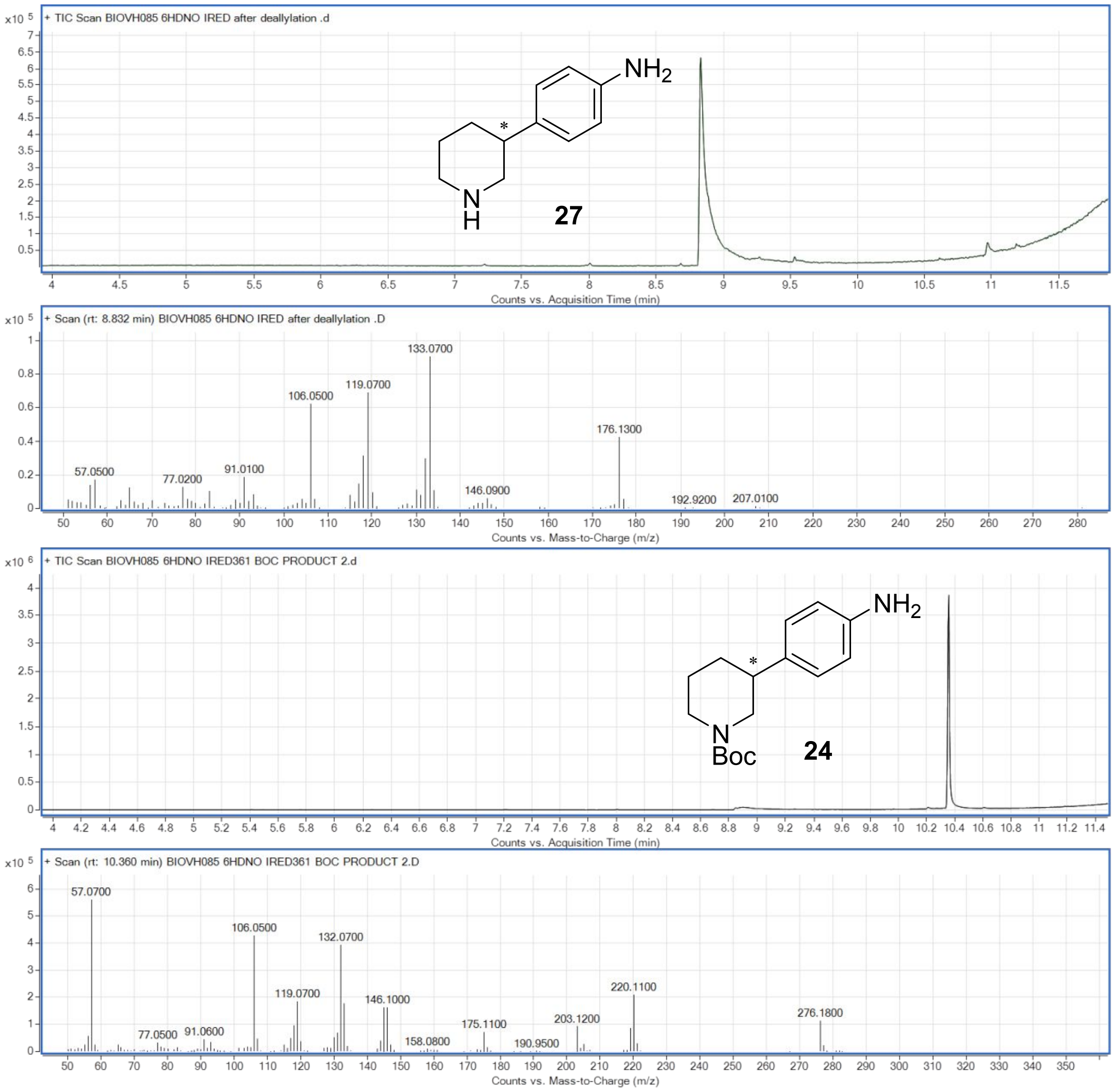

Figure S20: GC-MS spectra for piperidine **27** and **24**.

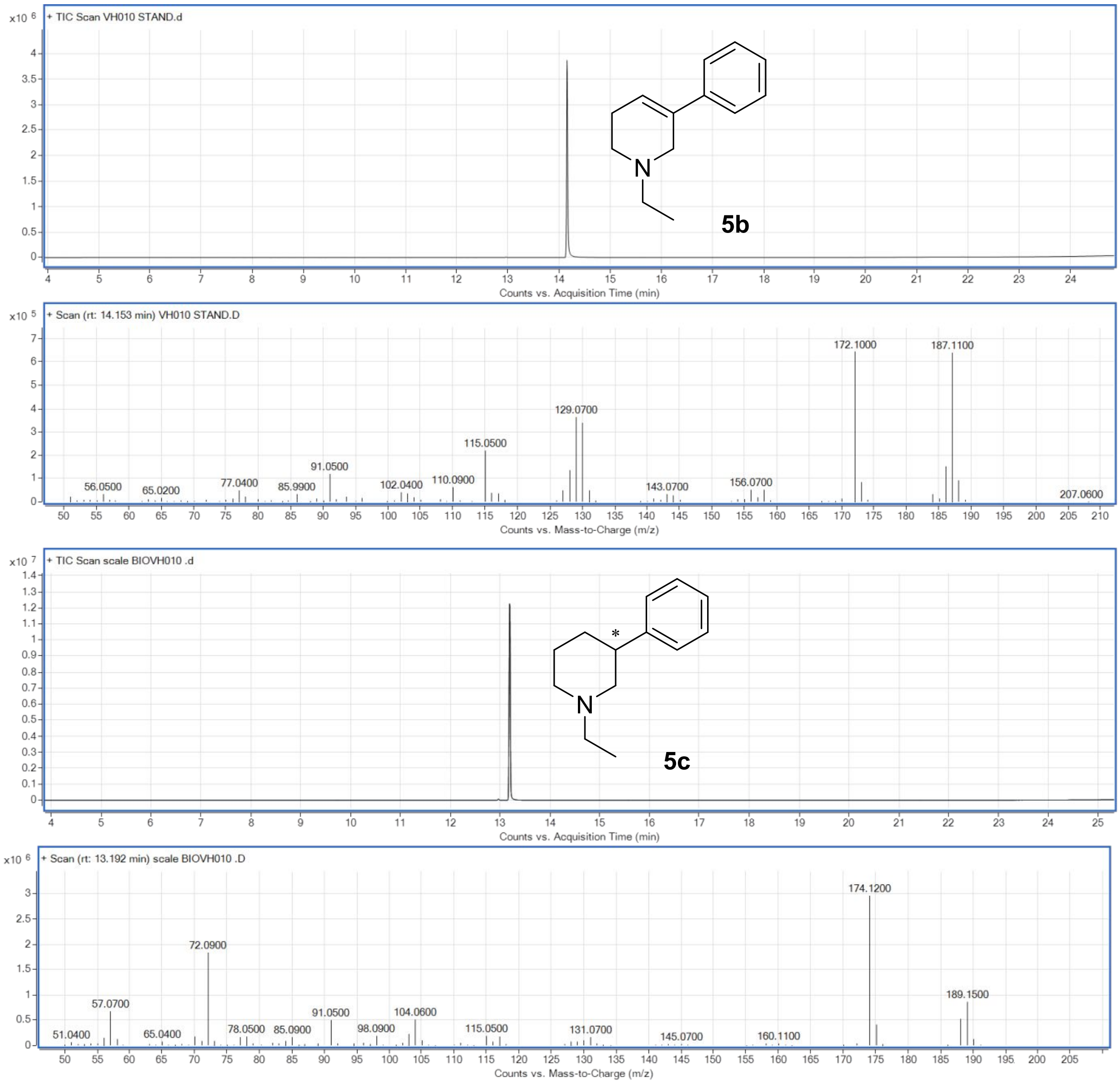

Figure S21: GC-MS spectra for piperidine **5c**.

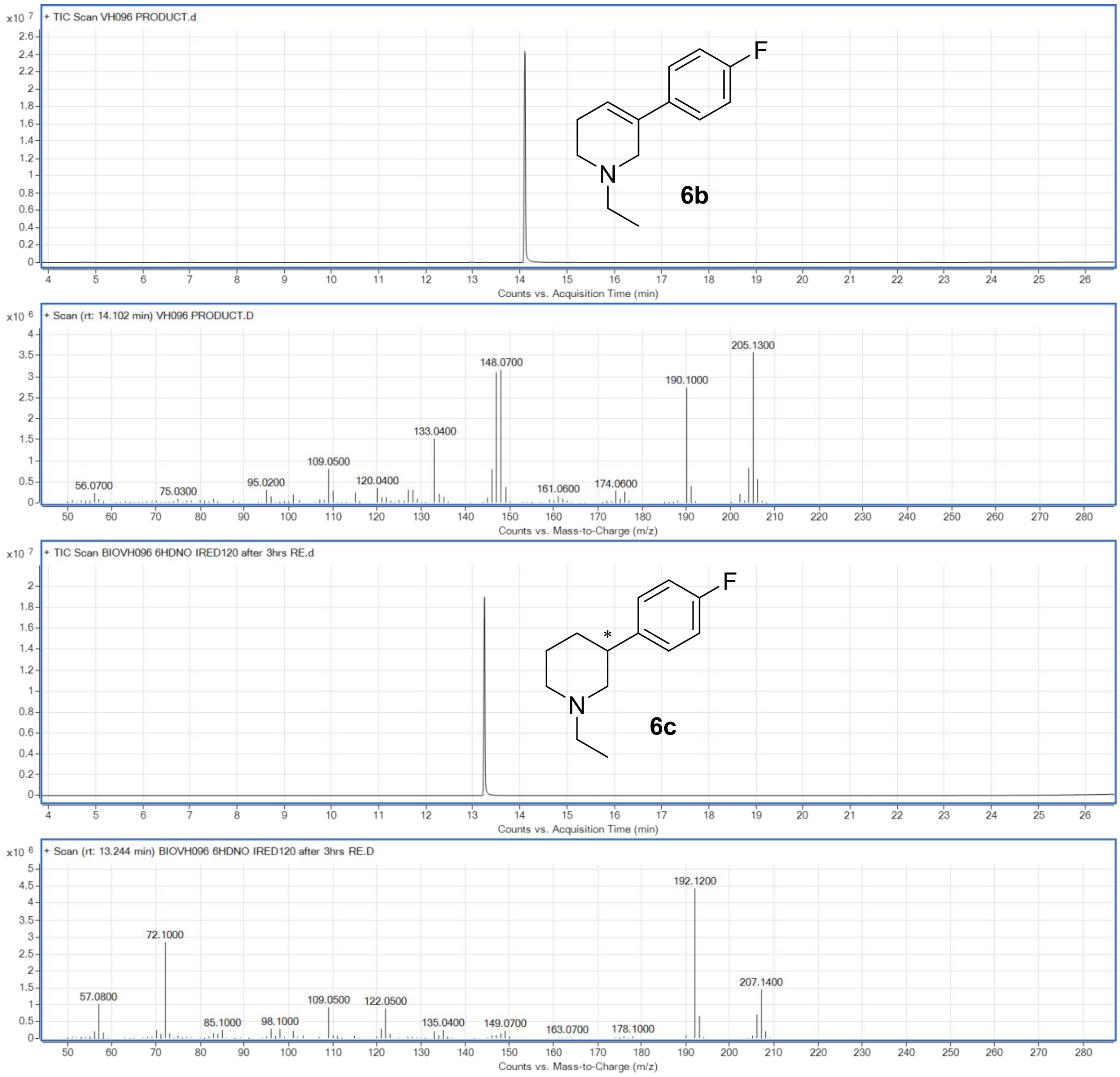

Figure S22: GC-MS spectra for piperidine **6c**.

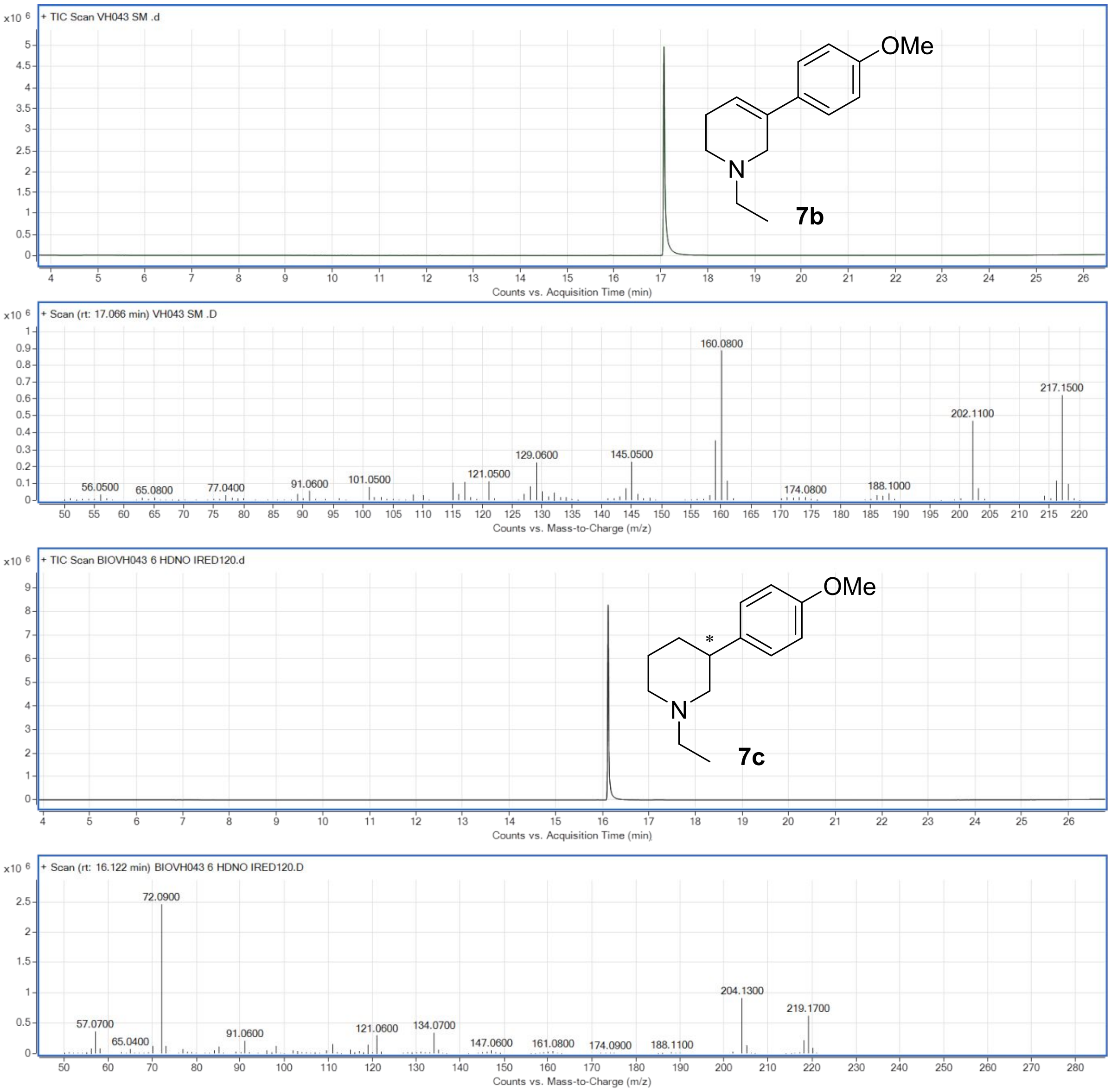

Figure S23: GC-MS spectra for piperidine **7c**.

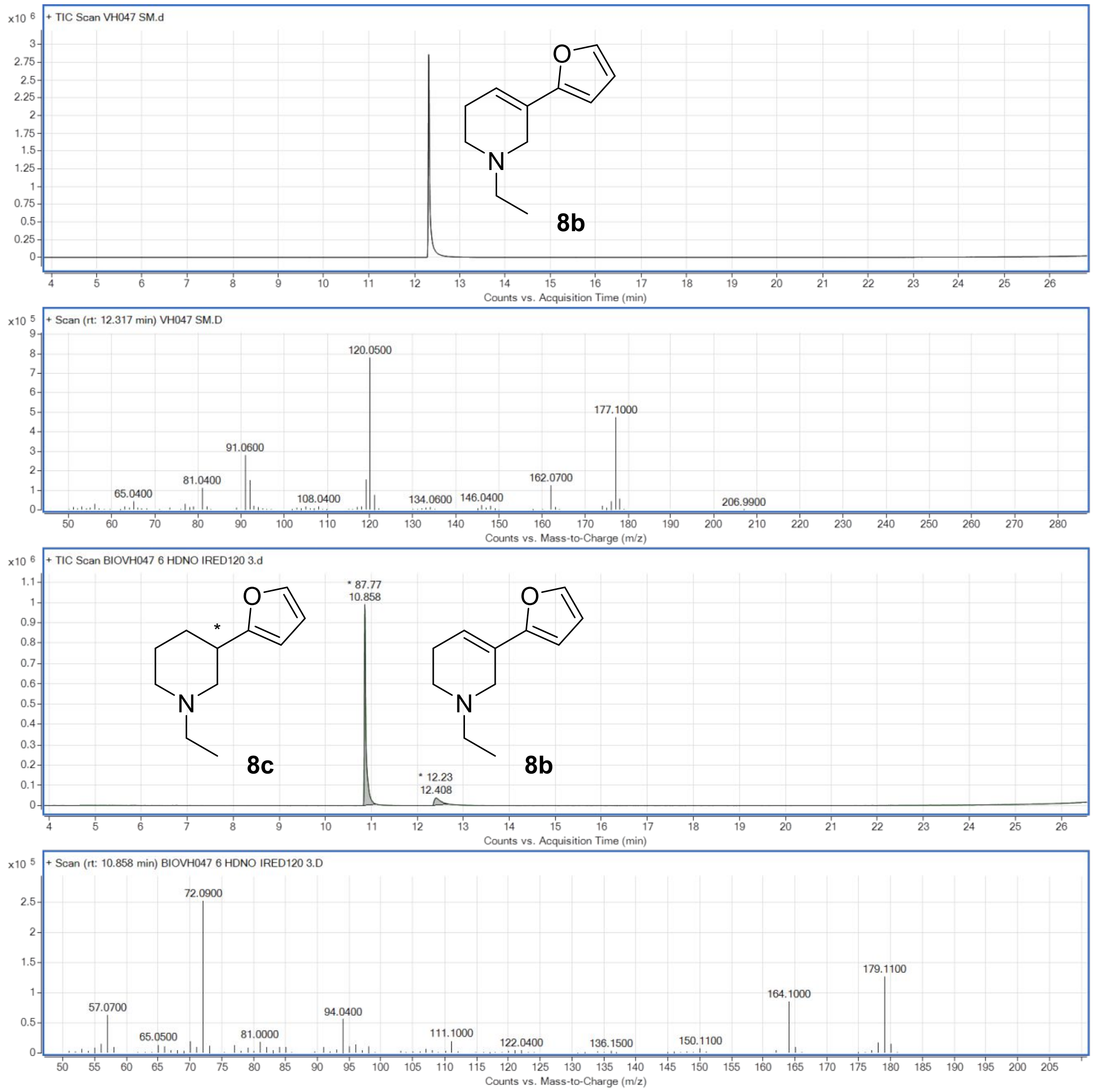

Figure S24: GC-MS spectra for piperidine **8c**.

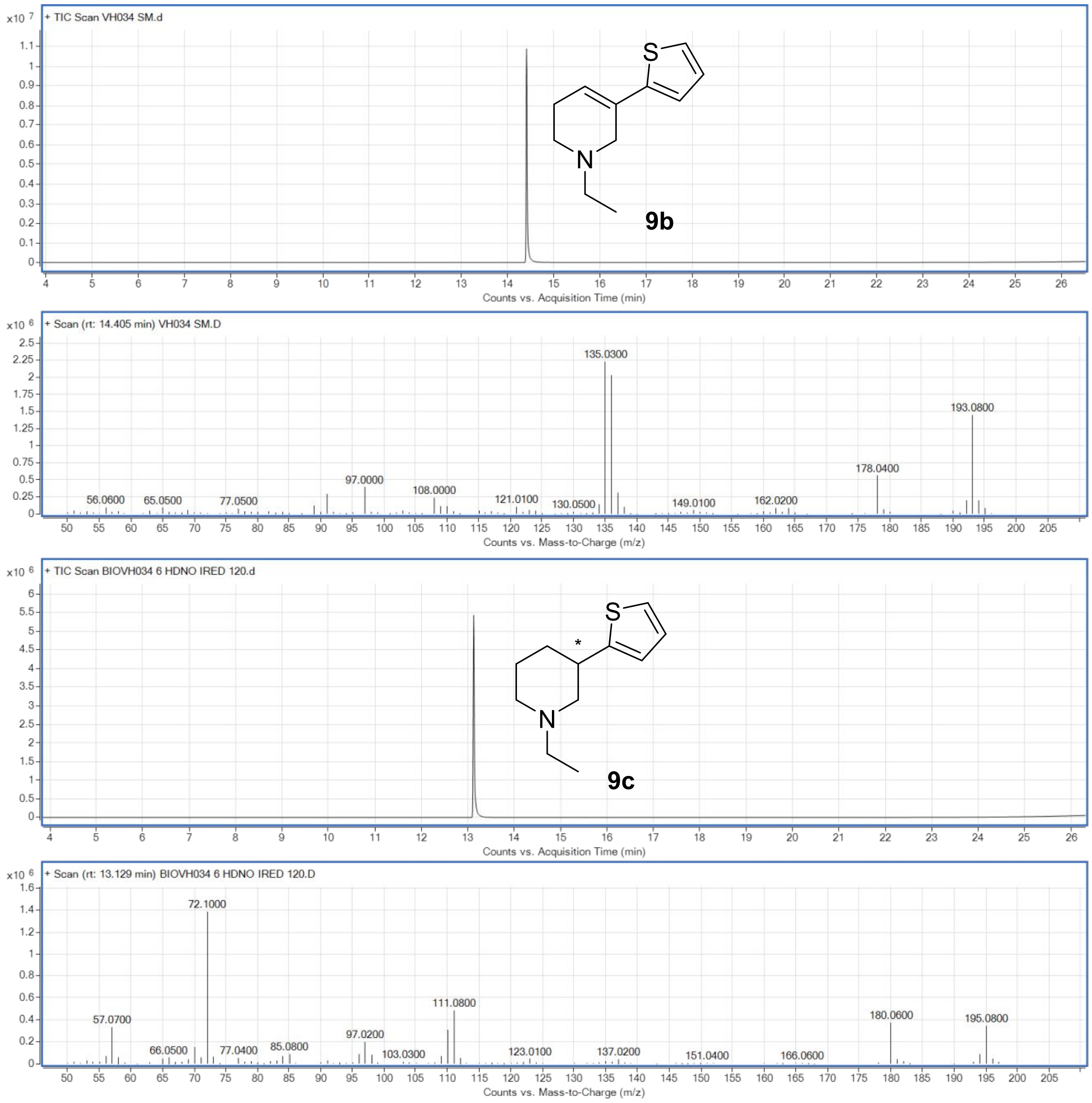

Figure S25: GC-MS spectra for piperidine **9c**.

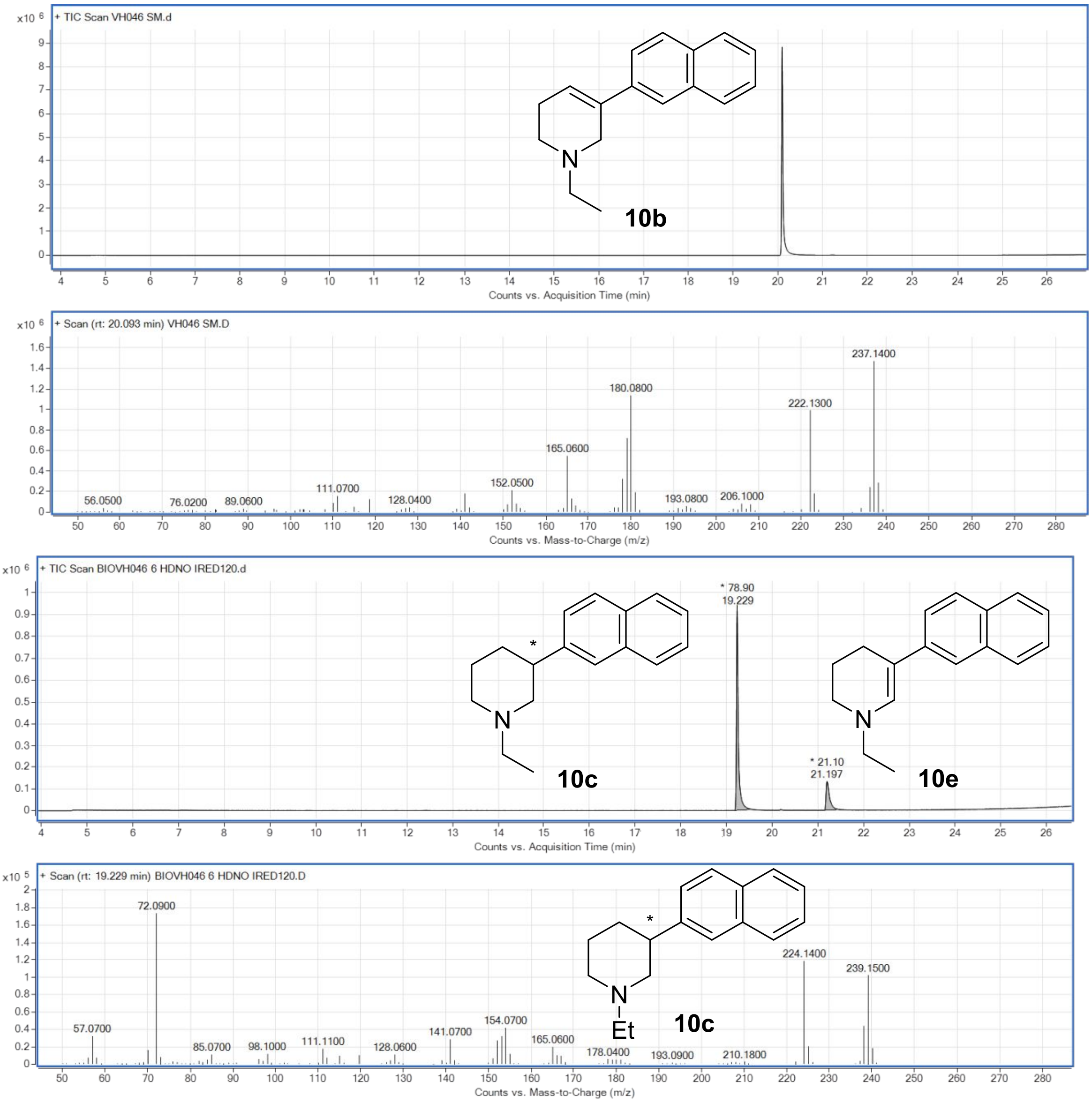

Figure S26: GC-MS spectra for piperidine **10c**.

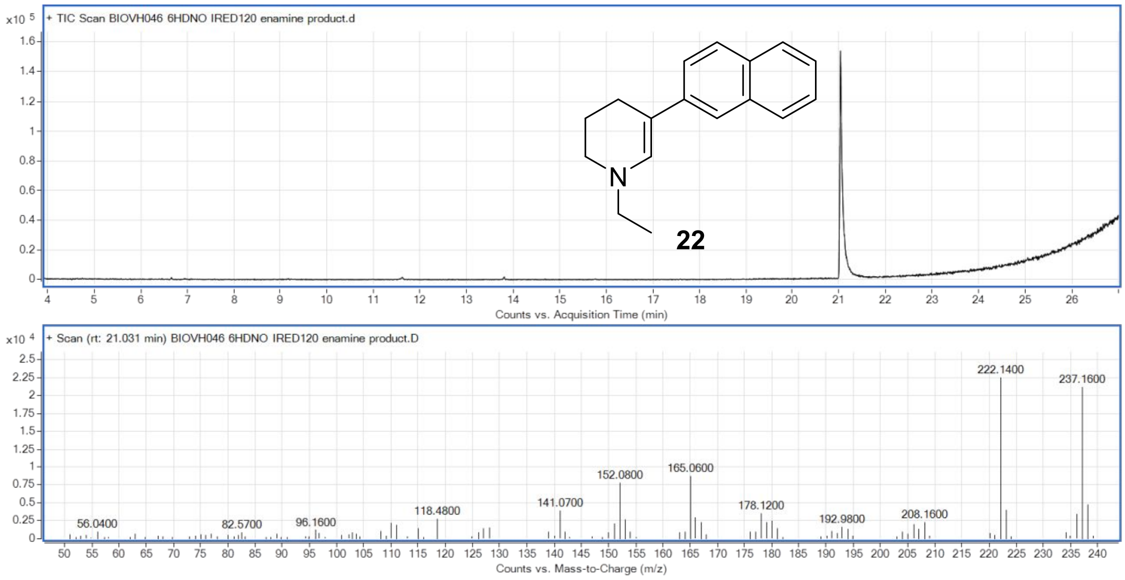

Figure S27: GC-MS spectra for enamine **22**.

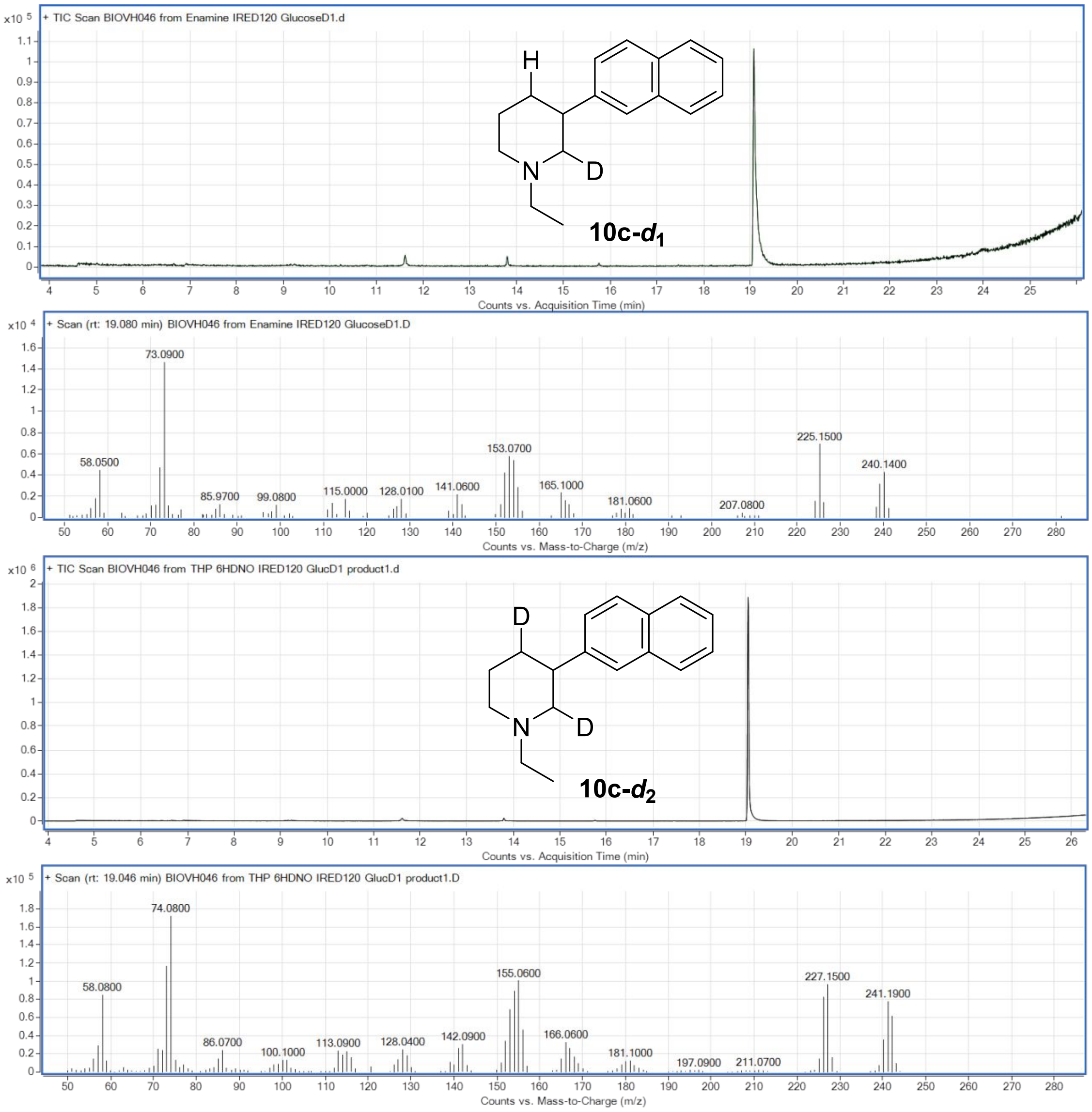

Figure S28: GC-MS spectra for piperidine **10c-d_1** and **10c-d_1**.

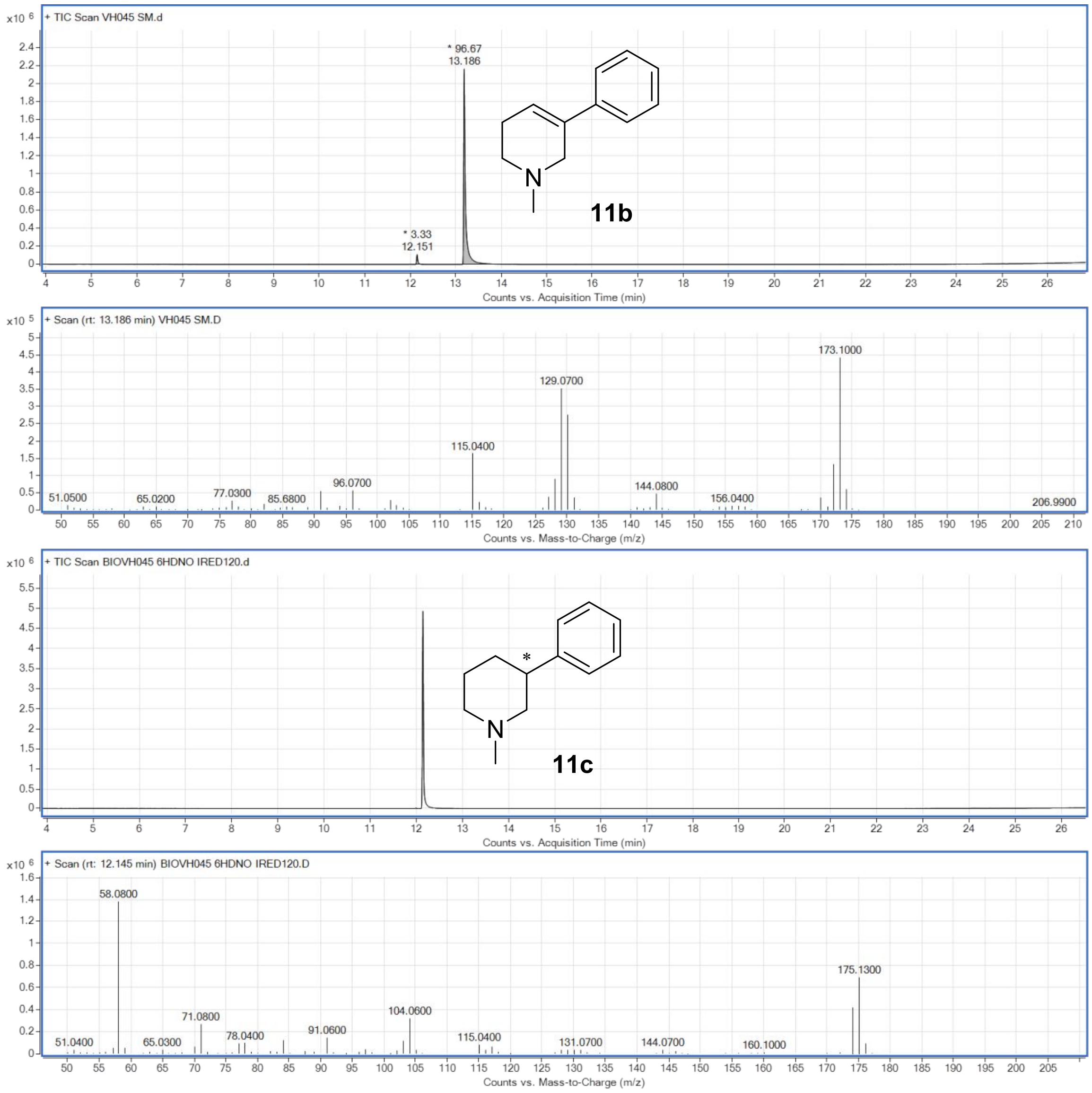

Figure S29: GC-MS spectra for piperidine **11c**.

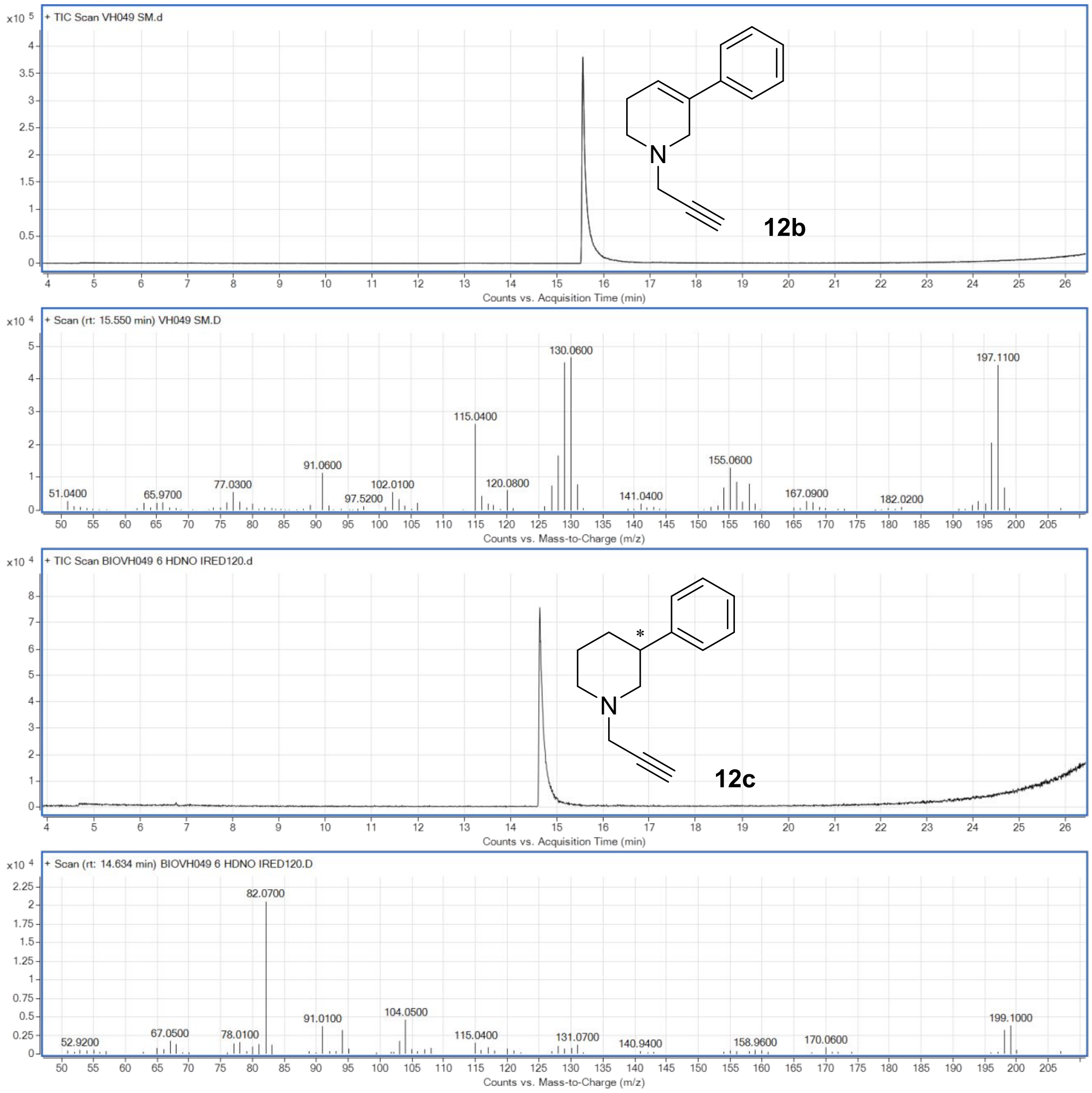

Figure S30: GC-MS spectra for piperidine **12c**.

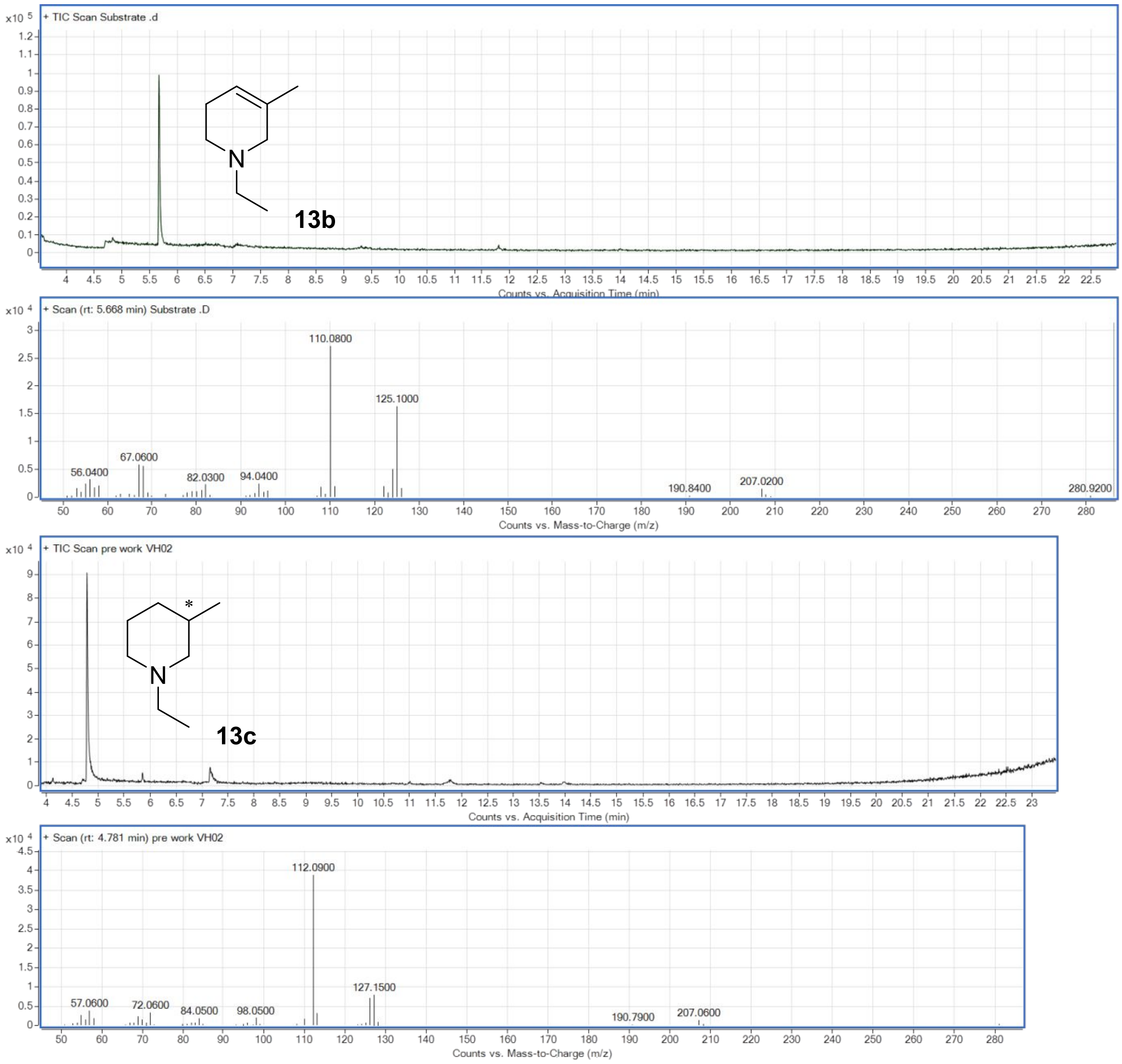

Figure S31: GC-MS spectra for piperidine **13c**.

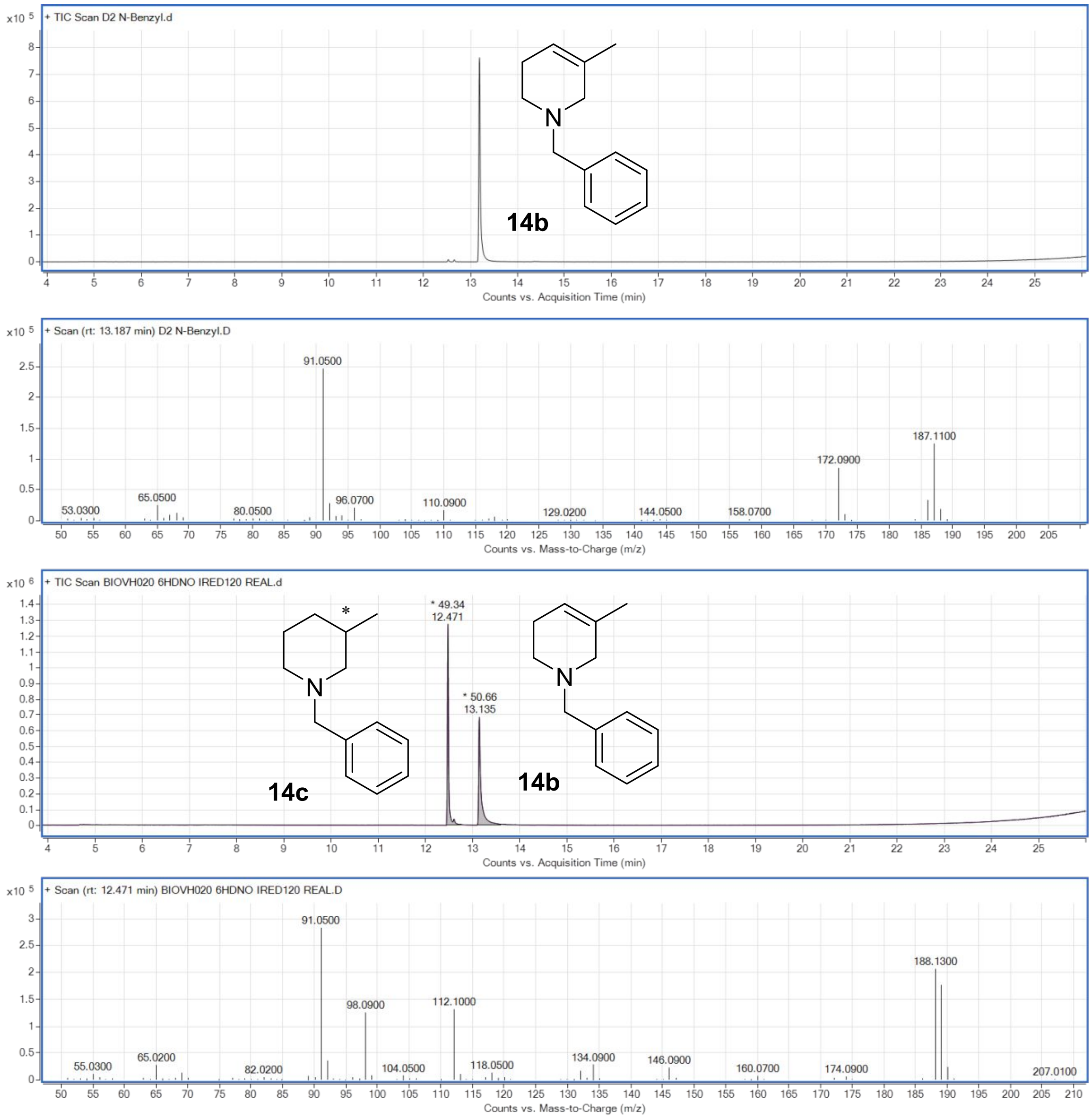

Figure S32: GC-MS spectra for piperidine **14c**.

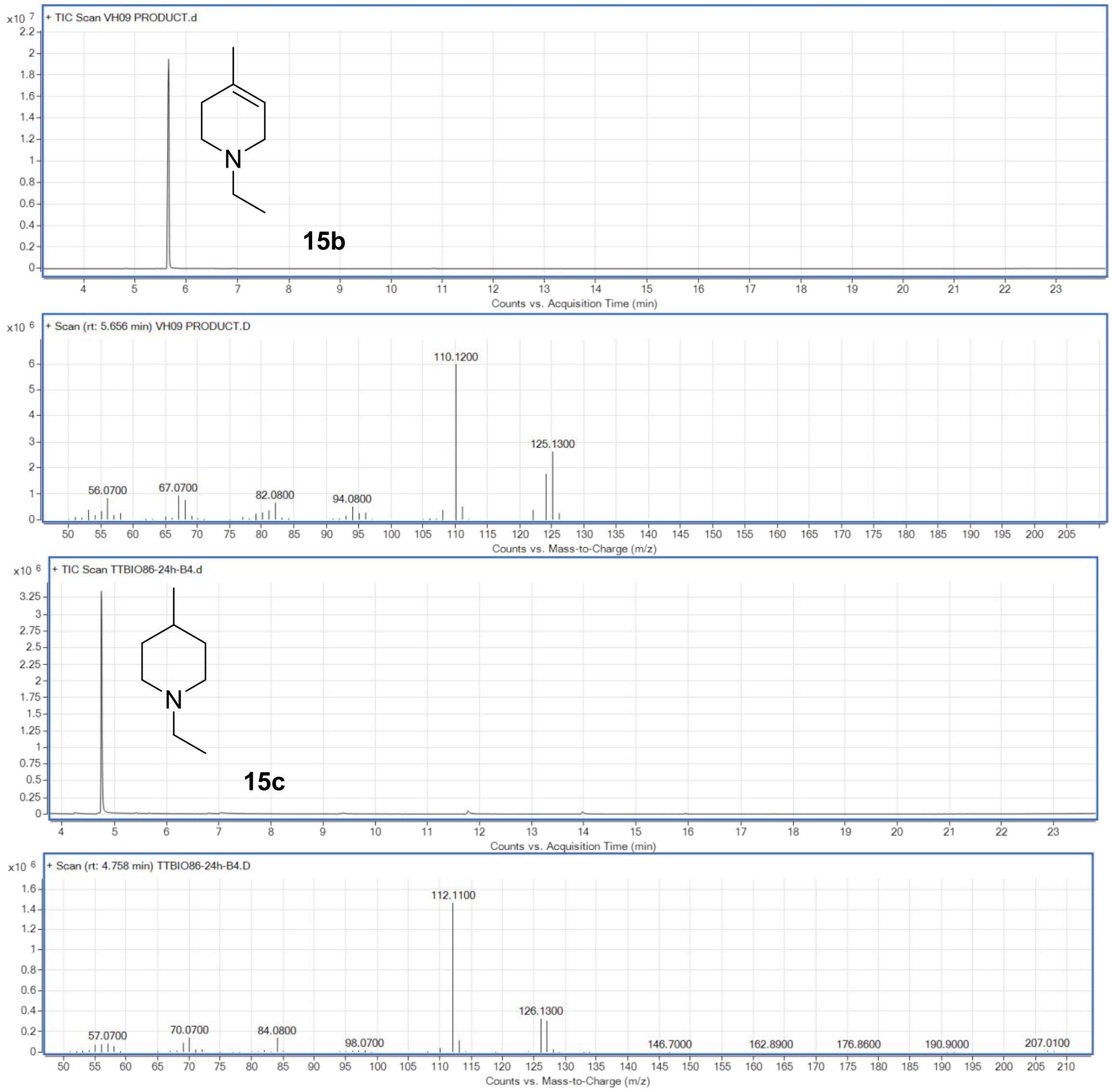

Figure S33: GC-MS spectra for piperidine **15c**.

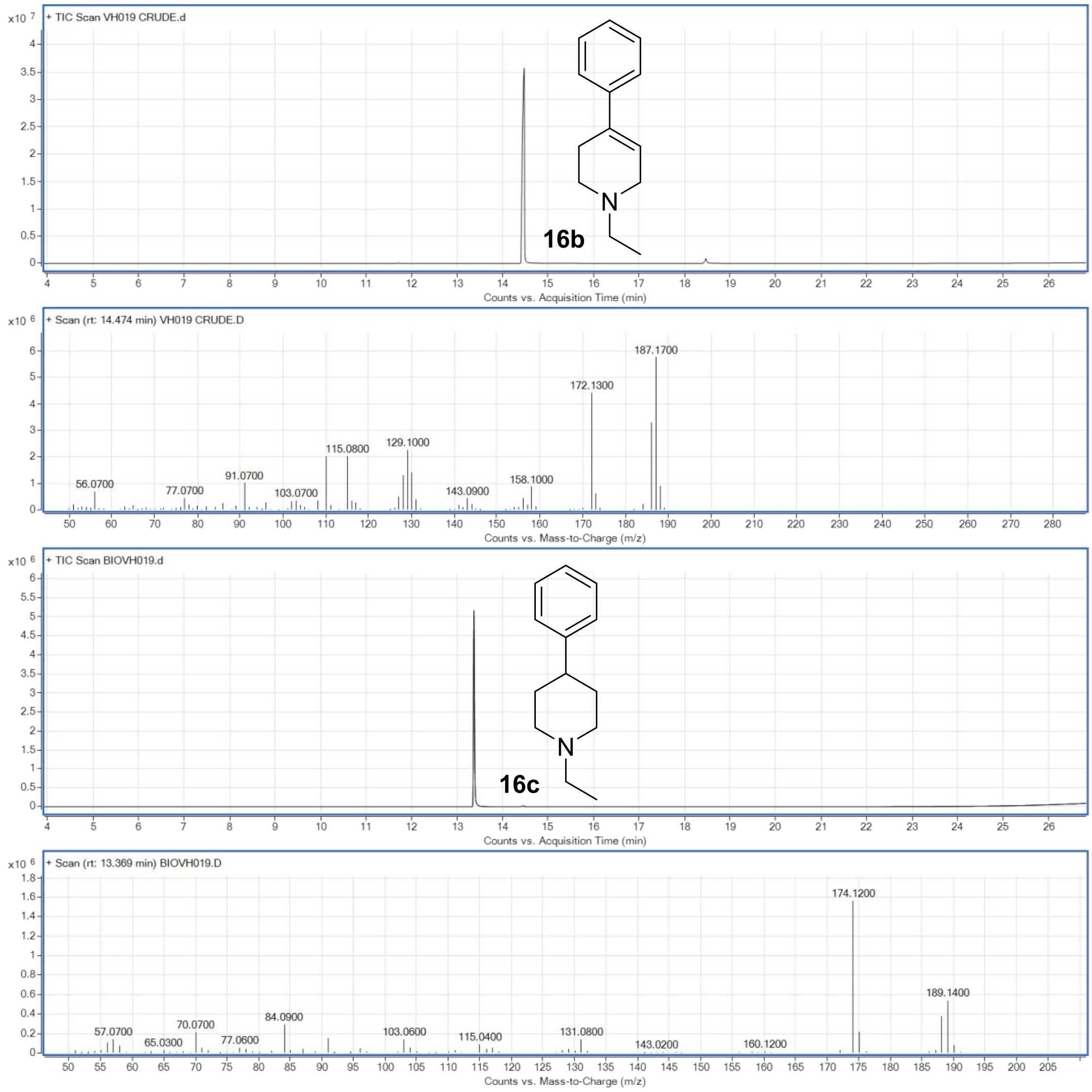

Figure S34: GC-MS spectra for piperidine **16c**.

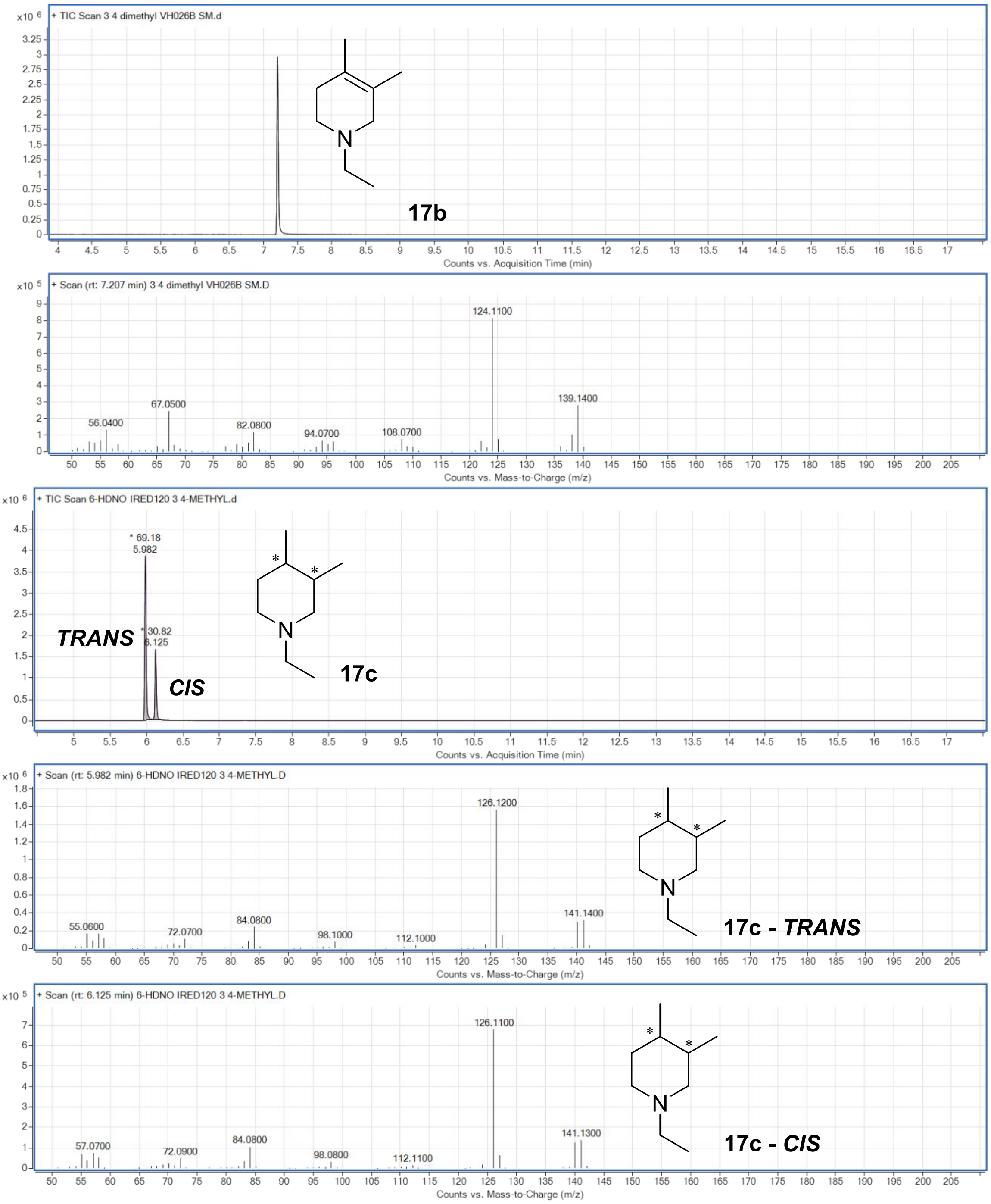

Figure S35: GC-MS spectra for piperidine **17c**.

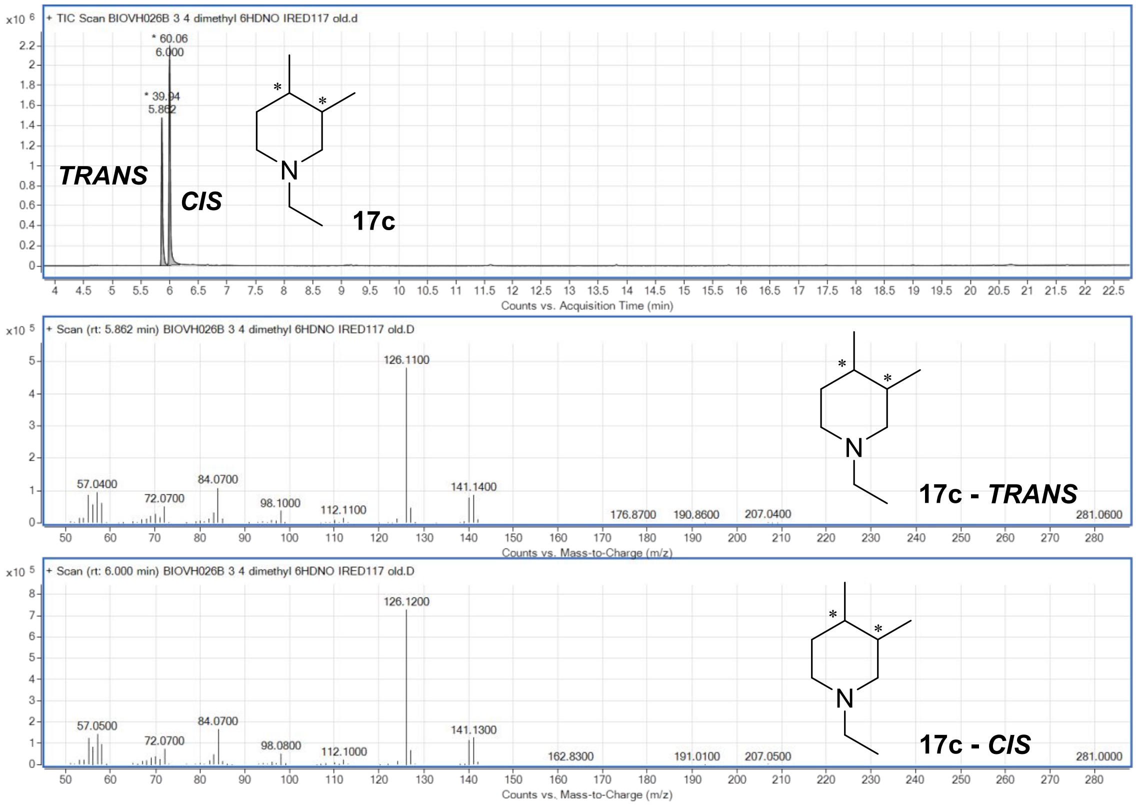

Figure S36: GC-MS spectra for piperidine **17c**.

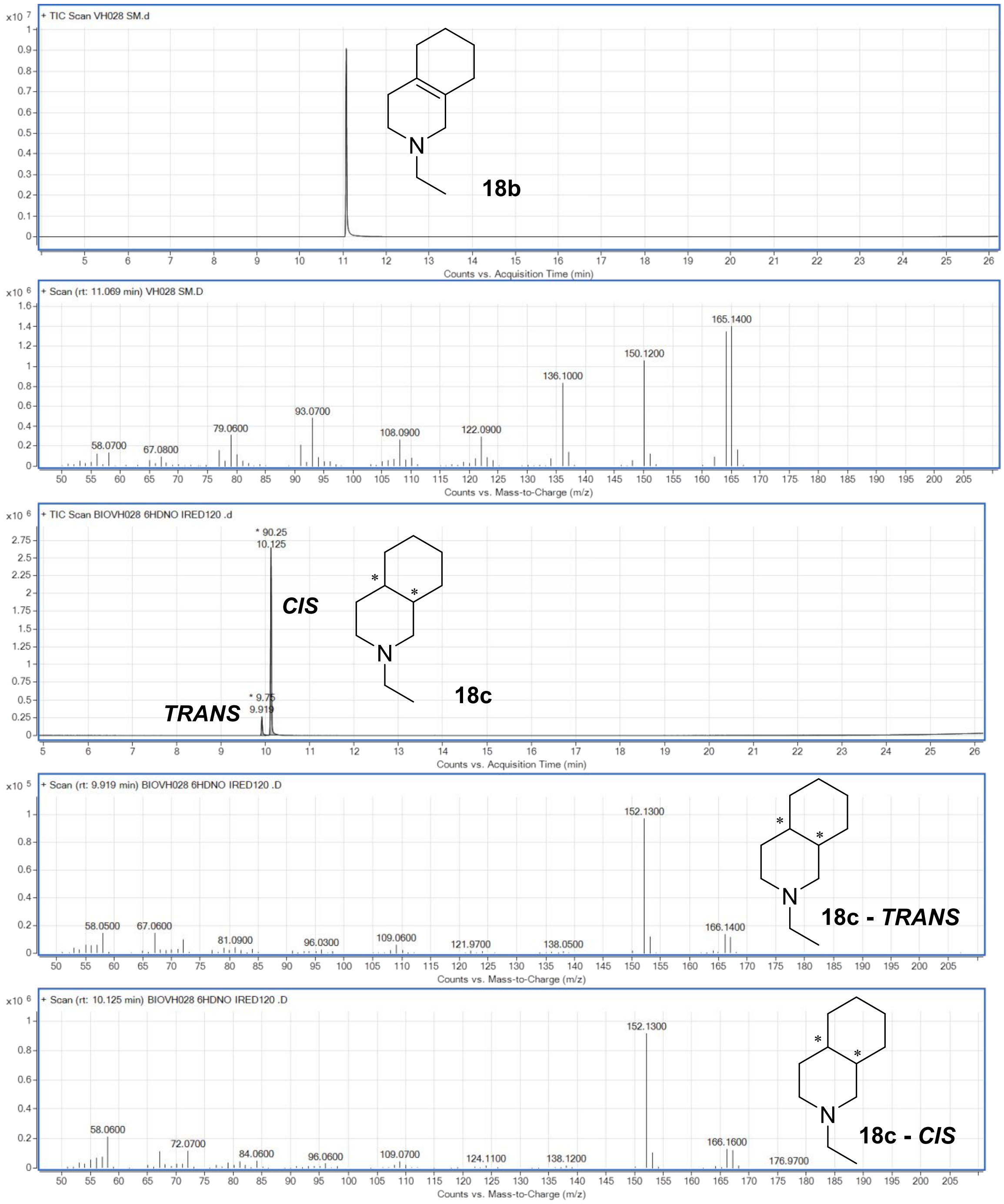

Figure S37: GC-MS spectra for piperidine **18c**.

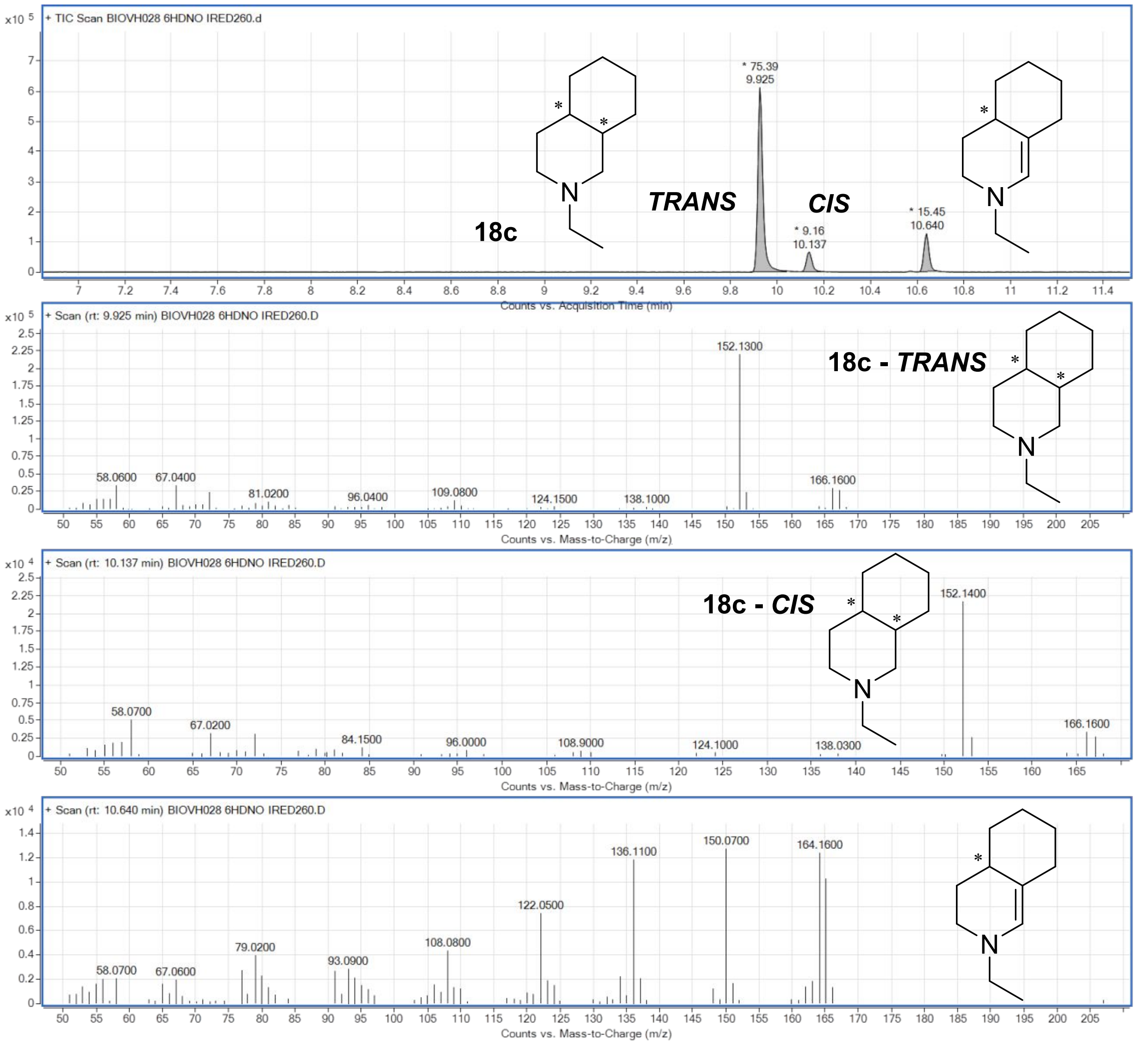

Figure S38: GC-MS spectra for piperidine **18c**.

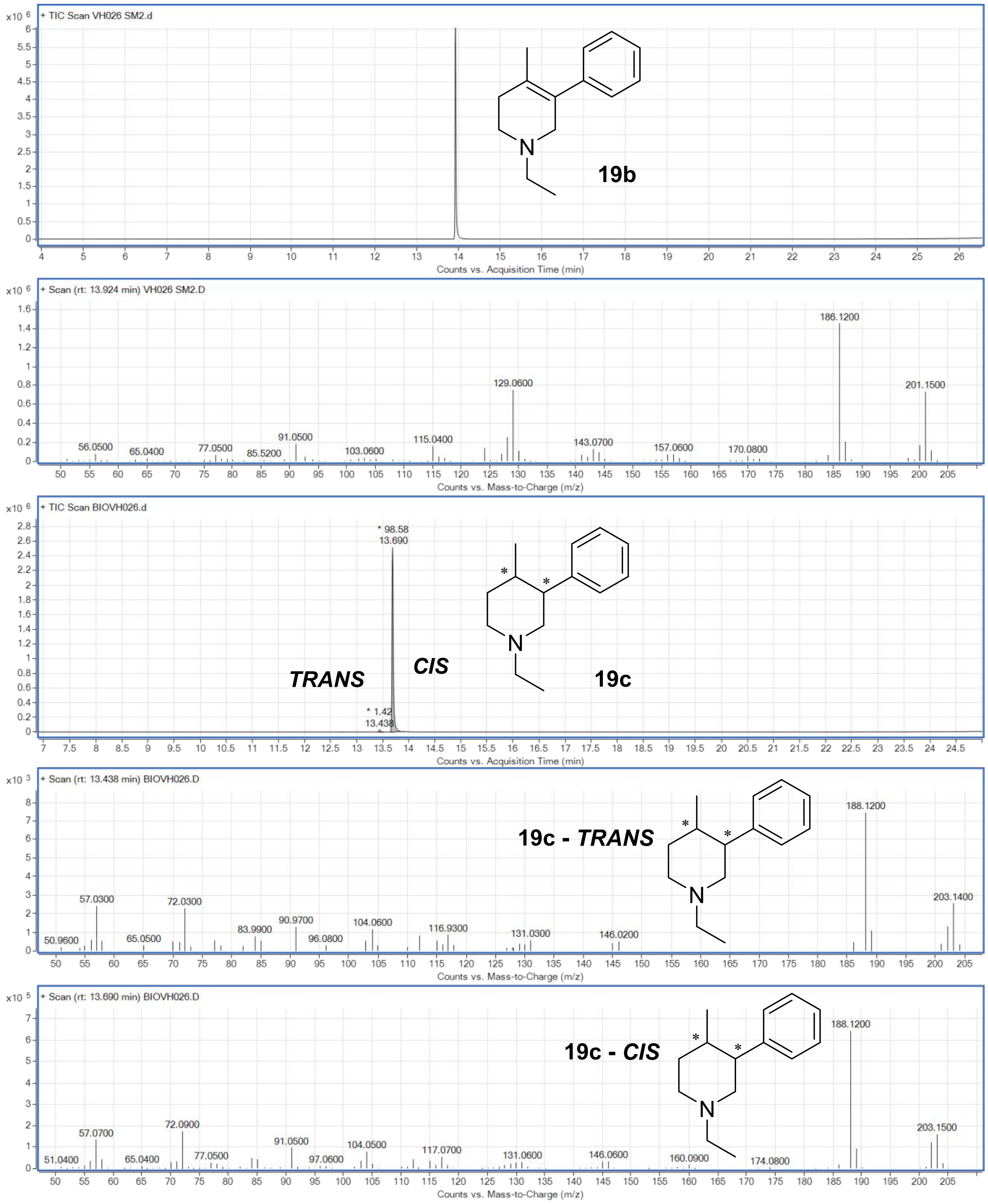

Figure S39: GC-MS spectra for piperidine **19c**.

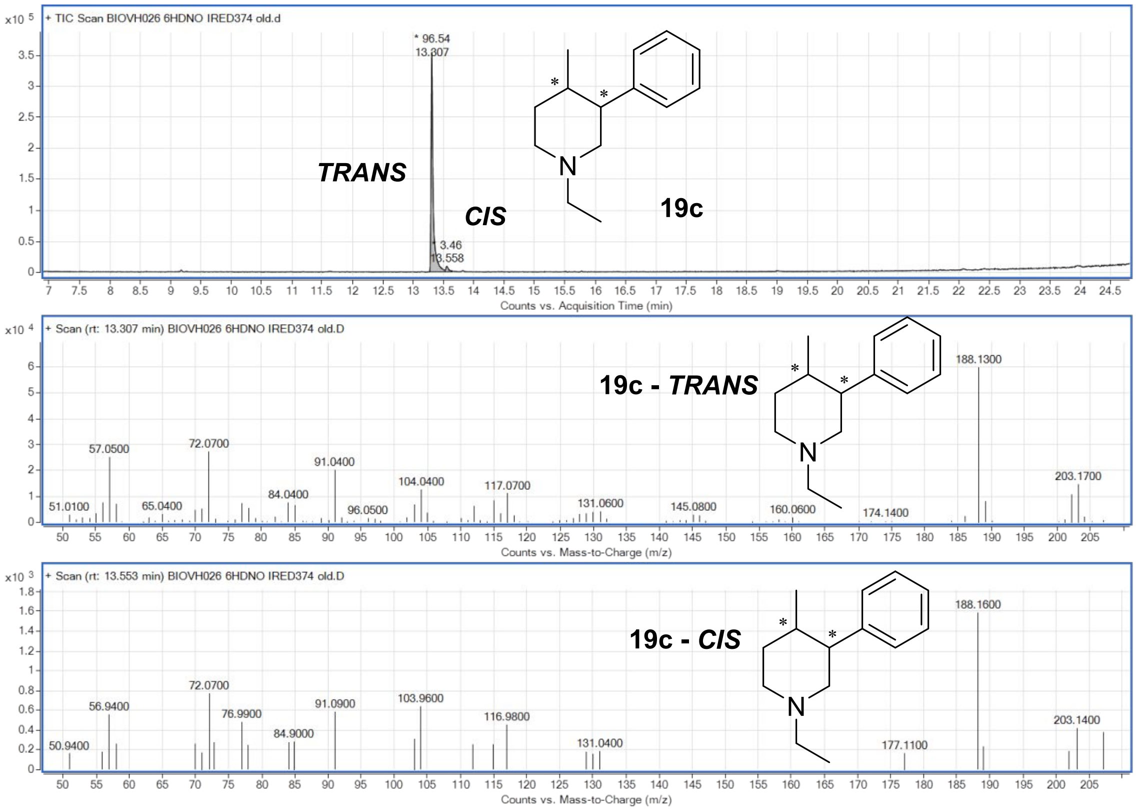

Figure S40: GC-MS spectra for piperidine **19c**.

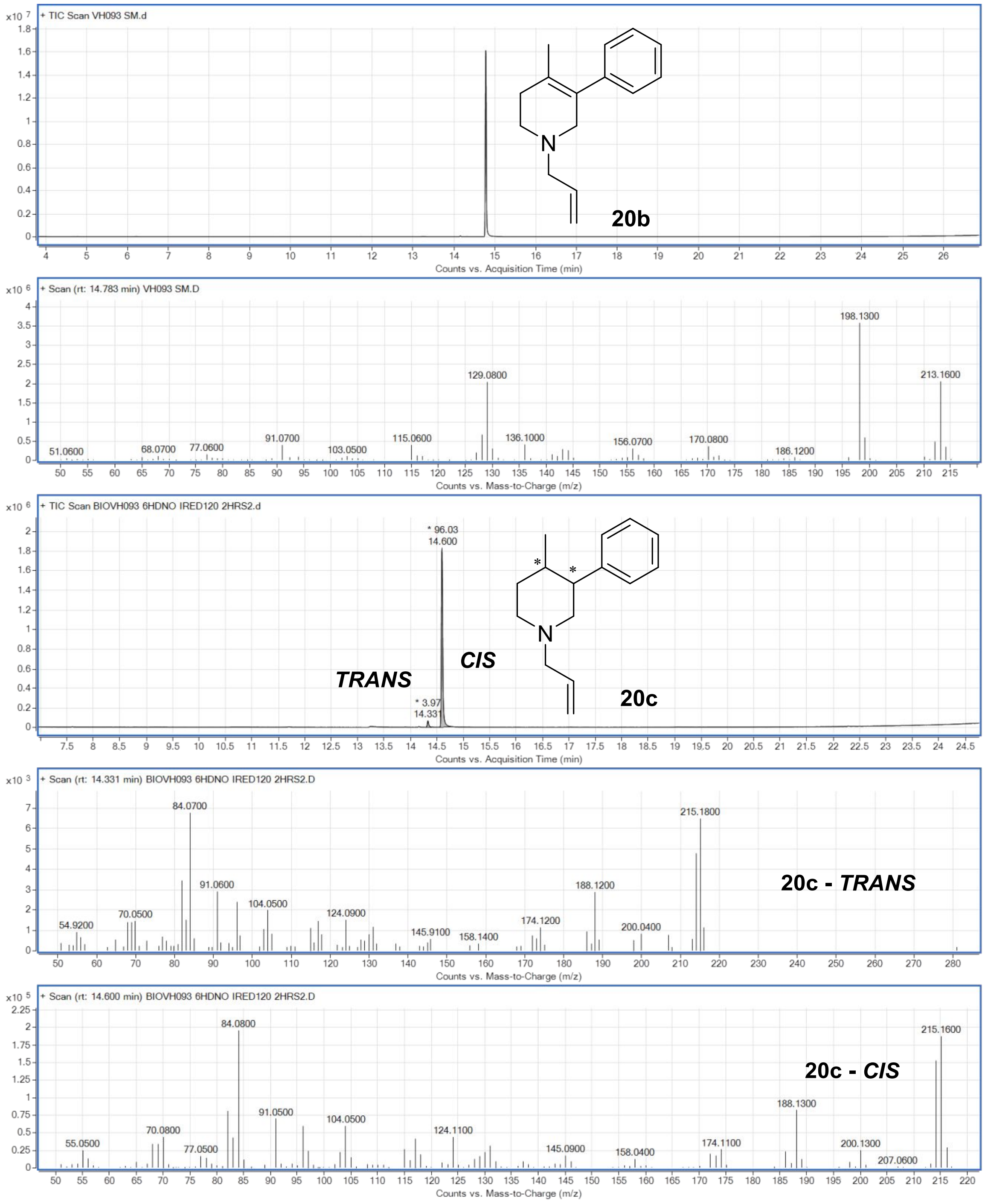

Figure S41: GC-MS spectra for piperidine **20c**.

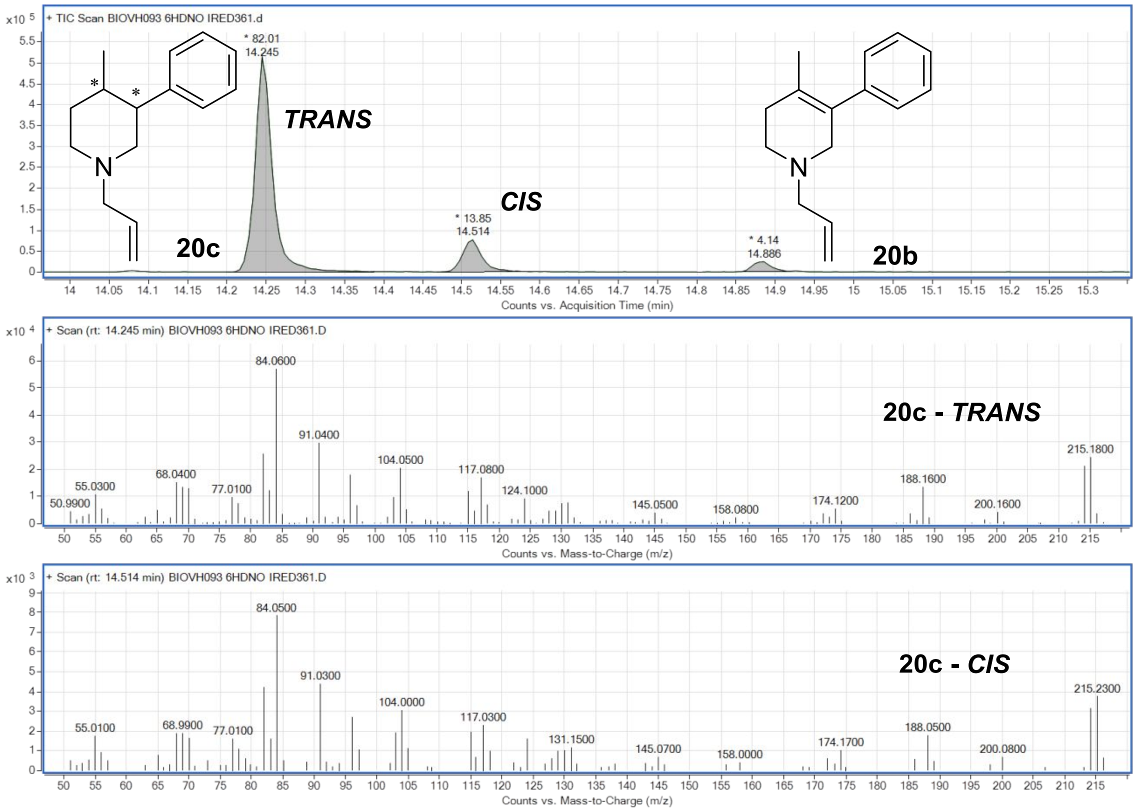

Figure S42: GC-MS spectra for piperidine **20c**.

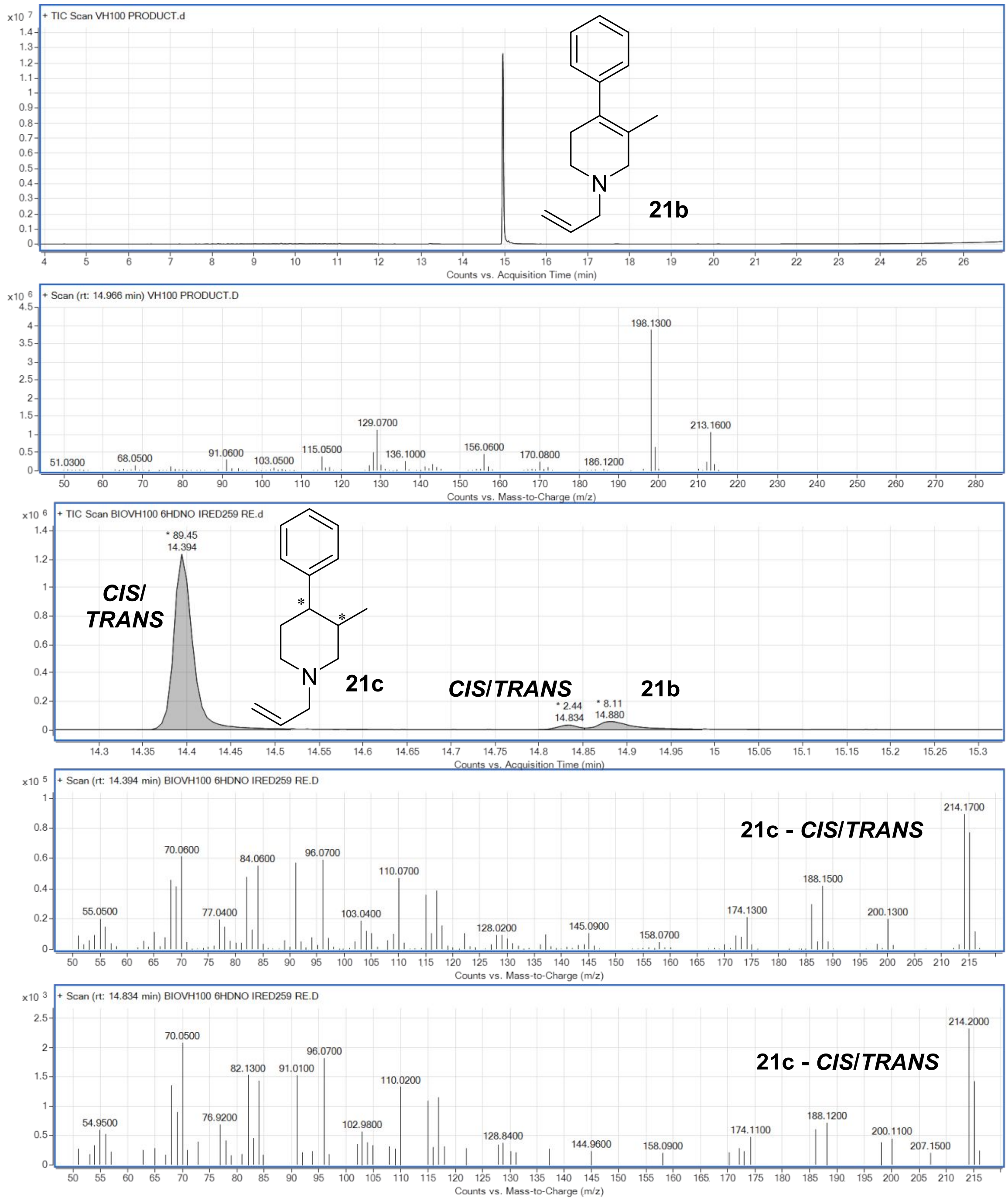

Figure S43: GC-MS spectra for piperidine **21c**.

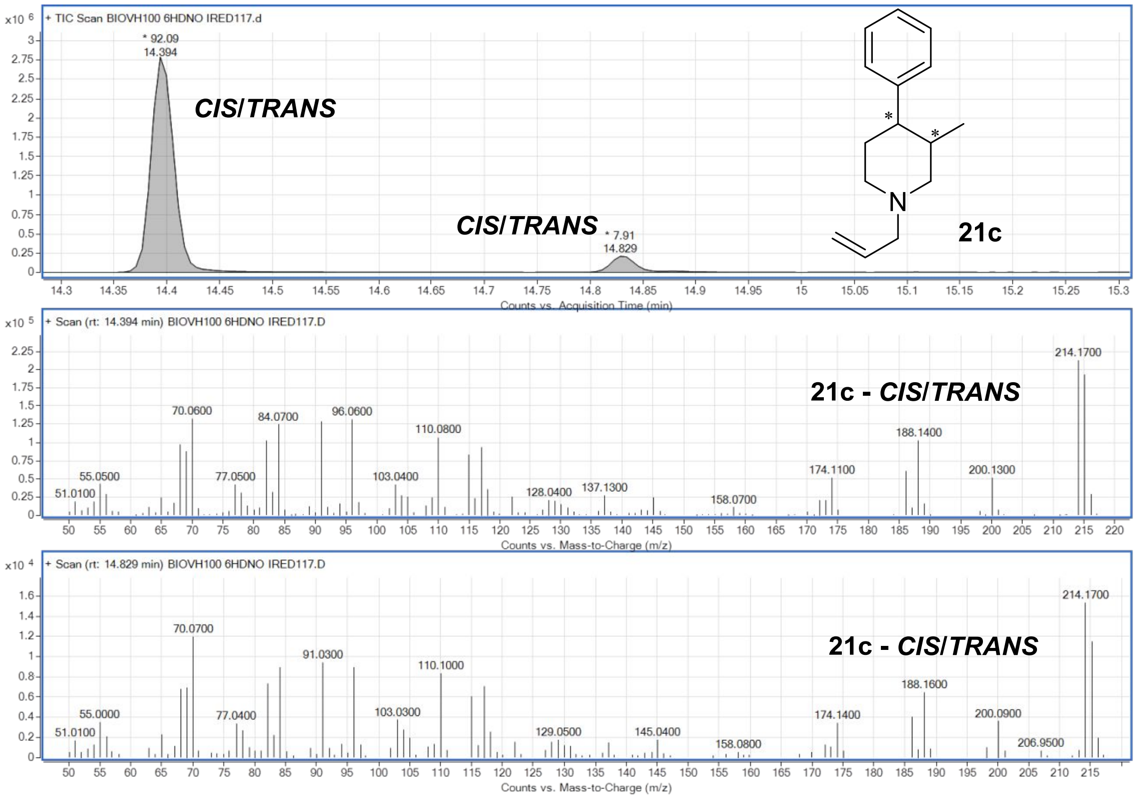

Figure S44: GC-MS spectra for piperidine **21c**.

9. Chiral HPLC Analysis: methods and retention times for piperidine products

Table S11: Chiral HPLC analysis: methods and retention times of piperidine from biotransformations.

Piperidine	Column	n-hexane/IPA/ diethylamine running solvent ratio	Piperidine retention time (min)	
			T[1]	T[2]
**1c**	CHIRALPAK®IE	99:1:0.1	9.7 (*S*)	11.3 (*R*)
**28**	CHIRALPAK®IC	98:2:0.1	19.2 (*S*)	27.0 (*R*)
**2c**	CHIRALPAK®AD-H	95:5:0.1	12.1 (*S*)	13.8 (*R*)
**3c** (after hydrogenation)	CHIRALPAK®IA	99.5:0.5:0.1	5.8 (*S*)	8.0 (*R*)
**4c** (after hydrogenation)	CHIRALPAK®AD-H	90:10:0.1	8.4 (*R*)	11.0 (*S*)
**6c**	CHIRALPAK®IE	99.5:0.5:0.1	7.5 (*S*)	8.1 (*R*)
**7c**	CHIRALPAK®IE	99:1:0.1	9.6 (*S*)	11.2 (*R*)
**9c**	CHIRALPAK®IE	99.5:0.5:0.1	7.3 (*R*)	7.8 (*S*)
**10c**	CHIRALPAK®AD-H	98:2:0.1	5.8 (*R*)	6.4 (*S*)
**12c** (after hydrogenation)	CHIRALPAK®IE	99.5:0.5:0.1	7.5 (*A*)	8.1 (*B*)

Absolute configurations were assigned by comparison with compounds of known configuration and VCD (see **section 4**).

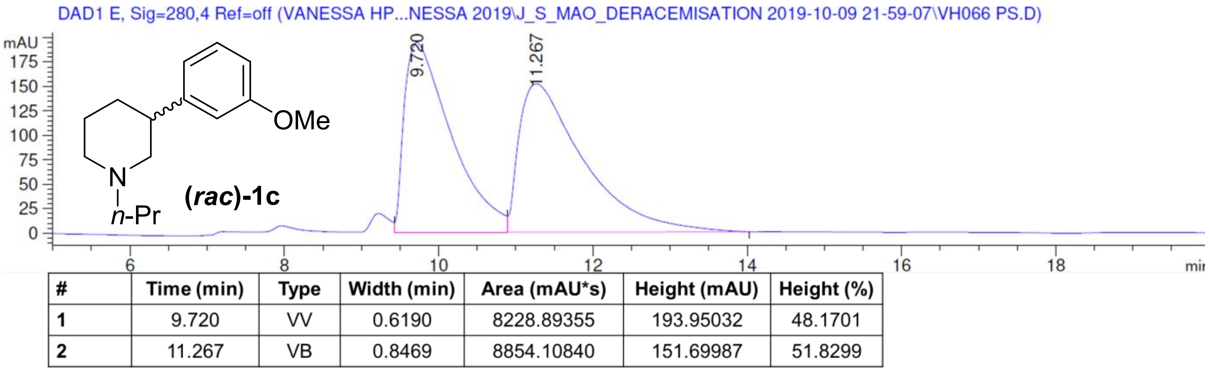

#	Time (min)	Type	Width (min)	Area (mAU*s)	Height (mAU)	Height (%)
1	9.720	VV	0.6190	8228.89355	193.95032	48.1701
2	11.267	VB	0.8469	8854.10840	151.69987	51.8299

With EneIRED-01

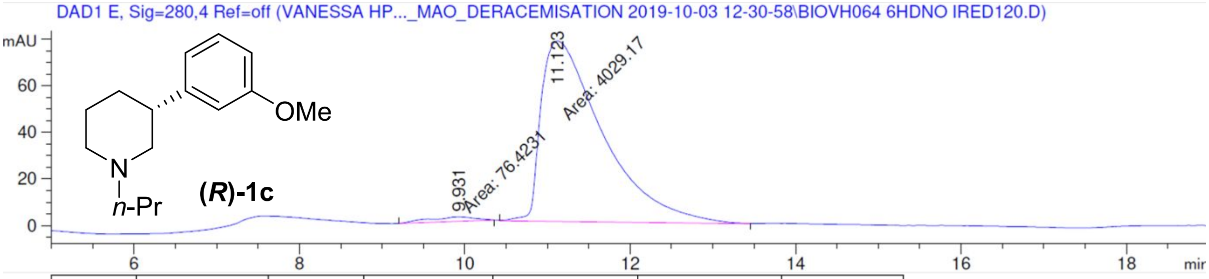

#	Time (min)	Type	Width (min)	Area (mAU*s)	Height (mAU)	Height (%)
1	9.931	MM	0.6687	76.42314	1.90478	1.8614
2	11.123	MM	0.8669	4029.17212	77.46283	98.1386

With EneIRED-05

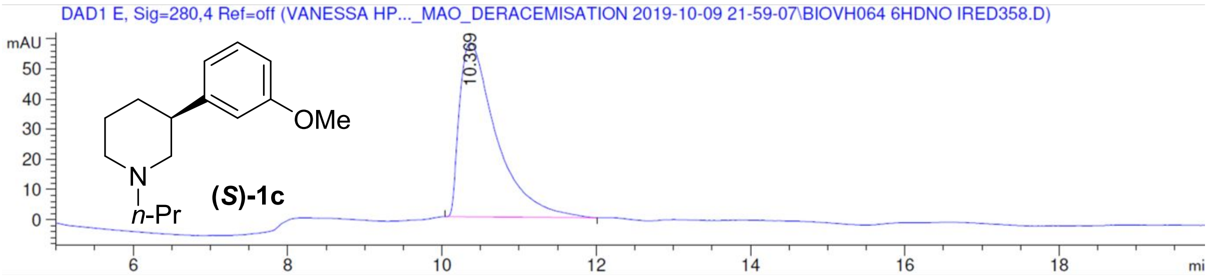

#	Time (min)	Type	Width (min)	Area (mAU*s)	Height (mAU)	Height (%)
1	10.369	BB	0.4932	1917.17712	57.76453	100.000

Figure S45: Chiral HPLC spectra for piperidine **1c**.

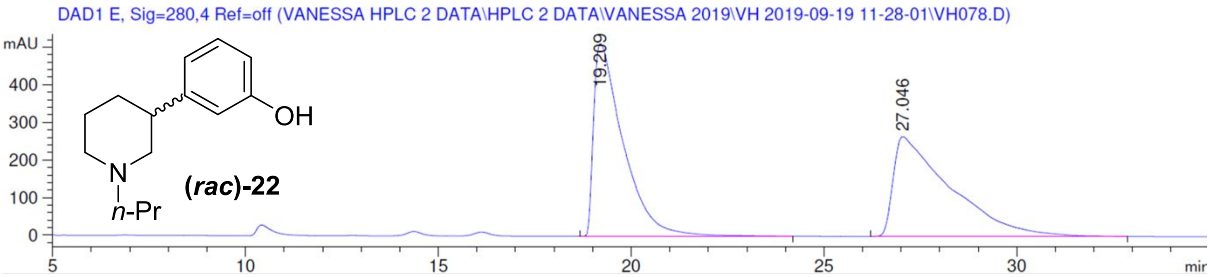

#	Time (min)	Type	Width (min)	Area (mAU*s)	Height (mAU)	Height (%)
1	19.209	BB	0.7734	27283.6	512.21387	50.0318
2	27.046	BB	1.3125	27249.0	265.24338	49.9682

With EneIRED-01

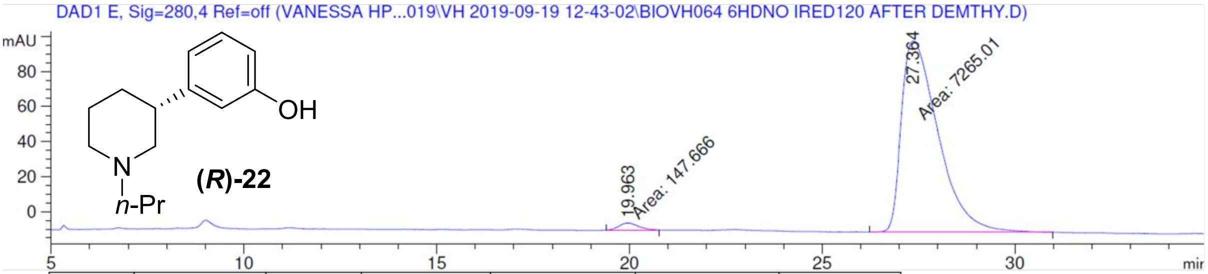

#	Time (min)	Type	Width (min)	Area (mAU*s)	Height (mAU)	Height (%)
1	19.963	MM	0.5928	147.66602	4.15166	1.9921
2	27.364	MM	1.1079	7265.01074	109.29315	98.0079

With EneIRED-05

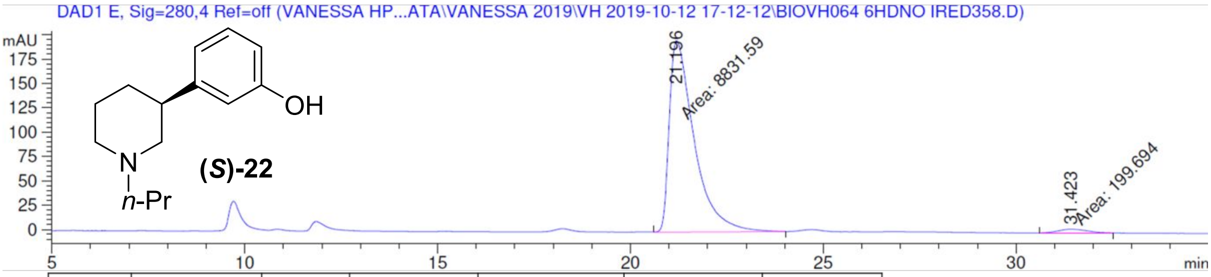

#	Time (min)	Type	Width (min)	Area (mAU*s)	Height (mAU)	Height (%)
1	21.196	MM	0.7488	8831.59082	196.57977	97.7889
2	31.423	MM	0.8651	199.69383	3.84723	2.2111

Figure S46: Chiral HPLC spectra for piperidine **22**.

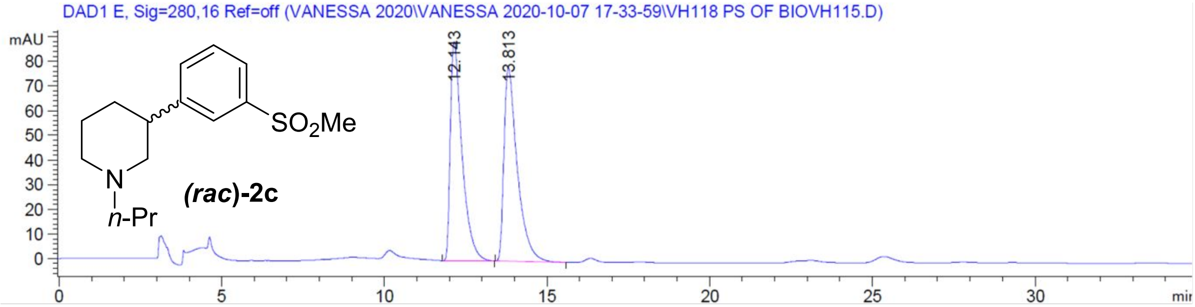

#	Time (min)	Type	Width (min)	Area (mAU*s)	Height (mAU)	Height (%)
**1**	12.143	BB	0.7288	4476.01464	182.34954	49.7474
**2**	13.813	BB	0.8258	4521.47314	161.38868	50.2526

With EneIRED-02

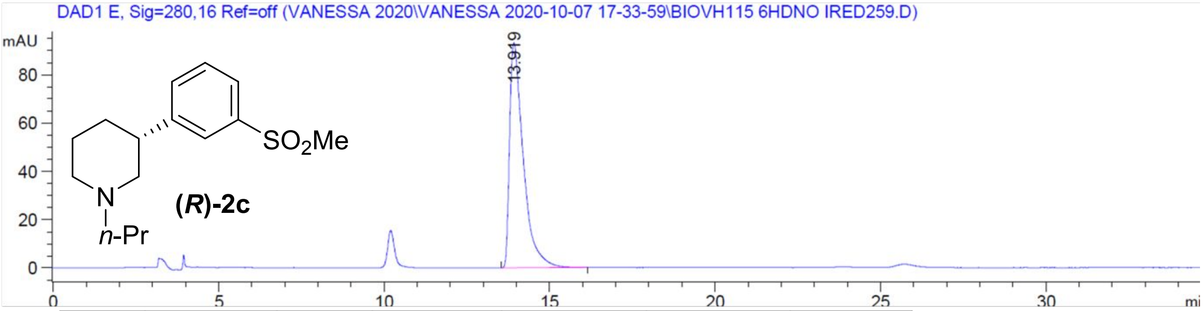

#	Time (min)	Type	Width (min)	Area (mAU*s)	Height (mAU)	Height (%)
**1**	13.919	BB	0.8456	5346.6084	186.29154	100.000

With EneIRED-06

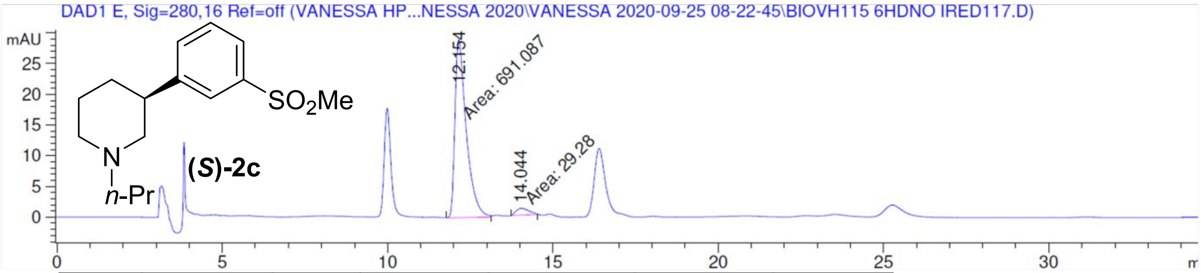

#	Time (min)	Type	Width (min)	Area (mAU*s)	Height (mAU)	Height (%)
**1**	12.154	MM	0.3974	691.08655	28.98159	95.9354
**2**	14.044	MM	0.4428	29.28002	1.10195	4.0646

Figure S47: Chiral HPLC spectra for piperidine **2c**.

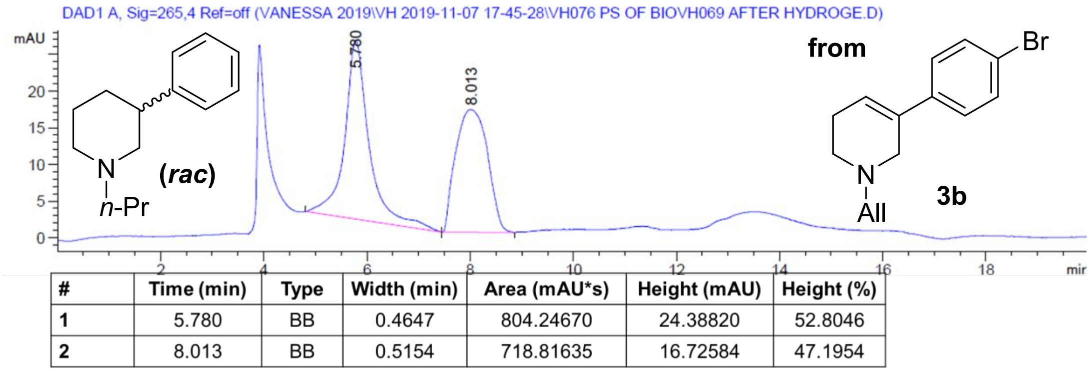

#	Time (min)	Type	Width (min)	Area (mAU*s)	Height (mAU)	Height (%)
1	5.780	BB	0.4647	804.24670	24.38820	52.8046
2	8.013	BB	0.5154	718.81635	16.72584	47.1954

With EneIRED-01

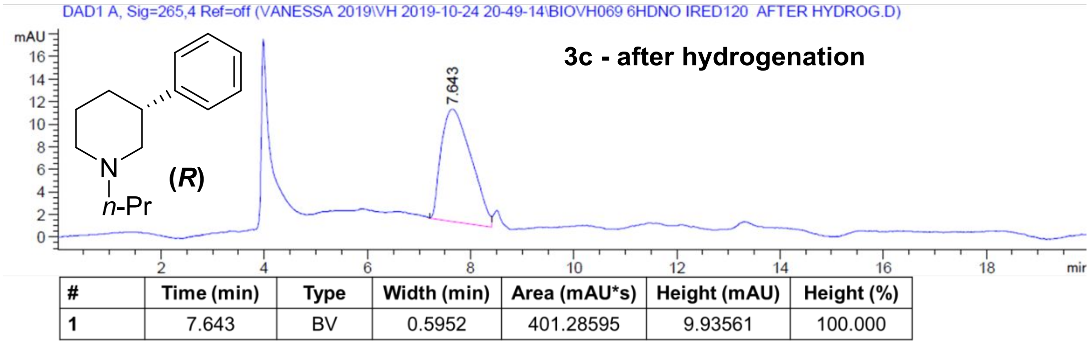

#	Time (min)	Type	Width (min)	Area (mAU*s)	Height (mAU)	Height (%)
1	7.643	BV	0.5952	401.28595	9.93561	100.000

With EneIRED-07

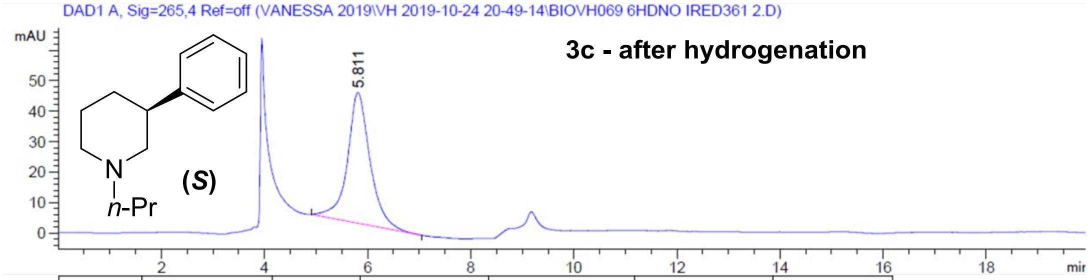

#	Time (min)	Type	Width (min)	Area (mAU*s)	Height (mAU)	Height (%)
1	5.811	BB	0.4742	1839.64380	55.00698	100.000

Figure S48: Chiral HPLC spectra for piperidine **3c** (after hydrogenation).

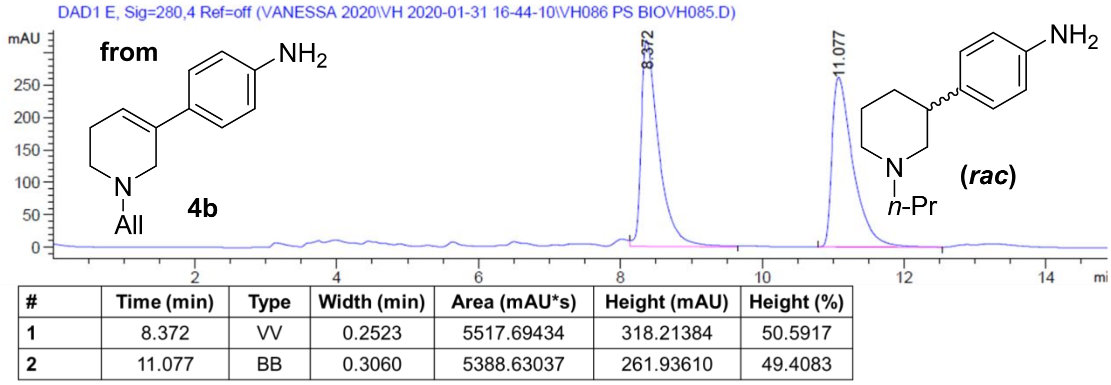

#	Time (min)	Type	Width (min)	Area (mAU*s)	Height (mAU)	Height (%)
1	8.372	VV	0.2523	5517.69434	318.21384	50.5917
2	11.077	BB	0.3060	5388.63037	261.93610	49.4083

With EneIRED-01

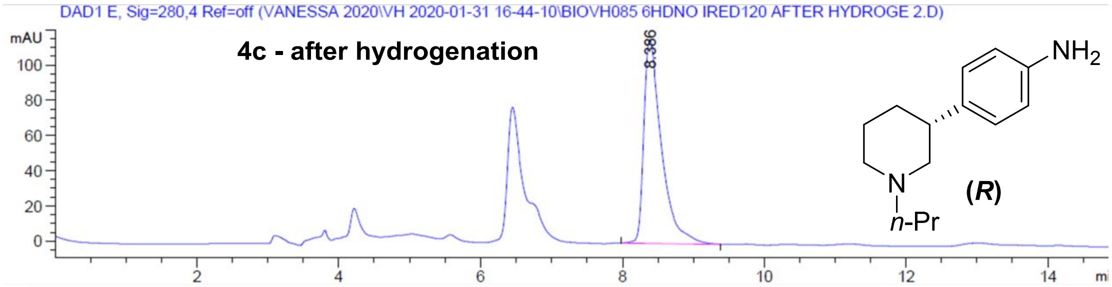

#	Time (min)	Type	Width (min)	Area (mAU*s)	Height (mAU)	Height (%)
1	8.386	BB	0.2559	1999.47095	115.53316	100.000

With EneIRED-07

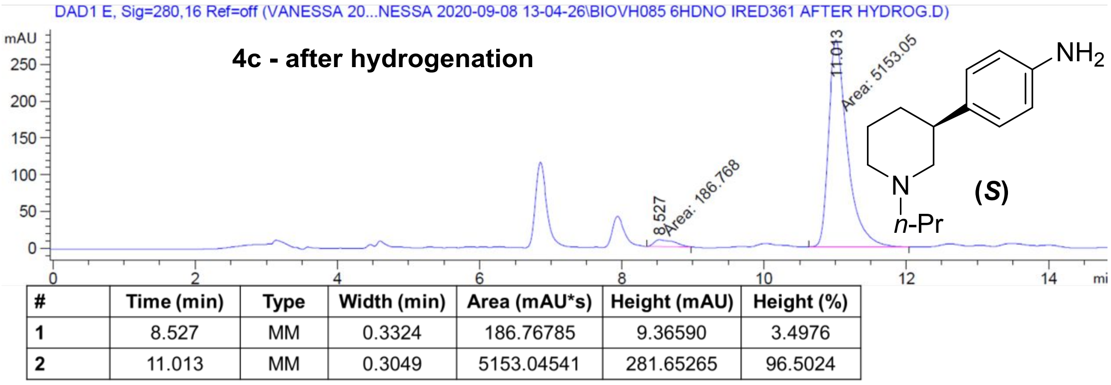

#	Time (min)	Type	Width (min)	Area (mAU*s)	Height (mAU)	Height (%)
1	8.527	MM	0.3324	186.76785	9.36590	3.4976
2	11.013	MM	0.3049	5153.04541	281.65265	96.5024

Figure S49: Chiral HPLC spectra for piperidine **4c** (after hydrogenation).

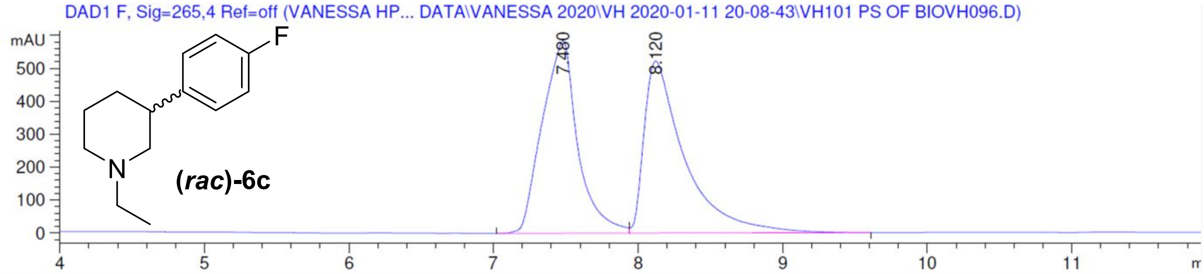

#	Time (min)	Type	Width (min)	Area (mAU*s)	Height (mAU)	Height (%)
1	7.480	BV	0.2790	9964.70801	584.19275	49.5003
2	8.120	VB	0.2793	10165.9	521.83789	50.4997

With EneIRED-01

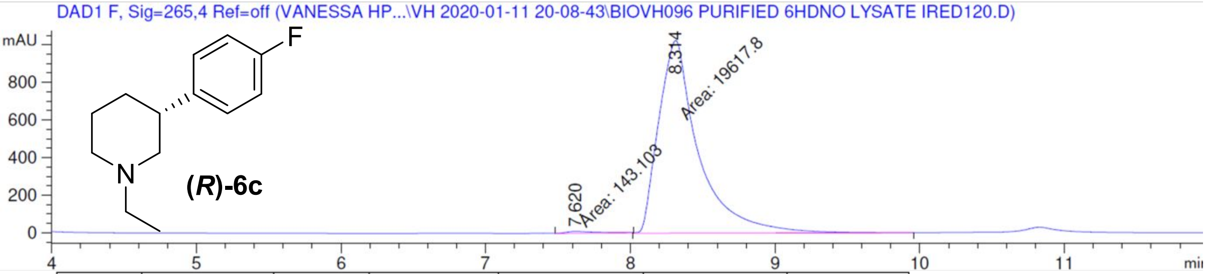

#	Time (min)	Type	Width (min)	Area (mAU*s)	Height (mAU)	Height (%)
1	7.620	MM	0.2373	143.10320	10.05132	0.7242
2	8.314	MM	0.3189	19617.8	1025.19812	99.2758

With EneIRED-07

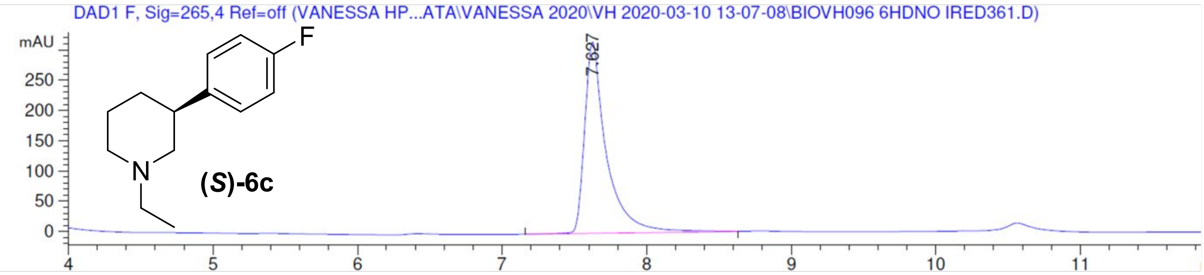

#	Time (min)	Type	Width (min)	Area (mAU*s)	Height (mAU)	Height (%)
1	7.627	BB	0.1501	3307.18018	316.12640	100.0000

Figure S50: Chiral HPLC spectra for piperidine **6c**.

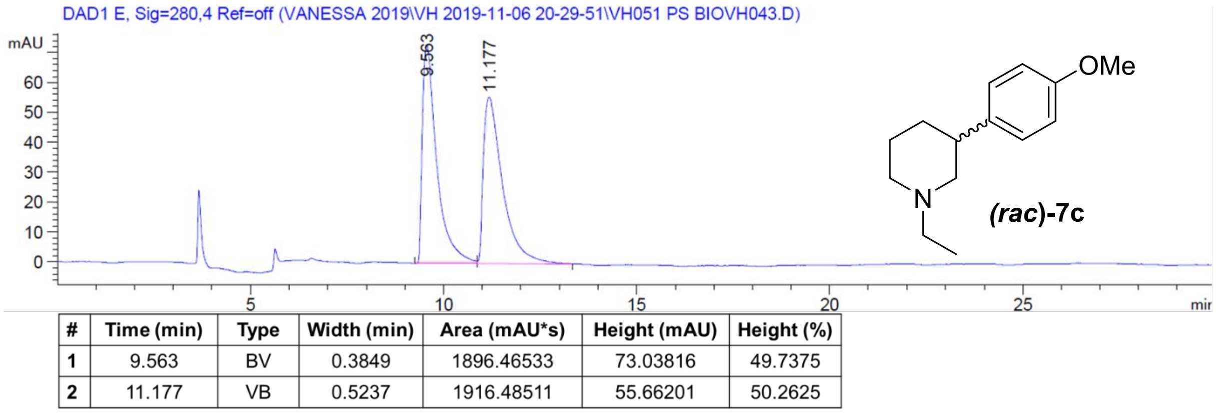

#	Time (min)	Type	Width (min)	Area (mAU*s)	Height (mAU)	Height (%)
1	9.563	BV	0.3849	1896.46533	73.03816	49.7375
2	11.177	VB	0.5237	1916.48511	55.66201	50.2625

With EneIRED-01

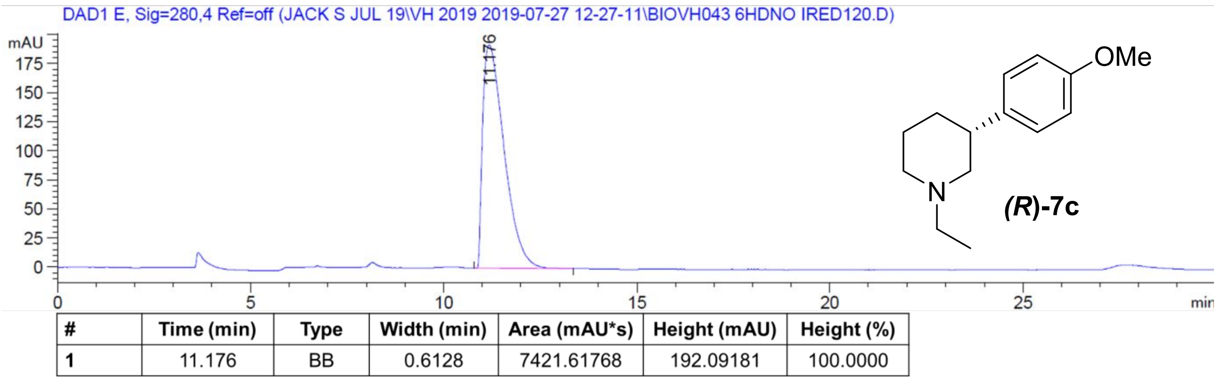

#	Time (min)	Type	Width (min)	Area (mAU*s)	Height (mAU)	Height (%)
1	11.176	BB	0.6128	7421.61768	192.09181	100.0000

With EneIRED-07

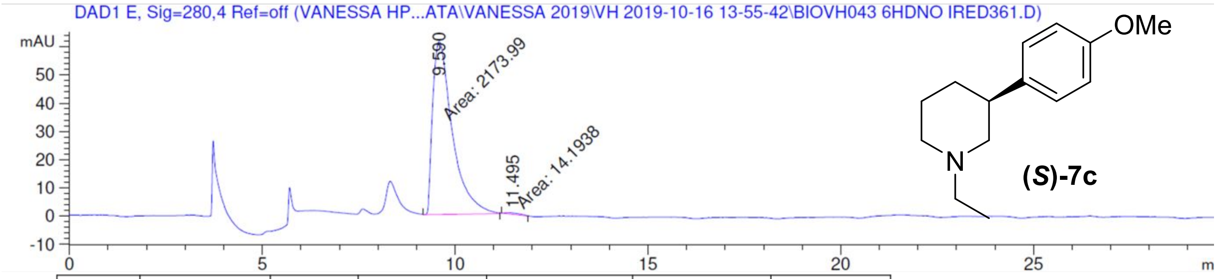

#	Time (min)	Type	Width (min)	Area (mAU*s)	Height (mAU)	Height (%)
1	9.590	MM	0.5916	2173.99194	61.24862	99.3513
2	11.495	MM	0.4559	14.19377	0.518934	0.6487

Figure S51: Chiral HPLC spectra for piperidine **7c**.

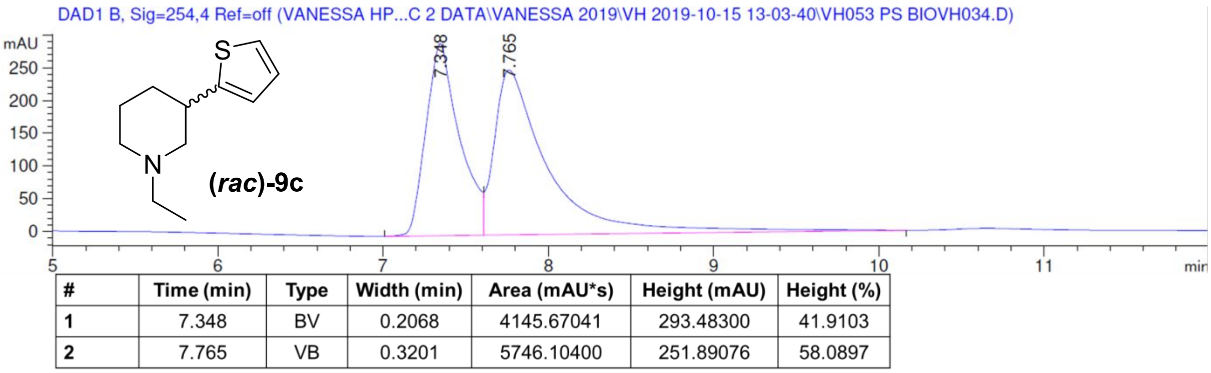

#	Time (min)	Type	Width (min)	Area (mAU*s)	Height (mAU)	Height (%)
1	7.348	BV	0.2068	4145.67041	293.48300	41.9103
2	7.765	VB	0.3201	5746.10400	251.89076	58.0897

With EneIRED-01

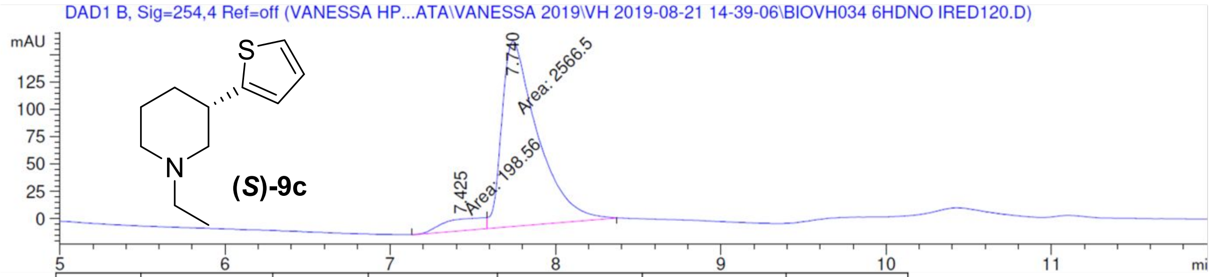

#	Time (min)	Type	Width (min)	Area (mAU*s)	Height (mAU)	Height (%)
1	7.425	MF	0.3090	198.56033	10.70852	7.1810
2	7.740	FM	0.2522	2566.50293	169.62709	92.8190

With EneIRED-06

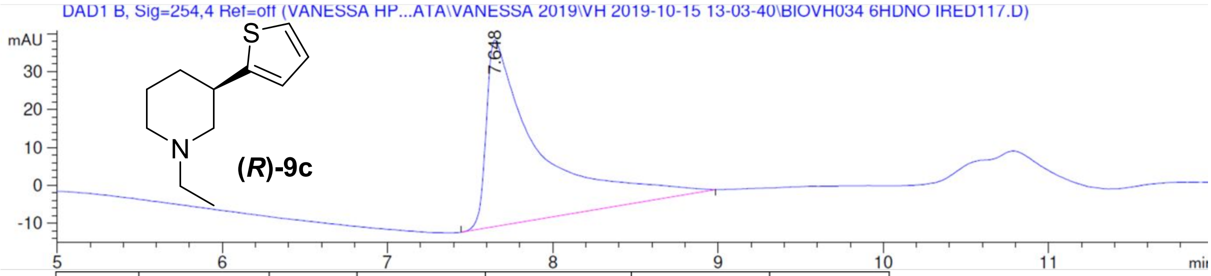

#	Time (min)	Type	Width (min)	Area (mAU*s)	Height (mAU)	Height (%)
1	7.648	BB	0.2934	1096.43250	49.30542	100.0000

Figure S52: Chiral HPLC spectra for piperidine **9c**.

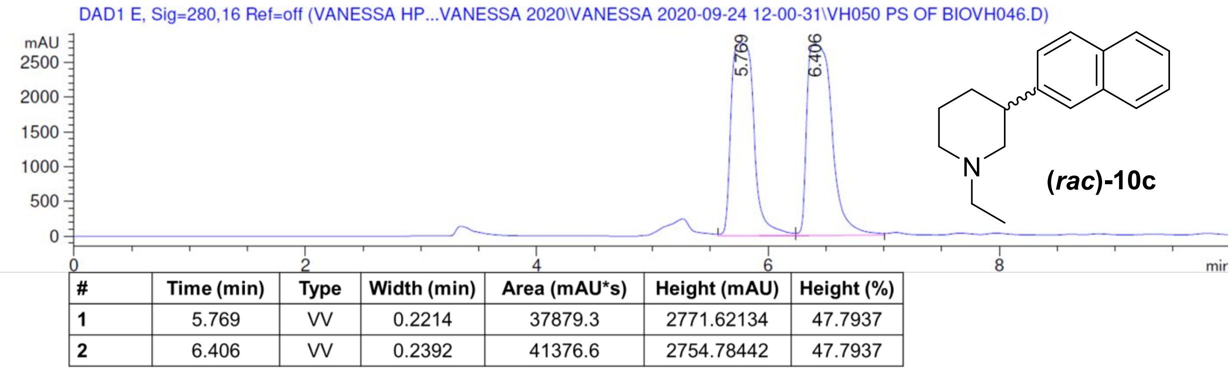

#	Time (min)	Type	Width (min)	Area (mAU*s)	Height (mAU)	Height (%)
1	5.769	VV	0.2214	37879.3	2771.62134	47.7937
2	6.406	VV	0.2392	41376.6	2754.78442	47.7937

With EneIRED-01

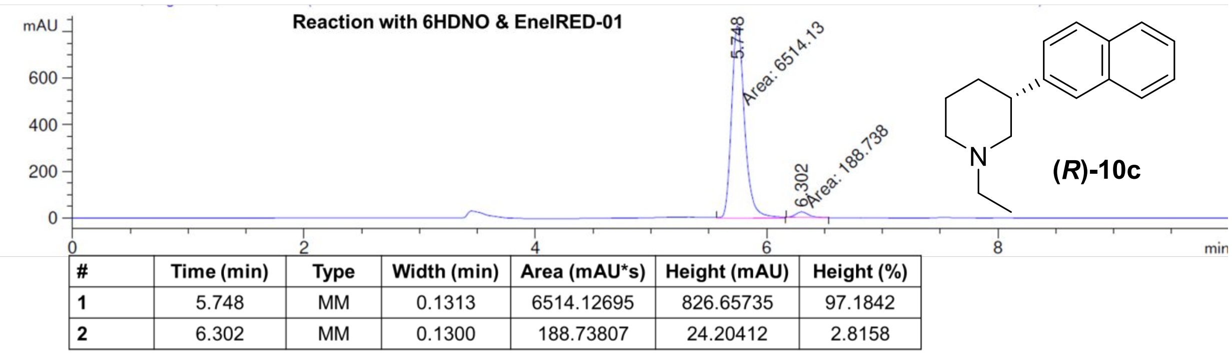

#	Time (min)	Type	Width (min)	Area (mAU*s)	Height (mAU)	Height (%)
1	5.748	MM	0.1313	6514.12695	826.65735	97.1842
2	6.302	MM	0.1300	188.73807	24.20412	2.8158

With EneIRED-01

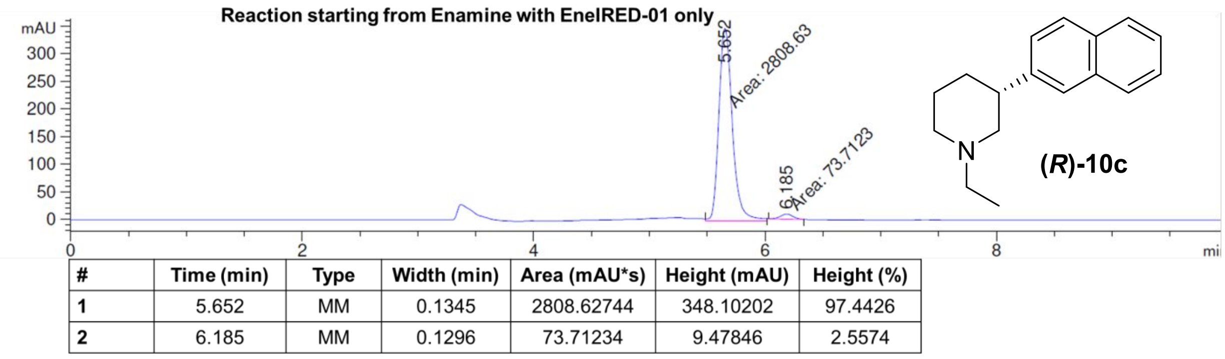

#	Time (min)	Type	Width (min)	Area (mAU*s)	Height (mAU)	Height (%)
1	5.652	MM	0.1345	2808.62744	348.10202	97.4426
2	6.185	MM	0.1296	73.71234	9.47846	2.5574

With EneIRED-07

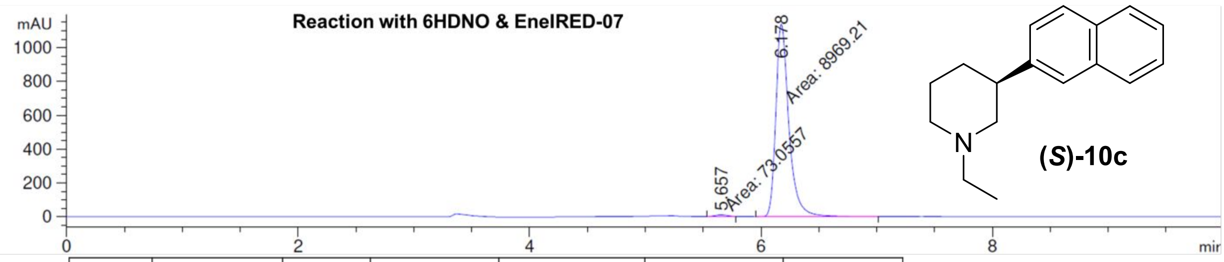

#	Time (min)	Type	Width (min)	Area (mAU*s)	Height (mAU)	Height (%)
1	5.657	MM	0.1268	73.05570	9.60555	0.8079
2	6.178	MM	0.1310	8969.21094	1140.84924	99.1921

Figure S53: Chiral HPLC spectra for piperidine **10c**.

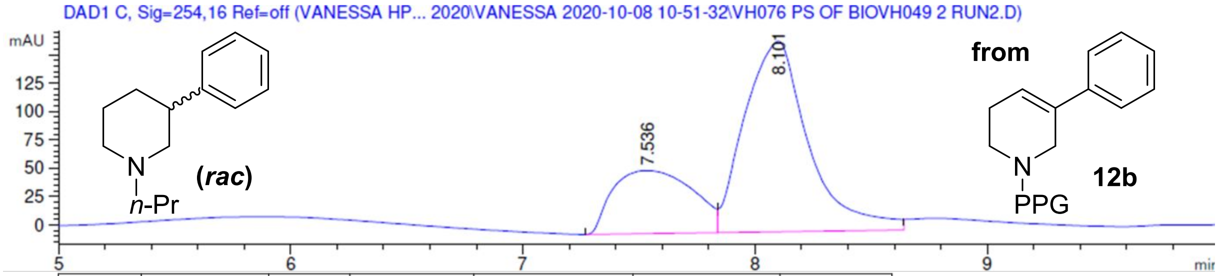

#	Time (min)	Type	Width (min)	Area (mAU*s)	Height (mAU)	Height (%)
1	7.536	BV	0.4154	1331.28906	55.58586	29.2320
2	8.101	VV	0.3038	3222.92456	168.00810	70.7680

With EneIRED-01

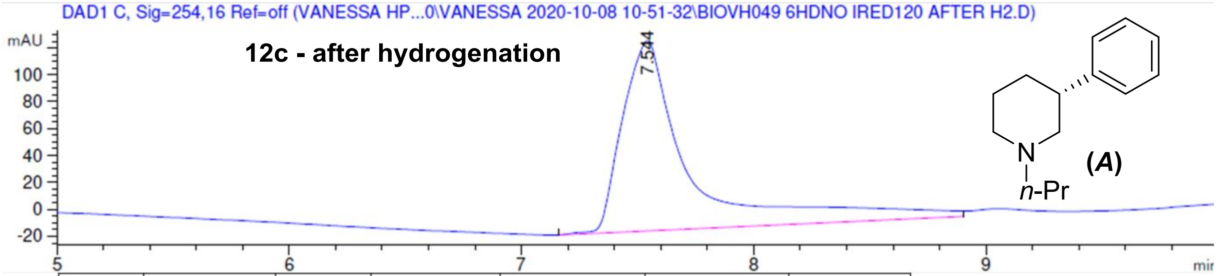

#	Time (min)	Type	Width (min)	Area (mAU*s)	Height (mAU)	Height (%)
1	7.544	BV	0.2954	2849.63379	141.21489	100.000

Figure S54: Chiral HPLC spectra for piperidine **12c**.

10. Chiral GC Analysis: methods and retention times for piperidine products

Table S12: Chiral GC analysis: methods and retention times of piperidine products from biotransformations.

Piperidine product	Column	Oven temp.	Piperidine products retention time (min)			
			T1		T2	
**8c**	Supelco BDEX-325	50 – 200 °C, 2 °C min^{-1}	41.1 (*S*)		41.3 (*R*)	
**17c**	Supelco BDEX-325	50 – 200 °C, 2 °C min^{-1}	17.0	17.2	17.9	18.4
**18c**	Supelco BDEX-325	50 – 200 °C, 2 °C min^{-1}	36.2	36.4	37.3	37.8

Injector temperature: 200 °C, detector temperature: 250 °C, helium flow: 1.2 mL/min.

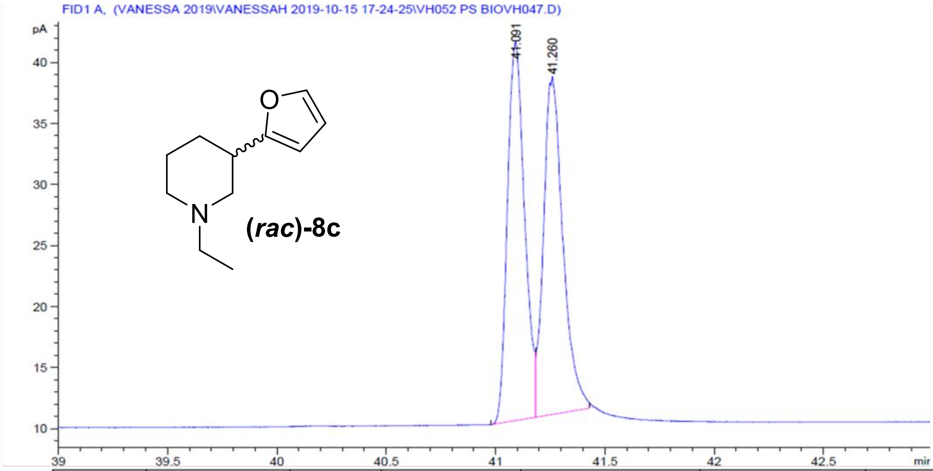

#	Time (min)	Type	Width (min)	Area (pA*s)	Height (pA)	Height (%)
1	41.091	MF	0.0914	171.42841	31.27003	49.41671
2	41.260	FM	0.1048	175.47528	27.90684	50.58329

With EneIRED-01

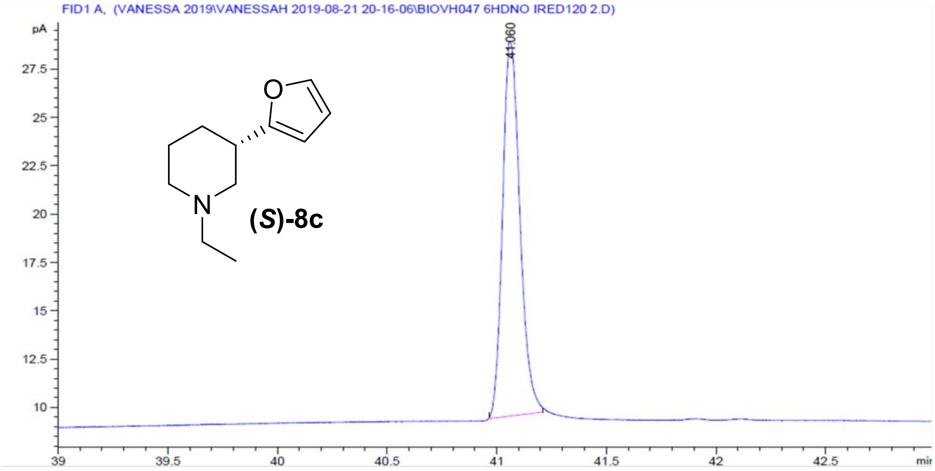

#	Time (min)	Type	Width (min)	Area (pA*s)	Height (pA)	Height (%)
1	41.060	BB	0.0793	106.56185	19.33856	100.0

With EneIRED-06

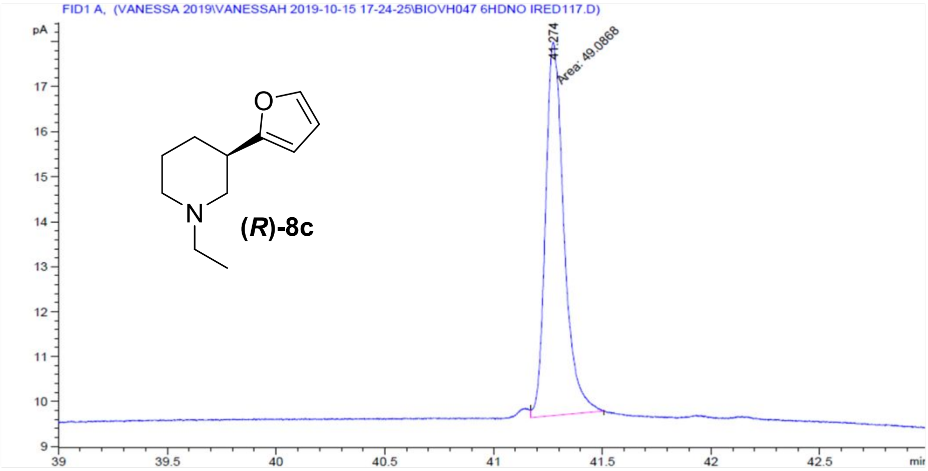

#	Time (min)	Type	Width (min)	Area (pA*s)	Height (pA)	Height (%)
1	41.145	BV	0.0365	0.535183	0.175764	1.10415
2	41.275	VB	0.0703	47.93508	8.24269	98.89585

Figure S55: Chiral GC spectra for piperidine **8c**.

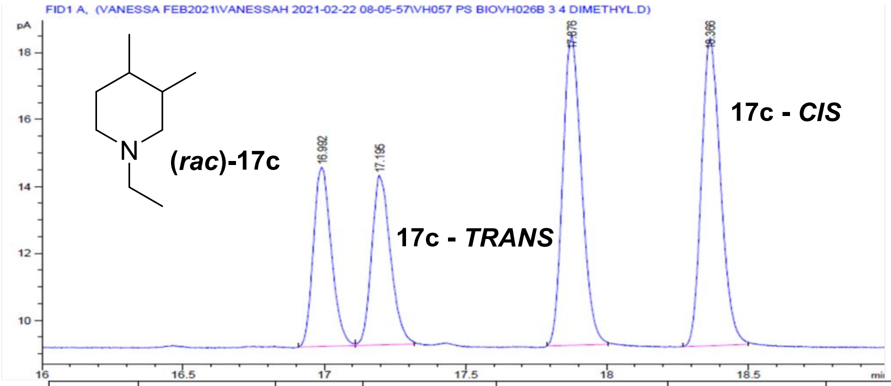

#	Time (min)	Type	Width (min)	Area (pA*s)	Height (pA)	Height (%)
1	16.992	BB	0.0619	11.39545	5.34035	17.92401
2	17.195	BB	0.0711	10.59307	5.06616	16.66194
3	17.876	BB	0.0952	21.06744	9.24981	33.13716
4	18.366	BB	0.0995	20.52052	9.16730	32.27690

With EneIRED-01

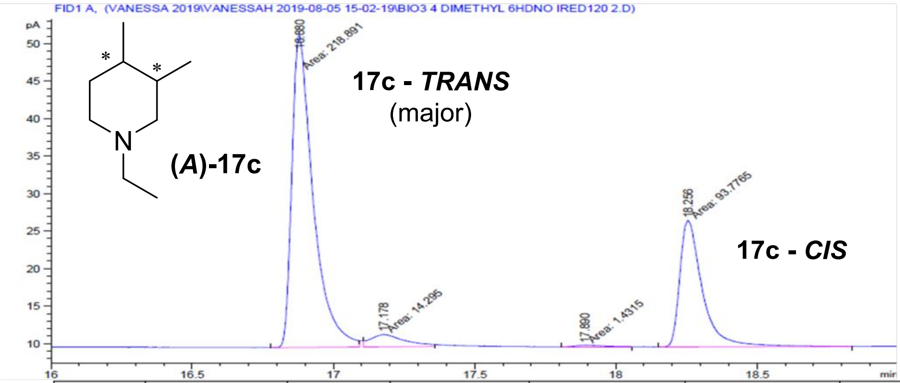

#	Time (min)	Type	Width (min)	Area (pA*s)	Height (pA)	Height (%)
1	16.880	VV	0.0655	218.41089	41.62736	66.88537
2	17.178	VB	0.0956	12.92578	1.59369	3.95834
3	17.896	MM	0.1064	1.43162	0.224337	0.43842
4	18.255	MM	0.0927	93.77681	16.85694	28.71787

With EneIRED-06

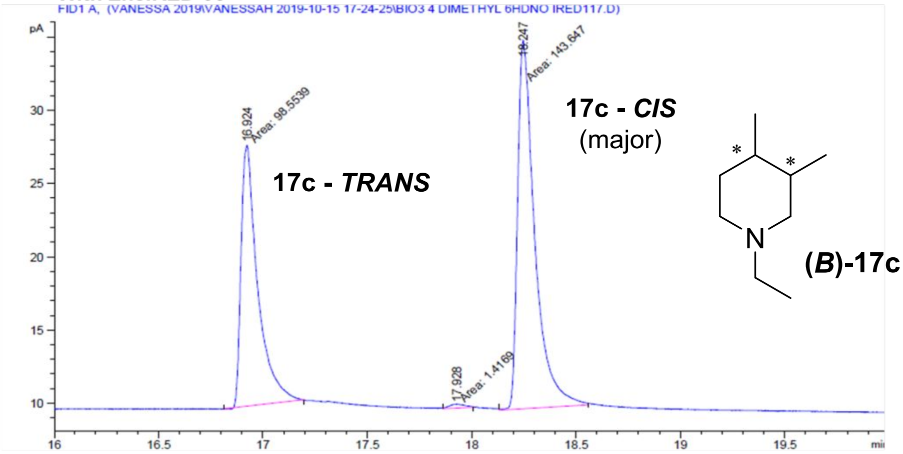

#	Time (min)	Type	Width (min)	Area (pA*s)	Height (pA)	Height (%)
1	16.924	MM	0.0922	98.55395	17.82218	40.45426
2	17.928	MM	0.0911	1.41690	0.259310	0.58161
3	18.247	MM	0.0954	143.64737	25.10015	58.96413

Figure S56: Chiral GC spectra for piperidine **17c**.

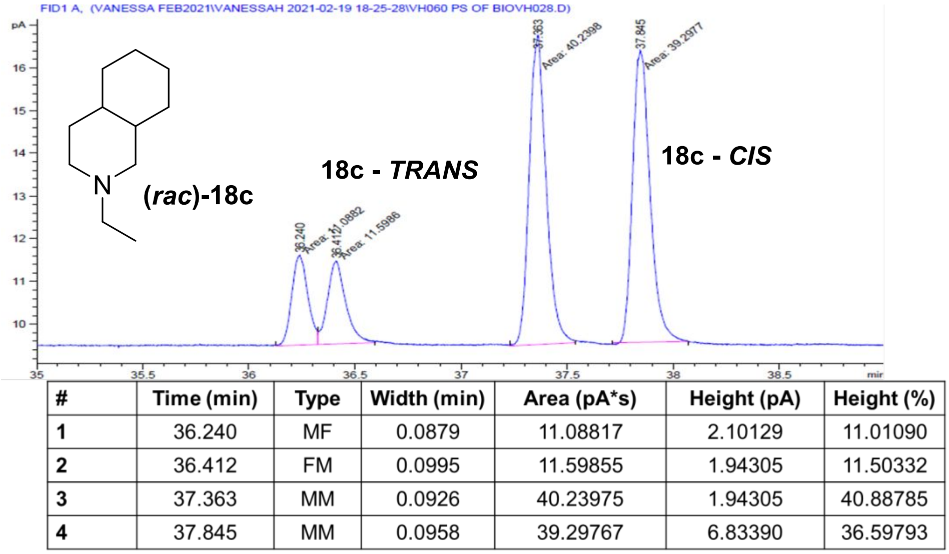

#	Time (min)	Type	Width (min)	Area (pA*s)	Height (pA)	Height (%)
1	36.240	MF	0.0879	11.08817	2.10129	11.01090
2	36.412	FM	0.0995	11.59855	1.94305	11.50332
3	37.363	MM	0.0926	40.23975	1.94305	40.88785
4	37.845	MM	0.0958	39.29767	6.83390	36.59793

With EneIRED-01

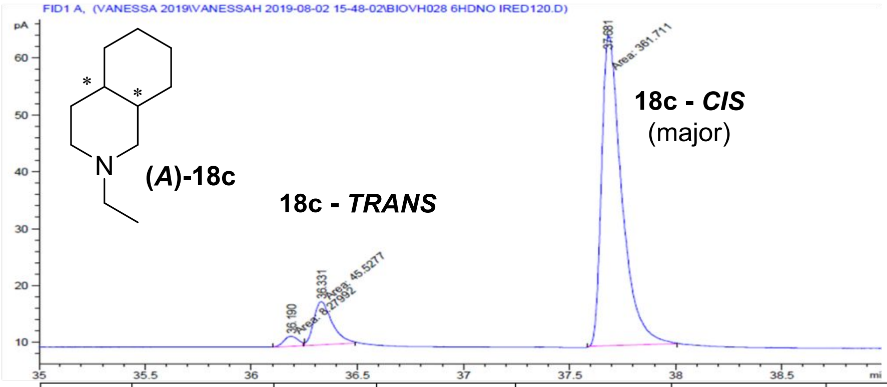

#	Time (min)	Type	Width (min)	Area (pA*s)	Height (pA)	Height (%)
1	36.190	MF	0.0785	8.27992	1.75811	1.99267
2	36.331	FM	0.1004	45.52773	7.56114	10.95685
3	37.681	MM	0.1104	361.71078	54.59454	87.05048

With EneIRED-08

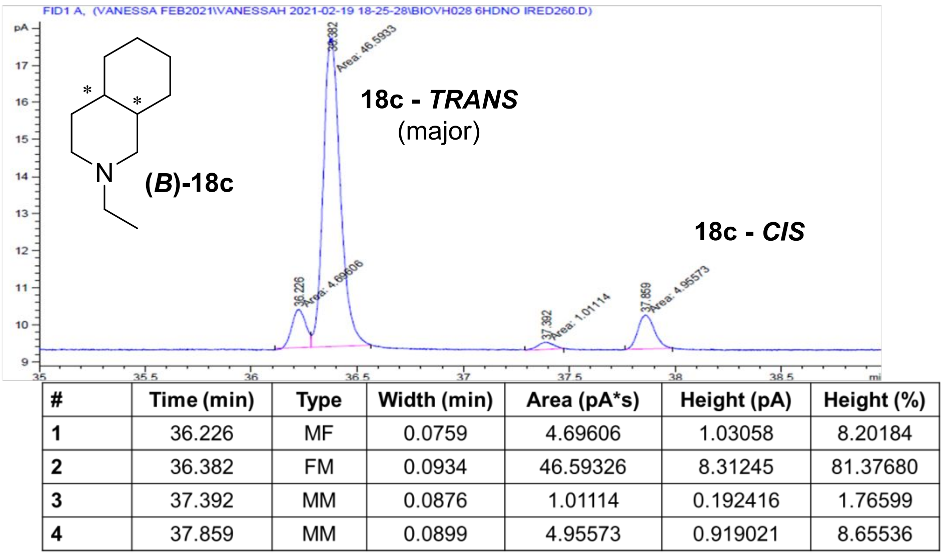

#	Time (min)	Type	Width (min)	Area (pA*s)	Height (pA)	Height (%)
1	36.226	MF	0.0759	4.69606	1.03058	8.20184
2	36.382	FM	0.0934	46.59326	8.31245	81.37680
3	37.392	MM	0.0876	1.01114	0.192416	1.76599
4	37.859	MM	0.0899	4.95573	0.919021	8.65536

Figure S57: Chiral GC spectra for piperidine **18c**.

11. Chiral SFC Analysis: methods and retention times for piperidine products

Table S13: Chiral SFC analysis: methods and retention times of piperidine from biotransformations.

Piperidine	Column	Mobile phase	Piperidine retention time (min)	
			T1	T2
**5c**	CHIRALPAK®IG	20% MeOH (0.1% TEA)/80% CO_2	0.7 (*S*)	1.1 (*R*)
**11c**	CHIRALPAK®IG	5% MeOH (0.1% TEA)/95% CO_2	3.1 (*R*)	3.9 (*S*)

Absolute configurations were assigned by comparison with traces of commercial optically pure product derivatives (see **section 4**).

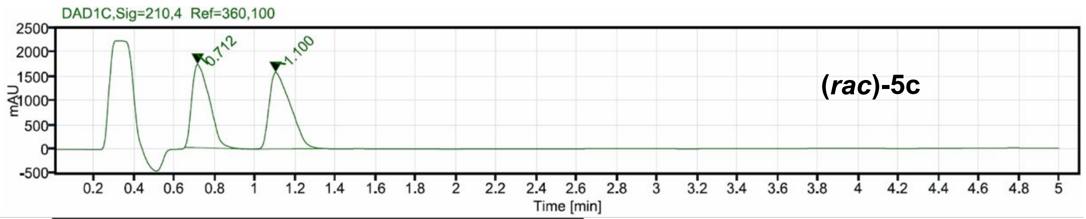

#	Time (min)	Area	Area (%)
1	0.712	10459.275	47.45
2	1.100	11581.830	52.55

With EneIRED-01

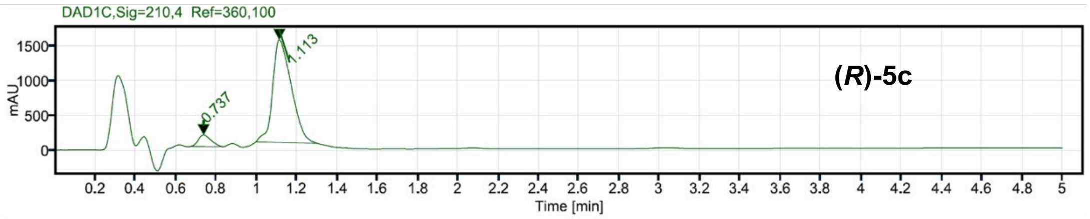

#	Time (min)	Area	Area (%)
1	0.737	728.123	7.07
2	1.113	9564.300	92.93

With EneIRED-07

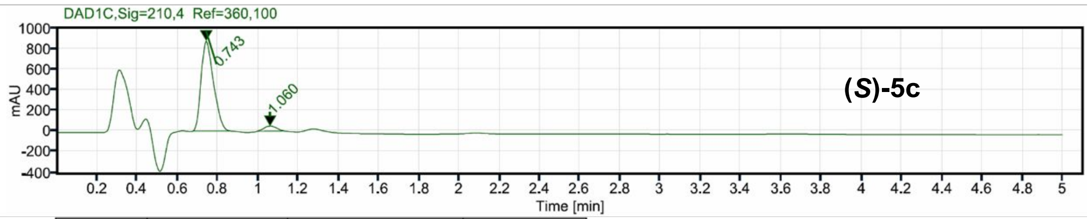

#	Time (min)	Area	Area (%)
1	0.743	3781.479	94.57
2	1.060	217.103	5.43

Figure S58: Chiral SFC spectra for piperidine **5c**.

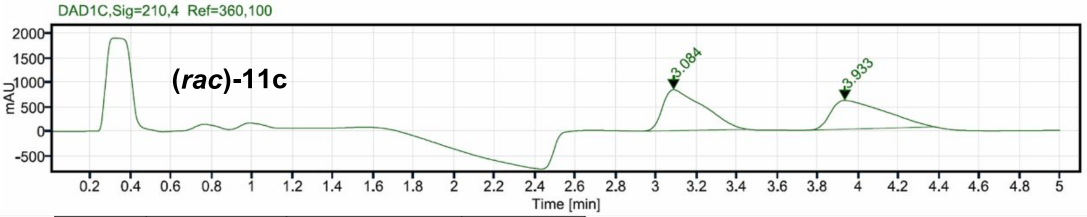

#	Time (min)	Area	Area (%)
1	3.084	1869.046	50.52
2	3.933	1830.440	49.48

With EneIRED-01

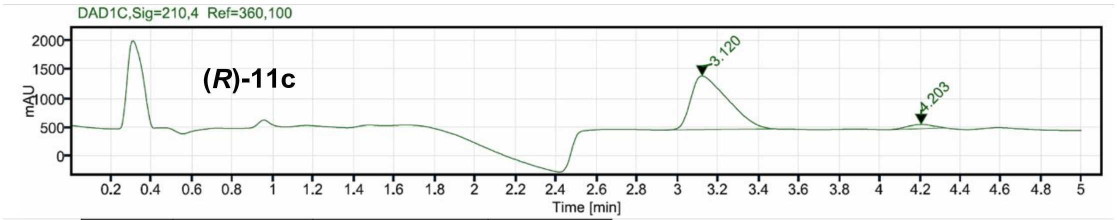

#	Time (min)	Area	Area (%)
1	3.120	11627.768	94.96
2	4.203	616.789	5.04

With EneIRED-07

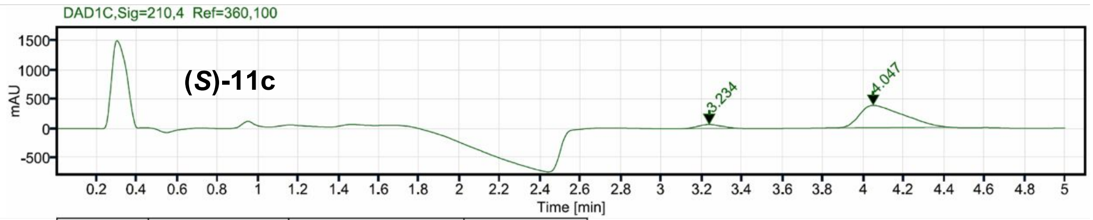

#	Time (min)	Area	Area (%)
1	3.234	515.131	8.30
2	4.047	5688.168	91.70

Figure S59: Chiral SFC spectra for piperidine **11c**.

12. VCD Analysis: methods for piperidine products

VCD Measurements: The Infrared (IR) and Vibrational Circular Dichroism (VCD) spectra were recorded using a ChiralIR™ VCD spectrometer equipped with the Dual PEM accessory (BioTools, Jupiter, Florida), with 4 cm^{-1} resolution. A 100 – 200 μL solution containing 6 - 15mg of chiral molecule dissolved in $CDCl_3$ was transferred to a BaF_2 IR cell with path length of 100 μm. The samples were measured for 8 blocks of 1 hour each while purged with dry air to remove water vapor. The IR was processed by solvent subtraction and offset to zero at 2000 cm^{-1}. The VCD blocks were averaged, then baseline corrected using either solvent or opposite enantiomer (if available) measured at the same concentration (then divided by 2) to produce the final spectrum.

VCD Calculations: Each chiral molecule (*R* configuration) was subjected to a conformer search (GMMX, MMF94) search using BioTools ComputeVOA software to find the lowest energy conformers in an 8 kcal/mol range. All conformers were minimized using Gaussian 09 at the 6-31G(d) / B3LYP and 6-31G(d) / B3PW91 level with CPCM solvent (chloroform) model. IR and VCD frequencies were calculated at the same level, then duplicates were removed. The lowest energy unique conformers (3 kcal / mol range) were then re-calculated at the cc-pVTZ / B3LYP and cc-pVTZ / B3PW91 level, and the resulting spectra from all four methods were Boltzmann averaged and plotted with a line width of 5 cm^{-1}. IR and VCD spectra were then frequency scaled and compared to the experimental data. In cases where the *S* enantiomer was the only available experimental compound, calculated spectra (*R* enantiomer) were multiplied by -1 to produce *S* theoretical spectra for comparison.

Analysis of piperidine 6c

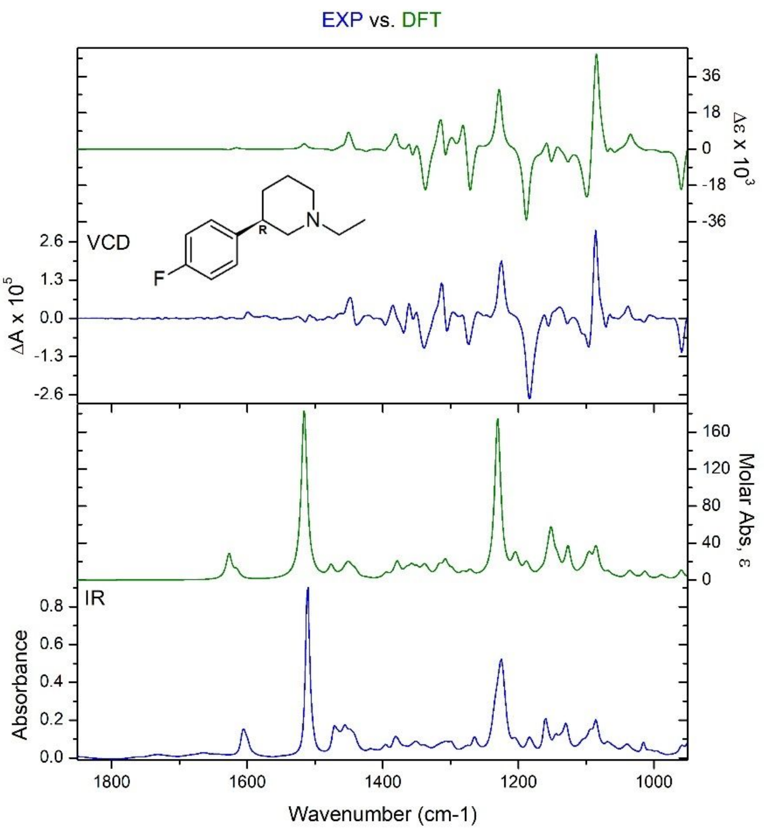

Figure S60: Experimental (blue) and calculated (green) VCD and IR spectra for piperidine (*R*)-**6c**, from the cascade reaction of 6-HDNO and EneIRED-01.

CompareVOA statistical data:

6c (*R*)-configuration

NS (IR) 91.1

NS (VCD – *R*) 82.6237

NS (VCD – *S*) 10.4038

ESI 72.220

Confidence Level 99%

DFT results for **6c** compound (5 conformers at 1% or greater Boltzmann weight)

*There are no imaginary frequencies in any of the conformations

(*R*)-configuration calculated at cc-pVTZ / B3PW91 level

Conformer 1:

Energy = -660.801118 Hartree

Standard orientation:

Center Number	Atomic Number	Atomic Type	Coordinates (Angstroms) X	Y	Z
1	6	0	-0.713855	1.856003	-0.028519
2	6	0	-0.285718	0.477754	0.483562
3	6	0	-1.175166	-0.603971	-0.137700
4	7	0	-2.582907	-0.367815	0.139914
5	6	0	-3.009118	0.911720	-0.408922
6	6	0	-2.207986	2.060030	0.184143
7	6	0	-3.400576	-1.478950	-0.330331
8	6	0	-4.813770	-1.482161	0.229637
9	6	0	2.015316	-0.085508	1.342265
10	6	0	1.178872	0.188451	0.261292
11	6	0	1.740018	0.176402	-1.017438
12	6	0	3.085488	-0.100263	-1.215740
13	6	0	3.875976	-0.365705	-0.113753
14	6	0	3.365338	-0.362783	1.168832
15	9	0	5.184884	-0.633649	-0.297815
16	1	0	-0.484852	1.932776	-1.096854
17	1	0	-0.138648	2.637628	0.473789
18	1	0	-0.467770	0.452204	1.562618
19	1	0	-0.901024	-1.579085	0.272106
20	1	0	-0.983559	-0.648096	-1.226877
21	1	0	-2.901063	0.918498	-1.511235
22	1	0	-4.068512	1.054287	-0.194013
23	1	0	-2.533434	3.000576	-0.266833
24	1	0	-2.422575	2.125029	1.255230
25	1	0	-3.438584	-1.502502	-1.434851
26	1	0	-2.899986	-2.399976	-0.020909
27	1	0	-5.403429	-0.633129	-0.118527
28	1	0	-5.334479	-2.388832	-0.084226
29	1	0	-4.794582	-1.458973	1.320799
30	1	0	1.604996	-0.082306	2.345625
31	1	0	1.122309	0.386845	-1.882207
32	1	0	3.520154	-0.109871	-2.206818
33	1	0	4.012523	-0.573642	2.010156

Conformer 2:

Energy = -660.801057 Hartree

Standard orientation:

Center Number	Atomic Number	Atomic Type	Coordinates (Angstroms) X	Y	Z
1	6	0	0.779424	-1.967746	0.093250
2	6	0	0.392655	-0.545081	0.506746
3	6	0	1.309267	0.466257	-0.190112
4	7	0	2.711348	0.208380	0.097801
5	6	0	3.096561	-1.118829	-0.360090
6	6	0	2.268219	-2.198434	0.317554
7	6	0	3.587949	1.233021	-0.456016
8	6	0	3.478655	2.579780	0.240153
9	6	0	-1.625253	-0.286119	-1.012193
10	6	0	-1.064876	-0.235391	0.265937
11	6	0	-1.896401	0.116337	1.328049
12	6	0	-3.240952	0.408257	1.136903
13	6	0	-3.751031	0.346924	-0.144419
14	6	0	-2.965102	0.004053	-1.228184
15	9	0	-5.054710	0.627802	-0.345406
16	1	0	0.544078	-2.113161	-0.966576
17	1	0	0.184563	-2.695244	0.650890
18	1	0	0.578028	-0.448723	1.581187
19	1	0	1.052793	1.469895	0.150722
20	1	0	1.120576	0.438846	-1.280567
21	1	0	2.984503	-1.208372	-1.458131
22	1	0	4.156362	-1.263058	-0.137240
23	1	0	2.565472	-3.178413	-0.063503
24	1	0	2.484799	-2.189636	1.390255
25	1	0	4.612621	0.867371	-0.351377
26	1	0	3.413542	1.359965	-1.540145
27	1	0	3.643826	2.471717	1.313725
28	1	0	4.232884	3.262965	-0.154942
29	1	0	2.506205	3.050812	0.090965

30	1	0	-1.011570	-0.558673	-1.862344
31	1	0	-1.486911	0.163172	2.330650
32	1	0	-3.884443	0.678865	1.963845
33	1	0	-3.399058	-0.035371	-2.218836

Conformer 3:

Energy = -660.799465 Hartree

Standard orientation:

Center Number	Atomic Number	Atomic Type	Coordinates (Angstroms) X	Y	Z
1	6	0	-0.800917	1.810750	-0.534344
2	6	0	-0.335516	0.700987	0.414806
3	6	0	-1.266121	-0.511605	0.285264
4	7	0	-2.643355	-0.134509	0.537524
5	6	0	-3.120118	0.861137	-0.406038
6	6	0	-2.276546	2.124194	-0.316350
7	6	0	-3.545070	-1.260297	0.731889
8	6	0	-3.782279	-2.185561	-0.463530
9	6	0	2.014644	0.411551	1.276579
10	6	0	1.111204	0.319144	0.218754
11	6	0	1.589914	-0.143921	-1.008664
12	6	0	2.919533	-0.503351	-1.179636
13	6	0	3.778365	-0.393741	-0.102624
14	6	0	3.350362	0.059754	1.129048
15	9	0	5.072640	-0.738617	-0.260875
16	1	0	-0.649338	1.488922	-1.570404
17	1	0	-0.189410	2.704993	-0.390746
18	1	0	-0.444781	1.071629	1.438832
19	1	0	-0.976397	-1.274298	1.013087
20	1	0	-1.128210	-0.955613	-0.716912
21	1	0	-3.098679	0.491211	-1.447390
22	1	0	-4.163573	1.085673	-0.170321
23	1	0	-2.623544	2.851084	-1.054975
24	1	0	-2.415448	2.573569	0.671652
25	1	0	-3.154464	-1.850334	1.566686

26	1	0	-4.505414	-0.852758	1.062485
27	1	0	-2.859067	-2.661186	-0.799556
28	1	0	-4.478196	-2.979153	-0.183284
29	1	0	-4.216170	-1.651460	-1.310668
30	1	0	1.669107	0.767808	2.240385
31	1	0	0.919181	-0.228025	-1.855074
32	1	0	3.289929	-0.861917	-2.131244
33	1	0	4.049476	0.134718	1.951736

Conformer 4:

Energy = -660.797848 Hartree

Standard orientation:

Center Number	Atomic Number	Atomic Type	Coordinates (Angstroms) X	Y	Z
1	6	0	-0.774267	-2.190187	0.449013
2	6	0	-0.300484	-1.319592	-0.727855
3	6	0	-1.468328	-0.466011	-1.228753
4	7	0	-2.127984	0.306140	-0.183221
5	6	0	-2.618033	-0.557597	0.881268
6	6	0	-1.485806	-1.358927	1.504754
7	6	0	-3.175534	1.142590	-0.754713
8	6	0	-3.709653	2.205414	0.191258
9	6	0	2.198253	-1.203599	-0.584164
10	6	0	0.971652	-0.557908	-0.412109
11	6	0	0.988696	0.757979	0.050470
12	6	0	2.183516	1.409544	0.334067
13	6	0	3.370793	0.729192	0.154673
14	6	0	3.402377	-0.573947	-0.303466
15	9	0	4.535022	1.354477	0.429585
16	1	0	0.070131	-2.733428	0.878867
17	1	0	-1.470708	-2.940969	0.059604
18	1	0	-0.050238	-1.997563	-1.549436
19	1	0	-2.190857	-1.143685	-1.722679
20	1	0	-1.115581	0.229965	-1.994134
21	1	0	-3.389524	-1.253726	0.496198

22	1	0	-3.098073	0.058206	1.642201
23	1	0	-1.888396	-2.004959	2.288596
24	1	0	-0.782180	-0.673260	1.985096
25	1	0	-2.748898	1.636654	-1.631543
26	1	0	-4.012745	0.522968	-1.126154
27	1	0	-2.898694	2.829573	0.571082
28	1	0	-4.240149	1.779573	1.043686
29	1	0	-4.413949	2.848789	-0.339414
30	1	0	2.215891	-2.223412	-0.952972
31	1	0	0.049016	1.275620	0.192126
32	1	0	2.195935	2.431584	0.690561
33	1	0	4.350612	-1.076908	-0.442053

Conformer 5:

Energy = -660.797728 Hartree

Standard orientation:

Center Number	Atomic Number	Atomic Type	Coordinates (Angstroms) X	Y	Z
1	6	0	-0.869772	-2.263711	0.012469
2	6	0	-0.453840	-1.100569	-0.904074
3	6	0	-1.626312	-0.126733	-1.061158
4	7	0	-2.196421	0.321185	0.203224
5	6	0	-2.635819	-0.808782	1.011211
6	6	0	-1.486937	-1.758125	1.307312
7	6	0	-3.283111	1.271670	0.004739
8	6	0	-2.839483	2.626331	-0.523143
9	6	0	0.950248	0.642175	0.350221
10	6	0	0.860128	-0.471392	-0.485264
11	6	0	2.050566	-1.036443	-0.948226
12	6	0	3.290180	-0.523007	-0.594985
13	6	0	3.331300	0.580512	0.235494
14	6	0	2.181416	1.175569	0.713580
15	9	0	4.530454	1.092486	0.584409
16	1	0	-0.013487	-2.912017	0.210678
17	1	0	-1.611256	-2.869231	-0.520018

18	1	0	-0.282111	-1.521324	-1.899375
19	1	0	-2.402314	-0.628856	-1.670918
20	1	0	-1.294546	0.744143	-1.627836
21	1	0	-3.445723	-1.367345	0.502050
22	1	0	-3.056198	-0.418238	1.940973
23	1	0	-0.735567	-1.239551	1.909229
24	1	0	-1.853628	-2.595865	1.905269
25	1	0	-4.055005	0.848753	-0.664731
26	1	0	-3.764337	1.414582	0.975581
27	1	0	-2.432333	2.569744	-1.533272
28	1	0	-2.080716	3.066281	0.126468
29	1	0	-3.693661	3.305251	-0.556389
30	1	0	0.039089	1.094656	0.719079
31	1	0	2.010585	-1.896769	-1.607462
32	1	0	4.209907	-0.961583	-0.959795
33	1	0	2.250788	2.041720	1.359165

Analysis of piperidine 7c

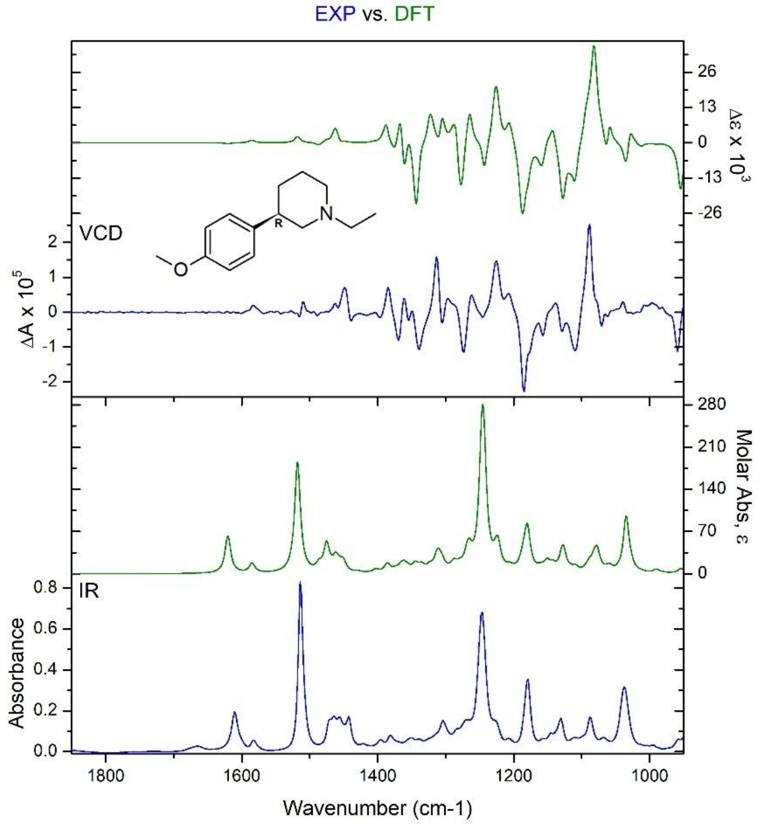

Figure S61: Experimental (blue) and calculated (green) VCD and IR spectra for piperidine (*R*)-**7c**, from the cascade reaction of 6-HDNO and EneIRED-01.

CompareVOA statistical data:

7c (*R*)-configuration

NS (IR) 92.5

NS (VCD – *R*) 84.2007

NS (VCD – *S*) 9.12698

ESI 75.074

Confidence Level 99%

DFT results for **7c** compound (6 conformers at 1% or greater Boltzmann weight)

*There are no imaginary frequencies in any of the conformations

(*R*)-configuration calculated at cc-pVTZ / B3LYP level

Conformer 1:

Energy = -676.346542 Hartree

Standard orientation:

Center Number	Atomic Number	Atomic Type	Coordinates (Angstroms) X	Y	Z
1	6	0	1.216499	1.881854	-0.062651
2	6	0	0.717310	0.488740	-0.480190
3	6	0	1.588282	-0.594744	0.177512
4	7	0	3.004164	-0.427142	-0.144988
5	6	0	3.494380	0.871022	0.317172
6	6	0	2.716286	2.016243	-0.323658
7	6	0	3.798686	-1.545349	0.364812
8	6	0	5.195621	-1.636405	-0.240897
9	6	0	-1.287475	0.342946	1.090700
10	6	0	-0.755242	0.270177	-0.203441
11	6	0	-1.638490	-0.022265	-1.238501
12	6	0	-2.998377	-0.236127	-1.016750
13	6	0	-3.503191	-0.160904	0.279009
14	6	0	-2.633660	0.131570	1.333511
15	8	0	-4.810913	-0.352505	0.615413
16	6	0	-5.739062	-0.649843	-0.420919
17	1	0	1.019510	2.034408	1.002679
18	1	0	0.659578	2.652775	-0.598996
19	1	0	0.866113	0.395536	-1.559495
20	1	0	1.270014	-1.577039	-0.175395
21	1	0	1.425210	-0.578207	1.270374
22	1	0	3.417235	0.946105	1.418410
23	1	0	4.550209	0.960658	0.066569
24	1	0	2.905089	2.012583	-1.400574
25	1	0	3.087400	2.967006	0.064590
26	1	0	3.874880	-1.506172	1.465819
27	1	0	3.255230	-2.461423	0.126268
28	1	0	5.825138	-0.790847	0.035251
29	1	0	5.692923	-2.541258	0.111372
30	1	0	5.139164	-1.677852	-1.329408
31	1	0	-0.641158	0.569090	1.928761
32	1	0	-1.263338	-0.086837	-2.252646
33	1	0	-3.642355	-0.458164	-1.854019

34	1	0	-3.033041	0.189723	2.337045
35	1	0	-6.704689	-0.761164	0.064166
36	1	0	-5.792732	0.160136	-1.151817
37	1	0	-5.481634	-1.580527	-0.931599

Conformer 2:

Energy = -676.346520 Hartree

Standard orientation:

Center Number	Atomic Number	Atomic Type	Coordinates (Angstroms) X	Y	Z
1	6	0	-1.181770	1.871837	-0.176960
2	6	0	-0.739311	0.560284	0.491471
3	6	0	-1.565503	-0.610579	-0.066012
4	7	0	-3.001163	-0.395575	0.105233
5	6	0	-3.434426	0.815915	-0.590190
6	6	0	-2.696465	2.046174	-0.070579
7	6	0	-3.763919	-1.579570	-0.291731
8	6	0	-5.208888	-1.575647	0.196566
9	6	0	1.399737	0.195995	-0.844091
10	6	0	0.748638	0.299095	0.384318
11	6	0	1.527310	0.139360	1.535362
12	6	0	2.888410	-0.109842	1.467115
13	6	0	3.520963	-0.210793	0.226561
14	6	0	2.766798	-0.055993	-0.935734
15	8	0	4.861901	-0.458587	0.253407
16	6	0	5.554867	-0.572674	-0.983795
17	1	0	-0.892608	1.857392	-1.231947
18	1	0	-0.662310	2.716530	0.279967
19	1	0	-0.982860	0.634220	1.554914
20	1	0	-1.291336	-1.528031	0.457313
21	1	0	-1.309215	-0.759680	-1.130691
22	1	0	-3.266423	0.719731	-1.679738
23	1	0	-4.505945	0.943721	-0.444837
24	1	0	-3.019393	2.924986	-0.632519
25	1	0	-2.975927	2.211118	0.973392

26	1	0	-3.744466	-1.712823	-1.387804
27	1	0	-3.255279	-2.446999	0.132747
28	1	0	-5.802190	-0.785817	-0.263611
29	1	0	-5.684968	-2.525813	-0.049908
30	1	0	-5.247323	-1.443909	1.278745
31	1	0	0.841044	0.312552	-1.763710
32	1	0	1.056286	0.212863	2.508016
33	1	0	3.478132	-0.229398	2.365890
34	1	0	3.225780	-0.127873	-1.909971
35	1	0	6.592406	-0.769638	-0.729030
36	1	0	5.166858	-1.399156	-1.583425
37	1	0	5.494025	0.352367	-1.561489

Conformer 3:

Energy = -646.346450 Hartree

Standard orientation:

Center Number	Atomic Number	Atomic Type	Coordinates (Angstroms) X	Y	Z
1	6	0	-1.297921	-1.983706	-0.175153
2	6	0	-0.822854	-0.559334	-0.505368
3	6	0	-1.711177	0.469126	0.216009
4	7	0	-3.124300	0.295143	-0.115420
5	6	0	-3.589555	-1.036246	0.270650
6	6	0	-2.796442	-2.127389	-0.441149
7	6	0	-3.967838	1.344870	0.457520
8	6	0	-3.786292	2.709587	-0.198975
9	6	0	1.181268	-0.483702	1.071688
10	6	0	0.646098	-0.334103	-0.214553
11	6	0	1.523046	0.041095	-1.228146
12	6	0	2.879631	0.261736	-0.993242
13	6	0	3.387624	0.108076	0.294280
14	6	0	2.524284	-0.267342	1.327425
15	8	0	4.692725	0.297282	0.642025
16	6	0	5.614102	0.680271	-0.371999
17	1	0	-1.095798	-2.199724	0.878159

18	1	0	-0.730072	-2.710377	-0.759634
19	1	0	-0.972323	-0.402438	-1.577123
20	1	0	-1.397978	1.470743	-0.074976
21	1	0	-1.553576	0.387665	1.307024
22	1	0	-3.509705	-1.182250	1.364295
23	1	0	-4.647566	-1.116020	0.016137
24	1	0	-3.150095	-3.106498	-0.111441
25	1	0	-2.988487	-2.060086	-1.515394
26	1	0	-5.004665	1.030446	0.325491
27	1	0	-3.805346	1.433243	1.546143
28	1	0	-3.942324	2.639181	-1.276223
29	1	0	-4.512258	3.415246	0.207553
30	1	0	-2.794821	3.126905	-0.025047
31	1	0	0.540136	-0.775298	1.893326
32	1	0	1.145390	0.166615	-2.235664
33	1	0	3.518566	0.549173	-1.814271
34	1	0	2.926006	-0.385295	2.324764
35	1	0	6.578358	0.775120	0.119281
36	1	0	5.681031	-0.077111	-1.156292
37	1	0	5.339987	1.638875	-0.818289

Conformer 4:

Energy = -676.346448 Hartree

Standard orientation:

Center Number	Atomic Number	Atomic Type	Coordinates (Angstroms) X	Y	Z
1	6	0	1.249610	-1.987602	0.024208
2	6	0	0.844411	-0.593190	0.528805
3	6	0	1.689255	0.482720	-0.175375
4	7	0	3.121283	0.254152	0.007835
5	6	0	3.516110	-1.043272	-0.539354
6	6	0	2.761587	-2.182899	0.137430
7	6	0	3.937506	1.337554	-0.541648
8	6	0	3.849011	2.639797	0.247224
9	6	0	-1.295122	-0.327020	-0.828120

10	6	0	-0.639432	-0.317623	0.402228
11	6	0	-1.410366	-0.035348	1.534704
12	6	0	-2.768556	0.223529	1.447036
13	6	0	-3.405779	0.209966	0.204871
14	6	0	-2.659181	-0.067294	-0.939355
15	8	0	-4.743727	0.474930	0.212052
16	6	0	-5.443447	0.463990	-1.026555
17	1	0	0.717139	-2.756876	0.587001
18	1	0	0.947305	-2.096234	-1.021672
19	1	0	1.097751	-0.540857	1.591230
20	1	0	1.432094	0.503875	-1.250331
21	1	0	1.429313	1.458444	0.232336
22	1	0	4.590128	-1.164573	-0.389817
23	1	0	3.336724	-1.084596	-1.630138
24	1	0	3.058620	-3.132417	-0.312559
25	1	0	3.050080	-2.220434	1.191257
26	1	0	4.973669	0.994491	-0.531523
27	1	0	3.682983	1.527423	-1.599298
28	1	0	4.097011	2.470087	1.295787
29	1	0	4.554677	3.366226	-0.158499
30	1	0	2.857432	3.089368	0.200562
31	1	0	-0.742805	-0.542561	-1.733550
32	1	0	-0.935974	-0.019718	2.508315
33	1	0	-3.352641	0.438174	2.331704
34	1	0	-3.121889	-0.085483	-1.914301
35	1	0	-5.056389	1.222768	-1.710427
36	1	0	-5.389261	-0.515922	-1.506155
37	1	0	-6.478763	0.690456	-0.787775

Conformer 5:

Energy = -676.344849 Hartree

Standard orientation:

Center Number	Atomic Number	Atomic Type	Coordinates (Angstroms) X	Y	Z
1	6	0	1.298309	1.841397	0.488978

2	6	0	0.766786	0.708977	-0.406671
3	6	0	1.671922	-0.528475	-0.267465
4	7	0	3.058831	-0.199143	-0.573572
5	6	0	3.596534	0.821052	0.319924
6	6	0	2.779673	2.106720	0.215432
7	6	0	3.926118	-1.360394	-0.761013
8	6	0	4.181834	-2.255682	0.460169
9	6	0	-1.151936	-0.041197	1.099788
10	6	0	-0.688843	0.378507	-0.154520
11	6	0	-1.627025	0.474486	-1.178320
12	6	0	-2.974165	0.171929	-0.983513
13	6	0	-3.409658	-0.244403	0.272048
14	6	0	-2.484417	-0.348747	1.314435
15	8	0	-4.698807	-0.566185	0.580141
16	6	0	-5.682274	-0.474895	-0.443500
17	1	0	1.169237	1.564229	1.539648
18	1	0	0.709507	2.747011	0.329547
19	1	0	0.853133	1.042128	-1.444466
20	1	0	1.340140	-1.302021	-0.963110
21	1	0	1.555325	-0.939922	0.749267
22	1	0	3.603863	0.488850	1.372824
23	1	0	4.634578	1.010018	0.039125
24	1	0	3.168870	2.845499	0.919287
25	1	0	2.899678	2.520873	-0.789155
26	1	0	3.489993	-1.966346	-1.559292
27	1	0	4.884252	-0.992210	-1.136765
28	1	0	3.258808	-2.690686	0.845152
29	1	0	4.842830	-3.078018	0.180819
30	1	0	4.662166	-1.707552	1.271310
31	1	0	-0.462460	-0.130333	1.929106
32	1	0	-1.306142	0.795748	-2.161832
33	1	0	-3.661634	0.265298	-1.810317
34	1	0	-2.830381	-0.671456	2.287169
35	1	0	-6.621722	-0.768458	0.016322
36	1	0	-5.769219	0.545712	-0.822983
37	1	0	-5.459316	-1.150528	-1.272410

Conformer 6:

Energy = -676.344822 Hartree

Standard orientation:

Center Number	Atomic Number	Atomic Type	Coordinates (Angstroms) X	Y	Z
1	6	0	1.276885	-1.750962	-0.754880
2	6	0	0.798837	-0.806159	0.360785
3	6	0	1.656732	0.472200	0.356667
4	7	0	3.073419	0.153612	0.485966
5	6	0	3.555123	-0.683390	-0.607379
6	6	0	2.782596	-1.999751	-0.652688
7	6	0	3.923089	1.304076	0.788311
8	6	0	4.045063	2.393653	-0.286865
9	6	0	-1.268561	0.095389	-0.825238
10	6	0	-0.681179	-0.493340	0.293729
11	6	0	-1.517795	-0.778337	1.378127
12	6	0	-2.873043	-0.492756	1.349453
13	6	0	-3.440973	0.098757	0.219408
14	6	0	-2.628502	0.393829	-0.874259
15	8	0	-4.781183	0.345523	0.279013
16	6	0	-5.408935	0.956792	-0.841715
17	1	0	1.049716	-1.306992	-1.728898
18	1	0	0.727353	-2.693119	-0.704273
19	1	0	0.986599	-1.303895	1.315999
20	1	0	1.368554	1.107040	1.197091
21	1	0	1.438609	1.039703	-0.563797
22	1	0	3.460070	-0.181985	-1.586343
23	1	0	4.618402	-0.875179	-0.449979
24	1	0	3.000419	-2.568856	0.254913
25	1	0	3.126749	-2.596616	-1.500095
26	1	0	3.543906	1.755278	1.708755
27	1	0	4.920377	0.919582	1.017064
28	1	0	3.080178	2.846890	-0.516938
29	1	0	4.705927	3.186495	0.067877
30	1	0	4.466076	2.003499	-1.214142
31	1	0	-0.663672	0.333706	-1.690454

32	1	0	-1.097669	-1.235658	2.265610
33	1	0	-3.508041	-0.720964	2.194797
34	1	0	-3.036525	0.850835	-1.762826
35	1	0	-6.457845	1.059693	-0.578271
36	1	0	-4.988916	1.944645	-1.044201
37	1	0	-5.320760	0.336435	-1.736490

Analysis of piperidine 8c

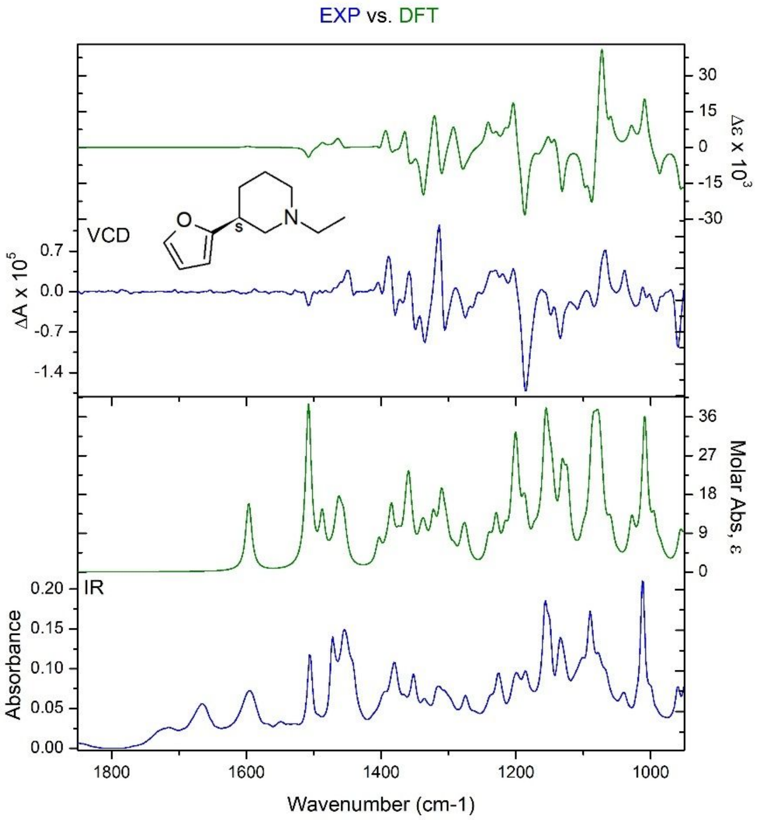

Figure S62: Experimental (blue) and calculated (green) VCD and IR spectra for piperidine (*S*)-**8c**, from the cascade reaction of 6-HDNO and EneIRED-01.

CompareVOA statistical data:

8c (*S*)-configuration

NS (IR) 90.4

NS (VCD – *S*) 72.9351

NS (VCD – *R*) 13.1508

ESI 59.784

Confidence Level 99%

DFT results for **8c** compound (7 conformers at 1% or greater Boltzmann weight)

*There are no imaginary frequencies in any of the conformations

(*S*)-configuration calculated at cc-pVTZ / B3LYP level

Conformer 1:

Energy = -559.561187 Hartree

Standard orientation:

Center Number	Atomic Number	Atomic Type	Coordinates (Angstroms) X	Y	Z
1	6	0	0.139107	1.763105	-0.004803
2	6	0	0.436058	0.345609	0.516800
3	6	0	-0.545316	-0.660530	-0.111276
4	7	0	-1.933348	-0.289662	0.153647
5	6	0	-2.238643	1.023829	-0.413542
6	6	0	-1.338694	2.102697	0.181521
7	6	0	-2.855816	-1.332482	-0.298215
8	6	0	-4.265905	-1.191970	0.265553
9	6	0	2.847822	-0.430128	1.165389
10	6	0	1.855284	-0.061112	0.313350
11	8	0	2.329584	-0.091813	-0.972802
12	6	0	3.635654	-0.485579	-0.917884
13	6	0	4.004379	-0.705774	0.365499
14	1	0	0.397111	1.811599	-1.066091
15	1	0	0.770925	2.489055	0.510043
16	1	0	0.264982	0.333154	1.595988
17	1	0	-0.366637	-1.650387	0.310785
18	1	0	-0.345419	-0.727831	-1.195078
19	1	0	-2.120185	1.009552	-1.513158
20	1	0	-3.280410	1.265254	-0.209145
21	1	0	-1.567862	3.062762	-0.285170
22	1	0	-1.562872	2.200749	1.246951
23	1	0	-2.899627	-1.371519	-1.400734
24	1	0	-2.443945	-2.289029	0.028242
25	1	0	-4.776250	-0.303371	-0.104956
26	1	0	-4.866781	-2.055872	-0.022143
27	1	0	-4.238730	-1.140444	1.354757
28	1	0	2.766560	-0.497121	2.236867
29	1	0	4.147342	-0.557135	-1.860549
30	1	0	4.974973	-1.023271	0.705930

Conformer 2:

Energy = -559.561148 Hartree

Standard orientation:

Center Number	Atomic Number	Atomic Type	Coordinates (Angstroms) X	Y	Z
1	6	0	0.115833	1.882384	0.121826
2	6	0	0.333328	0.415490	0.534454
3	6	0	-0.695930	-0.488961	-0.169060
4	7	0	-2.064237	-0.062773	0.114601
5	6	0	-2.291288	1.304792	-0.352105
6	6	0	-1.343215	2.285616	0.329679
7	6	0	-3.062563	-0.985920	-0.427698
8	6	0	-3.122873	-2.326857	0.296587
9	6	0	2.696803	-0.540389	1.119959
10	6	0	1.729354	-0.051388	0.300097
11	8	0	2.205317	-0.009858	-0.985188
12	6	0	3.486745	-0.480031	-0.962404
13	6	0	3.838484	-0.818663	0.299777
14	1	0	0.383186	1.996984	-0.932024
15	1	0	0.782077	2.532186	0.691994
16	1	0	0.158213	0.330424	1.609682
17	1	0	-0.561968	-1.512109	0.177804
18	1	0	-0.496791	-0.484351	-1.255342
19	1	0	-2.164707	1.375927	-1.448365
20	1	0	-3.325997	1.573048	-0.133733
21	1	0	-1.569971	2.310594	1.398818
22	1	0	-1.517687	3.290182	-0.060710
23	1	0	-4.033531	-0.496293	-0.334618
24	1	0	-2.901059	-1.154501	-1.506737
25	1	0	-3.282023	-2.178112	1.365398
26	1	0	-3.950743	-2.920470	-0.093787
27	1	0	-2.213414	-2.912742	0.166032
28	1	0	2.608381	-0.685607	2.183086
29	1	0	3.996591	-0.507459	-1.908384
30	1	0	4.788499	-1.215875	0.613510

Conformer 3:

Energy = -559.561080 Hartree

Standard orientation:

Center Number	Atomic Number	Atomic Type	Coordinates (Angstroms) X	Y	Z
1	6	0	-0.050020	1.951702	-0.145728
2	6	0	-0.338905	0.479648	-0.457182
3	6	0	0.676135	-0.421575	0.272148
4	7	0	2.048387	-0.080568	-0.091881
5	6	0	2.353050	1.305219	0.263747
6	6	0	1.413512	2.277090	-0.442856
7	6	0	3.027177	-1.008560	0.477273
8	6	0	3.011955	-2.391463	-0.165323
9	6	0	-2.767488	0.684718	0.533655
10	6	0	-1.741641	0.090262	-0.131287
11	8	0	-2.146774	-1.142758	-0.575207
12	6	0	-3.441586	-1.316409	-0.179908
13	6	0	-3.871277	-0.229531	0.501402
14	1	0	-0.256142	2.143101	0.911711
15	1	0	-0.713017	2.598682	-0.722353
16	1	0	-0.188959	0.319529	-1.530406
17	1	0	0.484267	-1.459729	0.010155
18	1	0	0.516852	-0.324826	1.361968
19	1	0	2.282853	1.458554	1.356589
20	1	0	3.385893	1.511462	-0.020464
21	1	0	1.644997	3.297038	-0.130410
22	1	0	1.591277	2.220583	-1.519905
23	1	0	4.013013	-0.565293	0.326105
24	1	0	2.893540	-1.104459	1.568789
25	1	0	3.150125	-2.313135	-1.244375
26	1	0	3.823077	-2.997174	0.241350
27	1	0	2.081610	-2.927781	0.020364
28	1	0	-2.749708	1.657812	0.992117
29	1	0	-3.900460	-2.247173	-0.460395
30	1	0	-4.849210	-0.087756	0.928346

Conformer 4:

Energy = -559.561068 Hartree

Standard orientation:

Center Number	Atomic Number	Atomic Type	Coordinates (Angstroms) X	Y	Z
1	6	0	-0.088204	1.831903	-0.108185
2	6	0	-0.441915	0.381076	-0.449282
3	6	0	0.528423	-0.576504	0.267556
4	7	0	1.916326	-0.293481	-0.087792
5	6	0	2.284525	1.069891	0.293034
6	6	0	1.389558	2.096764	-0.395065
7	6	0	2.821864	-1.301771	0.465301
8	6	0	4.204876	-1.305211	-0.177498
9	6	0	-2.861040	0.680477	0.538501
10	6	0	-1.862956	0.051728	-0.137208
11	8	0	-2.326642	-1.148627	-0.612122
12	6	0	-3.630360	-1.266740	-0.225819
13	6	0	-4.008707	-0.176492	0.480148
14	1	0	-0.719377	2.519337	-0.673602
15	1	0	-0.289020	2.011672	0.952308
16	1	0	-0.292936	0.234346	-1.524517
17	1	0	0.302141	-1.604185	-0.016089
18	1	0	0.363018	-0.494600	1.357388
19	1	0	2.216366	1.199080	1.389426
20	1	0	3.322670	1.246798	0.016556
21	1	0	1.666093	3.098371	-0.060648
22	1	0	1.567799	2.055677	-1.472743
23	1	0	2.921317	-1.186644	1.558663
24	1	0	2.358571	-2.275854	0.299057
25	1	0	4.761176	-0.390251	0.025045
26	1	0	4.792497	-2.136799	0.214121
27	1	0	4.123489	-1.422032	-1.258868
28	1	0	-2.797071	1.640319	1.020289
29	1	0	-4.133840	-2.166289	-0.530551
30	1	0	-4.980430	0.003708	0.906788

Conformer 5:

Energy = -559.559367 Hartree

Standard orientation:

Center Number	Atomic Number	Atomic Type	Coordinates (Angstroms) X	Y	Z
1	6	0	0.001869	1.867617	0.132102
2	6	0	-0.399997	0.519370	-0.477008
3	6	0	0.629761	-0.558188	-0.080857
4	7	0	1.968741	-0.166656	-0.497713
5	6	0	2.398931	1.080064	0.126600
6	6	0	1.445012	2.215959	-0.236262
7	6	0	2.953616	-1.245925	-0.443656
8	6	0	3.308197	-1.807098	0.940597
9	6	0	-2.704652	0.563708	0.791672
10	6	0	-1.786028	0.114240	-0.104031
11	8	0	-2.306035	-0.959043	-0.782190
12	6	0	-3.564022	-1.178608	-0.300772
13	6	0	-3.859235	-0.276087	0.662860
14	1	0	-0.679134	2.651654	-0.202988
15	1	0	-0.089344	1.810648	1.221290
16	1	0	-0.358869	0.608566	-1.567815
17	1	0	0.555088	-0.720048	1.008065
18	1	0	0.378224	-1.502248	-0.565548
19	1	0	3.407256	1.309321	-0.223247
20	1	0	2.450749	0.997340	1.226095
21	1	0	1.753212	3.129176	0.276540
22	1	0	1.513836	2.407377	-1.310100
23	1	0	3.864665	-0.877609	-0.921941
24	1	0	2.578666	-2.058740	-1.070658
25	1	0	2.436011	-2.226899	1.443046
26	1	0	4.044941	-2.605363	0.835433
27	1	0	3.738985	-1.044161	1.589839
28	1	0	-2.583804	1.394423	1.464528
29	1	0	-4.103498	-1.996053	-0.743891

30	1	0	-4.780606	-0.208775	1.215384

Conformer 6:

Energy = -559.559554 Hartree

Standard orientation:

Center Number	Atomic Number	Atomic Type	Coordinates (Angstroms) X	Y	Z
1	6	0	0.069222	1.766888	-0.387844
2	6	0	0.402769	0.552372	0.498479
3	6	0	-0.649326	-0.553861	0.286582
4	7	0	-1.990406	-0.045648	0.546298
5	6	0	-2.347830	1.054256	-0.343730
6	6	0	-1.375257	2.218162	-0.170405
7	6	0	-3.012017	-1.084962	0.664854
8	6	0	-3.331485	-1.908144	-0.591413
9	6	0	2.859520	-0.098269	1.116587
10	6	0	1.790337	0.050120	0.290491
11	8	0	2.133494	-0.365956	-0.970570
12	6	0	3.434069	-0.778685	-0.925764
13	6	0	3.927094	-0.636401	0.326524
14	1	0	0.763484	2.581910	-0.175567
15	1	0	0.213988	1.491172	-1.435977
16	1	0	0.340475	0.862316	1.544391
17	1	0	-0.539563	-0.949117	-0.736084
18	1	0	-0.453461	-1.378662	0.974098
19	1	0	-3.363456	1.373569	-0.102313
20	1	0	-2.350222	0.745700	-1.403274
21	1	0	-1.630394	3.015861	-0.871002
22	1	0	-1.486003	2.624248	0.838446
23	1	0	-3.927539	-0.599842	1.012631
24	1	0	-2.695491	-1.762253	1.462054
25	1	0	-2.459796	-2.455485	-0.951944
26	1	0	-4.108332	-2.639565	-0.362045
27	1	0	-3.698390	-1.281436	-1.405076
28	1	0	2.885457	0.147312	2.164559

29	1	0	3.848108	-1.136448	-1.851087
30	1	0	4.923450	-0.880953	0.652490

Conformer 7:

Energy = -559.558695 Hartree

Standard orientation:

Center Number	Atomic Number	Atomic Type	Coordinates (Angstroms) X	Y	Z
1	6	0	0.257392	2.083269	-0.072660
2	6	0	0.543591	0.902738	0.881128
3	6	0	-0.777953	0.247066	1.300247
4	7	0	-1.598367	-0.162600	0.161166
5	6	0	-1.920218	0.982805	-0.691378
6	6	0	-0.655622	1.655477	-1.219283
7	6	0	-2.788714	-0.885711	0.609941
8	6	0	-3.507119	-1.648181	-0.498367
9	6	0	1.474046	-1.336330	-0.174098
10	6	0	1.529882	-0.069688	0.315176
11	8	0	2.827675	0.375433	0.265460
12	6	0	3.586867	-0.629196	-0.261359
13	6	0	2.810037	-1.699935	-0.545589
14	1	0	-0.231612	2.873872	0.503602
15	1	0	1.193380	2.497057	-0.449260
16	1	0	0.997771	1.311058	1.788774
17	1	0	-1.321975	0.968315	1.936275
18	1	0	-0.578213	-0.632067	1.913725
19	1	0	-2.530025	0.641037	-1.525976
20	1	0	-2.523387	1.723821	-0.132788
21	1	0	-0.933262	2.520770	-1.824285
22	1	0	-0.129380	0.958947	-1.875496
23	1	0	-2.464151	-1.599551	1.369251
24	1	0	-3.499182	-0.202846	1.108714
25	1	0	-2.823444	-2.334695	-0.999311
26	1	0	-4.325494	-2.230785	-0.072973
27	1	0	-3.936371	-0.986350	-1.250211

28	1	0	0.583061	-1.929499	-0.273515
29	1	0	4.637475	-0.424811	-0.361610
30	1	0	3.138851	-2.633420	-0.969298

Analysis of piperidine 9c

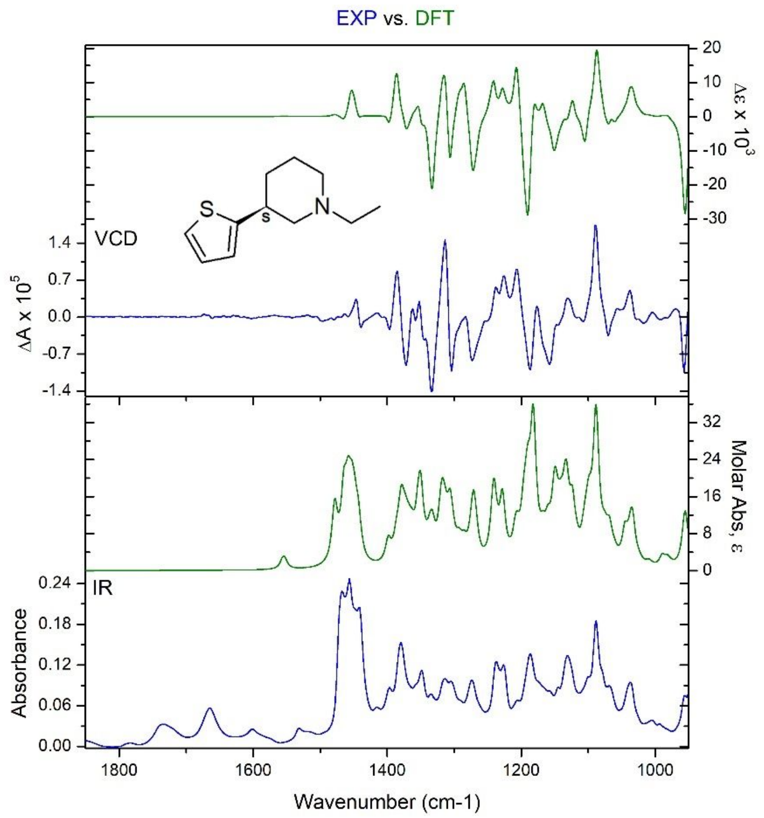

Figure S63: Experimental (blue) and calculated (green) VCD and IR spectra for piperidine (*S*)-**9c**, from the cascade reaction of 6-HDNO and EneIRED-01.

CompareVOA statistical data:

9c (*S*)-configuration

NS (IR) 92.8

NS (VCD – *S*) 74.8042

NS (VCD – *R*) 16.4716

ESI 58.333

Confidence Level 98%

DFT results for **9c** compound (8 conformers at 1% or greater Boltzmann weight)

*There are no imaginary frequencies in any of the conformations

(*S*)-configuration calculated at cc-pVTZ / B3PW91 level

Conformer 1:

Energy = -882.314212 Hartree

Standard orientation:

Center Number	Atomic Number	Atomic Type	Coordinates (Angstroms) X	Y	Z
1	6	0	-0.154225	1.792558	-0.000889
2	6	0	0.169559	0.402010	0.556036
3	6	0	-0.777187	-0.632922	-0.061437
4	7	0	-2.169143	-0.297178	0.186145
5	6	0	-2.501397	0.991924	-0.404566
6	6	0	-1.635133	2.100416	0.173055
7	6	0	-3.052586	-1.366491	-0.262712
8	6	0	-4.471992	-1.252757	0.268633
9	6	0	2.516647	-0.246611	1.383527
10	6	0	1.607356	0.017934	0.398042
11	16	0	2.358898	-0.152993	-1.156912
12	6	0	3.874355	-0.574275	-0.462507
13	6	0	3.807895	-0.583516	0.897287
14	1	0	0.103623	1.819919	-1.065422
15	1	0	0.462837	2.546563	0.492974
16	1	0	-0.028445	0.413863	1.632856
17	1	0	-0.573631	-1.614812	0.371522
18	1	0	-0.566885	-0.708916	-1.145836
19	1	0	-2.374922	0.960171	-1.504212
20	1	0	-3.551869	1.210833	-0.211794
21	1	0	-1.886139	3.046834	-0.311793
22	1	0	-1.866059	2.212532	1.236677
23	1	0	-3.072193	-1.426969	-1.366133
24	1	0	-2.621867	-2.307396	0.088836
25	1	0	-4.998653	-0.384193	-0.128536
26	1	0	-5.045491	-2.137155	-0.015253
27	1	0	-4.469387	-1.181577	1.357879
28	1	0	2.262698	-0.199163	2.434551
29	1	0	4.718313	-0.789884	-1.098201
30	1	0	4.649943	-0.822122	1.532647

Conformer 2:

Energy = -882.314212 Hartree

Standard orientation:

Center Number	Atomic Number	Atomic Type	Coordinates (Angstroms) X	Y	Z
1	6	0	-0.187428	1.907389	0.179202
2	6	0	0.069988	0.452609	0.586263
3	6	0	-0.916235	-0.470121	-0.140022
4	7	0	-2.293930	-0.097840	0.132699
5	6	0	-2.560775	1.261106	-0.318034
6	6	0	-1.654487	2.261045	0.381274
7	6	0	-3.248731	-1.040840	-0.437244
8	6	0	-3.267397	-2.393327	0.256083
9	6	0	2.372315	-0.409778	1.342011
10	6	0	1.489819	0.020935	0.391407
11	16	0	2.250567	0.004319	-1.168297
12	6	0	3.733592	-0.578828	-0.522645
13	6	0	3.650300	-0.751826	0.825234
14	1	0	0.078667	2.035253	-0.875962
15	1	0	0.459063	2.576482	0.751460
16	1	0	-0.134194	0.359061	1.657856
17	1	0	-0.749219	-1.496188	0.188050
18	1	0	-0.704007	-0.439868	-1.226542
19	1	0	-2.426317	1.349622	-1.413363
20	1	0	-3.607467	1.492163	-0.107288
21	1	0	-1.859360	3.265219	0.002594
22	1	0	-1.888763	2.264763	1.450103
23	1	0	-4.238593	-0.586824	-0.344702
24	1	0	-3.071745	-1.179305	-1.519291
25	1	0	-3.442033	-2.273433	1.326869
26	1	0	-4.069935	-3.008764	-0.154769
27	1	0	-2.336012	-2.944988	0.122473
28	1	0	2.108187	-0.478594	2.389329
29	1	0	4.573184	-0.758278	-1.175131
30	1	0	4.471208	-1.110740	1.430991

Conformer 3:

Energy = -882.313606 Hartree

Standard orientation:

Center Number	Atomic Number	Atomic Type	Coordinates (Angstroms) X	Y	Z
1	6	0	0.270096	1.952300	-0.119295
2	6	0	-0.072366	0.483895	-0.387613
3	6	0	0.949161	-0.424691	0.308231
4	7	0	2.306234	-0.126744	-0.119700
5	6	0	2.657511	1.249623	0.200122
6	6	0	1.721660	2.231277	-0.486446
7	6	0	3.280016	-1.062562	0.429331
8	6	0	3.185188	-2.462874	-0.154119
9	6	0	-2.079189	0.264755	1.240953
10	6	0	-1.472853	0.135407	0.021362
11	16	0	-2.577209	-0.557307	-1.114738
12	6	0	-3.835620	-0.666518	0.048664
13	6	0	-3.423712	-0.191408	1.257858
14	1	0	-0.406855	2.602768	-0.677621
15	1	0	0.114268	2.167751	0.942849
16	1	0	0.028567	0.307105	-1.463258
17	1	0	0.717027	-1.463620	0.073155
18	1	0	0.841765	-0.309347	1.403628
19	1	0	2.630179	1.422327	1.293429
20	1	0	3.686483	1.423415	-0.123008
21	1	0	1.995690	3.251589	-0.207699
22	1	0	1.851566	2.146368	-1.569601
23	1	0	4.270819	-0.657864	0.208000
24	1	0	3.205009	-1.112461	1.530893
25	1	0	3.255538	-2.430534	-1.242911
26	1	0	4.004439	-3.077496	0.223703
27	1	0	2.254560	-2.965507	0.112279
28	1	0	-1.579665	0.676656	2.107687
29	1	0	-4.798413	-1.067283	-0.225260
30	1	0	-4.058626	-0.164974	2.133145

Conformer 4:

Energy = -882.313604 Hartree

Standard orientation:

Center Number	Atomic Number	Atomic Type	Coordinates (Angstroms) X	Y	Z
1	6	0	0.222629	1.832232	-0.080543
2	6	0	-0.173048	0.382407	-0.375156
3	6	0	0.808348	-0.571801	0.316017
4	7	0	2.179510	-0.321378	-0.097786
5	6	0	2.582402	1.035206	0.244365
6	6	0	1.686733	2.061658	-0.431564
7	6	0	3.079949	-1.330315	0.446544
8	6	0	4.447744	-1.354798	-0.215798
9	6	0	-2.205492	0.224352	1.228917
10	6	0	-1.589578	0.081238	0.015548
11	16	0	-2.704504	-0.562903	-1.138382
12	6	0	-3.980370	-0.632804	0.008644
13	6	0	-3.566005	-0.181928	1.226385
14	1	0	0.065975	2.036611	0.983679
15	1	0	-0.424321	2.516859	-0.633609
16	1	0	-0.068079	0.218108	-1.452444
17	1	0	0.551633	-1.602782	0.062331
18	1	0	0.689873	-0.470009	1.411340
19	1	0	2.552286	1.185972	1.341184
20	1	0	3.615598	1.187449	-0.068630
21	1	0	1.996672	3.066045	-0.133215
22	1	0	1.822018	1.991405	-1.515141
23	1	0	3.197691	-1.208045	1.538726
24	1	0	2.601685	-2.301610	0.296868
25	1	0	5.021948	-0.447702	-0.023186
26	1	0	5.029813	-2.194670	0.168229
27	1	0	4.348935	-1.472774	-1.296448
28	1	0	-1.702029	0.611619	2.104701
29	1	0	-4.953949	-0.995282	-0.280151
30	1	0	-4.210124	-0.137789	2.094220

Conformer 5:

Energy = -882.312673 Hartree

Standard orientation:

Center Number	Atomic Number	Atomic Type	Coordinates (Angstroms) X	Y	Z
1	6	0	-0.222445	1.770676	-0.487168
2	6	0	0.138963	0.649687	0.494669
3	6	0	-0.872124	-0.496694	0.358838
4	7	0	-2.223421	-0.017830	0.571843
5	6	0	-2.606212	0.994516	-0.397750
6	6	0	-1.674867	2.194396	-0.309299
7	6	0	-3.206760	-1.075111	0.757700
8	6	0	-3.480043	-1.997845	-0.431645
9	6	0	2.547856	0.232067	1.294968
10	6	0	1.552756	0.178682	0.359479
11	16	0	2.155765	-0.542254	-1.099788
12	6	0	3.726231	-0.758303	-0.434187
13	6	0	3.786175	-0.299844	0.846425
14	1	0	-0.068970	1.410904	-1.510840
15	1	0	0.451235	2.619142	-0.347534
16	1	0	0.040810	1.044722	1.511019
17	1	0	-0.653490	-1.267354	1.102178
18	1	0	-0.740625	-0.960886	-0.635387
19	1	0	-2.588968	0.605889	-1.431927
20	1	0	-3.635094	1.298128	-0.188567
21	1	0	-1.802064	2.672019	0.666885
22	1	0	-1.950598	2.929981	-1.068832
23	1	0	-2.878224	-1.679123	1.609003
24	1	0	-4.143187	-0.596635	1.060125
25	1	0	-2.585062	-2.541322	-0.739866
26	1	0	-4.235533	-2.737096	-0.157260
27	1	0	-3.856684	-1.447364	-1.295521
28	1	0	2.393104	0.645693	2.283114
29	1	0	4.505950	-1.210312	-1.026312
30	1	0	4.681931	-0.338875	1.451245

Conformer 6:

Energy = -882.312024 Hartree

Standard orientation:

Center Number	Atomic Number	Atomic Type	Coordinates (Angstroms) X	Y	Z
1	6	0	0.306903	1.811184	0.450764
2	6	0	-0.135736	0.563857	-0.322321
3	6	0	0.899713	-0.553324	-0.128076
4	7	0	2.214550	-0.111942	-0.549377
5	6	0	2.681639	1.027095	0.222325
6	6	0	1.728436	2.202903	0.065874
7	6	0	3.188635	-1.185639	-0.683710
8	6	0	3.600863	-1.923250	0.591804
9	6	0	-2.019604	-0.210539	1.286910
10	6	0	-1.512195	0.105358	0.055960
11	16	0	-2.723504	-0.125794	-1.156608
12	6	0	-3.893115	-0.639867	-0.009363
13	6	0	-3.374287	-0.635038	1.250878
14	1	0	-0.387748	2.632976	0.262682
15	1	0	0.264088	1.599494	1.524416
16	1	0	-0.133721	0.812928	-1.388260
17	1	0	0.615713	-1.423780	-0.724762
18	1	0	0.875630	-0.865292	0.931075
19	1	0	2.779141	0.786971	1.296445
20	1	0	3.678200	1.298842	-0.134967
21	1	0	2.071040	3.037266	0.682837
22	1	0	1.748121	2.540004	-0.974915
23	1	0	2.780541	-1.907863	-1.397285
24	1	0	4.080362	-0.757454	-1.151523
25	1	0	2.752268	-2.416050	1.069801
26	1	0	4.335300	-2.694441	0.349953
27	1	0	4.057383	-1.251161	1.320787
28	1	0	-1.442337	-0.141401	2.199259
29	1	0	-4.887570	-0.909007	-0.327615

30	1	0	-3.939707	-0.922172	2.127159

Conformer 7:

Energy = -882.311811 Hartree

Standard orientation:

Center Number	Atomic Number	Atomic Type	Coordinates (Angstroms) X	Y	Z
1	6	0	-0.110243	2.239496	-0.377157
2	6	0	0.322828	1.283641	0.748840
3	6	0	-0.908981	0.564648	1.303010
4	7	0	-1.648038	-0.128768	0.261422
5	6	0	-2.140772	0.814176	-0.733875
6	6	0	-0.990985	1.547860	-1.407044
7	6	0	-2.704791	-0.956217	0.830347
8	6	0	-3.304181	-1.955689	-0.145157
9	6	0	2.765682	0.533737	0.669270
10	6	0	1.442314	0.357034	0.368258
11	16	0	1.238525	-1.108966	-0.537656
12	6	0	2.923886	-1.452747	-0.500650
13	6	0	3.612564	-0.492993	0.176979
14	1	0	0.767992	2.686406	-0.847303
15	1	0	-0.676514	3.056582	0.083267
16	1	0	0.725029	1.896329	1.560903
17	1	0	-1.545904	1.312077	1.812553
18	1	0	-0.603472	-0.164975	2.055890
19	1	0	-2.718987	0.271889	-1.481935
20	1	0	-2.825050	1.548480	-0.265583
21	1	0	-0.403314	0.832200	-1.987782
22	1	0	-1.390301	2.279600	-2.113539
23	1	0	-2.266225	-1.503808	1.668161
24	1	0	-3.507872	-0.327840	1.256837
25	1	0	-2.527544	-2.588428	-0.577987
26	1	0	-4.014906	-2.598158	0.378035
27	1	0	-3.844675	-1.473258	-0.960351
28	1	0	3.120405	1.383526	1.238639

29	1	0	3.303839	-2.345855	-0.971494
30	1	0	4.683442	-0.517607	0.327308

Conformer 8:

Energy = -882.311622 Hartree

Standard orientation:

Center Number	Atomic Number	Atomic Type	Coordinates (Angstroms) X	Y	Z
1	6	0	-0.229189	2.299167	0.173432
2	6	0	0.123790	1.048077	0.997825
3	6	0	-1.131061	0.186968	1.172872
4	7	0	-1.733868	-0.161210	-0.102298
5	6	0	-2.153479	1.037719	-0.816253
6	6	0	-0.971518	1.947653	-1.107614
7	6	0	-2.839840	-1.101005	0.040967
8	6	0	-2.418906	-2.503605	0.446961
9	6	0	2.585302	0.357208	0.978626
10	6	0	1.312845	0.290812	0.478625
11	16	0	1.271697	-0.796245	-0.873101
12	6	0	2.953972	-1.132860	-0.748771
13	6	0	3.522391	-0.450737	0.283831
14	1	0	0.673728	2.874602	-0.040356
15	1	0	-0.872140	2.932426	0.794470
16	1	0	0.416633	1.385084	1.996422
17	1	0	-1.848318	0.745248	1.804635
18	1	0	-0.872767	-0.725712	1.709614
19	1	0	-2.912592	1.595685	-0.234188
20	1	0	-2.630674	0.731233	-1.749707
21	1	0	-1.324755	2.857299	-1.599349
22	1	0	-0.299605	1.446696	-1.809251
23	1	0	-3.589334	-0.716105	0.756300
24	1	0	-3.341392	-1.154136	-0.928460
25	1	0	-3.291343	-3.159758	0.456953
26	1	0	-1.694215	-2.910219	-0.259995
27	1	0	-1.977600	-2.537365	1.443867

28	1	0	2.839644	0.973144	1.831828
29	1	0	3.419620	-1.821673	-1.436087
30	1	0	4.569194	-0.524437	0.546005

Analysis of piperidine 10c

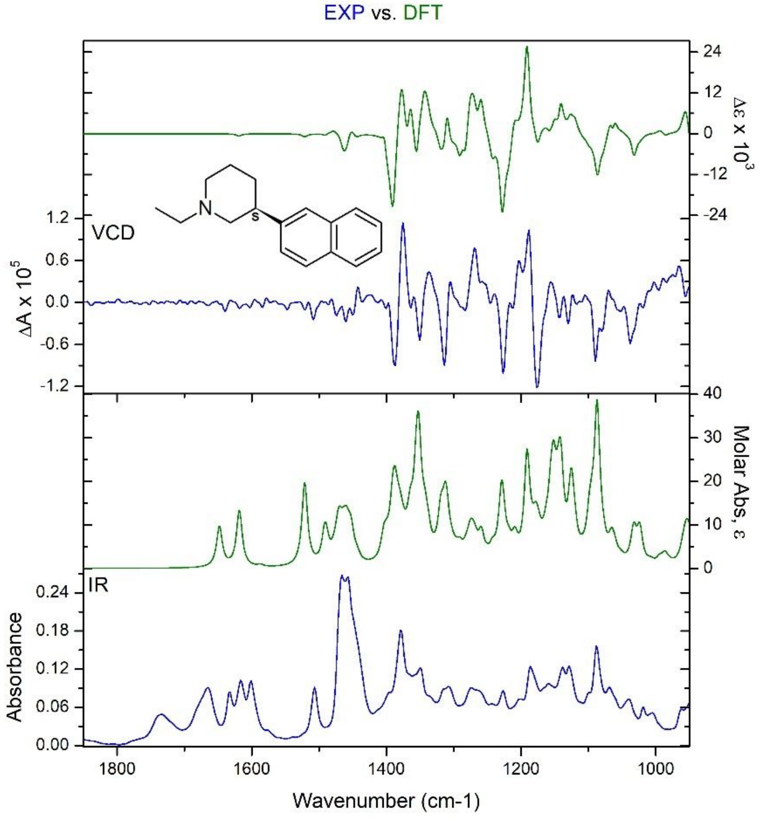

Figure S64: Experimental (blue) and calculated (green) VCD and IR spectra for piperidine (*S*)-**10c**, from the cascade reaction of 6-HDNO and EneIRED-07.

CompareVOA statistical data:

10c (*S*)-configuration

NS (IR) 85.5

NS (VCD – *S*) 70.4232

NS (VCD – *R*) 17.6977

ESI 52.726

Confidence Level 93%

DFT results for **10c** compound (9 conformers at 1% or greater Boltzmann weight)

*There are no imaginary frequencies in any of the conformations

(*S*)-configuration calculated at 6-31G(d) / B3PW91 level

Conformer 1:

Energy = -714.966781 Hartree

Standard orientation:

Center Number	Atomic Number	Atomic Type	Coordinates (Angstroms) X	Y	Z
1	6	0	-1.687038	-1.944655	-0.448657
2	6	0	-1.156024	-0.503673	-0.508820
3	6	0	-2.070858	0.423151	0.310102
4	7	0	-3.451684	0.361798	-0.156164
5	6	0	-3.977389	-0.994312	-0.026760
6	6	0	-3.159751	-1.981631	-0.853234
7	6	0	-4.308856	1.324839	0.531870
8	6	0	-4.029743	2.776226	0.155843
9	6	0	1.252693	0.074891	-0.948688
10	6	0	0.289058	-0.387696	-0.075638
11	6	0	3.000763	-0.165467	0.757603
12	6	0	2.614738	0.198081	-0.570351
13	6	0	0.690369	-0.749014	1.241144
14	6	0	1.999777	-0.640170	1.644368
15	6	0	4.361737	-0.040350	1.138940
16	6	0	3.608673	0.672804	-1.466001
17	6	0	5.302005	0.423732	0.247533
18	6	0	4.921166	0.783394	-1.067244
19	1	0	-1.582024	-2.331416	0.575264
20	1	0	-1.086121	-2.594945	-1.096244
21	1	0	-1.222103	-0.167412	-1.552775
22	1	0	-1.706804	1.451141	0.215698
23	1	0	-2.001925	0.154881	1.385799
24	1	0	-3.987100	-1.323511	1.034360
25	1	0	-5.019621	-0.992634	-0.368518
26	1	0	-3.568167	-2.991847	-0.726598
27	1	0	-3.258546	-1.721502	-1.915624
28	1	0	-5.343689	1.087318	0.255197
29	1	0	-4.246407	1.204979	1.632782
30	1	0	-4.073832	2.906033	-0.931021
31	1	0	-4.782587	3.429217	0.611281
32	1	0	-3.049652	3.121094	0.501536
33	1	0	0.970469	0.357640	-1.961809

34	1	0	-0.052653	-1.118077	1.944011
35	1	0	2.285049	-0.920463	2.656254
36	1	0	4.650507	-0.318332	2.150396
37	1	0	3.314885	0.949706	-2.476289
38	1	0	6.341906	0.515722	0.550239
39	1	0	5.672283	1.148918	-1.762840

Conformer 2:

Energy = -714.966742 Hartree

Standard orientation:

Center Number	Atomic Number	Atomic Type	Coordinates (Angstroms) X	Y	Z
1	6	0	1.616595	1.702912	-0.834137
2	6	0	1.058905	0.296306	-0.565773
3	6	0	1.947812	-0.426053	0.460159
4	7	0	3.333439	-0.501938	0.009658
5	6	0	3.886597	0.837138	-0.172427
6	6	0	3.093790	1.620879	-1.214529
7	6	0	4.131394	-1.315956	0.924043
8	6	0	5.489840	-1.719117	0.360151
9	6	0	-1.347104	-0.374507	-0.869237
10	6	0	-0.392855	0.304248	-0.138709
11	6	0	-3.116708	0.320381	0.683104
12	6	0	-2.714847	-0.388744	-0.491776
13	6	0	-0.809226	1.005135	1.027666
14	6	0	-2.124780	1.012344	1.425379
15	6	0	-4.483942	0.307741	1.062300
16	6	0	-3.699525	-1.084356	-1.241557
17	6	0	-5.414954	-0.376301	0.314392
18	6	0	-5.018297	-1.079014	-0.848286
19	1	0	1.508285	2.318931	0.070009
20	1	0	1.035100	2.194562	-1.623792
21	1	0	1.132379	-0.275402	-1.501033
22	1	0	1.574846	-1.447262	0.605700
23	1	0	1.868876	0.087833	1.441405

24	1	0	3.891394	1.397509	0.787234
25	1	0	4.928800	0.750630	-0.495793
26	1	0	3.198034	1.122093	-2.187348
27	1	0	3.520681	2.626099	-1.317888
28	1	0	4.268042	-0.807773	1.900478
29	1	0	3.555852	-2.227211	1.129474
30	1	0	6.165091	-0.865985	0.237619
31	1	0	5.979156	-2.425786	1.039662
32	1	0	5.371578	-2.204774	-0.614634
33	1	0	-1.052185	-0.918912	-1.765222
34	1	0	-0.073235	1.546426	1.617016
35	1	0	-2.422178	1.554427	2.320674
36	1	0	-4.785263	0.849100	1.956670
37	1	0	-3.393491	-1.623959	-2.135341
38	1	0	-6.459750	-0.379300	0.614212
39	1	0	-5.762267	-1.615887	-1.431291

Conformer 3:

Energy = -714.966301 Hartree

Standard orientation:

Center Number	Atomic Number	Atomic Type	Coordinates (Angstroms) X	Y	Z
1	6	0	1.494747	0.706326	1.736042
2	6	0	1.107005	0.803161	0.253096
3	6	0	1.876508	-0.256834	-0.552629
4	7	0	3.318675	-0.112844	-0.388645
5	6	0	3.694341	-0.281429	1.011884
6	6	0	3.013522	0.764099	1.890808
7	6	0	4.037967	-1.024904	-1.275096
8	6	0	5.520023	-0.695768	-1.420497
9	6	0	-1.122765	-0.388492	0.426519
10	6	0	-0.386277	0.707217	0.017554
11	6	0	-3.187772	0.595309	-0.468537
12	6	0	-2.520139	-0.478056	0.201088
13	6	0	-1.065012	1.762309	-0.649385

14	6	0	-2.418857	1.711712	-0.884741
15	6	0	-4.587053	0.506161	-0.689766
16	6	0	-3.282222	-1.600213	0.622576
17	6	0	-5.297099	-0.594464	-0.268650
18	6	0	-4.637595	-1.657694	0.393858
19	1	0	1.124454	-0.242957	2.148449
20	1	0	1.011450	1.509273	2.306258
21	1	0	1.444992	1.781680	-0.115295
22	1	0	1.635950	-0.148129	-1.617391
23	1	0	1.529889	-1.266581	-0.246906
24	1	0	3.428724	-1.298226	1.373367
25	1	0	4.780861	-0.184830	1.102683
26	1	0	3.299499	0.603256	2.937616
27	1	0	3.378345	1.759471	1.603902
28	1	0	3.918858	-2.079056	-0.951443
29	1	0	3.567183	-0.951956	-2.263438
30	1	0	6.079027	-0.851053	-0.491893
31	1	0	5.971060	-1.340331	-2.183195
32	1	0	5.652422	0.347061	-1.728847
33	1	0	-0.636125	-1.215109	0.940901
34	1	0	-0.493913	2.628791	-0.976618
35	1	0	-2.917195	2.533426	-1.394830
36	1	0	-5.090313	1.325326	-1.199104
37	1	0	-2.774634	-2.416554	1.132168
38	1	0	-6.368426	-0.652000	-0.443054
39	1	0	-5.208776	-2.522243	0.722430

Conformer 4:

Energy = -714.966259 Hartree

Standard orientation:

Center Number	Atomic Number	Atomic Type	Coordinates (Angstroms) X	Y	Z
1	6	0	1.566505	-1.987838	0.038201
2	6	0	1.208794	-0.613746	0.624708
3	6	0	1.983564	0.487047	-0.119883

4	7	0	3.422890	0.261320	-0.058338
5	6	0	3.768677	-1.010798	-0.686133
6	6	0	3.082545	-2.176293	0.019535
7	6	0	4.181818	1.363020	-0.646282
8	6	0	4.141702	2.645497	0.178120
9	6	0	-1.027137	-0.306156	-0.527985
10	6	0	-0.280908	-0.339561	0.634876
11	6	0	-3.075206	0.188863	0.734737
12	6	0	-2.421103	-0.044735	-0.515882
13	6	0	-0.946417	-0.102635	1.867612
14	6	0	-2.296816	0.151317	1.919379
15	6	0	-4.470842	0.447582	0.745775
16	6	0	-3.192845	-0.008513	-1.707869
17	6	0	-5.190326	0.475598	-0.426547
18	6	0	-4.544303	0.245249	-1.665057
19	1	0	1.178270	-2.057544	-0.988010
20	1	0	1.079539	-2.783531	0.615427
21	1	0	1.562274	-0.594417	1.664898
22	1	0	1.752226	1.454316	0.337526
23	1	0	1.630934	0.535956	-1.171960
24	1	0	3.490357	-1.017741	-1.761692
25	1	0	4.857678	-1.132849	-0.638411
26	1	0	3.347231	-3.115335	-0.482050
27	1	0	3.460555	-2.239184	1.048674
28	1	0	5.224643	1.030079	-0.720422
29	1	0	3.848874	1.574225	-1.682991
30	1	0	4.458282	2.449329	1.208360
31	1	0	4.821592	3.387427	-0.255366
32	1	0	3.144656	3.097091	0.207131
33	1	0	-0.550632	-0.484913	-1.490091
34	1	0	-0.367624	-0.125949	2.788726
35	1	0	-2.784774	0.327260	2.875773
36	1	0	-4.963354	0.624030	1.699729
37	1	0	-2.695759	-0.185766	-2.659299
38	1	0	-6.258652	0.674954	-0.406364
39	1	0	-5.122762	0.269936	-2.585065

Conformer 5:

Energy = -714.965746 Hartree

Standard orientation:

Center Number	Atomic Number	Atomic Type	Coordinates (Angstroms) X	Y	Z
1	6	0	-1.675731	-1.672650	0.909453
2	6	0	-1.097663	-0.742951	-0.170571
3	6	0	-2.027370	0.469737	-0.358872
4	7	0	-3.377379	0.038610	-0.689992
5	6	0	-3.961538	-0.777655	0.366081
6	6	0	-3.125312	-2.035285	0.586950
7	6	0	-4.242305	1.117255	-1.156240
8	6	0	-4.584560	2.229360	-0.156298
9	6	0	1.343586	-0.634660	-0.767174
10	6	0	0.327720	-0.323330	0.113887
11	6	0	3.004408	0.474681	0.659341
12	6	0	2.690056	-0.255995	-0.528733
13	6	0	0.657890	0.402657	1.292731
14	6	0	1.950256	0.789690	1.555526
15	6	0	4.350167	0.854550	0.899840
16	6	0	3.737783	-0.578158	-1.431080
17	6	0	5.343868	0.527096	0.005808
18	6	0	5.033975	-0.196141	-1.170638
19	1	0	-1.057626	-2.574712	0.997141
20	1	0	-1.637864	-1.167137	1.885372
21	1	0	-1.103849	-1.293078	-1.121565
22	1	0	-1.651543	1.094577	-1.179029
23	1	0	-1.996425	1.091068	0.558457
24	1	0	-4.041765	-0.227710	1.325833
25	1	0	-4.982010	-1.048168	0.066849
26	1	0	-3.560526	-2.630901	1.399044
27	1	0	-3.159244	-2.648213	-0.323535
28	1	0	-3.760527	1.563686	-2.036776
29	1	0	-5.173782	0.655893	-1.511517
30	1	0	-3.687473	2.751998	0.193977
31	1	0	-5.235187	2.971628	-0.633124

32	1	0	-5.113872	1.839757	0.720355
33	1	0	1.116114	-1.188964	-1.676621
34	1	0	-0.126197	0.656167	2.002080
35	1	0	2.180748	1.344768	2.462561
36	1	0	4.584287	1.410115	1.805517
37	1	0	3.498515	-1.134080	-2.335224
38	1	0	6.371600	0.822981	0.199926
39	1	0	5.826904	-0.449147	-1.869837

Conformer 6:

Energy = -714.965368 Hartree

Standard orientation:

Center Number	Atomic Number	Atomic Type	Coordinates (Angstroms) X	Y	Z
1	6	0	-1.601889	-1.297221	1.401721
2	6	0	-1.171079	-0.986181	-0.040667
3	6	0	-1.962051	0.227585	-0.562499
4	7	0	-3.393677	-0.013410	-0.476586
5	6	0	-3.825887	-0.234745	0.896840
6	6	0	-3.120196	-1.454213	1.485779
7	6	0	-4.201124	0.957672	-1.207245
8	6	0	-4.172972	2.410422	-0.715164
9	6	0	1.005494	0.185945	0.517560
10	6	0	0.321786	-0.783185	-0.192265
11	6	0	3.123312	-0.441255	-0.557381
12	6	0	2.401045	0.384483	0.360617
13	6	0	1.054987	-1.593254	-1.100625
14	6	0	2.408835	-1.431464	-1.278389
15	6	0	4.520035	-0.242150	-0.712889
16	6	0	3.108598	1.378952	1.087278
17	6	0	5.176423	0.730132	0.005947
18	6	0	4.462978	1.548266	0.914722
19	1	0	-1.285487	-0.476259	2.061122
20	1	0	-1.095109	-2.202163	1.759072
21	1	0	-1.462436	-1.841580	-0.665747

22	1	0	-1.702889	0.410384	-1.613122
23	1	0	-1.646763	1.124892	0.006912
24	1	0	-3.626394	0.642041	1.545932
25	1	0	-4.912154	-0.390069	0.894845
26	1	0	-3.430780	-2.348180	0.928738
27	1	0	-3.433206	-1.593541	2.528072
28	1	0	-3.875972	0.928423	-2.256154
29	1	0	-5.237876	0.594788	-1.192770
30	1	0	-3.163858	2.835189	-0.757448
31	1	0	-4.819586	3.030171	-1.347240
32	1	0	-4.534798	2.498577	0.315287
33	1	0	0.476215	0.825003	1.222121
34	1	0	0.525774	-2.358693	-1.664612
35	1	0	2.948885	-2.065783	-1.978241
36	1	0	5.064459	-0.873223	-1.412189
37	1	0	2.559947	2.007661	1.785477
38	1	0	6.246262	0.874036	-0.121340
39	1	0	4.991731	2.313462	1.477345

Conformer 7:

Energy = -714.964354 Hartree

Standard orientation:

Center Number	Atomic Number	Atomic Type	Coordinates (Angstroms) X	Y	Z
1	6	0	-1.868782	-2.166369	0.715907
2	6	0	-1.271658	-1.598614	-0.589339
3	6	0	-2.222008	-0.538088	-1.161829
4	7	0	-2.577776	0.511996	-0.209492
5	6	0	-3.196600	-0.054296	0.985553
6	6	0	-2.261216	-1.045351	1.672966
7	6	0	-3.422038	1.514299	-0.855429
8	6	0	-3.611137	2.787233	-0.037587
9	6	0	0.555878	0.149477	-0.192650
10	6	0	0.171429	-1.157572	-0.415209
11	6	0	2.925874	-0.506679	-0.103277

12	6	0	1.921210	0.508256	-0.035191
13	6	0	1.184436	-2.156044	-0.480634
14	6	0	2.513942	-1.845911	-0.329725
15	6	0	4.288053	-0.142599	0.054105
16	6	0	2.323256	1.851280	0.190487
17	6	0	4.645312	1.168797	0.271554
18	6	0	3.652657	2.174959	0.340711
19	1	0	-1.161908	-2.859585	1.187751
20	1	0	-2.765478	-2.749200	0.459819
21	1	0	-1.256525	-2.416978	-1.322949
22	1	0	-3.132050	-1.062238	-1.523638
23	1	0	-1.759829	-0.063292	-2.036137
24	1	0	-3.443126	0.758611	1.675769
25	1	0	-4.151222	-0.564412	0.731997
26	1	0	-1.365959	-0.513340	2.019119
27	1	0	-2.757068	-1.456971	2.560657
28	1	0	-2.938362	1.777845	-1.804332
29	1	0	-4.412698	1.089759	-1.118712
30	1	0	-2.641905	3.205842	0.255142
31	1	0	-4.140576	3.536051	-0.637045
32	1	0	-4.200954	2.622294	0.869811
33	1	0	-0.206868	0.921499	-0.127607
34	1	0	0.892457	-3.188988	-0.662743
35	1	0	3.269820	-2.626317	-0.390285
36	1	0	5.048164	-0.919344	0.000107
37	1	0	1.559228	2.624108	0.242873
38	1	0	5.692065	1.436417	0.391323
39	1	0	3.945583	3.207583	0.513051

Conformer 8:

Energy = -714.964263 Hartree

Standard orientation:

Center Number	Atomic Number	Atomic Type	Coordinates (Angstroms) X	Y	Z
1	6	0	-1.953387	-2.200444	0.517927

2	6	0	-1.409617	-1.406129	-0.688304
3	6	0	-2.347605	-0.227538	-0.989266
4	7	0	-2.613794	0.625601	0.167077
5	6	0	-3.174297	-0.148989	1.272185
6	6	0	-2.236342	-1.274033	1.696147
7	6	0	-3.490383	1.744240	-0.172632
8	6	0	-2.853128	2.782495	-1.089625
9	6	0	0.505320	0.178770	-0.078288
10	6	0	0.057405	-1.050442	-0.518424
11	6	0	2.847382	-0.560813	-0.230243
12	6	0	1.890041	0.456554	0.073803
13	6	0	1.023853	-2.051771	-0.819628
14	6	0	2.370447	-1.819520	-0.680647
15	6	0	4.229003	-0.278736	-0.074066
16	6	0	2.357104	1.719535	0.524148
17	6	0	4.649711	0.956065	0.365294
18	6	0	3.704036	1.964477	0.667192
19	1	0	-1.252464	-2.995645	0.799071
20	1	0	-2.889947	-2.690808	0.215054
21	1	0	-1.467160	-2.065455	-1.565865
22	1	0	-3.297046	-0.644707	-1.387774
23	1	0	-1.909257	0.383194	-1.784973
24	1	0	-3.351254	0.534268	2.111926
25	1	0	-4.159955	-0.582058	0.996452
26	1	0	-2.690147	-1.833466	2.523361
27	1	0	-1.299823	-0.841826	2.070787
28	1	0	-4.437843	1.383034	-0.623079
29	1	0	-3.765417	2.233676	0.770052
30	1	0	-2.645254	2.390812	-2.090575
31	1	0	-3.531582	3.634557	-1.208872
32	1	0	-1.913235	3.150936	-0.664235
33	1	0	-0.221086	0.949086	0.168599
34	1	0	0.681145	-3.022304	-1.174608
35	1	0	3.089683	-2.599550	-0.922116
36	1	0	4.952912	-1.056846	-0.307481
37	1	0	1.629131	2.494160	0.755984
38	1	0	5.710835	1.161131	0.482038
39	1	0	4.047062	2.936279	1.013269

Conformer 9:

Energy = -714.963795 Hartree

Standard orientation:

Center Number	Atomic Number	Atomic Type	Coordinates (Angstroms) X	Y	Z
1	6	0	-1.535719	-2.138844	0.635651
2	6	0	-1.059243	-1.358525	-0.608463
3	6	0	-2.240488	-0.568852	-1.195653
4	7	0	-2.928191	0.277185	-0.222481
5	6	0	-3.424326	-0.512262	0.900901
6	6	0	-2.282915	-1.234337	1.610507
7	6	0	-3.983276	1.048634	-0.875157
8	6	0	-4.546343	2.176531	-0.017393
9	6	0	1.431496	-1.137628	-0.507296
10	6	0	0.194939	-0.542318	-0.340239
11	6	0	2.584642	0.912857	0.187345
12	6	0	2.642845	-0.447389	-0.250844
13	6	0	0.154029	0.812154	0.091738
14	6	0	1.310270	1.514017	0.344305
15	6	0	3.796227	1.606790	0.443254
16	6	0	3.913994	-1.058865	-0.418002
17	6	0	5.012308	0.985730	0.274107
18	6	0	5.071125	-0.360407	-0.160731
19	1	0	-2.215505	-2.935307	0.299037
20	1	0	-0.684072	-2.629152	1.121926
21	1	0	-0.784803	-2.101340	-1.369506
22	1	0	-2.944794	-1.300721	-1.644904
23	1	0	-1.885262	0.071081	-2.012970
24	1	0	-4.175282	-1.257422	0.559544
25	1	0	-3.933259	0.154159	1.604210
26	1	0	-1.599324	-0.491869	2.041620
27	1	0	-2.687295	-1.822651	2.443385
28	1	0	-3.548209	1.485502	-1.782782
29	1	0	-4.808447	0.388642	-1.212900
30	1	0	-3.740996	2.828401	0.338586
31	1	0	-5.240356	2.781719	-0.611130

32	1	0	-5.099179	1.808967	0.853160
33	1	0	1.490412	-2.171083	-0.847804
34	1	0	-0.817229	1.282280	0.215634
35	1	0	1.257308	2.550722	0.671420
36	1	0	3.747206	2.641527	0.776271
37	1	0	3.957730	-2.093403	-0.752233
38	1	0	5.933766	1.526864	0.473454
39	1	0	6.037543	-0.840916	-0.290836

----

13. Substrate synthesis and characterisation

13.1. General

Commercially available chemicals and reagents were purchased from Sigma-Aldrich (Poole, Dorset, UK), Prozomix (Haltwhistle, Northumberland, UK), Alfa Aesar (Karlsruhe, Germany), Acros Organics (Geel, Belgium) or Fluorochem (Hadfield, Derbyshire, UK) unless stated otherwise. HPLC solvents were obtained from Sigma-Aldrich (Poole, Dorset, UK) or ROMIL (Waterbeach, Cambridge, UK) and GC gases from BOC gases (Guildford, UK). Many pyridine compounds were commercially available including compounds such as 3-methylpyridine, 3-phenylpyridine, 3-(4-methoxyphenyl)pyridine, 3-(thiophen-2-yl)pyridine, 4-methylpyridine, 4-phenylpyridine, 3-(4-bromophenyl)pyridine, 4-(pyridin-3-yl)aniline, 3,4-dimethylpyridine, 5,6,7,8-tetrahydroisoquinoline and 4-methyl-3-phenylpyridine were purchased from Sigma-Aldrich (Poole, Dorset, UK) or Fluorochem (Hadfield, Derbyshire, UK). Alkylating agents such as iodoethane, 1-iodopropane, iodomethane, allyl bromide, propargyl bromide and benzyl bromide were purchased from Sigma-Aldrich (Poole, Dorset, UK) or Fluorochem (Hadfield, Derbyshire, UK).

13.1.1. General Method 2: Suzuki–Miyaura Coupling for the preparation of pyridines

Based on the procedure of Yang[15], under nitrogen, a round bottom flask was sequentially charged with $Pd(OAc)_2$ (79 mg, 0.35 mmol), XPhos (210 mg, 0.44 mmol), 3-chloropyridine (2.00 g, 17.61 mmol, 1 equiv.), boronic acid (21.14 mmol, 1.2 equiv.), and 100 mL of degassed *n*-butanol. The mixture was prestirred at room temperature for 15 min, and then a solution of $CsOH{\cdot}H_2O$ (5.03 g, 29.94 mmol) in 25 mL of degassed H_2O was added in one portion to initiate the Suzuki reaction. The reaction mixture was stirred vigorously at room temperature until all the 3-chloropyridine was consumed (monitored by GC-MS). The mixture was concentrated *in vacuo* and the aqueous phase was extracted with DCM (25 mL). The organic phase was extracted with 2 M aqueous HCl (3 x 25 mL) and the combined aqueous phases were basified to pH 12 by addition of 5 M NaOH. The product was extracted into DCM (2 x 25 mL). The organic phase was dried over anhydrous $MgSO_4$ and concentrated *in vacuo* to afford the corresponding pyridine.

3-(3-methoxyphenyl)pyridine

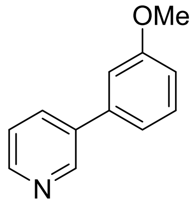

Following General Method 2 with (3-methoxyphenyl)boronic acid (3.21 g, 21.14 mmol) and subjecting the crude product to acid and base wash afforded the title compound as a yellow oil product (3.00 g, 16.20 mmol, 92%). **^{1}H NMR** (400 MHz, $CDCl_3$): δ 8.84 (dd, J = 2.4, 0.9 Hz, 1H), 8.58 (dd, J = 4.8, 1.6 Hz, 1H), 7.85 (ddd, J = 7.8, 2.4, 1.6 Hz, 1H), 7.41 – 7.31 (m, 2H), 7.16 (ddd, J = 7.8, 1.7, 1.0 Hz, 1H), 7.10 (dd, J = 2.6, 1.3 Hz, 1H), 6.94 (ddd, J = 8.3, 2.6, 1.3 Hz, 1H), 3.86 (s, 3H); **^{13}C NMR** (100 MHz, $CDCl_3$): δ 160.2, 148.7, 148.4, 139.4, 136.6, 134.5, 130.2, 123.6, 119.7, 113.5, 113.0, 55.4; **HRMS** calcd. for $C_{12}H_{12}NO^+$ 186.0913 $[M+H]^+$, found 186.0921.

3-(3-(methylsulfonyl)phenyl)pyridine

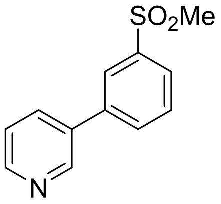

Following General Method 2 with (3-(methylsulfonyl)phenyl)boronic acid (3.84 g, 19.20 mmol) and subjecting the crude product to acid and base wash afforded the title compound as a white solid product (2.49 g, 10.67 mmol, 61%). **^{1}H NMR** (400 MHz, $CDCl_3$): 8.87 – 8.82 (m, 1H), 8.64 (dd, J = 4.9, 1.5 Hz, 1H), 8.16 – 8.11 (m, 1H), 8.00 – 7.82 (m, 3H), 7.68 (t, J = 7.8 Hz, 1H), 7.40 (dd, J = 8.0, 4.9 Hz, 1H), 3.10 (s, 3H); **^{13}C NMR** (100 MHz, $CDCl_3$): δ 149.6, 148.3, 141.7, 139.5, 134.8, 134.6, 132.3, 130.4, 126.9, 126.0, 123.9, 44.6; **HRMS** calcd. for $C_{12}H_{12}NO_2S^+$ 234.0583 $[M+H]^+$, found 234.0584.

3-(4-fluorophenyl)pyridine

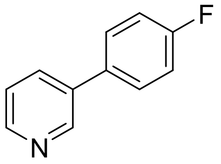

Following General Method 2 with (4-fluorophenyl)boronic acid (2.96 g, 21.14 mmol) and subjecting the crude product to acid and base wash afforded the title compound as a yellow oil product (2.99 g,

17.26 mmol, 98%). **^{1}H NMR** (400 MHz, $CDCl_3$): δ 8.79 (dd, *J* = 2.4, 0.9 Hz, 1H), 8.57 (dd, *J* = 4.8, 1.6 Hz, 1H), 7.80 (ddd, *J* = 7.9, 2.4, 1.7 Hz, 1H), 7.57 – 7.47 (m, 2H), 7.34 (ddd, *J* = 7.9, 4.8, 0.9 Hz, 1H), 7.20 – 7.11 (m, 2H); **^{13}C NMR** (100 MHz, $CDCl_3$): δ 164.6, 162.1, 148.9, 148.6, 136.1, 134.6, 134.4, 129.2, 124.0, 116.6, 116.4; **HRMS** calcd. for $C_{11}H_9FN^+$ 174.0714 $[M+H]^+$, found 174.0718.

3-(naphthalen-2-yl)pyridine

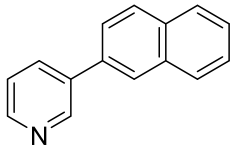

Following General Method 2 with naphthalen-2-ylboronic acid (3.64 g, 21.14 mmol) and subjecting the crude product to acid and base wash afforded the title compound as a cream coloured solid product (2.71 g, 13.18 mmol, 75%). **^{1}H NMR** (400 MHz, $CDCl_3$): δ 8.99 (dd, *J* = 2.4, 0.9 Hz, 1H), 8.63 (dd, *J* = 4.8, 1.8 Hz, 1H), 8.08 – 7.84 (m, 5H), 7.72 (dd, *J* = 8.5, 1.8 Hz, 1H), 7.59 – 7.46 (m, 2H), 7.41 (ddd, *J* = 7.9, 4.8, 0.9 Hz, 1H); **^{13}C NMR** (100 MHz, $CDCl_3$): δ 148.7, 148.7, 136.7, 135.3, 134.7, 133.7, 133.0, 129.0, 128.4, 127.9, 126.8, 126.6, 126.3, 125.2, 123.8; **HRMS** calcd. for $C_{15}H_{12}N^+$ 206.0964 $[M+H]^+$, found 206.0956.

3-(furan-2-yl)pyridine

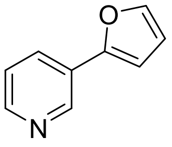

Following General Method 2 with furan-2-ylboronic acid (2.40 g, 21.14 mmol) and subjecting the crude product to acid and base wash afforded the title compound as a yellow oil product (1.96 g, 13.50 mmol, 77%). **^{1}H NMR** (400 MHz, $CDCl_3$): δ 8.91 (d, *J* = 2.3 Hz, 1H), 8.46 (dd, *J* = 4.9, 1.7 Hz, 1H), 7.90 (dt, *J* = 7.9, 2.0 Hz, 1H), 7.49 (d, *J* = 1.7 Hz, 1H), 7.27 (ddd, *J* = 8.1, 4.8, 1.0 Hz, 1H), 6.72 (d, *J* = 3.4 Hz, 1H), 6.48 (dd, *J* = 3.4, 1.8 Hz, 1H); **^{13}C NMR** (100 MHz, $CDCl_3$): δ 151.1, 148.3, 145.5, 143.1, 130.8, 126.9, 123.6, 111.9, 106.5; **HRMS** calcd. for $C_9H_8NO^+$ 146.0600 $[M+H]^+$, found 146.0604[15].

3-methyl-4-phenylpyridine

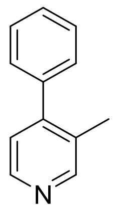

Following General Method 2 with 4-bromo-3-methylpyridine (2.30 g, 13.37 mmol) and phenylboronic acid (3.26 g, 26.74 mmol) and subjecting the crude product to acid and base wash. The title compound was isolated as a mixture (1.96 g, 11.58 mmol, 87%). **^{1}H NMR** (400 MHz, $CDCl_3$): δ 8.44 (s, 1H), 8.41 (d, *J* = 5.0 Hz, 1H), 7.48 – 7.34 (m, 3H), 7.31 – 7.25 (m, 2H), 7.13 (d, *J* = 5.0 Hz, 1H), 2.25 (s, 3H); **^{13}C NMR** (100 MHz, $CDCl_3$): δ 151.0, 150.0, 149.5, 147.1, 146.7, 139.0, 130.8, 128.6, 128.5, 128.1, 124.2, 123.3, 17.3; **HRMS** calcd. for $C_{12}H_{12}N^+$ 170.0964 $[M+H]^+$, found 170.0958.

***N*-(4-(pyridin-3-yl)phenyl)acetamide**

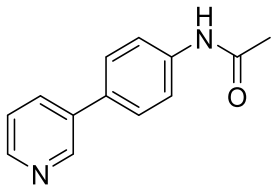

To a mixture of commercially available 4-(pyridin-3-yl)aniline (2.00 g, 11.75 mmol) and triethylamine (3.57g, 35.25 mmol) in THF (200 mL) was added acetyl chloride (1.84 g, 23.50 mmol) at room temperature. The reaction mixture was stirred vigorously at room temperature until all the 4-(pyridin-3-yl)aniline was consumed (monitored by GC-MS). Acetamide was removed *in vacuo* to yield crude product which was carried forward without further purification (2.23 g, 10.51 mmol, 89%). **^{1}H NMR** (400 MHz, $CDCl_3$): δ 8.98 (s, 1H), 8.78 – 8.73 (m, 1H), 8.50 (dd, *J* = 4.8, 1.5 Hz, 1H), 7.82 (dt, *J* = 8.2, 2.0 Hz, 1H), 7.75 (d, *J* = 8.2 Hz, 2H), 7.46 (d, *J* = 8.2 Hz, 2H), 7.32 (dd, *J* = 8.2, 4.9 Hz, 1H), 2.21 (s, 3H); **^{13}C NMR** (100 MHz, $CDCl_3$): δ 174.2, 169.3, 147.7, 147.6, 139.0, 136.4, 134.3, 132.8, 127.4, 123.8, 120.5, 24.6; **HRMS** calcd. for $C_{13}H_{12}N_2O^+$ 212.0950 $[M+H]^+$, found 212.0948.

13.1.2. General Method 3: *N*-alkylation for the preparation of pyridinium salts (1a-21a)

Based on the procedure of Baldwin[16], to a stirred solution of pyridine (1 equiv.) in acetone (0.62 ml/mmol of pyridine) was added alkyl or allyl halide (1 equiv.) and the reaction mixture was heated at reflux for 5 h under nitrogen. Acetone was removed *in vacuo* to yield crude product. The crude was

triturated with diethyl ether (3 x 30 mL) followed by concentration *in vacuo* to afford the corresponding pyridinium salt.

3-(3-methoxyphenyl)-1-propylpyridinium iodide (1a)

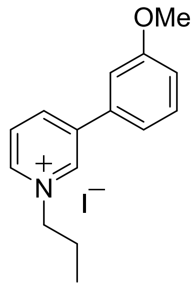

Following General Method 3 with 3-(3-methoxyphenyl)pyridine (1.54 g, 8.31 mmol) and 1-iodopropane (1.41 g, 8.31 mmol) afforded a viscous brown oil crude which was carried forward without trituration (2.56 g, 7.21 mmol, 87%). **^{1}H NMR** (500 MHz, $CDCl_3$): δ 9.59 (t, *J* = 1.7 Hz, 1H), 9.28 (d, *J* = 6.0 Hz, 1H), 8.64 (dt, *J* = 8.3, 1.5 Hz, 1H), 8.15 (dd, *J* = 8.3, 6.0 Hz, 1H), 7.43 – 7.34 (m, 3H), 6.99 (dt, *J* = 7.5, 2.3 Hz, 1H), 5.05 (t, *J* = 7.5 Hz, 2H), 3.90 (s, 3H), 2.09 (p, *J* = 7.5 Hz, 2H), 1.00 (t, *J* = 7.5 Hz, 3H); **^{13}C NMR** (126 MHz, $CDCl_3$): δ 160.7, 142.9, 142.4, 141.2, 133.9, 130.9, 128.5, 120.0, 117.1, 112.7, 63.3, 56.5, 25.5, 10.5; **HRMS** calcd. for $C_{15}H_{18}NO^+$ 228.1383 $[M-I]^+$, found 228.1382.

3-(3-(methylsulfonyl)phenyl)-1-propylpyridinium iodide (2a)

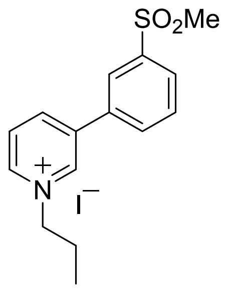

Following General Method 3 with 3-(3-(methylsulfonyl)phenyl)pyridine (3.37 g, 14.45 mmol) and iodoethane (2.25 g, 14.45 mmol) afforded the title compound as a hygroscopic brown powder (3.57 g, 8.85 mmol, 61%). **^{1}H NMR** (400 MHz, DMSO-d6) δ 9.64 (s, 1H), 9.17 (d, *J* = 6.0 Hz, 1H), 9.04 (dd, *J* = 8.1, 1.8 Hz, 1H), 8.44 (d, *J* = 2.0 Hz, 1H), 8.35 – 8.25 (m, 2H), 8.11 (dd, *J* = 7.4, 1.7 Hz, 1H), 7.91 (t, *J* = 7.8 Hz, 1H), 4.69 (t, *J* = 7.4 Hz, 2H), 3.35 (s, 3H), 2.05 (h, *J* = 7.4 Hz, 2H), 0.94 (t, *J* = 7.4 Hz, 3H);

^{13}C NMR (100 MHz, DMSO-d6) δ 143.6, 143.4, 143.3, 142.0, 138.1, 134.5, 132.7, 130.6, 128.3, 128.1, 126.1, 62.4, 43.5, 24.2, 10.3; **HRMS** calcd. for $C_{15}H_{18}NO_2S^+$ 276.1053 [M-I]$^+$, found 276.1043.

1-allyl-3-(4-bromophenyl)pyridinium bromide (3a)

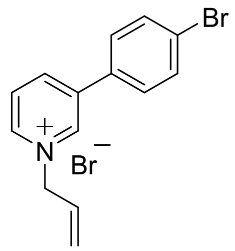

Following General Method 3 with commercially available 3-(4-bromophenyl)pyridine (1.00 g, 4.27 mmol) and allyl bromide (0.52 g, 4.272 mmol) afforded the title compound as a hygroscopic cream coloured powder (1.37 g, 3.86 mmol, 90%). **^{1}H NMR** (500 MHz, CD_3OD): δ 9.39 (s, 1H), 8.98 (d, *J* = 6.0 Hz, 1H), 8.90 (dd, *J* = 8.2, 1.8 Hz, 1H), 8.21 (dd, *J* = 8.2, 6.3 Hz, 1H), 7.84 – 7.74 (m, 4H), 6.27 (ddt, *J* = 16.8, 10.2, 6.3 Hz, 1H), 5.62 (d, *J* = 17.0 Hz, 1H), 5.58 (d, *J* = 10.2 Hz, 1H), 5.40 (d, *J* = 6.3 Hz, 2H); **^{13}C NMR** (126 MHz, CD_3OD): δ 144.6, 144.1, 141.7, 133.9, 133.7, 131.9, 130.5, 129.6, 126.0, 123.9, 64.9; **HRMS** calcd. for $C_{14}H_{13}BrN^+$ 274.0226 [M-Br]$^+$, found 274.0234.

3-(4-acetamidophenyl)-1-allylpyridinium bromide (4a)

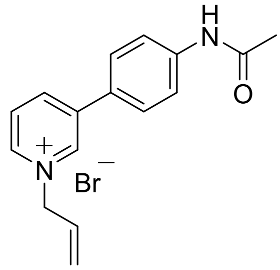

Following General Method 3 with *N*-(4-(pyridin-3-yl)phenyl)acetamide (1.83 g, 8.62 mmol) and allyl bromide (1.04 g, 8.62 mmol) afforded hygroscopic a pale-brown powder crude which was carried forward without further purification (2.59 g, 7.77 mmol, 90%). **^{1}H NMR** (400 MHz, CD_3OD): δ 9.33 (s, 1H), 8.92 – 8.83 (m, 2H), 8.20 – 8.12 (m, 1H), 7.83 (s, 4H), 6.26 (ddt, *J* = 16.8, 10.1, 6.4 Hz, 1H), 5.65 – 5.54 (m, 2H), 5.36 (d, *J* = 6.4 Hz, 2H), 2.18 (s, 3H); **^{13}C NMR** (100 MHz, CD_3OD): δ 172.0, 144.0, 143.6, 143.3, 142.4, 142.4, 132.0, 129.5, 129.5, 129.2, 123.7, 121.6, 64.8, 24.0; **HRMS** calcd. for $C_{16}H_{17}N_2O^+$ 253.1335 [M-Br]$^+$, found 253.1326.

1-ethyl-3-phenylpyridinium iodide (5a)

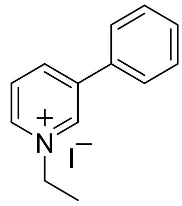

Following General Method 3 with commercially available 3-phenylpyridine (2.00 g, 6.43 mmol) and iodoethane (1.00 g, 6.43 mmol) afforded the title compound as a hygroscopic pale-yellow powder (1.84 g, 5.92 mmol, 92%). ^{1}**H NMR** (400 MHz, $(CD_3)_2SO$): δ 9.52 (t, *J* = 1.6 Hz, 1H), 9.17 – 9.08 (m, 1H), 8.96 – 8.89 (m, 1H), 8.29 – 8.20 (m, 1H), 7.98 – 7.90 (m, 2H), 7.68 – 7.53 (m, 3H), 4.74 (q, *J* = 7.3 Hz, 2H), 1.62 (t, *J* = 7.3 Hz, 3H); ^{13}**C NMR** (100 MHz, $(CD_3)_2SO$): δ 142.7, 142.5, 139.5, 133.1, 130.1, 129.4, 128.0, 127.5, 56.6, 16.4; **HRMS** calcd. for $C_{13}H_{14}N^+$ 184.1121 $[M-I]^+$, found 184.1130.

1-ethyl-3-(4-fluorophenyl)pyridinium iodide (6a)

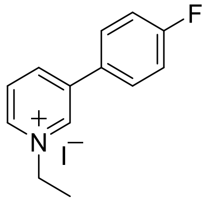

Following General Method 3 with 3-(4-fluorophenyl)pyridine (2.00 g, 11.55 mmol) and iodoethane (1.80 g, 11.55 mmol) afforded the title compound as a hygroscopic cream coloured powder (3.61 g, 10.97 mmol, 95%). ^{1}**H NMR** (400 MHz, $CDCl_3$): δ 9.65 (t, *J* = 1.7 Hz, 1H), 9.33 – 9.26 (m, 1H), 8.64 (dt, *J* = 8.4, 1.6 Hz, 1H), 8.17 (dd, *J* = 8.4, 6.0 Hz, 1H), 8.00 – 7.91 (m, 2H), 7.19 (t, *J* = 8.4 Hz, 2H), 5.13 (q, *J* = 7.3 Hz, 2H), 1.71 (t, *J* = 7.3 Hz, 3H); ^{13}**C NMR** (100 MHz, $CDCl_3$): δ 165.6, 142.7, 142.4, 142.2, 140.6, 130.3, 130.2, 128.8, 117.2, 117.0, 57.8, 17.5; **HRMS** calcd. for $C_{13}H_{13}FN^+$ 202.1027 $[M-I]^+$, found 202.1034.

1-ethyl-3-(4-methoxyphenyl)pyridinium iodide (7a)

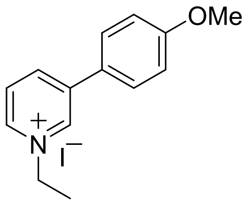

Following General Method 3 with commercially available 3-(4-methoxyphenyl)pyridine (1.00 g, 5.40 mmol) and iodoethane (0.84 g, 5.40 mmol) afforded the title compound as a hygroscopic pale-yellow powder (1.76 g, 5.16 mmol, 95%). **^{1}H NMR** (400 MHz, $CDCl_3$): δ 9.56 (t, *J* = 1.7 Hz, 1H), 9.18 (dt, *J* = 6.2, 1.3 Hz, 1H), 8.58 (dt, *J* = 8.4, 1.4 Hz, 1H), 8.10 (dd, *J* = 8.4, 6.0 Hz, 1H), 7.91 – 7.83 (m, 2H), 7.05 – 6.97 (m, 2H), 5.10 (q, *J* = 7.3 Hz, 2H), 3.81 (s, 3H), 1.70 (t, *J* = 7.3 Hz, 3H); **^{13}C NMR** (100 MHz, $CDCl_3$): δ 161.8, 141.8, 141.2, 129.3, 128.6, 124.6, 115.4, 57.7, 55.7, 17.6; **HRMS** calcd. for $C_{14}H_{16}NO^+$ 214.1226 [M-I]$^+$, found 214.1234.

1-ethyl-3-(furan-2-yl)pyridinium iodide (8a)

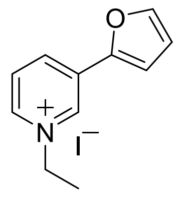

Following General Method 3 with 3-(furan-2-yl)pyridine (1.00 g, 6.89 mmol) and iodoethane (1.07 g, 6.89 mmol) afforded the title compound as a hygroscopic orange powder (1.77 g, 5.88 mmol, 85%). **^{1}H NMR** (400 MHz, $CDCl_3$): δ 9.85 (d, *J* = 1.8 Hz, 1H), 9.19 (dt, *J* = 6.1, 1.3 Hz, 1H), 8.61 (dt, *J* = 8.3, 1.5 Hz, 1H), 8.10 (dd, *J* = 8.3, 6.0 Hz, 1H), 7.62 (dd, *J* = 13.2, 2.7 Hz, 2H), 6.57 (dd, *J* = 3.6, 1.8 Hz, 1H), 5.10 (q, *J* = 7.3 Hz, 2H), 1.77 – 1.68 (m, 3H); **^{13}C NMR** (100 MHz, $CDCl_3$): δ 146.2, 145.7, 141.5, 139.3, 137.9, 131.7, 128.8, 113.9, 113.4, 57.7, 17.6; **HRMS** calcd. for $C_{11}H_{12}NO^+$ 174.0913 [M-I]$^+$, found 174.0918.

1-ethyl-3-(thiophen-2-yl)pyridinium iodide (9a)

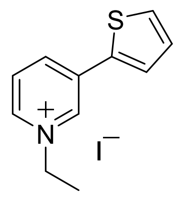

Following General Method 3 with commercially available 3-(thiophen-2-yl)pyridine (1.00 g, 6.20 mmol) and iodoethane (0.97 g, 6.89 mmol) afforded the title compound as a hygroscopic pale-yellow powder (1.59 g, 5.01 mmol, 81%). **^{1}H NMR** (400 MHz, $CDCl_3$): δ 9.80 (s, 1H), 9.18 (d, *J* = 6.0 Hz, 1H), 8.51 (d, *J* = 8.2 Hz, 1H), 8.12 – 8.02 (m, 2H), 7.51 (d, *J* = 5.1 Hz, 1H), 7.14 (dd, *J* = 5.1, 3.8 Hz, 1H), 5.11

(q, J = 7.3 Hz, 2H), 1.71 (t, J = 7.3 Hz, 3H); **^{13}C NMR** (100 MHz, $CDCl_3$): δ 141.7, 140.5, 135.6, 134.8, 129.9, 129.7, 129.6, 128.8, 57.6, 17.6; **HRMS** calcd. for $C_{11}H_{12}NS^+$ 190.0685 [M-I]$^+$, found 190.0689.

1-ethyl-3-(naphthalen-2-yl)pyridinium iodide (10a)

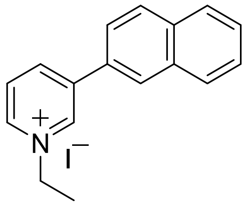

Following General Method 3 with 3-(naphthalen-2-yl)pyridine (1.00 g, 4.87 mmol) and iodoethane (0.76 g, 4.87 mmol) afforded the title compound as a hygroscopic pale-yellow powder (1.06 g, 4.43 mmol, 60%). **^{1}H NMR** (400 MHz, $(CD_3)_2SO$): δ 9.65 (t, J = 1.4 Hz, 1H), 9.12 (dt, J = 6.0, 1.4 Hz, 1H), 9.07 (dt, J = 8.4, 1.4 Hz, 1H), 8.56 (d, J = 1.9 Hz, 1H), 8.29 (dd, J = 8.4, 6.0 Hz, 1H), 8.18 (d, J = 8.4 Hz, 1H), 8.11 – 8.00 (m, 3H), 7.70 – 7.61 (m, 2H), 4.74 (q, J = 7.3 Hz, 2H), 1.65 (t, J = 7.3 Hz, 3H); **^{13}C NMR** (100 MHz, $(CD_3)_2SO$): δ 143.0, 142.7, 142.6, 139.5, 133.3, 132.9, 130.5, 129.2, 128.5, 128.1, 127.8, 127.7, 127.4, 127.2, 124.4, 56.8, 16.5; **HRMS** calcd. for $C_{17}H_{16}N^+$ 234.1277 [M-I]$^+$, found 234.1268.

1-methyl-3-phenylpyridinium iodide (11a)

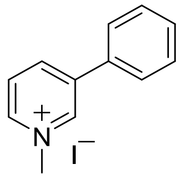

Following General Method 3 with commercially available 3-phenylpyridine (1.00 g, 6.44 mmol) and iodomethane (0.91 g, 6.44 mmol) afforded the title compound as a hygroscopic pale-yellow powder (1.86 g, 6.26 mmol, 97%). **^{1}H NMR** (400 MHz, $CDCl_3$): 9.39 (t, J = 1.7 Hz, 1H), 9.18 (dd, J = 6.0, 1.4 Hz, 1H), 8.58 (dt, J = 8.3, 1.5 Hz, 1H), 8.12 (dd, J = 8.3, 6.0 Hz, 1H), 7.90 – 7.80 (m, 2H), 7.56 – 7.44 (m, 3H), 4.74 (s, 3H); **^{13}C NMR** (100 MHz, $CDCl_3$): δ 143.5, 143.3, 142.8, 141.3, 132.7, 130.8, 130.0, 128.4, 127.9, 50.0; **HRMS** calcd. for $C_{12}H_{12}N^+$ 170.0965 [M-I]$^+$, found 170.0962.

3-phenyl-1-(prop-2-yn-1-yl)pyridinium bromide (12a)

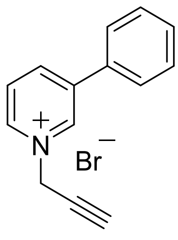

Following General Method 3 with commercially available 3-phenylpyridine (2.00 g, 12.89 mmol) and 3-bromopropyne (1.53 g, 12.89 mmol) afforded the title compound as a hygroscopic brown powder (1.49 g, 11.86 mmol, 92%). **^{1}H NMR** (400 MHz, CD_3OD): δ 9.43 (t, *J* = 1.8 Hz, 1H), 9.13 (dt, *J* = 6.1, 1.4 Hz, 1H), 8.94 (dt, *J* = 8.3, 1.5 Hz, 1H), 8.24 (dd, *J* = 8.3, 6.1 Hz, 1H), 7.90 – 7.80 (m, 2H), 7.67 – 7.55 (m, 3H), 5.70 (d, *J* = 2.6 Hz, 2H), 3.59 (t, *J* = 2.6 Hz, 1H); **^{13}C NMR** (100 MHz, CD_3OD): δ 145.3, 143.8, 143.5, 142.9, 134.5, 131.7, 130.9, 129.6, 128.6, 81.6, 75.1, 51.7; **HRMS** calcd. for $C_{14}H_{12}N^+$ 194.0964 $[M\text{-}Br]^+$, found 194.0960.

1-ethyl-3-methylpyridinium iodide (13a)

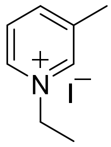

Following General Method 3 with commercially available 3-methylpyridine (4.79 g, 51.38 mmol) and iodoethane (8.01 g, 51.38 mmol) afforded the title compound as a hygroscopic white powder (12.15 g, 48.78 mmol, 95%). **^{1}H NMR** (400 MHz, $CDCl_3$): δ 9.40 (s, 1H), 9.22 (d, *J* = 6.1 Hz, 1H), 8.30 (d, *J* = 8.0 Hz, 1H), 8.02 (dd, *J* = 8.0, 6.1 Hz, 1H), 4.94 (q, *J* = 7.3 Hz, 2H), 2.64 (s, 3H), 1.71 (t, *J* = 7.3 Hz, 3H); **^{13}C NMR** (100 MHz, $CDCl_3$): δ 146.1, 144.3, 141.8, 139.9, 128.1, 57.3, 19.9, 17.4; **HRMS** calcd. for $C_8H_{12}N^+$ 122.0964 $[M\text{-}I]^+$, found 122.0962. Spectroscopic data in accord with previously published reports.[16]

1-benzyl-3-methylpyridinium bromide (14a)

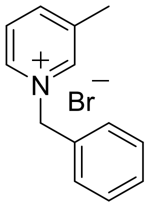

Following General Method 3 with commercially available 3-methylpyridine (4.60 g, 49.39 mmol) and (bromomethyl)benzene (8.45 g, 49.39 mmol) afforded the title compound as a hygroscopic white powder (12.12 g, 45.88 mmol, 93%). **^{1}H NMR** (400 MHz, $(CD_3)_2SO$): δ 9.47 (s, 1H), 9.35 – 9.29 (m, 1H), 8.69 – 8.61 (m, 1H), 8.26 (dd, *J* = 8.0, 6.1 Hz, 1H), 7.81 – 7.71 (m, 2H), 7.64 – 7.52 (m, 3H), 6.05 (s, 2H), 2.65 (s, 3H); **^{13}C NMR** (100 MHz, $(CD_3)_2SO$): δ 146.3, 144.2, 142.0, 139.2, 134.4, 129.3, 129.1, 128.8, 127.7, 62.9, 17.9. Spectroscopic data in accord with previously published reports.[17]

1-ethyl-4-methylpyridinium iodide (15a)

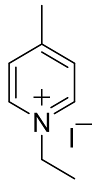

Following General Method 3 with commercially available 4-methylpyridine (4.80 g, 51.54 mmol) and iodoethane (8.04 g, 51.54 mmol) afforded the title compound as a hygroscopic white powder (11.83 g, 47.49 mmol, 92%). **^{1}H NMR** (400 MHz, $CDCl_3$): δ 9.30 – 9.24 (m, 2H), 7.89 (d, *J* = 6.3 Hz, 2H), 4.91 (q, *J* = 7.4 Hz, 2H), 2.66 (s, 3H), 1.68 (t, *J* = 7.4 Hz, 3H); **^{13}C NMR** (100 MHz, $CDCl_3$): δ 159.1, 143.9, 129.1, 56.7, 22.5, 17.3; **HRMS** calcd. for $C_8H_{12}N^+$ 122.0964 $[M-I]^+$, found 122.0970.

1-ethyl-4-phenylpyridinium iodide (16a)

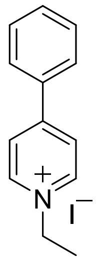

Following General Method 3 with commercially available 4-phenylpyridine (2.50 g, 16.11 mmol) and iodoethane (2.51 g, 16.11 mmol) afforded the title compound as a hygroscopic yellow powder (4.91 g, 15.78 mmol, 98%). **^{1}H NMR** (400 MHz, $(CD_3)_2SO$): δ 9.17 – 9.10 (m, 2H), 8.57 – 8.50 (m, 2H), 8.13 – 8.04 (m, 2H), 7.71 – 7.60 (m, 3H), 4.63 (q, *J* = 7.3 Hz, 2H), 1.57 (t, *J* = 7.3 Hz, 3H); **^{13}C NMR** (100 MHz, $(CD_3)_2SO$): δ 154.6, 144.6, 133.6, 132.1, 129.7, 128.1, 124.5, 55.5, 16.3; **HRMS** calcd. for $C_{13}H_{14}N^+$ 184.1121 $[M-I]^+$, found 184.1114.

1-ethyl-3,4-dimethylpyridinium iodide (17a)

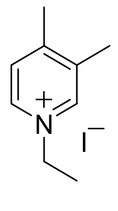

Following General Method 3 with commercially available 3,4-dimethylpyridine (2.50 g, 23.33 mmol) and iodoethane (3.64 g, 23.33 mmol) afforded the title compound as a hygroscopic white powder (5.92 g, 22.50 mmol, 96%). **^{1}H NMR** (400 MHz, $(CD_3)_2SO$): δ 8.96 (s, 1H), 8.85 (dd, *J* = 6.2, 1.6 Hz, 1H), 7.96 (d, *J* = 6.2 Hz, 1H), 4.55 (q, *J* = 7.3 Hz, 2H), 2.53 (s, 3H), 2.40 (s, 3H), 1.52 (t, *J* = 7.3 Hz, 3H); **^{13}C NMR** (100 MHz, $(CD_3)_2SO$): δ 157.5, 142.5, 141.1, 137.5, 127.9, 55.3, 19.6, 16.3, 16.2; **HRMS** calcd. for $C_9H_{14}N^+$ 136.1121 $[M-I]^+$, found 136.1126.

2-ethyl-5,6,7,8-tetrahydroisoquinolinium iodide (18a)

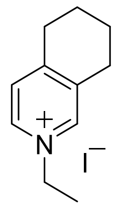

Following General Method 3 with commercially available 5,6,7,8-tetrahydroisoquinoline (1.00 g, 7.51 mmol) and iodoethane (1.17 g, 7.51 mmol) afforded the title compound as a hygroscopic white powder (1.26 g, 4.36 mmol, 58%). **^{1}H NMR** (400 MHz, $CDCl_3$): δ 9.23 (s, 1H), 8.91 (dd, *J* = 6.3, 1.5 Hz, 1H), 7.71 (d, *J* = 6.3 Hz, 1H), 4.85 (q, *J* = 7.3 Hz, 2H), 3.00 (dd, *J* = 21.8, 5.6 Hz, 4H), 1.88 (p, *J* = 3.2 Hz, 4H), 1.68 (t, *J* = 7.3 Hz, 3H); **^{13}C NMR** (100 MHz, $CDCl_3$): δ 158.2, 143.7, 140.1, 139.1, 128.2, 56.5, 29.8, 26.4, 21.1, 21.0, 17.3

1-ethyl-4-methyl-3-phenylpyridinium iodide (19a)

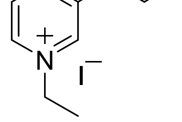

Following General Method 3 with commercially available 4-methyl-3-phenylpyridine (0.52 g, 3.07 mmol) and iodoethane (0.47 g, 3.07 mmol) afforded the title compound as a hygroscopic brown powder (0.87 g, 2.68 mmol, 87%). **^{1}H NMR** (400 MHz, $CDCl_3$): δ 9.37 (d, *J* = 6.4 Hz, 1H), 8.65 (s, 1H), 8.00 (d, *J* = 6.4 Hz, 1H), 7.54 (d, *J* = 6.4 Hz, 3H), 7.48 (dd, *J* = 7.0, 2.0 Hz, 2H), 5.02 (q, *J* = 7.4 Hz, 2H), 2.56 (s, 3H), 1.73 (t, *J* = 7.4 Hz, 3H); **^{13}C NMR** (100 MHz, $CDCl_3$): δ 156.9, 142.5, 142.5, 142.2, 133.0, 129.9, 129.8, 129.3, 129.3, 57.1, 21.4, 17.1; **HRMS** calcd. for $C_{14}H_{16}N^+$ 198.1227 $[M\text{-}I]^+$, found 198.1229.

1-allyl-4-methyl-3-phenylpyridinium bromide (20a)

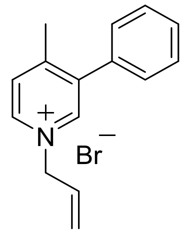

Following General Method 3 with commercially available 4-methyl-3-phenylpyridine (1.00 g, 5.91 mmol) and allyl bromide (0.71 g, 5.91 mmol) afforded the title compound as a hygroscopic pale-brown powder (1.59 g, 5.48 mmol, 93%). **^{1}H NMR** (400 MHz, CD_3OD): δ 8.88 – 8.78 (m, 2H), 8.07 (d, *J* = 6.4 Hz, 1H), 7.64 – 7.55 (m, 3H), 7.55 – 7.47 (m, 2H), 6.22 (ddt, *J* = 16.7, 10.3, 6.4 Hz, 1H), 5.61 – 5.51 (m, 2H), 5.27 (dd, *J* = 6.4, 1.4 Hz, 2H), 2.59 (s, 3H); **^{13}C NMR** (100 MHz, CD_3OD): δ 144.8, 143.5, 143.1, 135.1, 132.0, 130.7, 130.7, 130.4, 130.2, 123.5, 63.8, 21.1; **HRMS** calcd. for $C_{15}H_{16}N^+$ 210.1277 $[M\text{-}Br]^+$, found 210.1268.

1-allyl-3-methyl-4-phenylpyridinium bromide (21a)

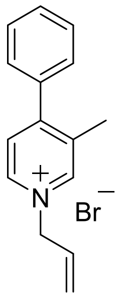

Following General Method 3 with 3-methyl-4-phenylpyridine (1.96 g, 11.59 mmol) and allyl bromide (1.40 g, 11.59 mmol) afforded a viscous brown oil crude which was carried forward without trituration (1.81 g, 6.24 mmol, 54%). **^{1}H NMR** (400 MHz, $CDCl_3$): δ 9.50 (s, 1H), 9.28 (dd, *J* = 6.3, 1.5 Hz, 1H),

7.81 (d, *J* = 6.3 Hz, 1H), 7.59 – 7.49 (m, 3H), 7.42 – 7.33 (m, 2H), 6.20 (ddt, *J* = 16.8, 10.1, 6.7 Hz, 1H), 5.78 – 5.59 (m, 4H), 2.55 (s, 3H); **^{13}C NMR** (100 MHz, $CDCl_3$): δ 145.5, 142.0, 137.1, 135.2, 134.3, 130.6, 130.4, 129.3, 128.5, 128.2, 124.4, 118.4, 62.8, 18.2; **HRMS** calcd. for $C_{15}H_{16}N^+$ 210.1277 [M-Br]$^+$, found 210.1271.

13.1.3. General Method 4: Pyridinium salt reduction for the preparation of THPs (1b-21b)

To a solution of pyridinium salt (1 equiv.) in methanol:H_2O (9:1, 2.5 mL/mmol of pyridinium salt) was added $NaBH_4$ (2 equiv.) at -78 °C. The reaction mixture was stirred at -30 to -40 °C for 30 min, then allowed to warm to room temperature over 1 h. The reaction mixture was then quenched with saturated aqueous $NaHCO_3$. The aqueous phase was separated and extracted with DCM (3 × 20 mL). The combined organic extracts were washed with brine, dried over anhydrous $MgSO_4$ and concentrated *in vacuo* to give the crude product. The crude was purified by gradient flash column chromatography on silica (DCM:methanol) to afford the corresponding THP.

5-(3-methoxyphenyl)-1-propyl-1,2,3,6-THP (1b)

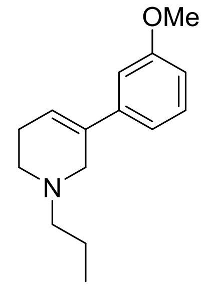

Following General Method 4 with 3-(3-methoxyphenyl)-1-propylpyridinium iodide (**1a**) (2.95 g, 8.31 mmol), sodium borohydride (2 equiv.) was added at 0 °C. The reaction mixture was stirred at 0 °C for 30 min, then allowed to warm to room temperature over 1 h. The crude product was subjected to flash column chromatography on silica (gradient elution from 99:1 to 98:2 DCM:methanol) afforded the title compound as an orange oil (1.62 g, 7.00 mmol, 84%). **^{1}H NMR** (500 MHz, $CDCl_3$): δ 7.22 (t, *J* = 8.1 Hz, 1H), 6.96 – 6.91 (m, 1H), 6.90 – 6.86 (m, 1H), 6.79 (dd, *J* = 8.1, 2.6 Hz, 1H), 6.14 – 6.09 (m, 1H), 3.81 (s, 3H), 3.33 (q, *J* = 2.4 Hz, 2H), 2.62 (t, *J* = 5.8 Hz, 2H), 2.52 – 2.45 (m, 2H), 2.41 – 2.33 (m, 2H), 1.62 (h, *J* = 7.4 Hz, 2H), 0.95 (t, *J* = 7.4 Hz, 3H); **^{13}C NMR** (126 MHz, $CDCl_3$): δ 159.7, 142.0, 135.5, 129.4, 123.0, 117.8, 112.3, 111.2, 60.7, 55.4, 54.9, 49.7, 26.6, 20.4, 12.2; **HRMS** calcd. for $C_{15}H_{22}NO^+$ 232.1696 [M+H]$^+$, found 232.1694.

3-(1-propyl-1,2,5,6-tetrahydropyridin-3-yl)phenol

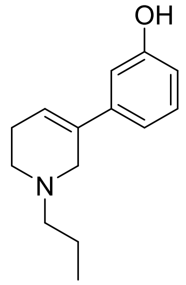

5-(3-methoxyphenyl)-1-propyl-1,2,3,6-THP (**1b**) (0.50 g, 2.16 mmol) was stirred in aqueous HBr (48% in H_2O, 15 mL, 132.59 mmol) at 100 ^oC for 3 h. The reaction mixture was cooled over ice and basified to pH 12 by addition of 5 M NaOH. The product was extracted into DCM (3 x 10 mL). The organic phase was dried over anhydrous $MgSO_4$ and concentrated *in vacuo* to afford the title compound as a pale-yellow oil (0.34 g, 1.56 mmol, 72%). **^{1}H NMR** (400 MHz, $CDCl_3$): δ 7.15 (t, *J* = 7.7 Hz, 1H), 6.78 – 6.69 (m, 3H), 5.89 (tt, *J* = 3.8, 1.8 Hz, 1H), 3.41 – 3.35 (m, 2H), 2.70 (t, *J* = 5.8 Hz, 2H), 2.58 – 2.49 (m, 2H), 2.41 – 2.31 (m, 2H), 1.75 – 1.61 (m, 2H), 0.95 (t, *J* = 7.3 Hz, 3H); **^{13}C NMR** (100 MHz, $CDCl_3$): δ 157.1, 142.2, 135.7, 129.7, 123.4, 117.2, 115.5, 113.4, 61.0, 54.8, 50.2, 25.8, 19.6, 12.2; **HRMS** calcd. for $C_{14}H_{20}NO^+$ 218.1539 $[M+H]^+$, found 218.1541.

5-(3-(methylsulfonyl)phenyl)-1-propyl-1,2,3,6-THP (2b)

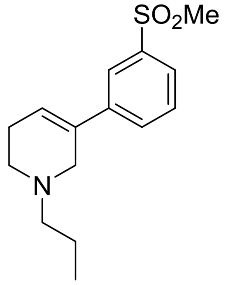

Following General Method 4 with 3-(3-(methylsulfonyl)phenyl)-1-propylpyridinium iodide (**2a**) (1.69 g, 4.20 mmol), sodium borohydride (2 equiv.) was added at 0 °C. The reaction mixture was stirred at 0 °C for 30 min, then allowed to warm to room temperature over 1 h. The crude product was subjected to flash column chromatography on silica (gradient elution from 99.5:0.5 to 95:5 DCM:methanol) to afford the title compound as a yellow oil (0.70 g, 2.51 mmol, 60%). **^{1}H NMR** (400 MHz, $CDCl_3$): δ 7.88 (d, *J* = 1.8 Hz, 1H), 7.79 (dd, *J* = 7.9, 1.8 Hz, 1H), 7.61 (dd, *J* = 7.9, 1.8 Hz, 1H), 7.50 (t, *J* = 7.9 Hz, 1H), 6.24 (td, *J* = 4.0, 2.1 Hz, 1H), 3.34 (q, *J* = 2.1 Hz, 2H), 3.05 (d, *J* = 1.3 Hz, 3H), 2.62 (t, *J* = 5.8

Hz, 2H), 2.53 – 2.45 (m, 2H), 2.39 (dt, J = 6.8, 3.2 Hz, 2H), 1.61 (h, J = 7.4 Hz, 2H), 0.94 (t, J = 7.4 Hz, 3H); **^{13}C NMR** (100 MHz, $CDCl_3$): δ 141.9, 140.8, 134.1, 130.2, 129.5, 125.7, 125.5, 123.8, 60.6, 54.6, 49.4, 44.7, 26.7, 20.4, 12.1; **HRMS** calcd. for $C_{15}H_{21}NO_2S^+$ 279.1293 $[M+H]^+$, found 279.1287.

1-allyl-5-(4-bromophenyl)-1,2,3,6-THP (3b)

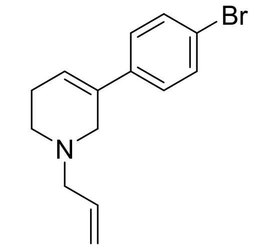

Following General Method 4 with 1-allyl-3-(4-bromophenyl)pyridinium bromide (**3a**) (1.00 g, 2.82 mmol), sodium borohydride (2 equiv.) was added at 0 °C. The reaction mixture was stirred at 0 °C for 30 min, then allowed to warm to room temperature over 1 h. The crude product was subjected on a short pad of silica (elution 9:1 DCM:methanol) to afford the title compound as a bright yellow oil (0.70 g, 2.52 mmol, 89%). **^{1}H NMR** (400 MHz, $CDCl_3$): δ 7.45 – 7.37 (m, 2H), 7.23 – 7.15 (m, 2H), 6.11 (tt, J = 3.9, 1.8 Hz, 1H), 6.02 – 5.87 (m, 1H), 5.30 – 5.20 (m, 1H), 5.20 – 5.14 (m, 1H), 3.29 (q, J = 2.4 Hz, 2H), 3.21 – 3.14 (m, 2H), 2.61 (t, J = 5.8 Hz, 2H), 2.39 – 2.30 (m, 2H); **^{13}C NMR** (100 MHz, $CDCl_3$): δ 139.2, 135.3, 134.6, 131.5, 126.7, 123.4, 120.9, 118.2, 61.6, 54.4, 49.1, 26.6; **HRMS** calcd. for $C_{14}H_{17}BrN^+$ 278.0539 $[M+H]^+$, found 278.0528.

***N*-(4-(1-allyl-1,2,5,6-tetrahydropyridin-3-yl)phenyl)acetamide**

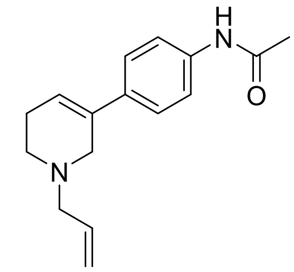

Following General Method 4 with 3-(4-acetamidophenyl)-1-allylpyridinium bromide (**4a**) (1.00 g, 3.00 mmol), sodium borohydride (2 equiv.) was added at 0 °C. The reaction mixture was stirred at 0 °C for 30 min, then allowed to warm to room temperature over 1 h. The crude product was subjected to flash column chromatography on silica (gradient elution from 99.5:0.5 to 98:2 DCM:methanol) to afford the title compound as an orange oil (0.55 g, 2.14 mmol, 71%). **^{1}H NMR** (400 MHz, $CDCl_3$): δ 7.47 (d, J =

8.3 Hz, 2H), 7.36 – 7.28 (m, 3H), 6.16 – 6.09 (m, 1H), 6.07 – 5.92 (m, 1H), 5.26 (dd, *J* = 25.8, 13.7 Hz, 2H), 3.38 – 3.32 (m, 2H), 3.22 (d, *J* = 6.5 Hz, 2H), 2.65 (t, *J* = 5.8 Hz, 2H), 2.45 – 2.36 (m, 2H), 2.21 (s, 3H); **^{13}C NMR** (100 MHz, $CDCl_3$): δ 168.3, 136.8, 136.4, 135.4, 134.8, 125.7, 122.2, 119.8, 118.1, 61.7, 54.6, 49.3, 26.6, 24.8; **HRMS** calcd. for $C_{16}H_{21}N_2O^+$ 257.1648 [M+H]$^+$, found 257.1639.

4-(1-allyl-1,2,5,6-tetrahydropyridin-3-yl)aniline (4b)

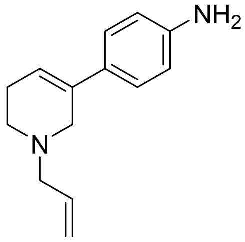

N-(4-(1-allyl-1,2,5,6-tetrahydropyridin-3-yl)phenyl)acetamide (250 mg, 0.98 mmol) was stirred in aqueous 2 M HCl (25 mL, 50 mmol) at 50 °C for 3 h. The reaction mixture was cooled over ice and basified to pH 12 by addition of 5 M NaOH. The product was extracted into DCM (3 x 10 mL). The organic phase was dried over anhydrous $MgSO_4$ and concentrated *in vacuo* to afford the title compound as a yellow oil (203 mg, 0.95 mmol, 97%). **^{1}H NMR** (400 MHz, $CDCl_3$): δ 7.19 – 7.11 (m, 2H), 6.67 – 6.59 (m, 2H), 6.04 – 5.89 (m, 2H), 5.29 – 5.14 (m, 2H), 3.63 (s, 0H), 3.29 (q, *J* = 2.4 Hz, 2H), 3.21 – 3.14 (m, 2H), 2.60 (t, *J* = 5.8 Hz, 2H), 2.38 – 2.29 (m, 2H); **^{13}C NMR** (100 MHz, $CDCl_3$): δ 145.6, 135.6, 135.1, 130.9, 126.1, 119.9, 118.0, 115.1, 61.7, 54.8, 49.4, 26.5; **HRMS** calcd. for $C_{14}H_{19}N_2^+$ 215.1534 [M+H]$^+$, found 215.1526.

1-ethyl-5-phenyl-1,2,3,6-THP (5b)

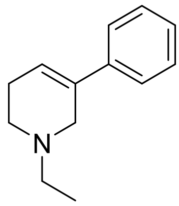

Following General Method 4 with 1-ethyl-3-phenylpyridinium iodide (**5a**) (2.50 g, 8.03 mmol) and subjecting the crude product to flash column chromatography on silica (gradient elution from 98:2 to 95:5 DCM:methanol) afforded the title compound as a yellow oil (1.31 g, 6.99 mmol, 87%). **^{1}H NMR** (400 MHz, $CDCl_3$): δ 7.36 – 7.22 (m, 4H), 7.22 – 7.16 (m, 1H), 6.08 (tt, *J* = 3.9, 1.8 Hz, 1H), 3.31 (q, *J* = 2.5 Hz, 2H), 2.62 – 2.52 (m, 4H), 2.35 (tt, *J* = 5.9, 2.9 Hz, 2H), 1.16 (t, *J* = 7.2 Hz, 3H); **^{13}C NMR**

(100 MHz, $CDCl_3$): δ 140.5, 135.5, 128.4, 127.0, 125.1, 122.7, 54.5, 52.3, 49.3, 26.6, 12.4; **HRMS** calcd. for $C_{13}H_{18}N^+$ 188.1434 $[M+H]^+$, found 188.1427.

1-ethyl-5-(4-fluorophenyl)-1,2,3,6-THP (6b)

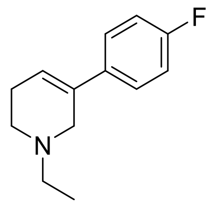

Following General Method 4 with 1-ethyl-3-(4-fluorophenyl)pyridinium iodide (**6a**) (1.00 g, 3.04 mmol) and subjecting the crude product to flash column chromatography on silica (gradient elution from 99:1 to 98:2 DCM:methanol) afforded the title compound as a pale-yellow oil (0.55 g, 2.68 mmol, 88%). **^{1}H NMR** (400 MHz, $CDCl_3$): δ 7.34 – 7.24 (m, 2H), 7.04 – 6.94 (m, 2H), 6.04 (tt, *J* = 3.9, 1.8 Hz, 1H), 3.29 (td, *J* = 2.7, 1.8 Hz, 2H), 2.64 – 2.55 (m, 4H), 2.41 – 2.32 (m, 2H), 1.18 (t, *J* = 7.2 Hz, 3H); **^{13}C NMR** (100 MHz, $CDCl_3$): δ 163.4, 160.9, 136.7, 134.8, 126.8, 126.7, 122.7, 115.3, 115.1, 54.7, 52.4, 49.3, 26.7, 12.5; **HRMS** calcd. for $C_{13}H_{17}FN^+$ 206.1340 $[M+H]^+$, found 206.1344.

1-ethyl-5-(4-methoxyphenyl)-1,2,3,6-THP (7b)

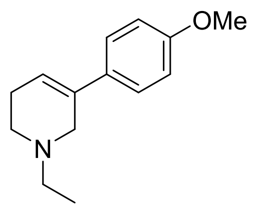

Following General Method 4 with 1-ethyl-3-(4-methoxyphenyl)pyridinium iodide (**7a**) (1.00 g, 2.93 mmol) and subjecting the crude product to flash column chromatography on silica (gradient elution from 98:2 to 95:5 DCM:methanol) afforded the title compound as a pale-yellow oil (0.39 g, 1.79 mmol, 61%). **^{1}H NMR** (400 MHz, $CDCl_3$): δ 7.32 – 7.23 (m, 2H), 6.89 – 6.81 (m, 2H), 6.01 (tt, *J* = 3.8, 1.8 Hz, 1H), 3.80 (d, *J* = 1.2 Hz, 3H), 3.31 (q, *J* = 2.4 Hz, 2H), 2.65 – 2.58 (m, 4H), 2.36 (tt, *J* = 5.9, 3.0 Hz, 2H), 1.19 (t, *J* = 7.2 Hz, 3H); **^{13}C NMR** (100 MHz, $CDCl_3$): δ 158.8, 134.9, 133.1, 126.3, 121.2, 113.8, 55.4, 54.6, 52.3, 49.4, 26.6, 12.4; **HRMS** calcd. for $C_{14}H_{20}NO^+$ 218.1539 $[M+H]^+$, found 218.1539.

1-ethyl-5-(furan-2-yl)-1,2,3,6-THP (8b)

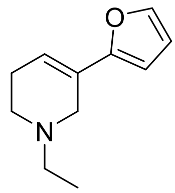

Following General Method 4 with 1-ethyl-3-(furan-2-yl)pyridinium iodide (**8a**) (1.00 g, 3.32 mmol) and subjecting the crude product to flash column chromatography on silica (gradient elution from 99:1 to 98:2 DCM:methanol) afforded the title compound as a red oil (0.44 g, 2.48 mmol, 74%). **^{1}H NMR** (400 MHz, $CDCl_3$): δ 7.32 (d, *J* = 1.8 Hz, 1H), 6.35 (dd, *J* = 3.3, 1.8 Hz, 1H), 6.28 (tt, *J* = 4.0, 1.8 Hz, 1H), 6.14 (d, *J* = 3.3 Hz, 1H), 3.23 (q, *J* = 2.4 Hz, 2H), 2.64 – 2.54 (m, 4H), 2.41 – 2.31 (m, 2H), 1.18 (t, *J* = 7.2 Hz, 3H); **^{13}C NMR** (100 MHz, $CDCl_3$): δ 153.5, 141.3, 126.4, 120.3, 111.0, 103.7, 52.2, 51.9, 49.5, 26.0, 12.4; **HRMS** calcd. for $C_{11}H_{16}NO^+$ 178.1226 $[M+H]^+$, found 178.1224.

1-ethyl-5-(thiophen-2-yl)-1,2,3,6-THP (9b)

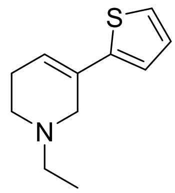

Following General Method 4 with 1-ethyl-3-(thiophen-2-yl)pyridinium iodide (**9a**) (1.00 g, 3.15 mmol) and subjecting the crude product to flash column chromatography on silica (gradient elution from 99:1 to 98:2 DCM:methanol) afforded the title compound as a brown oil (0.31 g, 1.60 mmol, 52%). **^{1}H NMR** (400 MHz, $CDCl_3$): δ 7.10 (dd, *J* = 5.1, 1.2 Hz, 1H), 6.94 (dd, *J* = 5.1, 3.7 Hz, 1H), 6.90 (d, *J* = 3.7 Hz, 1H), 6.17 (tt, *J* = 4.0, 1.7 Hz, 1H), 3.33 (q, *J* = 2.4 Hz, 2H), 2.63 – 2.51 (m, 4H), 2.34 (tt, *J* = 5.8, 2.9 Hz, 2H), 1.18 (t, *J* = 7.2 Hz, 3H); **^{13}C NMR** (100 MHz, $CDCl_3$): δ 144.2, 130.1, 127.2, 123.1, 121.9, 121.2, 54.2, 52.1, 49.3, 26.4, 12.4; **HRMS** calcd. for $C_{11}H_{16}NS^+$ 194.0998 $[M+H]^+$, found 194.1002.

1-ethyl-5-(naphthalen-2-yl)-1,2,3,6-THP (10b)

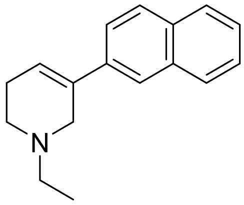

Following General Method 4 with 1-ethyl-3-(naphthalen-2-yl)pyridinium iodide (**10a**) (1.00 g, 2.77 mmol) and subjecting the crude product to flash column chromatography on silica (gradient elution from 99:1 to 98:2 DCM:methanol) afforded the title compound as a colourless oil (0.43 g, 1.81 mmol, 65%). **^{1}H NMR** (400 MHz, $CDCl_3$): δ 7.87 – 7.76 (m, 3H), 7.76 – 7.71 (m, 1H), 7.57 (dd, *J* = 8.6, 1.9 Hz, 1H), 7.51 – 7.39 (m, 2H), 6.30 (tt, *J* = 3.9, 1.8 Hz, 1H), 3.49 (q, *J* = 2.4 Hz, 2H), 2.71 – 2.61 (m, 4H), 2.45 (tt, *J* = 5.8, 3.0 Hz, 2H), 1.24 (t, *J* = 7.2 Hz, 3H); **^{13}C NMR** (100 MHz, $CDCl_3$): δ 137.5, 135.2, 133.5, 132.7, 128.1, 127.9, 127.6, 126.2, 125.7, 123.9, 123.4, 123.2, 54.4, 52.3, 49.3, 26.7, 12.4; **HRMS** calcd. for $C_{17}H_{20}N^+$ 238.1590 $[M+H]^+$, found 238.1588.

1-methyl-5-phenyl-1,2,3,6-THP (11b)

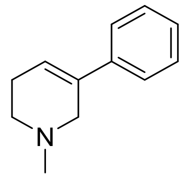

Following General Method 4 with 1-methyl-3-phenylpyridinium iodide (**11a**) (1.00 g, 3.37 mmol) and subjecting the crude product to flash column chromatography on silica (gradient elution from 98:2 to 95:5 DCM:methanol) afforded the title compound as a pale-yellow oil (0.35 g, 2.02 mmol, 60%). **^{1}H NMR** (400 MHz, $CDCl_3$): δ 7.29 – 7.08 (m, 5H), 6.03 (tt, *J* = 3.9, 1.8 Hz, 1H), 3.21 (td, *J* = 2.7, 1.8 Hz, 2H), 2.49 (t, *J* = 5.8 Hz, 2H), 2.38 (s, 3H), 2.35 – 2.26 (m, 2H); **^{13}C NMR** (100 MHz, $CDCl_3$): δ 140.2, 135.4, 128.4, 127.1, 125.1, 122.2, 56.5, 51.6, 46.1, 26.6; **HRMS** calcd. for $C_{12}H_{16}N^+$ 174.1277 $[M+H]^+$, found 174.1278.

5-phenyl-1-(prop-2-yn-1-yl)-1,2,3,6-THP (12b)

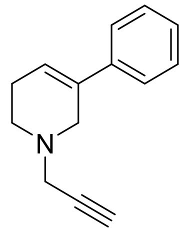

Following General Method 4 with 3-phenyl-1-(prop-2-yn-1-yl)pyridinium bromide (**12a**) (1.00 g, 3.65 mmol) and subjecting the crude product to flash column chromatography on silica (gradient elution from 99.5:0.5 to 99:1 DCM:methanol) afforded the title compound as a yellow oil (0.43 g, 2.17 mmol, 60%). **^{1}H NMR** (400 MHz, $CDCl_3$): δ 7.43 – 7.35 (m, 4H), 7.35 – 7.26 (m, 2H), 6.16 (tt, *J* = 3.7, 1.8

Hz, 1H), 3.54 (dd, J = 17.0, 2.5 Hz, 4H), 2.77 (t, J = 5.8 Hz, 2H), 2.44 (tt, J = 6.0, 3.0 Hz, 2H); **^{13}C NMR** (100 MHz, $CDCl_3$): δ 140.2, 135.4, 128.5, 127.2, 125.2, 122.2, 78.9, 73.4, 53.0, 48.4, 46.8, 26.6; **HRMS** calcd. for $C_{14}H_{15}N^+$ 197.1204 $[M+H]^+$, found 197.1211.

1-ethyl-5-methyl-1,2,3,6-THP (13b)

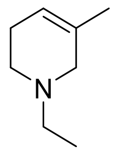

Following General Method 4 with 1-ethyl-3-methylpyridinium iodide (**13a**) (5.00 g, 20.07 mmol) and subjecting the crude product to flash column chromatography on silica (gradient elution from 99:1 to 95:5 DCM:methanol) afforded the title compound as a colourless oil (1.21 g, 9.66 mmol, 48%). **^{1}H NMR** (400 MHz, $CDCl_3$): δ 5.46 – 5.38 (m, 1H), 2.84 – 2.77 (m, 2H), 2.50 – 2.41 (m, 4H), 2.18 – 2.08 (m, 2H), 1.66 – 1.60 (m, 3H), 1.11 (t, J = 7.2 Hz, 3H); **^{13}C NMR** (100 MHz, $CDCl_3$): δ 132.3, 119.5, 56.7, 52.3, 49.6, 26.2, 21.2, 12.4; **HRMS** calcd. for $C_8H_{16}N^+$ 126.1277 $[M+H]^+$, found 126.1274.

1-benzyl-5-methyl-1,2,3,6-THP (14b)

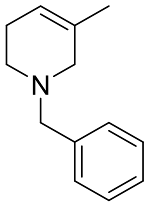

Following General Method 4 with 1-benzyl-3-methylpyridinium bromide (**14a**) (5.00 g, 18.93 mmol) and subjecting the crude product to flash column chromatography on silica (gradient elution from 99:1 to 95:5 DCM:methanol) afforded the title compound as a yellow oil (2.41 g, 12.87 mmol, 68%). **^{1}H NMR** (400 MHz, $CDCl_3$): δ 7.34 – 7.15 (m, 5H), 5.43 – 5.35 (m, 1H), 3.53 (s, 2H), 2.82 – 2.76 (m, 2H), 2.44 (t, J = 5.8 Hz, 2H), 2.11 – 2.02 (m, 2H), 1.60 – 1.54 (m, 3H); **^{13}C NMR** (100 MHz, $CDCl_3$): δ 138.5, 132.4, 129.3, 128.3, 127.1, 119.6, 63.0, 57.2, 49.6, 26.1, 21.2; **HRMS** calcd. for $C_{13}H_{18}N^+$ 188.1434 $[M+H]^+$, found 188.1429.

1-ethyl-4-methyl-1,2,3,6-THP (15b)

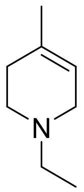

Following General Method 4 with 1-ethyl-4-methylpyridinium iodide (**15a**) (5.00 g, 20.07 mmol) and subjecting the crude product to a short pad of silica (elution 9:1 DCM:methanol) afforded the title compound as a pale-yellow oil (1.95 g, 15.57 mmol, 78%). ^{1}**H NMR** (400 MHz, $CDCl_3$): δ 5.38 – 5.31 (m, 1H), 2.92 – 2.84 (m, 2H), 2.51 (t, *J* = 5.8 Hz, 2H), 2.43 (q, *J* = 7.2 Hz, 2H), 2.11 – 2.03 (m, 2H), 1.67 – 1.62 (m, 3H), 1.09 (t, *J* = 7.2 Hz, 3H); ^{13}**C NMR** (100 MHz, $CDCl_3$): δ 132.8, 119.3, 52.5, 52.2, 50.2, 31.1, 23.0, 12.5.

1-ethyl-4-phenyl-1,2,3,6-THP (16b)

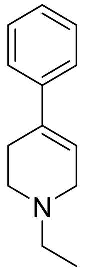

Following General Method 4 with 1-ethyl-4-phenylpyridinium iodide (**16a**) (2.00 g, 6.43 mmol) and subjecting the crude product to a short pad of silica (elution 9:1 DCM:methanol) afforded the title compound as a yellow oil (1.03 g, 5.50 mmol, 85%). ^{1}**H NMR** (400 MHz, $CDCl_3$): δ 7.38 – 7.30 (m, 2H), 7.30 – 7.22 (m, 2H), 7.22 – 7.13 (m, 1H), 6.02 (tt, *J* = 3.5, 1.6 Hz, 1H), 3.11 (q, *J* = 3.0 Hz, 2H), 2.65 (t, *J* = 5.9 Hz, 2H), 2.59 – 2.44 (m, 4H), 1.12 (t, *J* = 7.2 Hz, 3H); ^{13}**C NMR** (100 MHz, $CDCl_3$): δ 141.1, 135.1, 128.3, 127.0, 125.0, 122.0, 52.9, 52.2, 50.2, 28.3, 12.5.

1-ethyl-4,5-dimethyl-1,2,3,6-THP (17b)

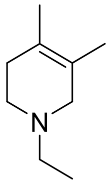

Following General Method 4 with 1-ethyl-3,4-dimethylpyridinium iodide (**17a**) (0.80 g, 3.04 mmol) and subjecting the crude product to flash column chromatography on silica (gradient elution from 99:1 to 95:5 DCM:methanol) afforded the title compound as a yellow oil (0.28 g, 1.98 mmol, 65%).

17b (HCl salt): **^{1}H NMR** (400 MHz, $CDCl_3$): δ 12.37 (s, 1H), 3.73 (d, *J* = 16.0 Hz, 1H), 3.42 (dt, *J* = 11.1, 5.1 Hz, 1H), 3.11 (ddq, *J* = 26.5, 13.0, 6.3, 5.8 Hz, 3H), 2.93 (qd, *J* = 12.4, 10.4, 5.4 Hz, 1H), 2.81 (d, *J* = 19.0 Hz, 1H), 2.21 – 2.06 (m, 1H), 1.72 (s, 3H), 1.66 (s, 3H), 1.50 (t, *J* = 7.2 Hz, 3H); **^{13}C NMR** (100 MHz, $CDCl_3$): δ 125.8, 118.2, 52.8, 50.5, 48.0, 27.2, 18.4, 16.2, 9.5.

2-ethyl-1,2,3,4,5,6,7,8-octahydroisoquinoline (18b)

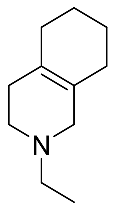

Following General Method 4 with 2-ethyl-5,6,7,8-tetrahydroisoquinolinium iodide (**18a**) (1.00 g, 3.46 mmol) and subjecting the crude product to a short pad of silica (elution 9:1 DCM:methanol) afforded the title compound as a dark yellow oil (0.49 g, 2.97 mmol, 86%). **^{1}H NMR** (400 MHz, $CDCl_3$): δ 2.77 – 2.71 (m, 2H), 2.51 (t, *J* = 5.9 Hz, 2H), 2.43 (q, *J* = 7.2 Hz, 2H), 2.07 – 1.98 (m, 2H), 1.89 – 1.79 (m, 4H), 1.63 – 1.52 (m, 4H), 1.11 (t, *J* = 7.2 Hz, 3H); **^{13}C NMR** (100 MHz, $CDCl_3$): δ 126.9, 126.5, 56.8, 52.3, 50.5, 31.0, 29.5, 27.8, 23.0, 22.9, 12.5; **HRMS** calcd. for $C_{11}H_{20}N^+$ 166.1590 $[M+H]^+$, found 166.1590.

1-ethyl-4-methyl-5-phenyl-1,2,3,6-THP (19b)

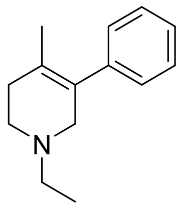

Following General Method 4 with 1-ethyl-4-methyl-3-phenylpyridinium iodide (**19a**) (0.50 g, 1.54 mmol), sodium borohydride (2 equiv.) was added at 0 °C. The reaction mixture was stirred at 0 °C for 30 min, then allowed to warm to room temperature over 1 h. The crude product was subjected to flash column chromatography on silica (gradient elution from 98:2 to 95:5 DCM:methanol) to afford the title

compound as a yellow oil (0.26 g, 1.29 mmol, 84%). **^{1}H NMR** (400 MHz, $CDCl_3$): δ 7.31 (t, *J* = 7.5 Hz, 2H), 7.26 – 7.18 (m, 1H), 7.17 (d, *J* = 7.5 Hz, 2H), 3.20 – 3.13 (m, 2H), 2.68 (t, *J* = 5.9 Hz, 2H), 2.55 (q, *J* = 7.2 Hz, 2H), 2.32 – 2.26 (m, 2H), 1.58 (s, 3H), 1.16 (t, *J* = 7.2 Hz, 3H); **^{13}C NMR** (100 MHz, $CDCl_3$): δ 141.6, 130.8, 128.9, 128.2, 128.0, 126.6, 57.8, 52.2, 50.2, 32.3, 19.8, 12.5; **HRMS** calcd. for $C_{14}H_{20}N^+$ 202.1590 [M+H]$^+$, found 202.1592.

1-allyl-4-methyl-5-phenyl-1,2,3,6-THP (20b)

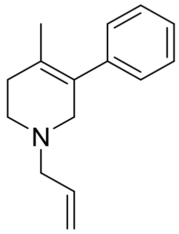

Following General Method 4 with 1-allyl-4-methyl-3-phenylpyridinium bromide (**20a**) (1.34 g, 4.62 mmol), sodium borohydride (2 equiv.) was added at 0 °C. The reaction mixture was stirred at 0 °C for 30 min, then allowed to warm to room temperature over 1 h. The crude product was subjected to flash column chromatography on silica (gradient elution from 99.5:0.5 to 98:2 DCM:methanol) to afford the title compound as a yellow oil (0.45 g, 2.11 mmol, 45%). **^{1}H NMR** (400 MHz, $CDCl_3$): δ 7.36 – 7.28 (m, 2H), 7.27 – 7.22 (m, 1H), 7.22 – 7.13 (m, 2H), 6.02 – 5.87 (m, 1H), 5.25 – 5.10 (m, 2H), 3.17 – 3.08 (m, 4H), 2.65 (t, *J* = 5.8 Hz, 2H), 2.31 – 2.21 (m, 2H), 1.62 – 1.57 (m, 3H); **^{13}C NMR** (100 MHz, $CDCl_3$): δ 141.4, 135.5, 130.8, 128.9, 128.2, 128.0, 126.6, 118.0, 61.5, 58.0, 50.1, 32.1, 19.8; **HRMS** calcd. for $C_{15}H_{20}N^+$ 214.1590 [M+H]$^+$, found 214.1597.

1-allyl-5-methyl-4-phenyl-1,2,3,6-THP (21b)

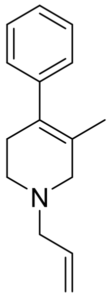

Following General Method 4 with 1-allyl-3-methyl-4-phenylpyridinium bromide (**21a**) (1.70 g, 5.86 mmol), sodium borohydride (2 equiv.) was added at 0 °C. The reaction mixture was stirred at 0 °C for 30 min, then allowed to warm to room temperature over 1 h. The crude product was subjected to flash

column chromatography on silica (gradient elution from 99.5:0.5 to 98:2 DCM:methanol) to afford the title compound as a yellow oil (0.94 g, 4.40 mmol, 75%). **^{1}H NMR** (400 MHz, $CDCl_3$): δ 7.36 – 7.28 (m, 2H), 7.28 – 7.20 (m, 1H), 7.20 – 7.12 (m, 2H), 6.04 – 5.90 (m, 1H), 5.30 – 5.22 (m, 1H), 5.22 – 5.14 (m, 1H), 3.13 (dt, *J* = 6.7, 1.3 Hz, 2H), 3.02 – 2.96 (m, 2H), 2.66 (t, *J* = 5.8 Hz, 2H), 2.49 – 2.39 (m, 2H), 1.59 – 1.55 (m, 3H); **^{13}C NMR** (100 MHz, $CDCl_3$): δ 142.7, 135.5, 130.8, 128.6, 128.2, 127.5, 126.4, 118.1, 61.6, 58.1, 50.5, 32.3, 18.1; **HRMS** calcd. for $C_{15}H_{20}N^+$ 214.1590 [M+H]$^+$, found 214.1585.

13.2. General Method 5: Hydrogenation of THP for the preparation of racemic standards

To a schlenk flask charged with 10% Pd/C (0.1 equiv.), THP (1 equiv.) in methanol (3.60 mL/mmol of THP) was added under nitrogen. The sealed flask was purged with two cycles of hydrogen (balloons) and the mixture was vigorously stirred at room temperature under hydrogen (balloon) overnight. Excess hydrogen was evacuated and replaced with nitrogen before the reaction mixture was filtered through celite. Methanol was removed *in vacuo* to afford the corresponding racemic standard.

3-(3-methoxyphenyl)-1-propylpiperidine

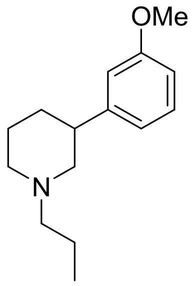

Following General Method 5 with 5-(3-methoxyphenyl)-1-propyl-1,2,3,6-THP (**1b**) (1.81 g, 7.81 mmol) afforded the title compound as a yellow oil (0.90 g, 3.86 mmol, 49%). **^{1}H NMR** (500 MHz, $CDCl_3$): δ 7.21 (t, *J* = 7.8 Hz, 1H), 6.83 (d, *J* = 7.8 Hz, 1H), 6.81 – 6.77 (m, 1H), 6.75 (dd, *J* = 8.2, 2.7 Hz, 1H), 3.79 (s, 3H), 3.06 – 2.94 (m, 2H), 2.81 (tt, *J* = 11.6, 3.7 Hz, 1H), 2.31 (td, *J* = 7.0, 2.2 Hz, 2H), 1.99 – 1.89 (m, 3H), 1.83 – 1.66 (m, 2H), 1.60 – 1.38 (m, 3H), 0.89 (t, *J* = 7.4 Hz, 3H); **^{13}C NMR** (126 MHz, $CDCl_3$): δ 159.7, 146.6, 129.4, 119.7, 113.4, 111.4, 61.4, 61.3, 55.2, 54.0, 43.1, 31.7, 25.8, 20.1, 12.1; **HRMS** calcd. for $C_{15}H_{24}NO^+$ 234.1852 [M+H]$^+$, found 234.1842.

3-(1-propylpiperidin-3-yl)phenol

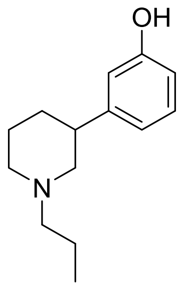

Following General Method 5 with 3-(1-propyl-1,2,5,6-tetrahydropyridin-3-yl)phenol (0.50 g, 2.30 mmol) afforded the title compound as a yellow oil (0.38 g, 1.73 mmol, 75%). **^{1}H NMR** (400 MHz, $CDCl_3$): δ 7.14 (t, *J* = 8.1 Hz, 1H), 6.70 – 6.64 (m, 3H), 3.14 (d, *J* = 10.0 Hz, 1H), 3.02 (d, *J* = 11.5 Hz, 1H), 2.83 (tt, *J* = 11.9, 3.5 Hz, 1H), 2.35 (td, *J* = 11.6, 5.3 Hz, 1H), 2.22 (td, *J* = 11.6, 5.3 Hz, 1H), 2.03 – 1.84 (m, 3H), 1.82 – 1.62 (m, 2H), 1.59 – 1.40 (m, 3H), 0.77 (t, *J* = 7.3 Hz, 3H); **^{13}C NMR** (100 MHz, $CDCl_3$): δ 157.4, 145.6, 130.2, 116.9, 115.2, 114.6, 61.6, 61.5, 54.2, 43.3, 30.2, 25.5, 19.1, 12.2; **HRMS** calcd. for $C_{14}H_{22}NO^+$ 220.1696 $[M+H]^+$, found 220.1697.

3-(3-(methylsulfonyl)phenyl)-1-propylpiperidine

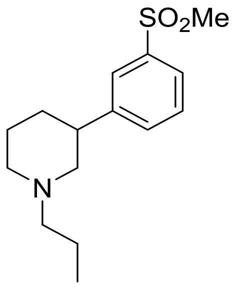

Following General Method 5 with 5-(3-(methylsulfonyl)phenyl)-1-propyl-1,2,3,6-THP (**2b**) (1.00 g, 3.58 mmol) afforded the title compound as an orange oil (0.89 g, 3.15 mmol, 88%). **^{1}H NMR** (400 MHz, $CDCl_3$): δ 7.82 – 7.73 (m, 2H), 7.54 – 7.44 (m, 2H), 3.04 (s, 3H), 3.02 – 2.84 (m, 3H), 2.36 – 2.26 (m, 2H), 2.05 – 1.88 (m, 3H), 1.83 – 1.75 (m, 1H), 1.75 – 1.63 (m, 1H), 1.57 – 1.37 (m, 3H), 0.88 (t, *J* = 7.4 Hz, 3H); **^{13}C NMR** (100 MHz, $CDCl_3$): δ 146.9, 140.7, 133.0, 129.5, 125.9, 125.4, 61.2, 60.9, 53.8, 44.6, 42.9, 31.7, 25.6, 20.1, 12.1; **HRMS** calcd. for $C_{15}H_{23}NO_2S^+$ 281.1449 $[M+H]^+$, found 281.1458.

3-phenyl-1-propylpiperidine

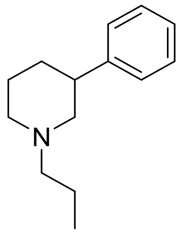

Following General Method 5 with 1-allyl-5-(4-bromophenyl)-1,2,3,6-THP (**3b**) (200 mg, 0.72 mmol) afforded the title compound as a colourless oil (52.6 mg, 0.26 mmol, 36%). **^{1}H NMR** (400 MHz, $CDCl_3$): δ 7.34 – 7.16 (m, 5H), 3.62 – 3.45 (m, 3H), 3.02 – 2.83 (m, 2H), 2.81 – 2.67 (m, 2H), 2.41 (qt, *J* = 13.3, 3.8 Hz, 1H), 2.13 – 2.03 (m, 1H), 2.03 – 1.83 (m, 3H), 1.71 (qd, *J* = 13.3, 3.8 Hz, 1H), 0.97 (t, *J* = 7.4 Hz, 3H); **^{13}C NMR** (100 MHz, $CDCl_3$): δ 140.6, 128.9, 127.5, 127.1, 59.3, 58.1, 52.7, 39.8, 29.5, 22.9, 17.5, 11.3; **HRMS** calcd. for $C_{14}H_{22}N^+$ 204.1747 $[M+H]^+$, found 204.1756.

4-(1-propylpiperidin-3-yl)aniline

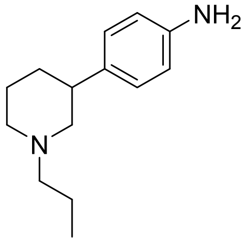

Following General Method 5 with 4-(1-allyl-1,2,5,6-tetrahydropyridin-3-yl)aniline (**4b**) (0.50 g, 2.33 mmol) afforded the title compound as a colourless oil (0.36 g, 1.65 mmol, 71%). **^{1}H NMR** (400 MHz, $CDCl_3$): δ 7.02 (d, *J* = 8.4 Hz, 2H), 6.63 (d, *J* = 8.4 Hz, 2H), 3.57 (s, 1H), 3.02 – 2.92 (m, 2H), 2.71 (tt, *J* = 11.7, 3.6 Hz, 1H), 2.34 – 2.25 (m, 2H), 1.94 – 1.83 (m, 3H), 1.81 – 1.62 (m, 2H), 1.58 – 1.46 (m, 2H), 1.38 (qd, *J* = 12.3, 4.4 Hz, 1H), 0.88 (t, *J* = 7.4 Hz, 3H); **^{13}C NMR** (100 MHz, $CDCl_3$): δ 144.7, 135.2, 128.1, 115.3, 61.9, 61.4, 54.1, 42.2, 31.9, 26.0, 20.2, 12.2; **HRMS** calcd. for $C_{14}H_{23}N_2^+$ 219.1856 $[M+H]^+$, found 219.1855.

1-ethyl-3-phenylpiperidine

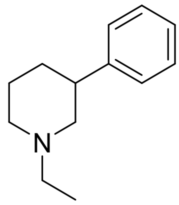

Following General Method 5 with 1-ethyl-5-phenyl-1,2,3,6-THP (**5b**) (1.00 g, 5.34 mmol) afforded the title compound as a colourless oil (0.75 g, 3.95 mmol, 74%).

Racemic HCl salt: **^{1}H NMR** (400 MHz, $CDCl_3$): δ 12.33 (s, 1H), 7.31 – 7.20 (m, 3H), 7.19 – 7.14 (m, 2H), 3.63 – 3.44 (m, 3H), 3.01 (ddq, *J* = 18.4, 13.1, 6.5, 6.0 Hz, 2H), 2.65 – 2.38 (m, 3H), 2.01 (dd, *J* = 47.1, 14.0 Hz, 2H), 1.84 (s, 1H), 1.60 (qd, *J* = 12.8, 3.6 Hz, 1H), 1.43 (t, *J* = 7.2 Hz, 3H); **^{13}C NMR** (100 MHz, $CDCl_3$): δ 140.5, 129.1, 127.8, 127.2, 57.7, 52.9, 52.4, 39.6, 29.7, 22.8, 9.3.

(*S*)-1-ethyl-3-phenylpiperidine

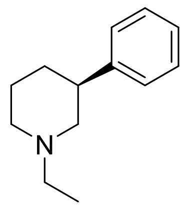

To a solution of the commercially available (*S*)-3-phenylpiperidine (50 mg, 0.31 mmol) and K_2CO_3 (85 mg, 0.62 mmol) in MeCN (1 ml) was added iodoethane (27 µl, 0.34 mmol) at room temperature. The solution was allowed to stir at room temperature overnight. The reaction was stopped by the addition of H_2O and washed with DCM. The organic phase was dried over $MgSO_4$, filtered and concentrated under vacuo to yield the pure compound **5c** as an off white oil in 51% yield. Spectroscopic data is in accord with previous.

1-ethyl-3-(4-fluorophenyl)piperidine

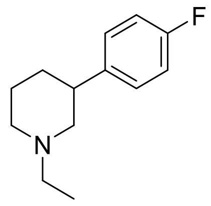

Following General Method 5 with 1-ethyl-5-(4-fluorophenyl)-1,2,3,6-THP (**6b**) (0.50 g, 2.44 mmol) afforded the title compound as a pale-yellow oil (0.10 g, 0.48 mmol, 20%). **^{1}H NMR** (400 MHz, $CDCl_3$): δ 7.15 (dd, *J* = 8.5, 5.5 Hz, 2H), 6.94 (t, *J* = 8.5 Hz, 2H), 3.01 – 2.93 (m, 2H), 2.85 – 2.73 (m, 1H), 2.40 (q, *J* = 7.2 Hz, 2H), 1.92 – 1.82 (m, 3H), 1.81 – 1.62 (m, 2H), 1.38 (qd, *J* = 12.4, 4.4 Hz, 1H), 1.07 (t, *J* = 7.2 Hz, 3H); **^{13}C NMR** (100 MHz, $CDCl_3$): δ 162.7, 160.2, 140.6, 128.6, 128.5, 115.2,

115.0, 61.2, 53.4, 52.8, 42.3, 31.9, 25.8, 12.1; **HRMS** calcd. for $C_{13}H_{19}FN^+$ 208.1496 $[M+H]^+$, found 208.1488.

1-ethyl-3-(4-methoxyphenyl)piperidine

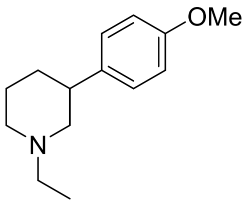

Following General Method 5 with 1-ethyl-5-(4-methoxyphenyl)-1,2,3,6-THP (**7b**) (0.21 g, 0.97 mmol) afforded the title compound as a yellow oil (0.19 g, 0.87 mmol, 89%). **^{1}H NMR** (400 MHz, $CDCl_3$): δ 7.19 – 7.12 (m, 2H), 6.84 (d, *J* = 8.1 Hz, 2H), 3.79 (s, 3H), 3.01 (d, *J* = 11.5 Hz, 2H), 2.79 (t, *J* = 11.8 Hz, 1H), 2.43 (q, *J* = 7.2 Hz, 2H), 1.89 (t, *J* = 11.2 Hz, 3H), 1.81 – 1.67 (m, 2H), 1.42 (qd, *J* = 12.3, 4.3 Hz, 1H), 1.09 (t, *J* = 7.9 Hz, 3H); **^{13}C NMR** (100 MHz, $CDCl_3$): δ 158.2, 137.2, 128.2, 113.9, 61.4, 55.4, 53.6, 52.9, 42.3, 32.0, 26.0, 12.2; **HRMS** calcd. for $C_{14}H_{22}NO^+$ 220.1696 $[M+H]^+$, found 220.1694.

1-ethyl-3-(furan-2-yl)piperidine

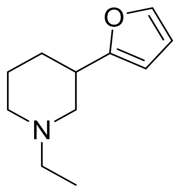

Following General Method 5 with 1-ethyl-5-(furan-2-yl)-1,2,3,6-THP (**8b**) (0.29 g, 1.66 mmol) afforded the title compound as a yellow oil (0.22 g, 1.23 mmol, 74%). **^{1}H NMR** (500 MHz, $CDCl_3$): δ 7.33 – 7.29 (m, 1H), 6.31 – 6.27 (m, 1H), 6.06 (d, *J* = 3.2 Hz, 1H), 3.46 – 3.39 (m, 1H), 3.38 – 3.22 (m, 2H), 2.82 – 2.73 (m, 2H), 2.35 – 2.24 (m, 2H), 2.14 – 2.03 (m, 2H), 1.91 – 1.83 (m, 1H), 1.52 (qd, *J* = 13.5, 13.0, 3.9 Hz, 1H), 1.30 (t, *J* = 7.3 Hz, 3H); **^{13}C NMR** (126 MHz, $CDCl_3$): δ 155.9, 141.5, 110.2, 104.9, 56.6, 52.9, 52.7, 34.8, 28.5, 23.7, 10.6; **HRMS** calcd. for $C_{11}H_{18}NO^+$ 180.1383 $[M+H]^+$, found 180.1375.

1-ethyl-3-(thiophen-2-yl)piperidine

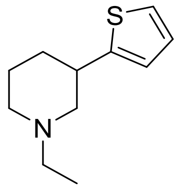

Following General Method 5 with 1-ethyl-5-(thiophen-2-yl)-1,2,3,6-THP (**9b**) (0.61 g, 3.15 mmol) afforded the title compound as a brown oil (0.18 g, 0.92 mmol, 29%). **^{1}H NMR** (500 MHz, $CDCl_3$): δ 7.13 (d, *J* = 5.1 Hz, 1H), 6.93 (dd, *J* = 5.1, 3.5 Hz, 1H), 6.84 (d, *J* = 3.5 Hz, 1H), 3.29 – 3.16 (m, 2H), 3.08 – 3.00 (m, 1H), 2.53 (q, *J* = 7.2 Hz, 2H), 2.14 – 1.97 (m, 3H), 1.86 – 1.77 (m, 2H), 1.51 – 1.40 (m, 1H), 1.15 (t, *J* = 7.2 Hz, 3H); **^{13}C NMR** (126 MHz, $CDCl_3$): δ 148.0, 126.7, 123.1, 123.0, 61.0, 53.2, 52.7, 37.8, 33.0, 25.2, 11.7; **HRMS** calcd. for $C_{11}H_{18}NS^+$ 196.1154 $[M+H]^+$, found 196.1148.

1-ethyl-3-(naphthalen-2-yl)piperidine

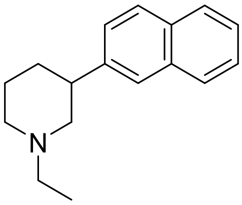

Following General Method 5 with 1-ethyl-5-(naphthalen-2-yl)-1,2,3,6-THP (**10b**) (0.33 g, 1.39 mmol) afforded the title compound as a yellow oil (0.27 g, 1.12 mmol, 81%). **^{1}H NMR** (400 MHz, $CDCl_3$): δ 7.84 – 7.75 (m, 3H), 7.67 (s, 1H), 7.49 – 7.36 (m, 3H), 3.18 – 3.10 (m, 1H), 3.10 – 2.95 (m, 2H), 2.48 (q, *J* = 7.2 Hz, 2H), 2.10 – 1.92 (m, 3H), 1.83 – 1.73 (m, 2H), 1.58 (qd, *J* = 12.3, 4.7 Hz, 1H), 1.12 (t, *J* = 7.2 Hz, 3H); **^{13}C NMR** (100 MHz, $CDCl_3$): δ 142.4, 133.7, 132.5, 128.0, 127.8, 127.7, 126.4, 126.1, 125.5, 125.4, 61.0, 53.7, 52.9, 43.2, 31.8, 26.0, 12.2; **HRMS** calcd. for $C_{17}H_{22}N^+$ 240.1747 $[M+H]^+$, found 240.1745.

1-methyl-3-phenylpiperidine

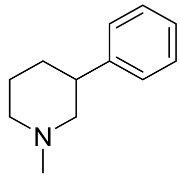

Following General Method 5 with 1-methyl-5-phenyl-1,2,3,6-THP (**11b**) (0.29 g, 1.68 mmol) afforded the title compound as a colourless oil (0.21 g, 1.20 mmol, 71%). **^{1}H NMR** (400 MHz, $CDCl_3$): δ 7.34 –

7.16 (m, 5H), 3.00 – 2.88 (m, 2H), 2.88 – 2.77 (m, 1H), 2.30 (s, 3H), 2.10 – 1.86 (m, 3H), 1.85 – 1.66 (m, 2H), 1.43 (qd, J = 12.4, 4.5 Hz, 1H); ^{13}**C NMR** (100 MHz, $CDCl_3$): δ 144.8, 128.5, 127.3, 126.5, 63.4, 56.0, 46.7, 43.2, 31.1, 26.0; **HRMS** calcd. for $C_{12}H_{18}N^+$ 176.1434 $[M+H]^+$, found 176.1433.

(*S*)-1-methyl-3-phenylpiperidine

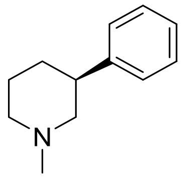

To a solution of the commercially available (*S*)-3-phenylpiperidine (50 mg, 0.31 mmol) and K_2CO_3 (85 mg, 0.62 mmol) in MeCN (1 ml) was added iodomethane (21 µl, 0.34 mmol) at room temperature. The solution was allowed to stir at room temperature overnight. The reaction was stopped by the addition of H_2O and washed with DCM. The organic phase was dried over $MgSO_4$, filtered and concentrated under vacuo to yield the pure compound **11c** as a white solid in 59% yield. Spectroscopic data is in accord with previous.

1-ethyl-3-methylpiperidine

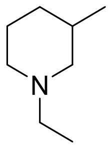

Following General Method 5 with 1-ethyl-5-methyl-1,2,3,6-THP (**13b**) (1.00 g, 7.99 mmol) afforded the title compound the title compound as a colourless oil (0.42 g, 3.27 mmol, 41%).

Racemic HCl salt: ^{1}**H NMR** (400 MHz, $CDCl_3$): δ 11.69 (s, 1H), 3.53 (d, J = 12.0 Hz, 1H), 3.45 – 3.34 (m, 1H), 3.11 – 2.97 (m, 2H), 2.55 – 2.09 (m, 4H), 1.97 – 1.77 (m, 2H), 1.46 (t, J = 7.3 Hz, 3H), 1.14 – 1.00 (m, 1H), 0.95 (d, J = 6.6 Hz, 3H); ^{13}**C NMR** (100 MHz, $CDCl_3$): δ 58.6, 52.7, 52.3, 31.0, 28.8, 22.7, 19.1, 9.2.

1-benzyl-3-methylpiperidine

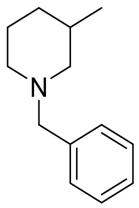

Following General Method 5 with 1-benzyl-5-methyl-1,2,3,6-THP (**14b**) (2.00 g, 10.68 mmol) afforded the title compound as a colourless oil (1.86 g, 9.82 mmol, 92%). **^{1}H NMR** (400 MHz, $CDCl_3$): δ 7.30 – 7.13 (m, 5H), 3.42 (s, 2H), 2.90 – 2.68 (m, 2H), 1.86 – 1.75 (m, 1H), 1.70 – 1.40 (m, 6H), 0.78 (d, *J* = 6.3 Hz, 3H); **^{13}C NMR** (100 MHz, $CDCl_3$): δ 138.8, 129.2, 128.2, 126.9, 63.7, 62.1, 54.1, 33.2, 31.2, 25.7, 19.9.

1-ethyl-4-methylpiperidine

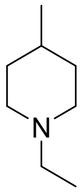

Following General Method 5 with 1-ethyl-4-methyl-1,2,3,6-THP (**15b**) (1.00 g, 7.99 mmol) afforded the title compound the title compound as a colourless oil (0.30 g, 2.40 mmol, 30%).

Racemic HCl salt: **^{1}H NMR** (400 MHz, $CDCl_3$): δ 10.95 (s, 1H), 3.43 (d, *J* = 11.5 Hz, 2H), 3.09 – 2.96 (m, 2H), 2.79 – 2.65 (m, 2H), 1.87 – 1.75 (m, 3H), 1.67 – 1.56 (m, 1H), 1.36 (t, *J* = 7.0 Hz, 3H), 0.99 (dd, *J* = 11.9, 6.5 Hz, 1H), 0.91 (d, *J* = 6.3 Hz, 3H); **^{13}C NMR** (100 MHz, $CDCl_3$): δ 52.4, 52.3, 30.8, 29.1, 20.9, 9.3; **HRMS** calcd. for $C_8H_{18}ClN^+$ 128.1434 [M-Cl]$^+$, found 128.1429.

1-ethyl-3,4-dimethylpiperidine

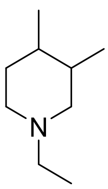

Following General Method 5 with 1-ethyl-4,5-dimethyl-1,2,3,6-THP (**17b**) (0.27 mg, 1.90 mmol) afforded the title compound as a colourless oil (0.16 g, 1.13 mmol, 59%). **^{1}H NMR** (400 MHz, $CDCl_3$): δ 3.55 – 2.95 (m, 6H), 1.44 (t, *J* = 7.3 Hz, 3H), 1.34 – 1.07 (m, 4H), 1.04 – 0.88 (m, 6H); **^{13}C NMR**

(100 MHz, $CDCl_3$): δ 52.4, 46.4, 36.2, 31.2, 31.0, 29.8, 18.5, 16.5, 9.3; **HRMS** calcd. for $C_9H_{20}N^+$ 142.1590 $[M+H]^+$, found 142.1585.

2-ethyldecahydroisoquinoline

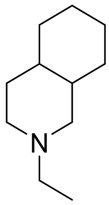

Following General Method 5 with 2-ethyl-1,2,3,4,5,6,7,8-octahydroisoquinoline (**18b**) (230 mg, 1.39 mmol) afforded the title compound as a colourless oil (140 mg, 0.83 mmol, 60%). **^{1}H NMR** (400 MHz, $CDCl_3$): δ 3.06 – 2.55 (m, 6H), 1.91 – 1.12 (m, 15H); **^{13}C NMR** (100 MHz, $CDCl_3$): δ 58.1, 53.1, 52.7, 40.8, 39.2, 32.0, 30.2, 29.8, 26.1, 25.4, 10.1; **HRMS** calcd. for $C_{11}H_{22}N^+$ 168.1747 $[M+H]^+$, found 168.1746.

1-ethyl-4-methyl-3-phenylpiperidine

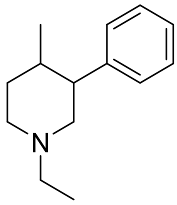

Following General Method 5 with 1-ethyl-4-methyl-5-phenyl-1,2,3,6-THP (**19b**) (0.31 mg, 1.54 mmol) afforded the title compound as a pale-yellow oil (0.22 g, 1.08 mmol, 70%). **^{1}H NMR** (400 MHz, $CDCl_3$): δ 7.32 – 7.25 (m, 4H), 7.24 – 7.15 (m, 1H), 3.11 – 3.02 (m, 1H), 2.78 – 2.70 (m, 1H), 2.66 – 2.53 (m, 2H), 2.47 (q, *J* = 7.2 Hz, 2H), 2.37 (s, 1H), 2.13 – 2.02 (m, 1H), 1.90 (s, 1H), 1.65 – 1.54 (m, 1H), 1.11 (t, *J* = 7.2 Hz, 3H), 0.71 (d, *J* = 7.2 Hz, 3H); **^{13}C NMR** (100 MHz, $CDCl_3$): δ 142.6, 128.2, 128.0, 126.3, 58.3, 52.9, 44.2, 32.3, 31.4, 29.8, 18.5, 11.6; **HRMS** calcd. for $C_{14}H_{22}N^+$ 204.1747 $[M+H]^+$, found 204.1746.

4-methyl-3-phenyl-1-propylpiperidine

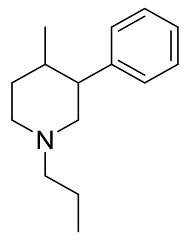

Following General Method 5 4-methyl-5-phenyl-1-propyl-1,2,3,6-THP (**20b**) (0.50 mg, 2.32 mmol) afforded the title compound as a pale-yellow oil (0.42 g, 2.32 mmol, 83%). **^{1}H NMR** (400 MHz, $CDCl_3$): δ 7.39 – 7.13 (m, 5H), 3.07 – 2.97 (m, 1H), 2.70 – 2.60 (m, 1H), 2.54 – 2.38 (m, 2H), 2.39 – 2.25 (m, 2H), 2.08 – 2.00 (m, 1H), 1.78 (d, *J* = 43.1 Hz, 2H), 1.63 – 1.47 (m, 3H), 0.91 (t, *J* = 7.4 Hz, 3H), 0.70 (d, *J* = 7.1 Hz, 3H); **^{13}C NMR** (100 MHz, $CDCl_3$): δ 143.8, 128.7, 127.9, 126.0, 61.4, 54.4, 51.0, 45.3, 33.1, 31.9, 20.4, 19.9, 12.2.

14. Compound ^{1}H NMR and ^{13}C NMR Spectra

^{1}H & ^{13}C NMR spectra for 1-ethyl-3-methyl-1,2,5,6-THP-2-carbonitrile (25)

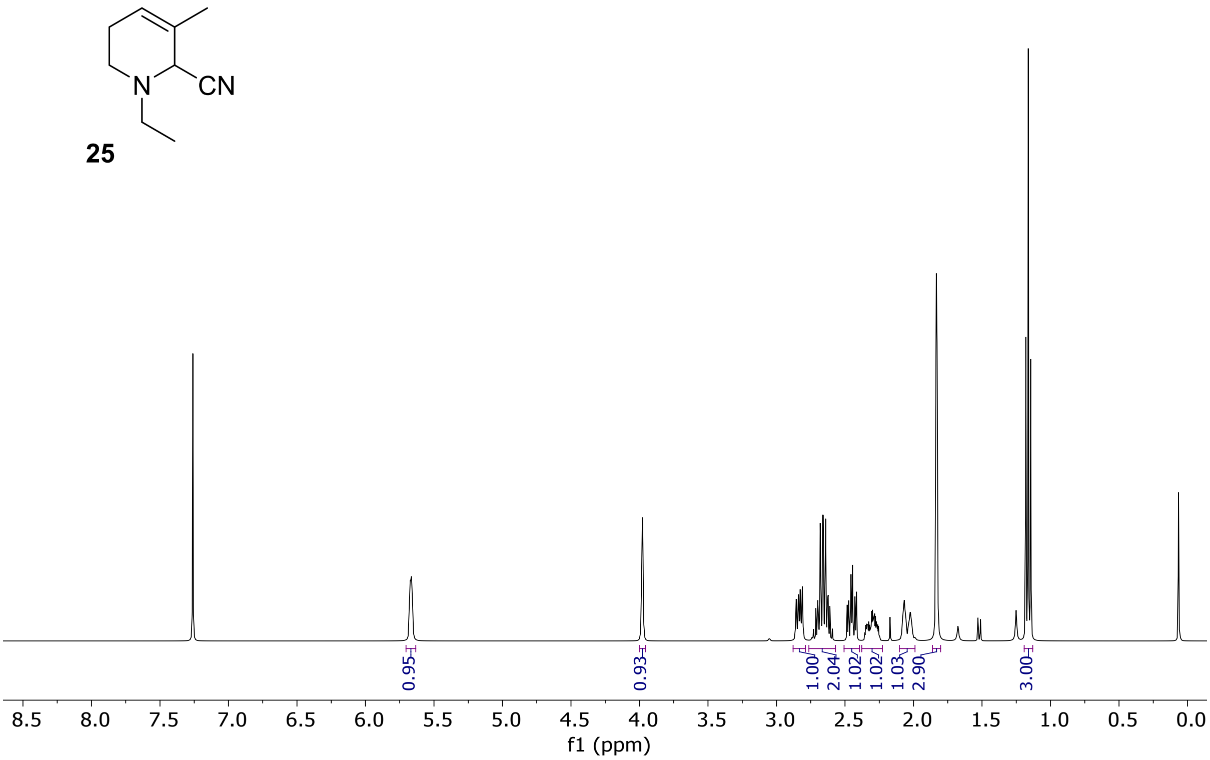

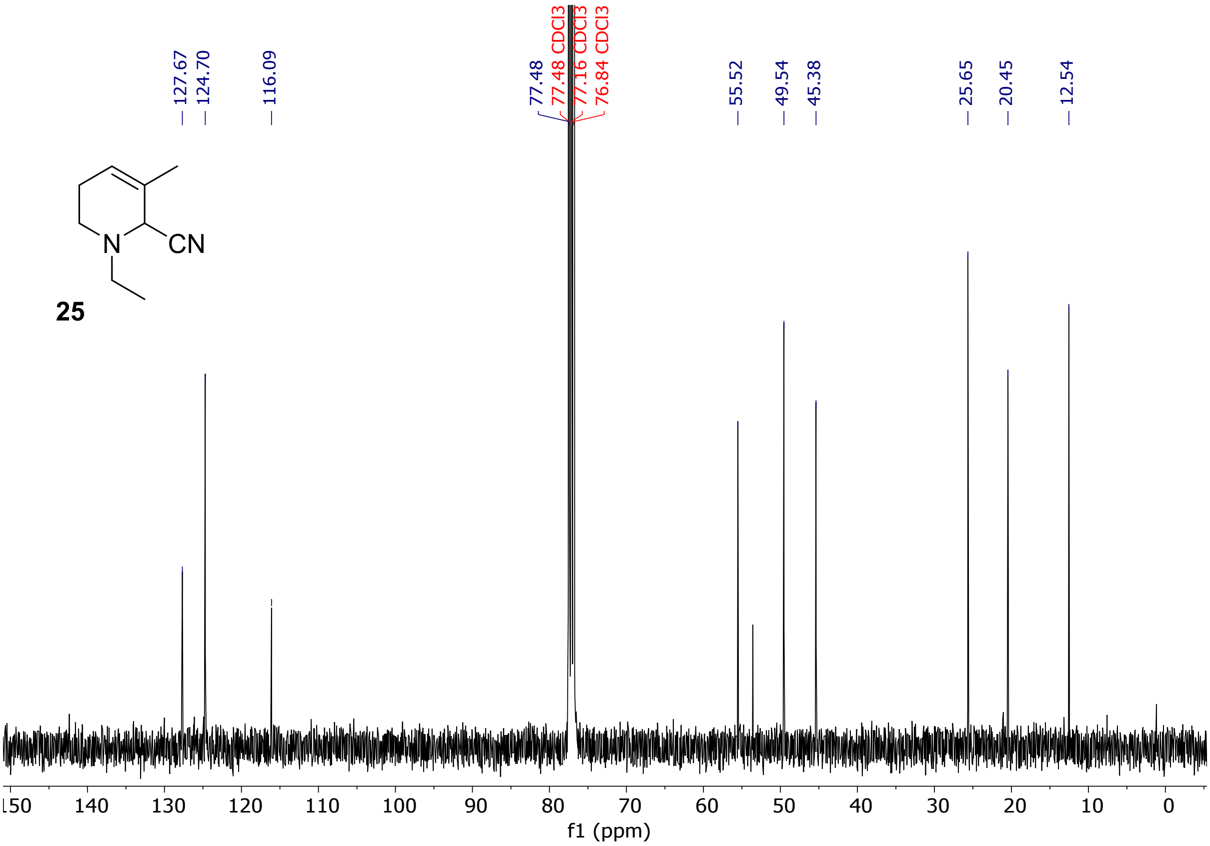

^{1}H & ^{13}C NMR spectra for (*R*)-3-(3-methoxyphenyl)-1-propylpiperidine (1c)

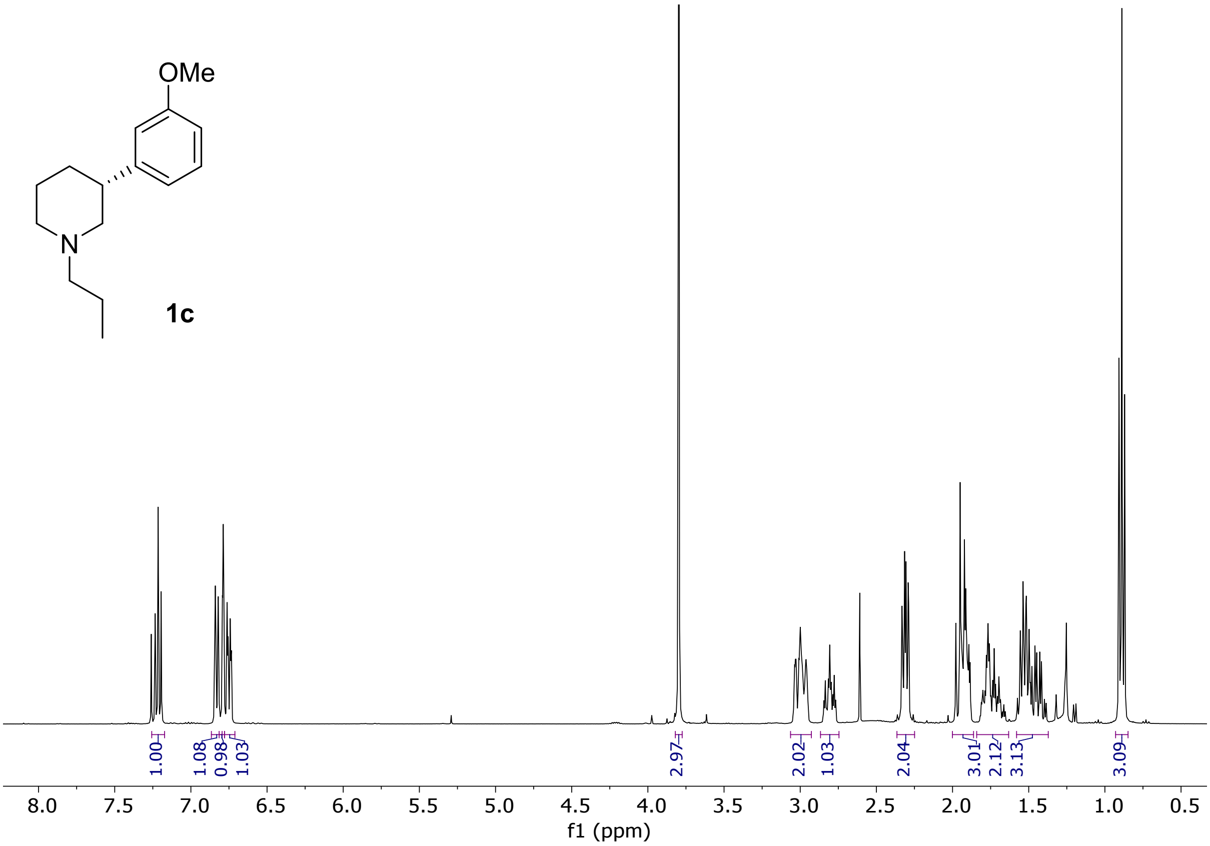

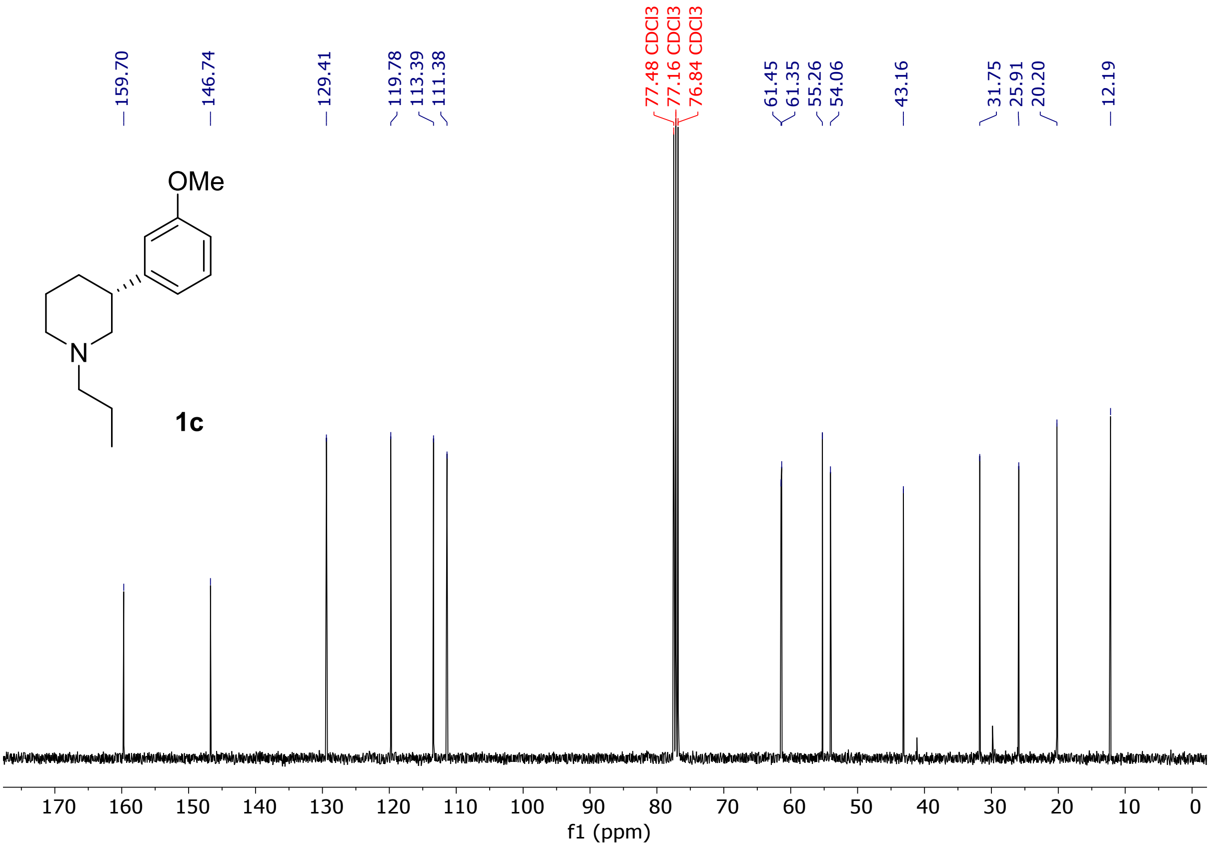

^{1}H & ^{13}C NMR spectra for (*R*)-3-(1-propylpiperidin-3-yl)phenol (22)

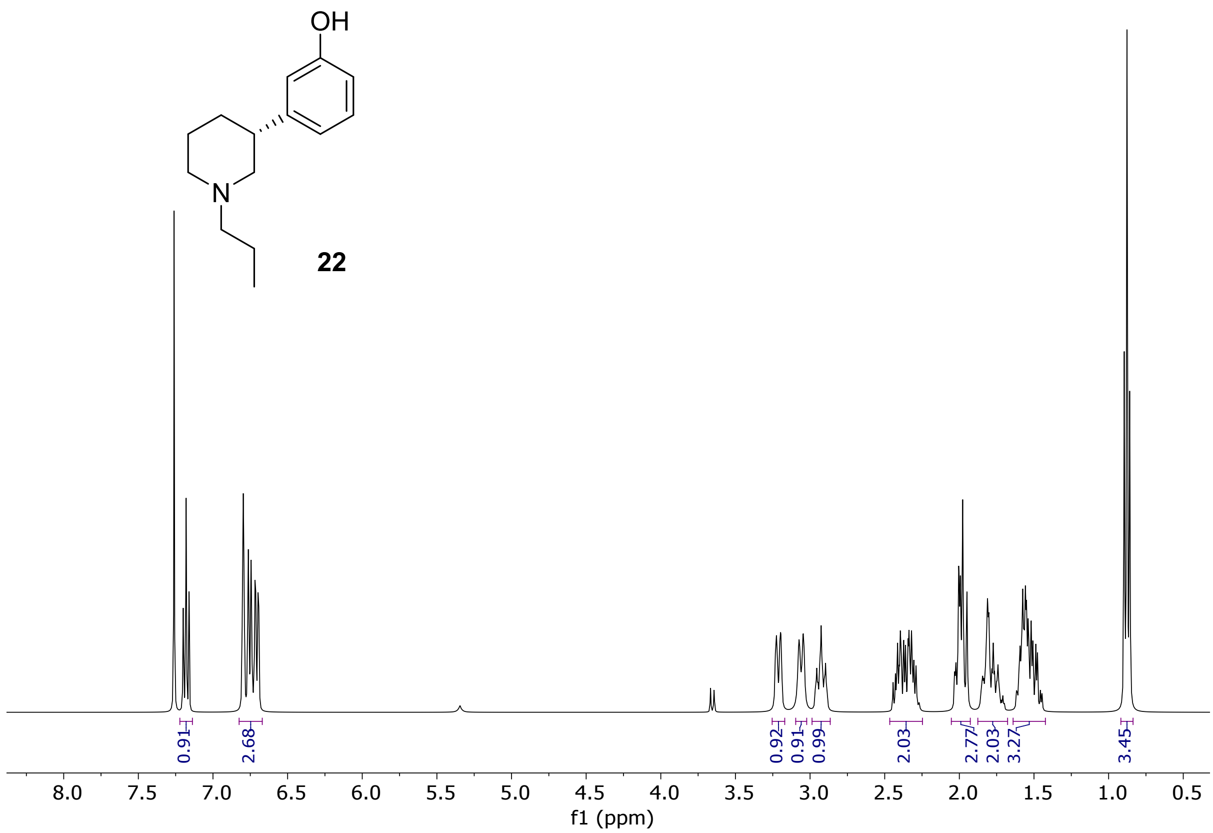

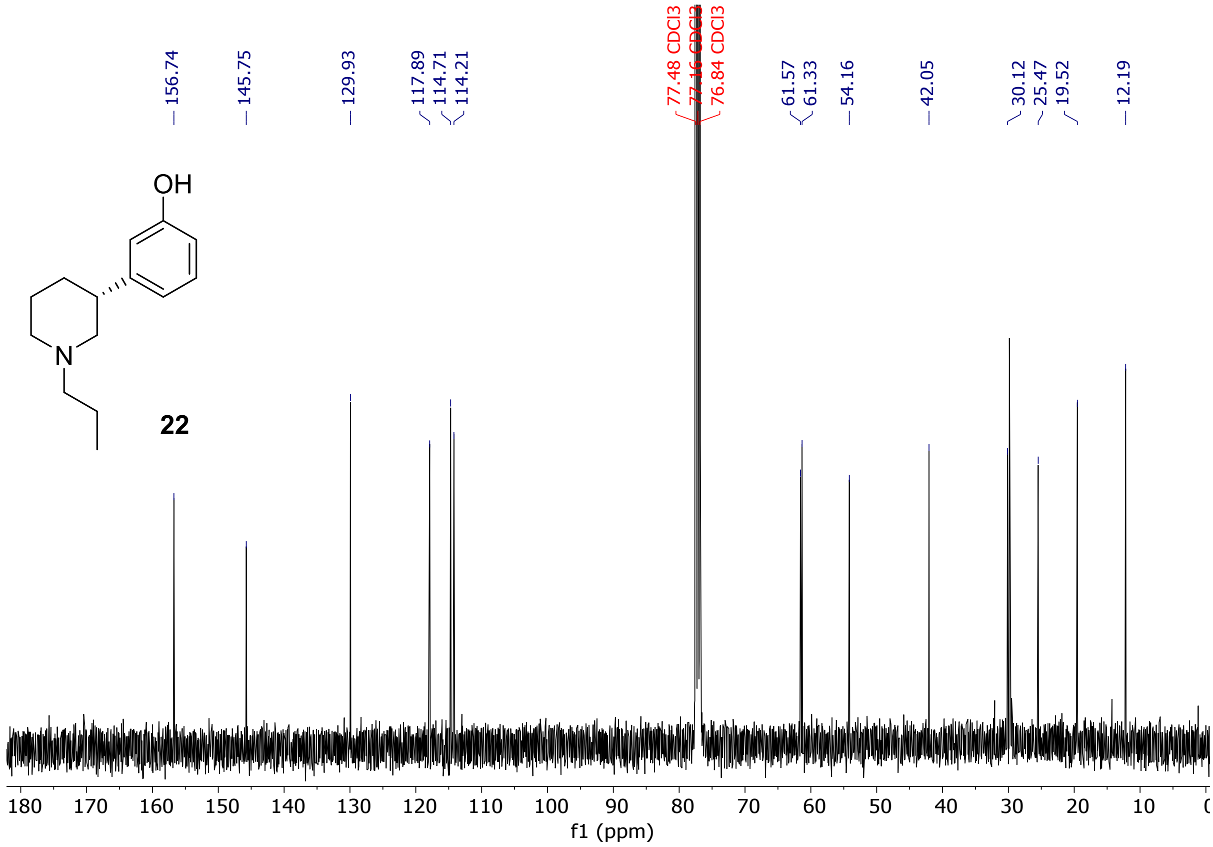

^{1}H & ^{13}C NMR spectra for (*R*)-3-(3-(methylsulfonyl)phenyl)-1-propylpiperidine (2c)

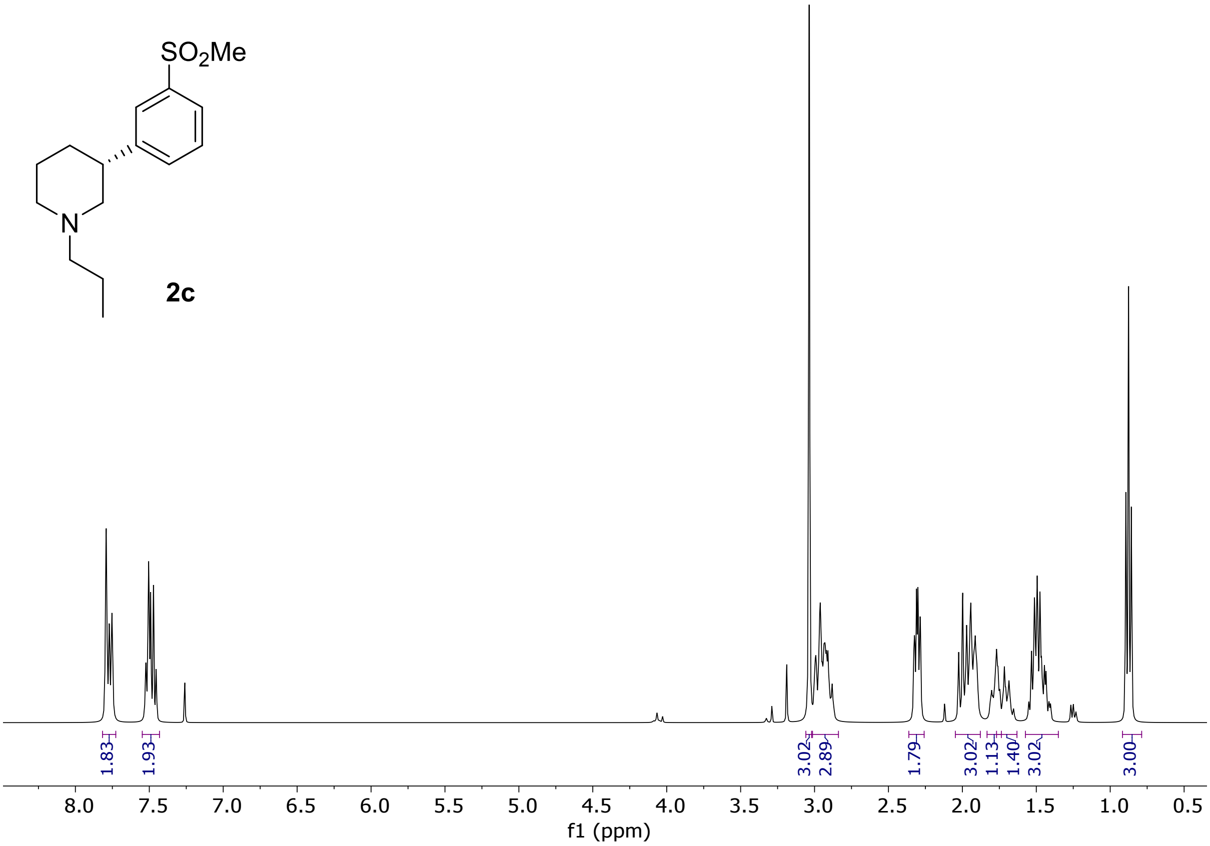

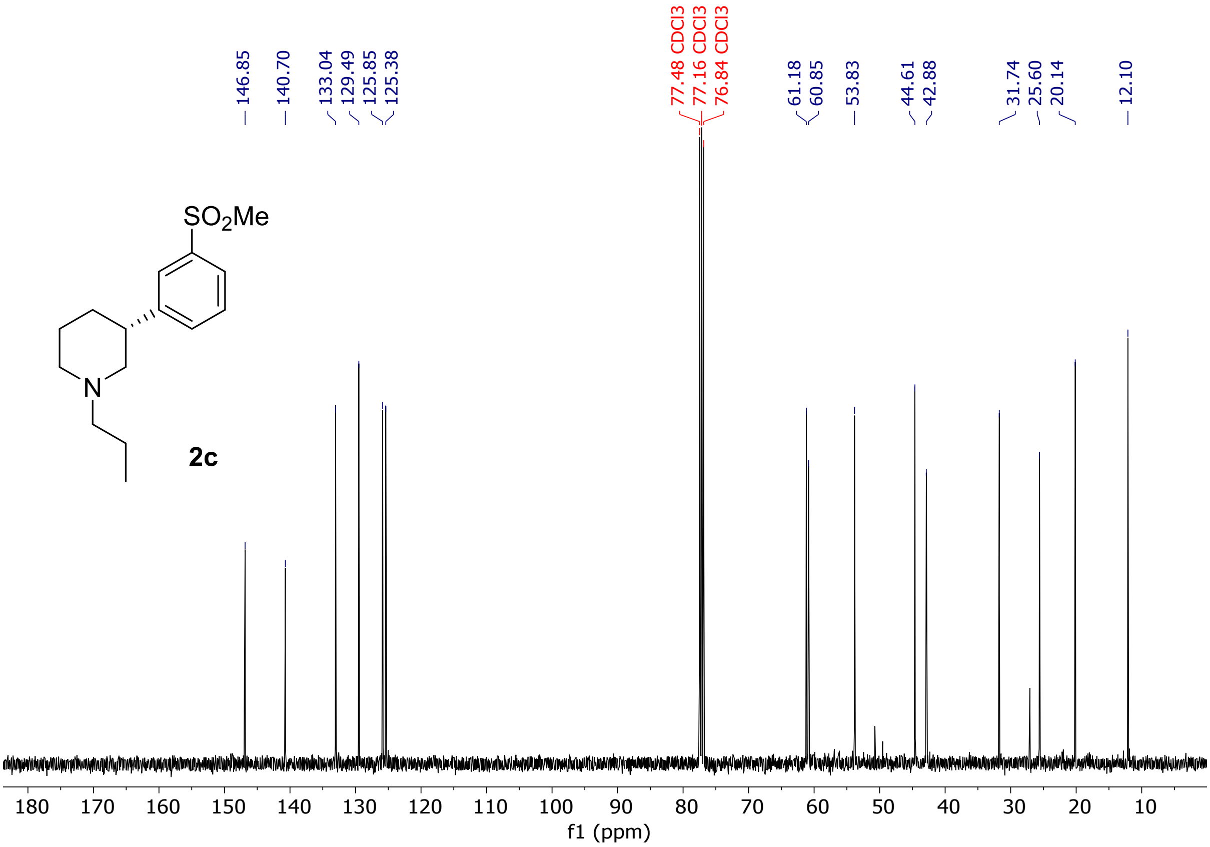

^{1}H & ^{13}C NMR spectra for (*R*)-1-allyl-3-(4-bromophenyl)piperidine (3c)

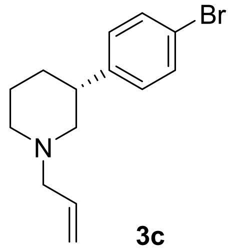

3c

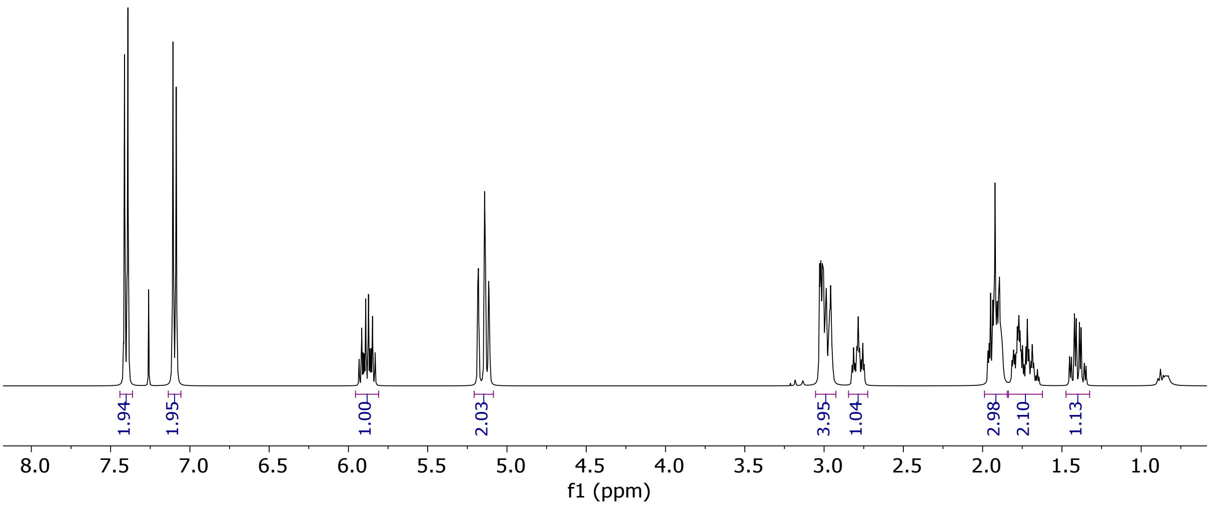

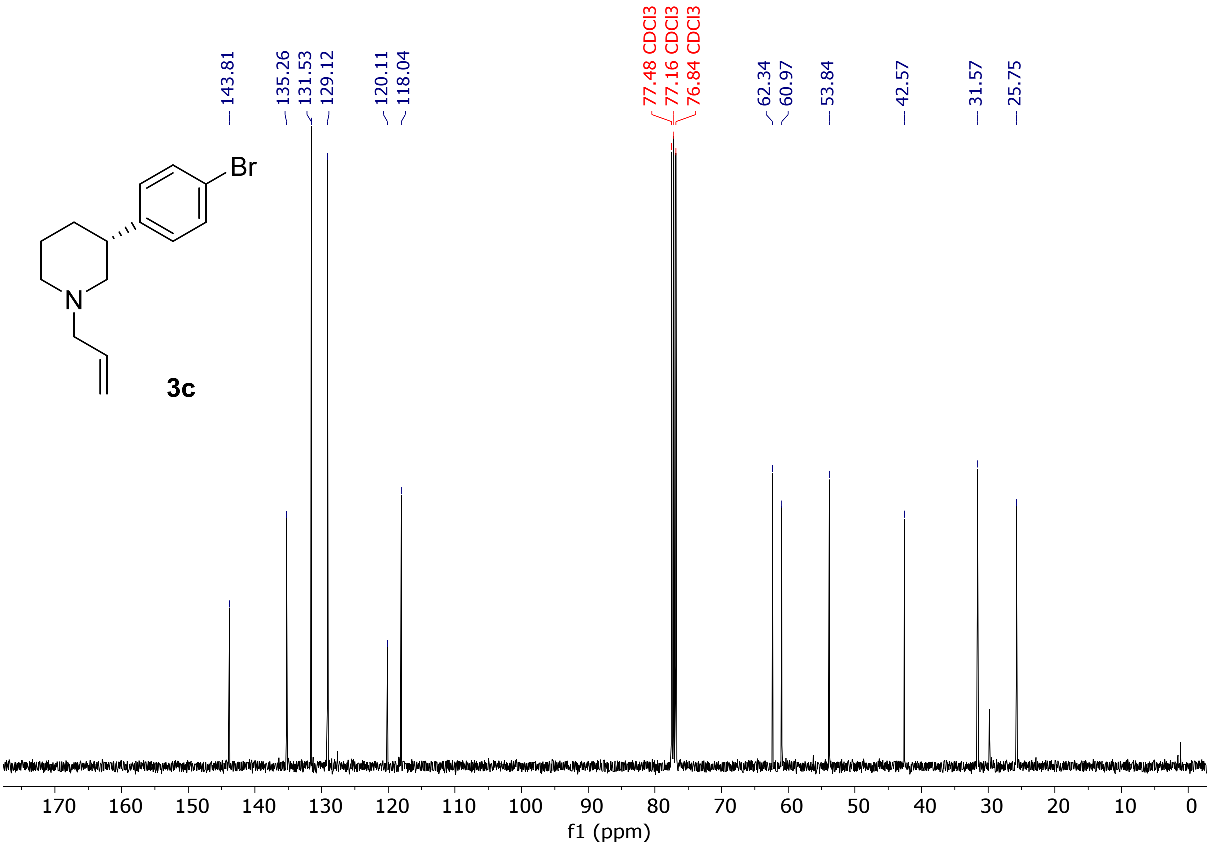

3c

^{1}H & ^{13}C NMR spectra for (*R*)-3-(4-bromophenyl)piperidine (Crude-26)

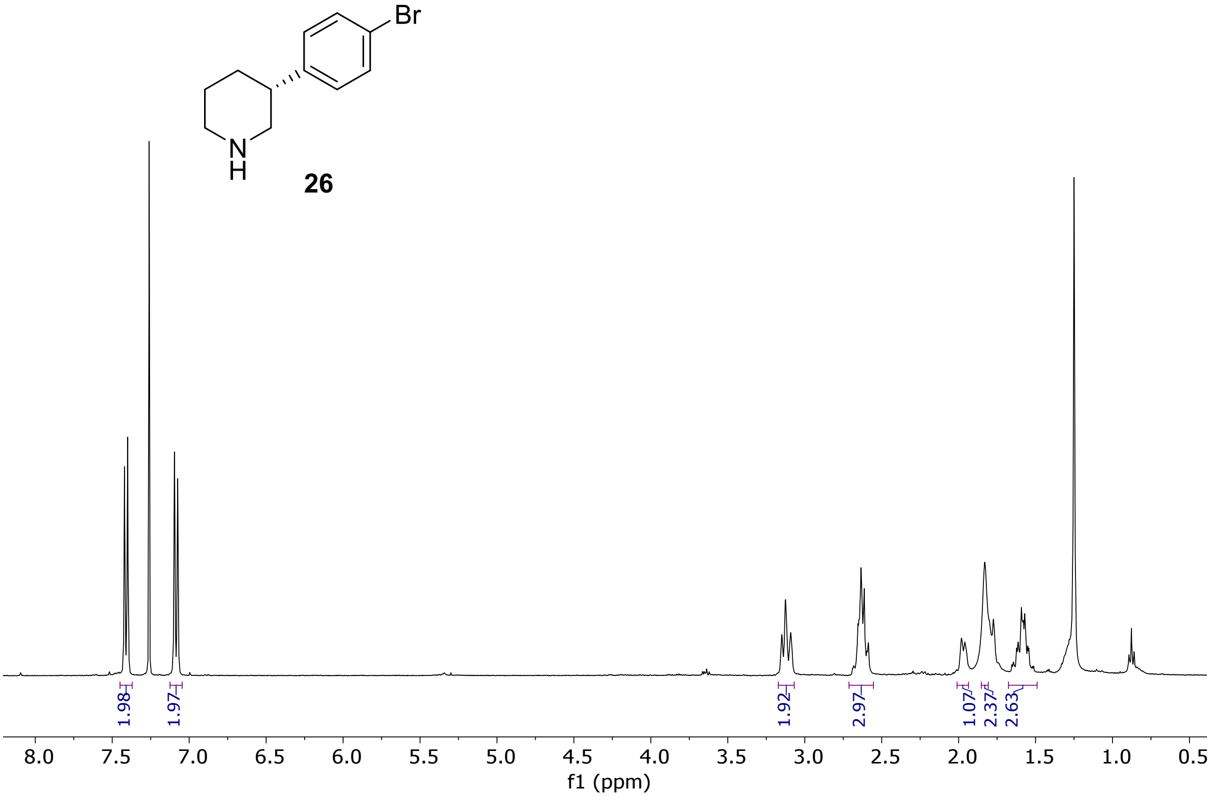

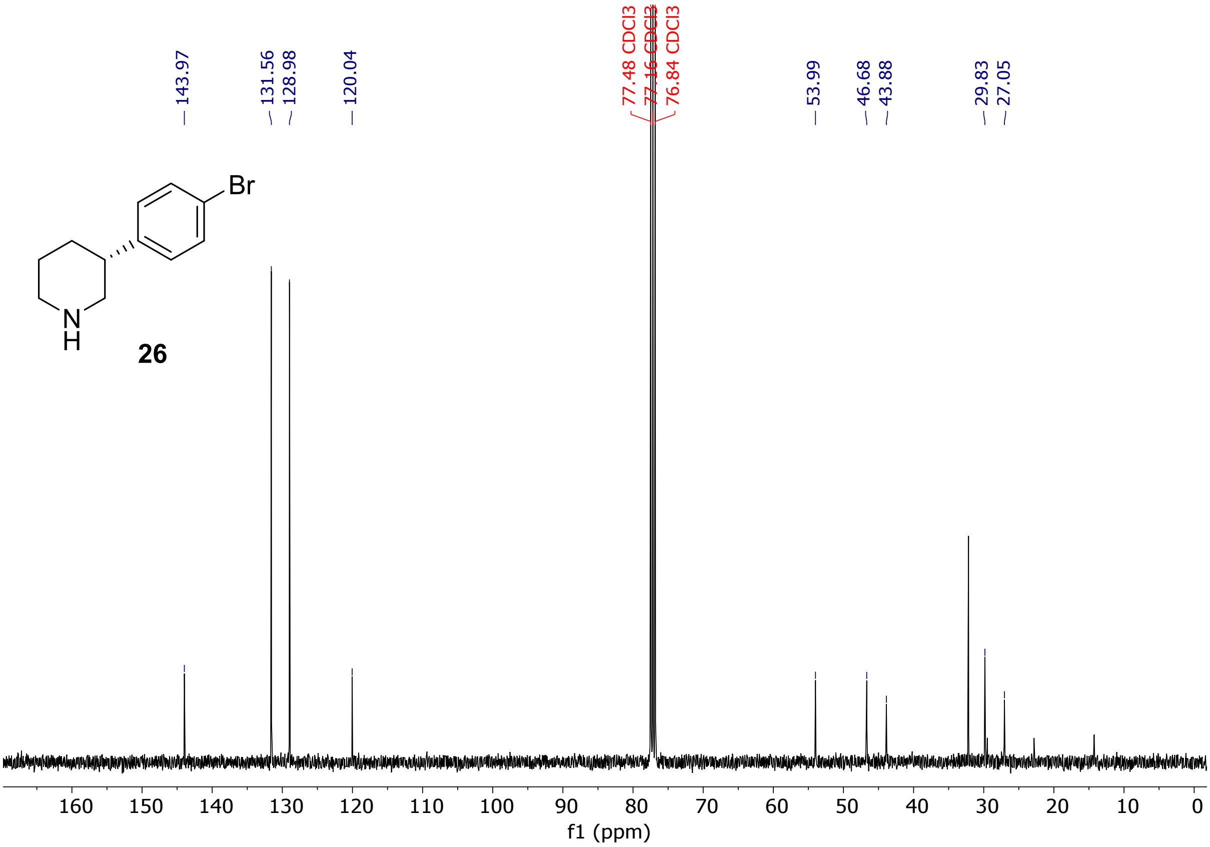

^{1}H & ^{13}C NMR spectra for *tert*-butyl (*R*)-3-(4-bromophenyl)piperidine-1-carboxylate (23)

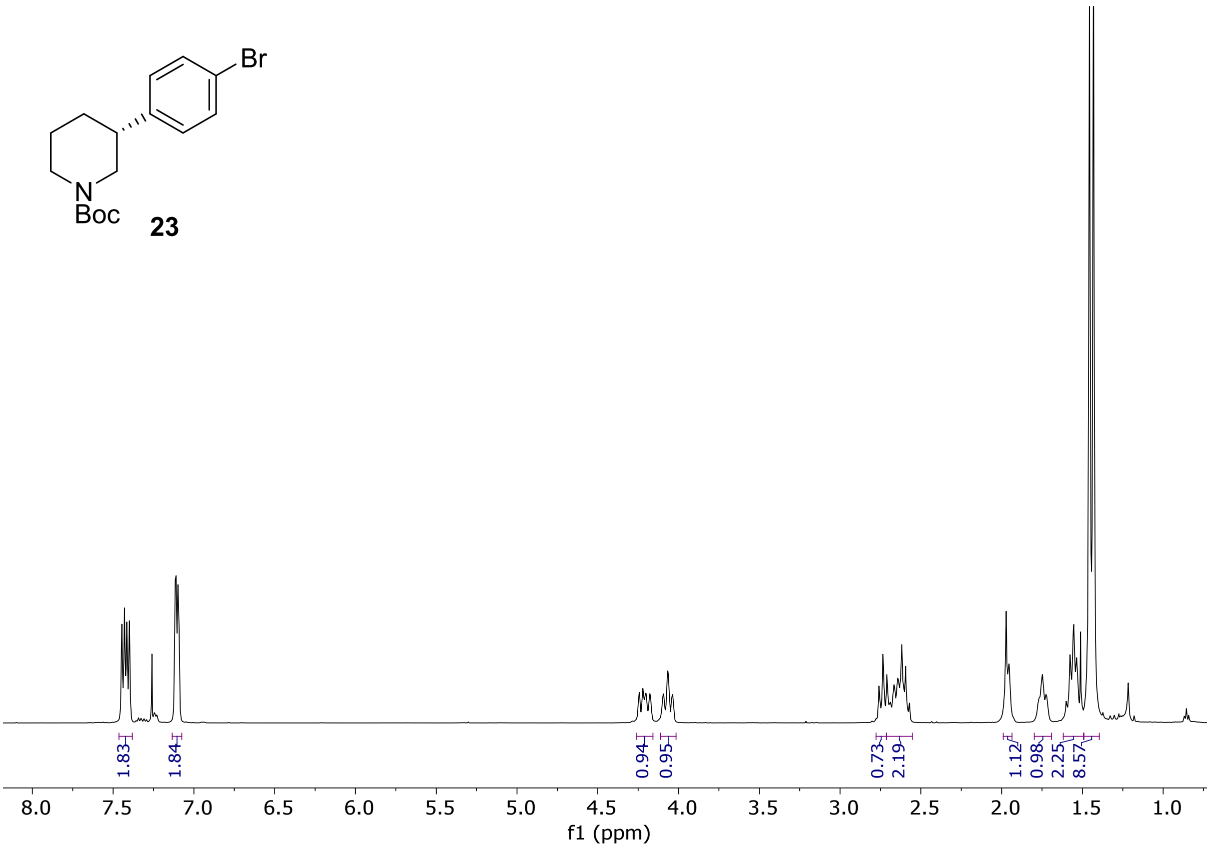

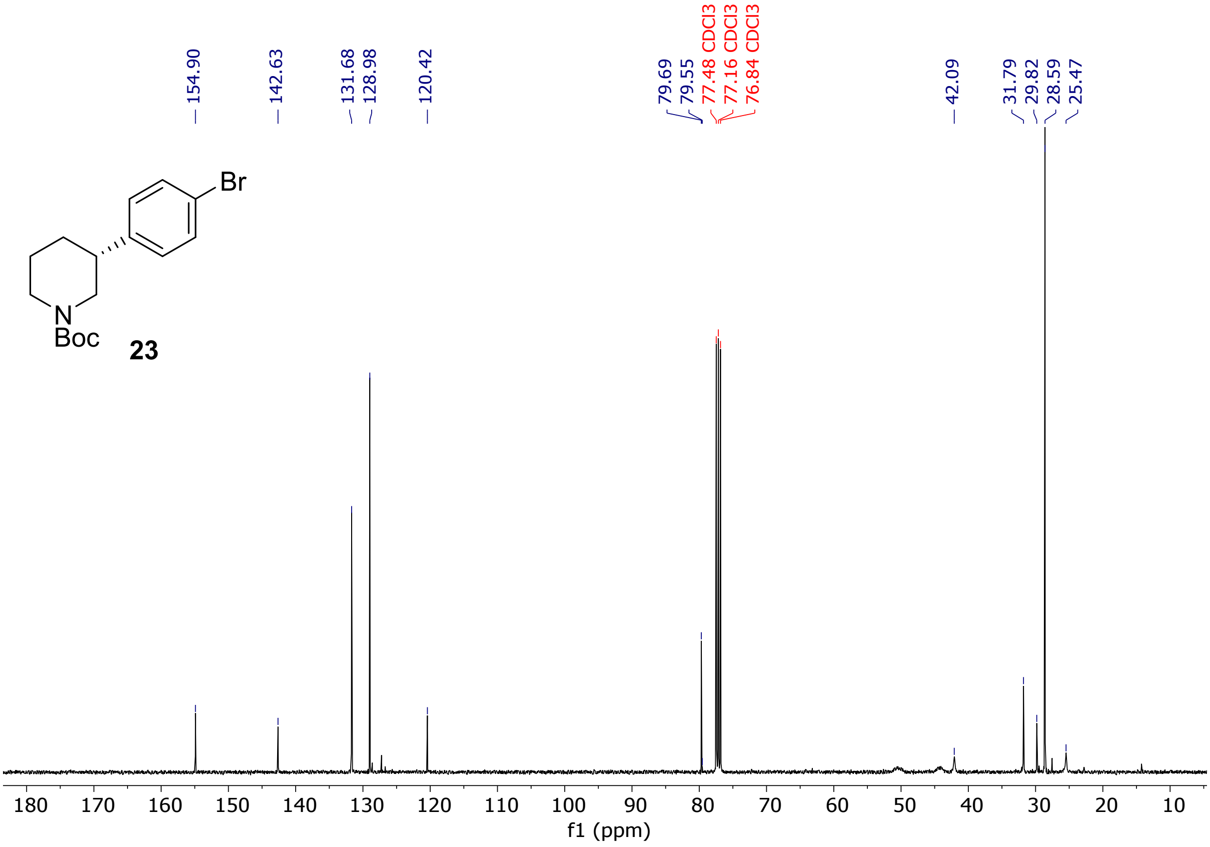

^{1}H & ^{13}C NMR spectra for (*R*)-4-(1-allylpiperidin-3-yl)aniline (4c)

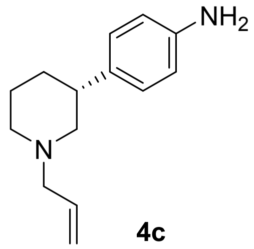

4c

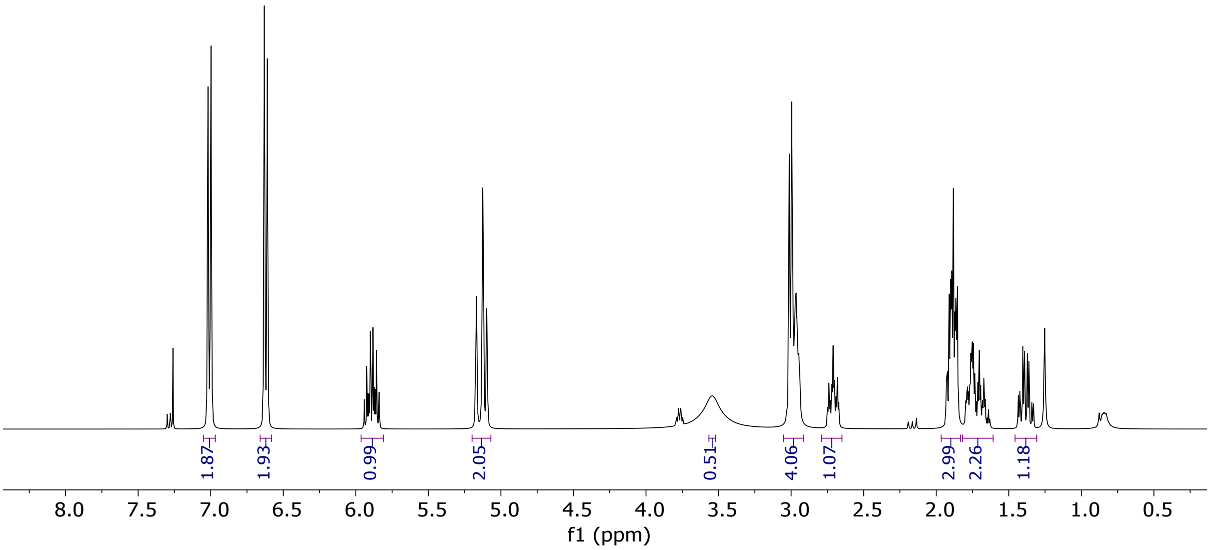

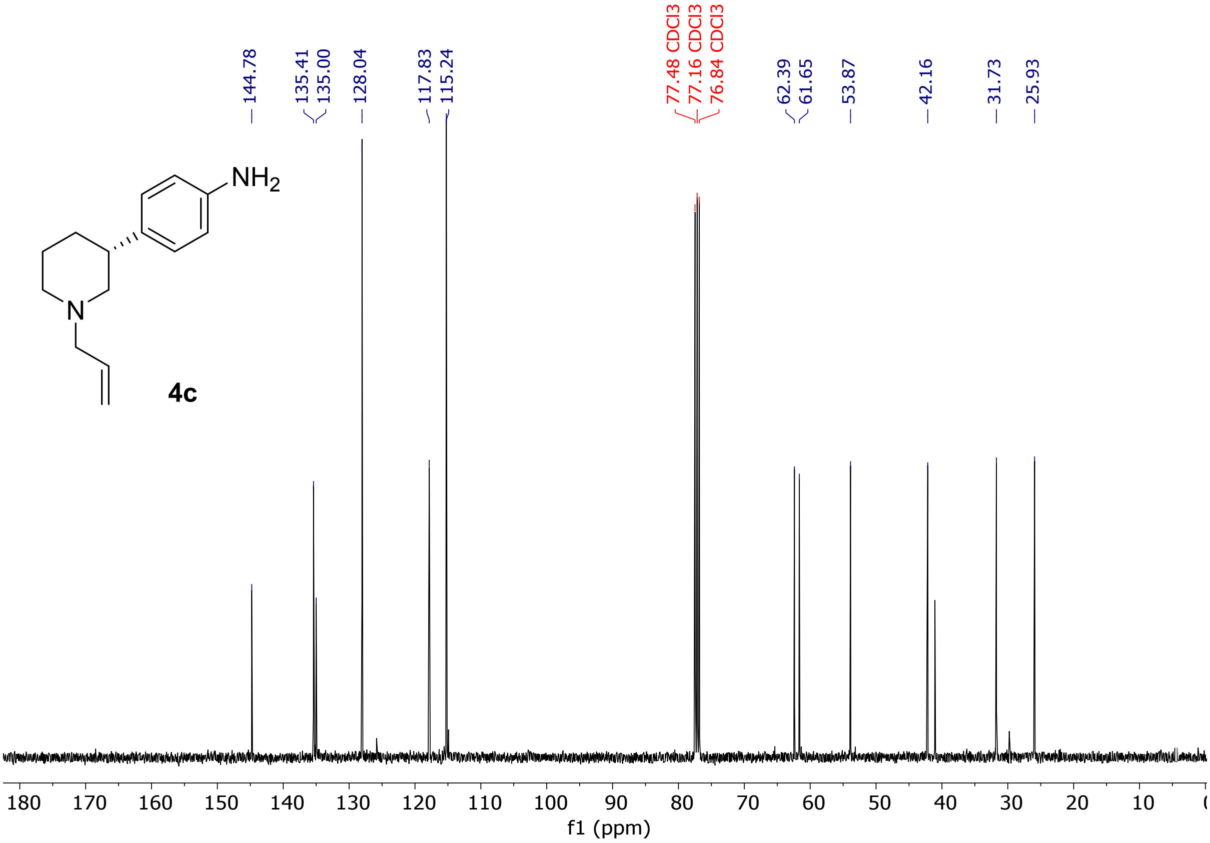

^{1}H & ^{13}C NMR spectra for (*R*)-4-(piperidin-3-yl)aniline (Crude-27)

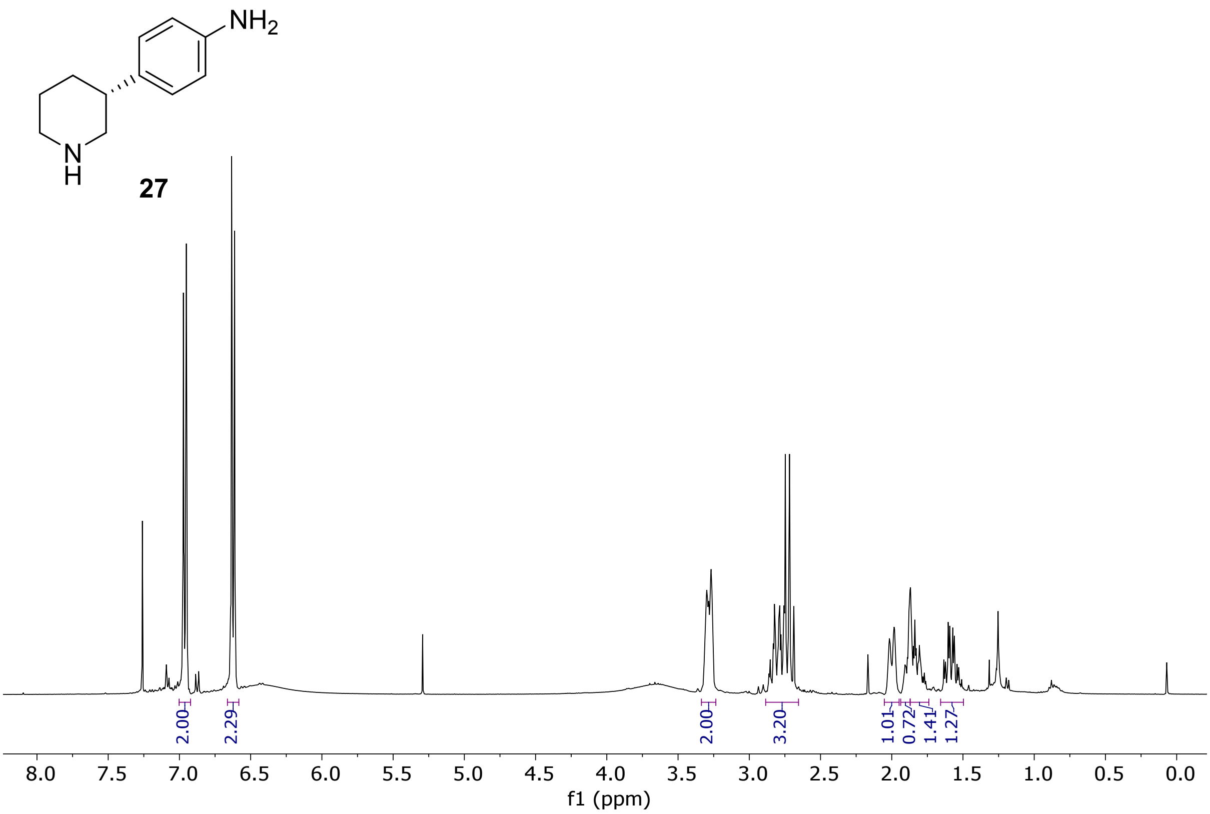

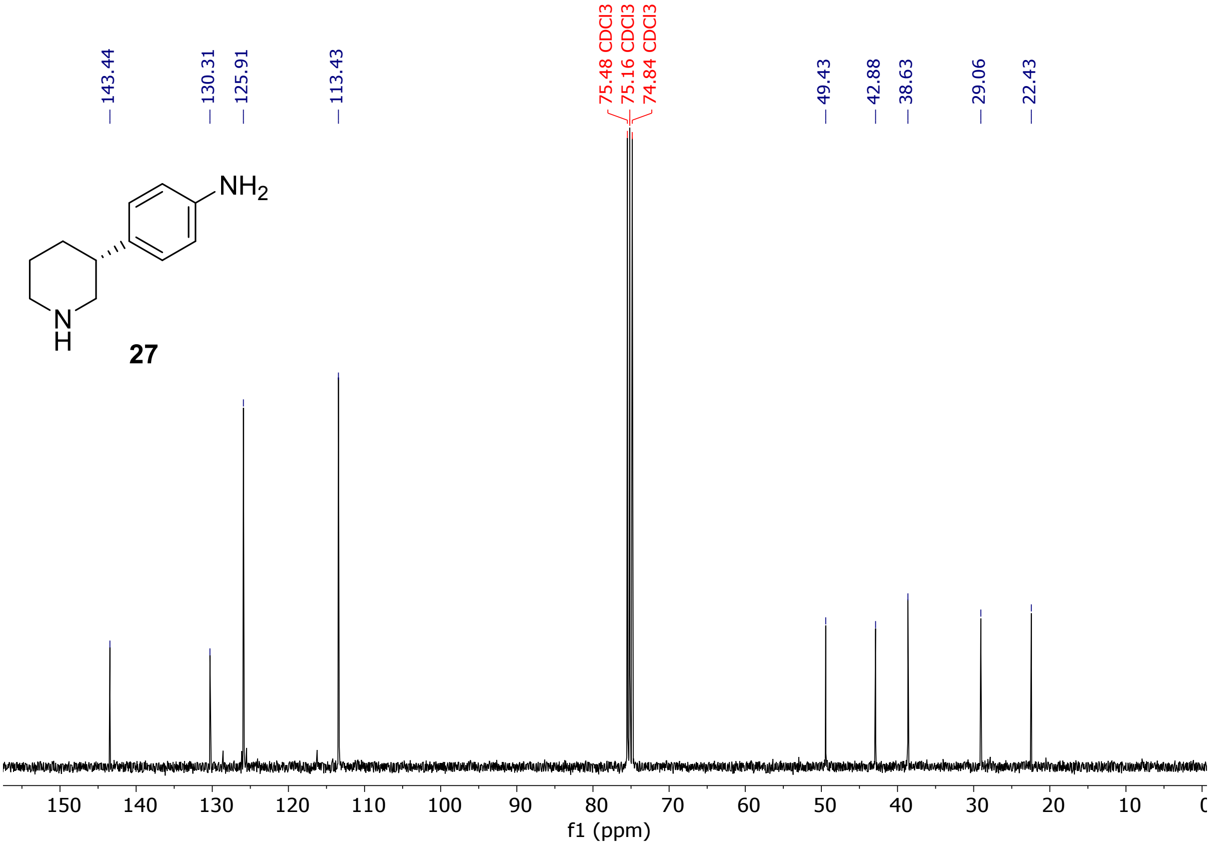

^{1}H & ^{13}C NMR spectra for *tert*-butyl (*R*)-3-(4-aminophenyl)piperidine-1-carboxylate (24)

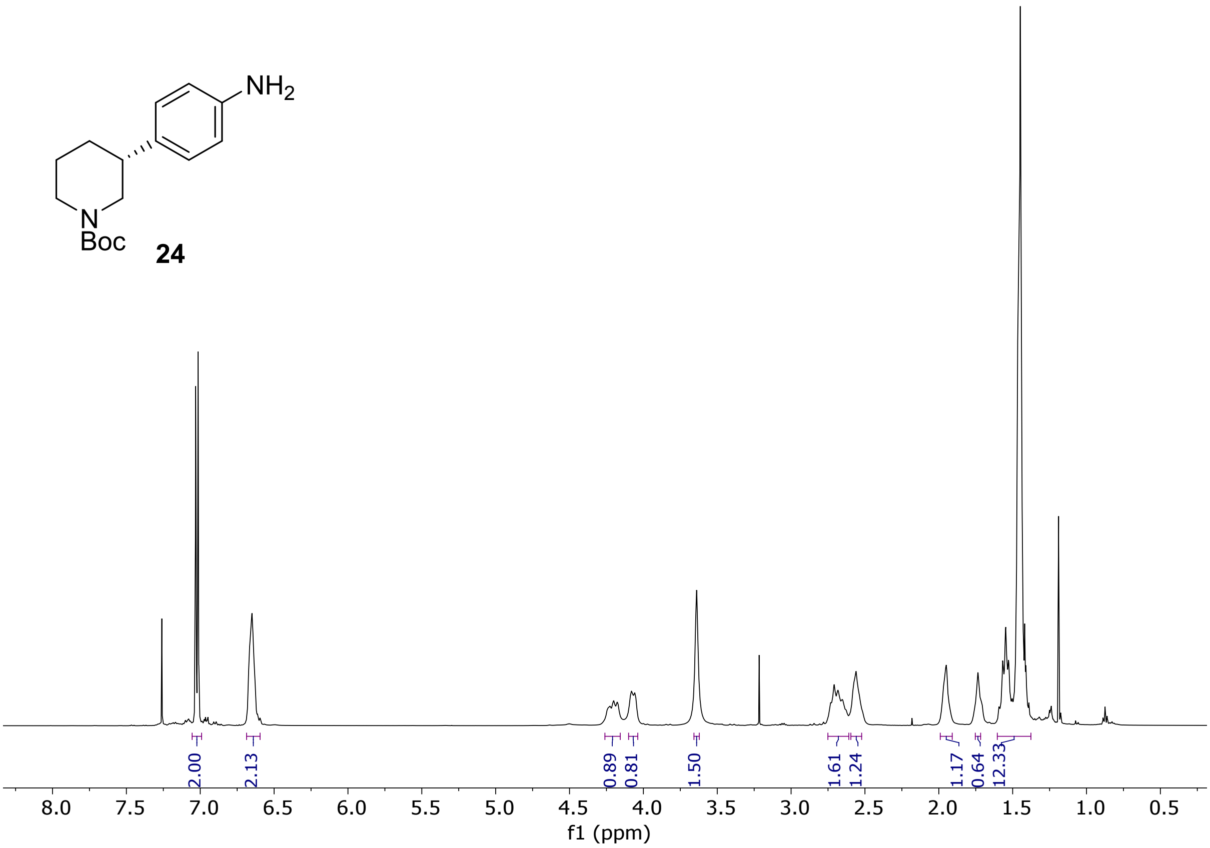

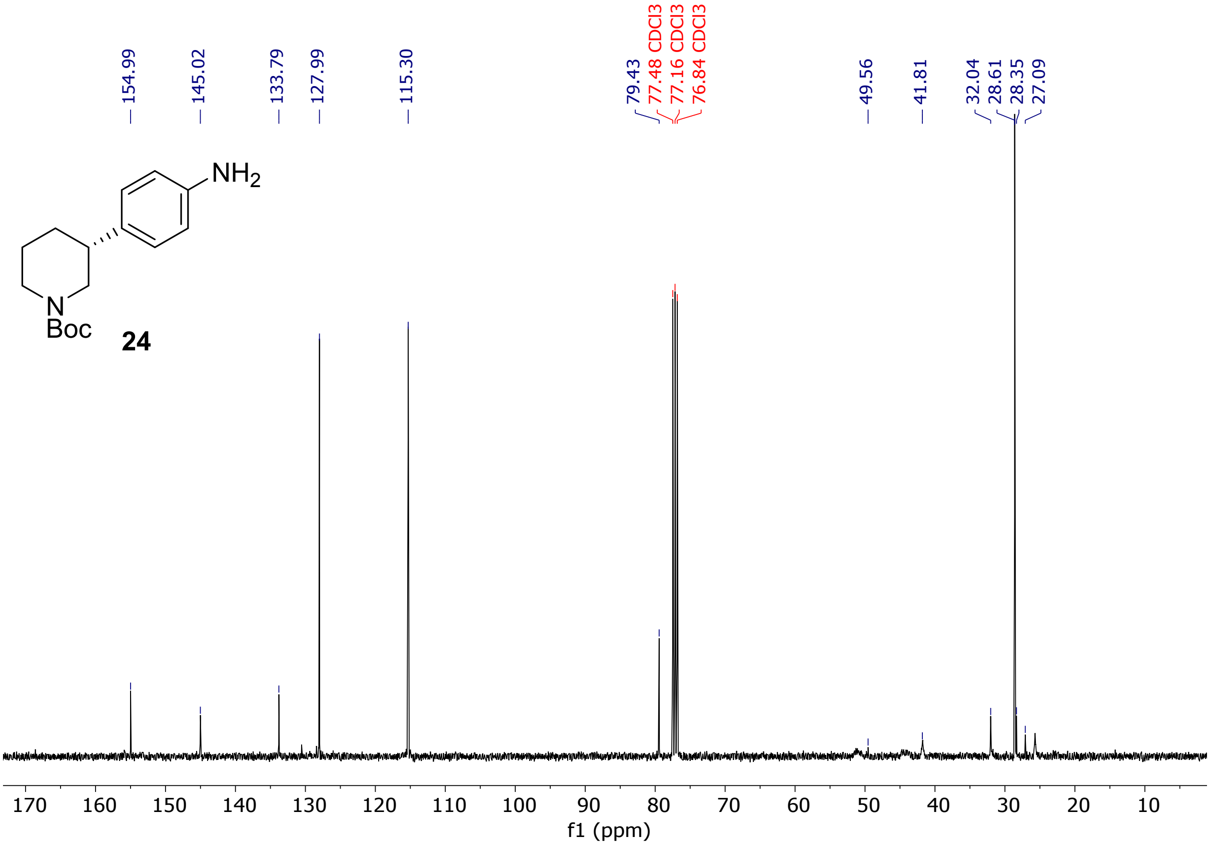

^{1}H & ^{13}C NMR spectra for (*R*)-1-ethyl-3-phenylpiperidine (5c)

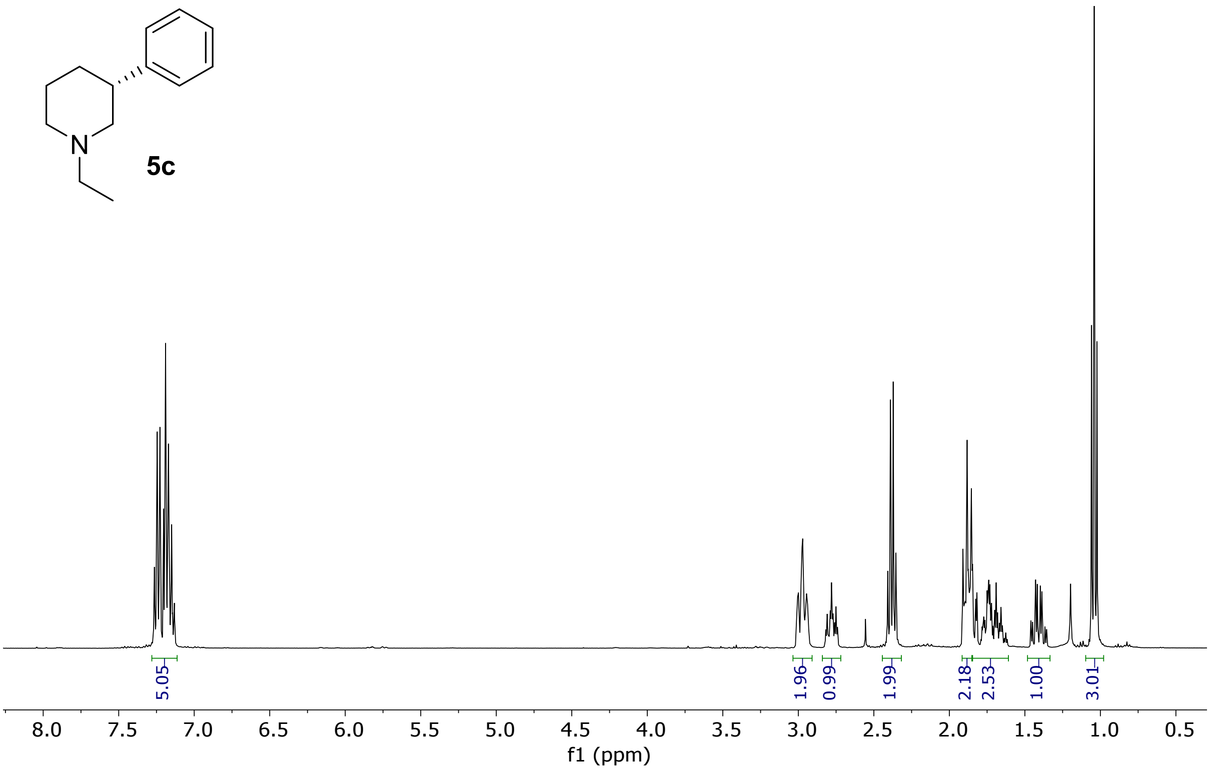

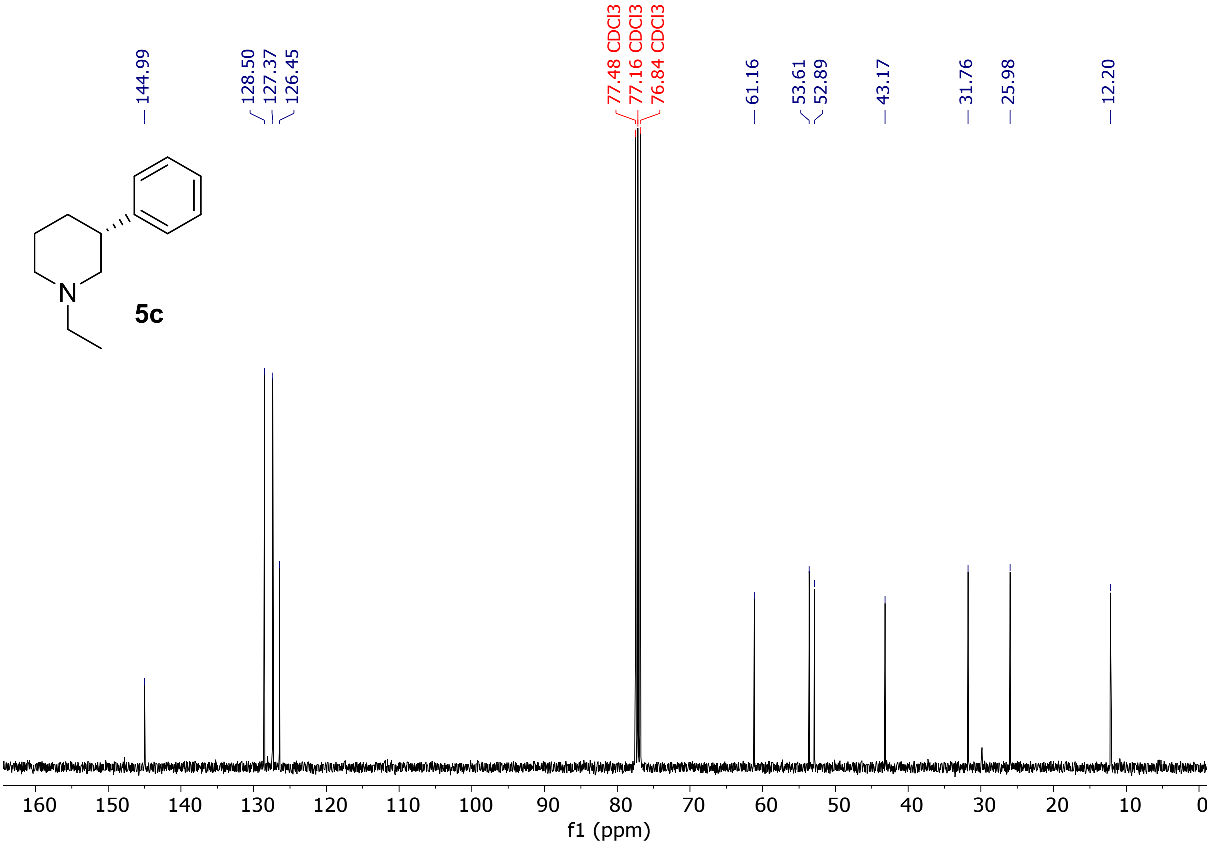

^{1}H & ^{13}C NMR spectra for (*R*)-1-ethyl-3-(4-fluorophenyl)piperidine (6c)

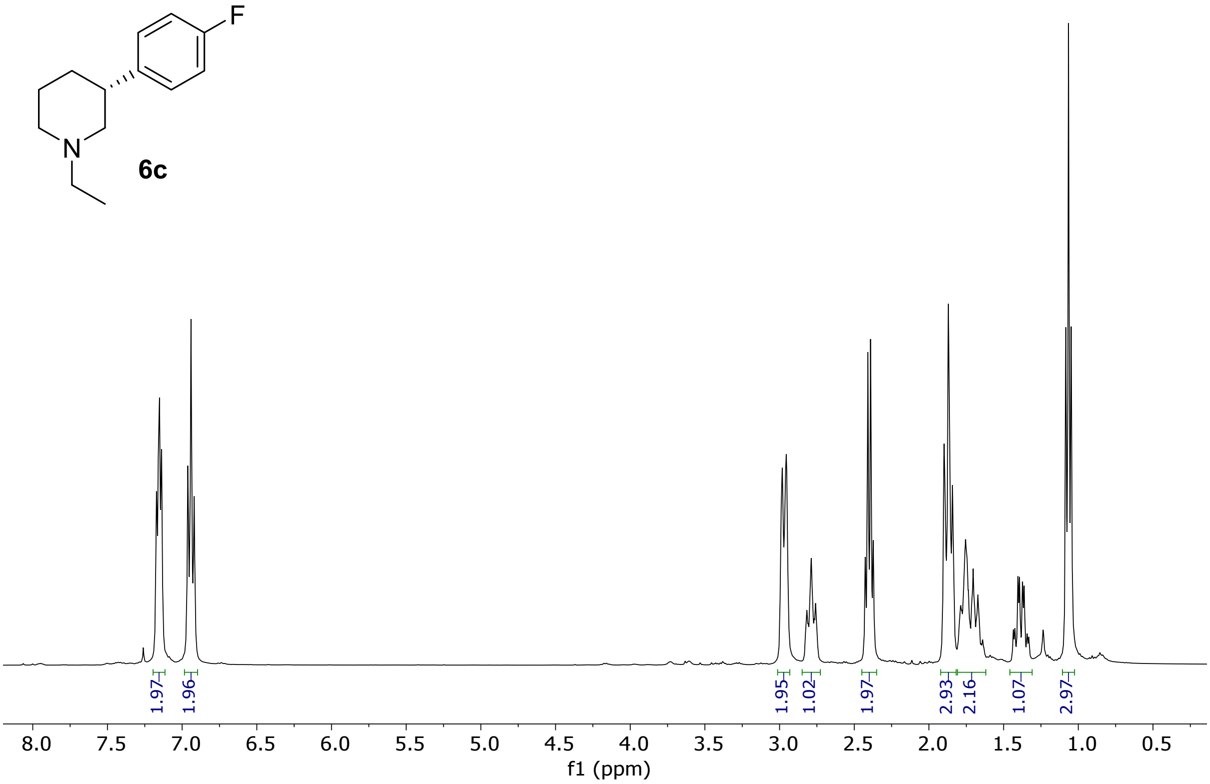

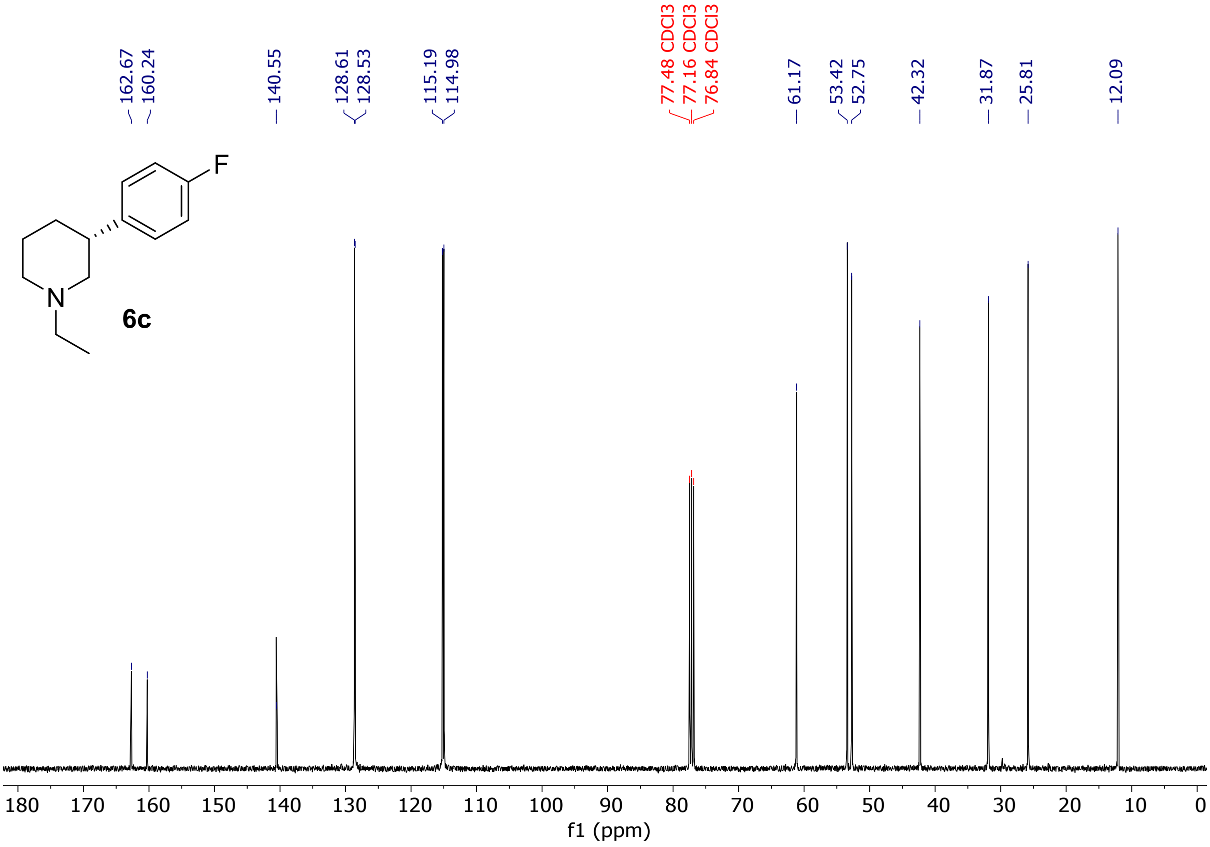

^{1}H & ^{13}C NMR spectra for (*R*)-1-ethyl-3-(4-methoxyphenyl)piperidine (7c)

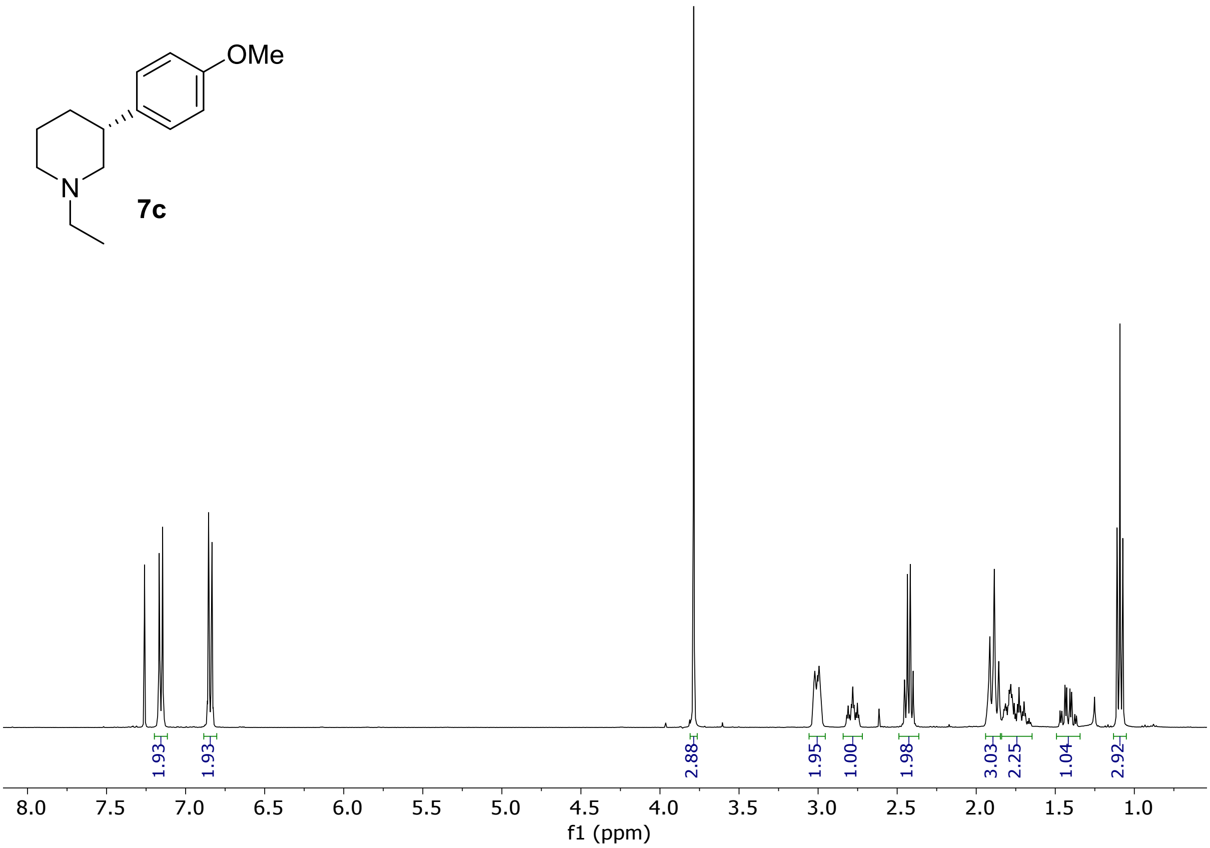

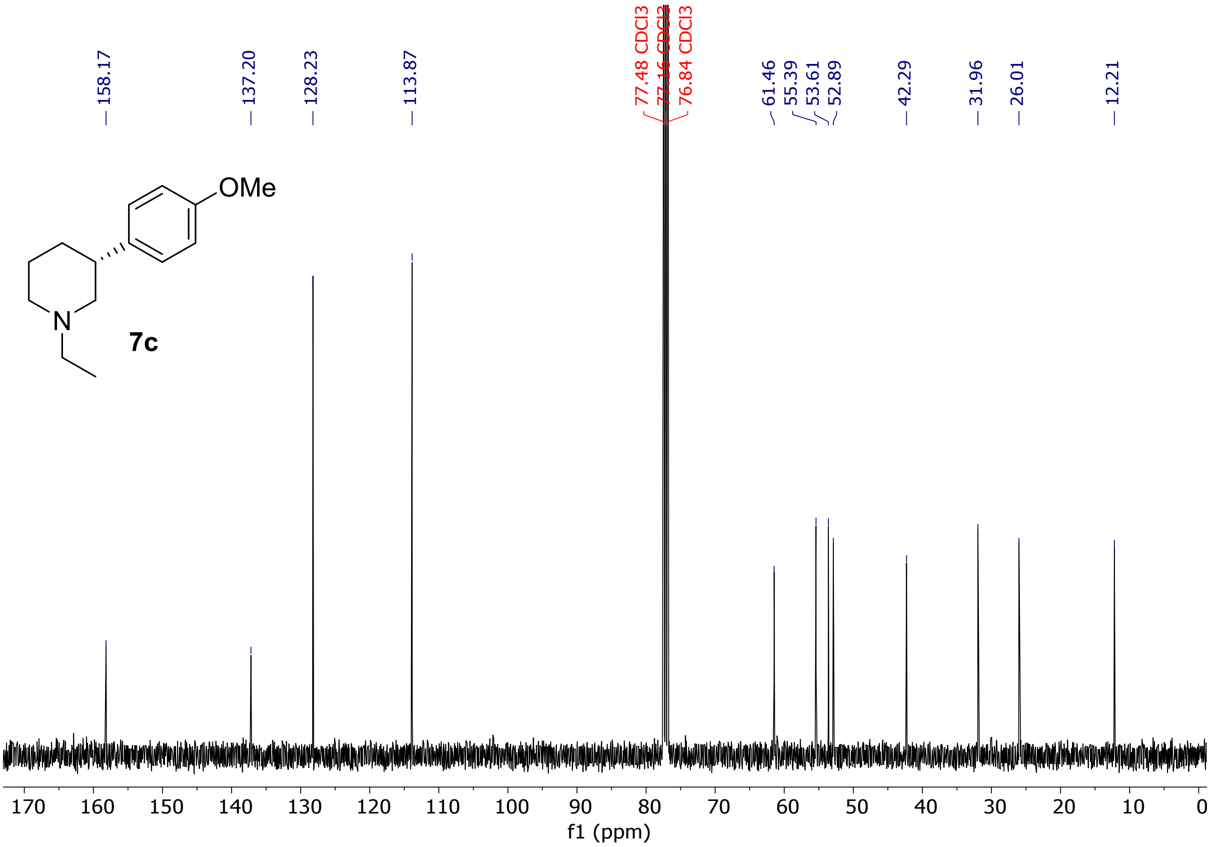

^{1}H & ^{13}C NMR spectra for (*S*)-1-ethyl-3-(furan-2-yl)piperidine (8c)

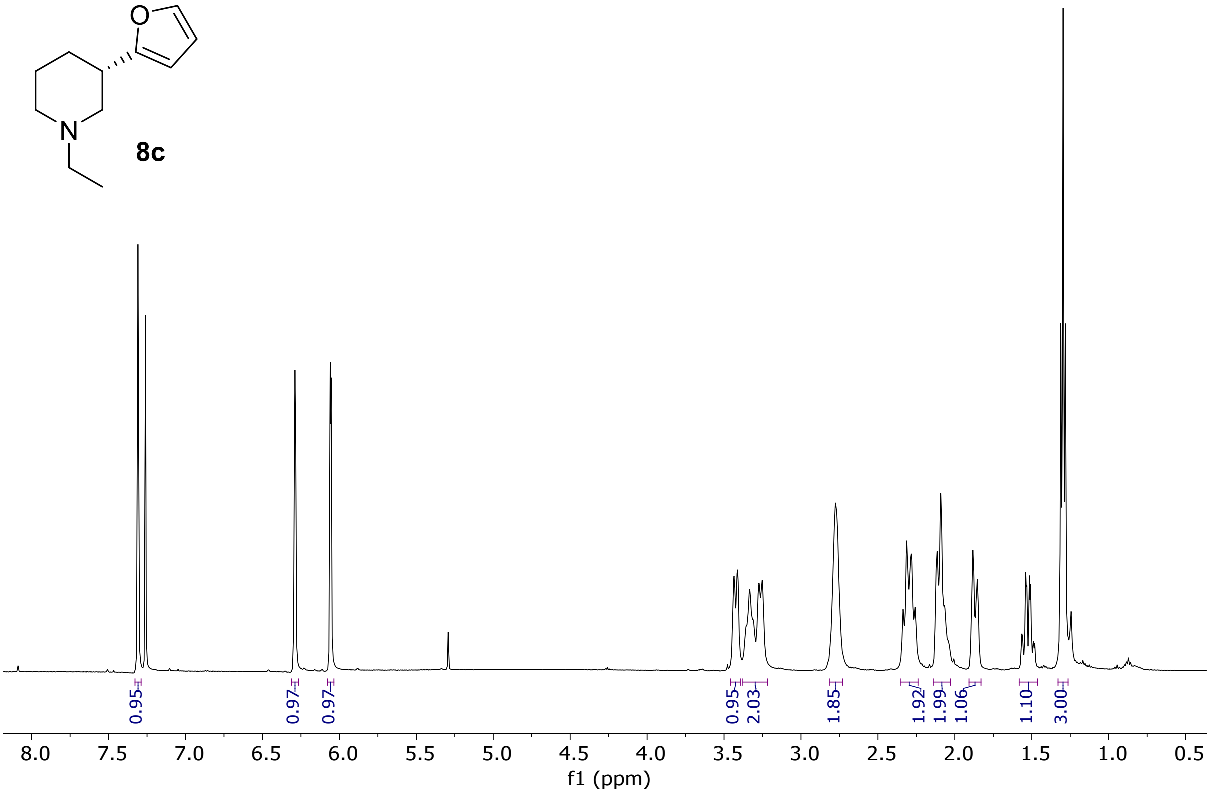

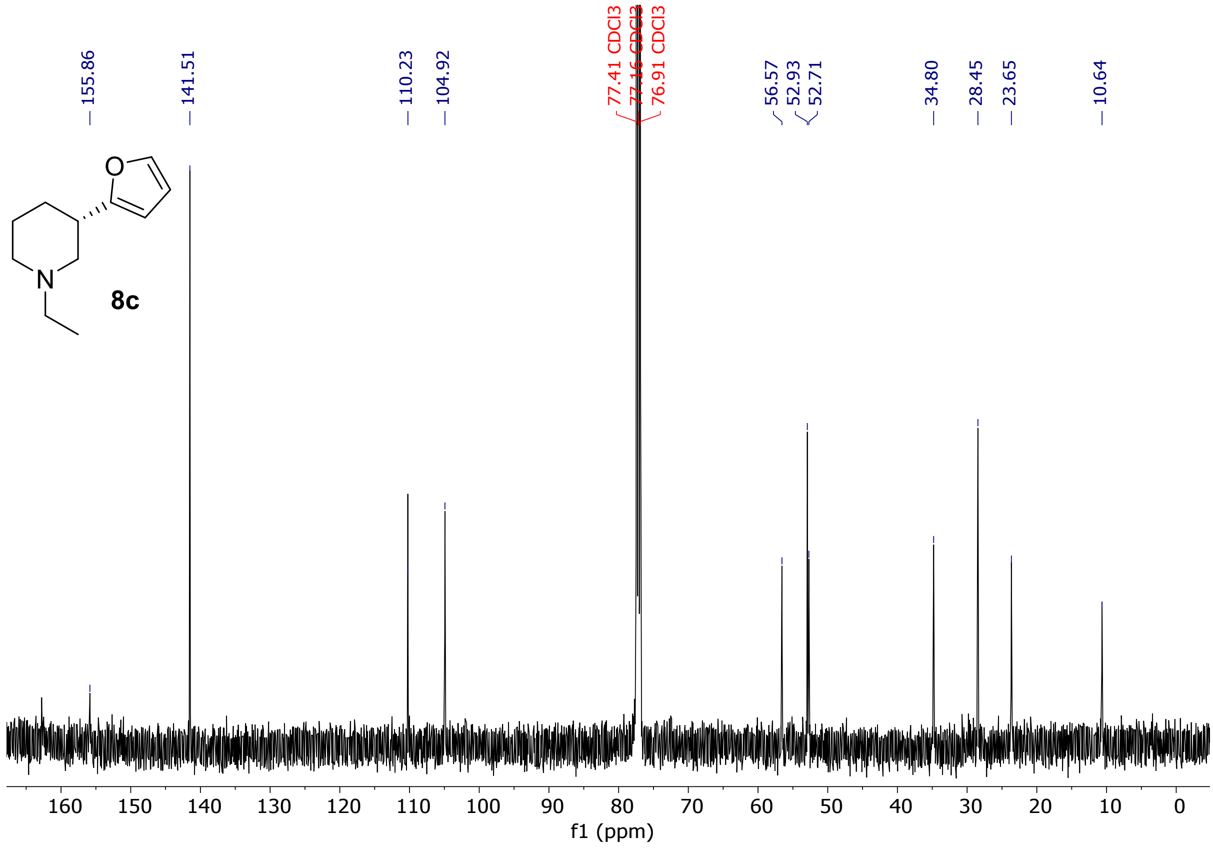

^{1}H & ^{13}C NMR spectra for (*S*)-1-ethyl-3-(thiophen-2-yl)piperidine (9c)

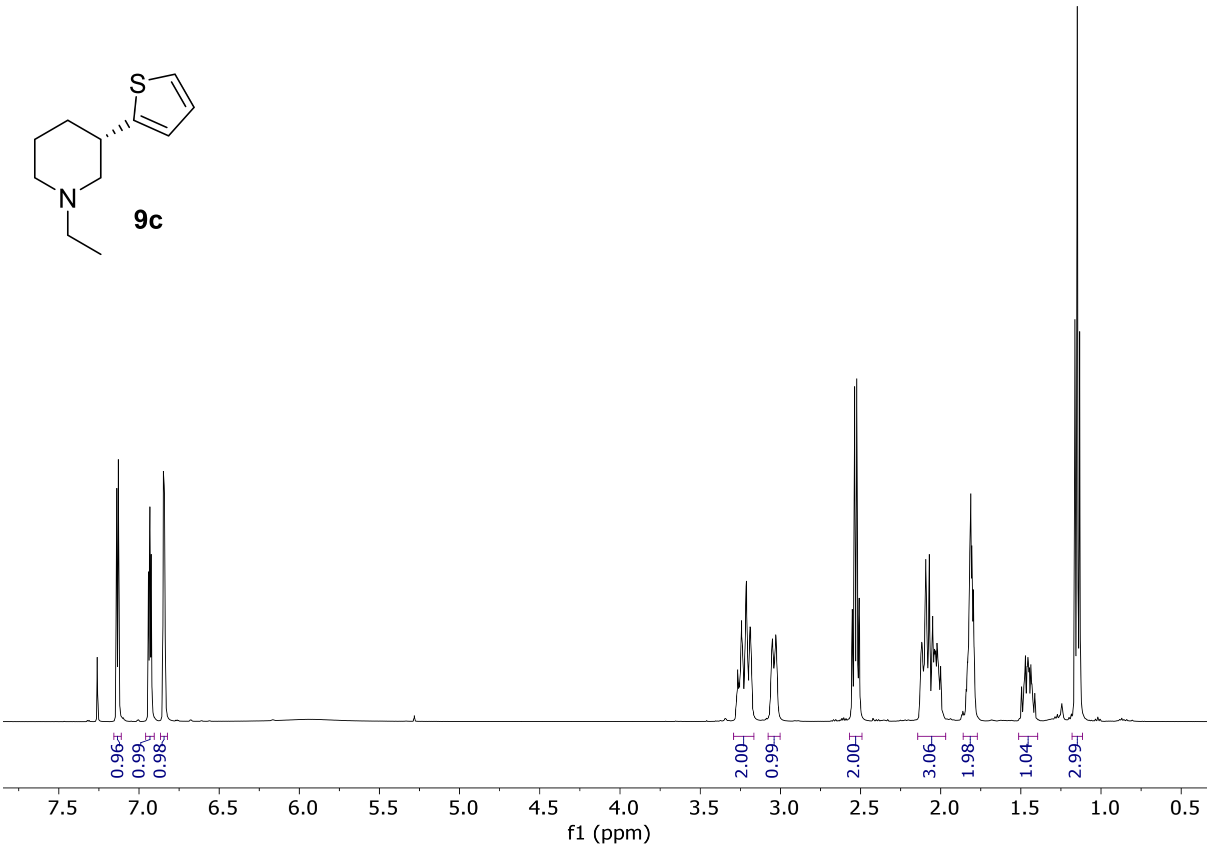

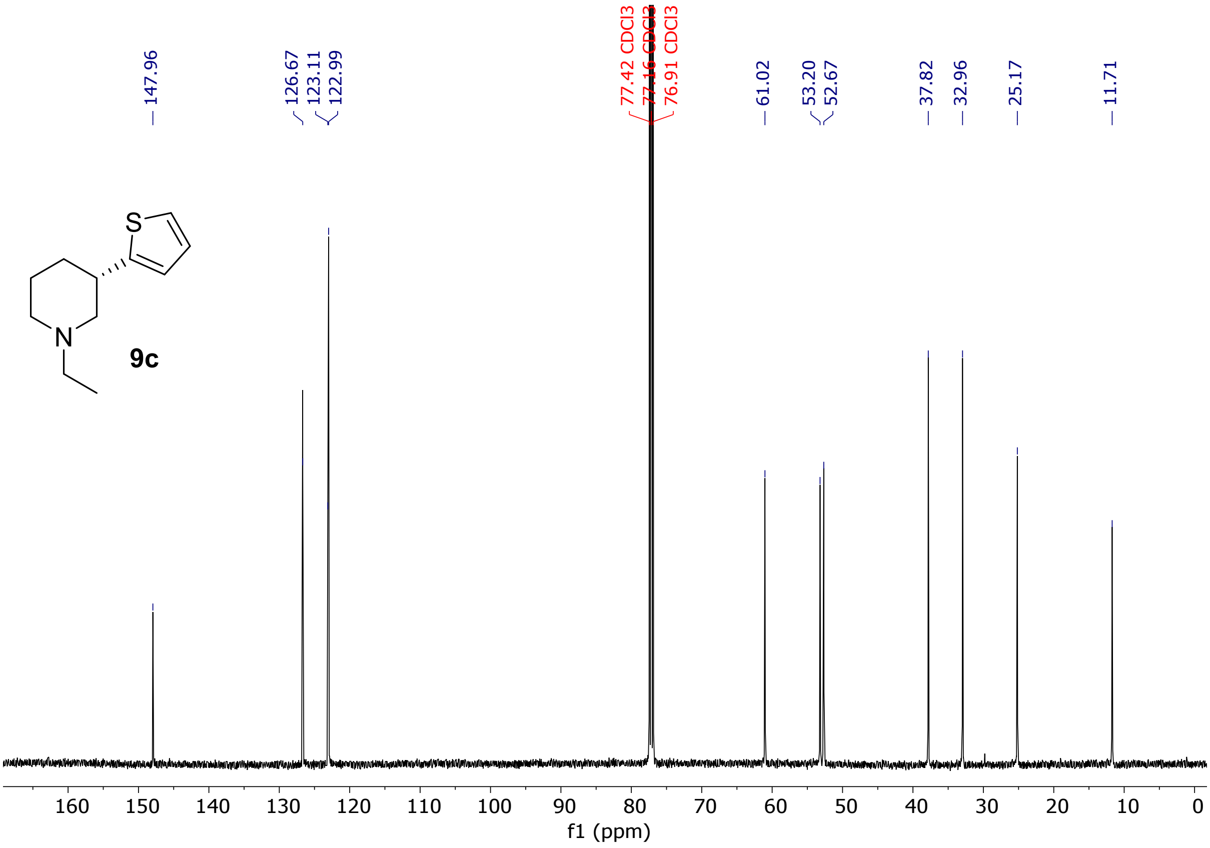

^{1}H & ^{13}C NMR spectra for 1-ethyl-5-(naphthalen-2-yl)-1,2,3,4-THP (10e)

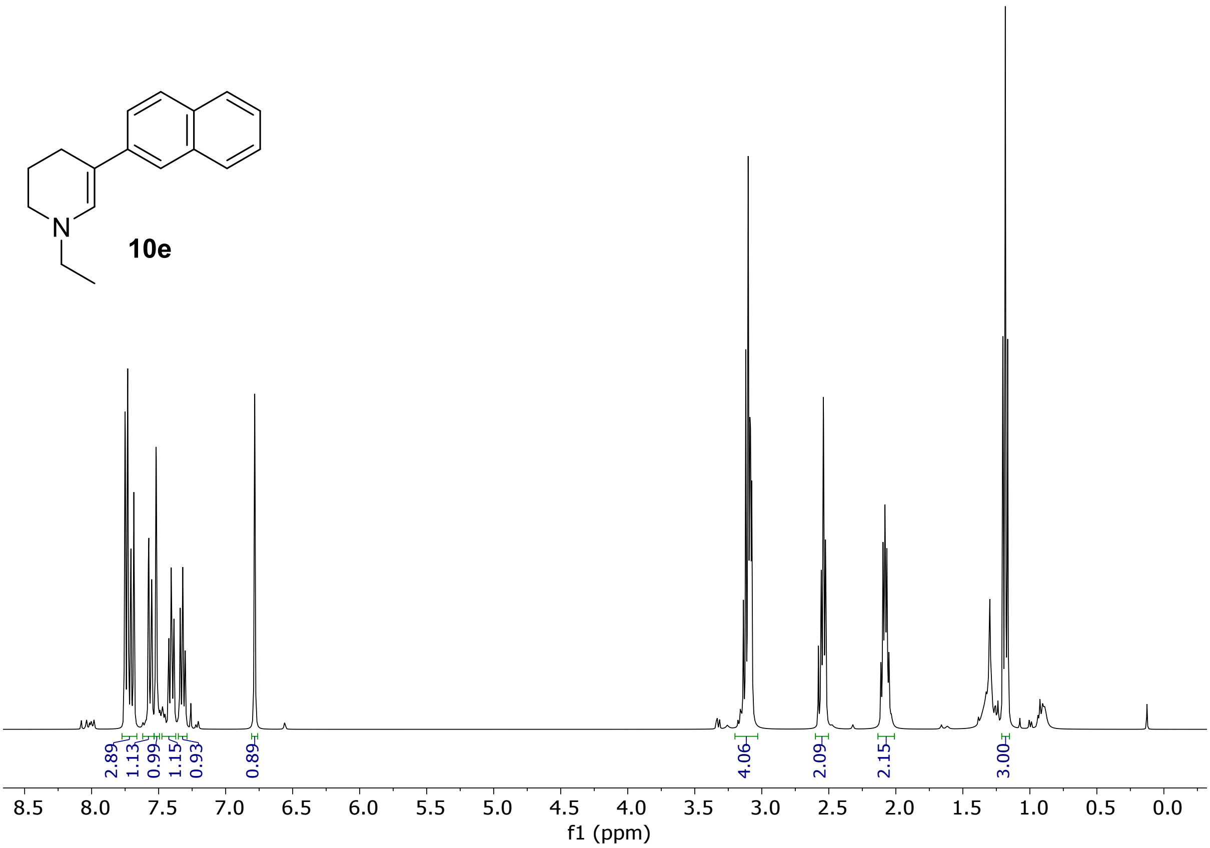

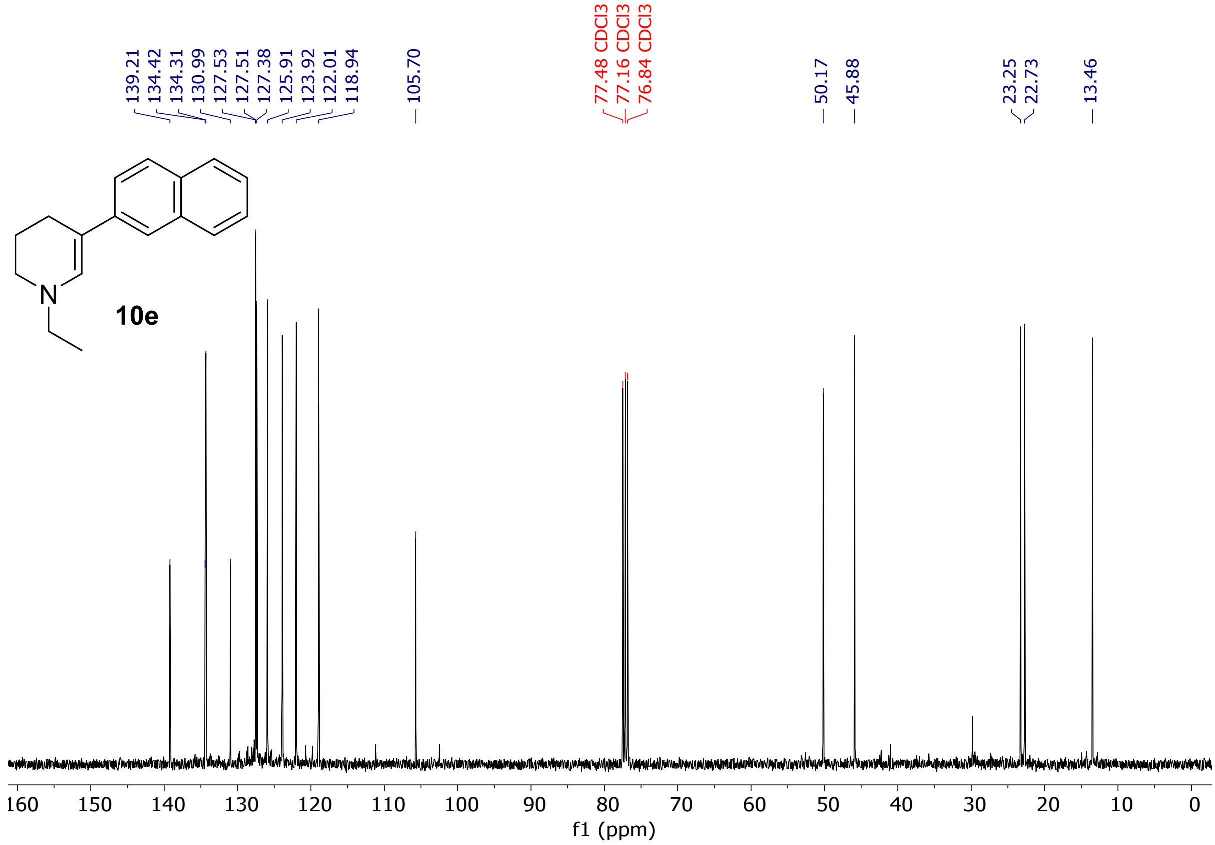

^{1}H & ^{13}C NMR spectra for (*R*)-1-ethyl-3-(naphthalen-2-yl)piperidine (10c)

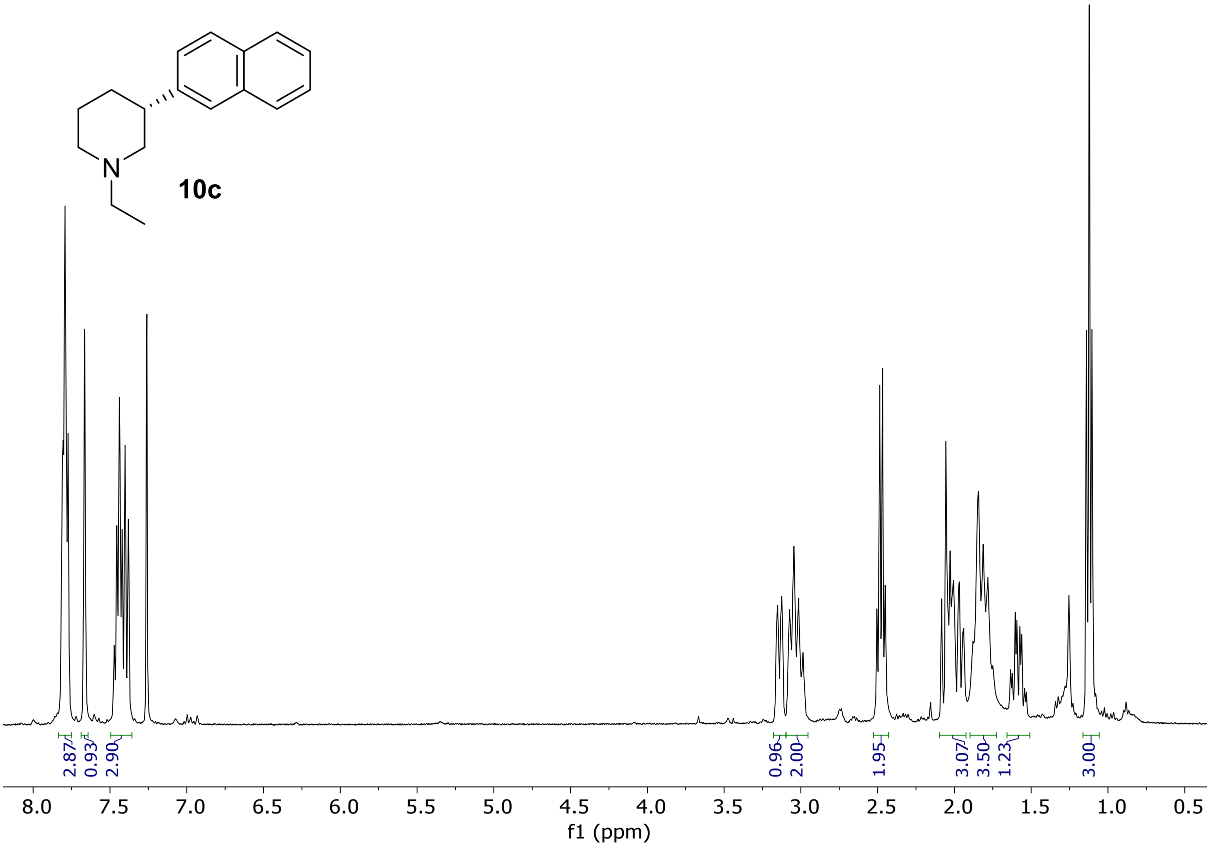

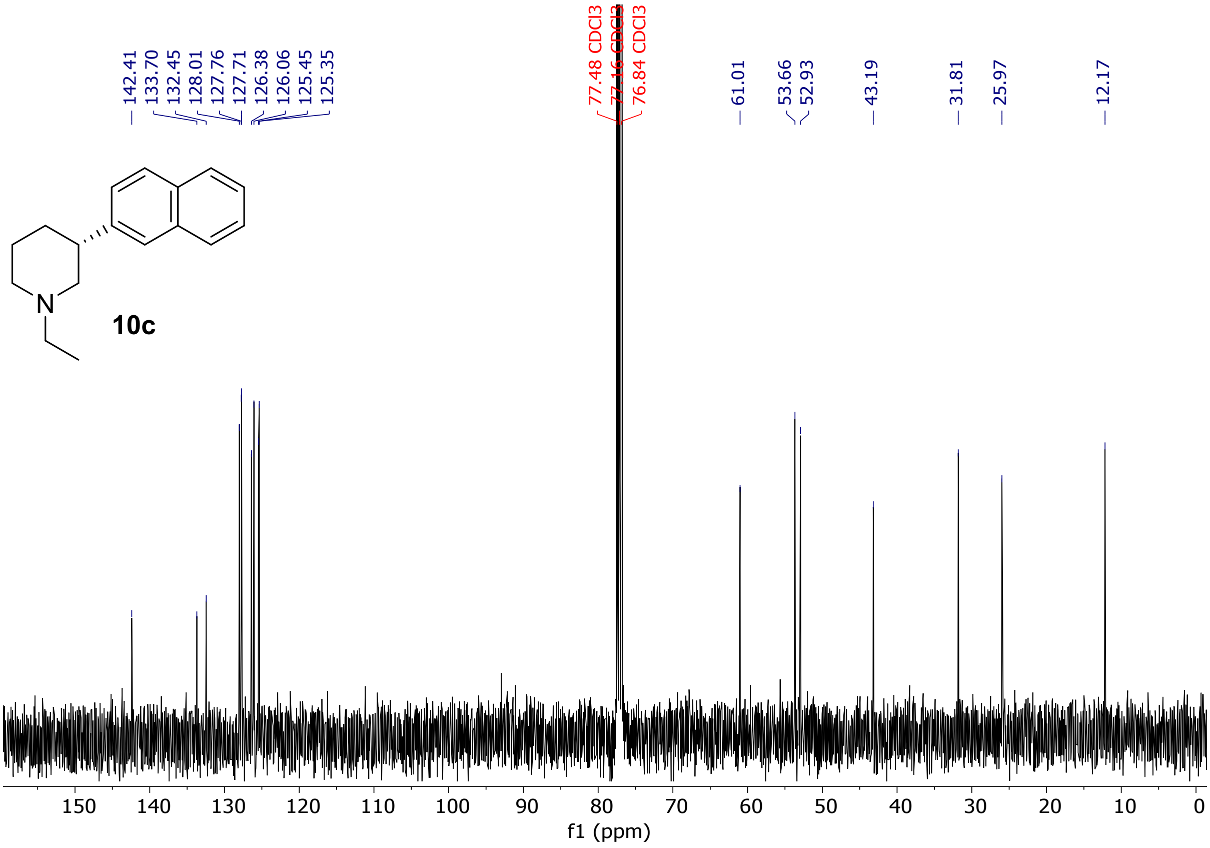

^{1}H & ^{13}C NMR spectra for (3*A*)-1-ethyl-3-(naphthalen-2-yl)piperidine-2-*d* (10c)

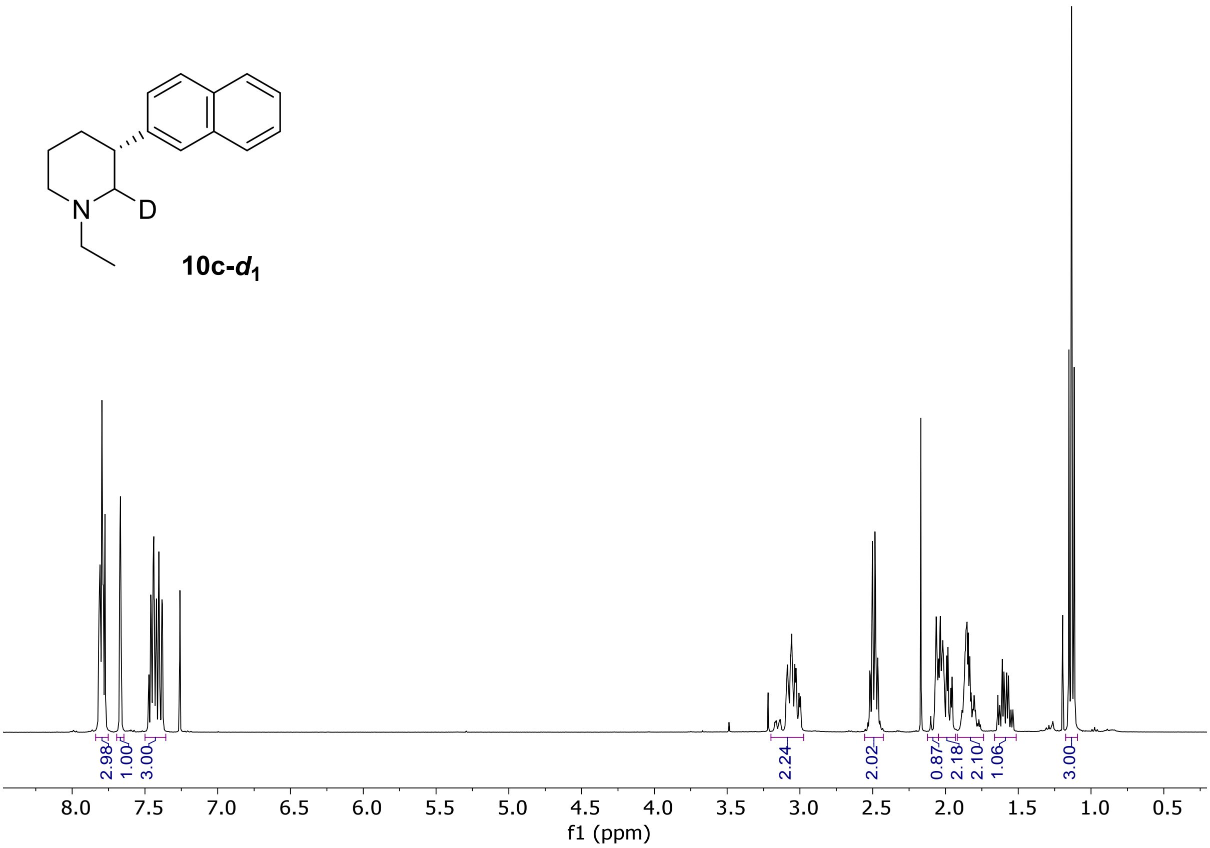

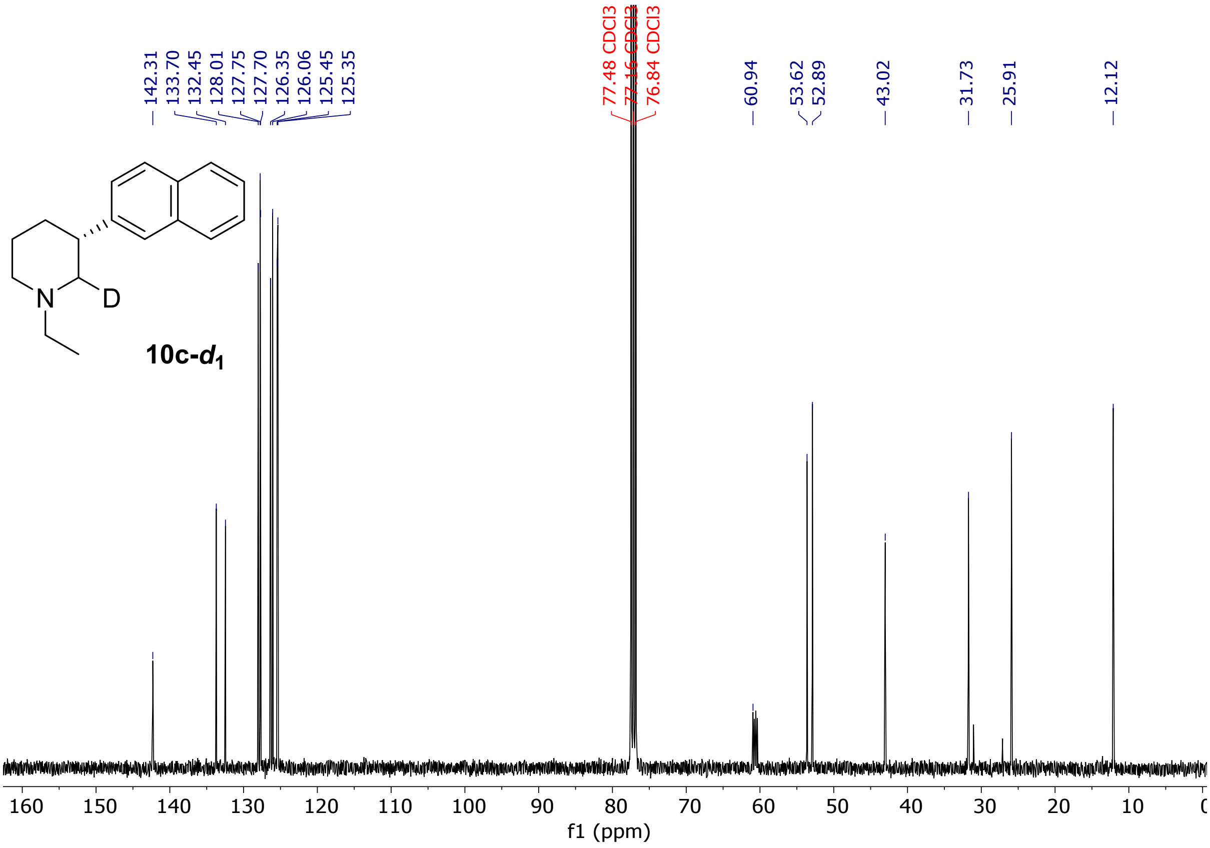

^{1}H & ^{13}C NMR spectra for (3*A*)-1-ethyl-3-(naphthalen-2-yl)piperidine-2,4-d$_2$ (10c)

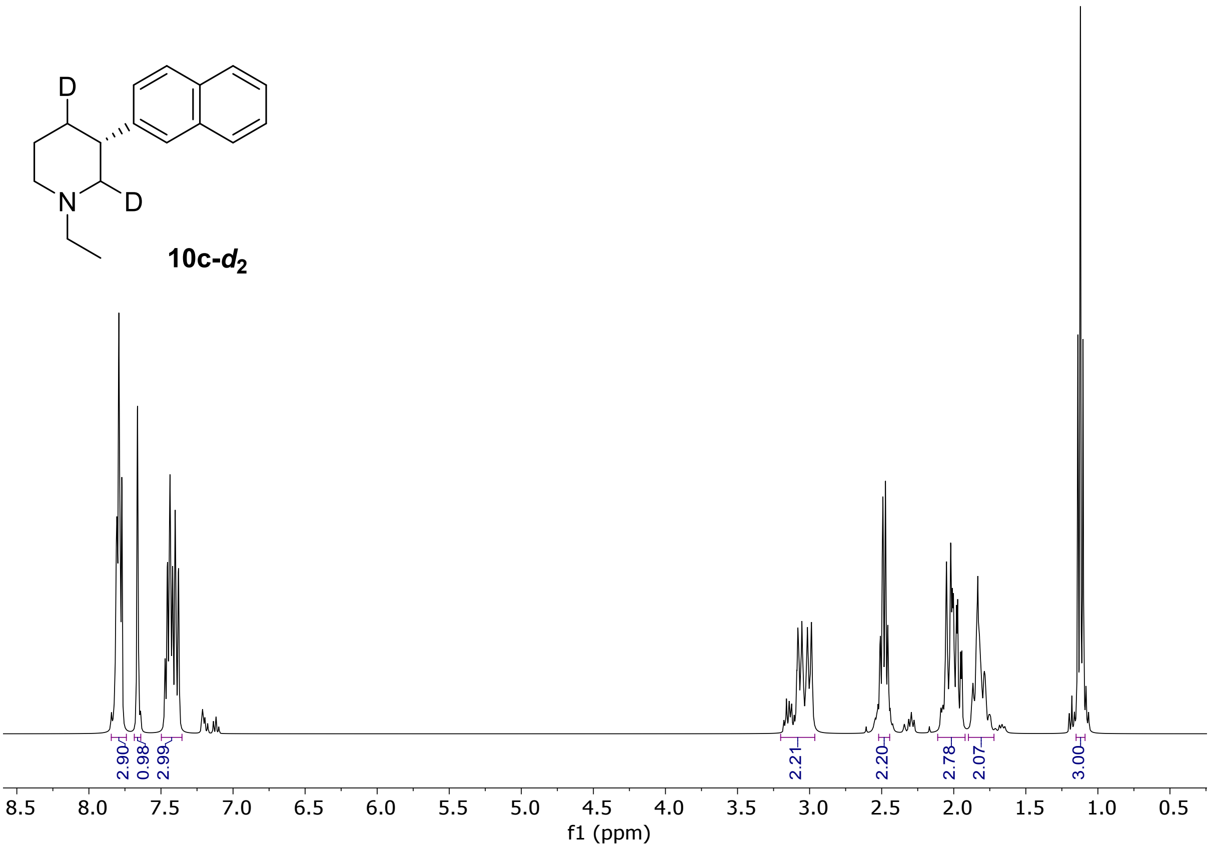

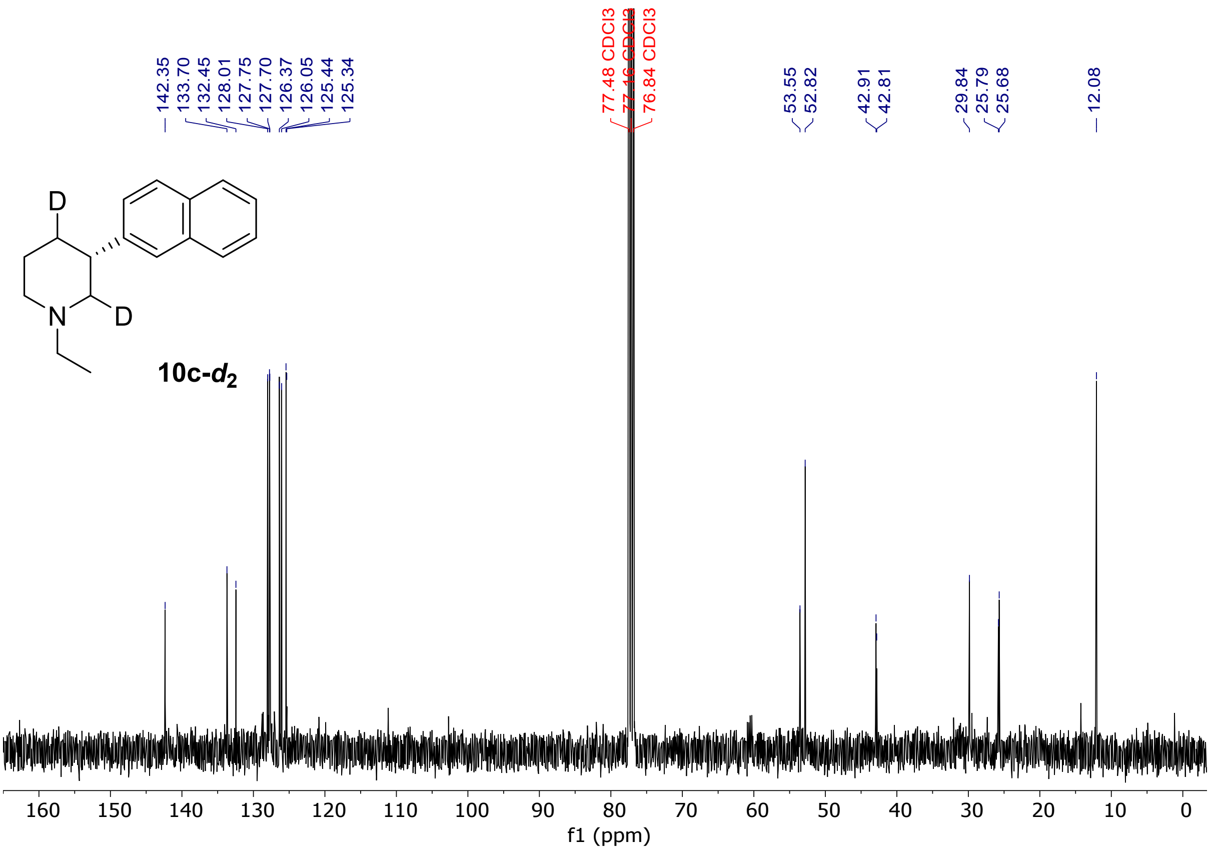

^{1}H & ^{13}C NMR spectra for (*R*)-1-methyl-3-phenylpiperidine (11c)

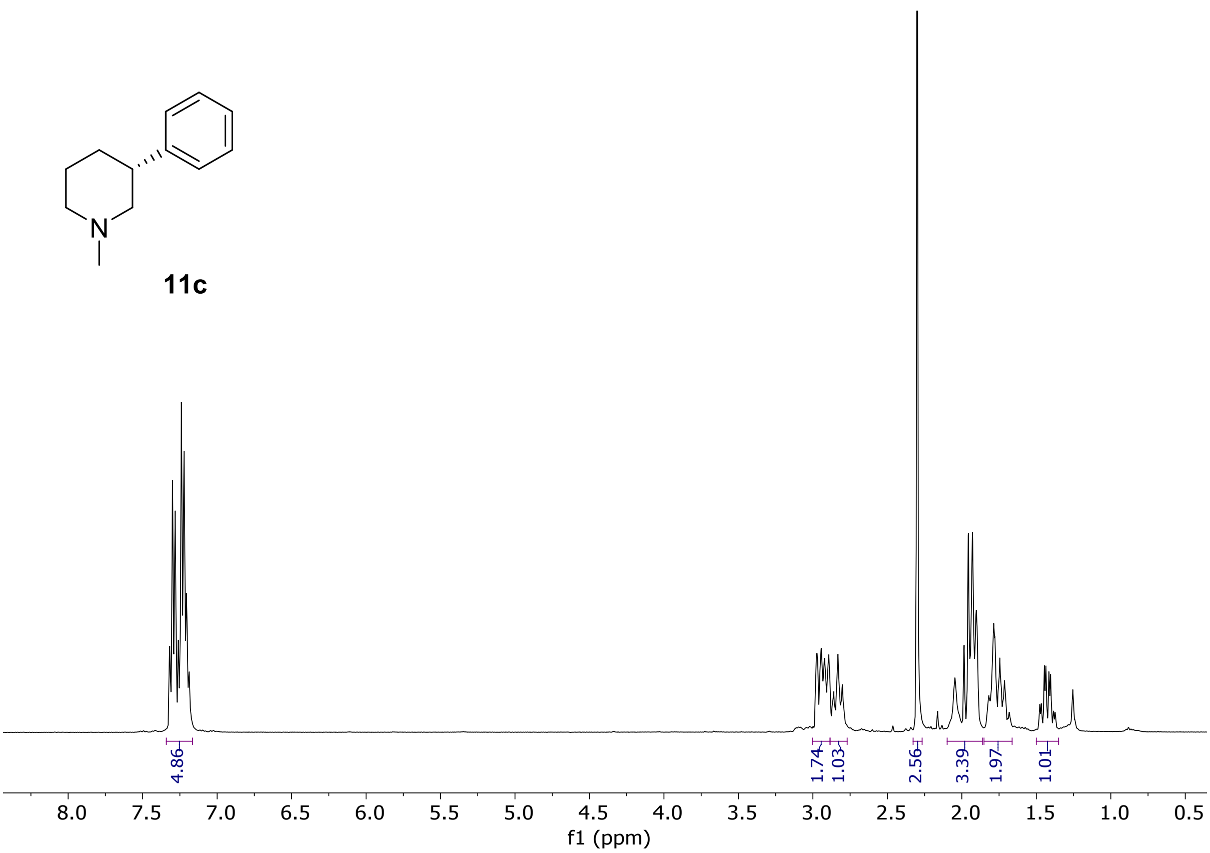

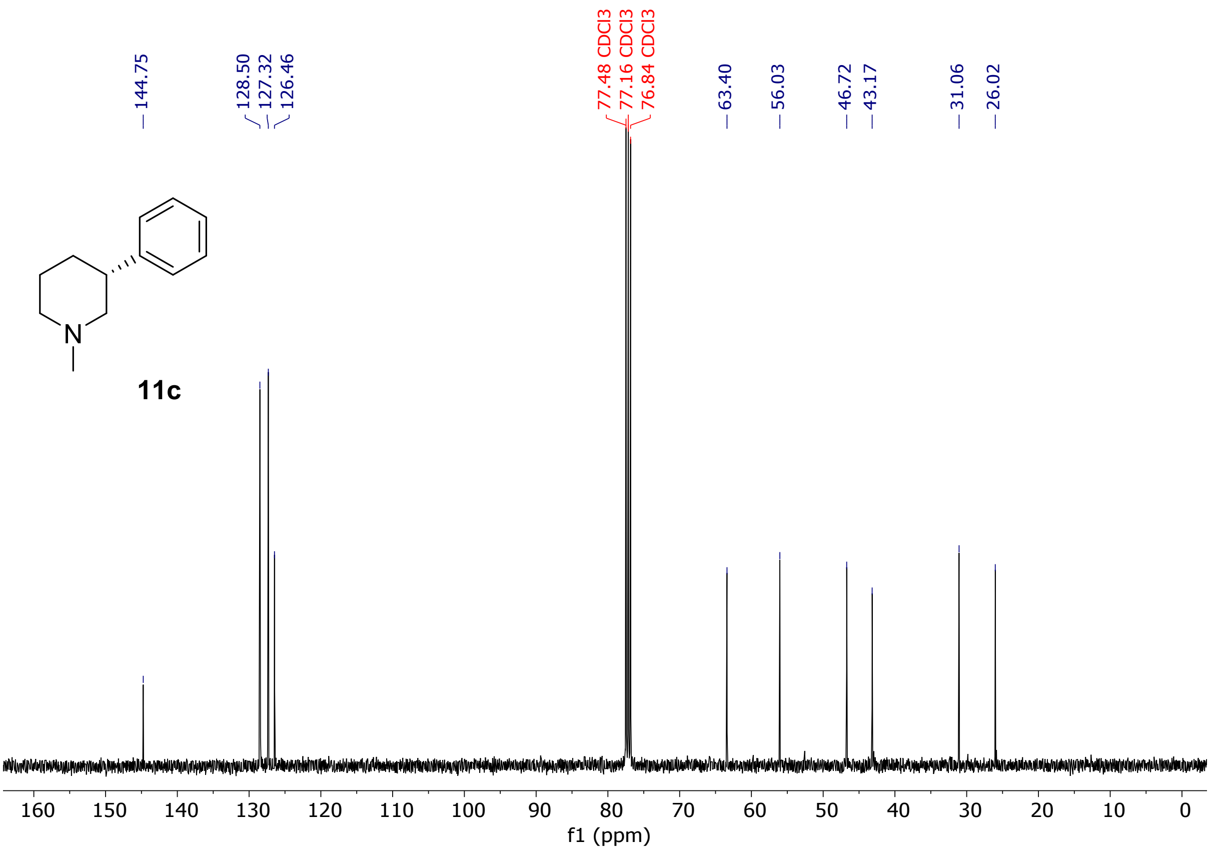

^{1}H & ^{13}C NMR spectra for (*A*)-3-phenyl-1-(prop-2-yn-1-yl)piperidine (12c)

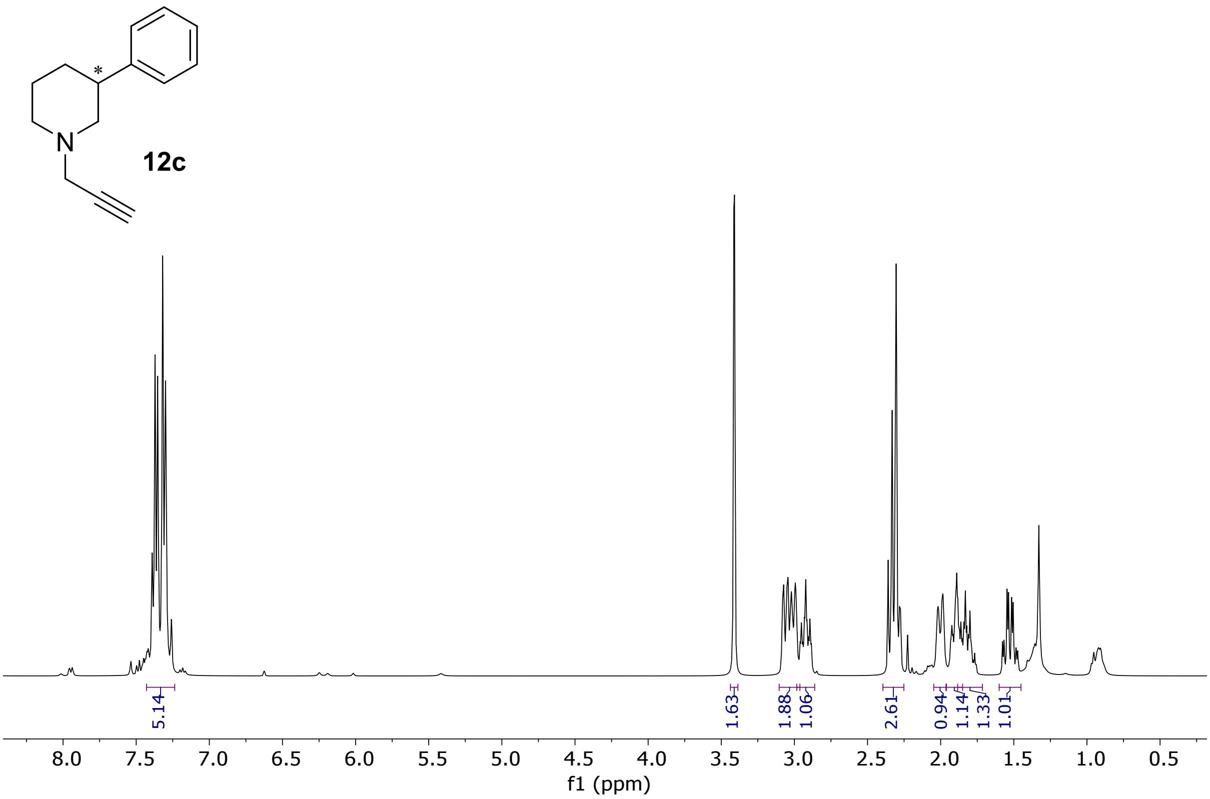

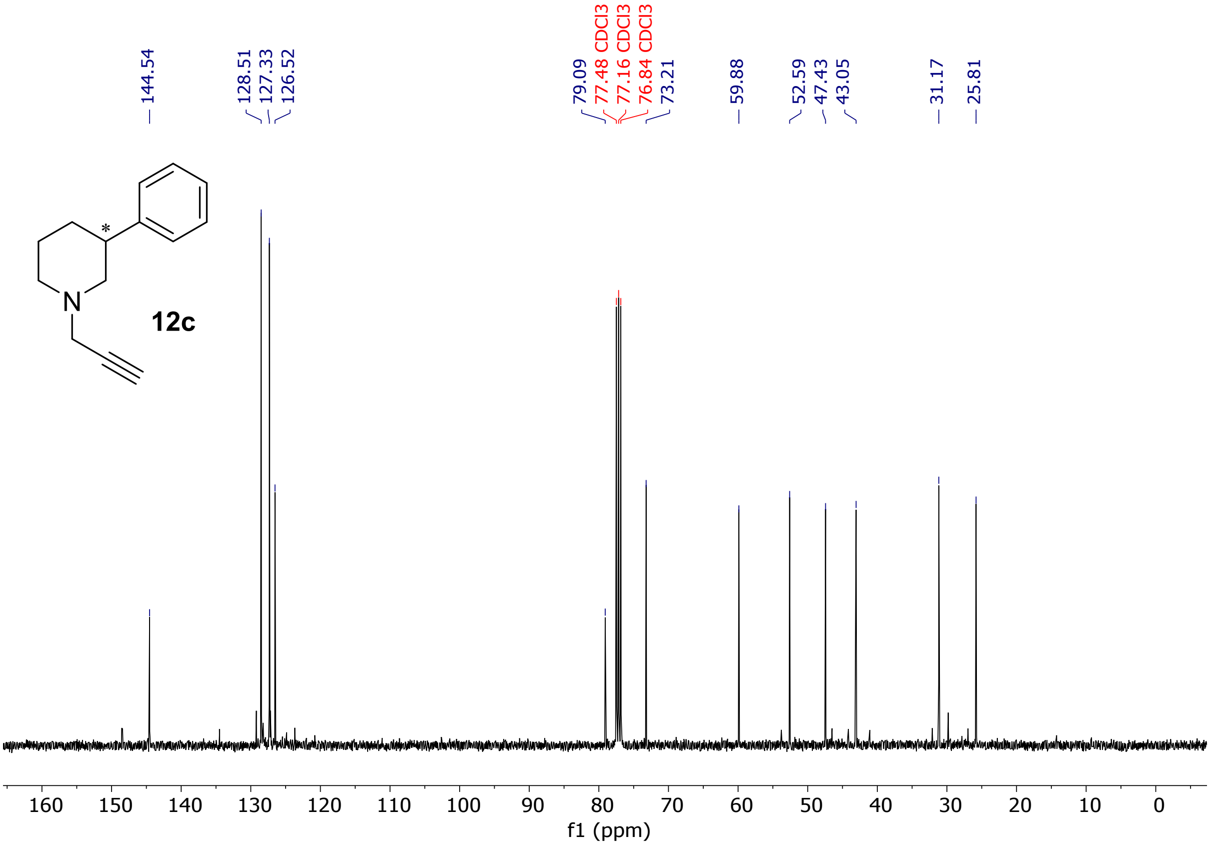

^{1}H & ^{13}C NMR spectra for (*A*)-1-ethyl-3-methylpiperidine (13c.HCl salt)

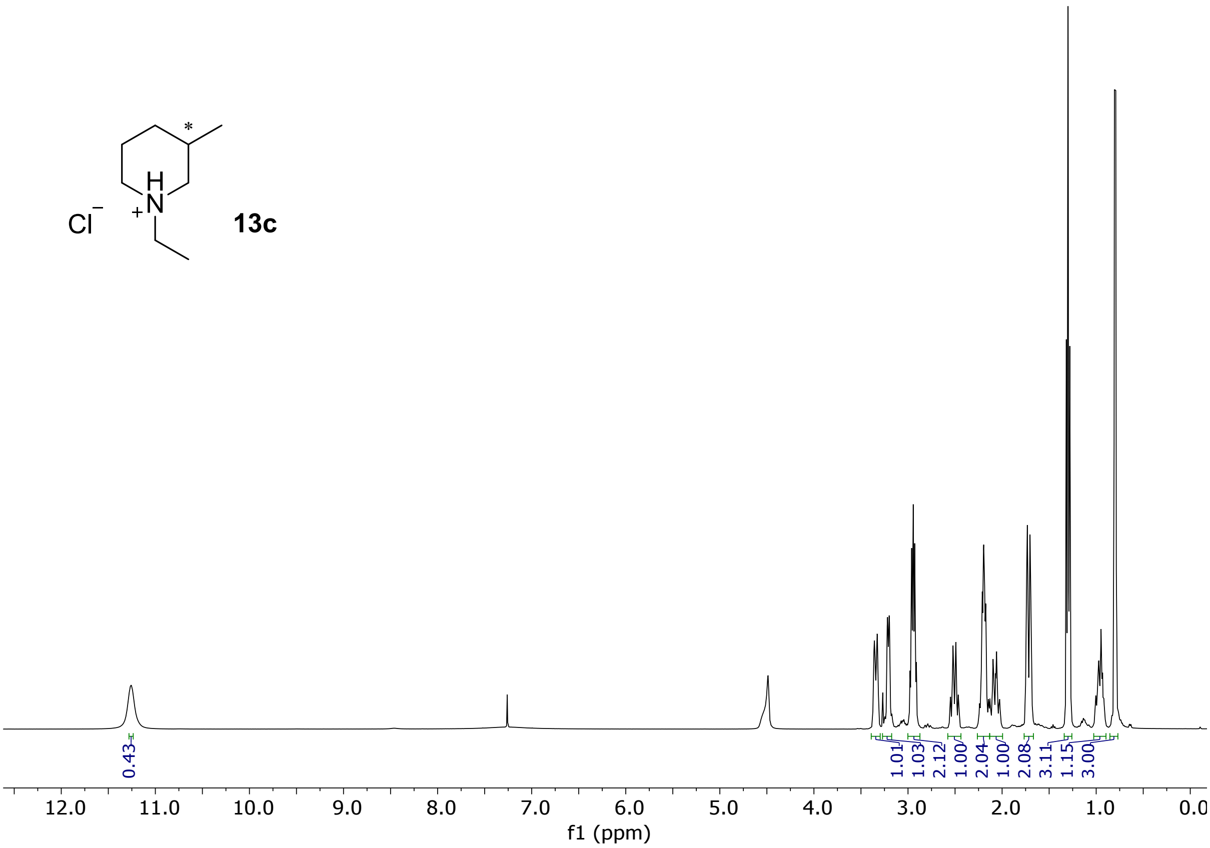

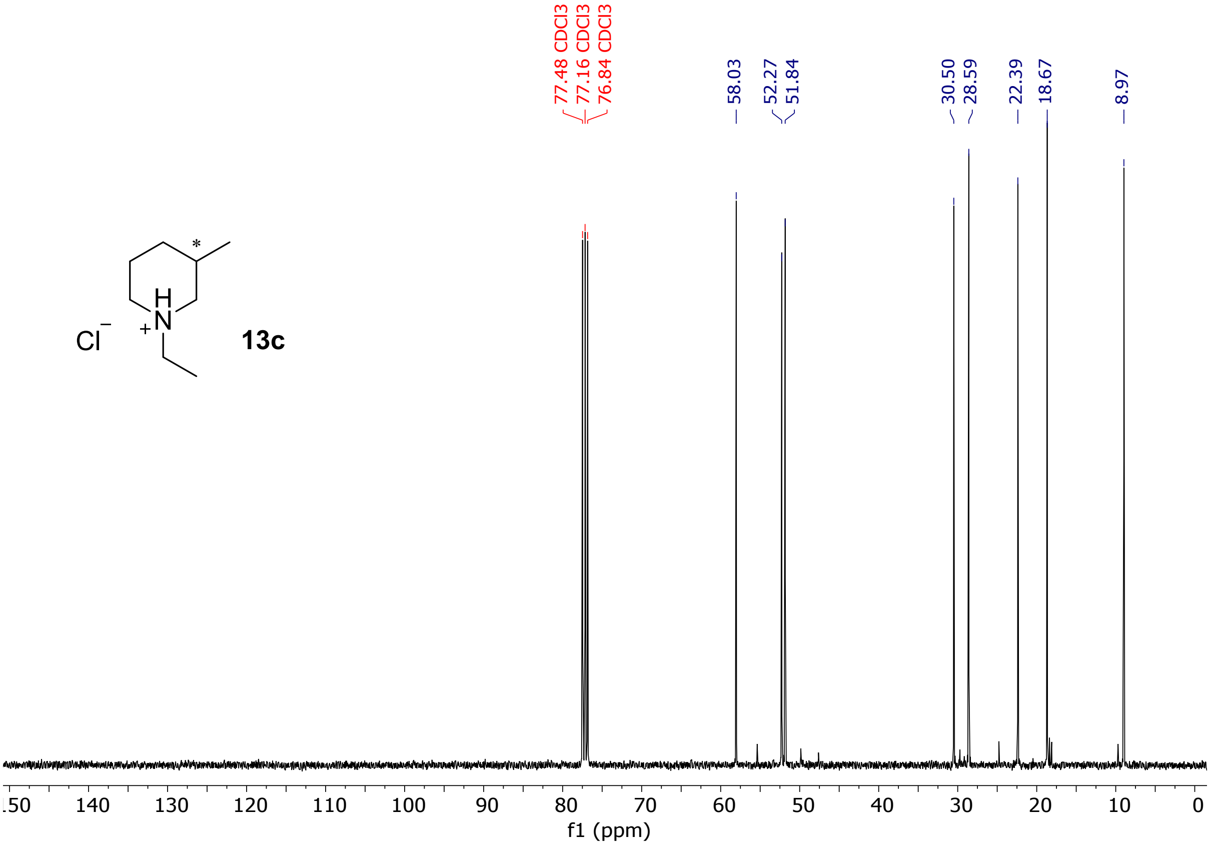

^{1}H & ^{13}C NMR spectra for 1-ethyl-4-methylpiperidine (15c.HCl salt)

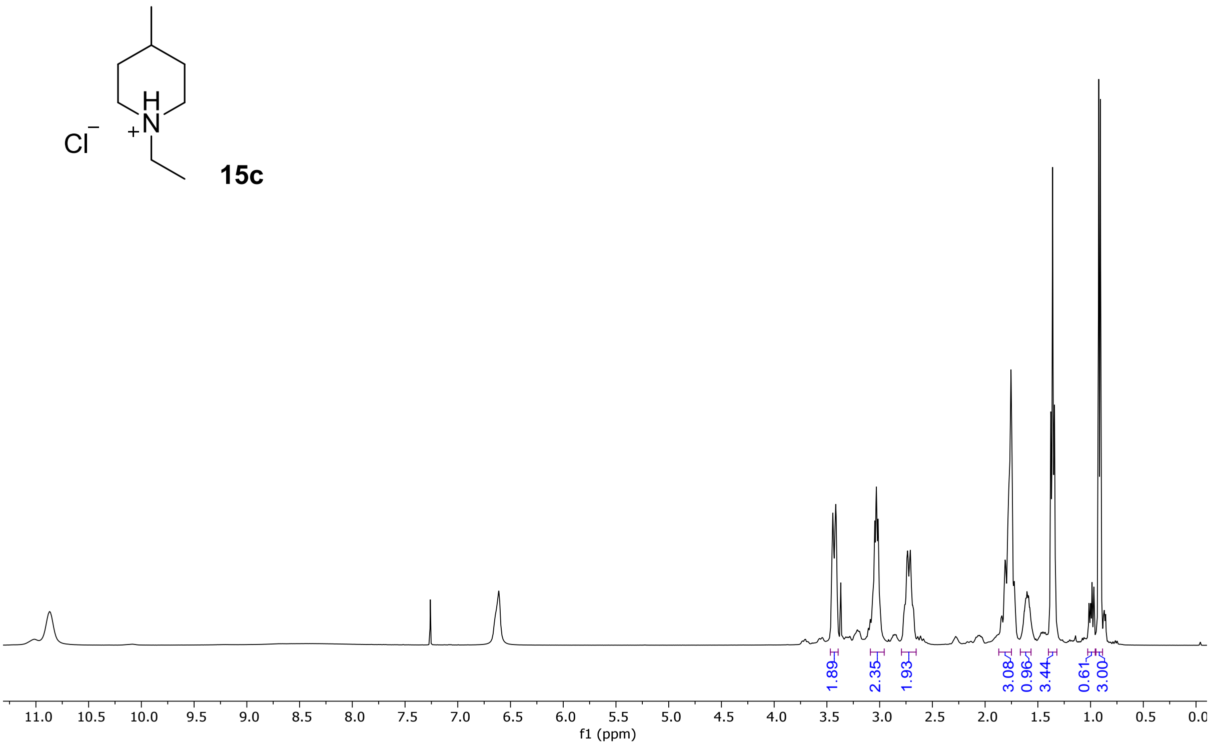

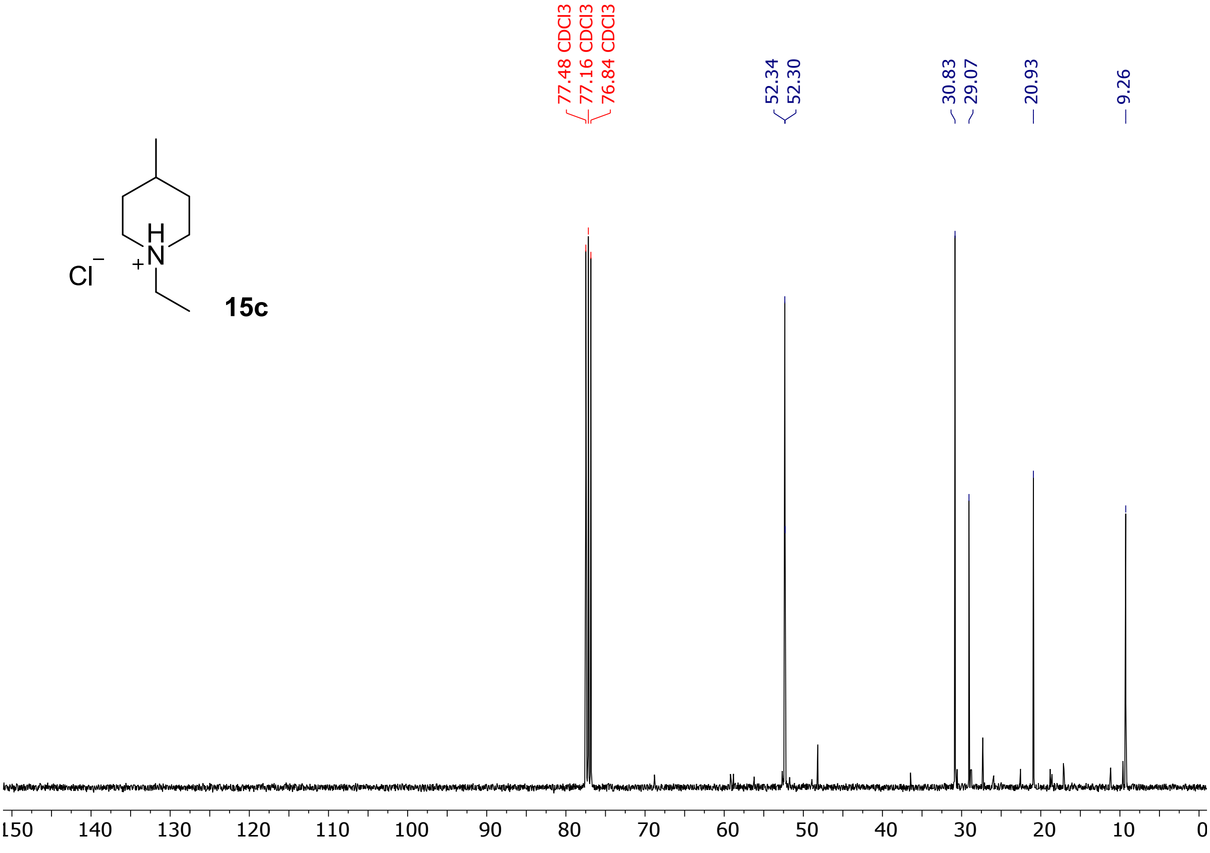

^{1}H & ^{13}C NMR spectra for 1-ethyl-4-phenylpiperidine (16c)

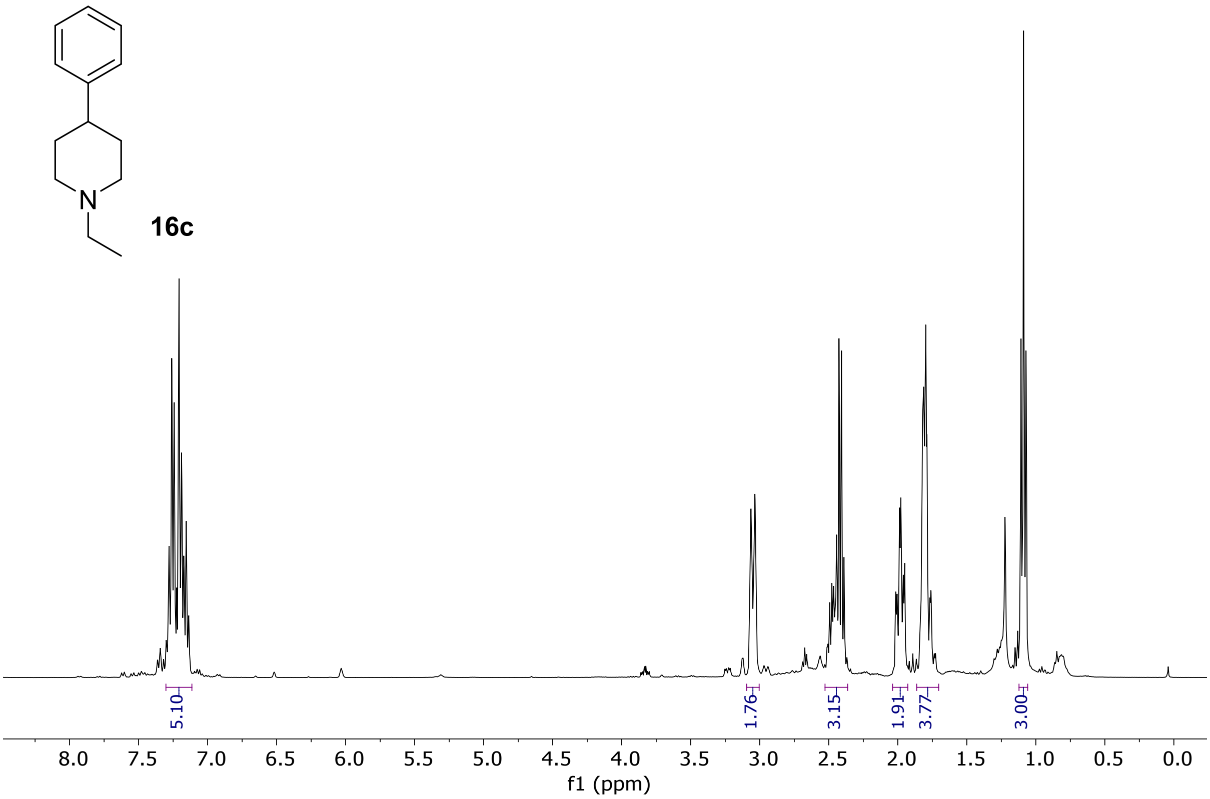

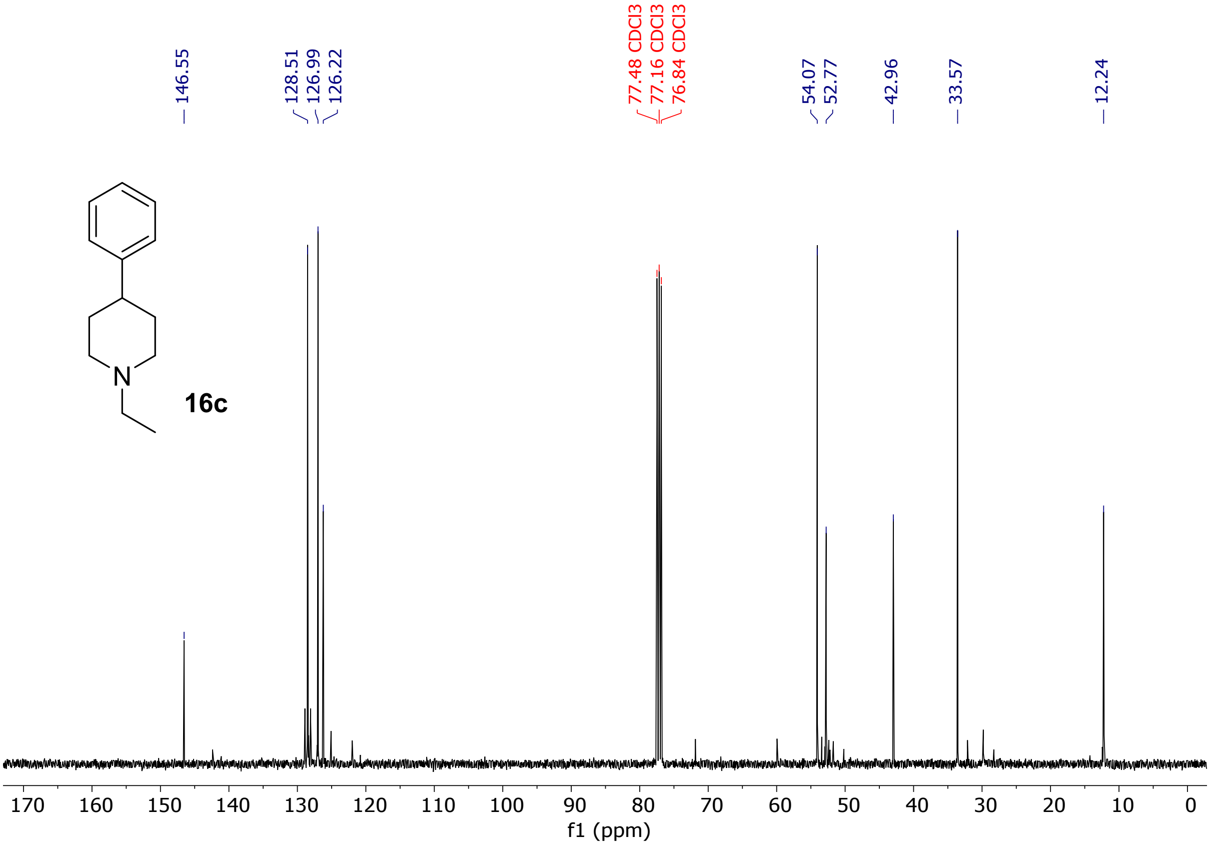

^{1}H & ^{13}C NMR spectra for 1-ethyl-3,4-dimethylpiperidine (17c)

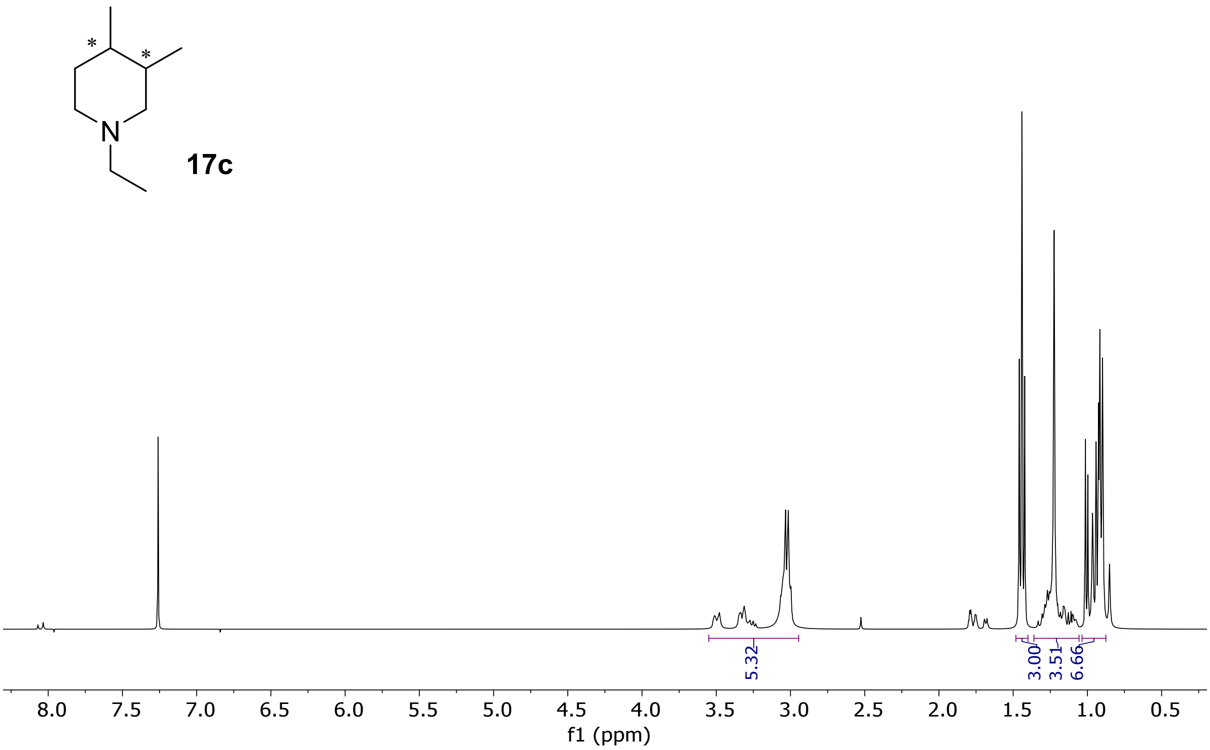

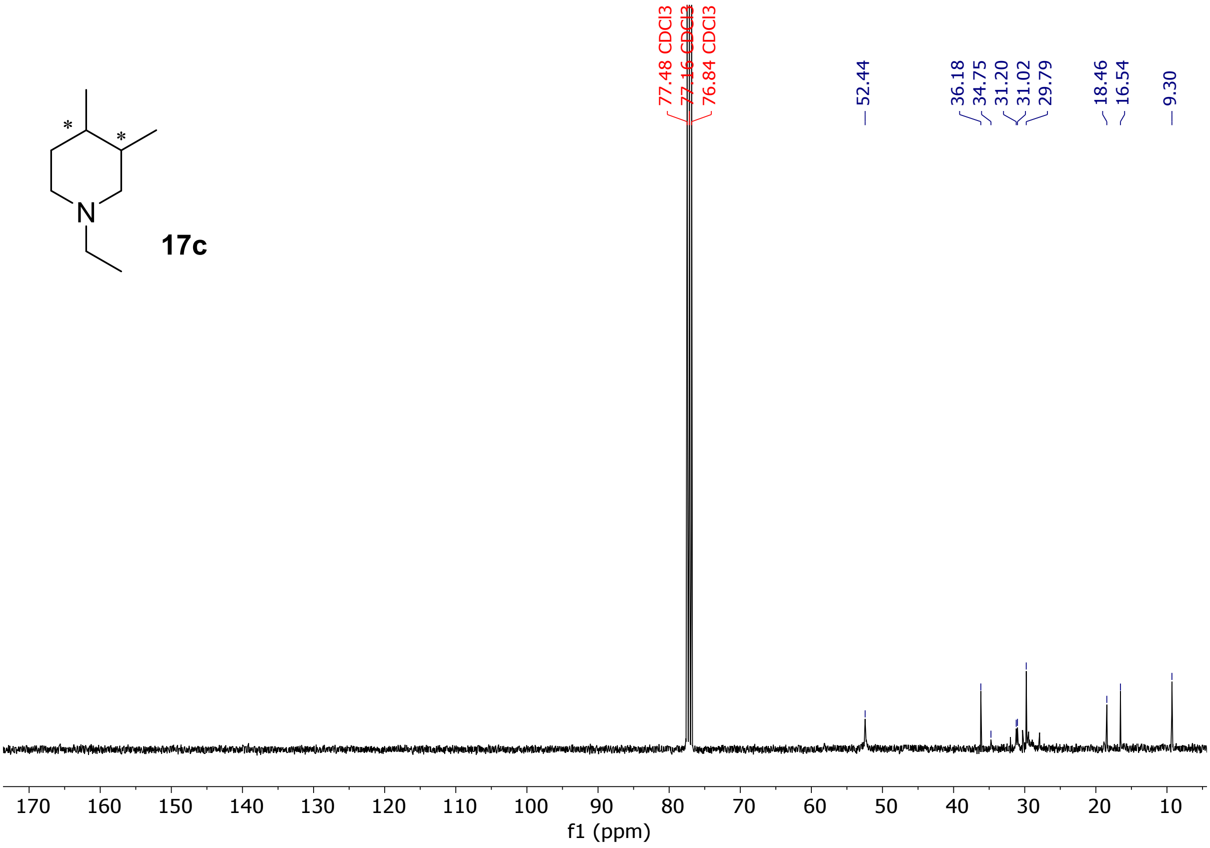

^{1}H & ^{13}C NMR spectra for 2-ethyldecahydroisoquinoline (18c)

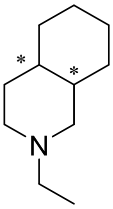

18c

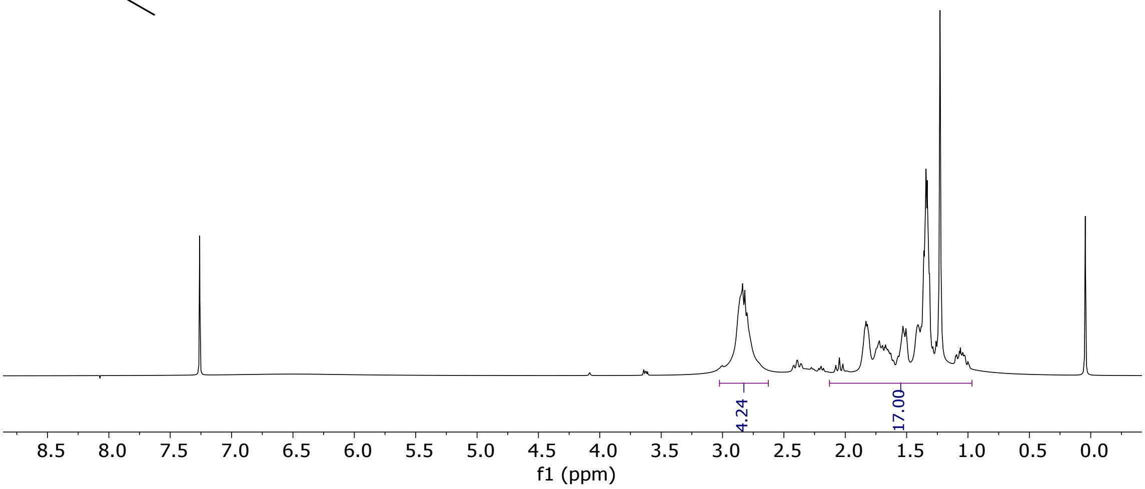

18c

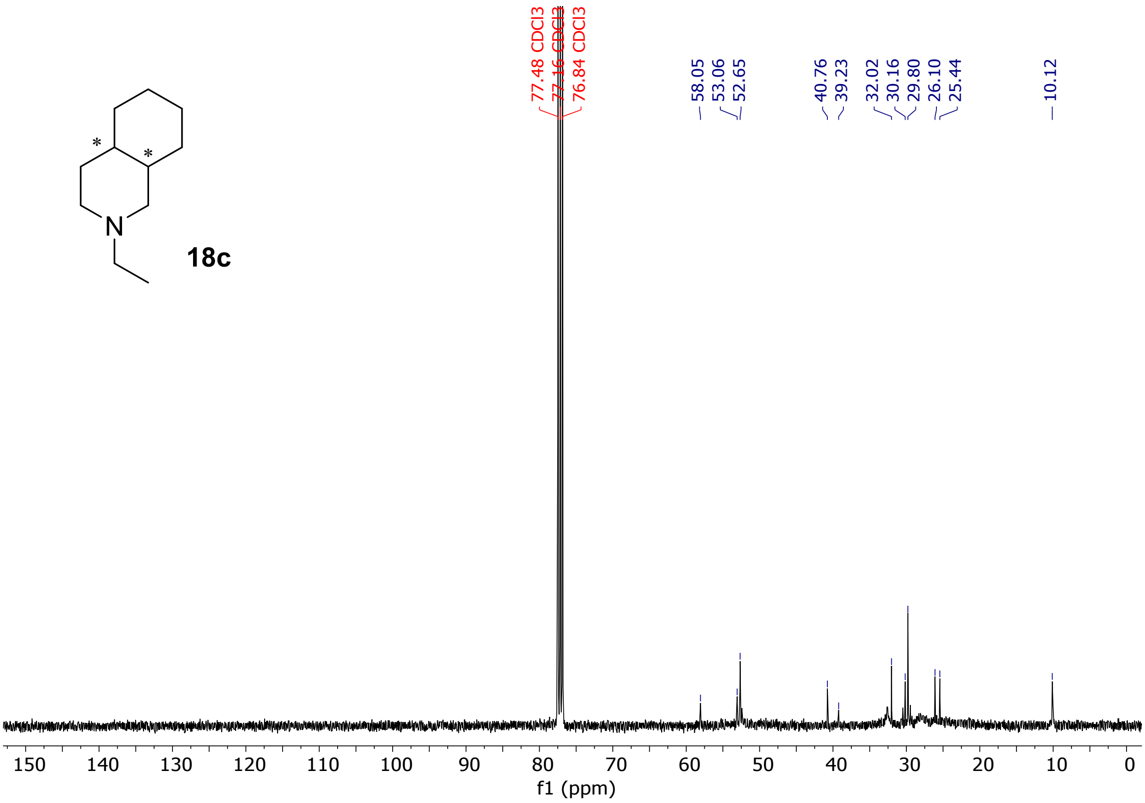

^{1}H & ^{13}C NMR spectra for 1-ethyl-4-methyl-3-phenylpiperidine (19c)

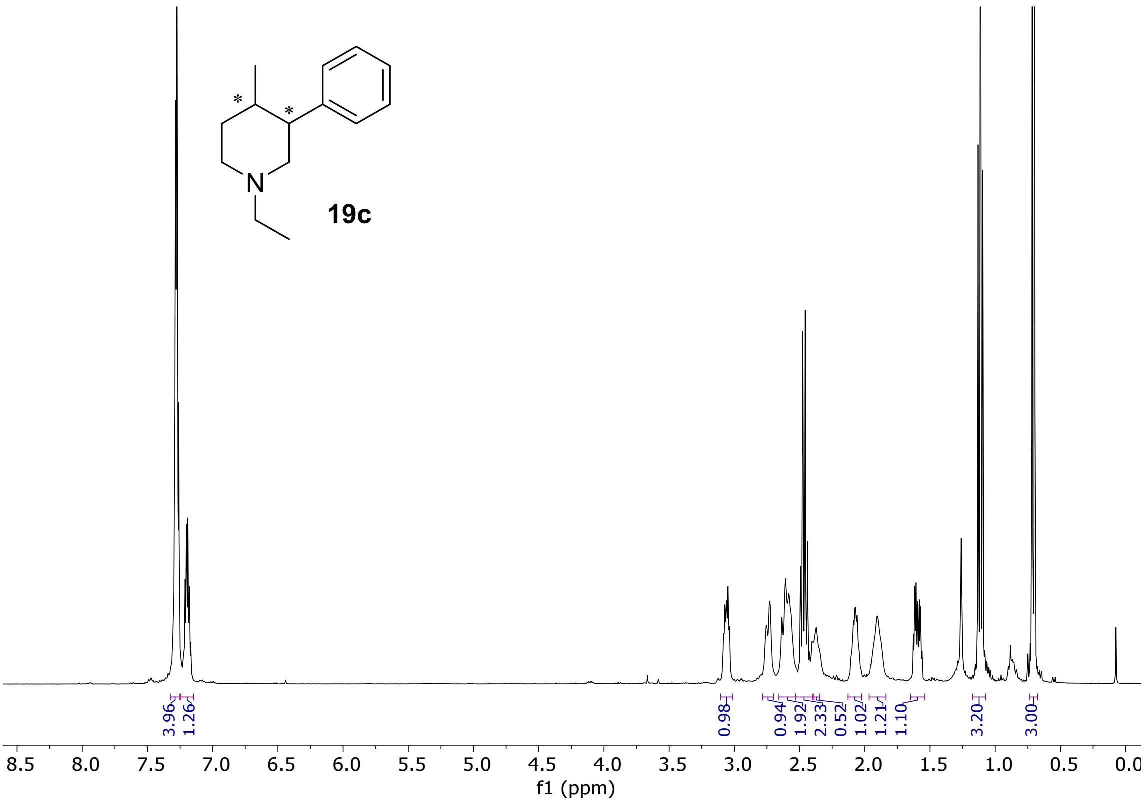

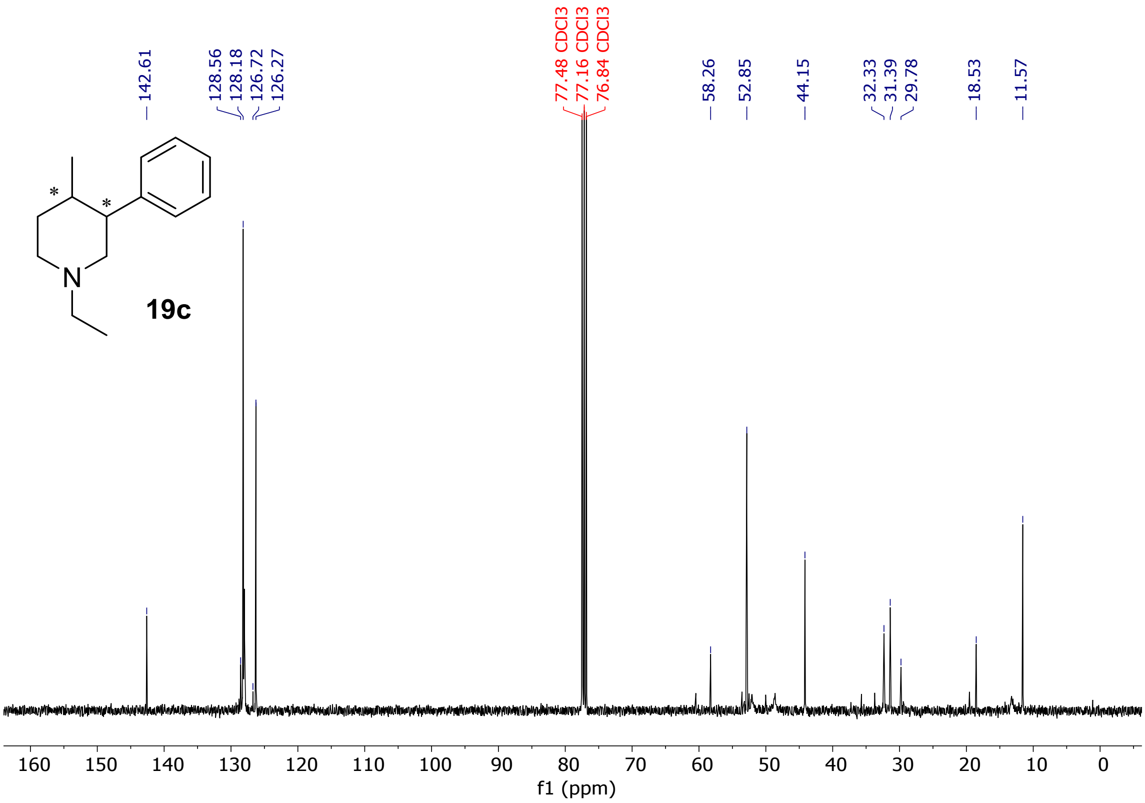

^{1}H & ^{13}C NMR spectra for 1-allyl-4-methyl-3-phenylpiperidine (20c)

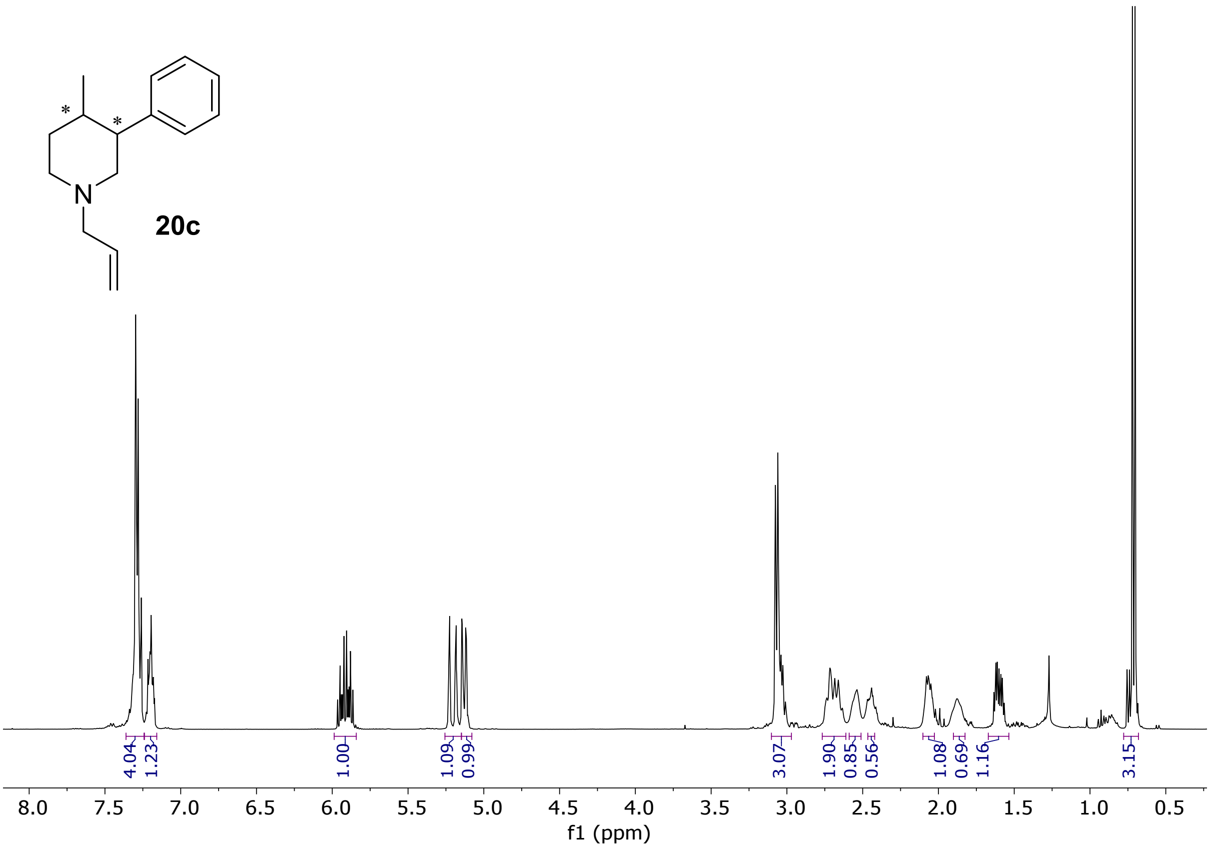

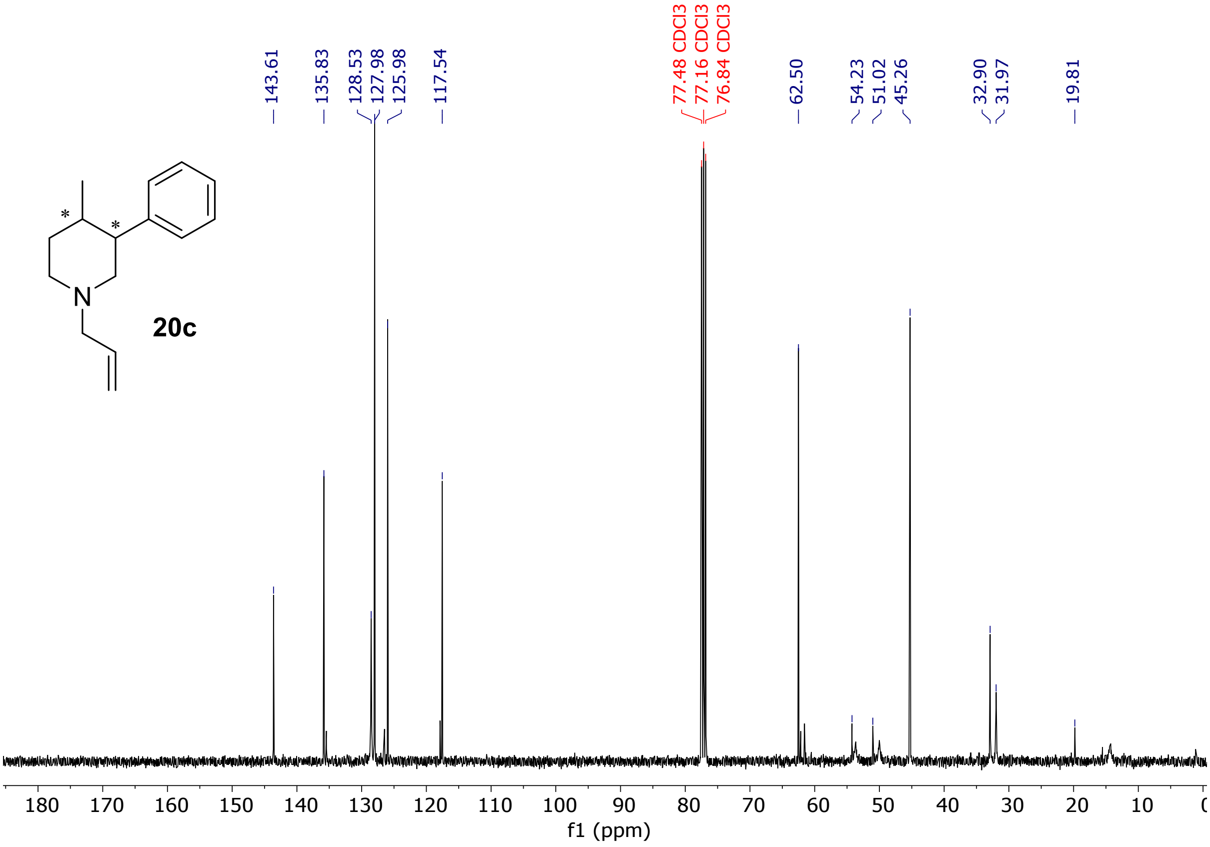

^{1}H & ^{13}C NMR spectra for 3-(3-methoxyphenyl)pyridine

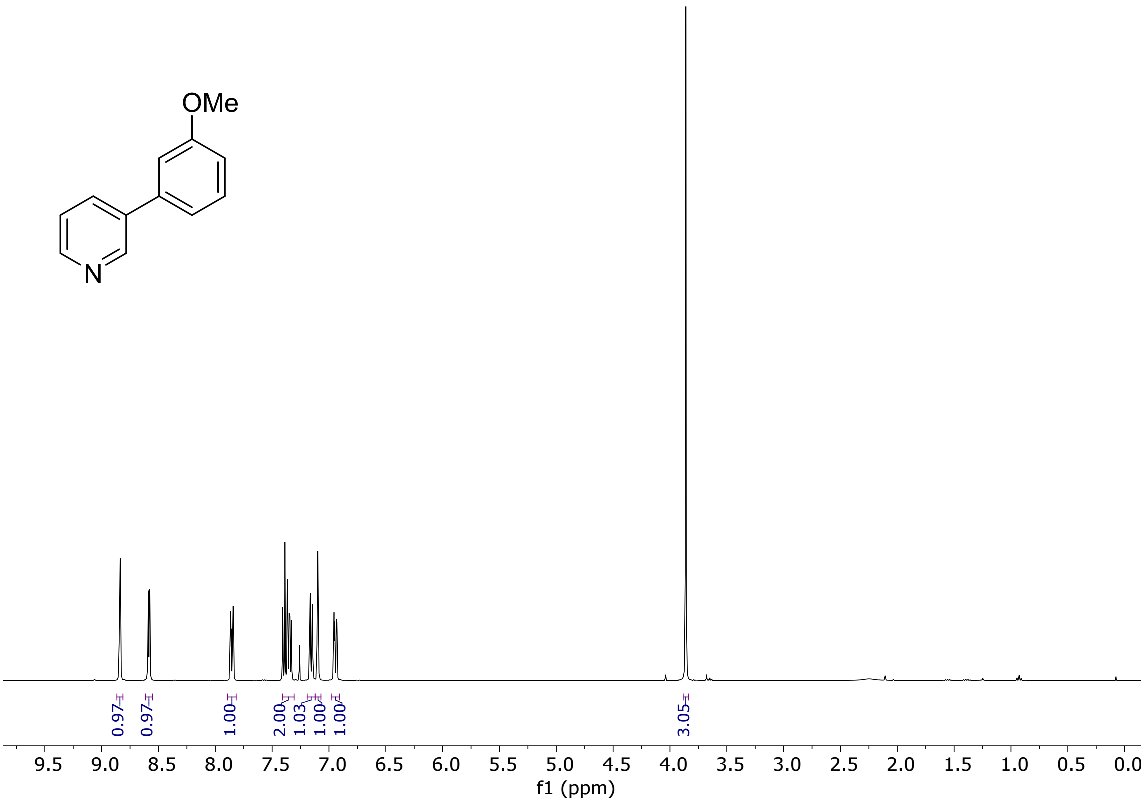

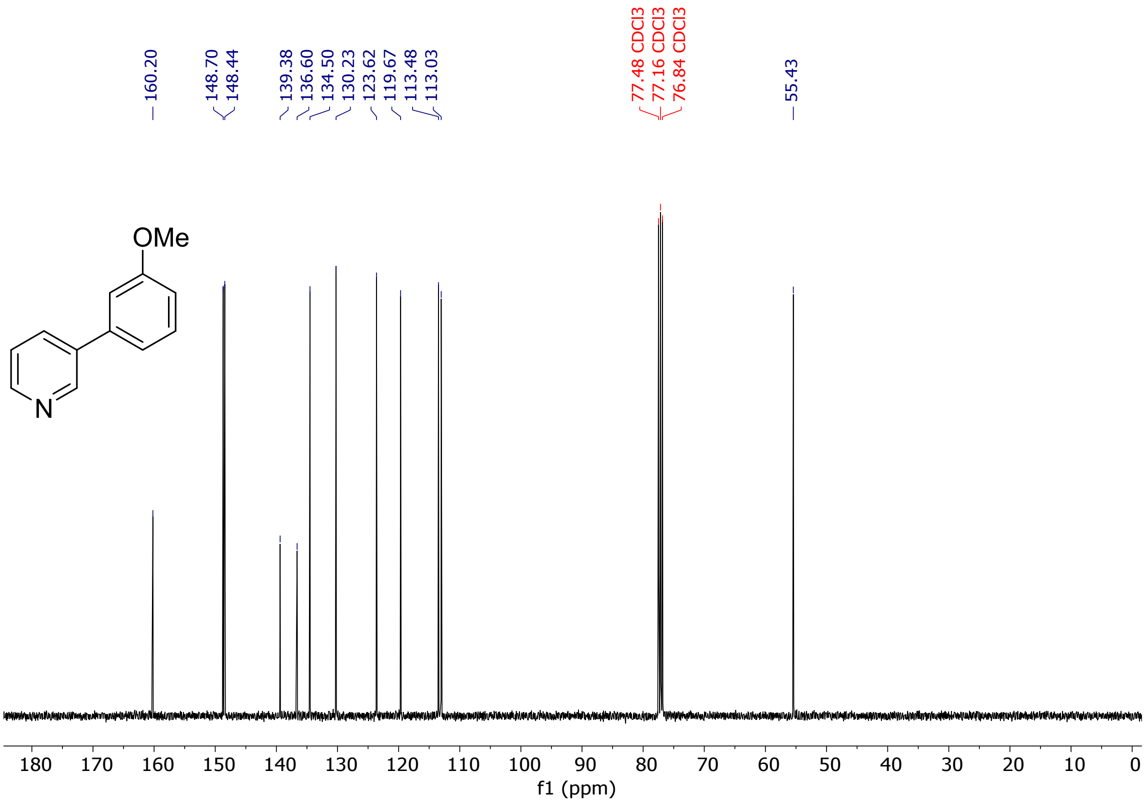

^{1}H & ^{13}C NMR spectra for 3-(3-(methylsulfonyl)phenyl)pyridine

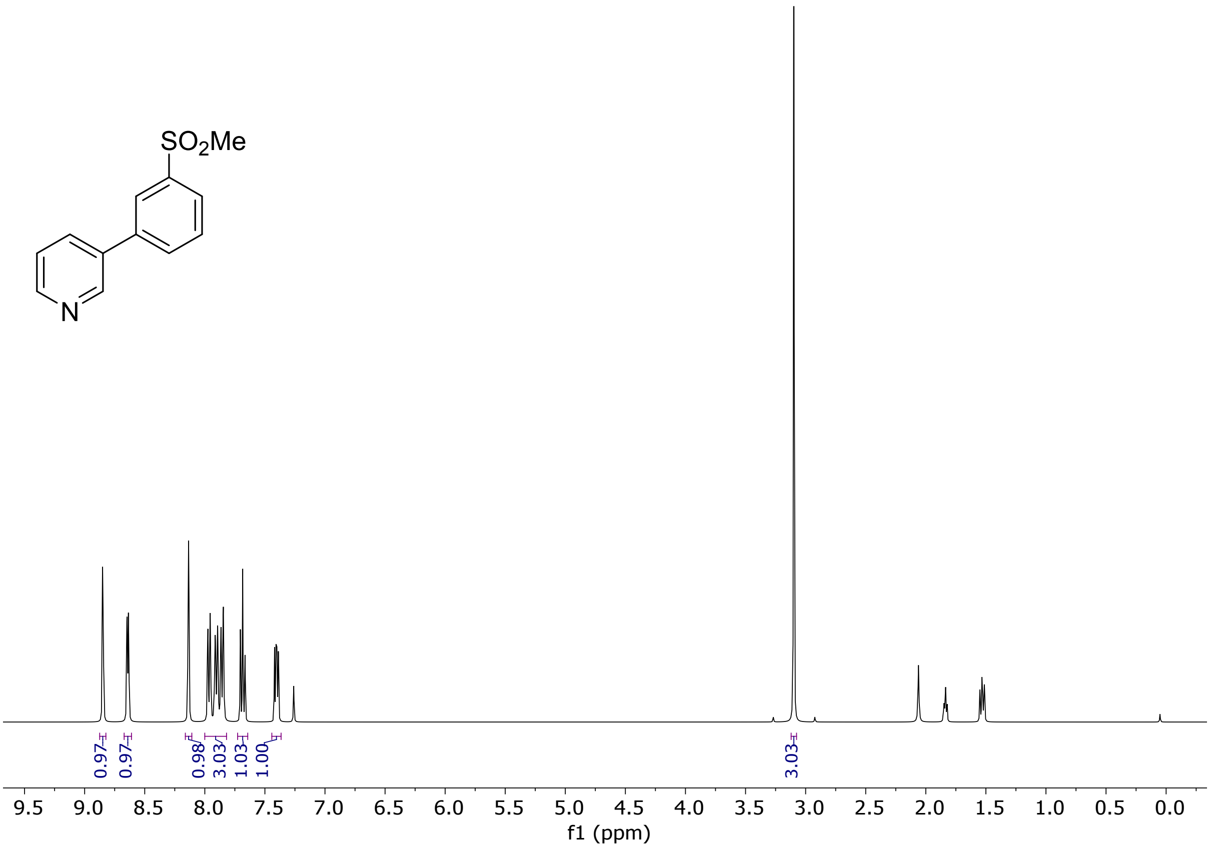

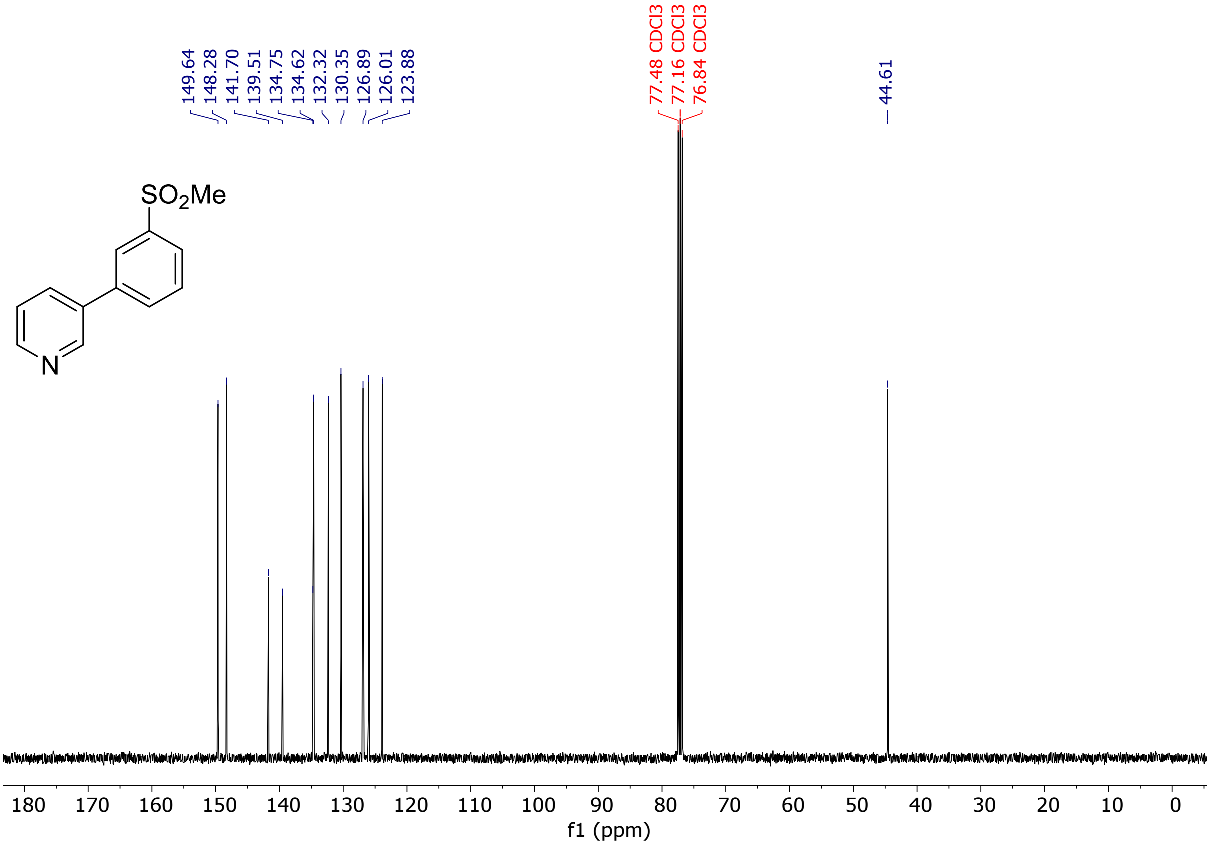

^{1}H & ^{13}C NMR spectra for 3-(4-fluorophenyl)pyridine

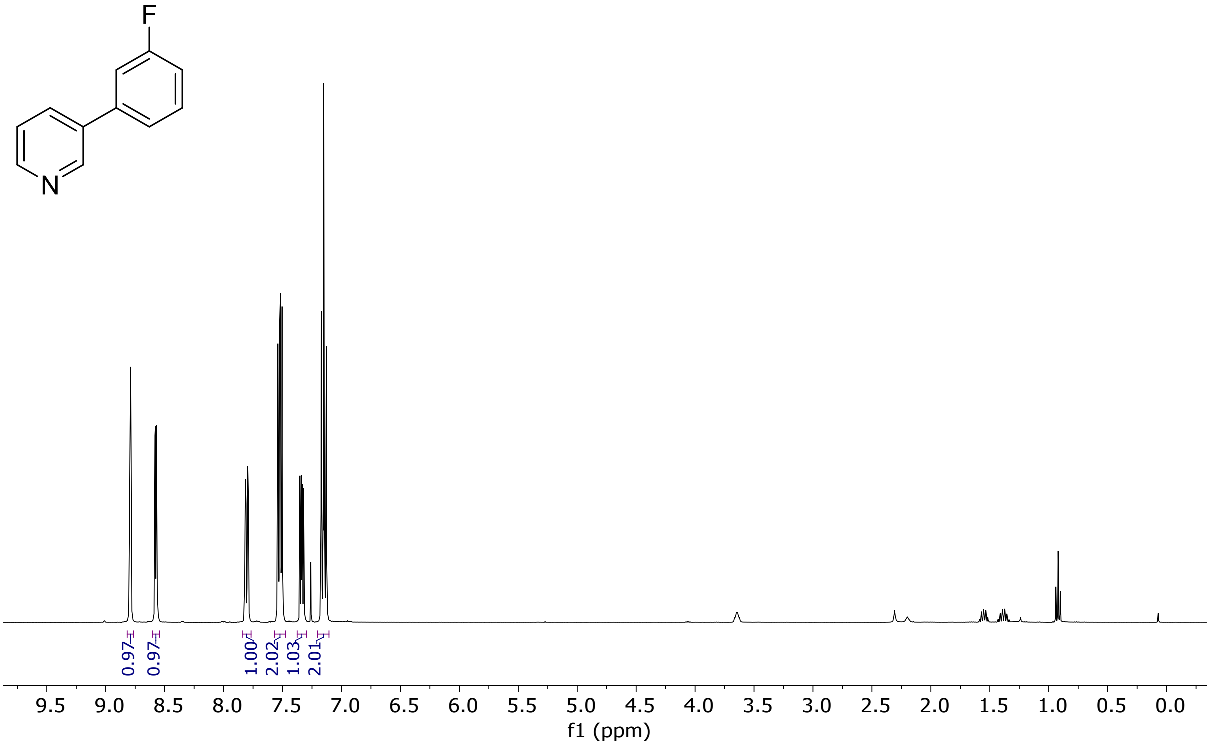

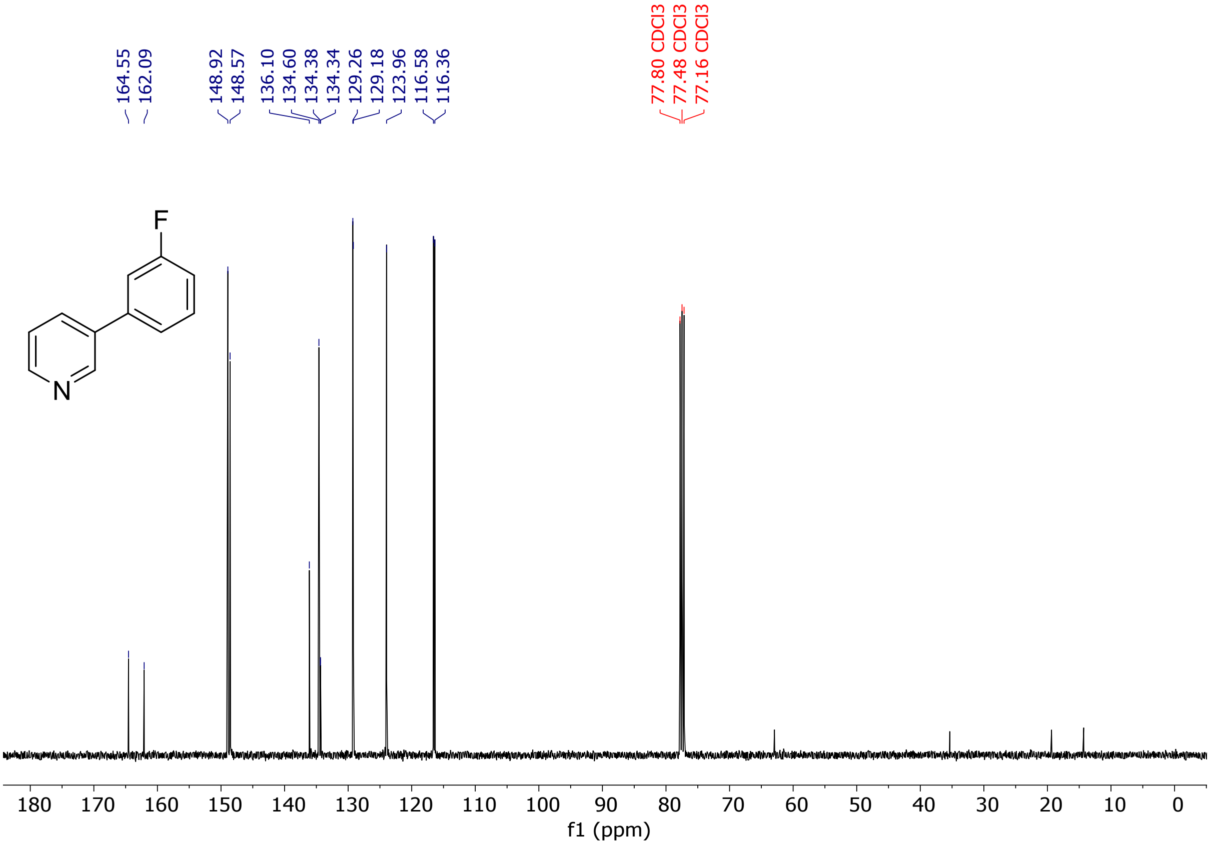

^{1}H & ^{13}C NMR spectra for 3-(naphthalen-2-yl)pyridine

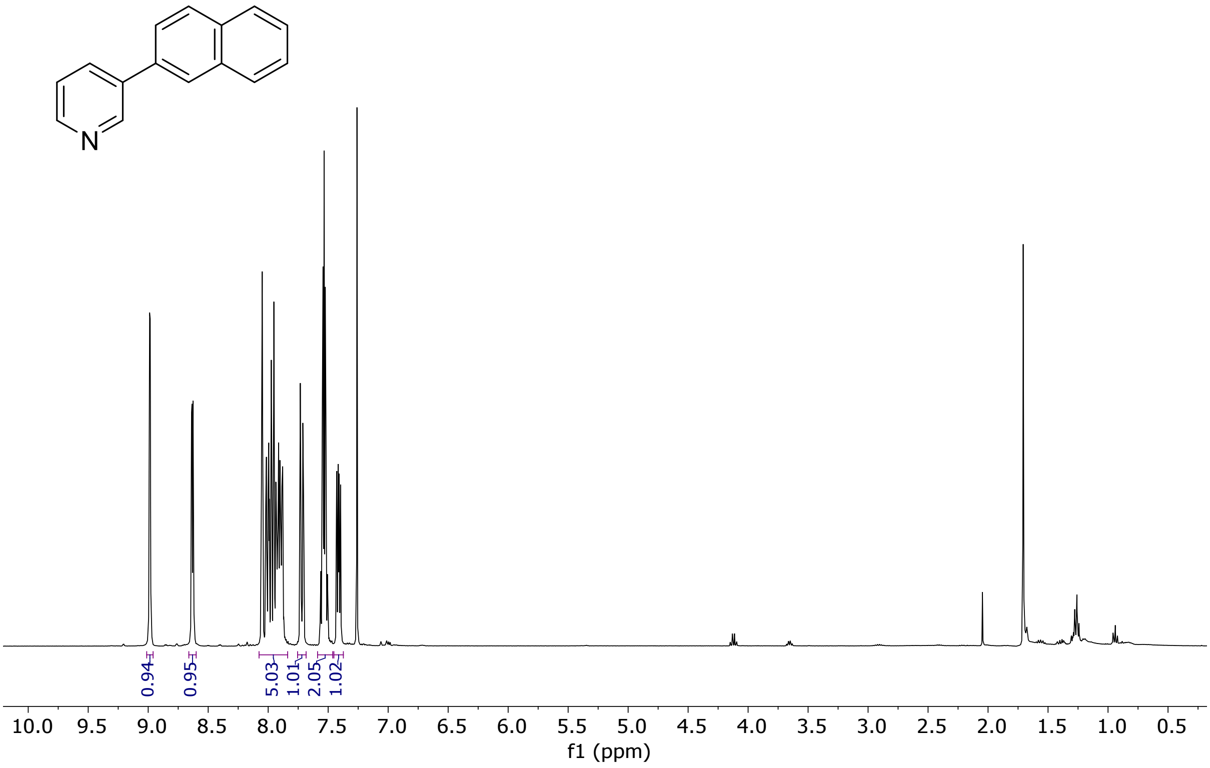

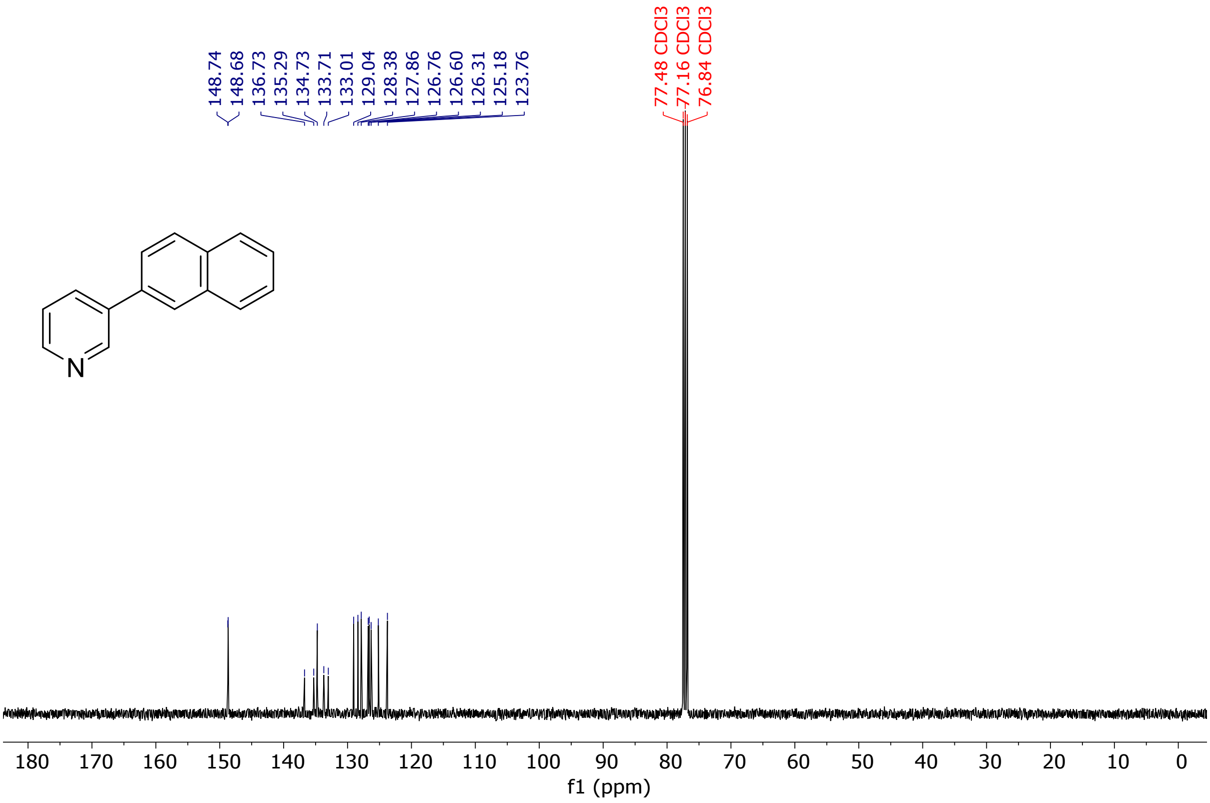

^{1}H & ^{13}C NMR spectra for 3-(furan-2-yl)pyridine

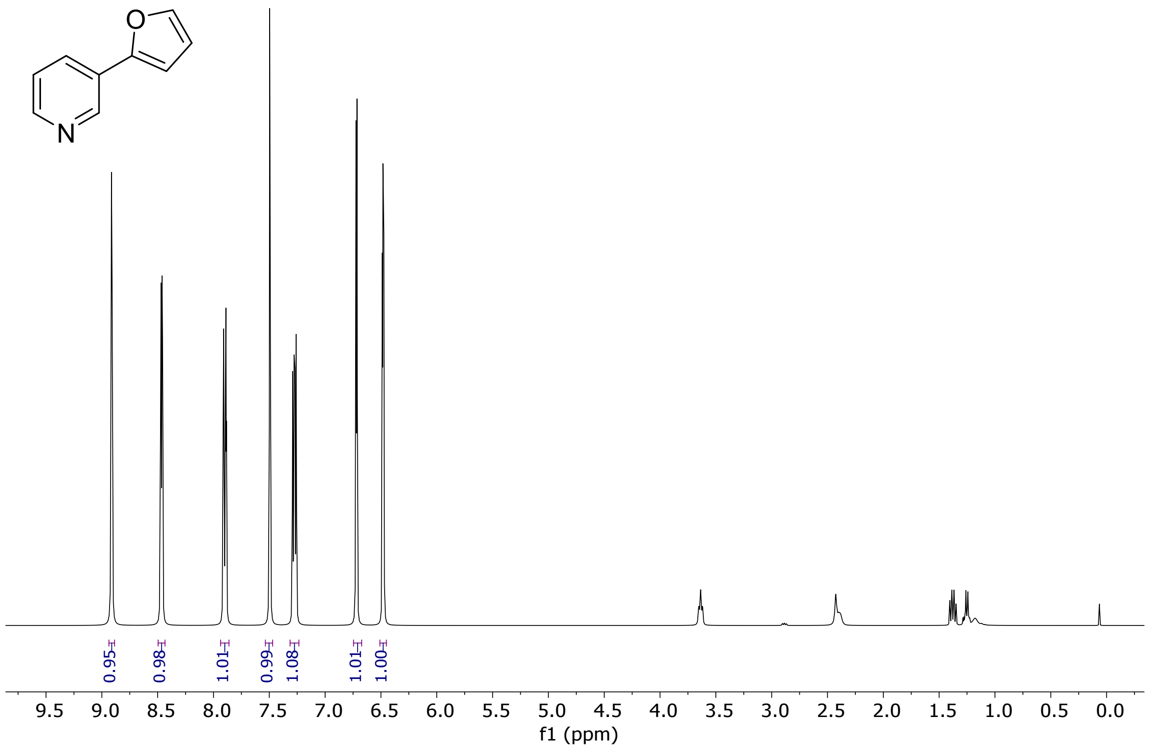

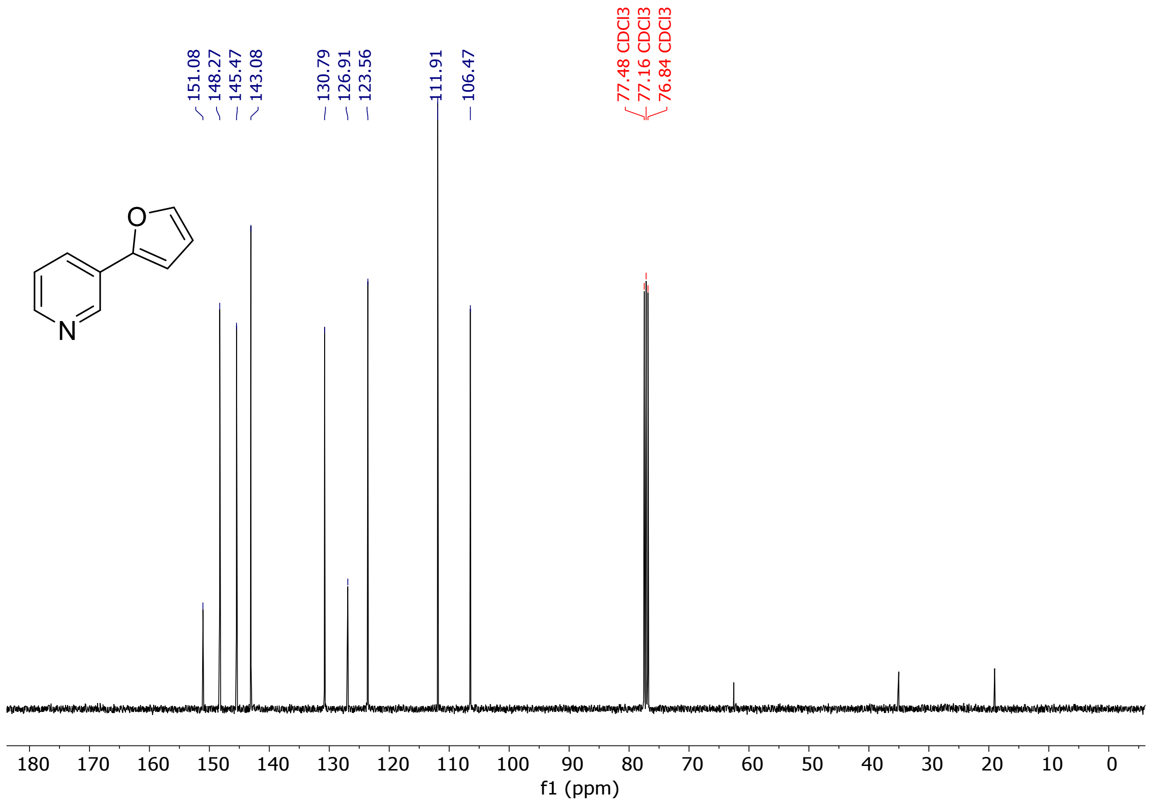

^{1}H & ^{13}C NMR spectra for 3-methyl-4-phenylpyridine (Crude)

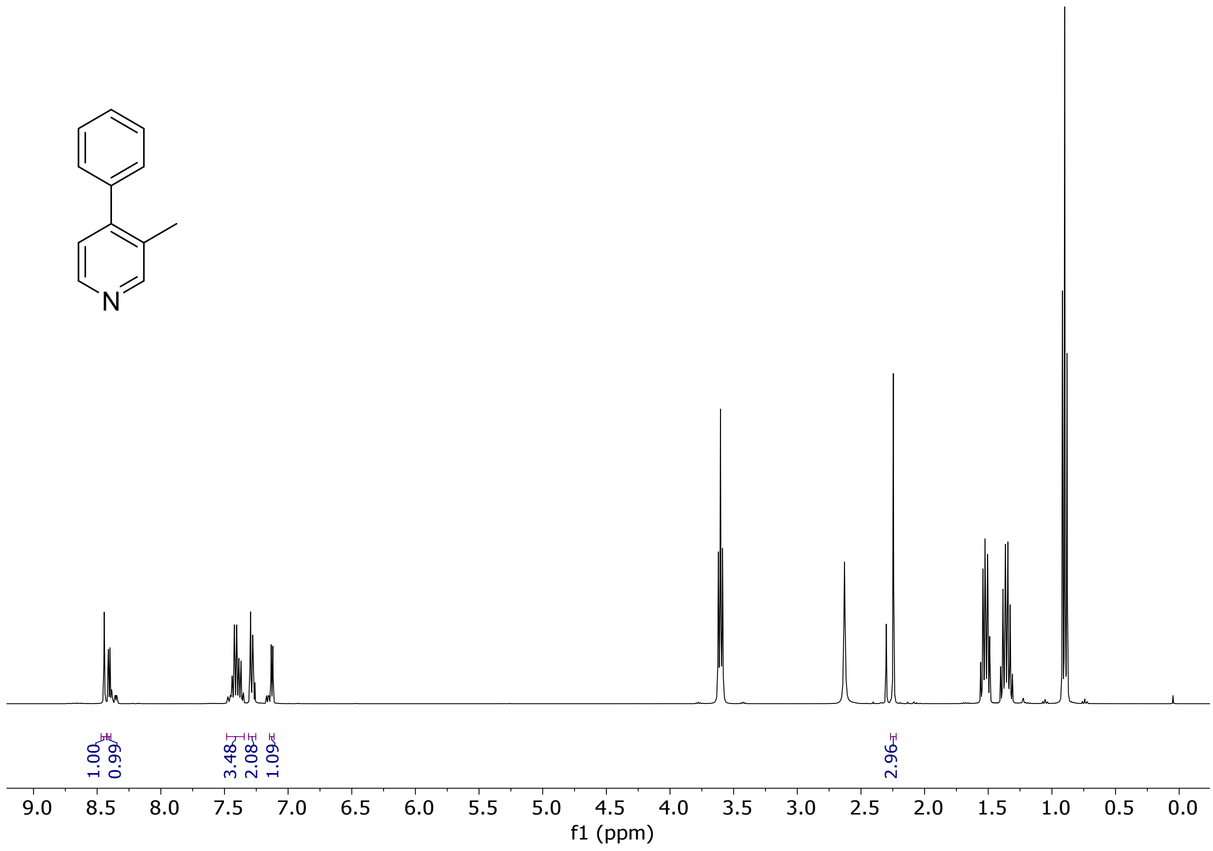

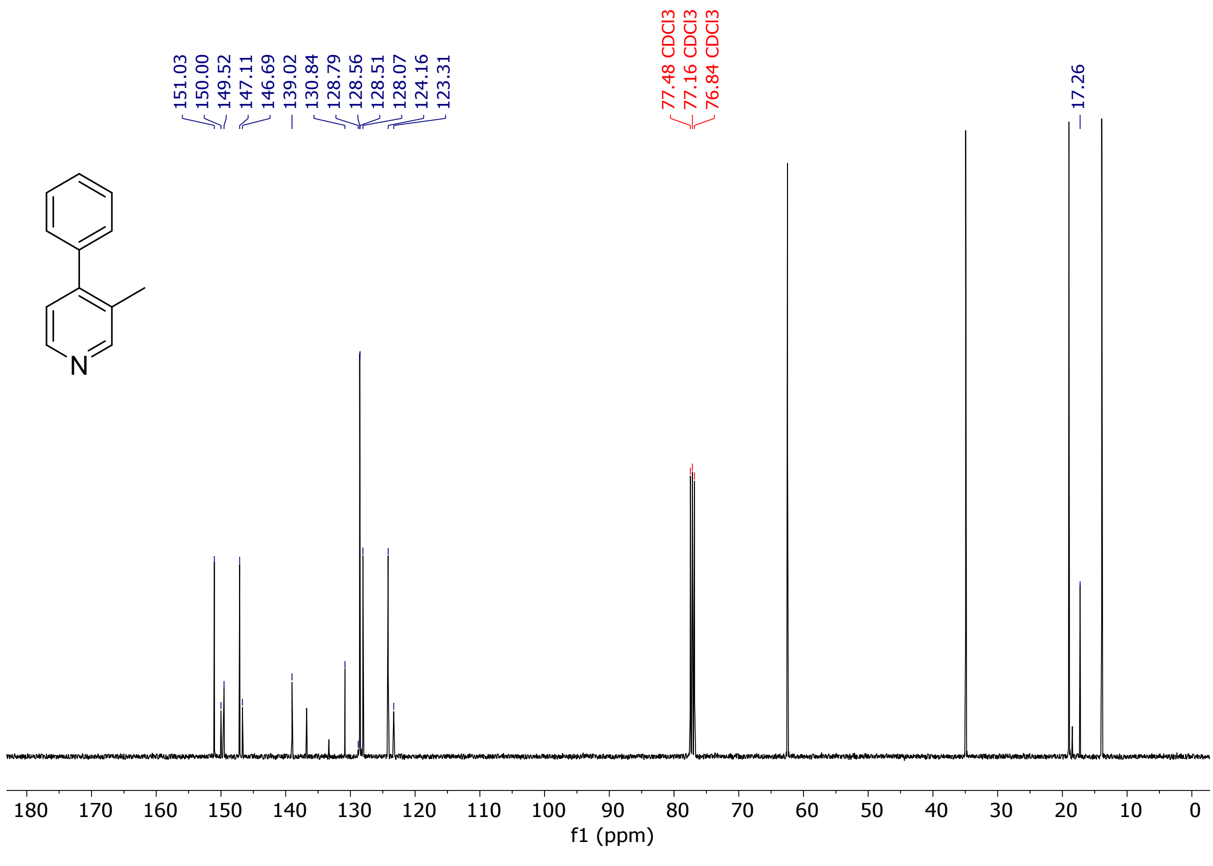

^{1}H & ^{13}C NMR spectra for *N*-(4-(pyridin-3-yl)phenyl)acetamide (Crude)

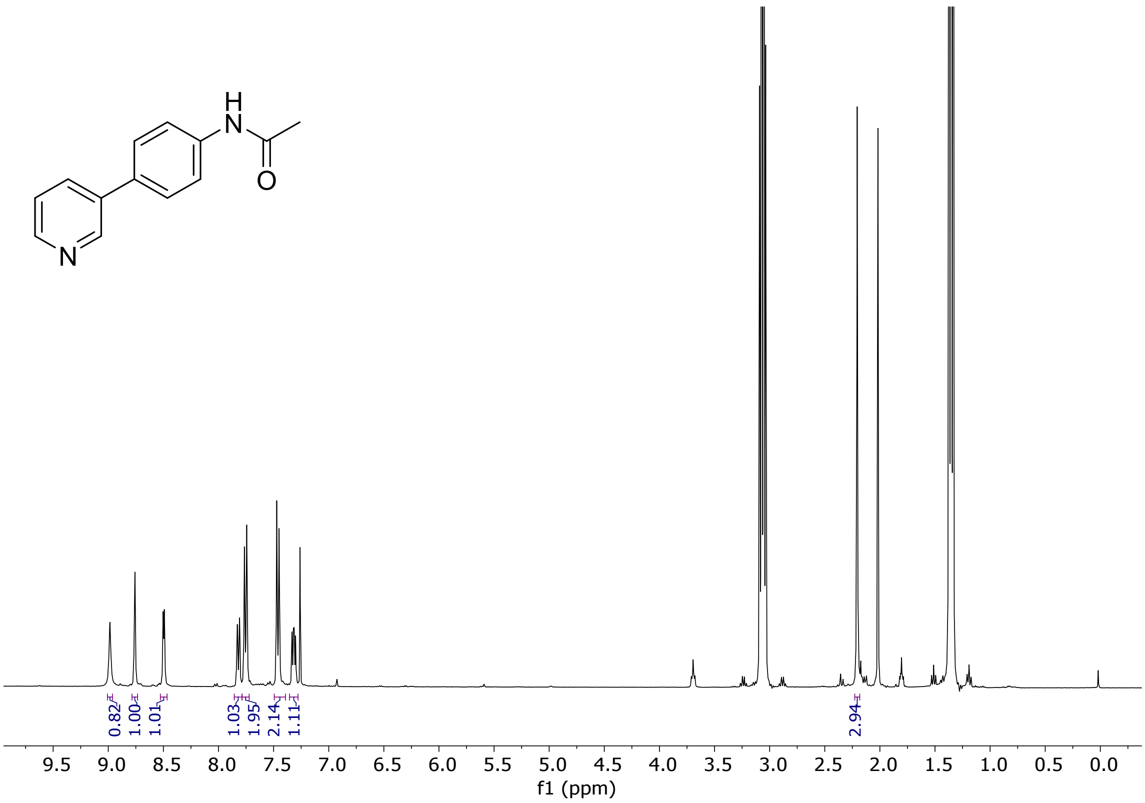

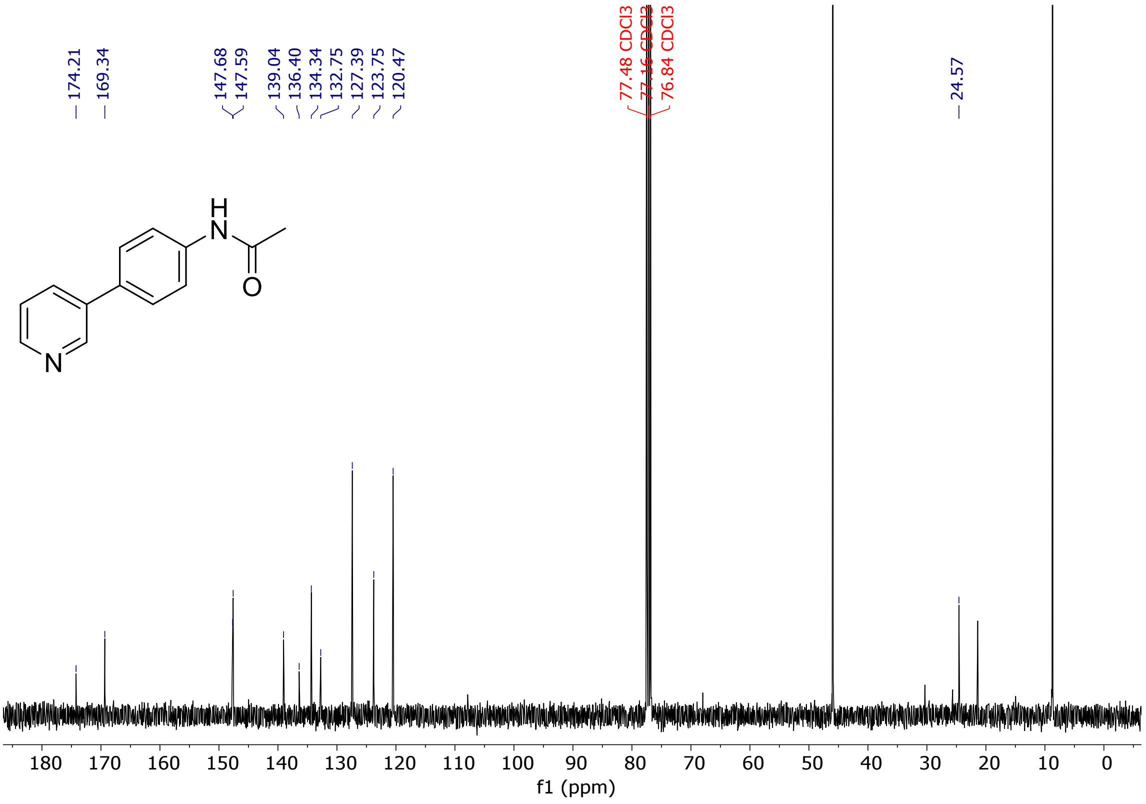

^{1}H & ^{13}C NMR spectra for 3-(3-methoxyphenyl)-1-propylpyridinium iodide (Crude-1a)

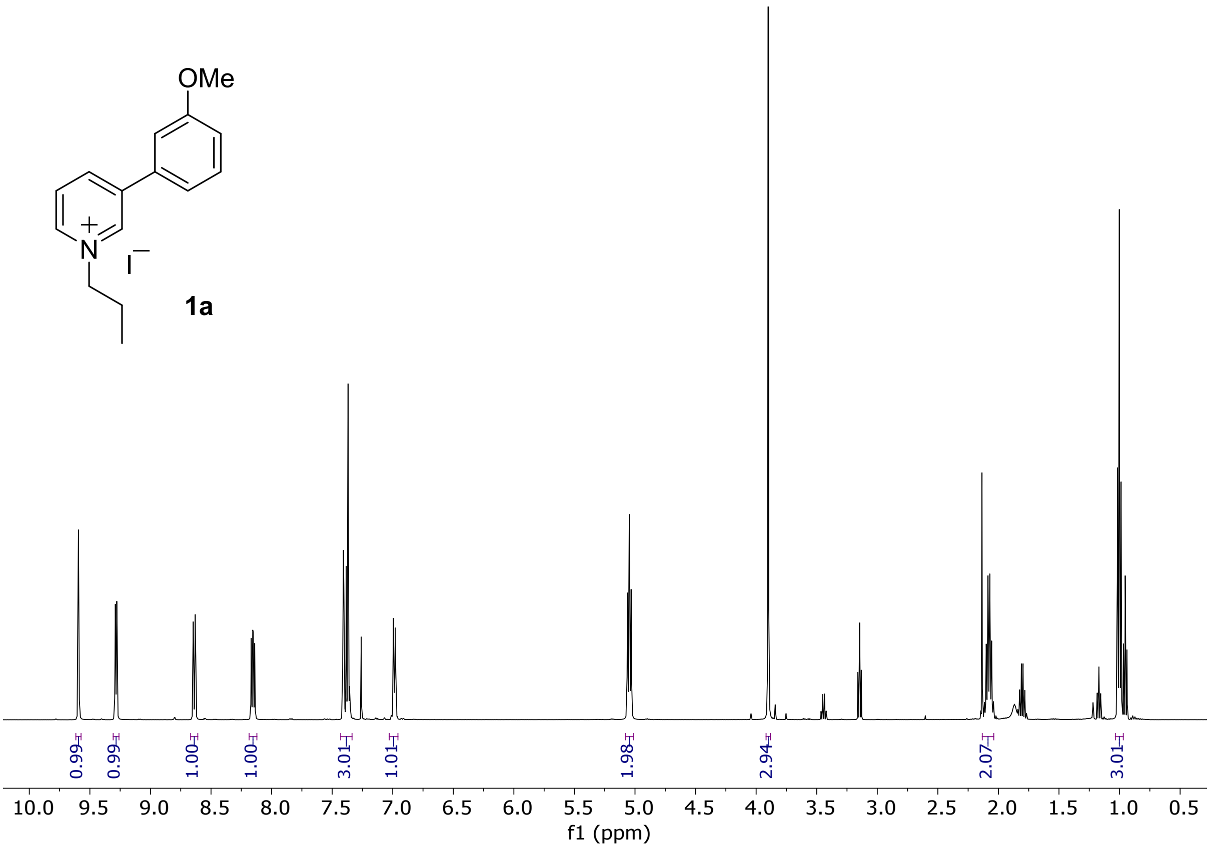

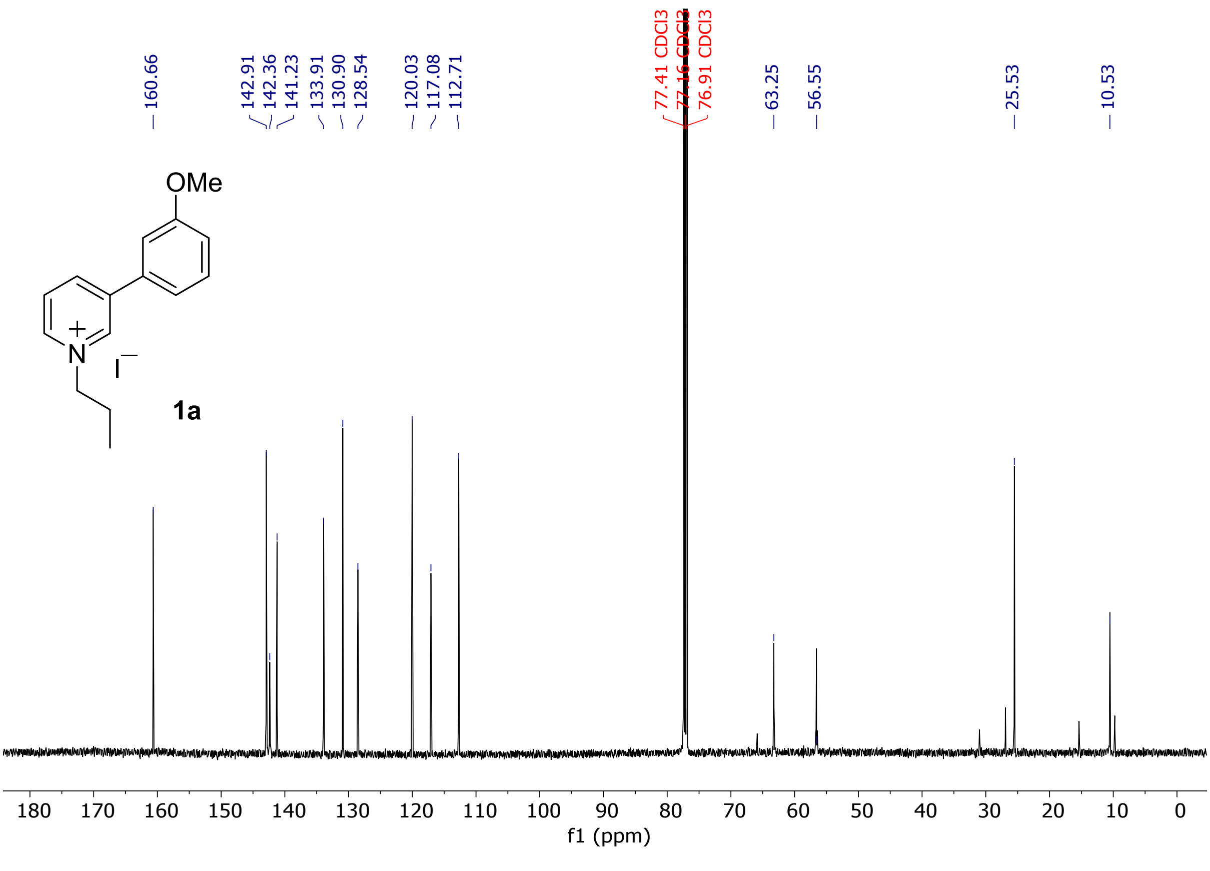

^{1}H & ^{13}C NMR spectra for 3-(3-(methylsulfonyl)phenyl)-1-propylpyridinium iodide (2a)

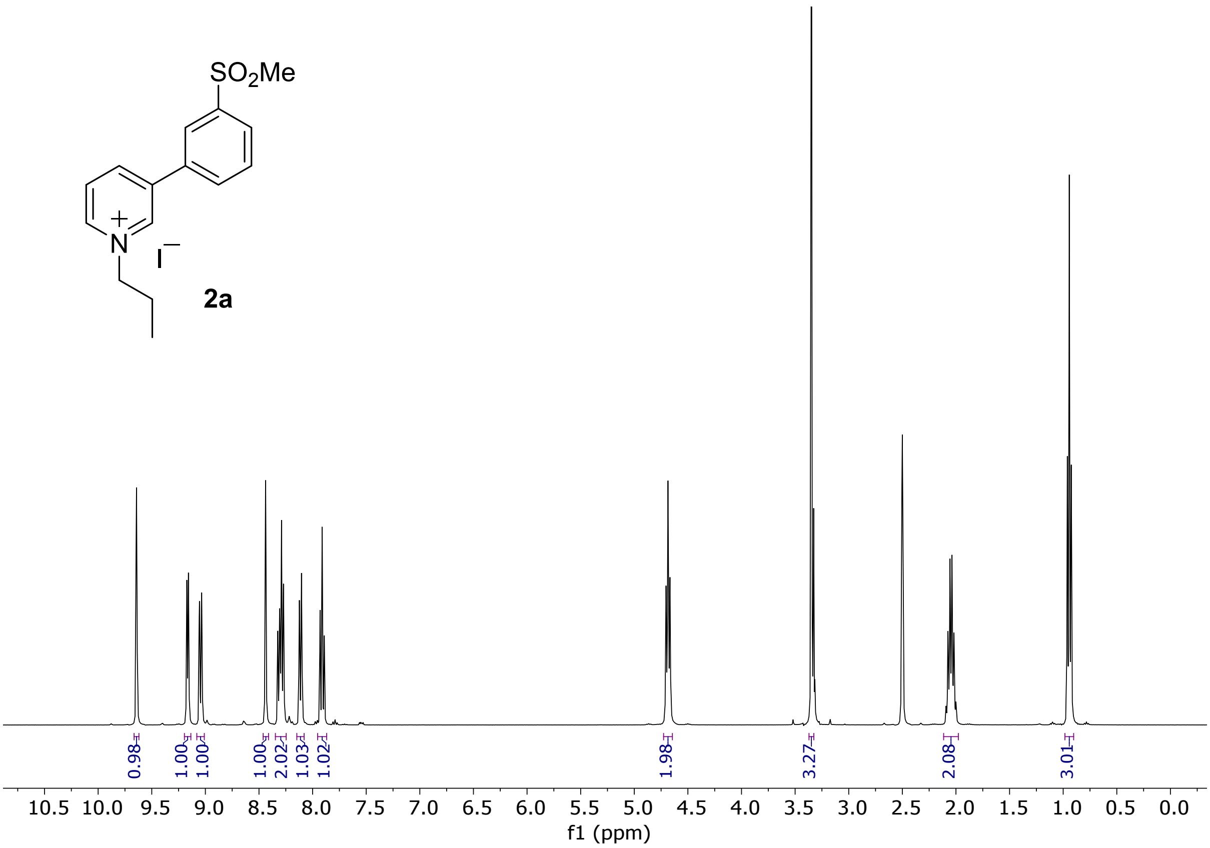

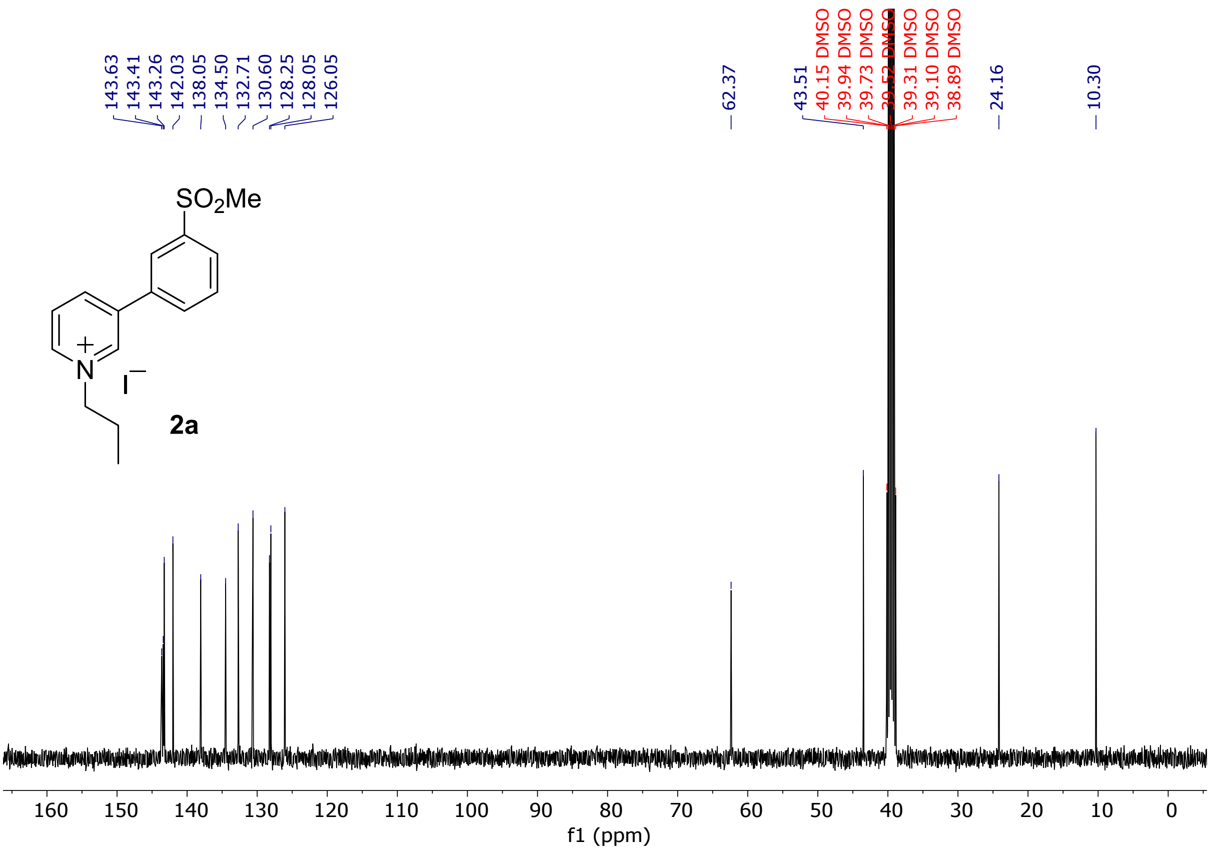

^{1}H & ^{13}C NMR spectra for 1-allyl-3-(4-bromophenyl)pyridinium bromide (3a)

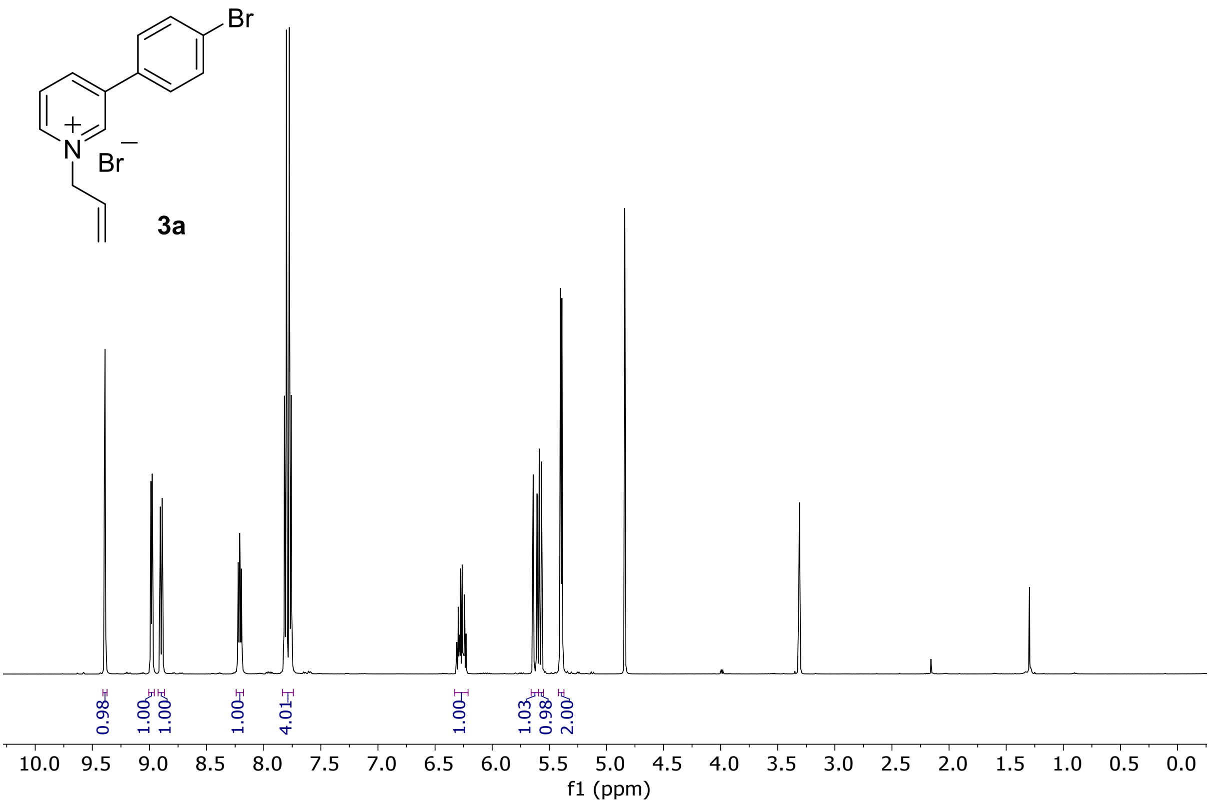

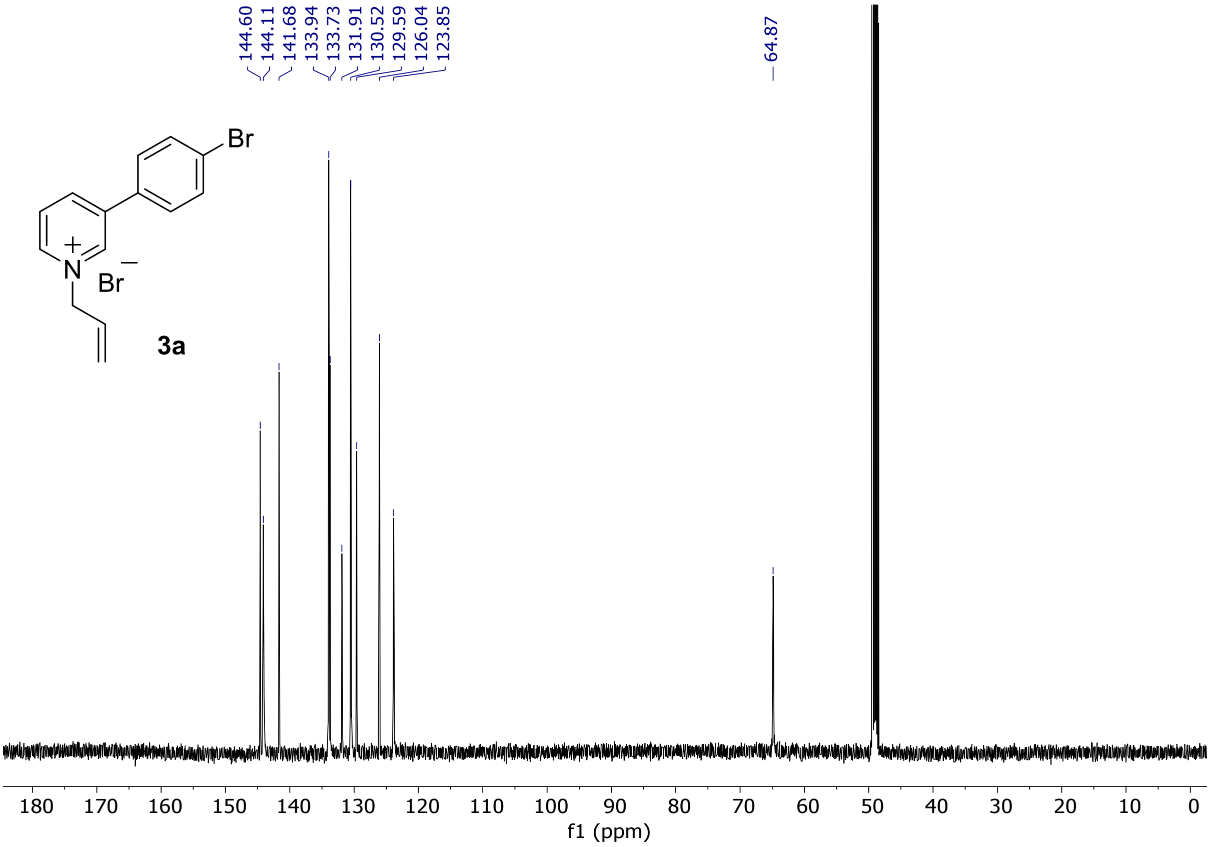

^{1}H & ^{13}C NMR spectra for 3-(4-acetamidophenyl)-1-allylpyridinium bromide (Crude-4a)

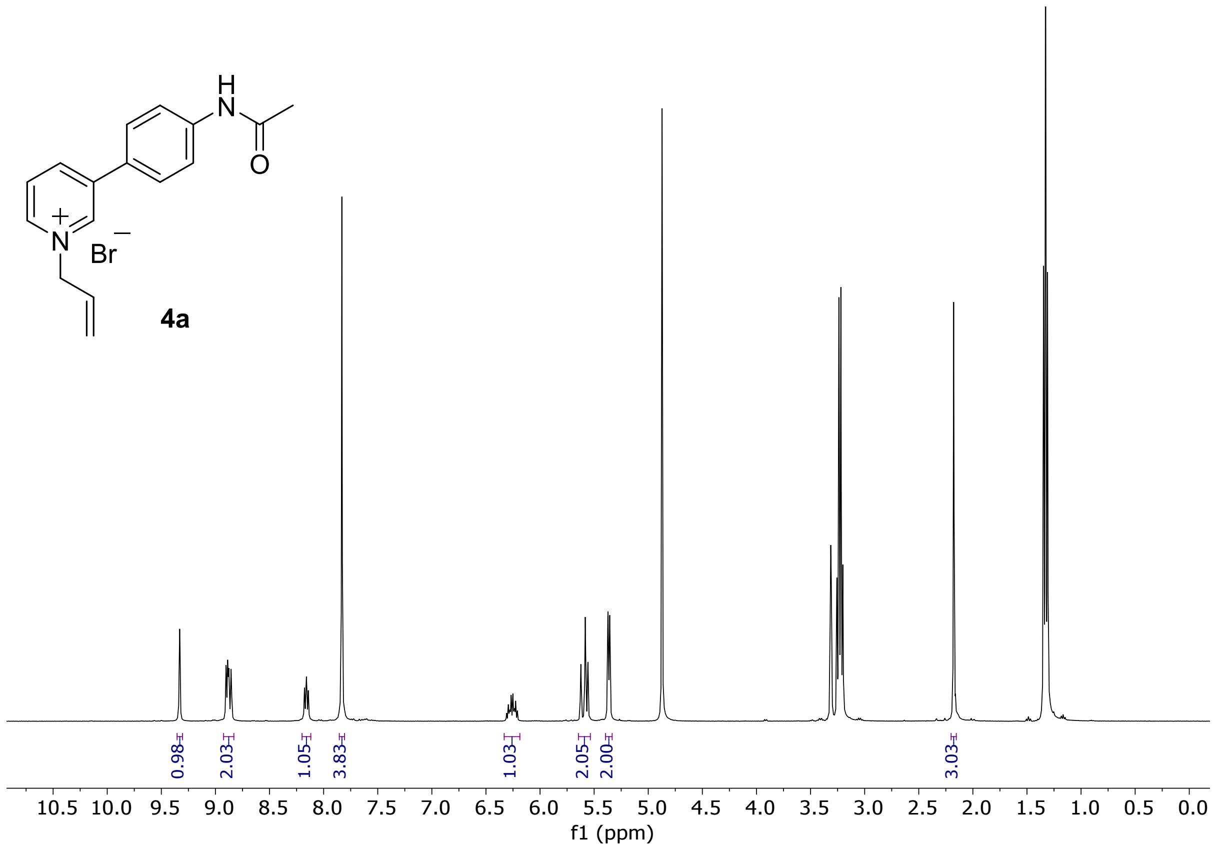

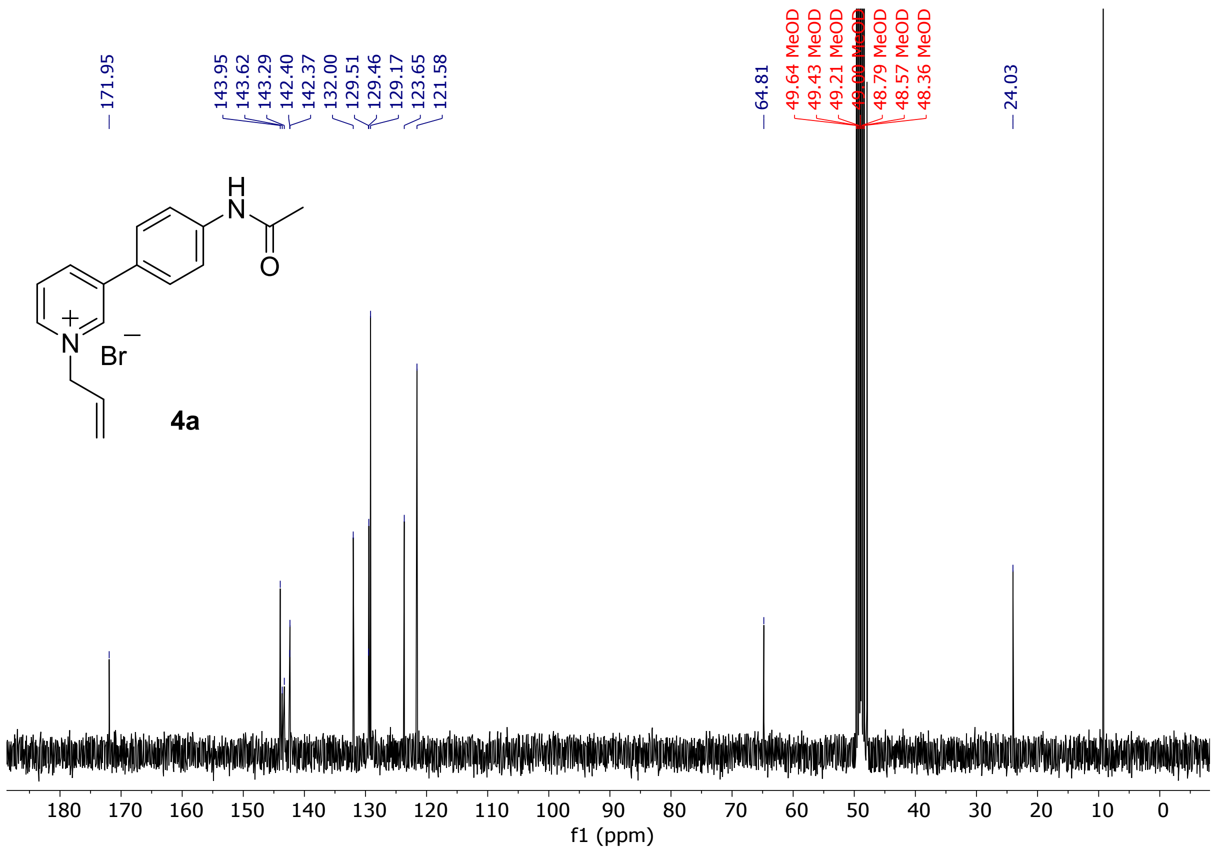

^{1}H & ^{13}C NMR spectra for 1-ethyl-3-phenylpyridinium iodide (5a)

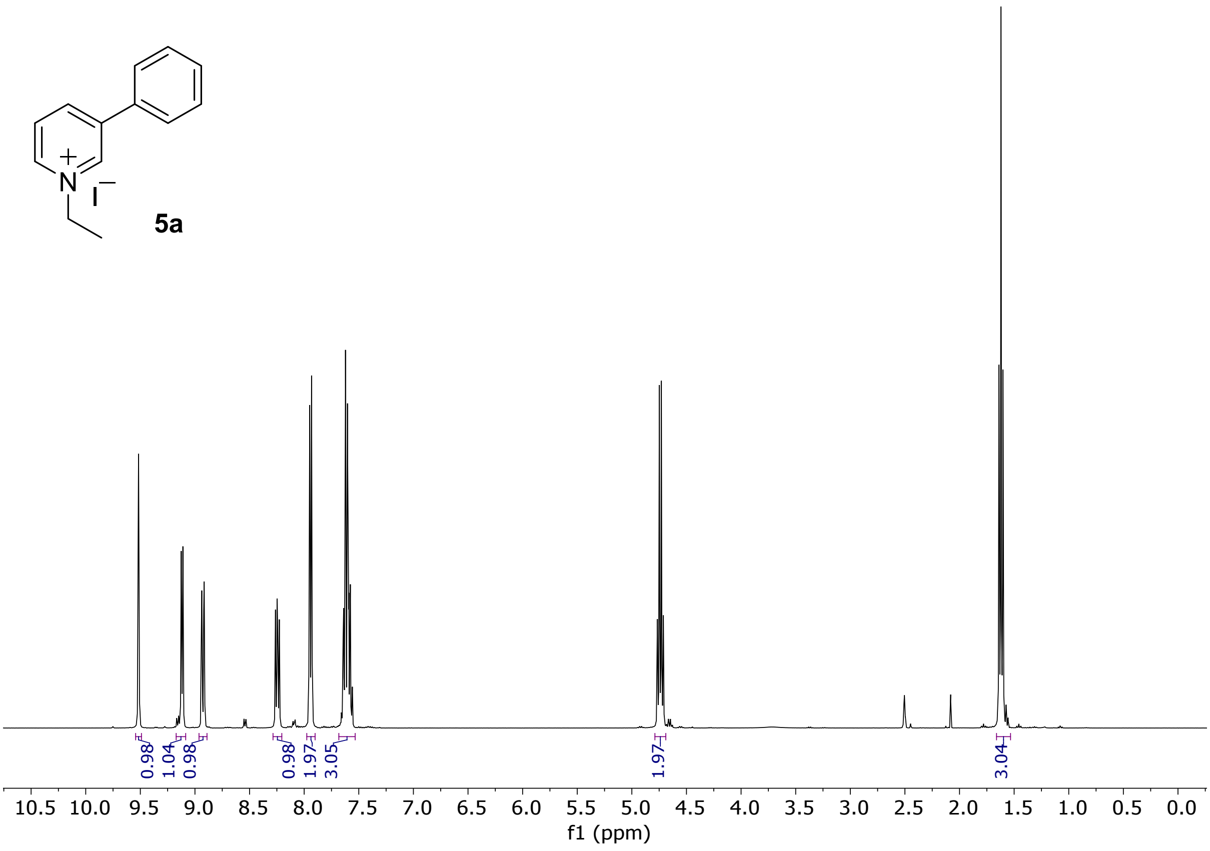

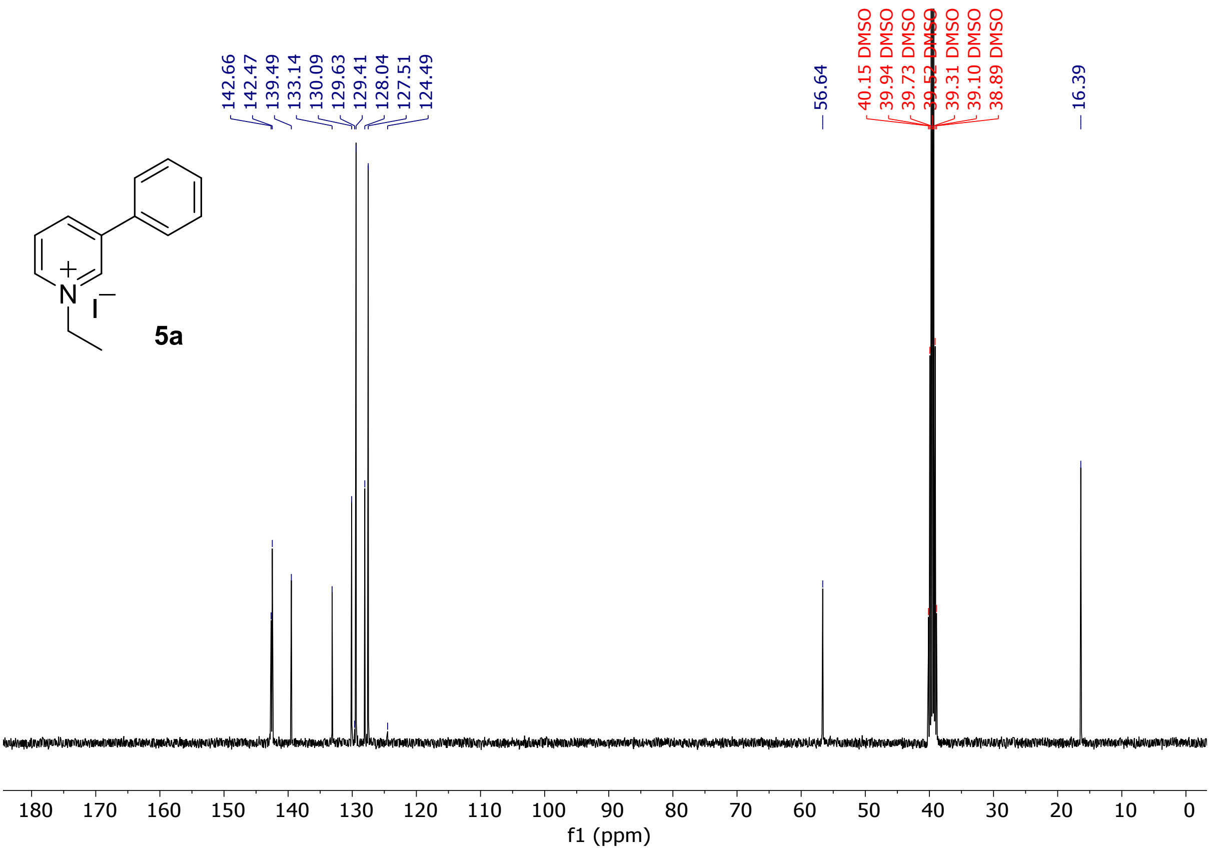

^{1}H & ^{13}C NMR spectra for 1-ethyl-3-(4-fluorophenyl)pyridinium iodide (6a)

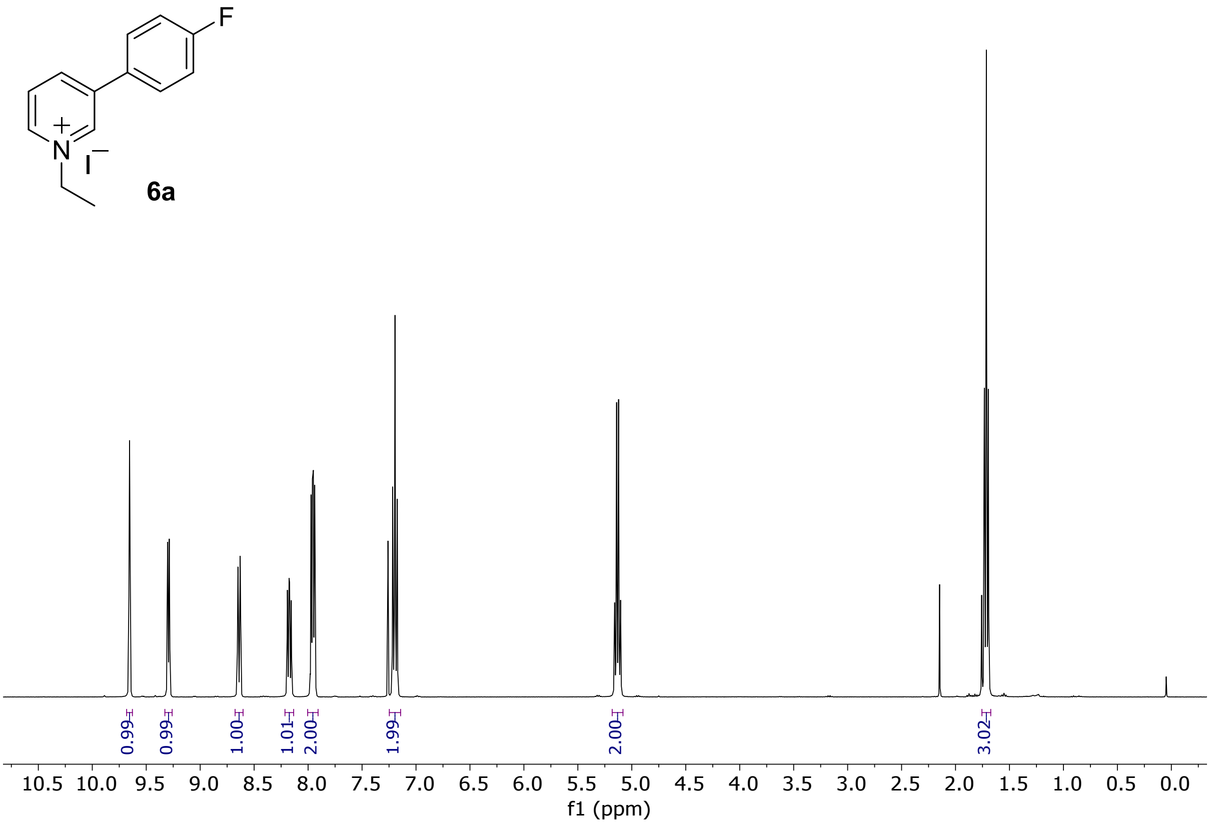

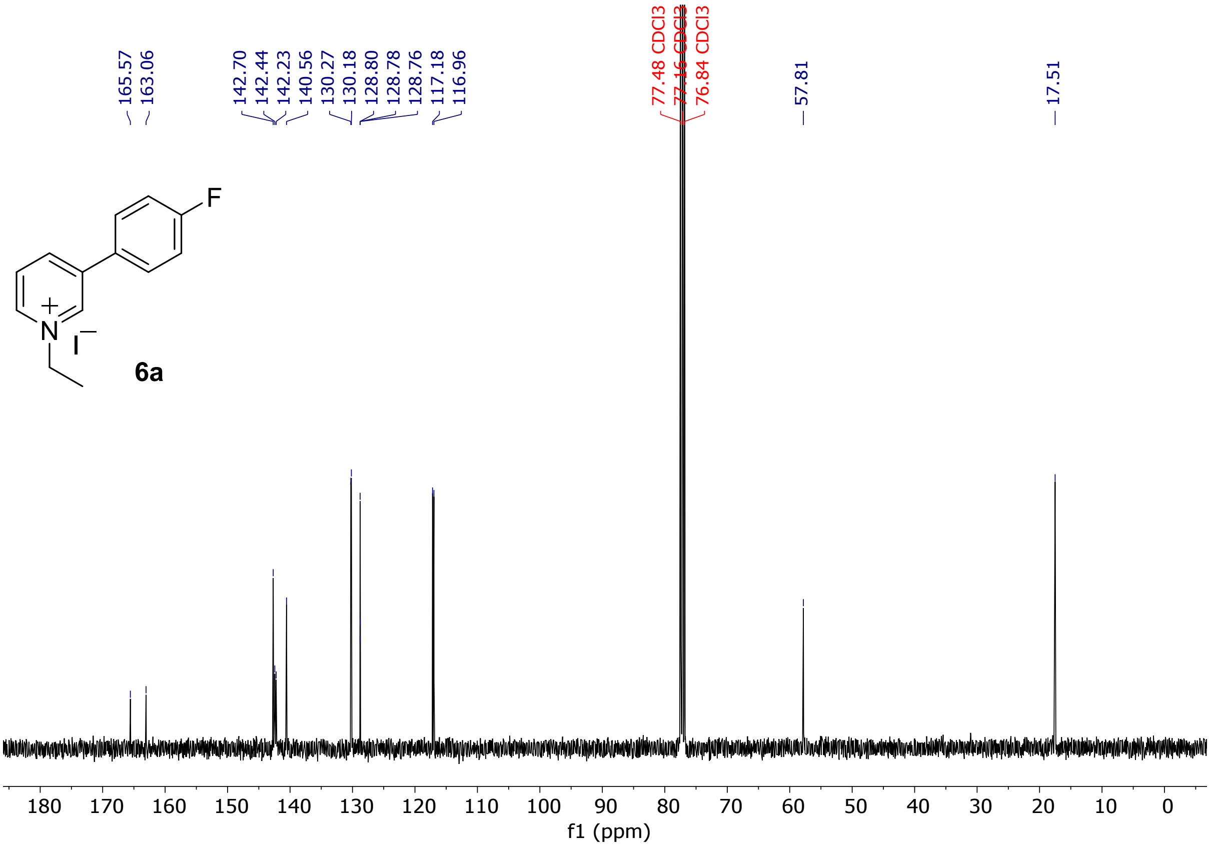

^{1}H & ^{13}C NMR spectra for 1-ethyl-3-(4-methoxyphenyl)pyridinium iodide (7a)

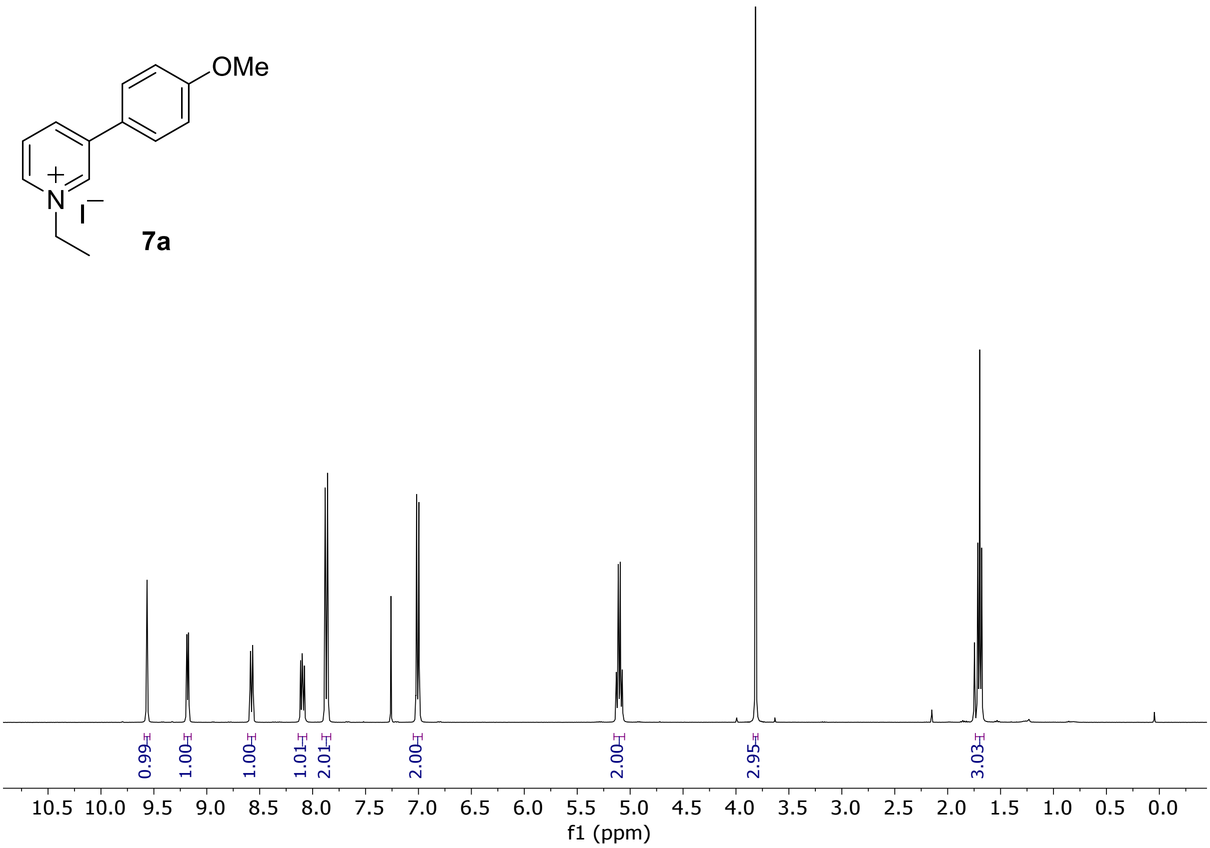

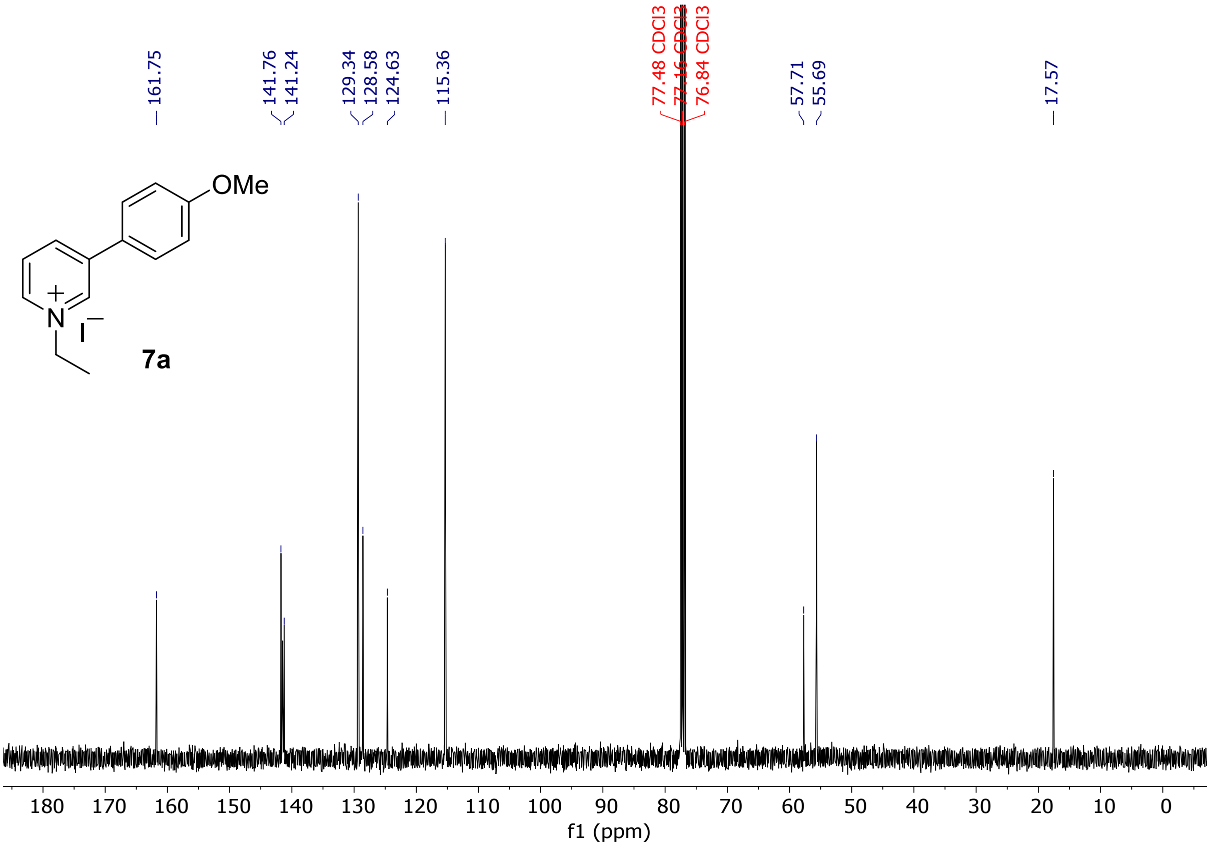

^{1}H & ^{13}C NMR spectra for 1-ethyl-3-(furan-2-yl)pyridinium iodide (8a)

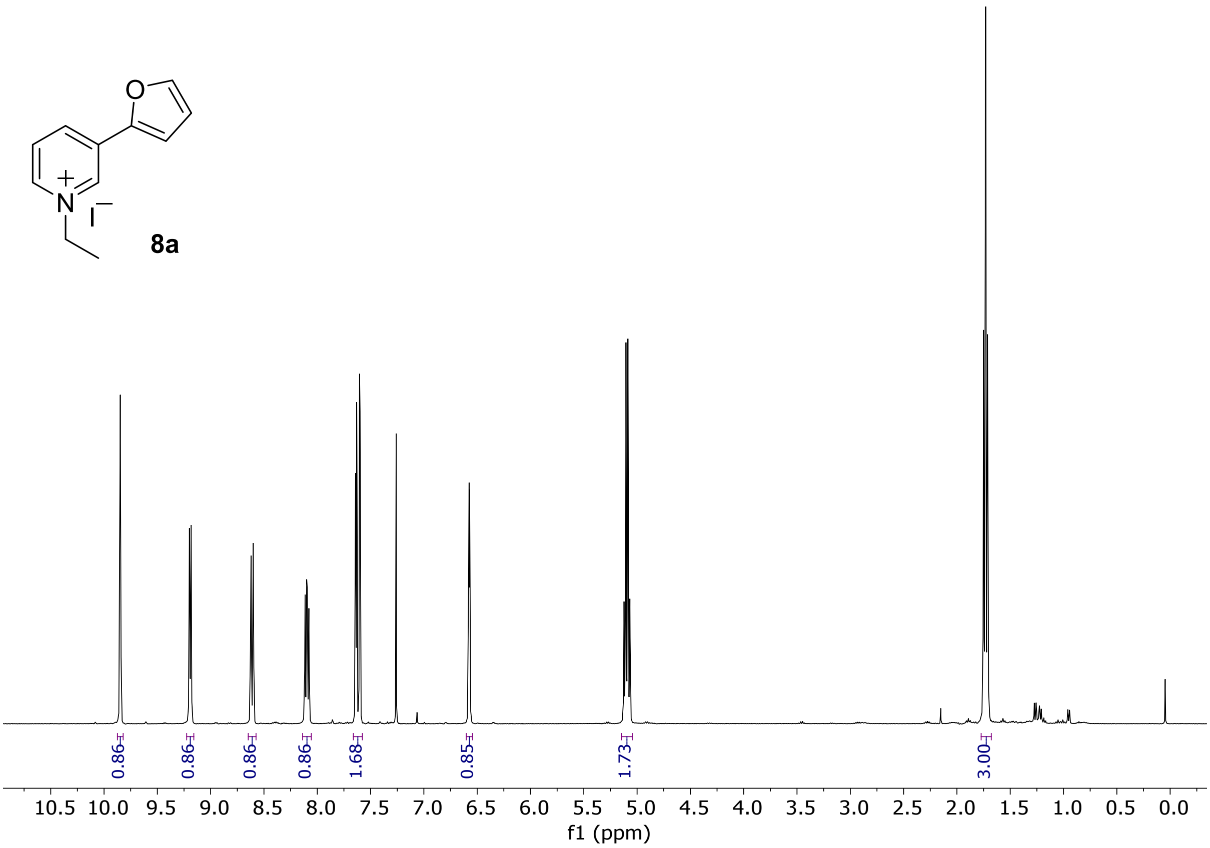

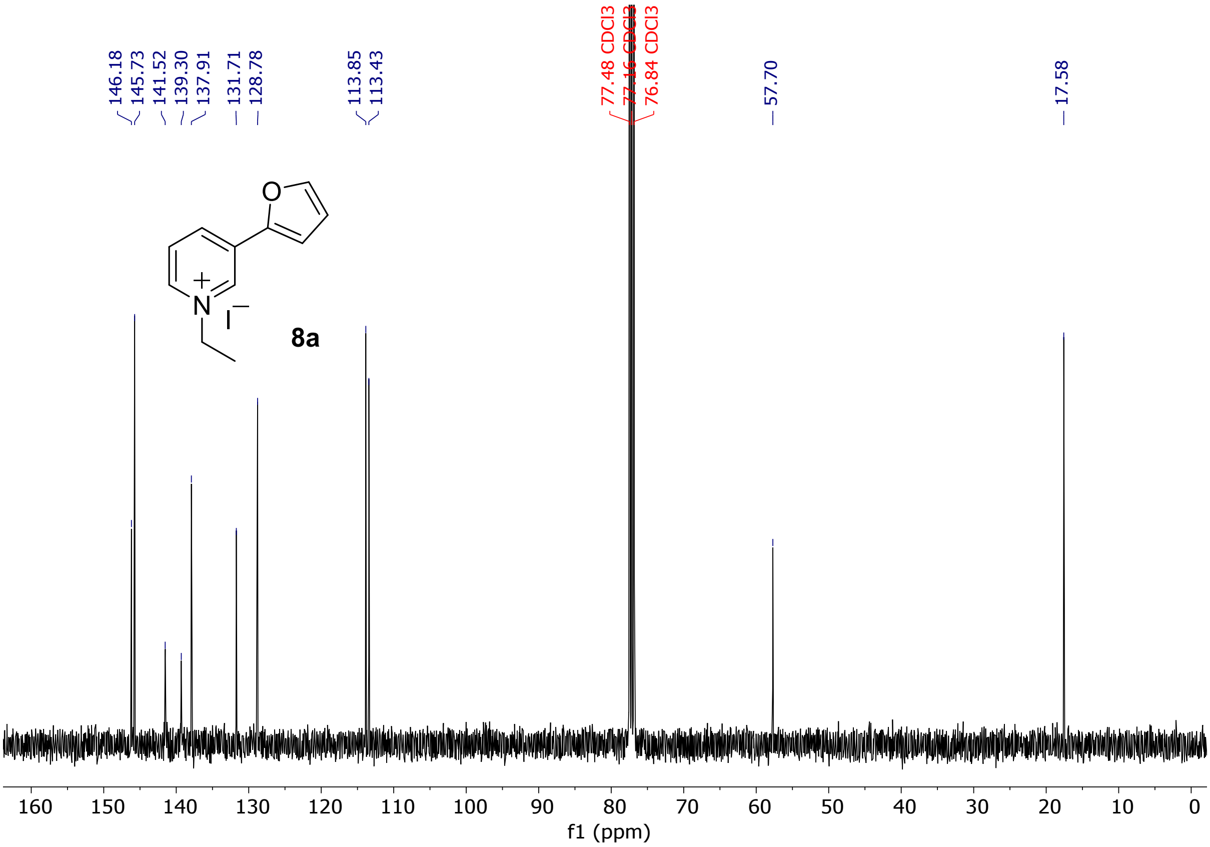

^{1}H & ^{13}C NMR spectra for 1-ethyl-3-(thiophen-2-yl)pyridinium iodide (9a)

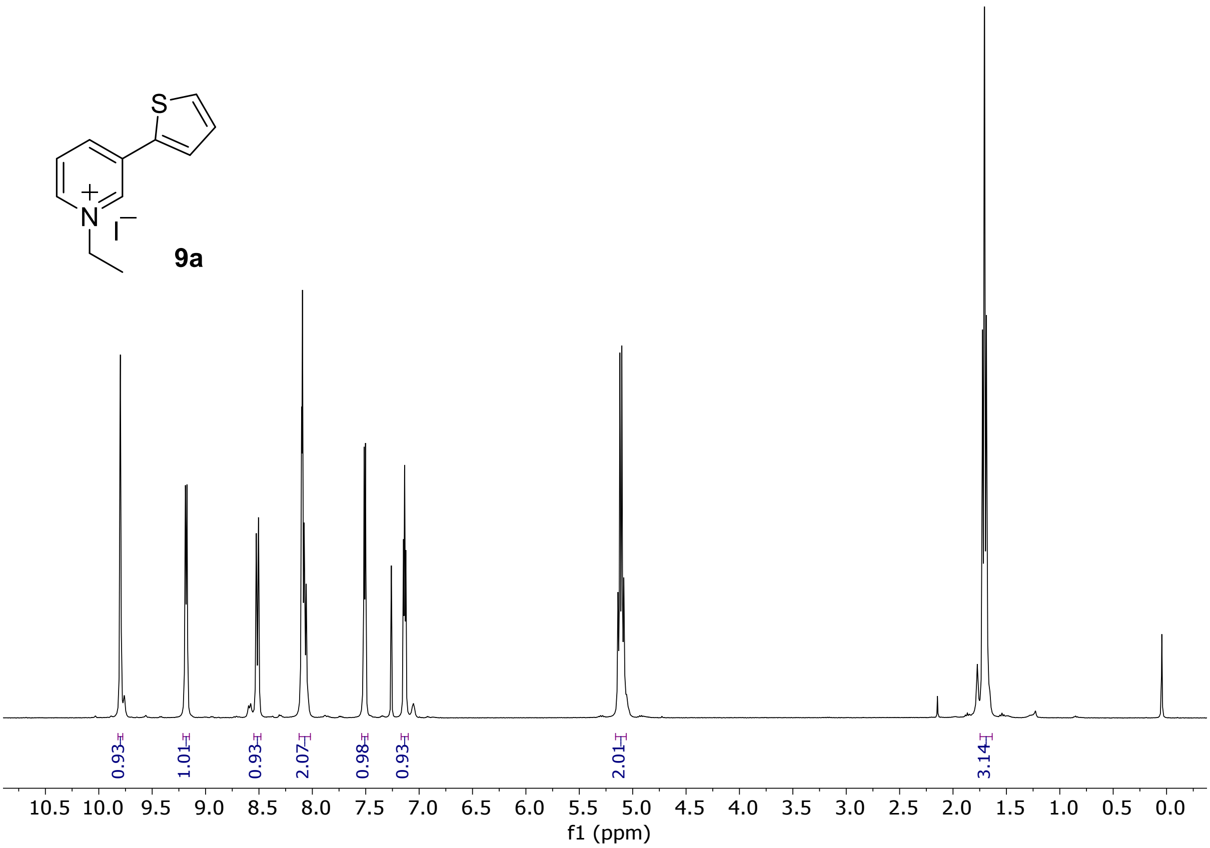

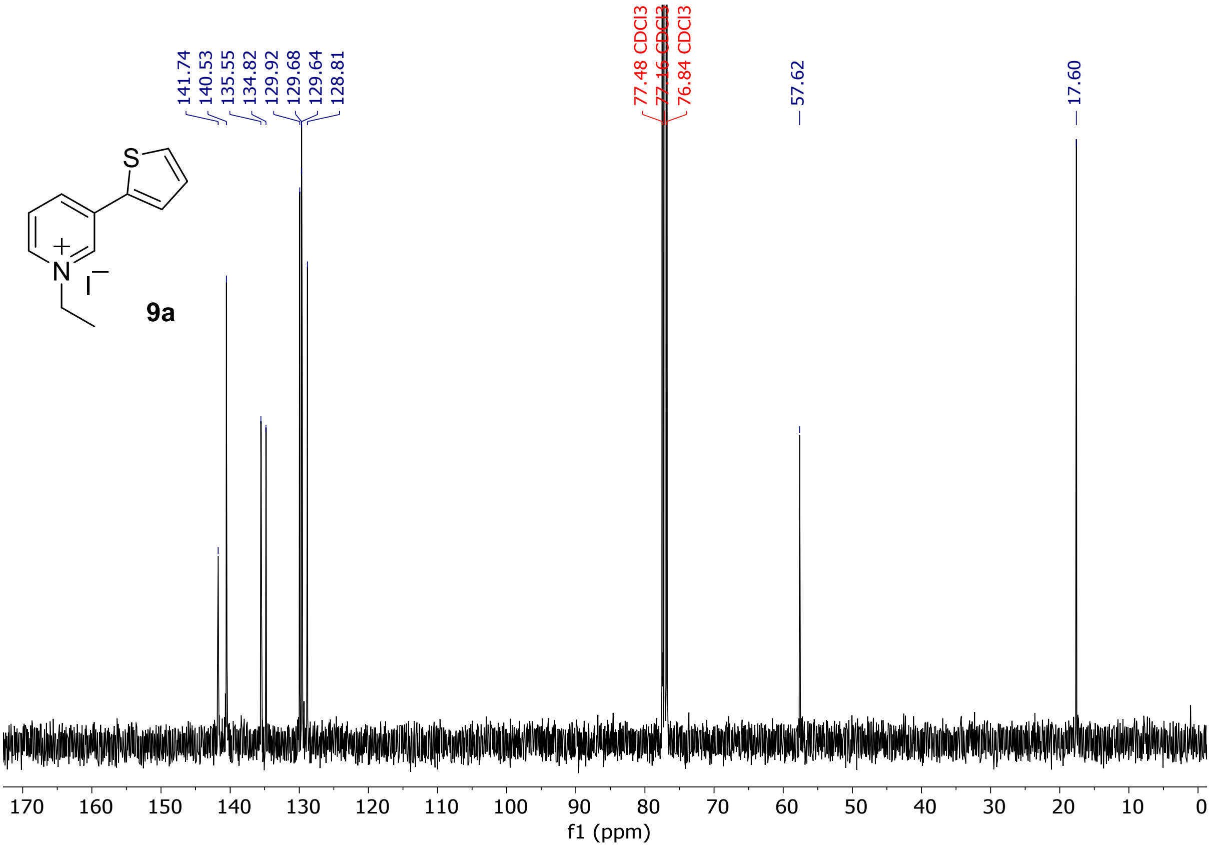

^{1}H & ^{13}C NMR spectra for 1-ethyl-3-(naphthalen-2-yl)pyridinium iodide (10a)

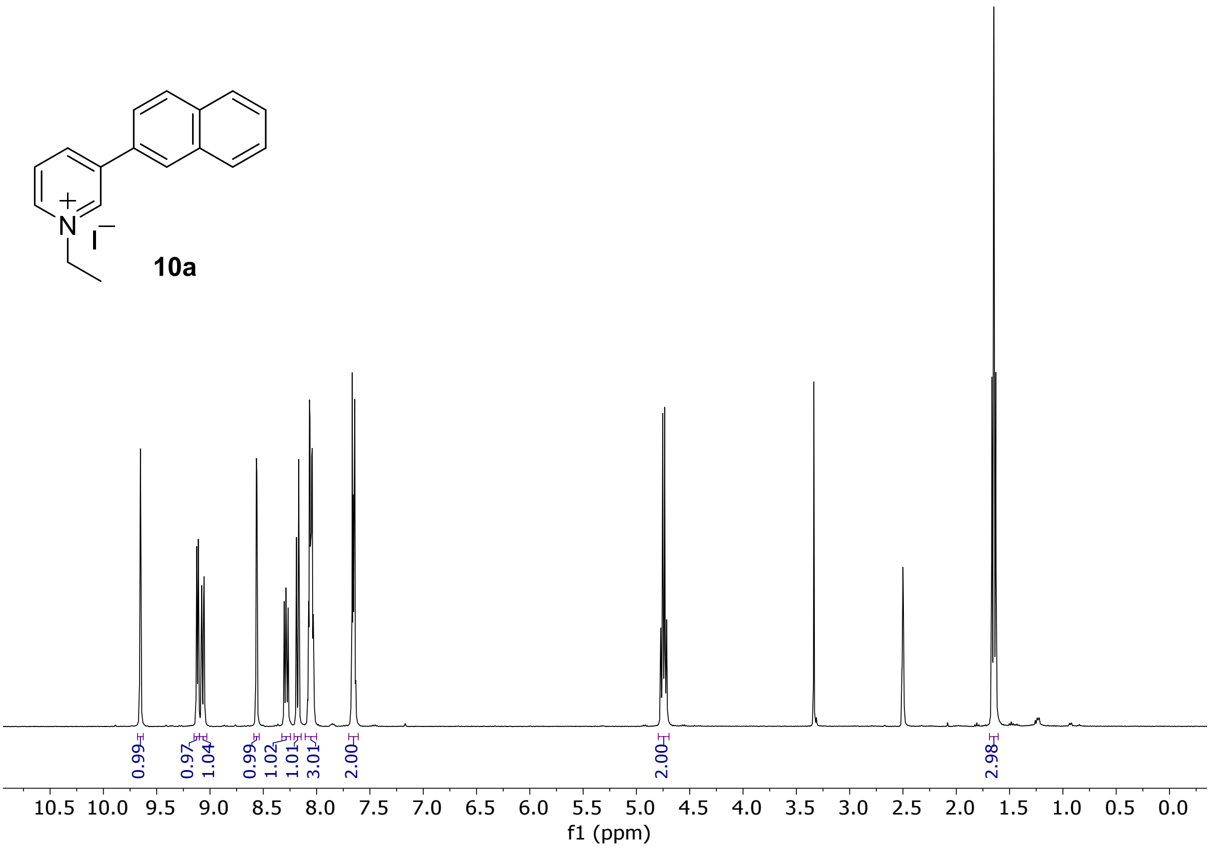

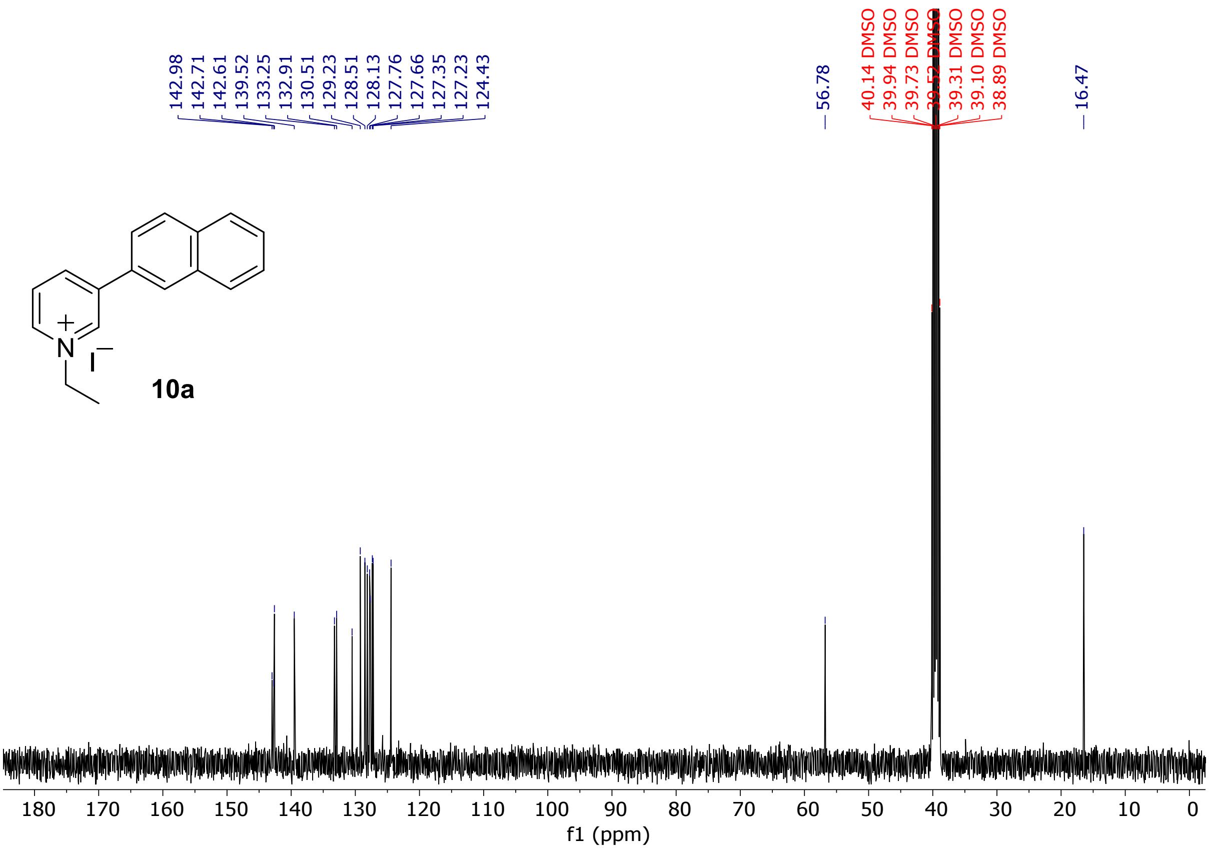

^{1}H & ^{13}C NMR spectra for 1-methyl-3-phenylpyridinium iodide (11a)

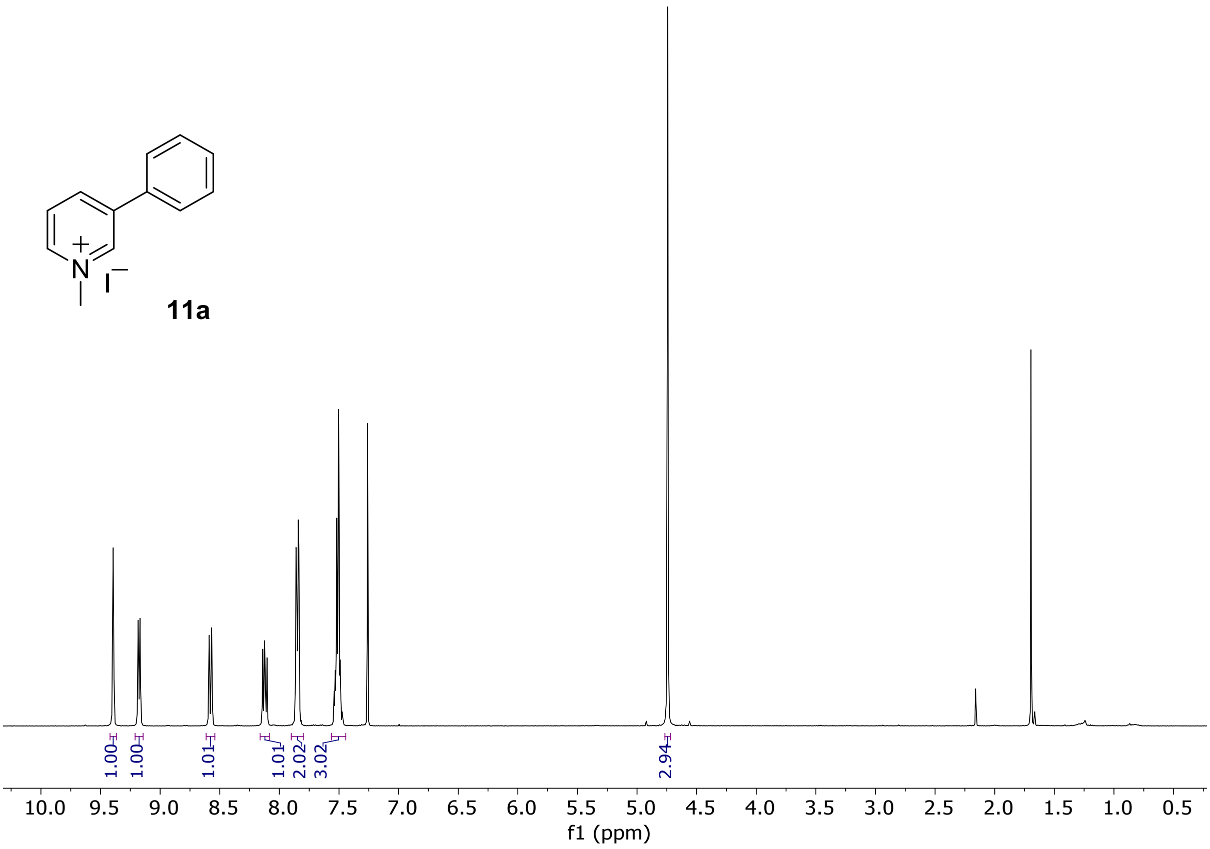

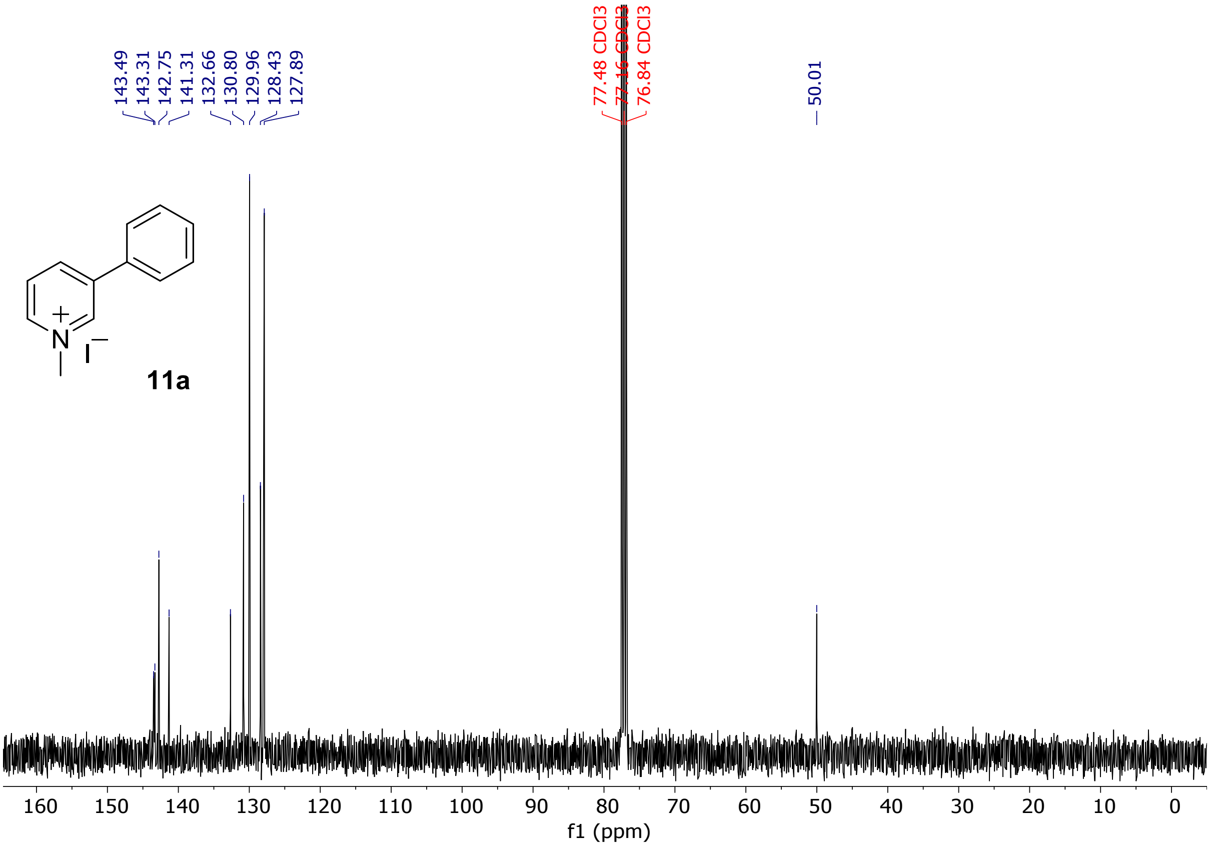

^{1}H & ^{13}C NMR spectra for 3-phenyl-1-(prop-2-yn-1-yl)pyridinium bromide (12a)

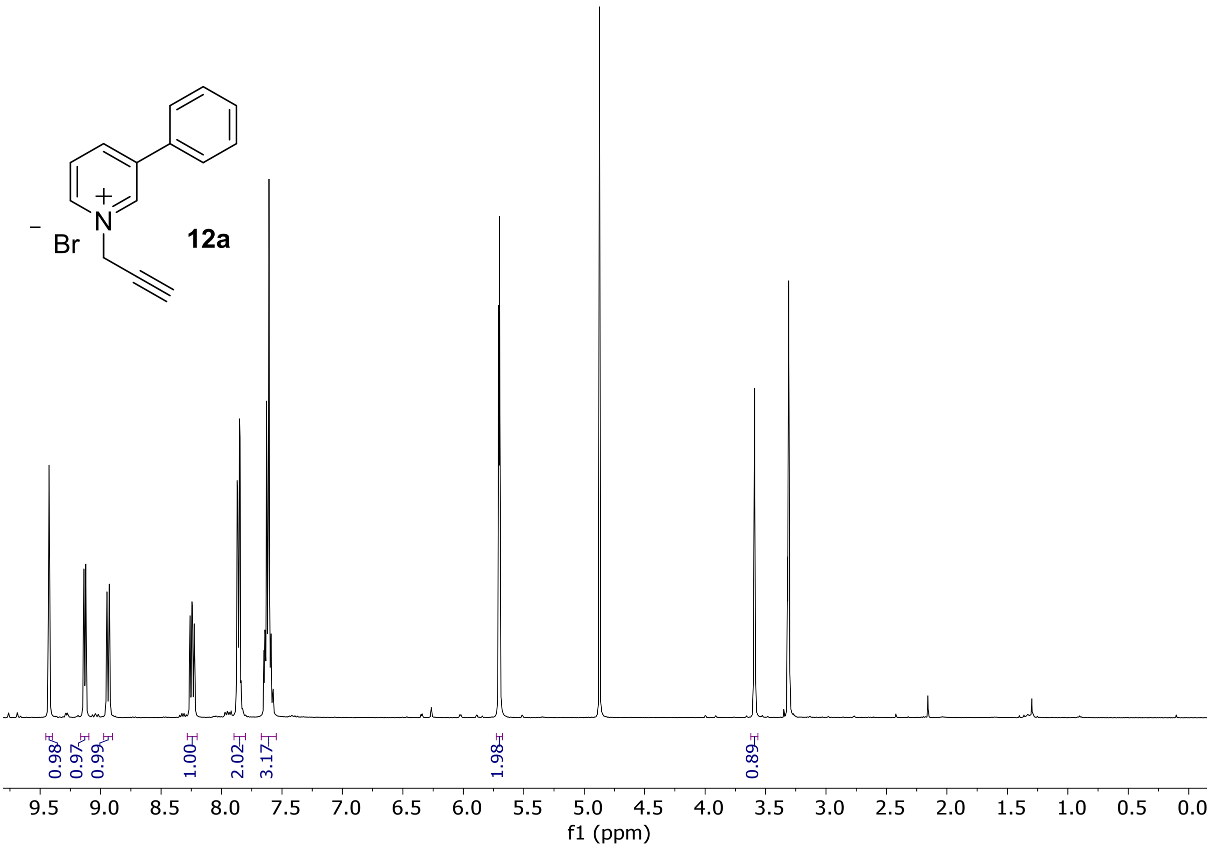

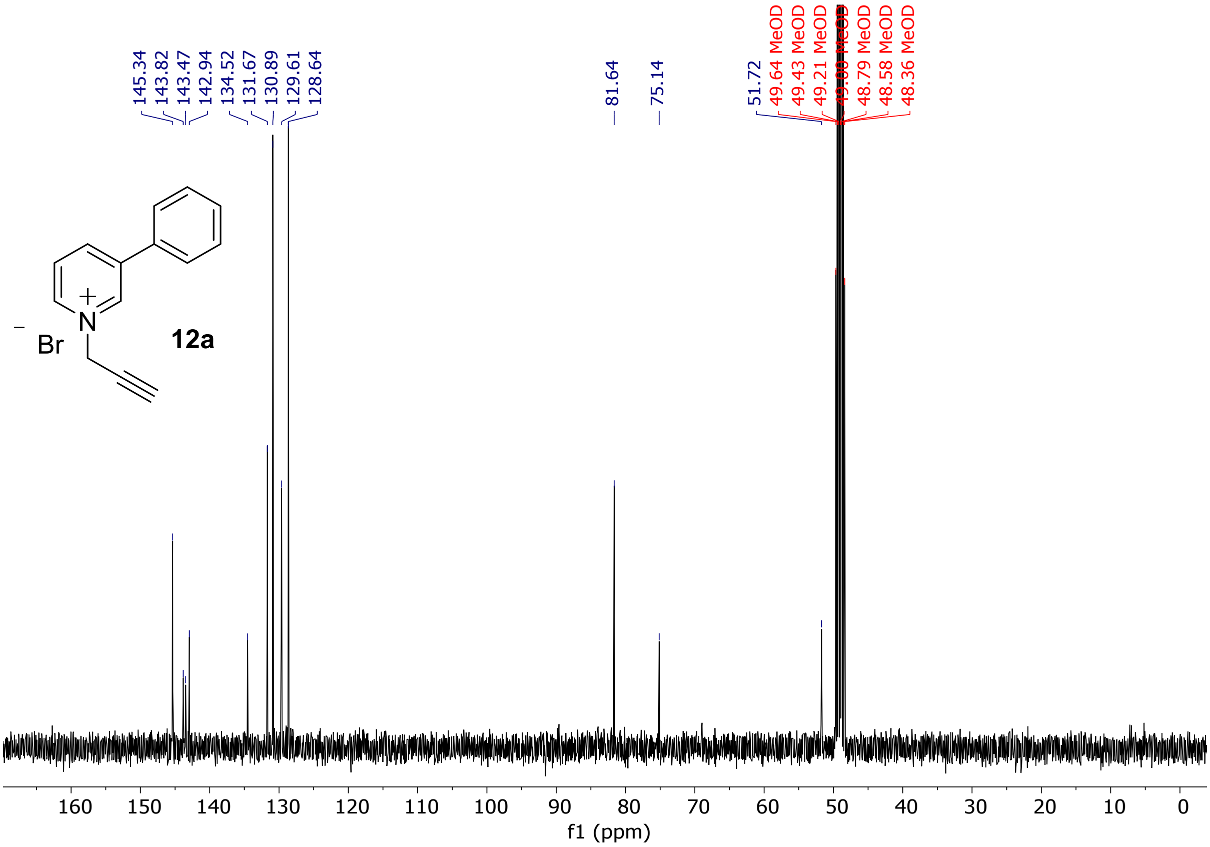

^{1}H & ^{13}C NMR spectra for 1-ethyl-3-methylpyridinium iodide (13a)

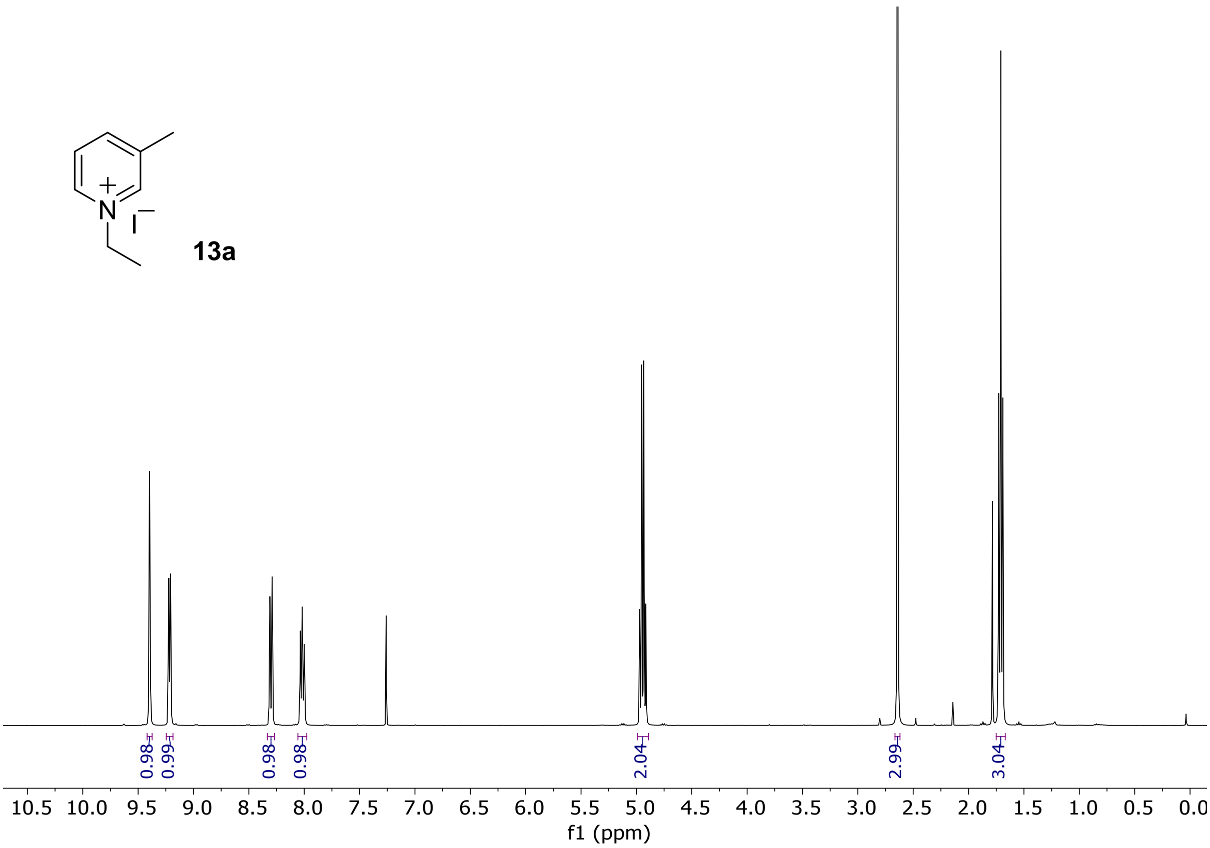

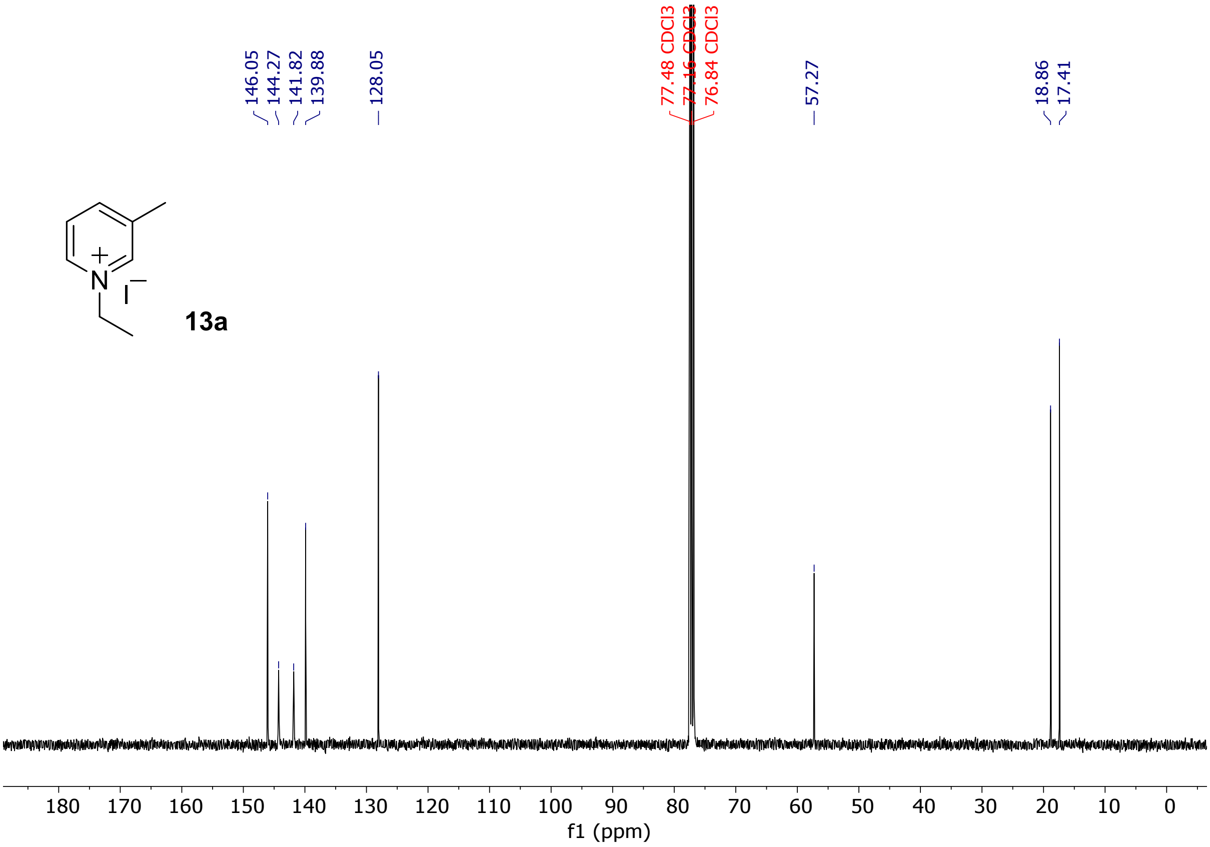

^{1}H & ^{13}C NMR spectra for 1-benzyl-3-methylpyridinium bromide (14a)

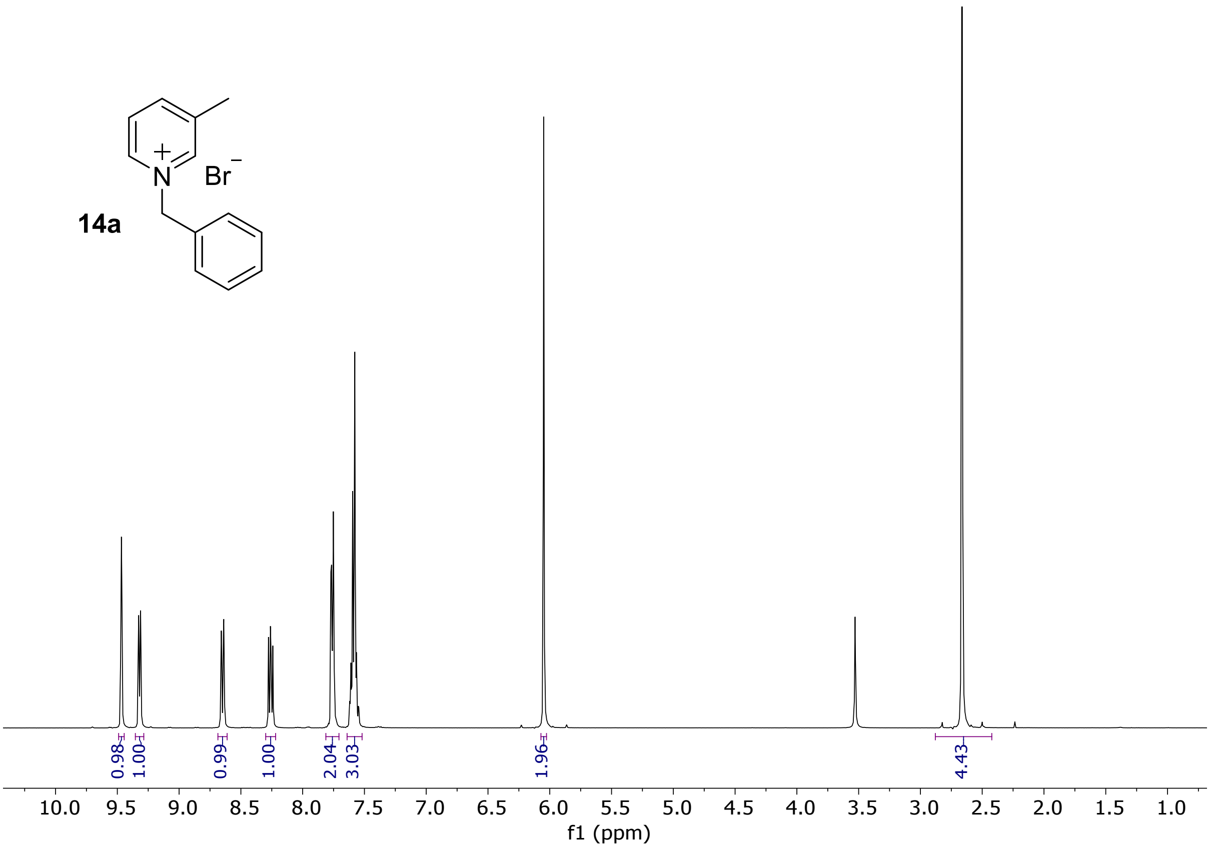

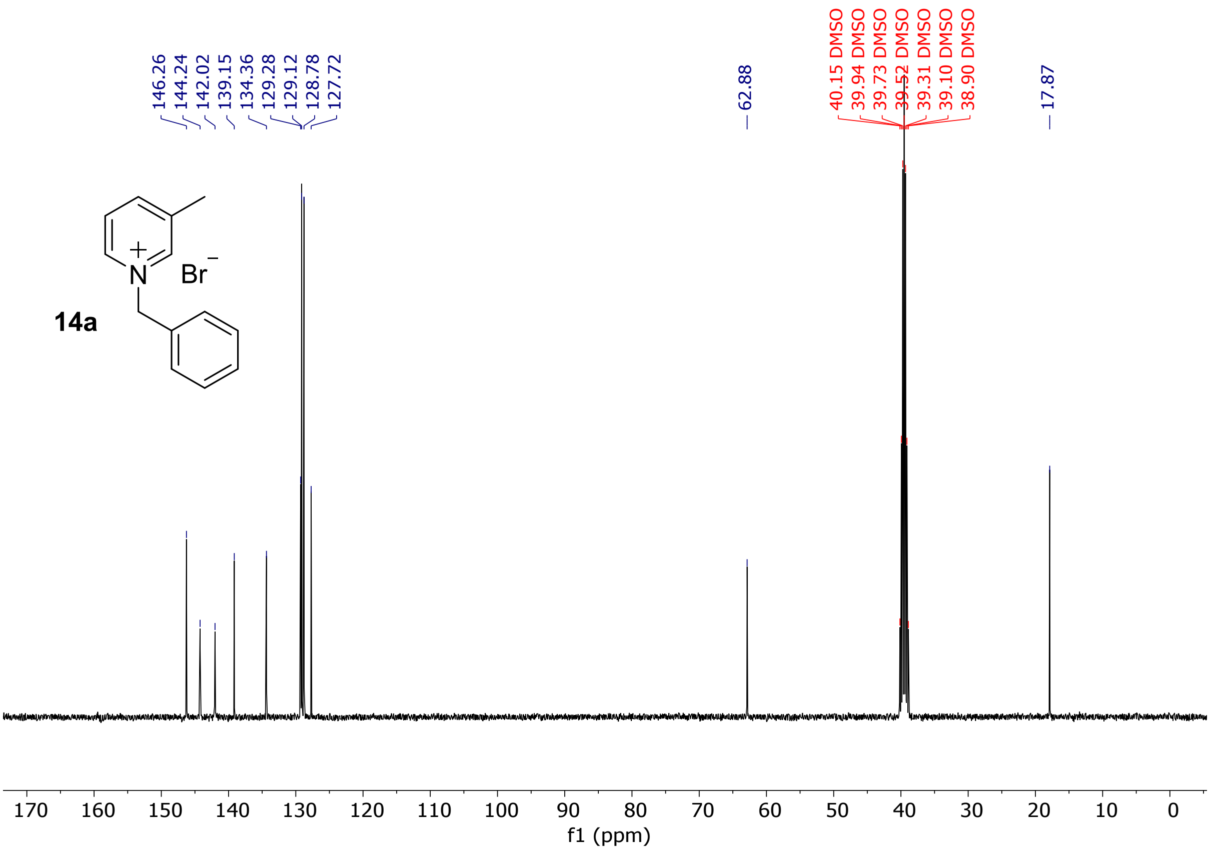

^{1}H & ^{13}C NMR spectra for 1-ethyl-4-methylpyridinium iodide (15a)

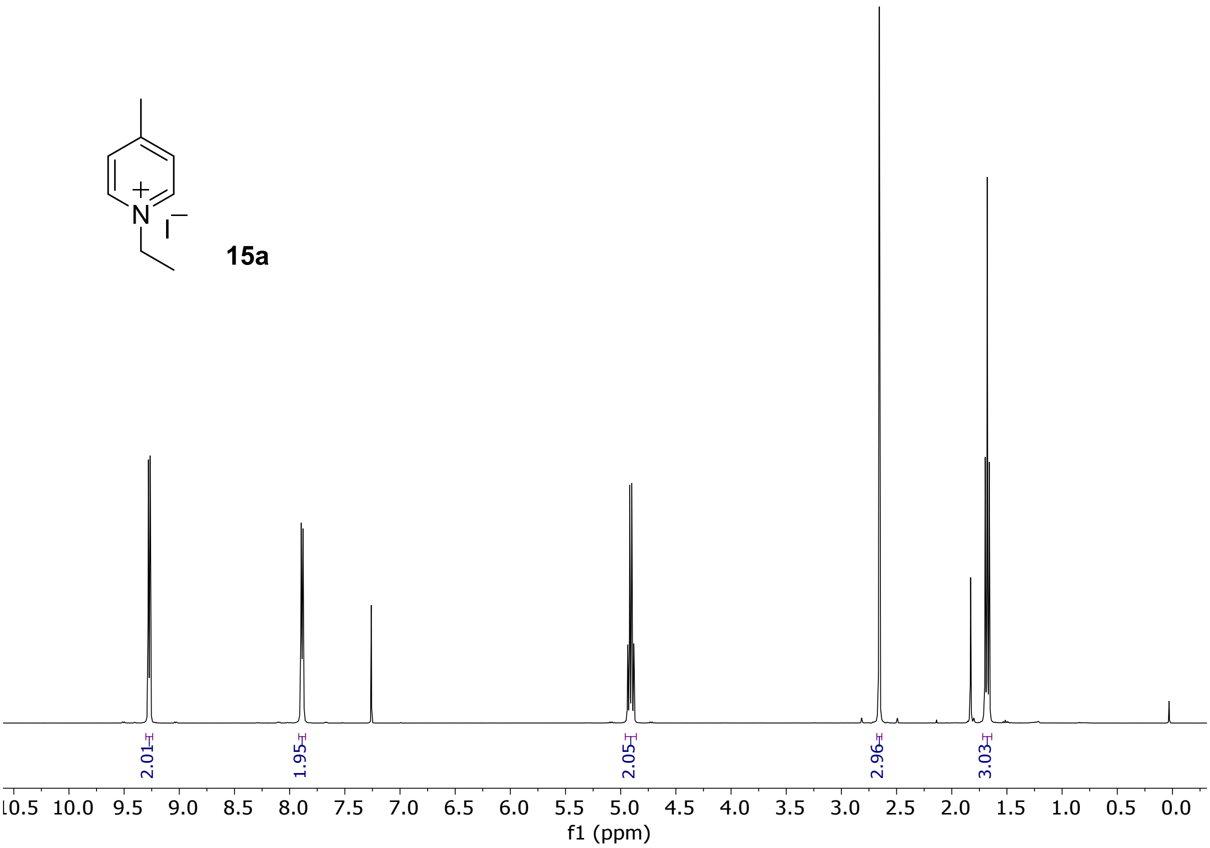

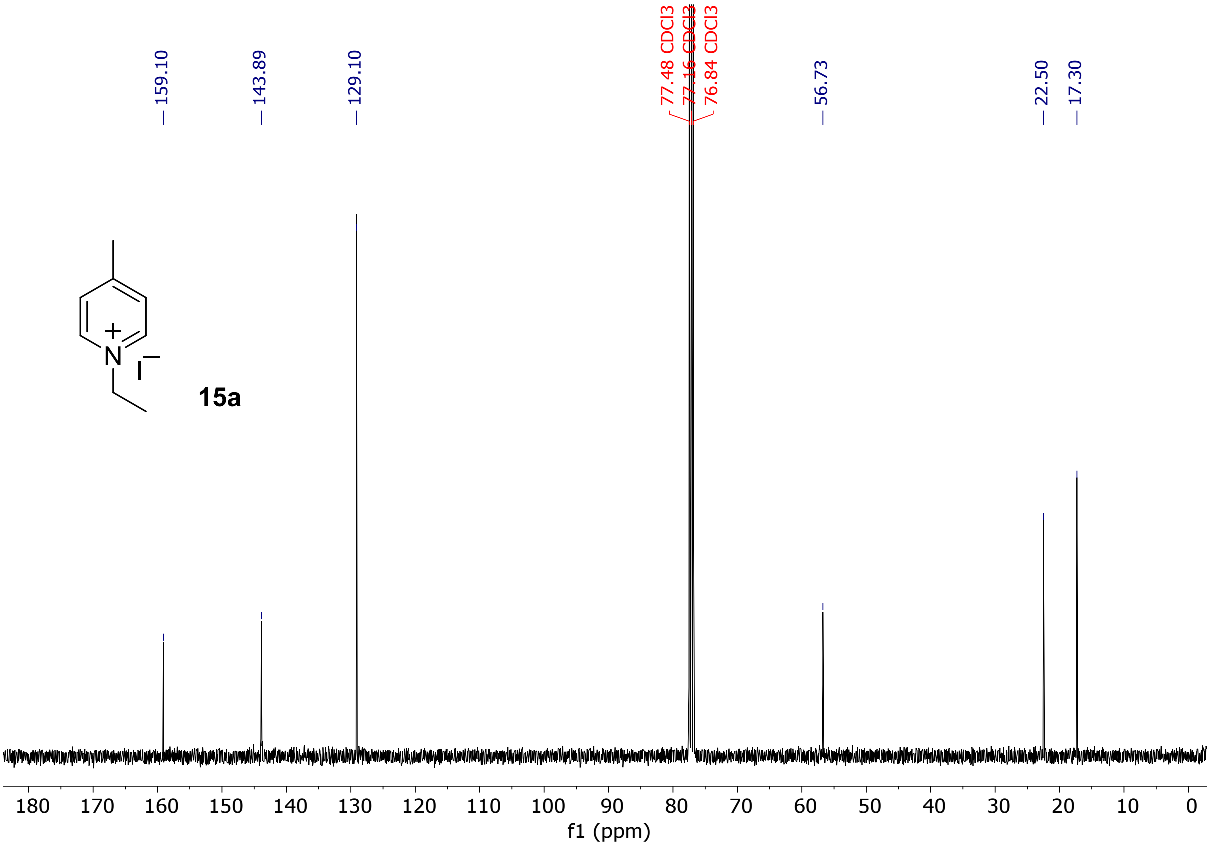

^{1}H & ^{13}C NMR spectra for 1-ethyl-4-phenylpyridinium iodide (16a)

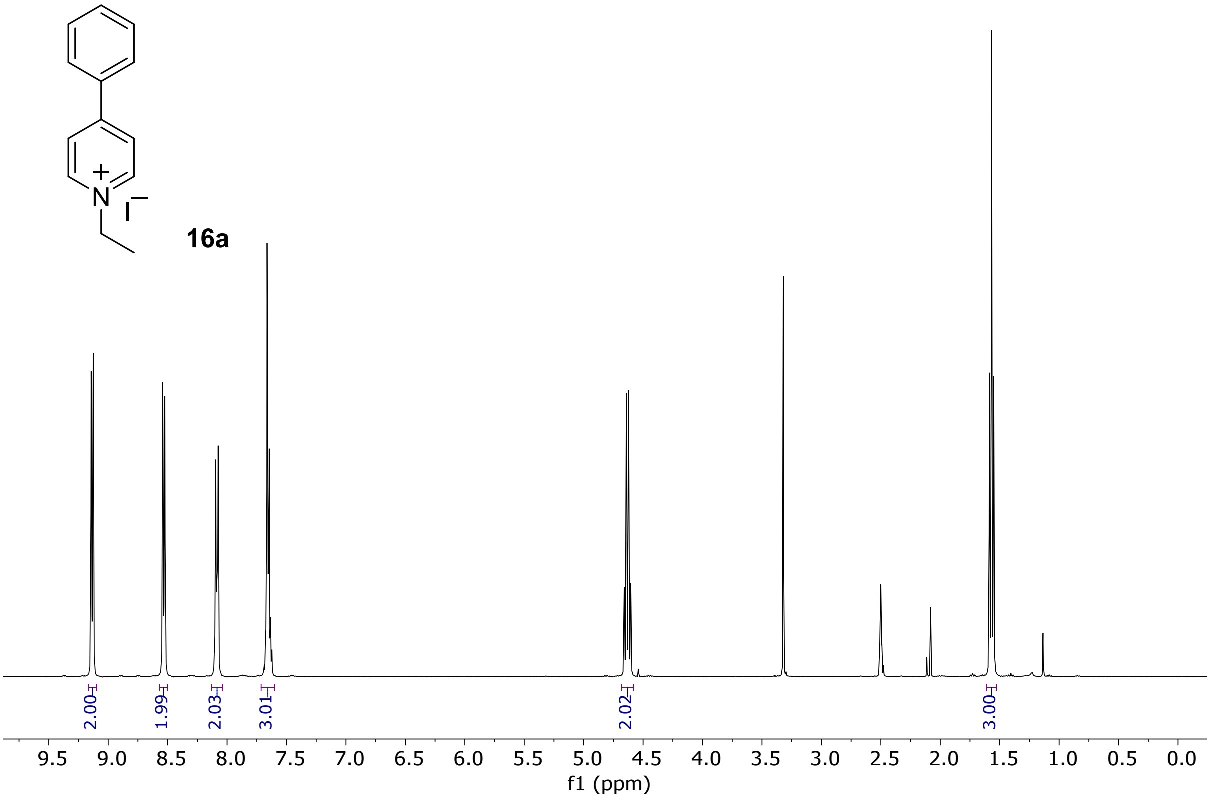

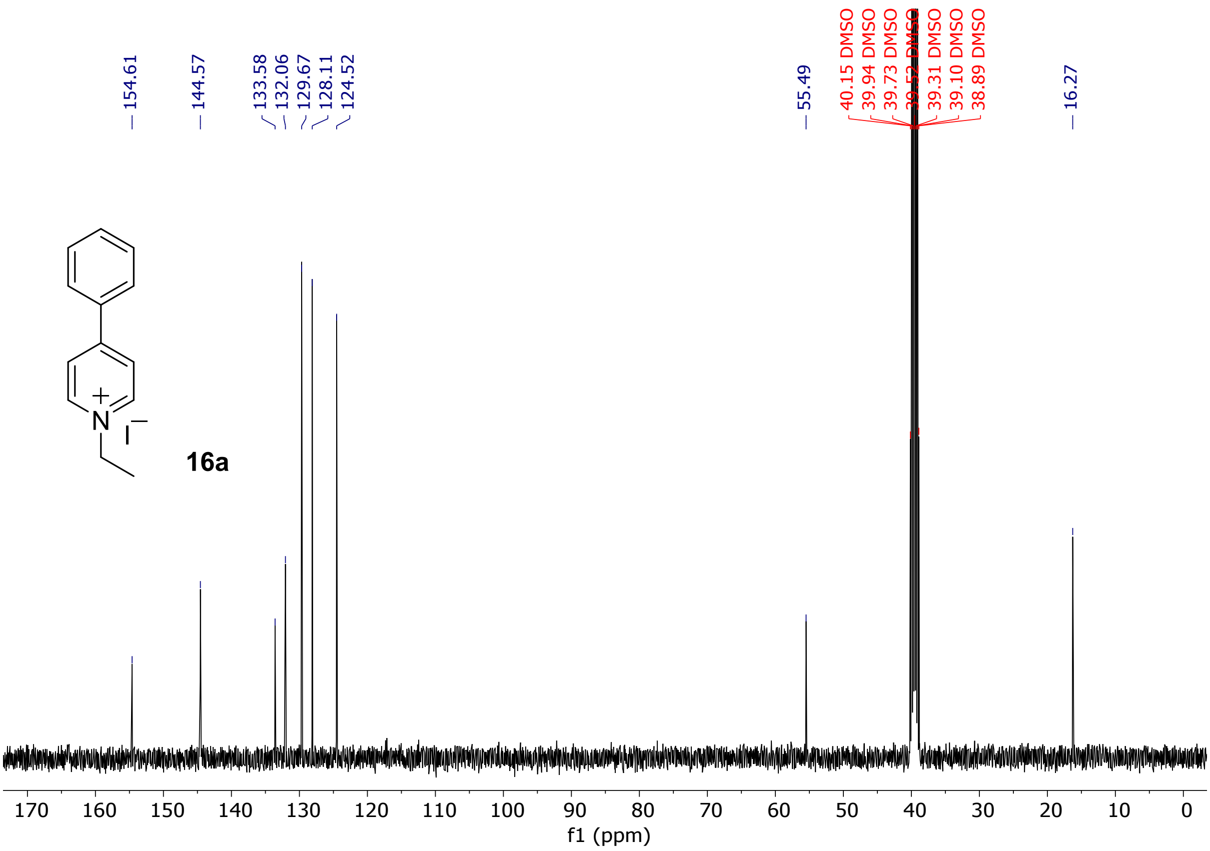

^{1}H & ^{13}C NMR spectra for 1-ethyl-3,4-dimethylpyridinium iodide (17a)

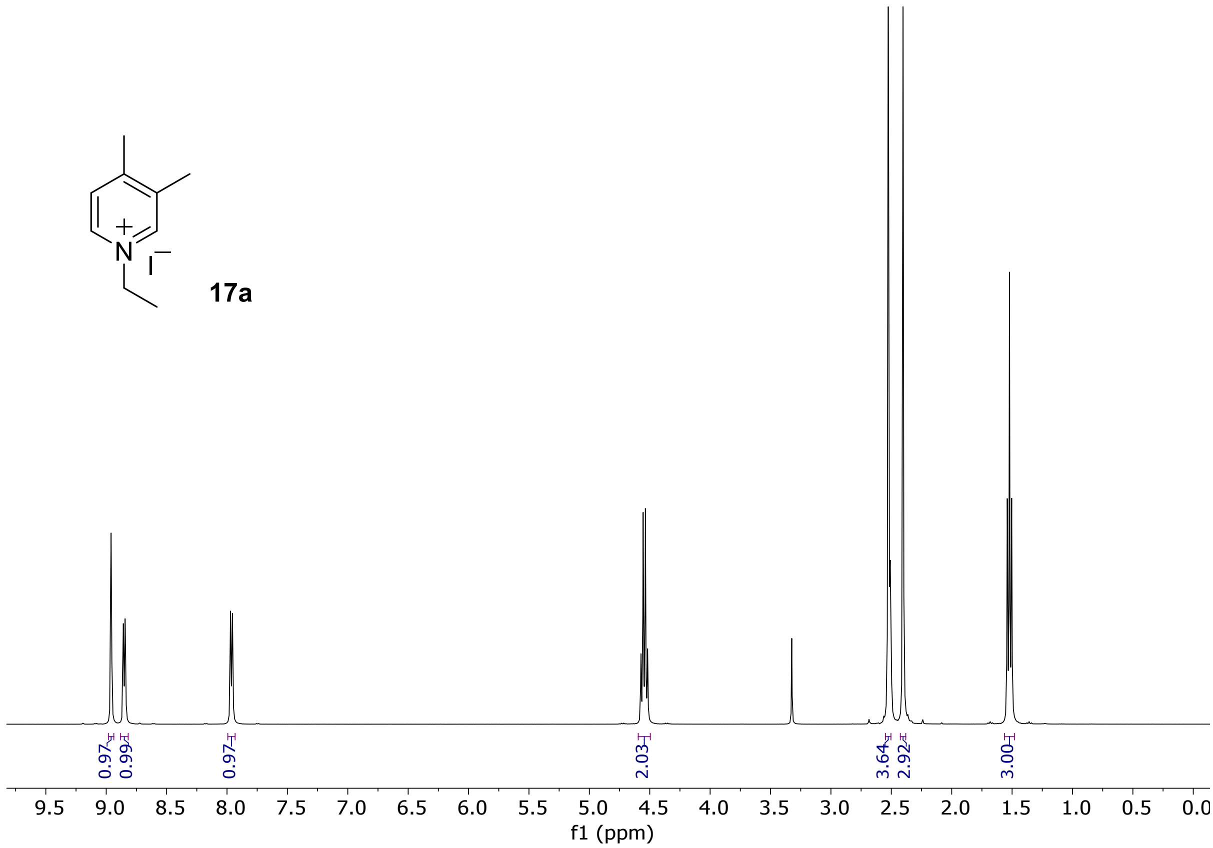

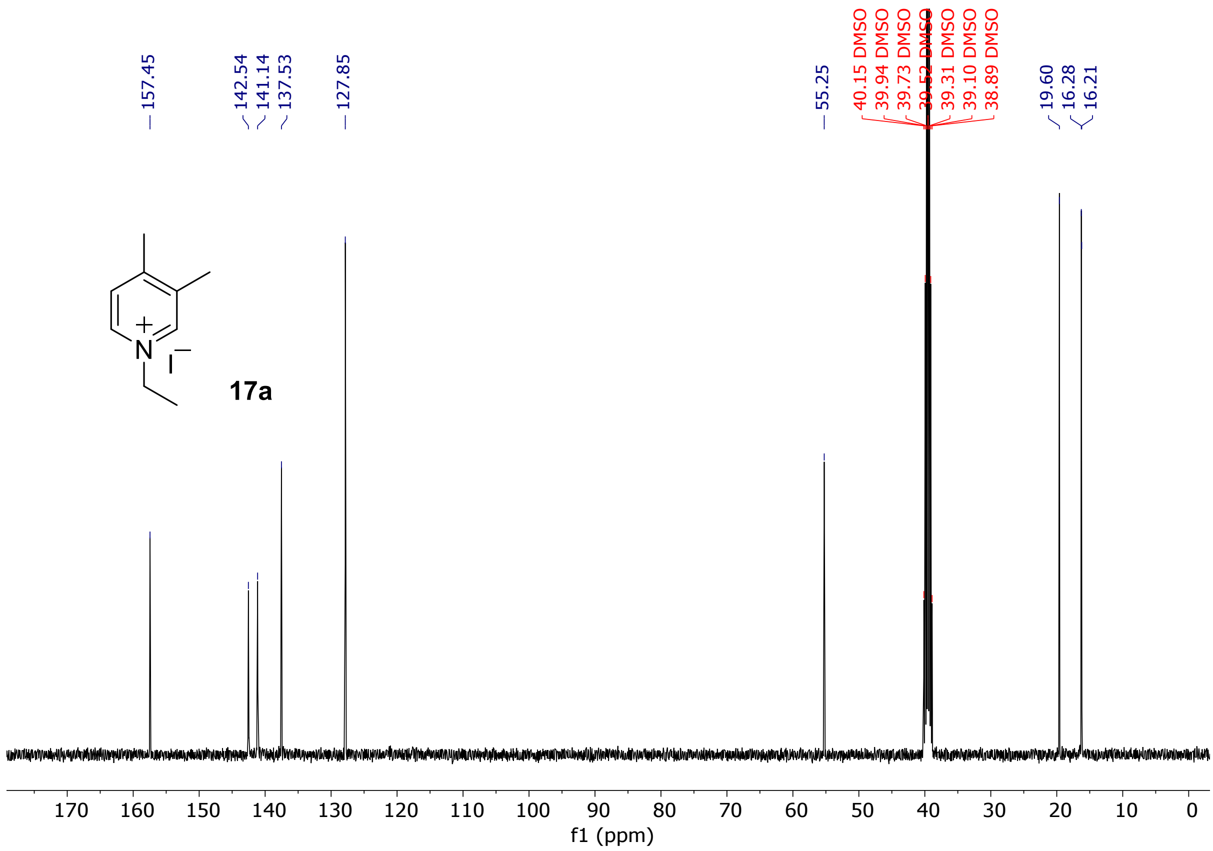

^{1}H & ^{13}C NMR spectra for 2-ethyl-5,6,7,8-tetrahydroisoquinolinium iodide (18a)

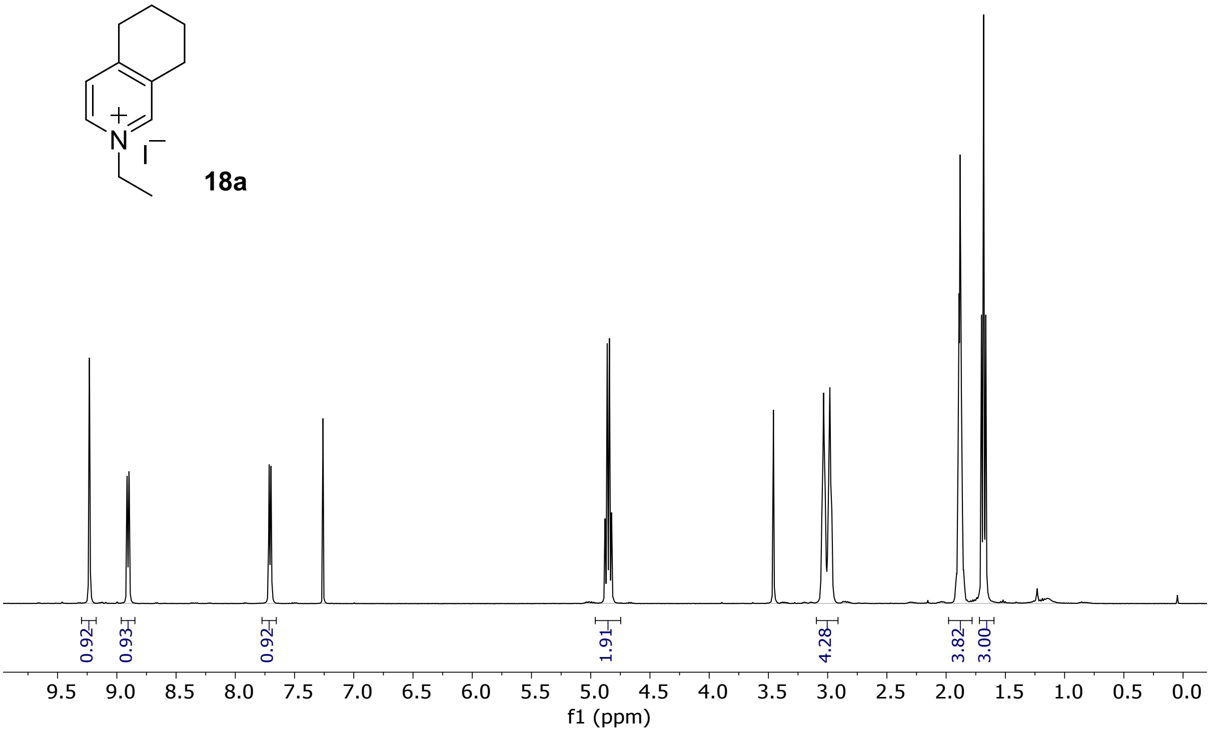

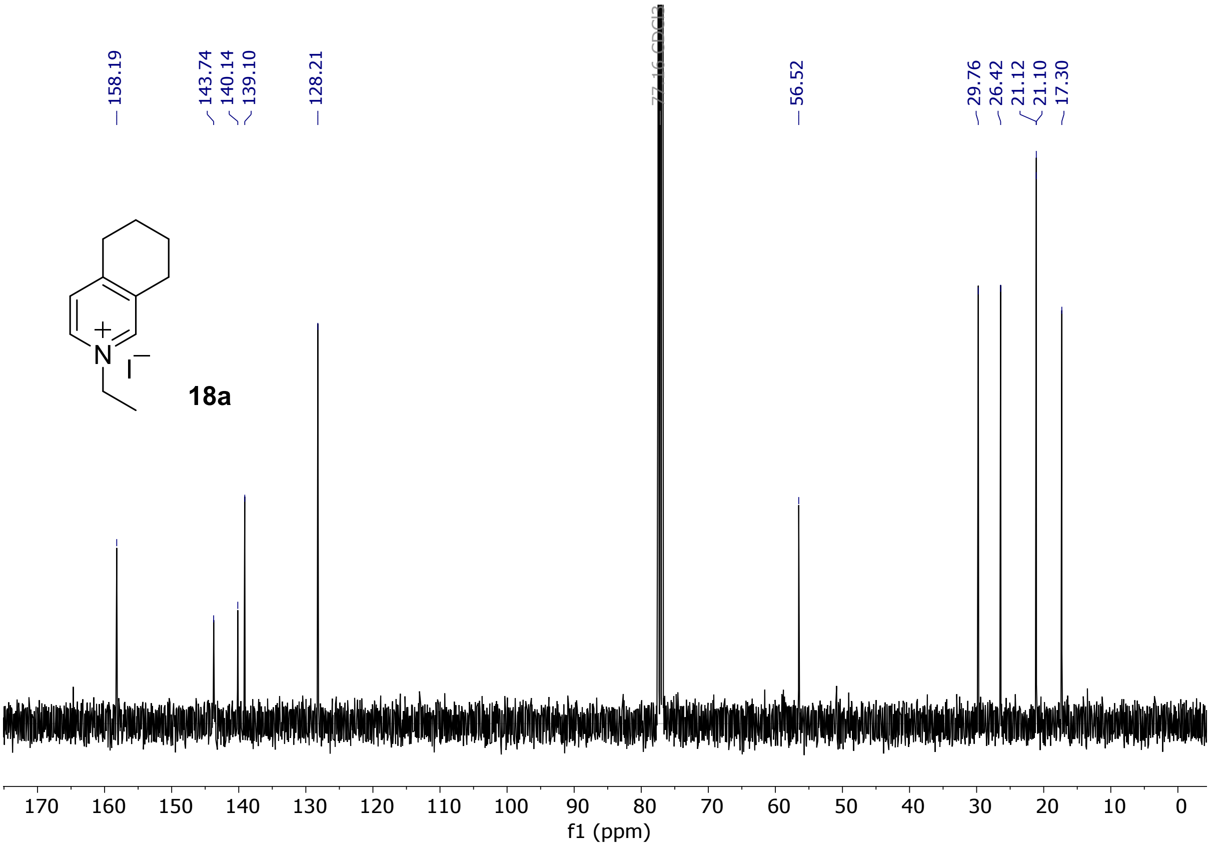

^{1}H & ^{13}C NMR spectra for 1-ethyl-4-methyl-3-phenylpyridinium iodide (19a)

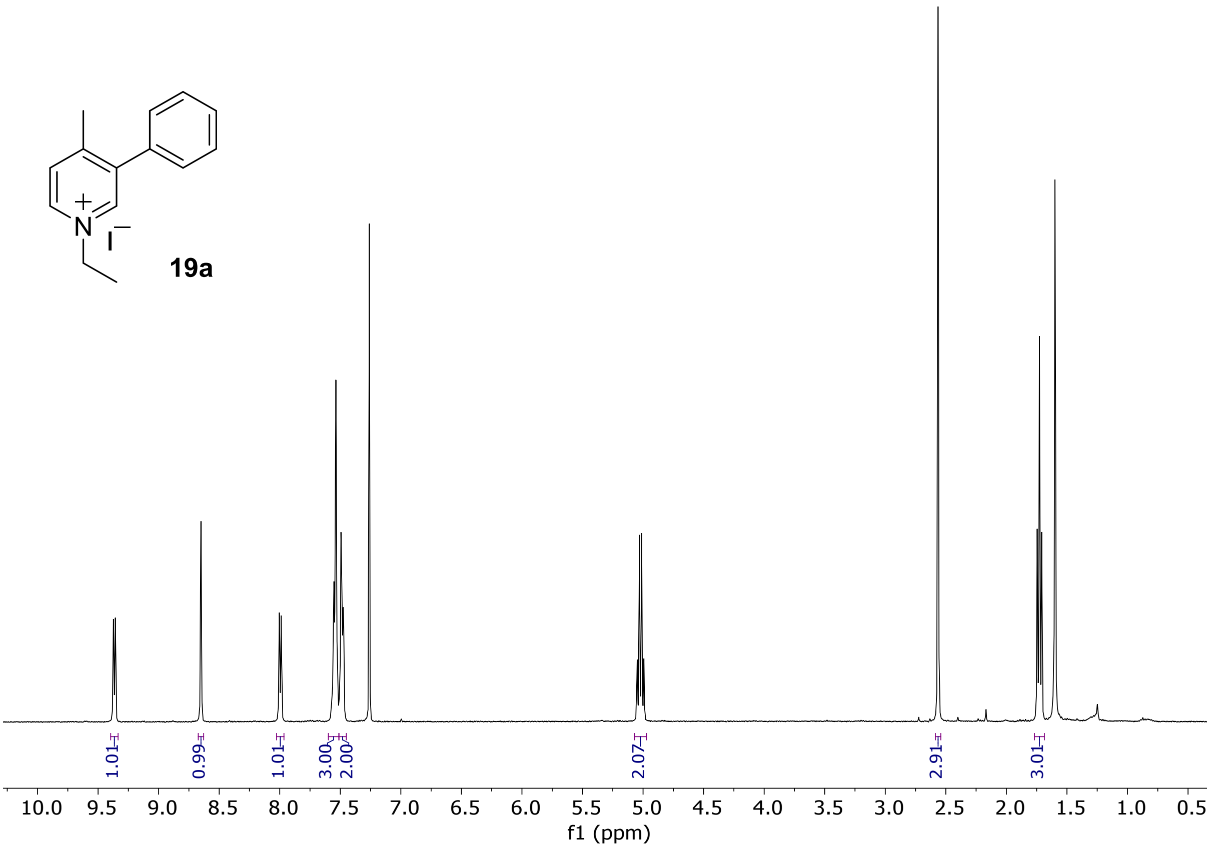

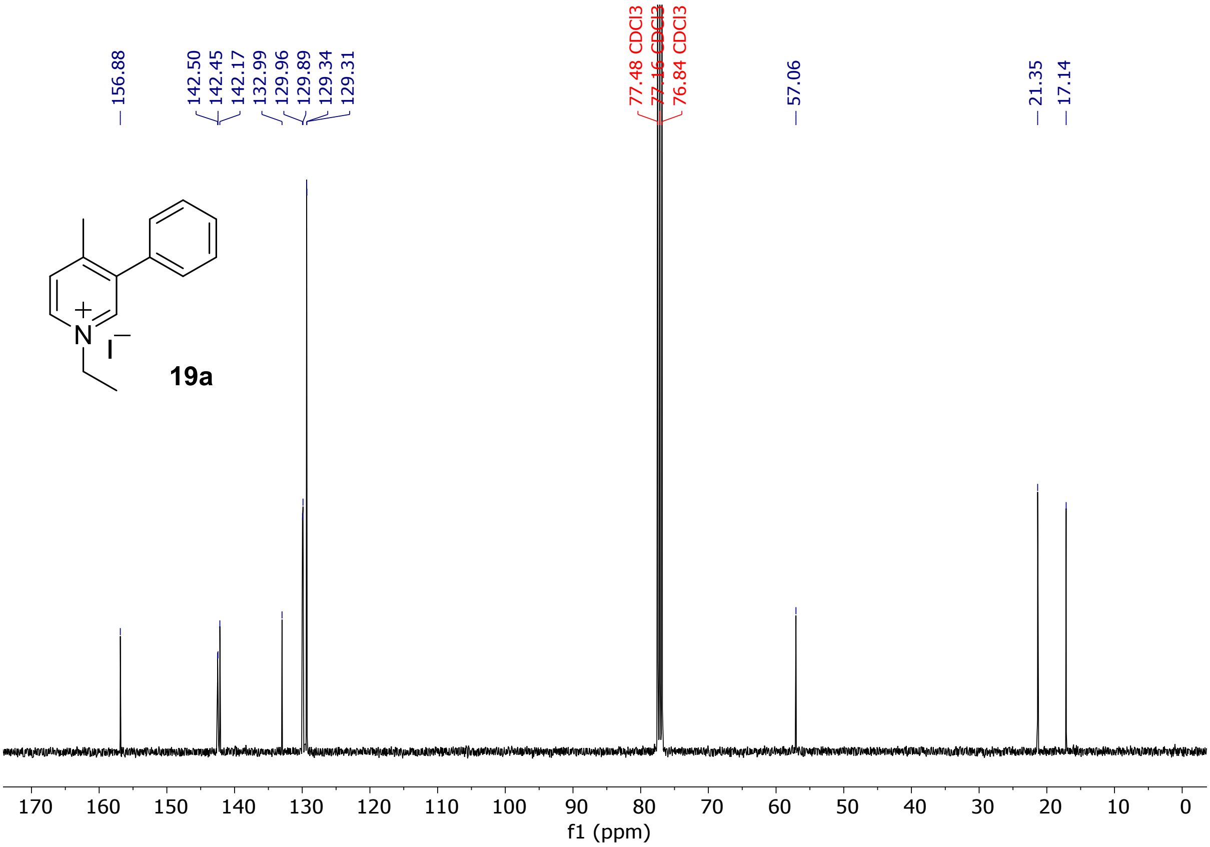

^1H & ^{13}C NMR spectra for 1-allyl-4-methyl-3-phenylpyridinium bromide (20a)

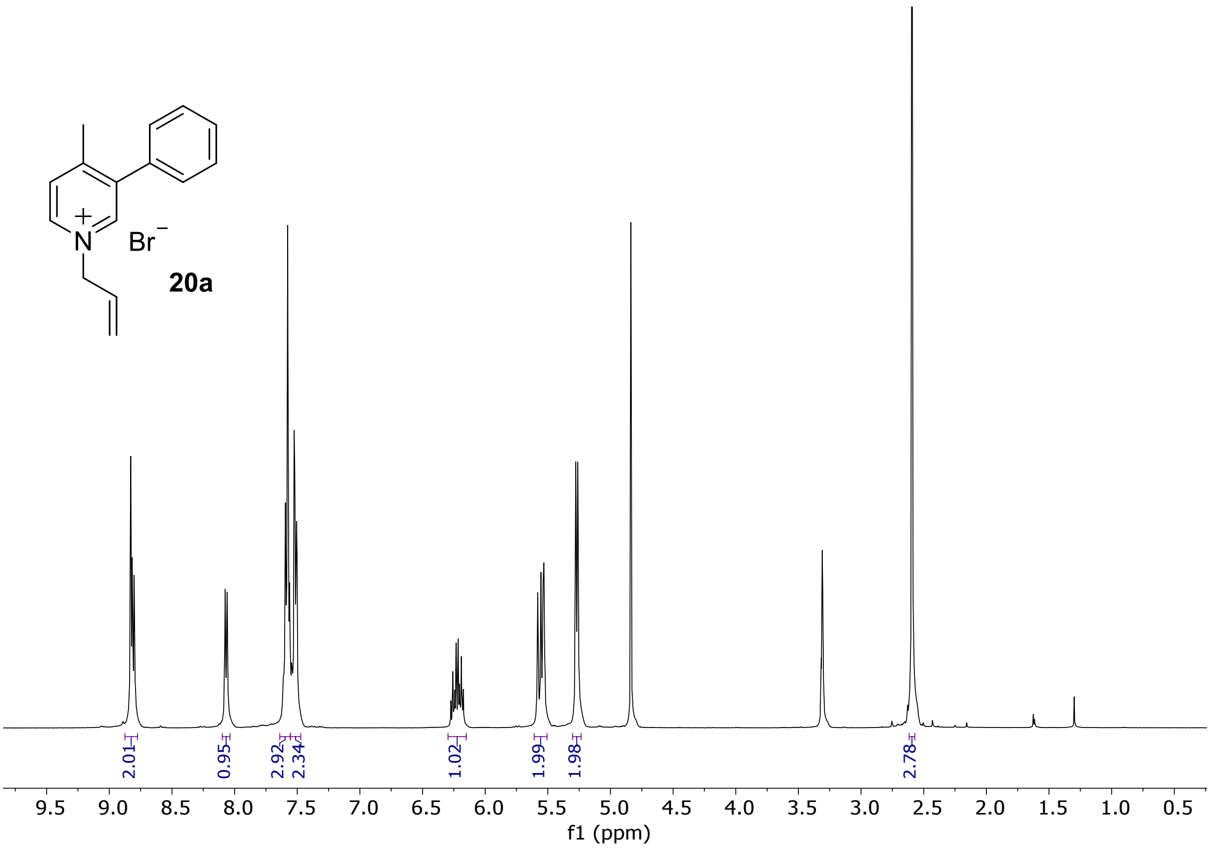

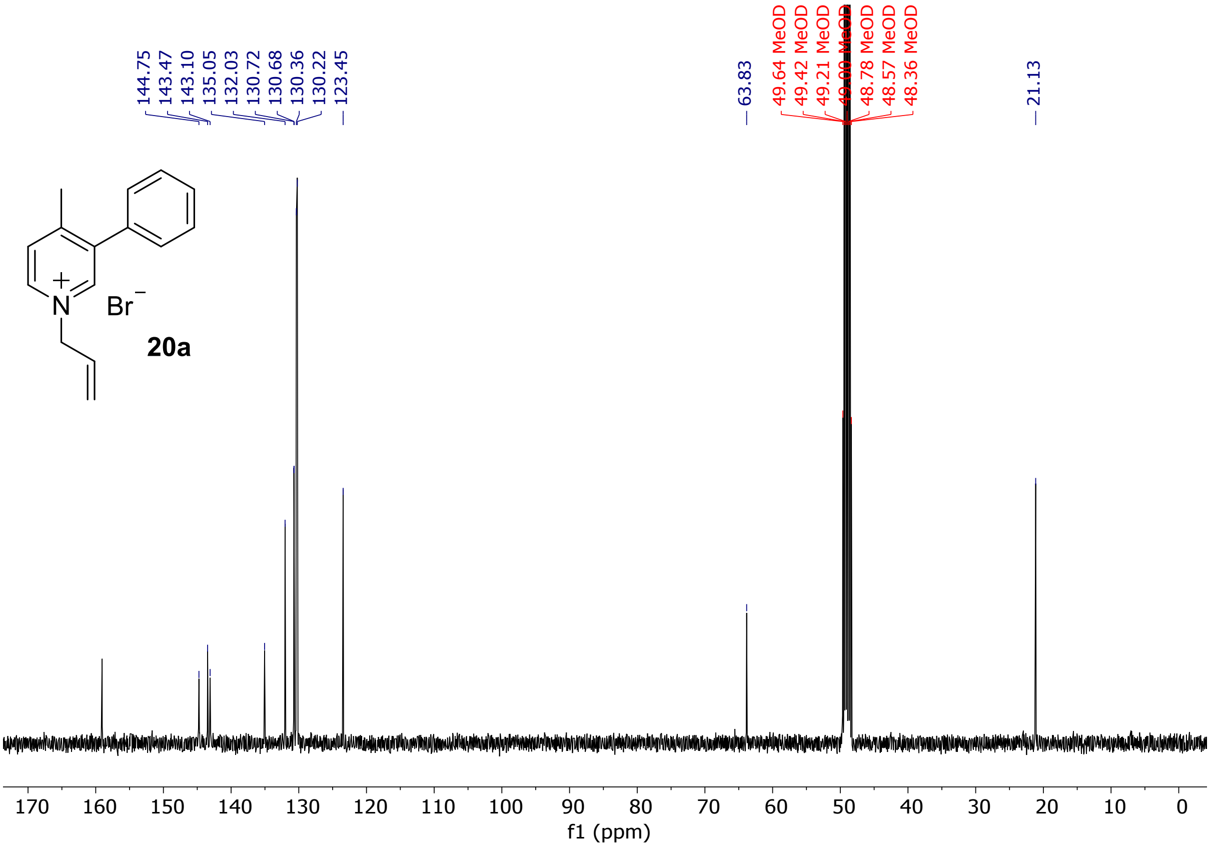

^{1}H & ^{13}C NMR spectra for 1-allyl-3-methyl-4-phenylpyridinium bromide (Crude-21a)

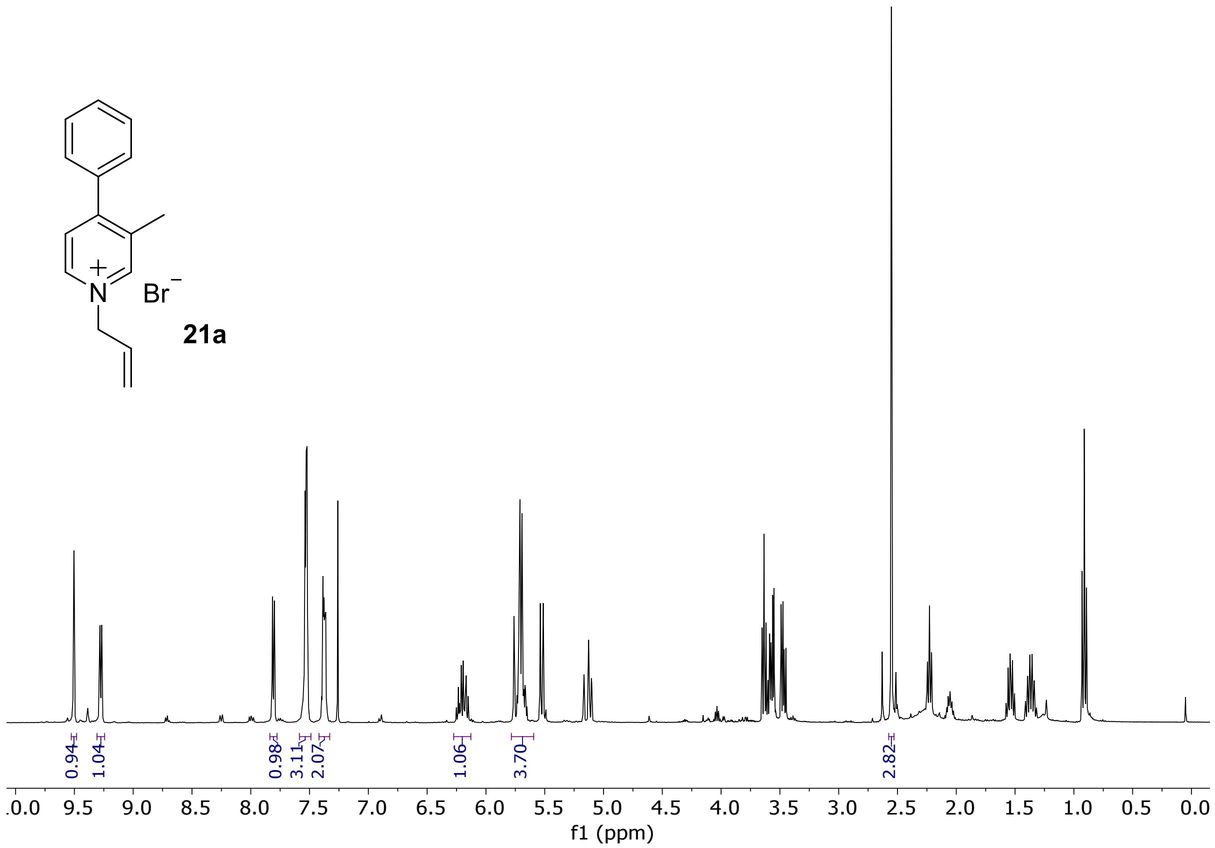
Br−
21a
0.94
1.04
0.98
3.11
2.07
1.06
3.70
2.82
.0.0 9.5 9.0 8.5 8.0 7.5 7.0 6.5 6.0 5.5 5.0 4.5 4.0 3.5 3.0 2.5 2.0 1.5 1.0 0.5 0.0
f1 (ppm)

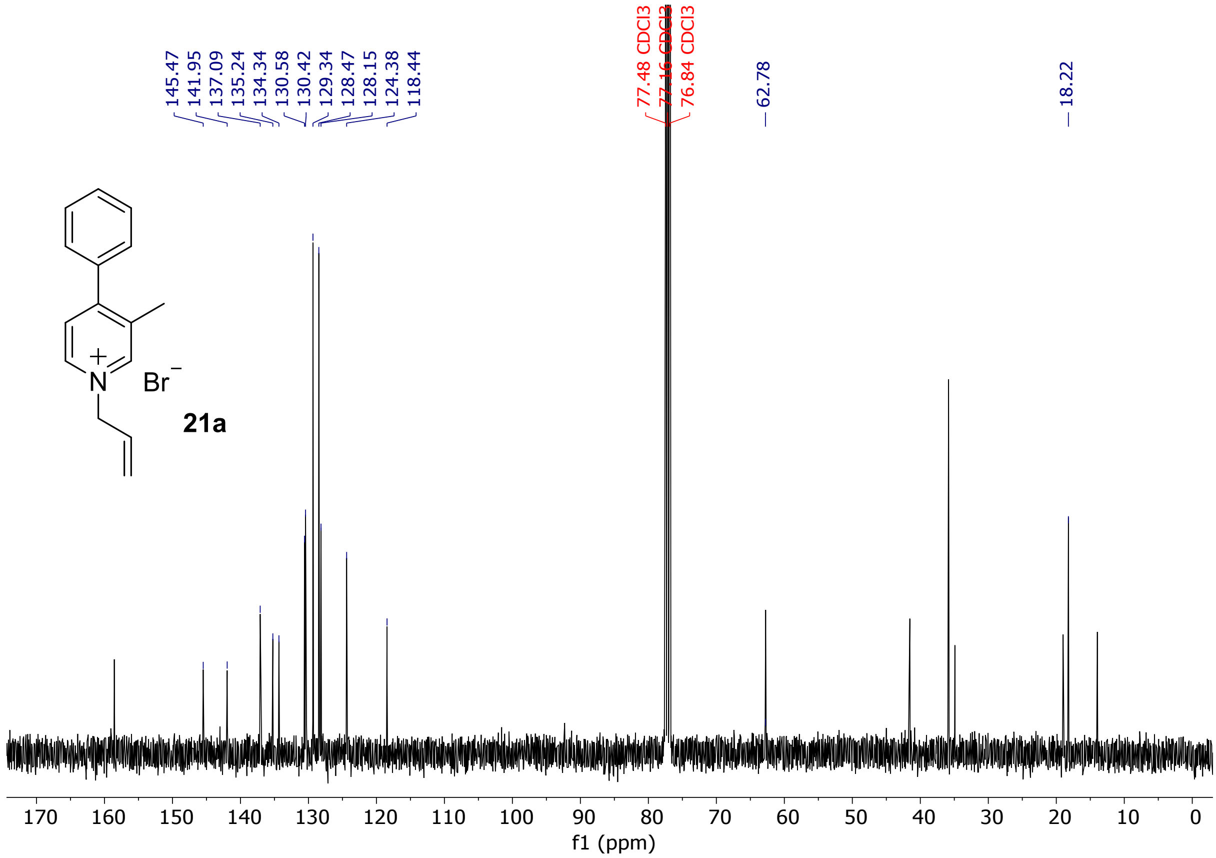
145.47
141.95
137.09
135.24
134.34
130.58
130.42
129.34
128.47
128.15
124.38
118.44
77.48 CDCl3
77.16 CDCl3
76.84 CDCl3
62.78
18.22
Br−
21a
170 160 150 140 130 120 110 100 90 80 70 60 50 40 30 20 10 0
f1 (ppm)

^{1}H & ^{13}C NMR spectra for 5-(3-methoxyphenyl)-1-propyl-1,2,3,6-THP (1b)

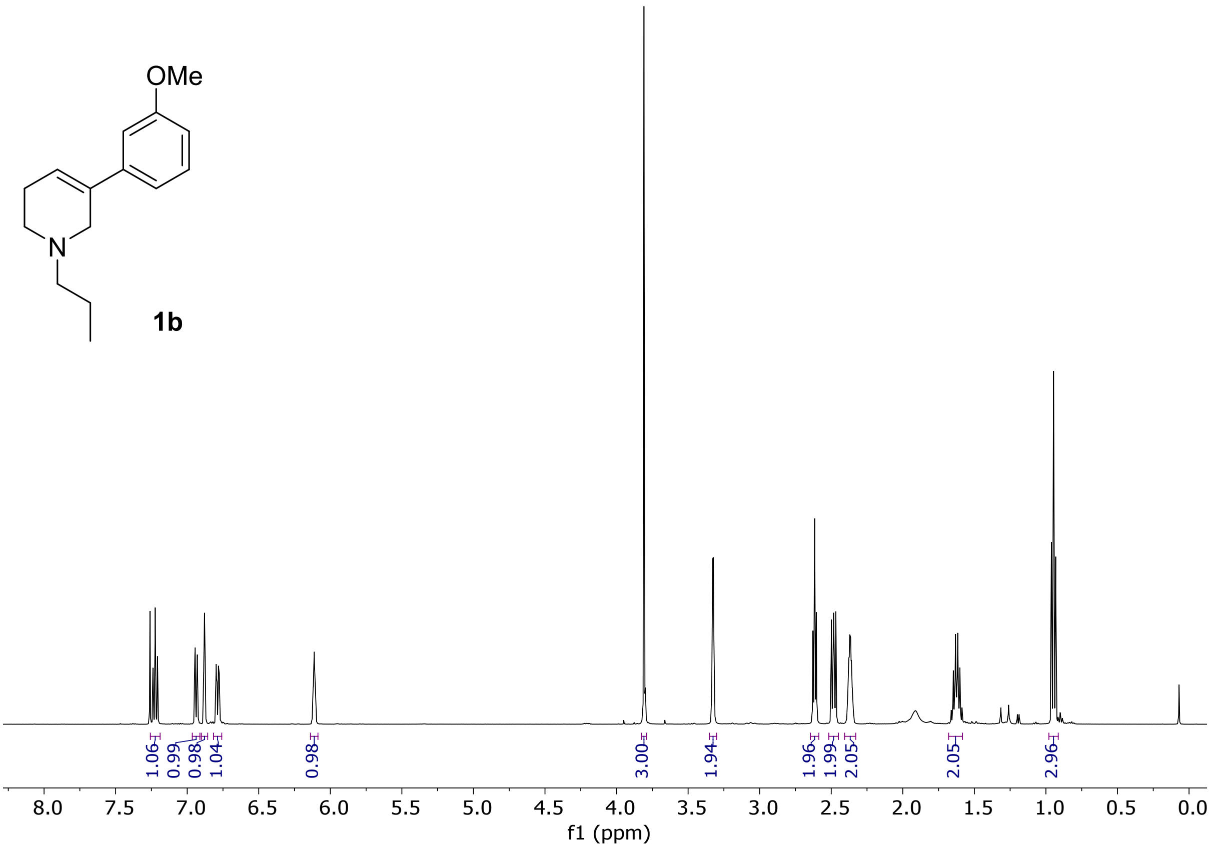

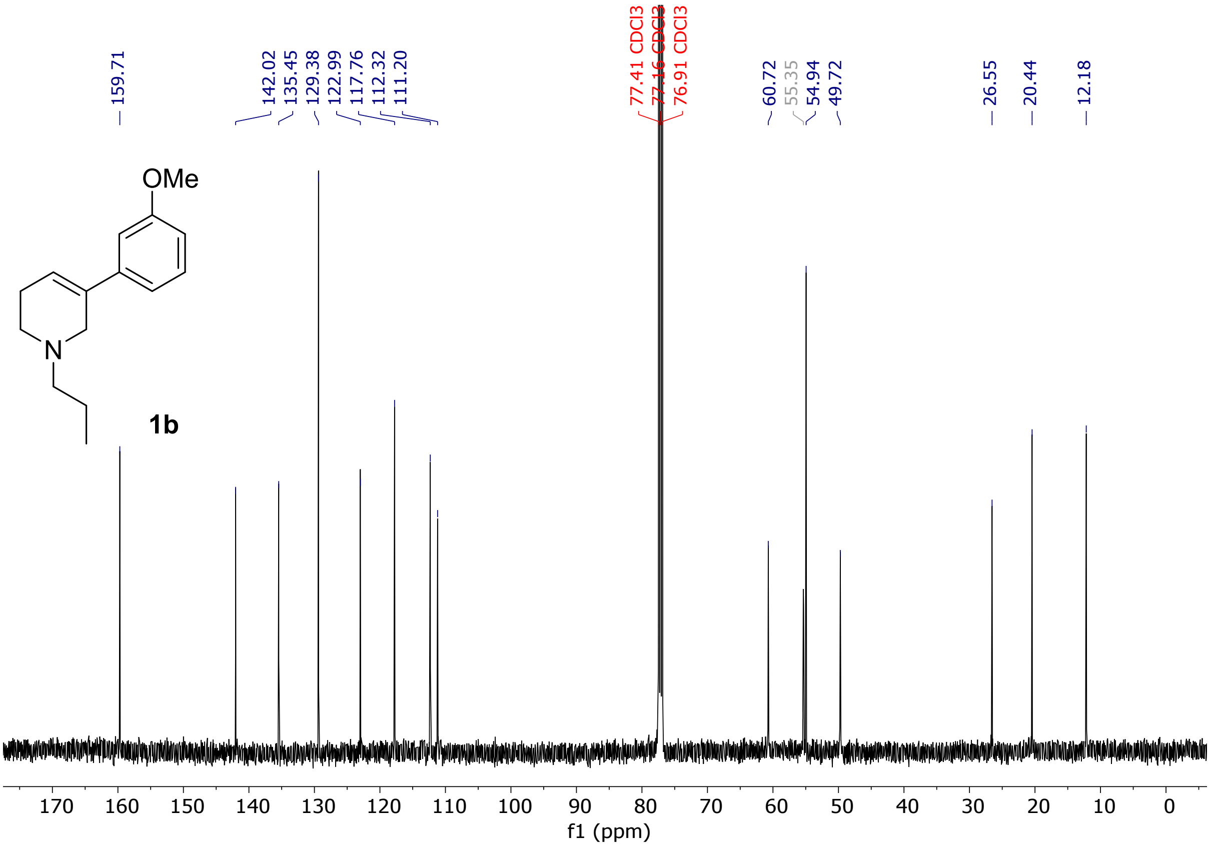

^{1}H & ^{13}C NMR spectra for 3-(1-propyl-1,2,5,6-tetrahydropyridin-3-yl)phenol

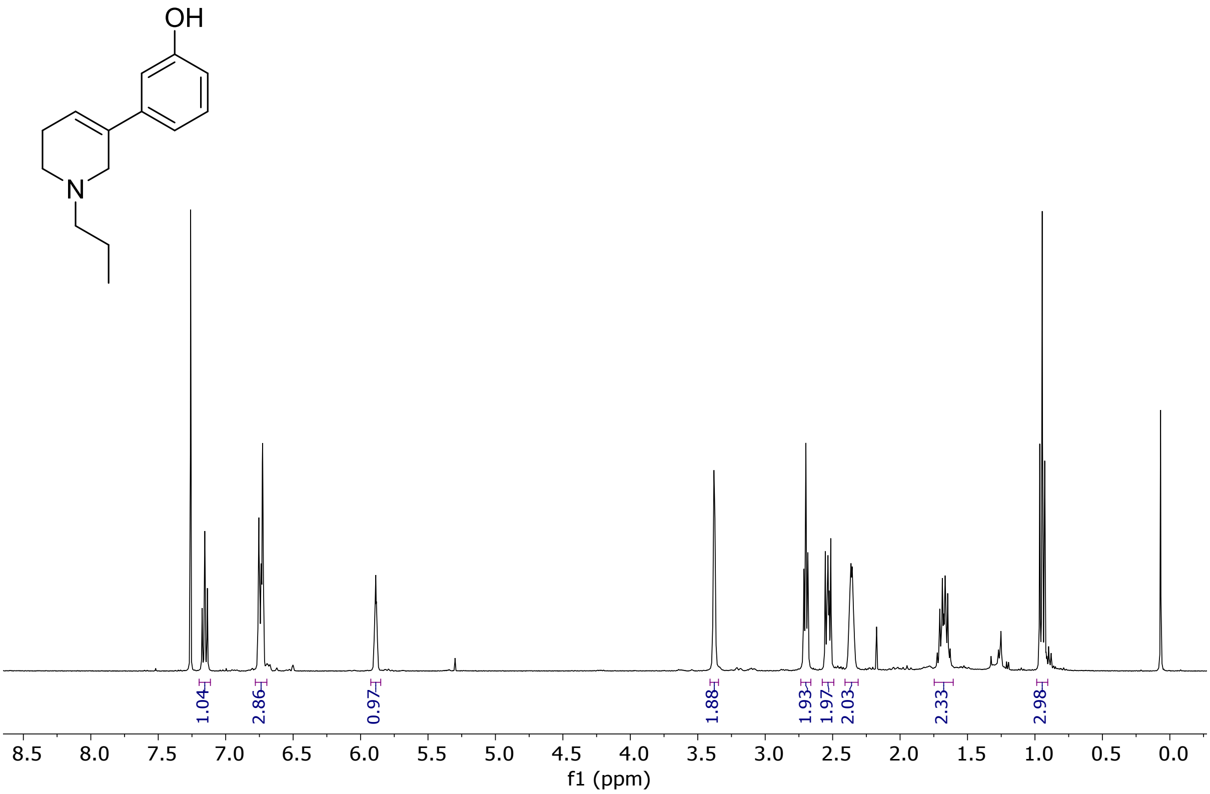

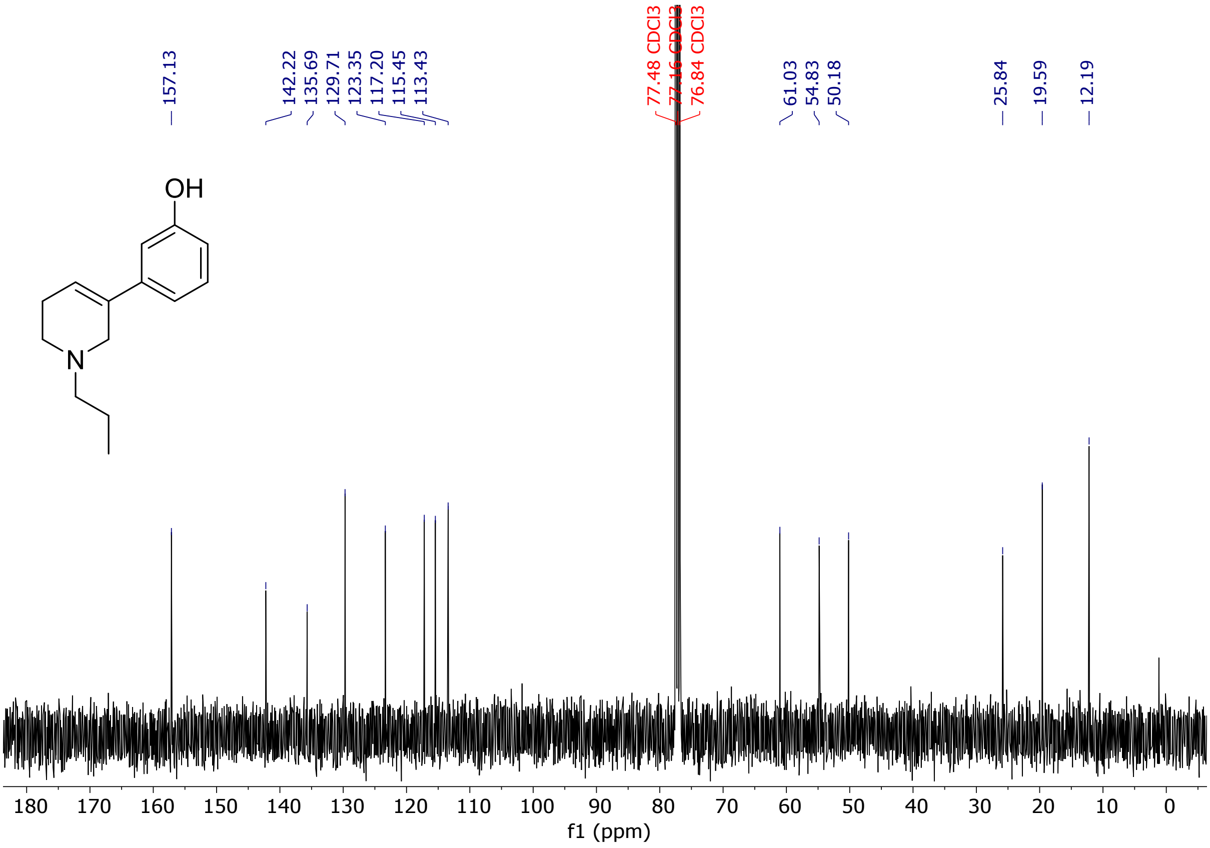

^1H & ^{13}C NMR spectra for 5-(3-(methylsulfonyl)phenyl)-1-propyl-1,2,3,6-THP (2b)

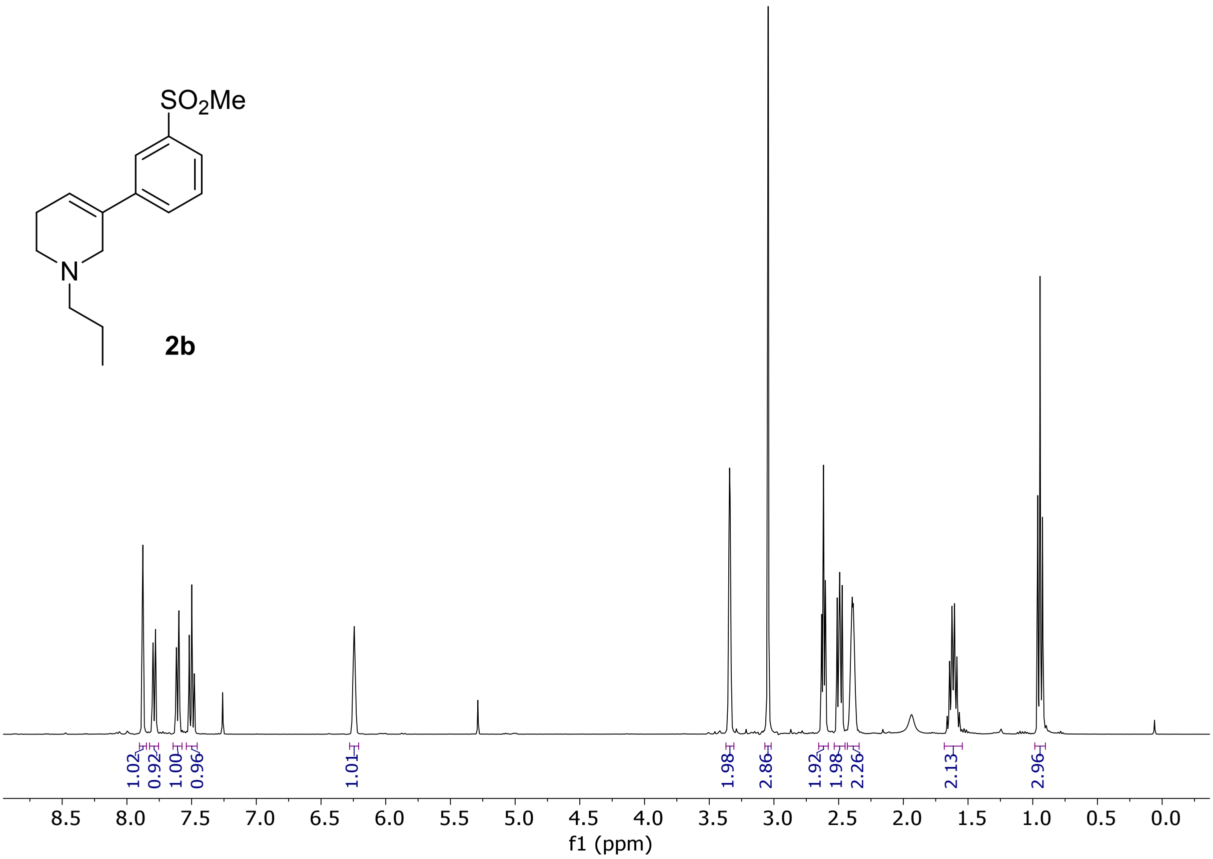

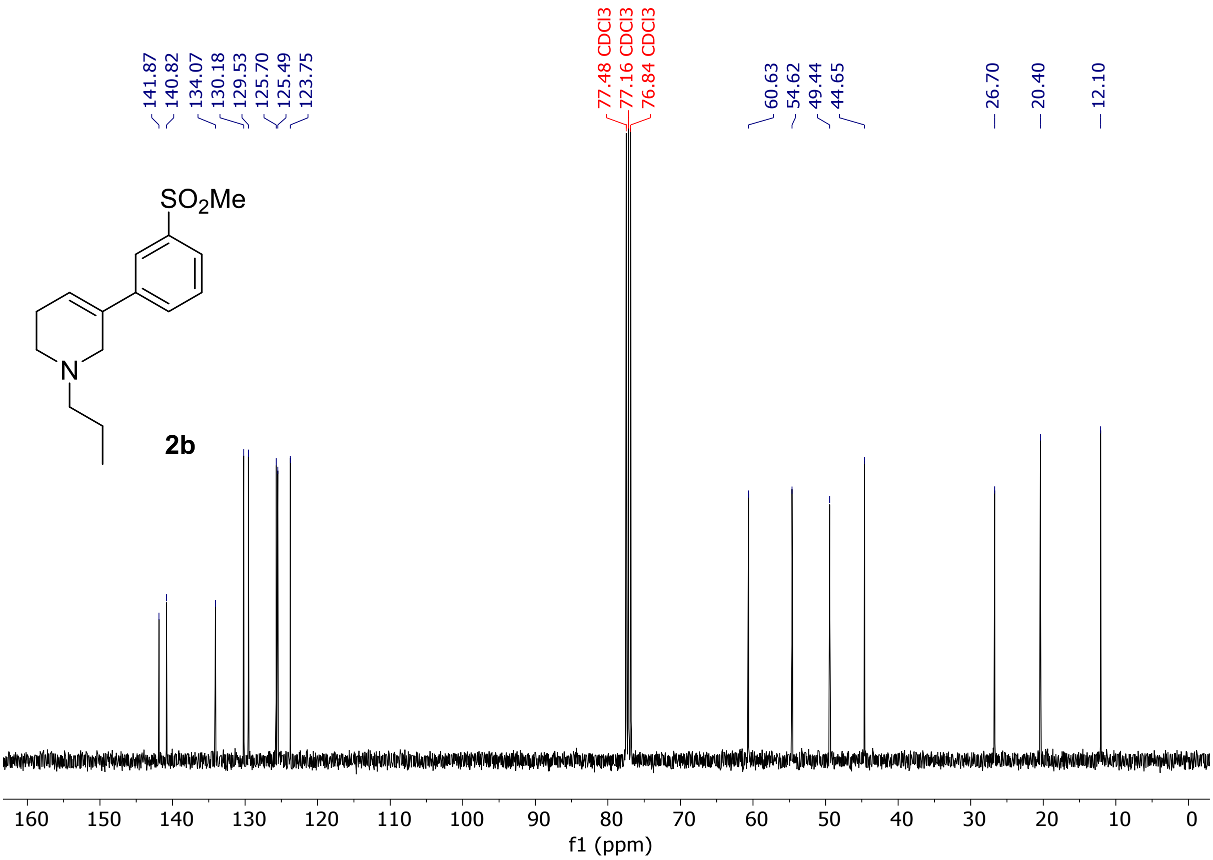

^1H & ^{13}C NMR spectra for 1-allyl-5-(4-bromophenyl)-1,2,3,6-THP (3b)

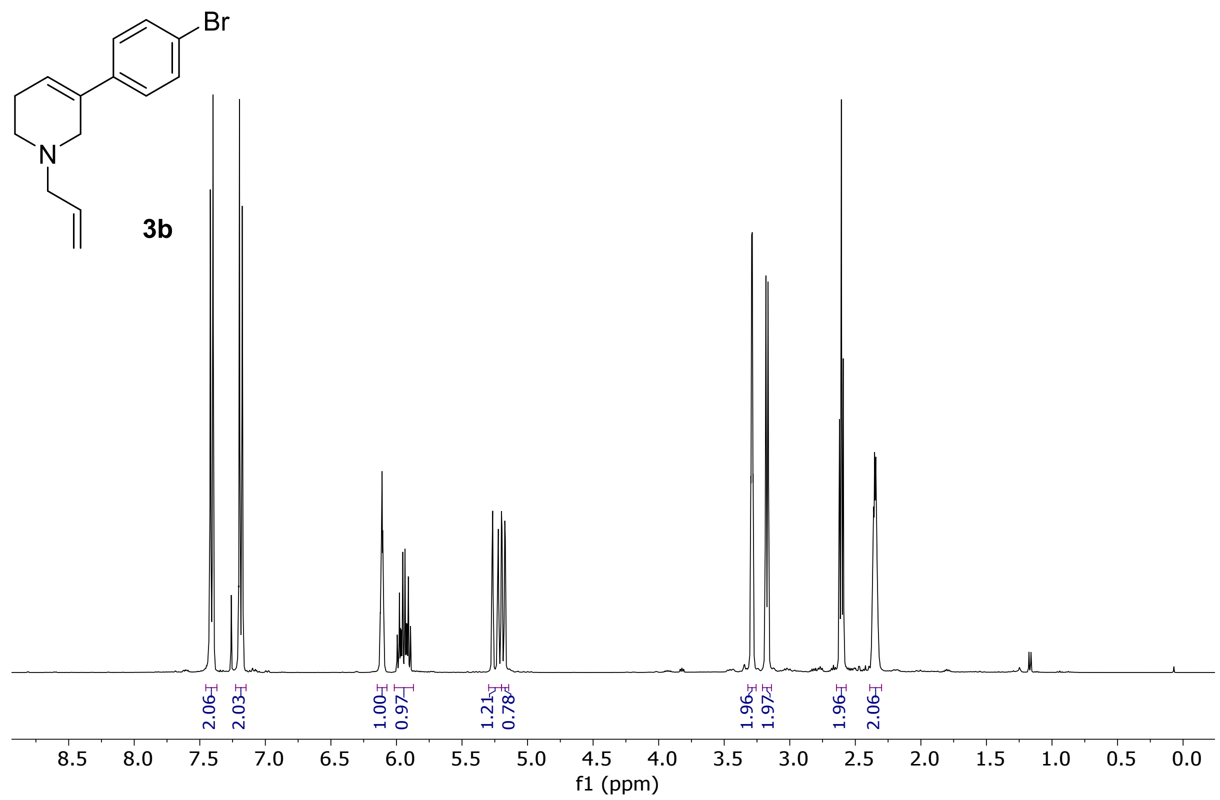

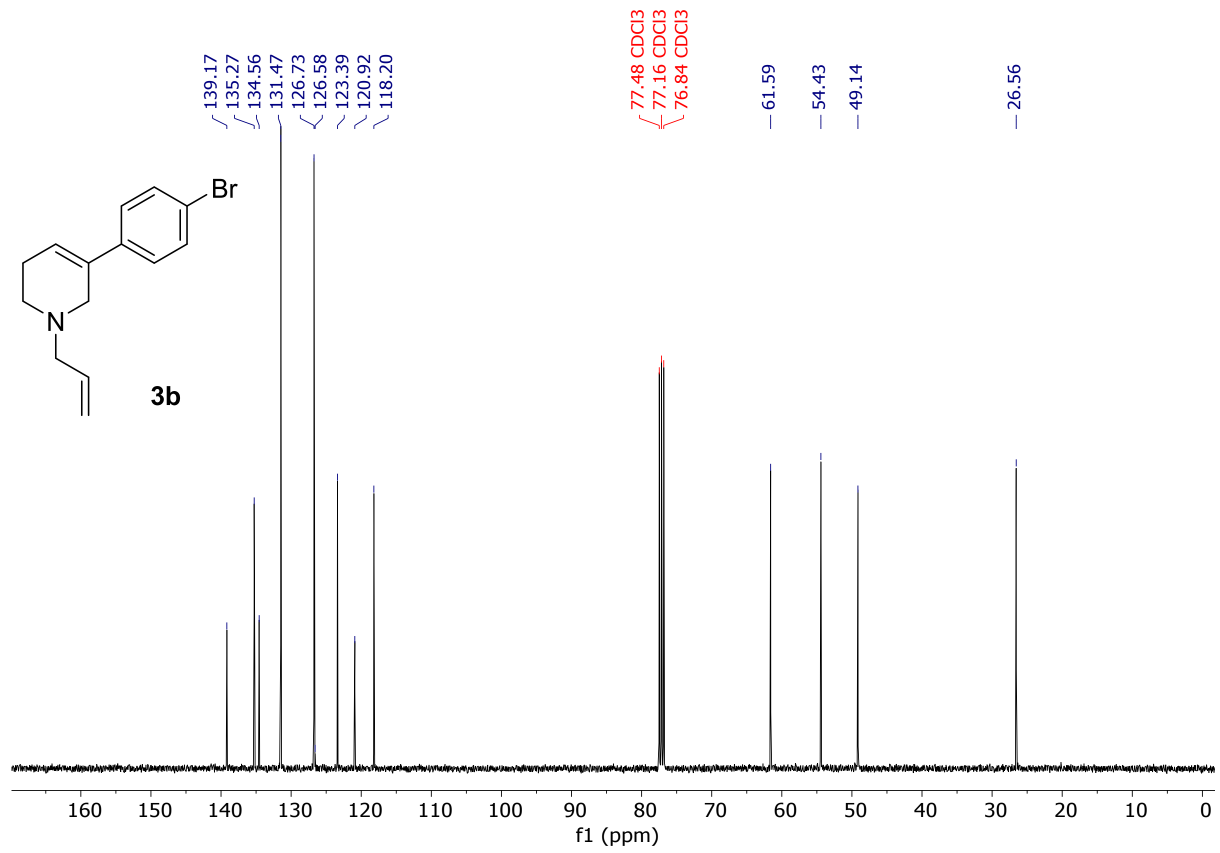

^{1}H & ^{13}C NMR spectra for *N*-(4-(1-allyl-1,2,5,6-tetrahydropyridin-3-yl)phenyl)acetamide

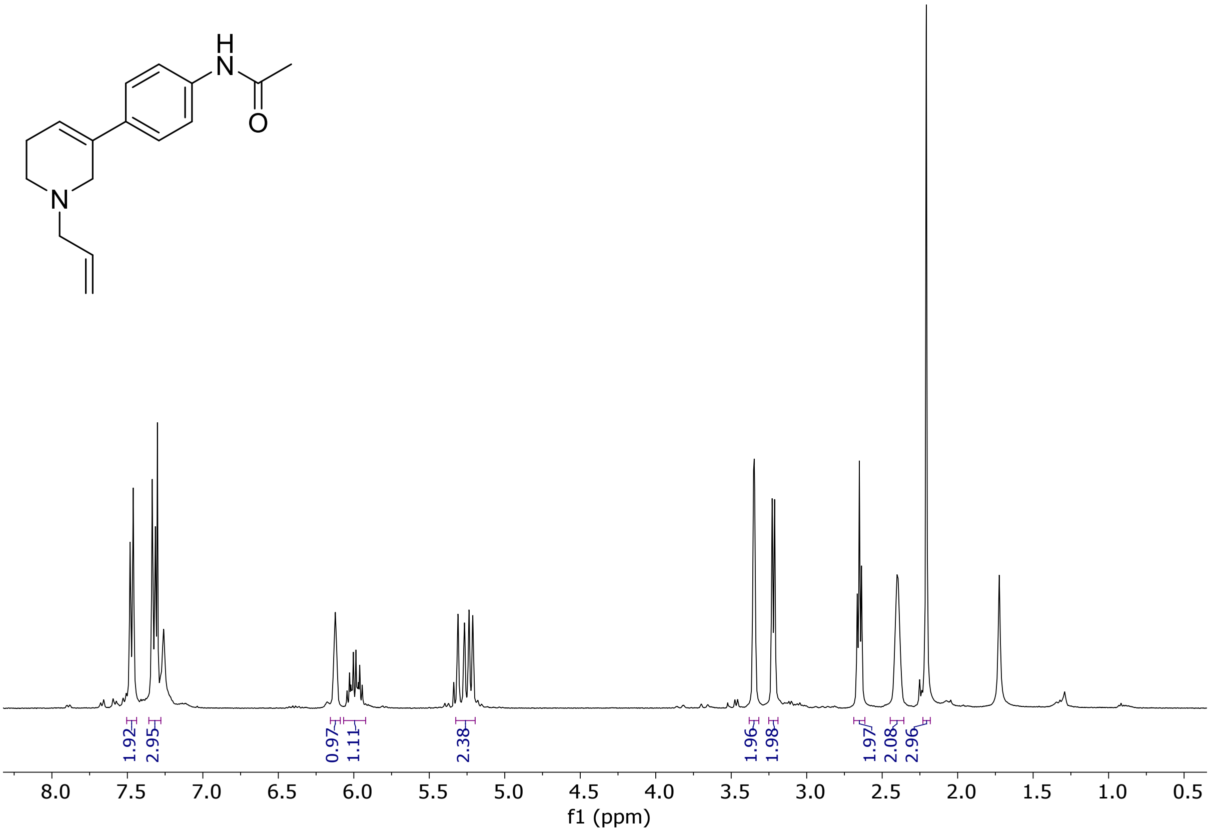

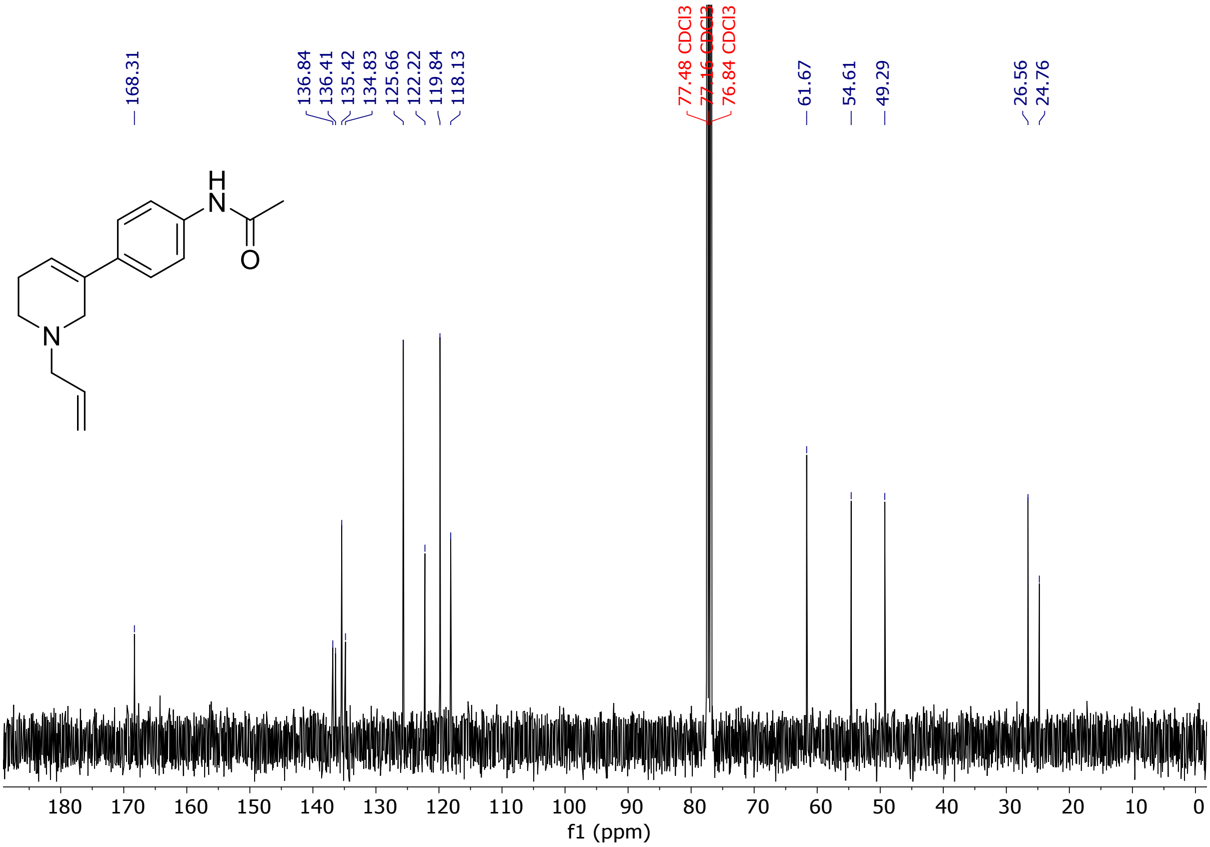

^{1}H & ^{13}C NMR spectra for 4-(1-allyl-1,2,5,6-tetrahydropyridin-3-yl)aniline (4b)

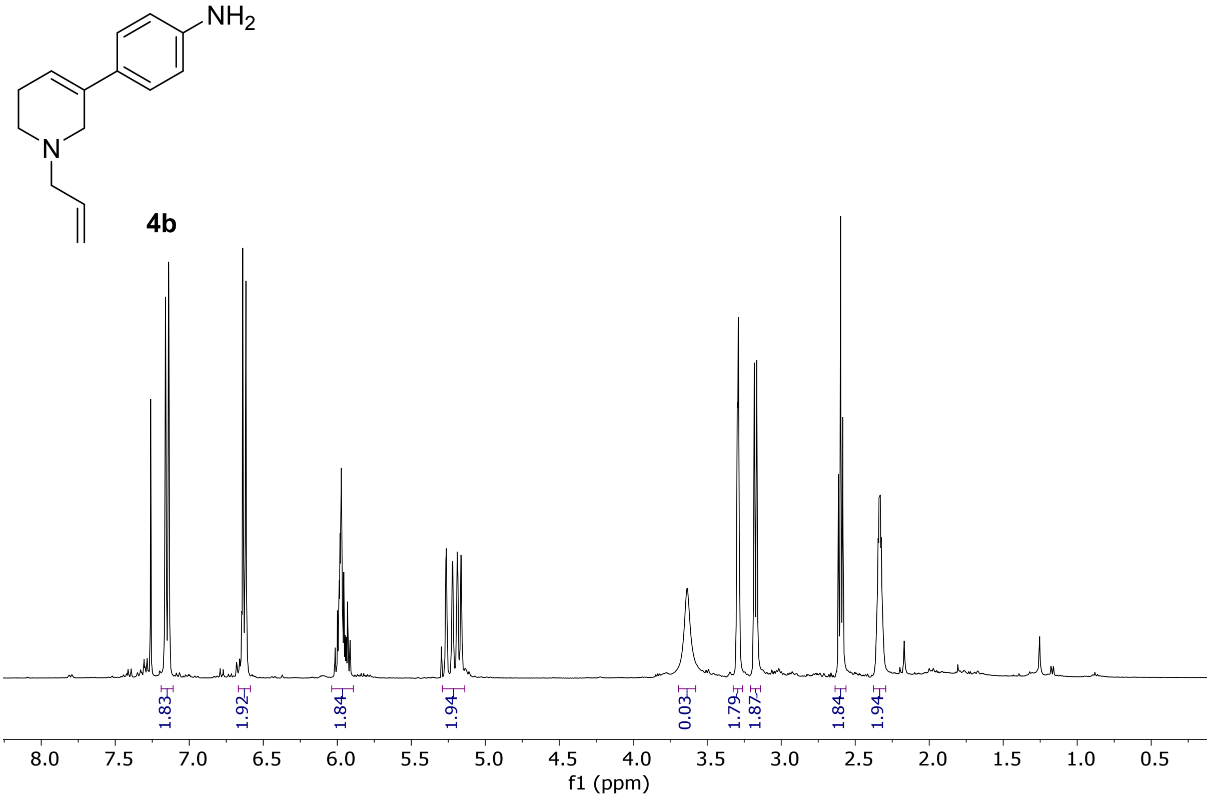

NH2
N
4b
1.83
1.92
1.84
1.94
0.03
1.79
1.87
1.84
1.94
8.0 7.5 7.0 6.5 6.0 5.5 5.0 4.5 4.0 3.5 3.0 2.5 2.0 1.5 1.0 0.5
f1 (ppm)

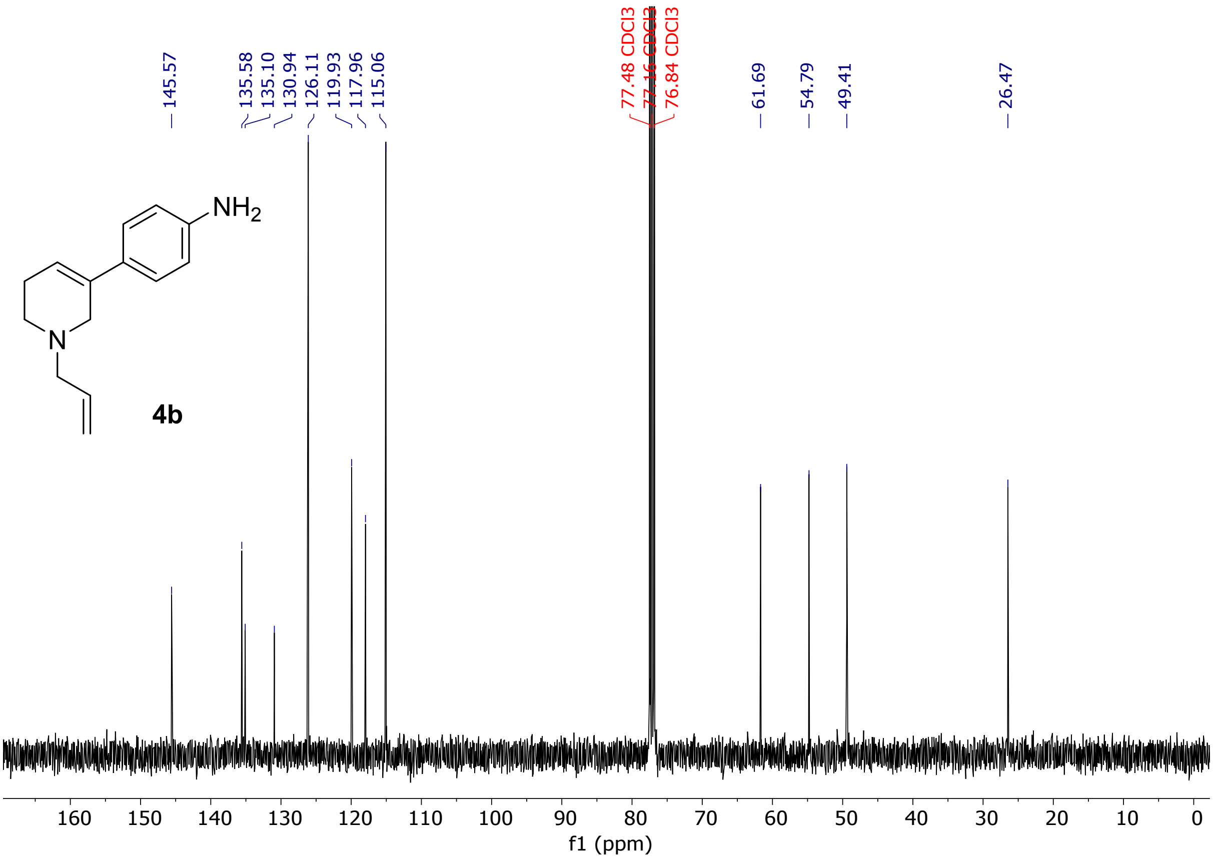

145.57
135.58
135.10
130.94
126.11
119.93
117.96
115.06
77.48 CDCl3
77.16 CDCl3
76.84 CDCl3
61.69
54.79
49.41
26.47
NH2
N
4b
160 150 140 130 120 110 100 90 80 70 60 50 40 30 20 10 0
f1 (ppm)

^{1}H & ^{13}C NMR spectra for 1-ethyl-5-phenyl-1,2,3,6-THP (5b)

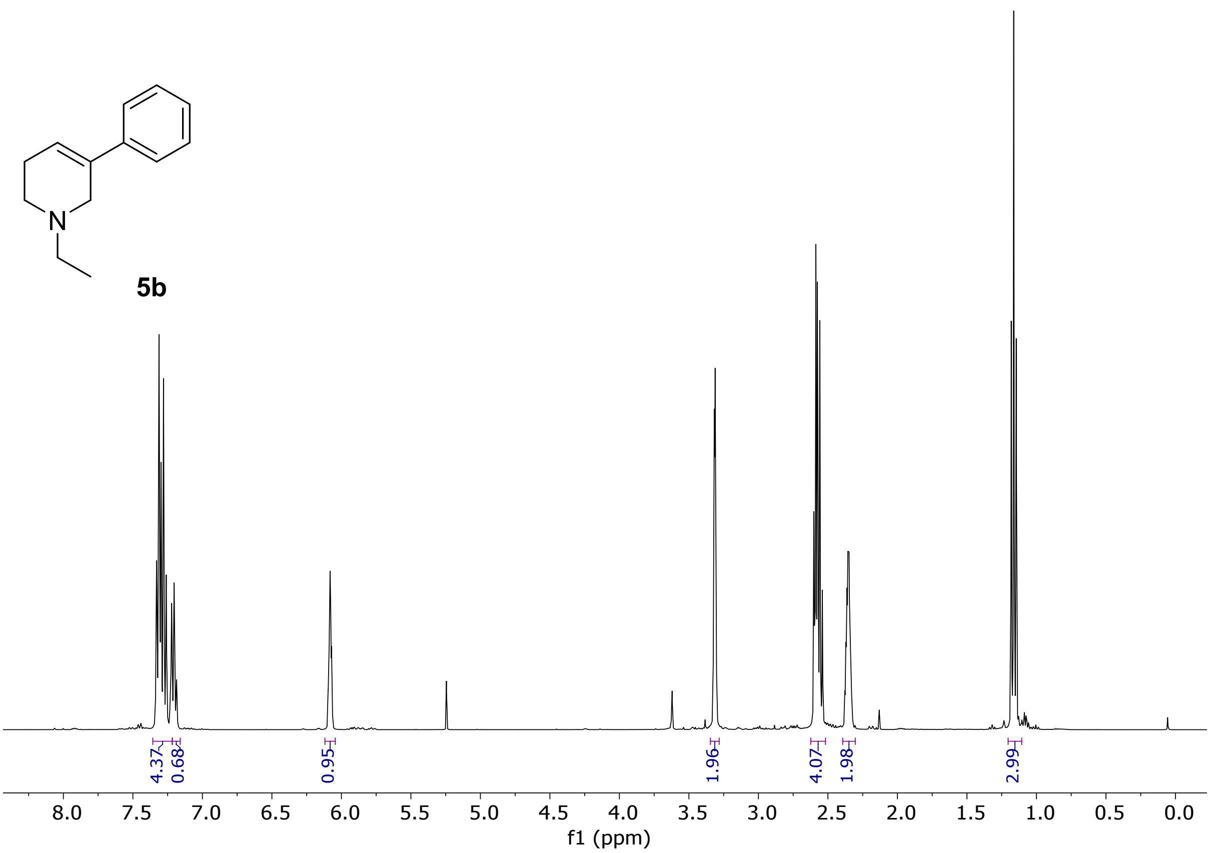

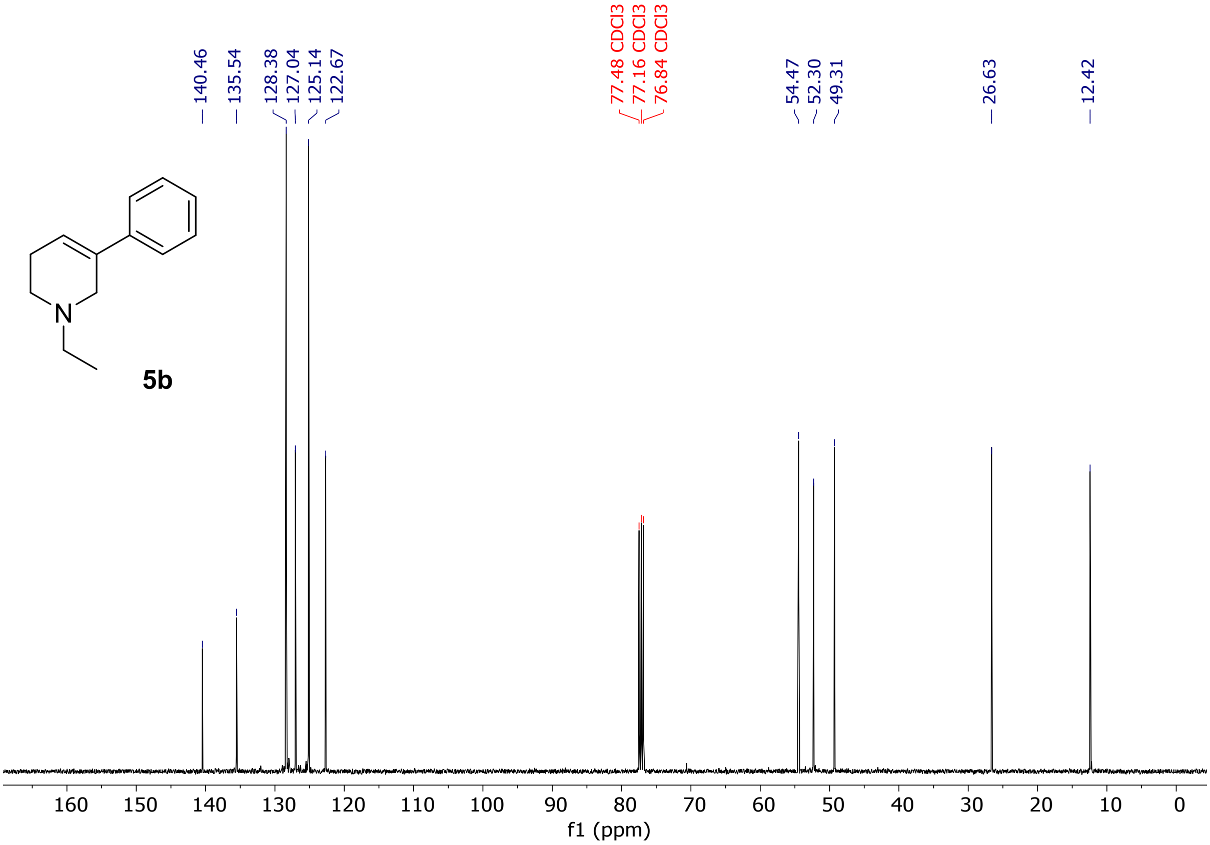

^{1}H & ^{13}C NMR spectra for 1-ethyl-5-(4-fluorophenyl)-1,2,3,6-THP (6b)

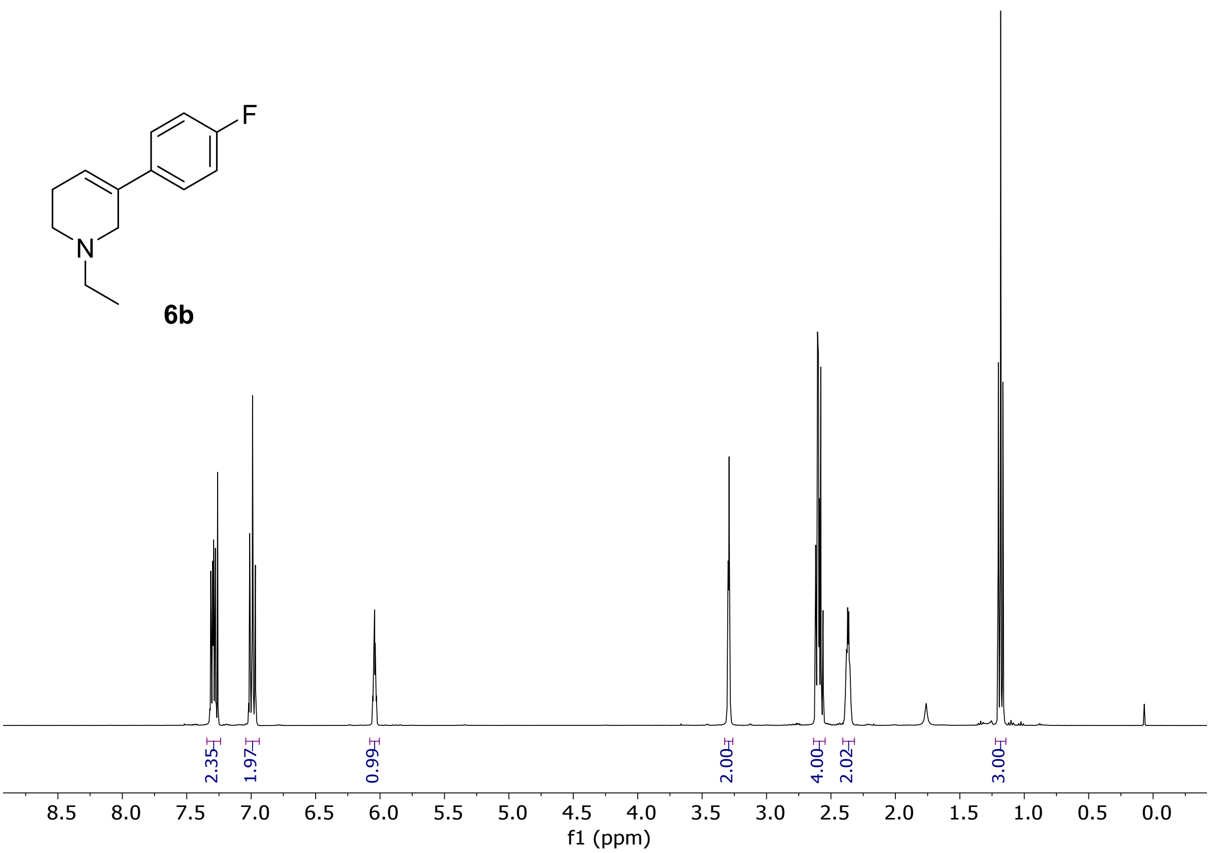

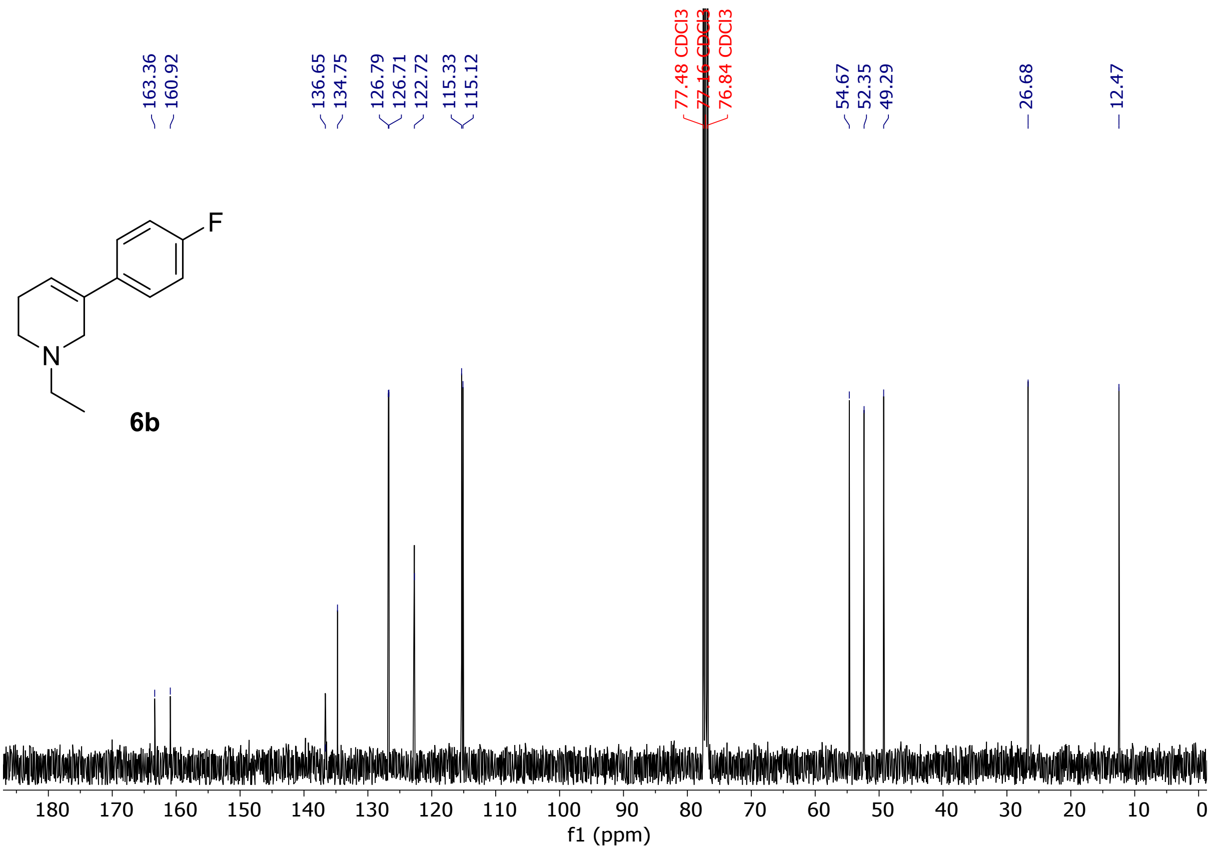

^{1}H & ^{13}C NMR spectra for 1-ethyl-5-(4-methoxyphenyl)-1,2,3,6-THP (7b)

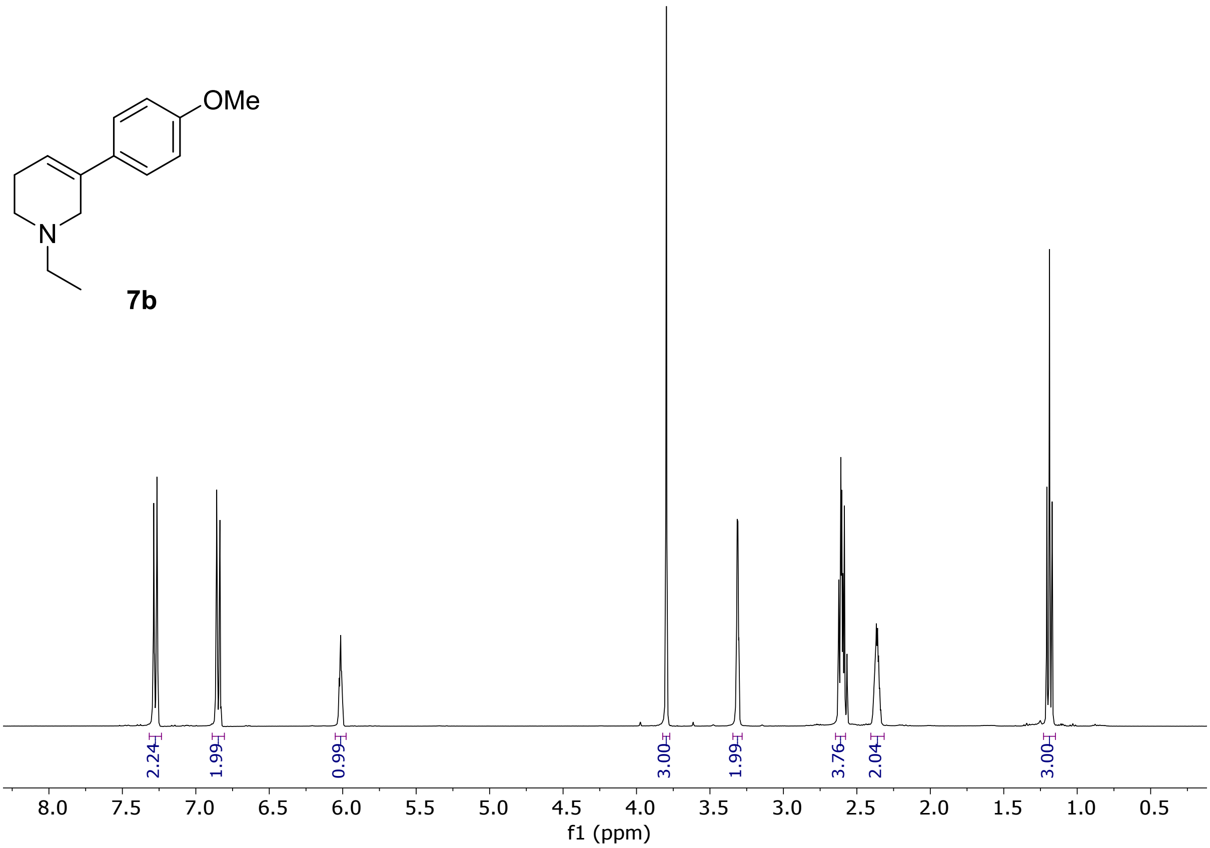

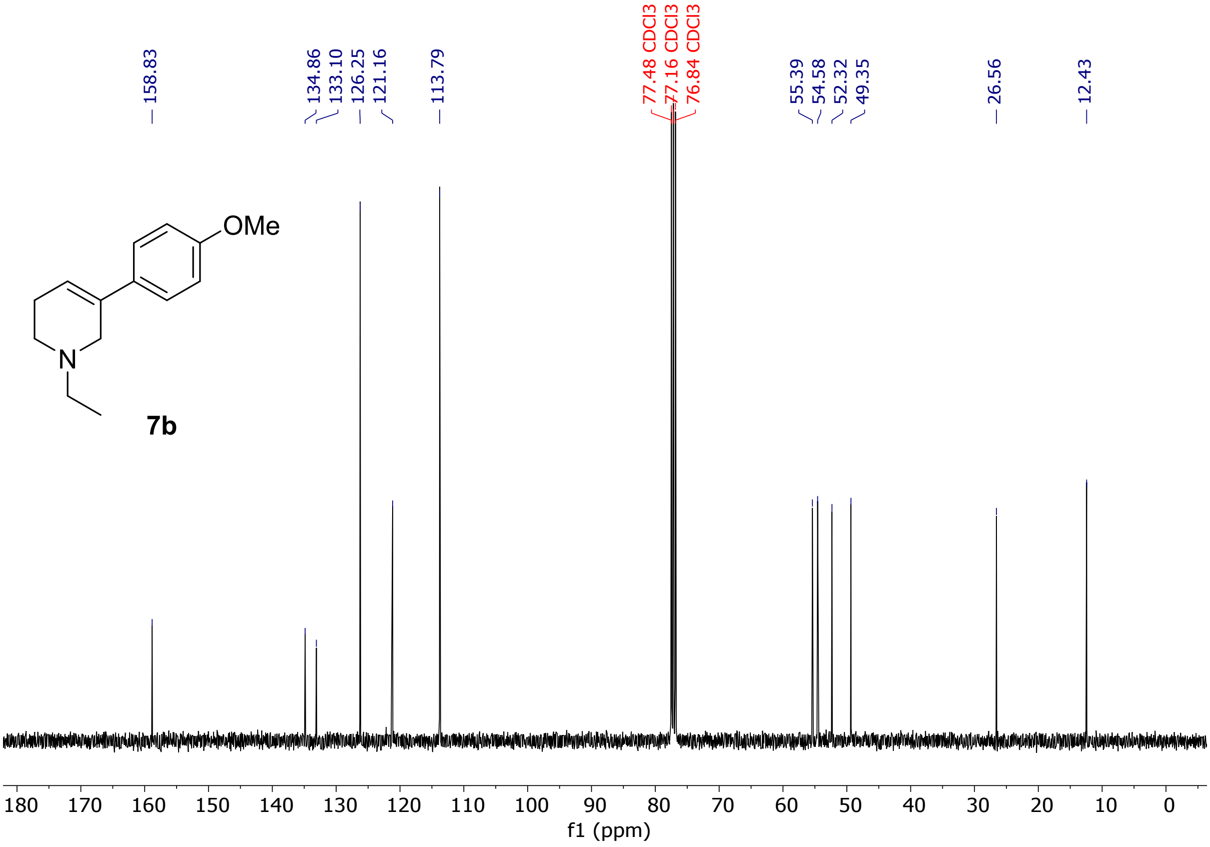

^{1}H & ^{13}C NMR spectra for 1-ethyl-5-(furan-2-yl)-1,2,3,6-THP (8b)

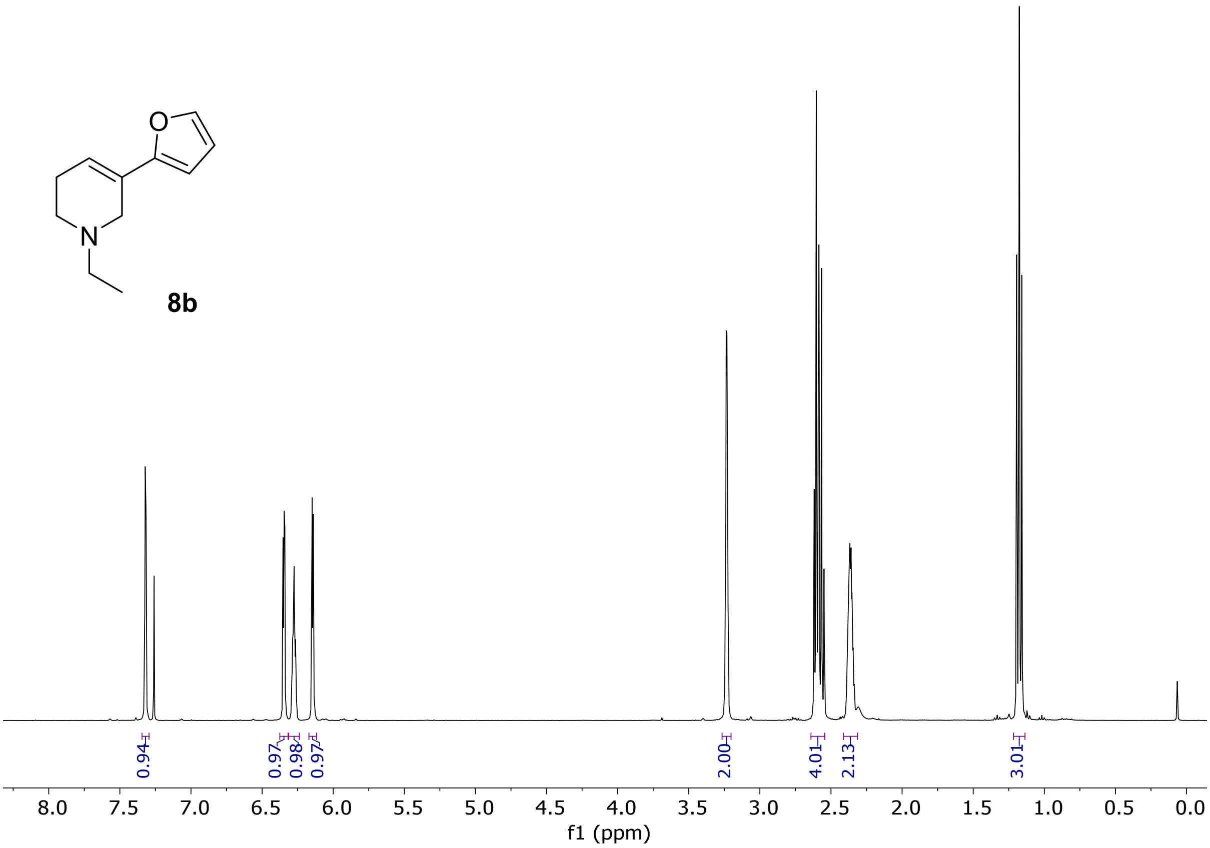

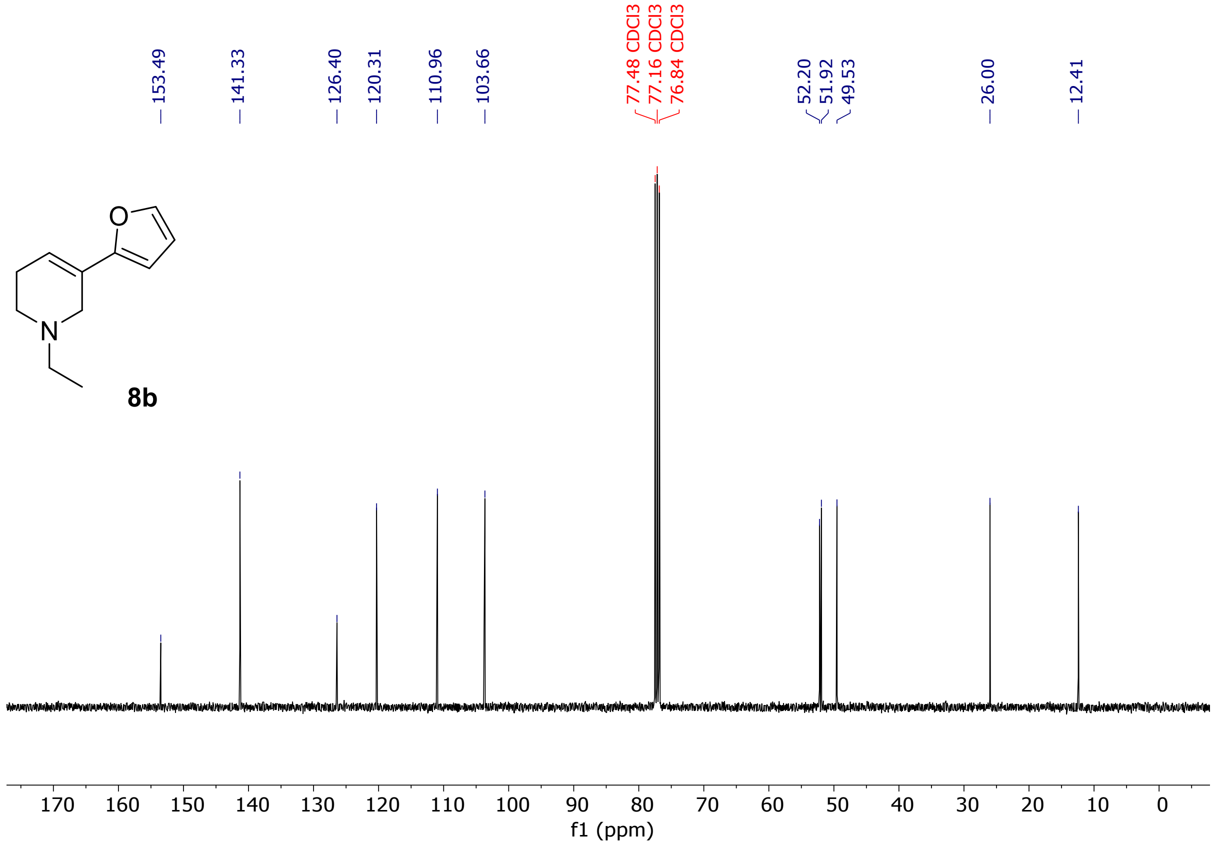

^{1}H & ^{13}C NMR spectra for 1-ethyl-5-(thiophen-2-yl)-1,2,3,6-THP (9b)

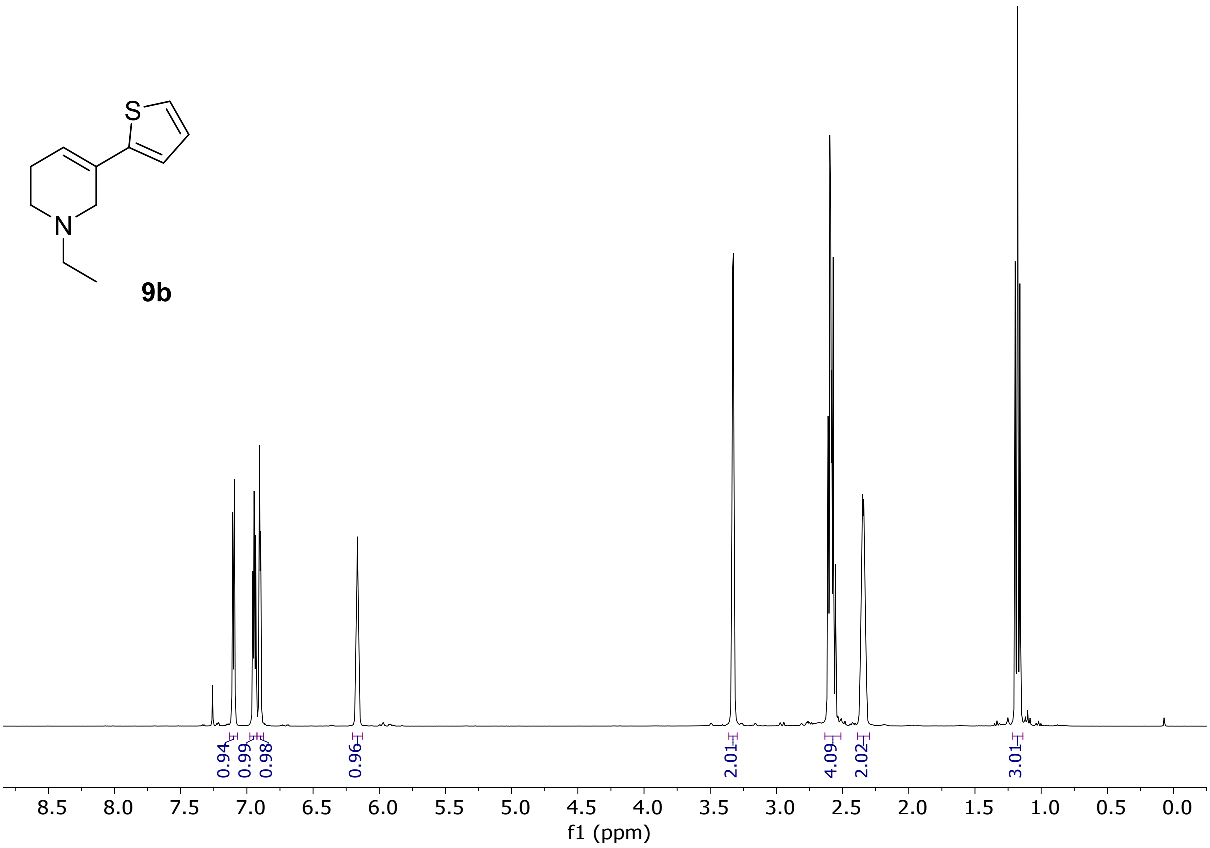

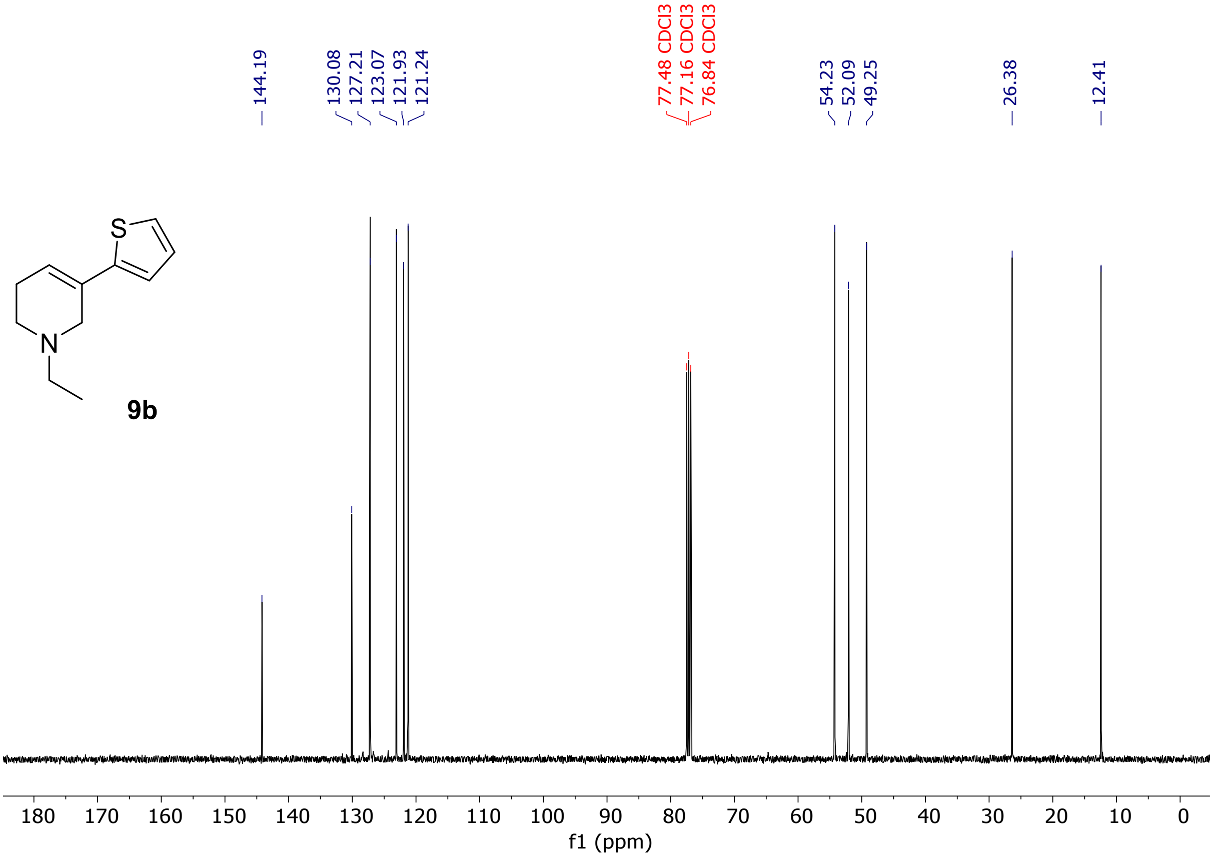

^{1}H & ^{13}C NMR spectra for 1-ethyl-5-(naphthalen-2-yl)-1,2,3,6-THP (10b)

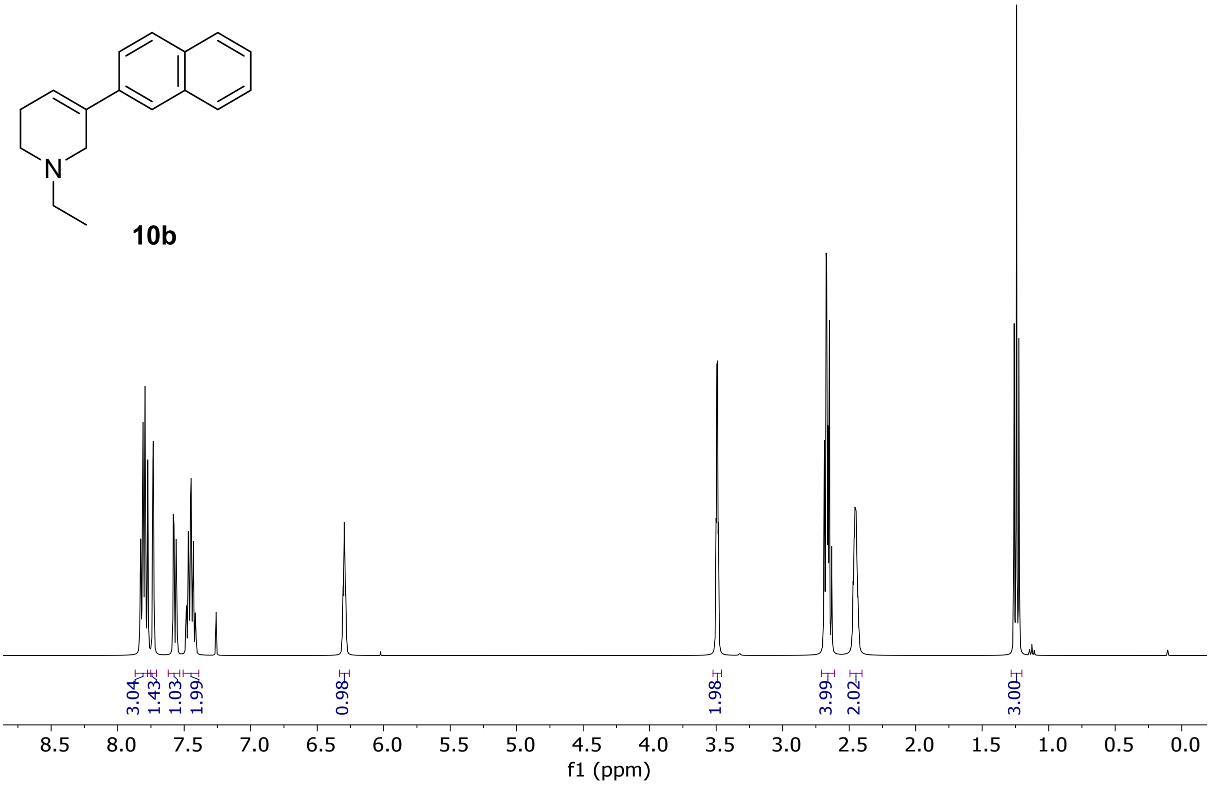

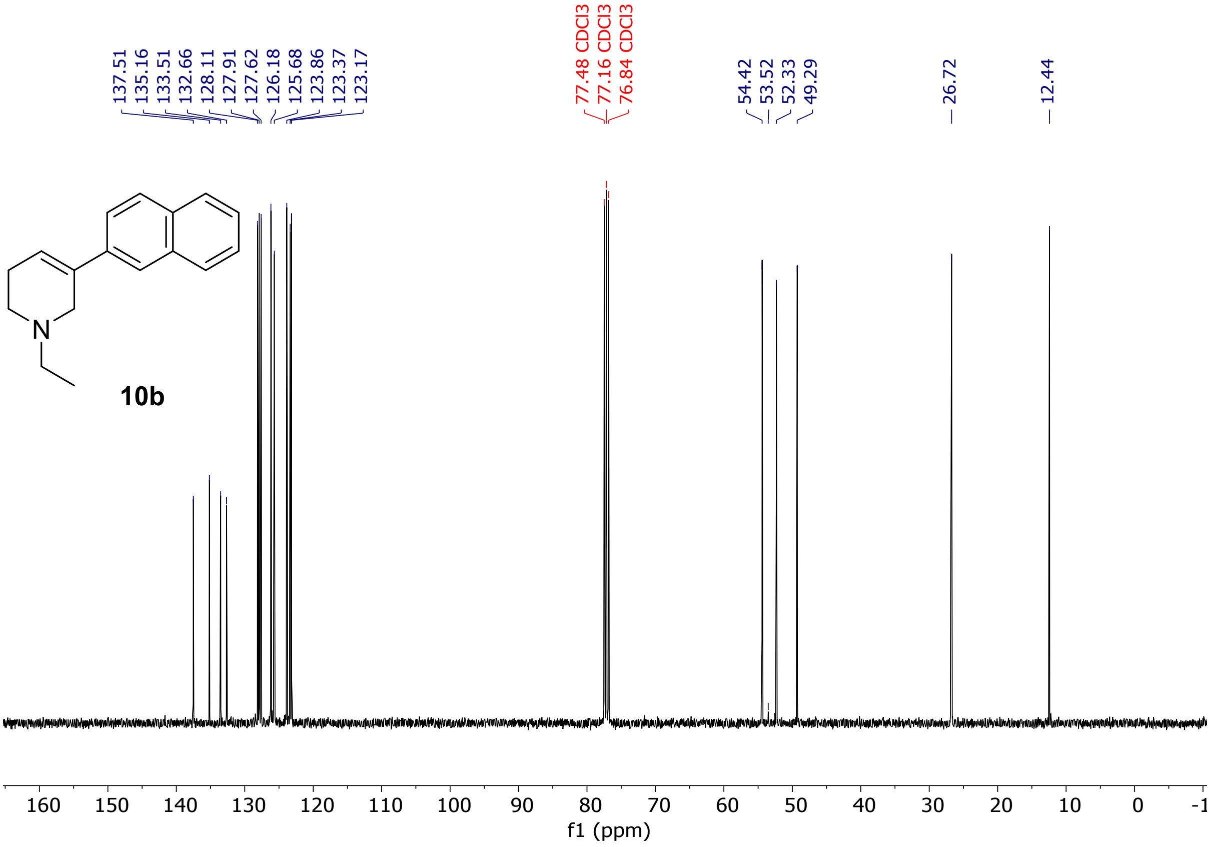

^{1}H & ^{13}C NMR spectra for 1-methyl-5-phenyl-1,2,3,6-THP (11b)

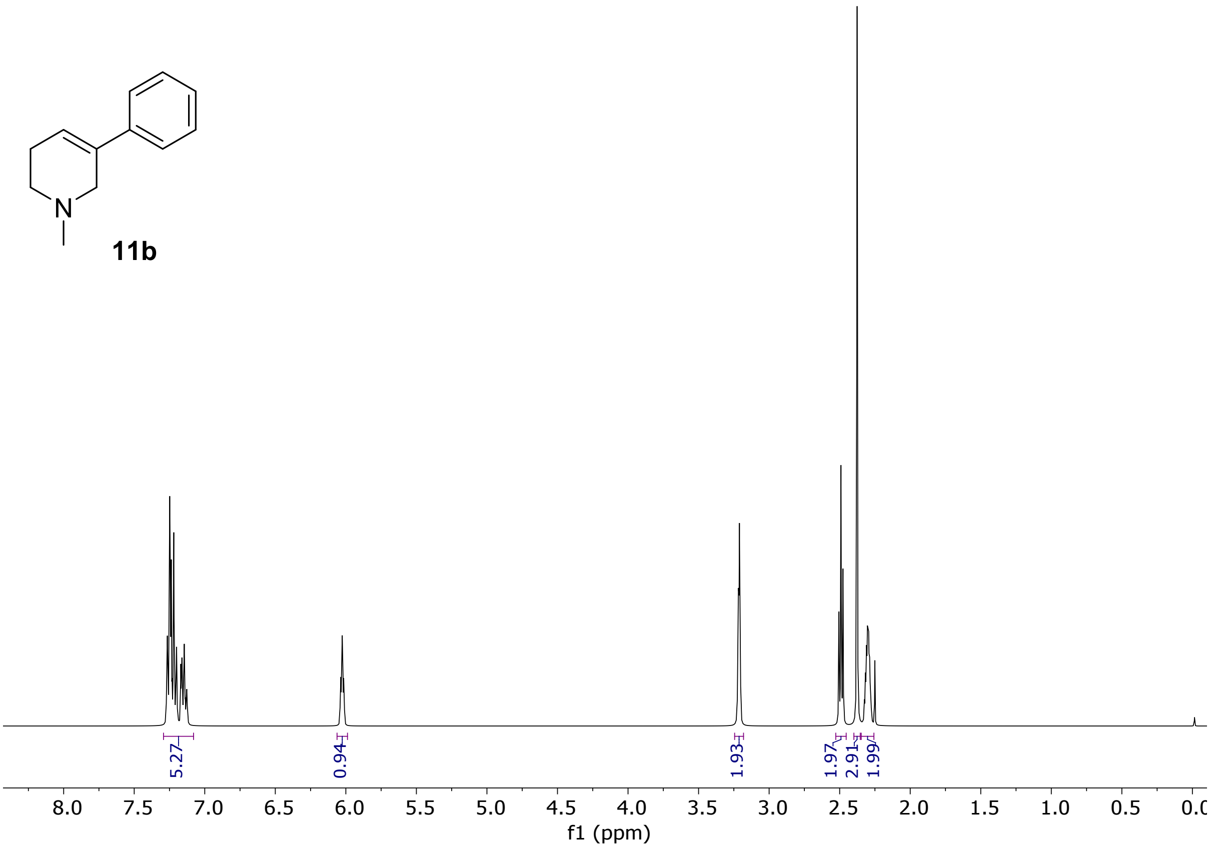

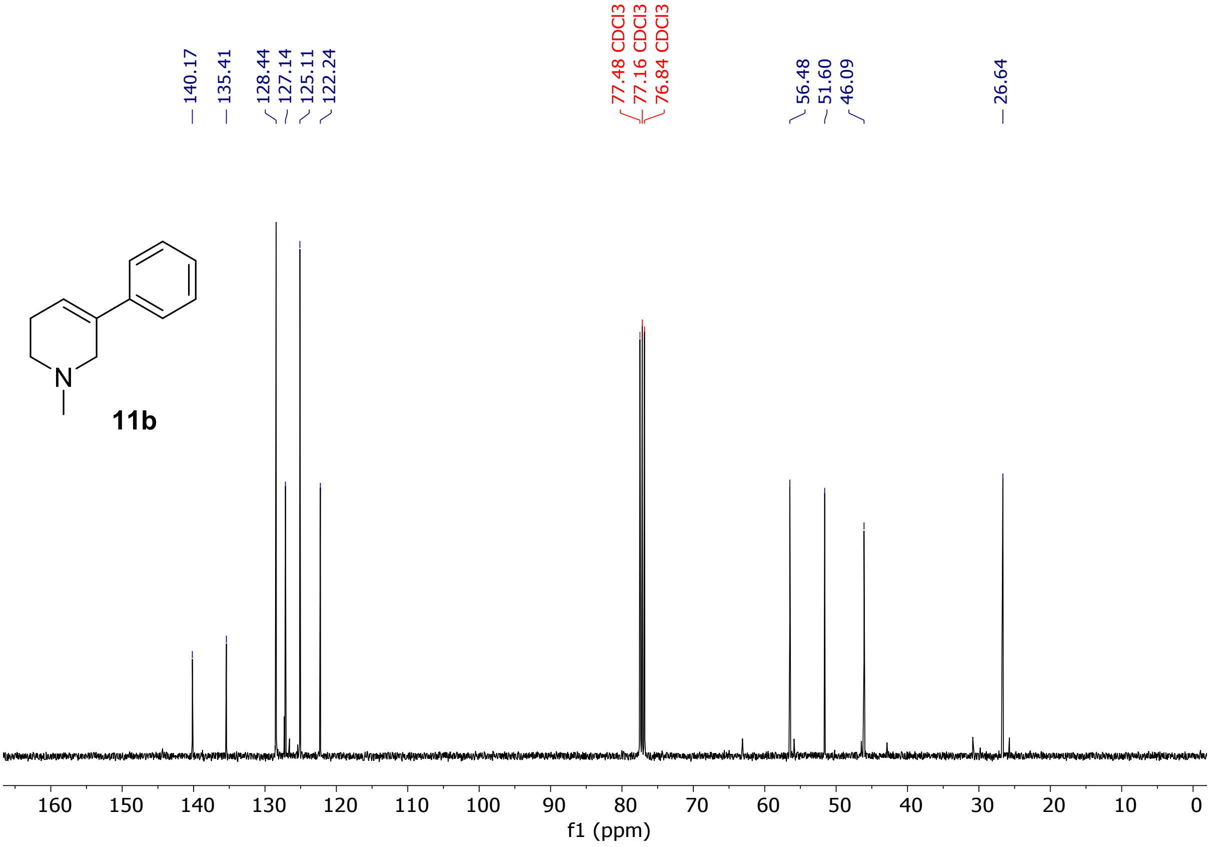

^{1}H & ^{13}C NMR spectra for 5-phenyl-1-(prop-2-yn-1-yl)-1,2,3,6-THP (12b)

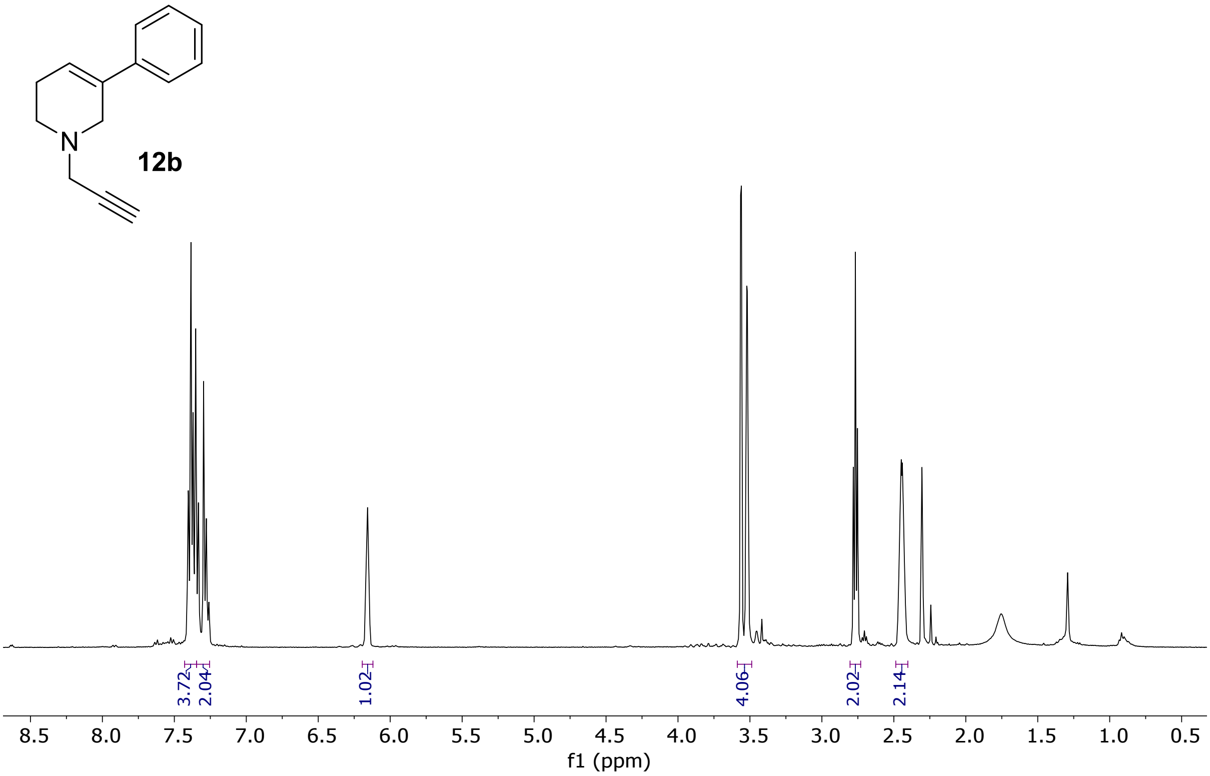

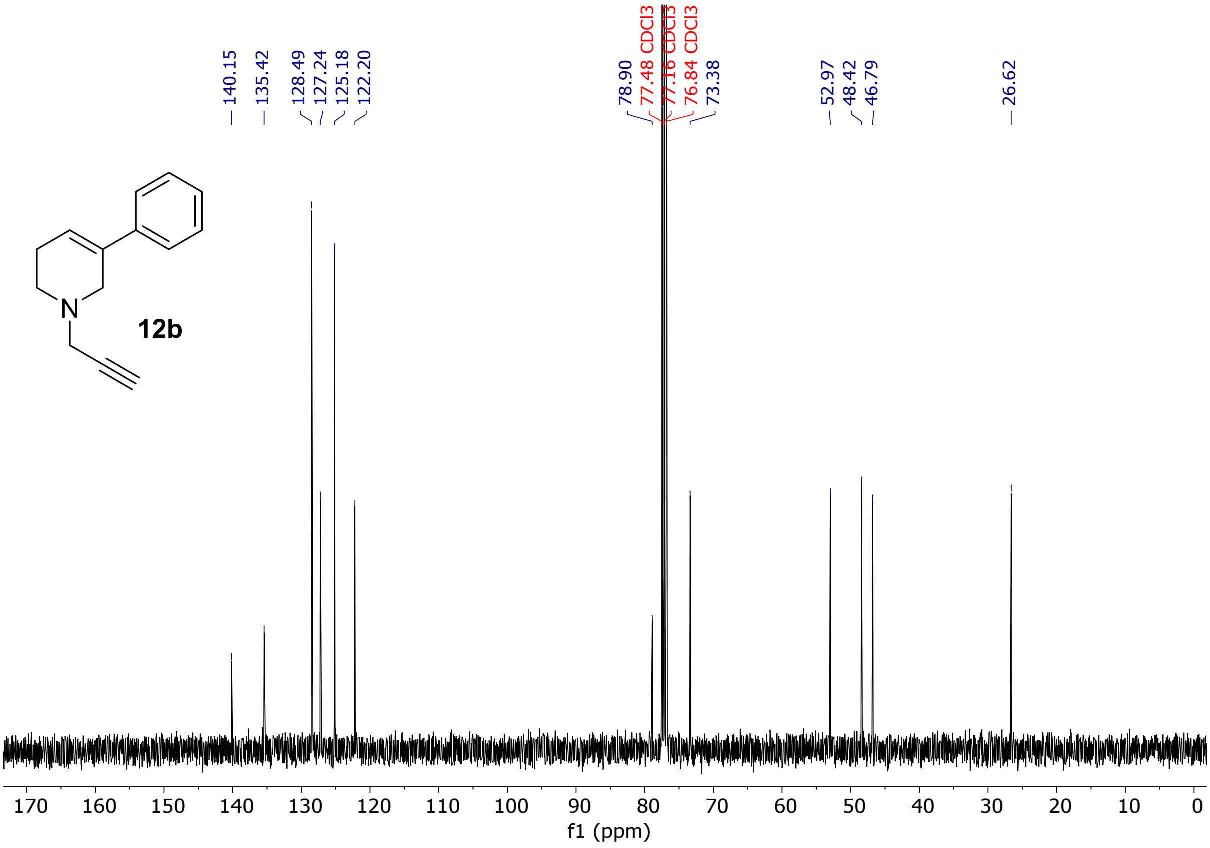

^{1}H & ^{13}C NMR spectra for 1-ethyl-5-methyl-1,2,3,6-THP (13b)

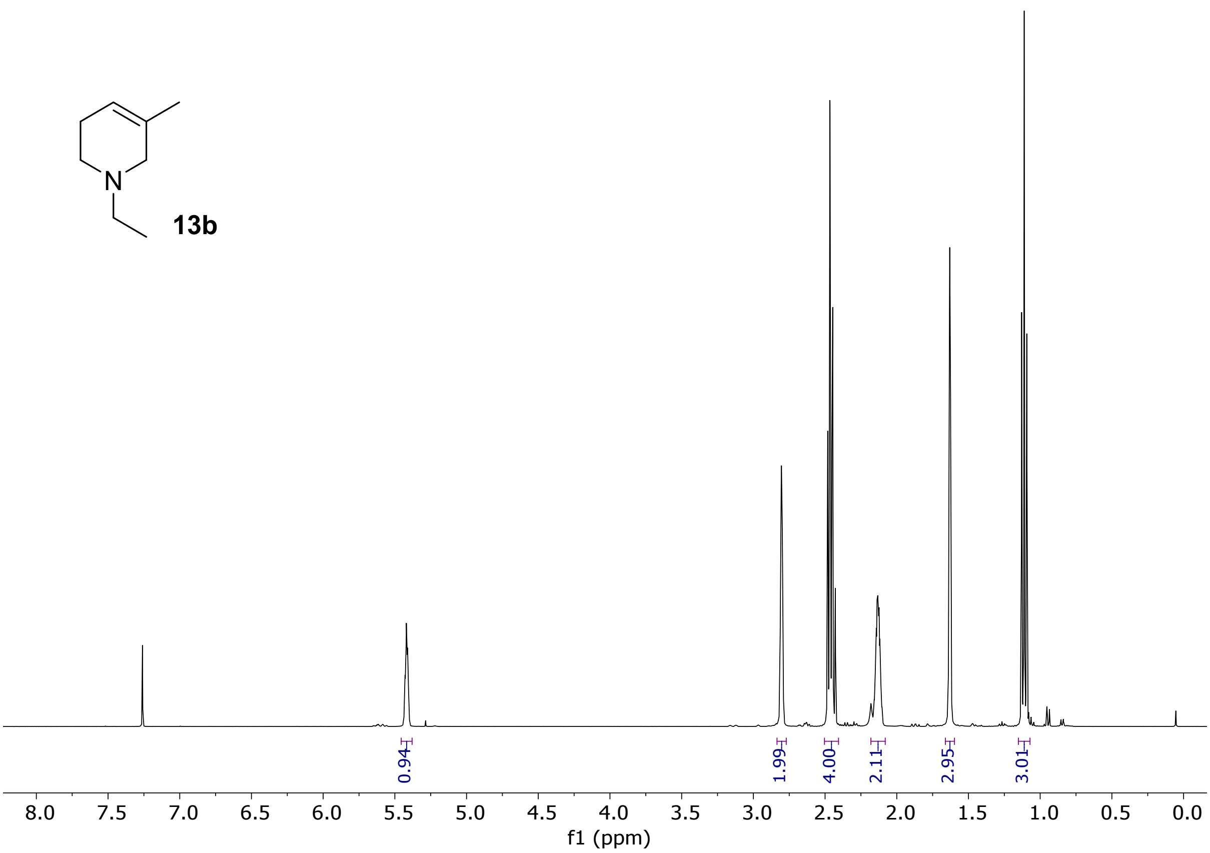

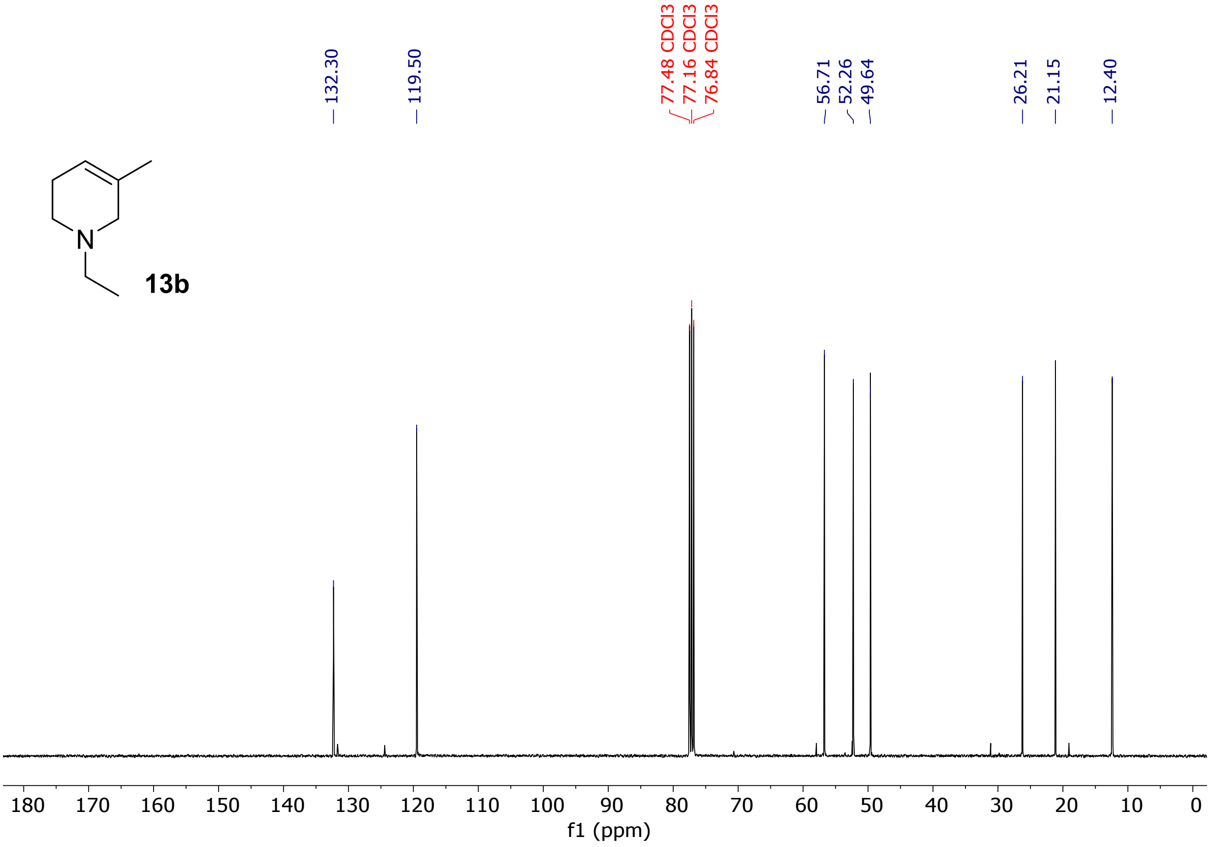

^{1}H & ^{13}C NMR spectra for 1-benzyl-5-methyl-1,2,3,6-THP (14b)

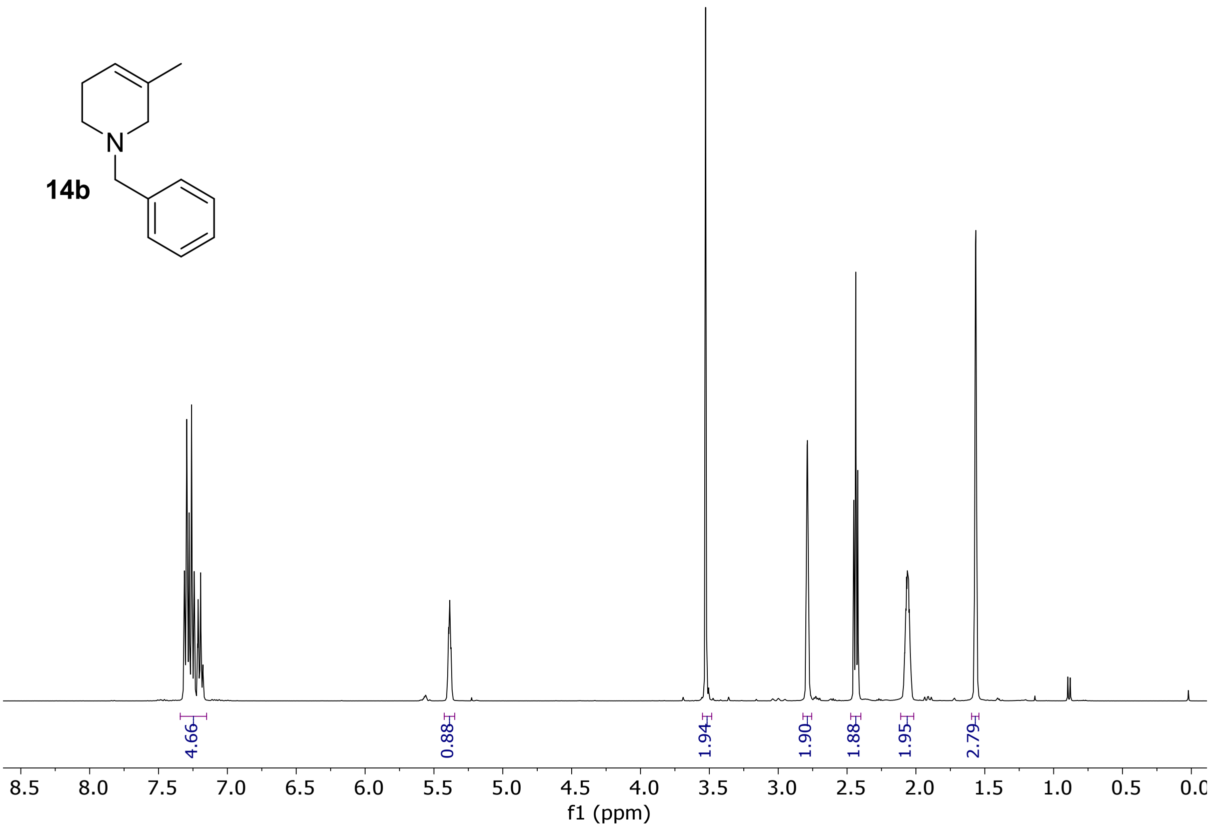

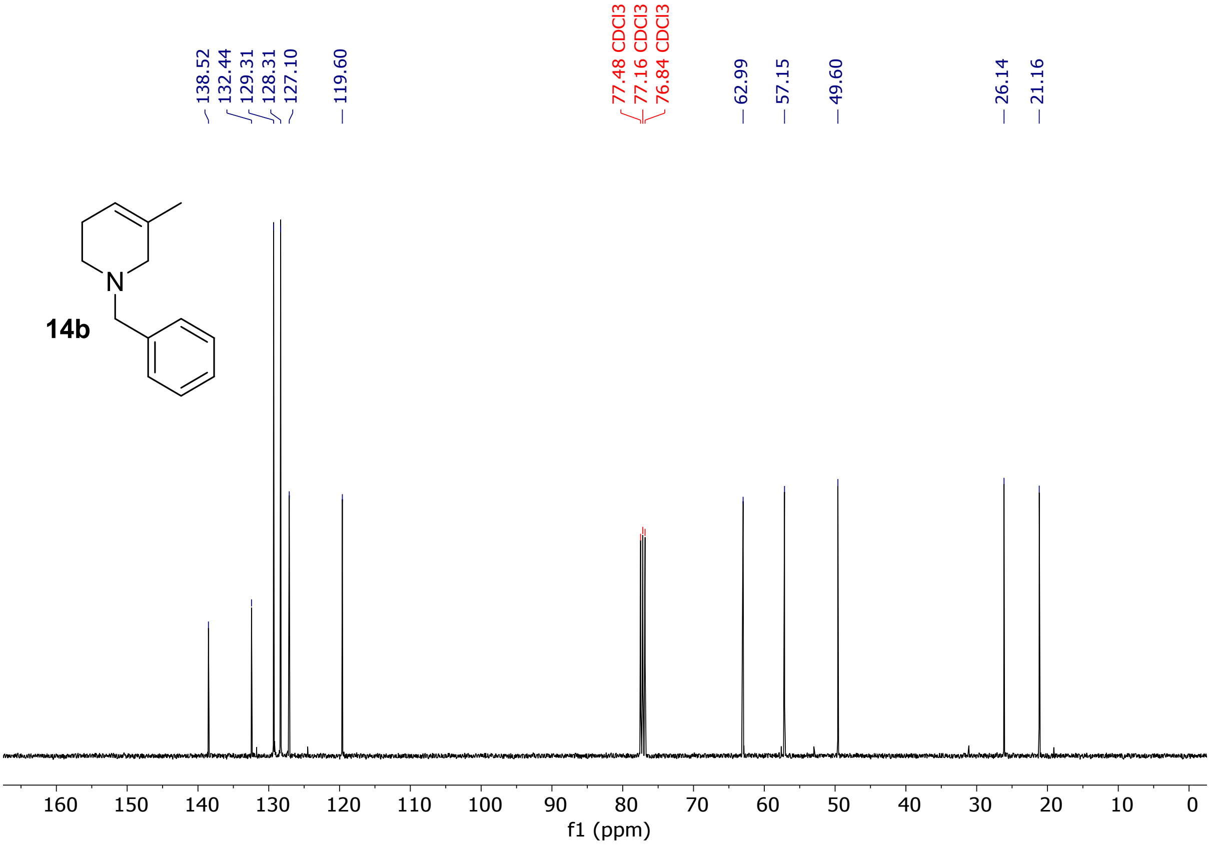

^{1}H & ^{13}C NMR spectra for 1-ethyl-4-methyl-1,2,3,6-THP (15b)

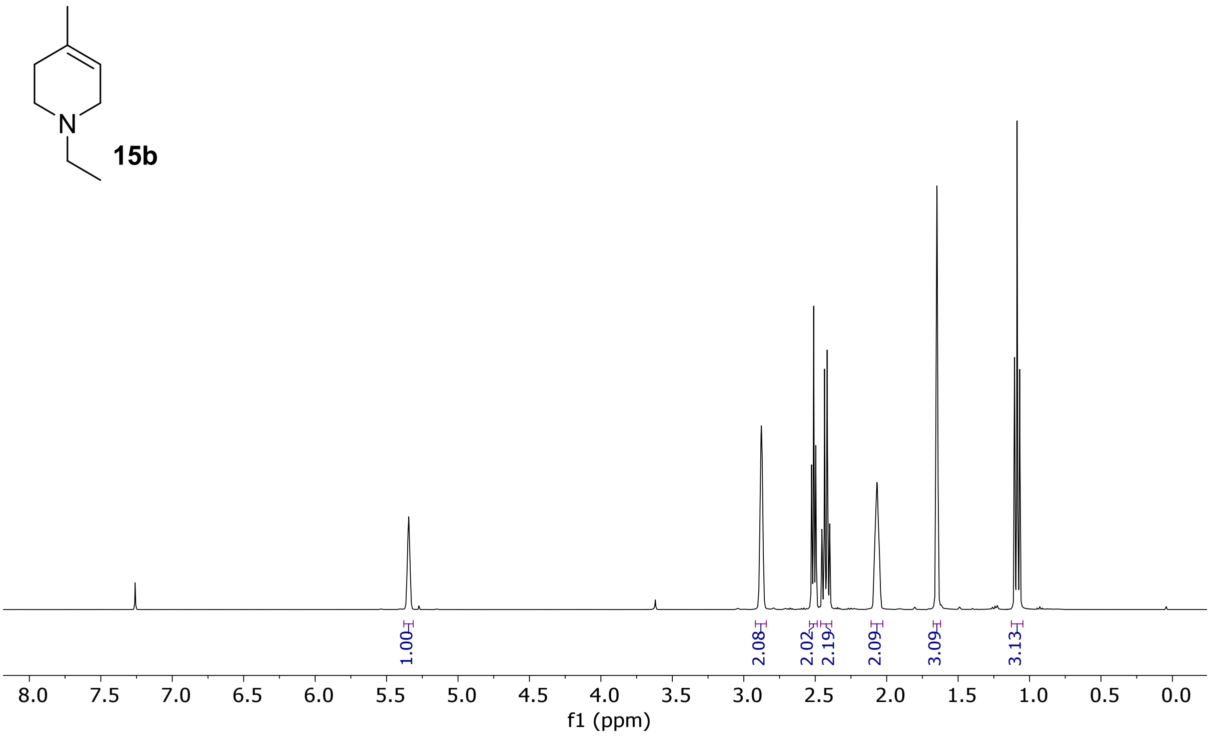

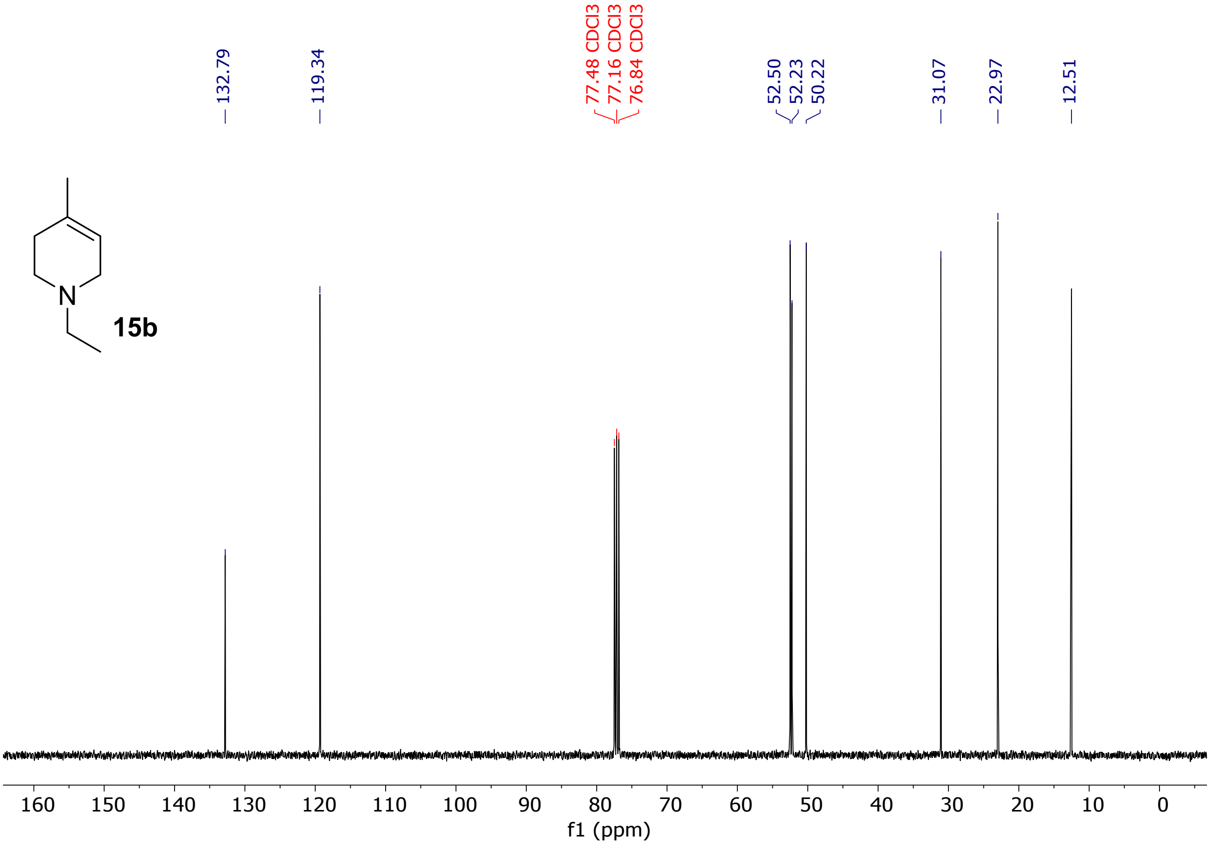

^{1}H & ^{13}C NMR spectra for 1-ethyl-4-phenyl-1,2,3,6-THP (16b)

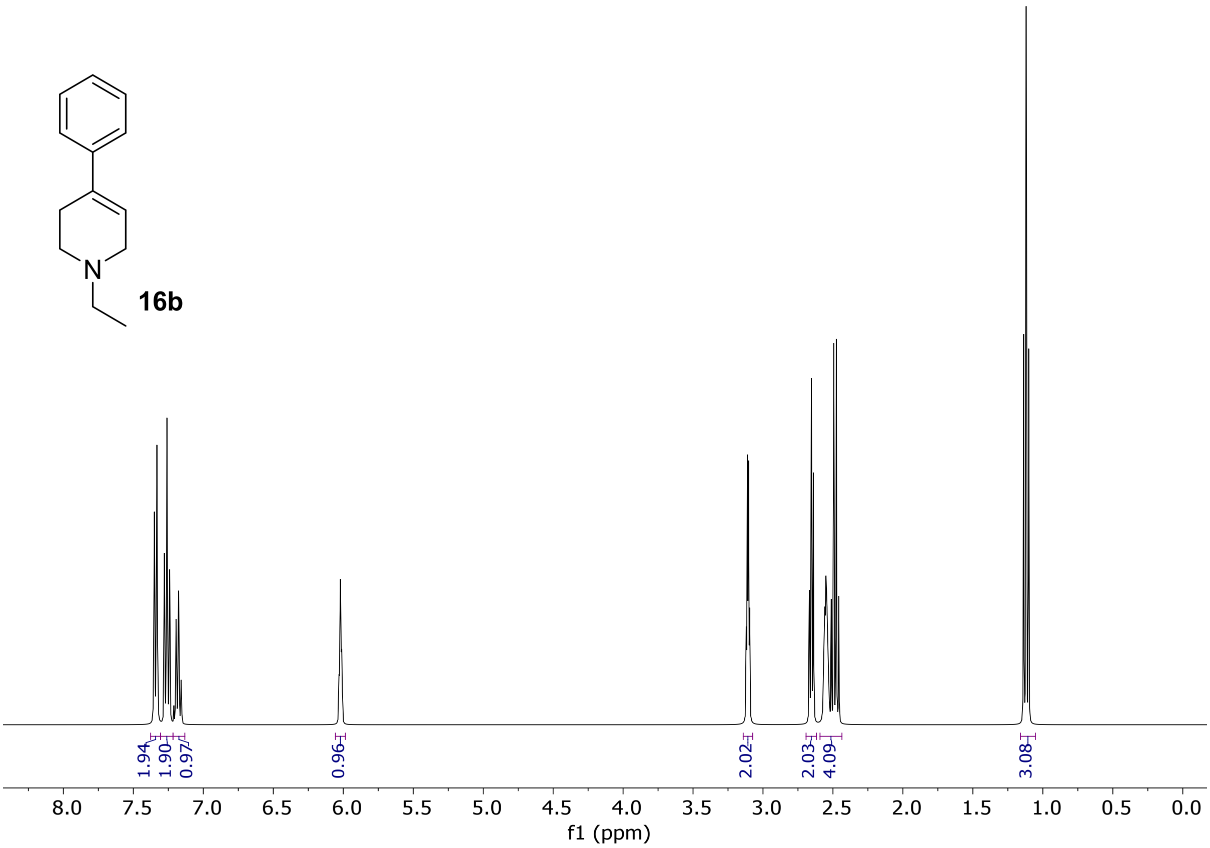

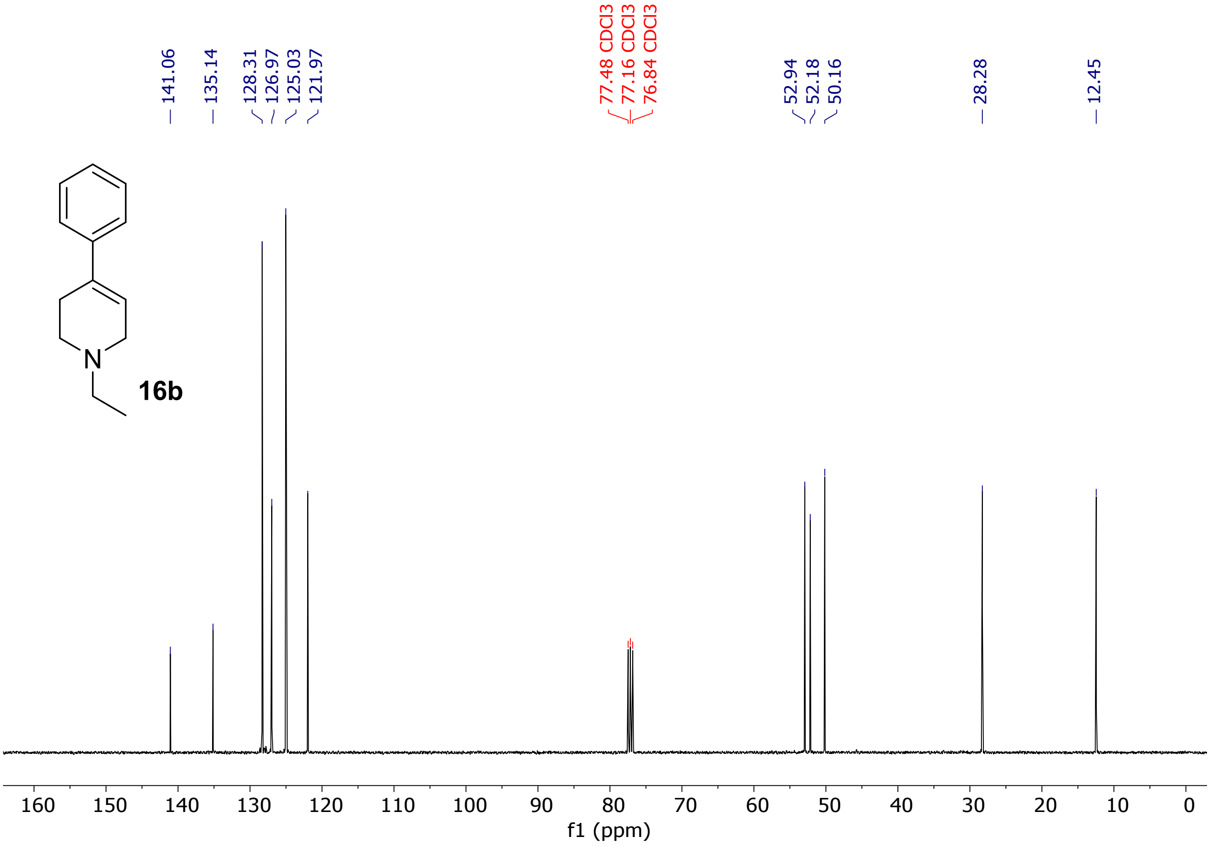

^{1}H & ^{13}C NMR spectra for 1-ethyl-4,5-dimethyl-1,2,3,6-THP (17b.HCl salt)

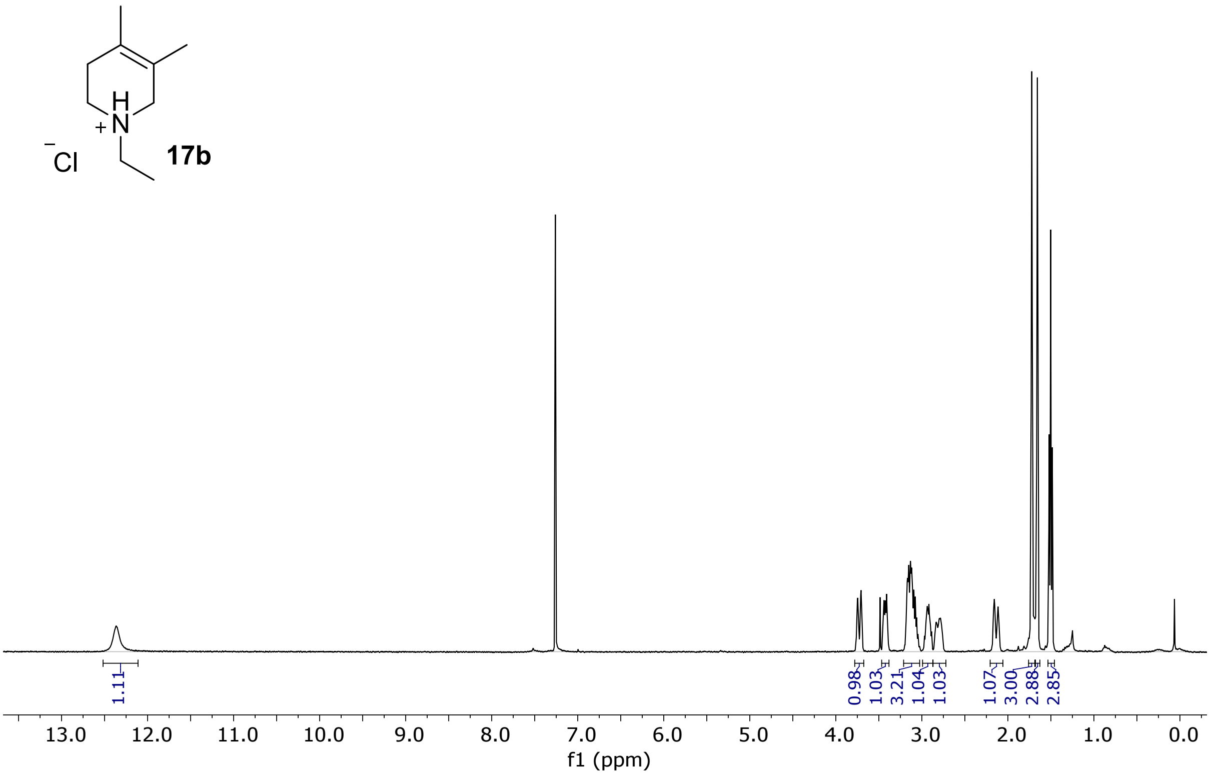

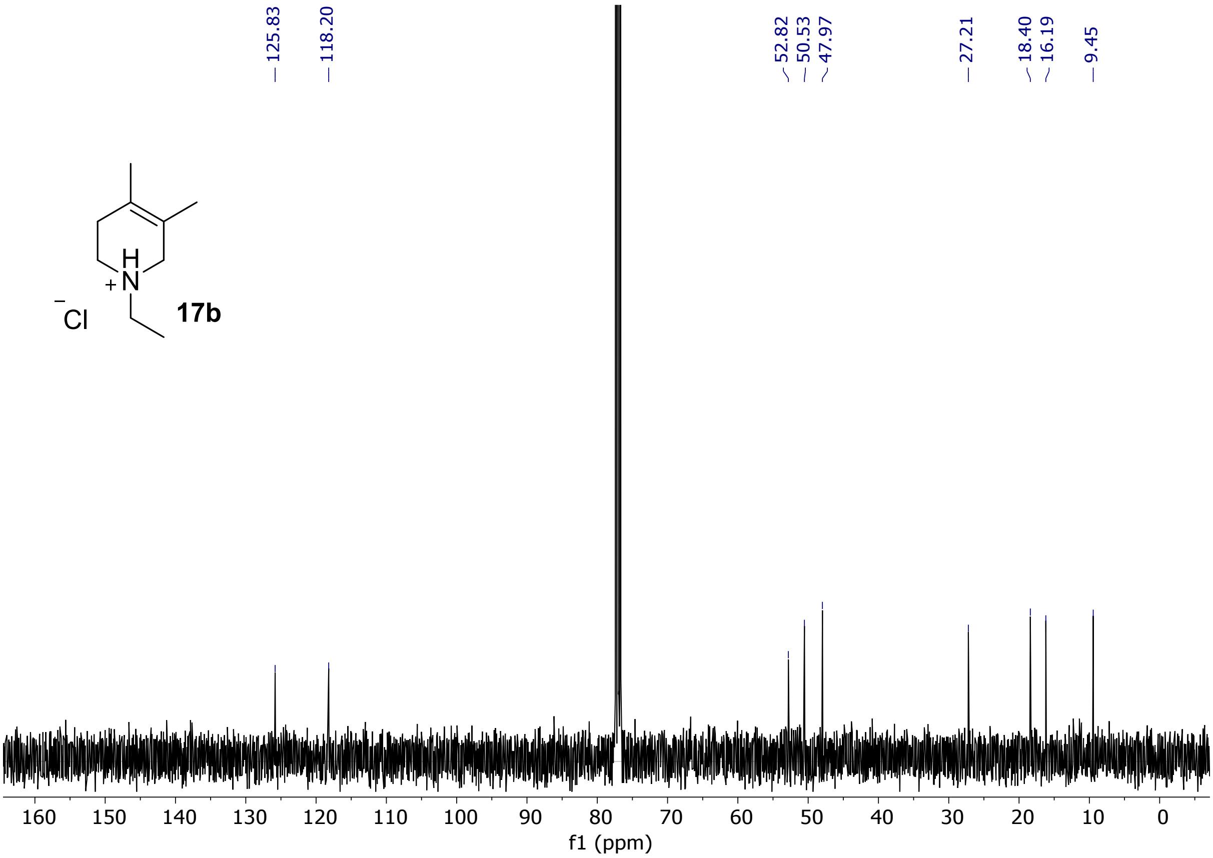

^{1}H & ^{13}C NMR spectra for 2-ethyl-1,2,3,4,5,6,7,8-octahydroisoquinoline (18b)

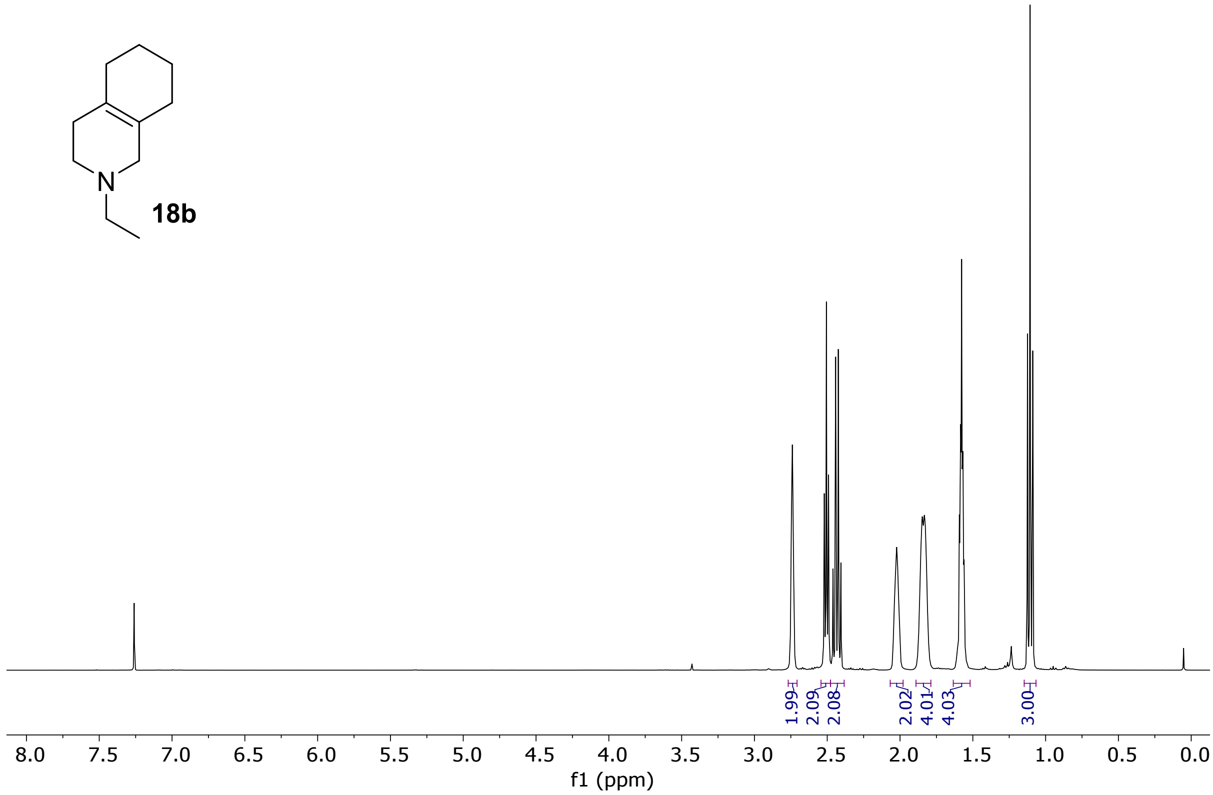

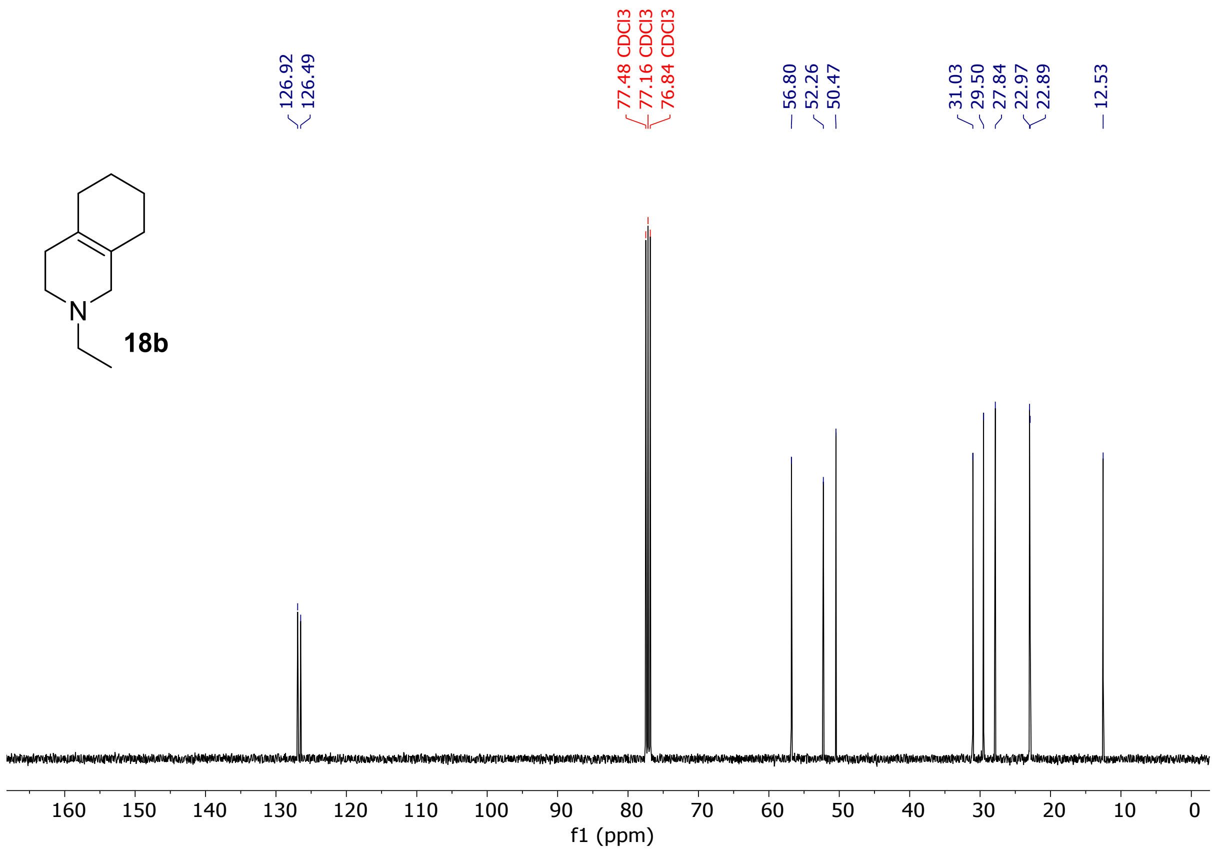

^{1}H & ^{13}C NMR spectra for 1-ethyl-4-methyl-5-phenyl-1,2,3,6-THP (19b)

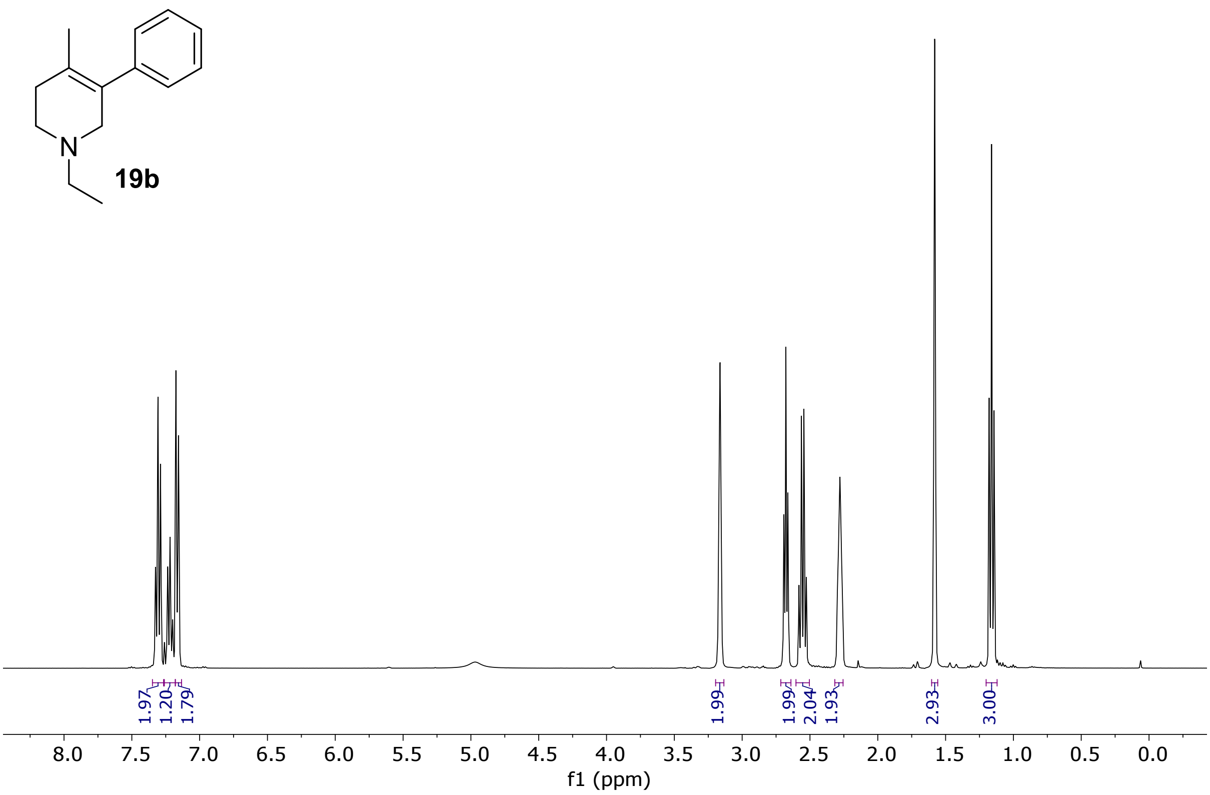

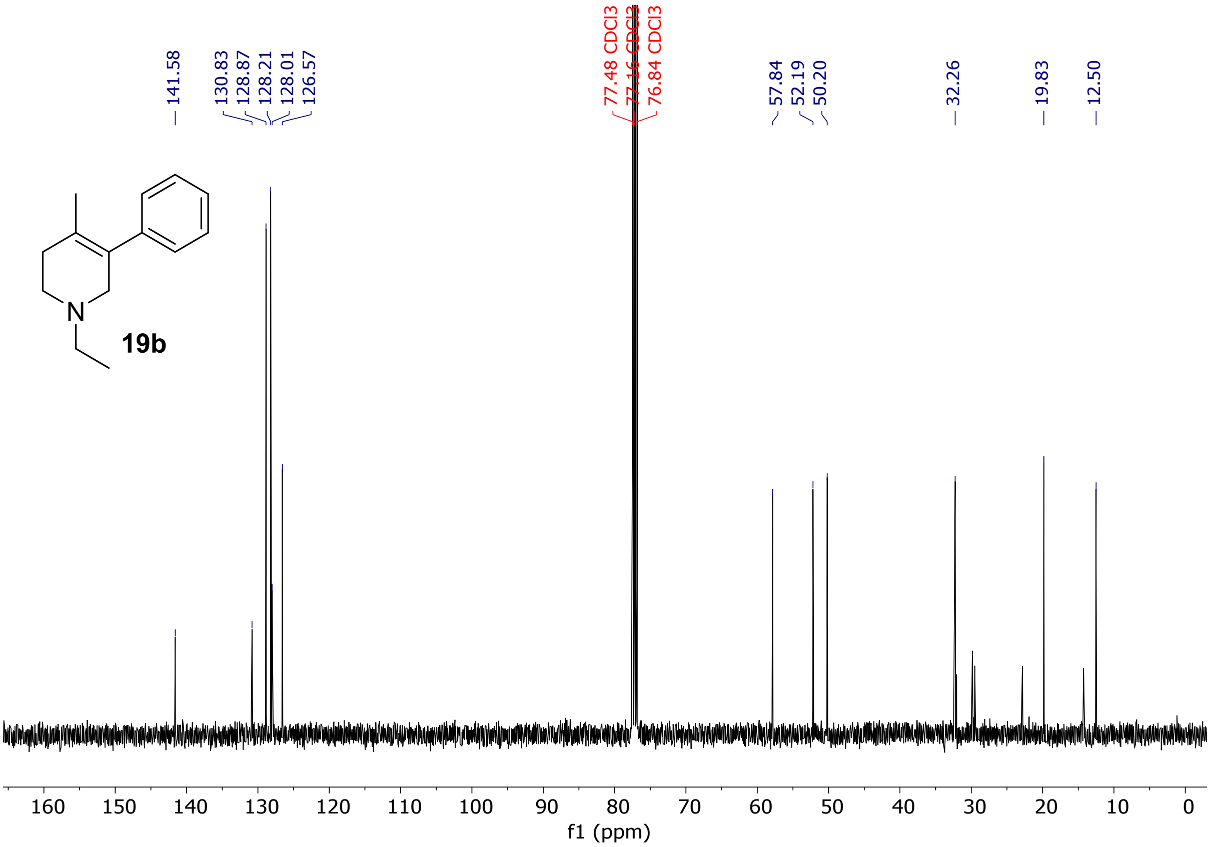

^{1}H & ^{13}C NMR spectra for 1-allyl-4-methyl-5-phenyl-1,2,3,6-THP (20b)

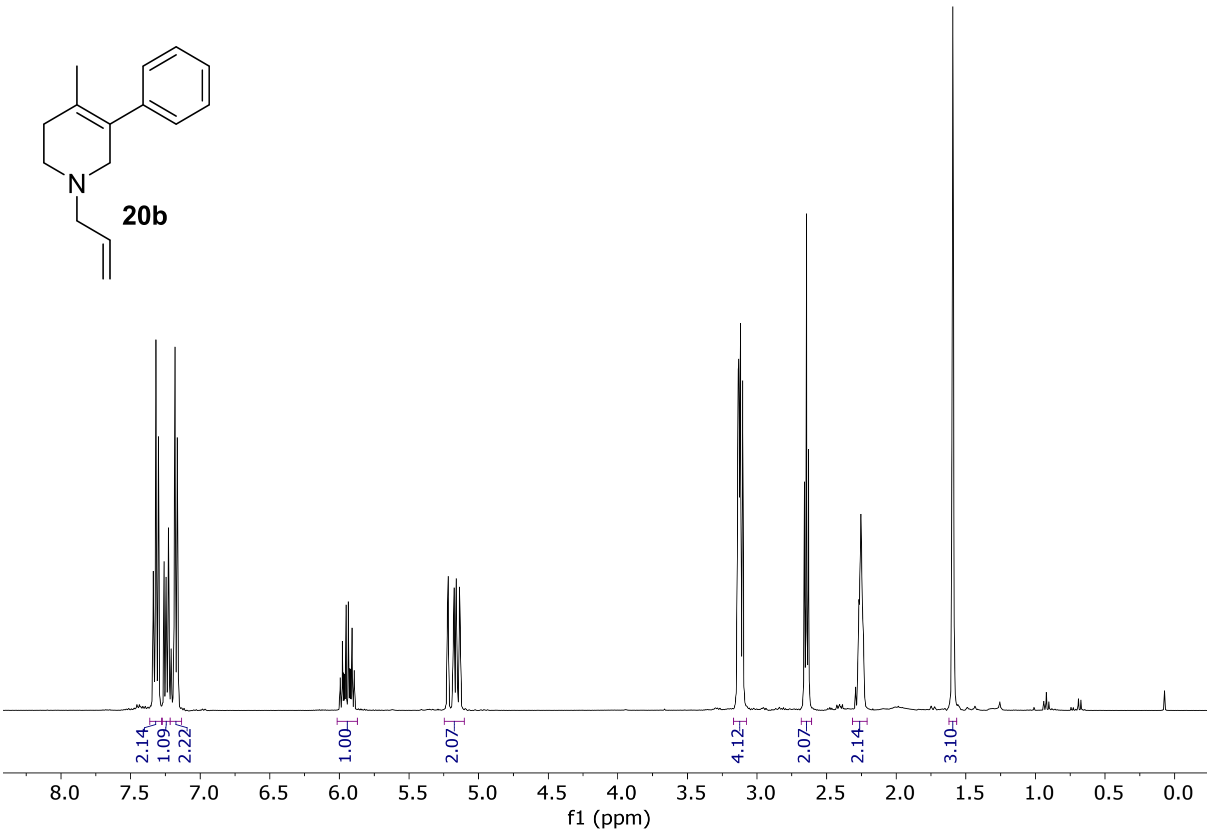

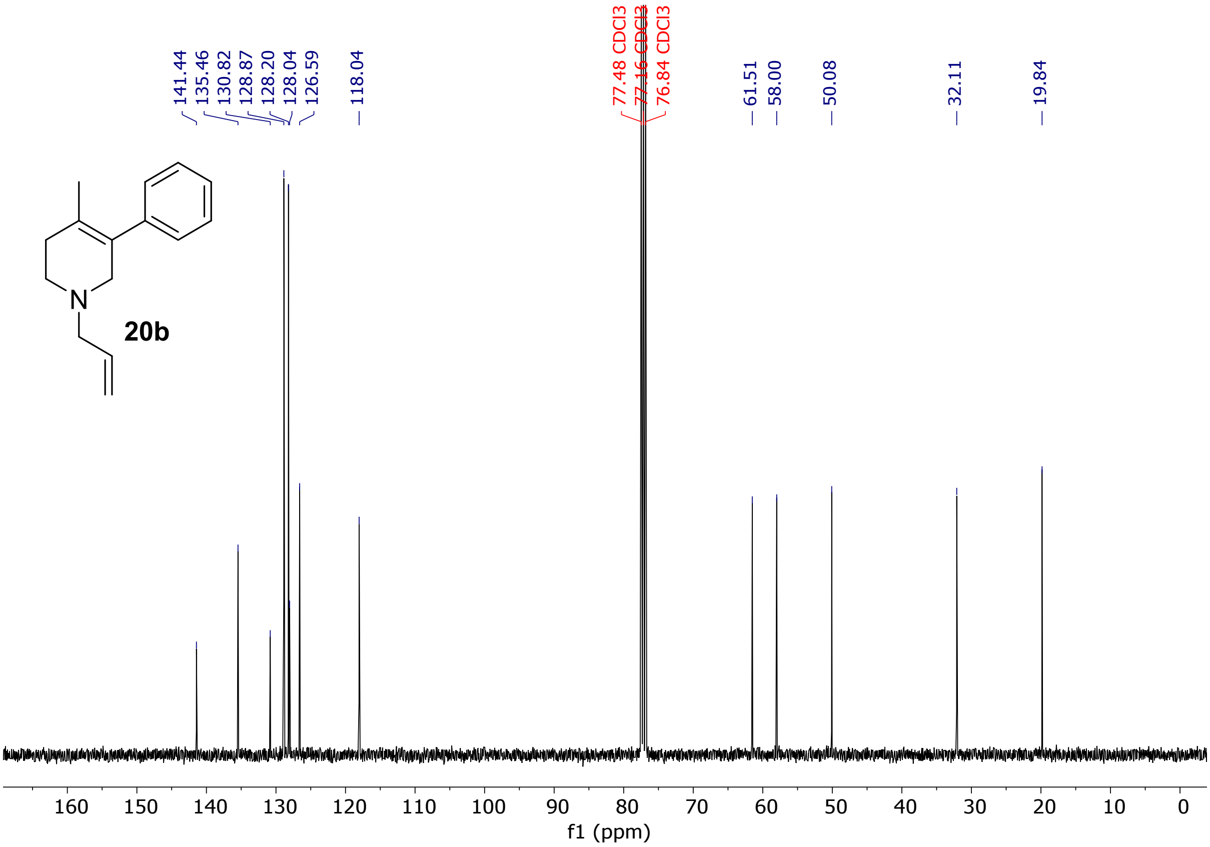

^{1}H & ^{13}C NMR spectra for 1-allyl-5-methyl-4-phenyl-1,2,3,6-THP (21b)

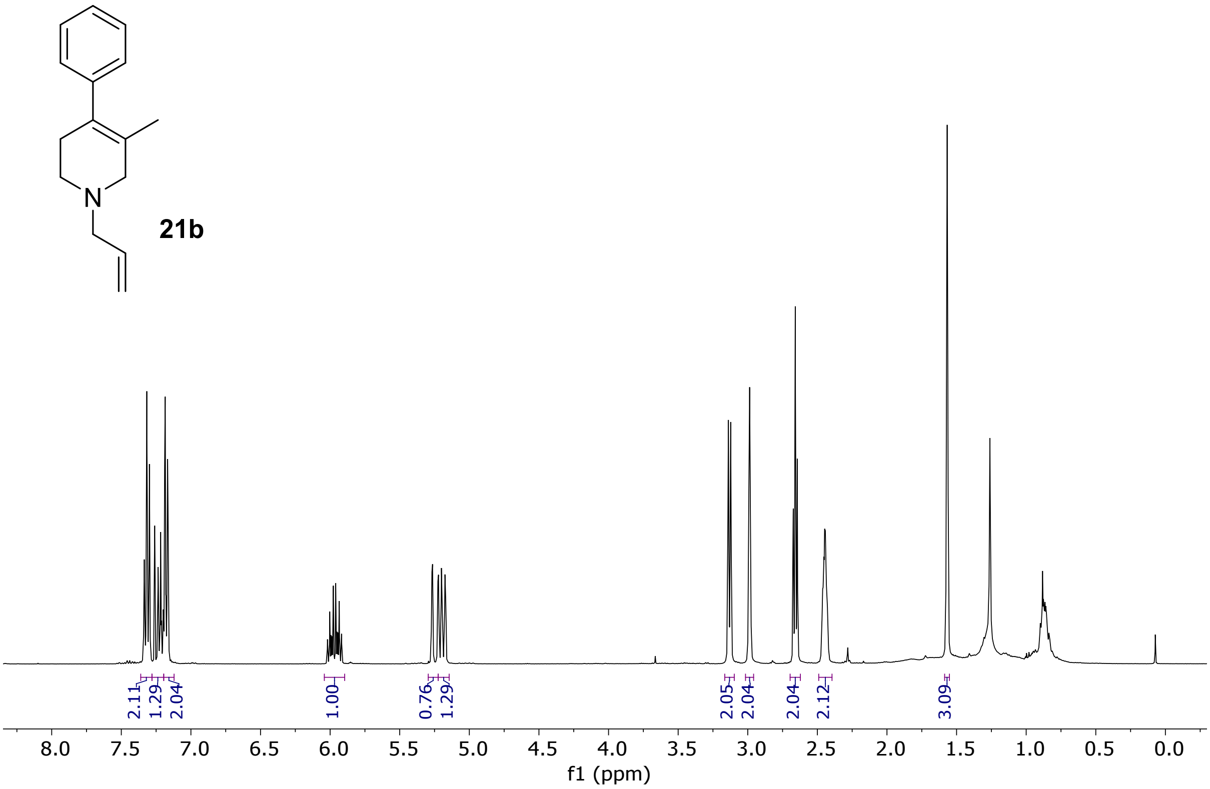

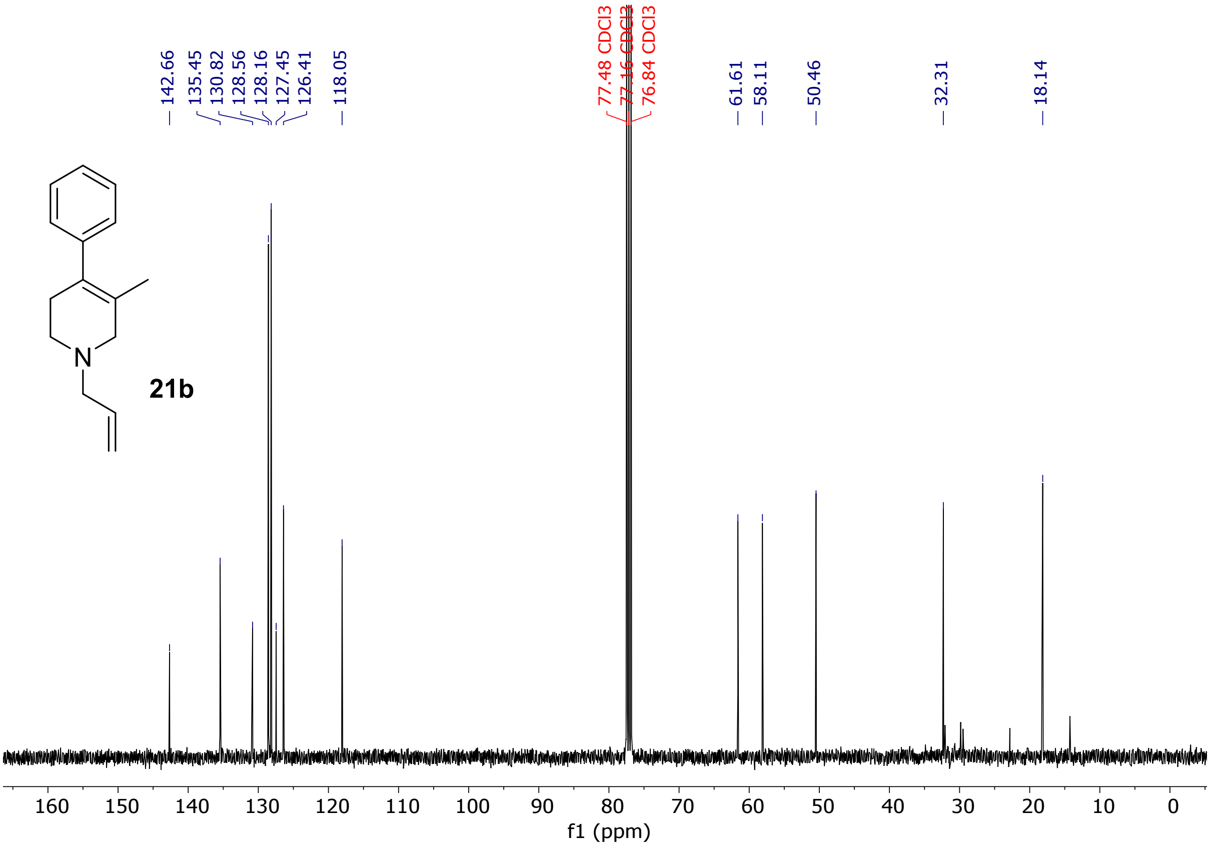

References

(1) Thorpe, T.W.; Marshall, J.R.; Harawa, V.; Ruscoe, R.E.; Cuetos, A.; Finnigan, J.D.; Angelastro, A.; Heath, R.S.; Parmeggiani, F.; Charnock, S.J.; Howard, R.M.; Daniels, D.S.B.; Grogan, G.; Turner, N.J. Multifunctional Biocatalyst for Conjugate Reduction and Reductive Amination. *Nature* **2022**, 604, 86-91.

(2) Chung, C. K.; Bulger, P. G.; Kosjek, B.; Belyk, K. M.; Rivera, N.; Scott, M. E.; Humphrey, G. R.; Limanto, J.; Bachert, D. C.; Emerson, K. M. Process Development of C–N Cross-Coupling and Enantioselective Biocatalytic Reactions for the Asymmetric Synthesis of Niraparib. *Org. Process Res. Dev.* **2014**, *18* (1), 215–227.

(3) Jones, P.; Altamura, S.; Boueres, J.; Ferrigno, F.; Fonsi, M.; Giomini, C.; Lamartina, S.; Monteagudo, E.; Ontoria, J. M.; Orsale, M. V.; Palumbi, M. C.; Pesci, S.; Roscilli, G.; Scarpelli, R.; Schultz-Fademrecht, C.; Toniatti, C.; Rowley, M. Discovery of 2-{4-[(3*S*)-Piperidin-3-yl]phenyl}-2*H*-Indazole-7-Carboxamide (MK-4827): A Novel Oral Poly(ADP-Ribose)Polymerase (PARP) Inhibitor Efficacious in BRCA-1 and -2 Mutant Tumors. *J. Med. Chem.* **2009**, *52* (22), 7170–7185.

(4) Atkin, K. E.; Reiss, R.; Koehler, V.; Bailey, K. R.; Hart, S.; Turkenburg, J. P.; Turner, N. J.; Brzozowski, A. M.; Grogan, G. The Structure of Monoamine Oxidase from Aspergillus Niger Provides a Molecular Context for Improvements in Activity Obtained by Directed Evolution. *J. Mol. Biol.* **2008**, *384* (5), 1218–1231.

(5) Rowles, I.; Malone, K. J.; Etchells, L. L.; Willies, S. C.; Turner, N. J. Directed Evolution of the Enzyme Monoamine Oxidase (MAO-N): Highly Efficient Chemo-Enzymatic Deracemisation of the Alkaloid (±)-Crispinea. *ChemCatChem* **2012**.

(6) Ghislieri, D.; Green, A. P.; Pontini, M.; Willies, S. C.; Rowles, I.; Frank, A.; Grogan, G.; Turner, N. J. Engineering an Enantioselective Amine Oxidase for the Synthesis of Pharmaceutical Building Blocks and Alkaloid Natural Products. *J. Am. Chem. Soc.* **2013**, *135* (29), 10863–10869.

(7) Kabsch, W. XDS. *Acta Crystallogr. Sect. D Biol. Crystallogr.* **2010**, *66* (2), 125–132.

(8) Evans, P. Scaling and Assessment of Data Quality. *Acta Crystallogr. Sect. D Biol. Crystallogr.* **2006**, *62* (1), 72–82.

(9) Winter, G. Xia2 : An Expert System for Macromolecular Crystallography Data Reduction. *J. Appl. Crystallogr.* **2010**, *43* (1), 186–190.

(10) Vagin, A.; Teplyakov, A. MOLREP : An Automated Program for Molecular Replacement. *J. Appl. Crystallogr.* **1997**, *30* (6), 1022–1025.

(11) Lenz, M.; Fademrecht, S.; Sharma, M.; Pleiss, J.; Grogan, G.; Nestl, B. M. New Imine-Reducing Enzymes from *β*-Hydroxyacid Dehydrogenases by Single Amino Acid Substitutions. *Protein Eng. Des. Sel.* **2018**, *31* (4), 109–120.

(12) Emsley, P.; Cowtan, K. Coot : Model-Building Tools for Molecular Graphics. *Acta Crystallogr. Sect. D Biol. Crystallogr.* **2004**, *60* (12), 2126–2132.

(13) Murshudov, G. N.; Vagin, A. A.; Dodson, E. J. Refinement of Macromolecular Structures by the Maximum-Likelihood Method. *Acta Crystallogr. Sect. D Biol. Crystallogr.* **1997**, *53* (3), 240–255.

(14) Trott, O.; Olson, A. J. AutoDock Vina: Improving the Speed and Accuracy of Docking with a New Scoring Function, Efficient Optimization, and Multithreading. *J. Comput. Chem.* **2010**, *31*, 455–461.

(15) Yang, J.; Liu, S.; Zheng, J.-F.; Zhou, J. S. Room-Temperature Suzuki-Miyaura Coupling of Heteroaryl Chlorides and Tosylates. *European J. Org. Chem.* **2012**, *2012* (31), 6248–6259.

(16) Baldwin, J. E.; Bischoff, L.; Claridge, T. D. W.; Heupel, F. A.; Spring, D. R.; Whitehead, R. C. An Approach to the Manzamine Alkaloids Modelled on a Biogenetic Theory. *Tetrahedron*

1997, *53* (6), 2271–2290.

(17) Renom-Carrasco, M.; Gajewski, P.; Pignataro, L.; de Vries, J. G.; Piarulli, U.; Gennari, C.; Lefort, L. Asymmetric Hydrogenation of 3-Substituted Pyridinium Salts. *Chem. - A Eur. J.* **2016**, *22* (28), 9528–9532.
